# Supplementary figures and images for: The association between depressive symptoms and antibody response following SARS-CoV-2 vaccination among central North Carolina residents
Source: PLOS Ment Health. 2025 Sep 17;2(9):e0000410. doi: 10.1371/journal.pmen.0000410 (PMC12448651; doi:10.1371/journal.pmen.0000410)

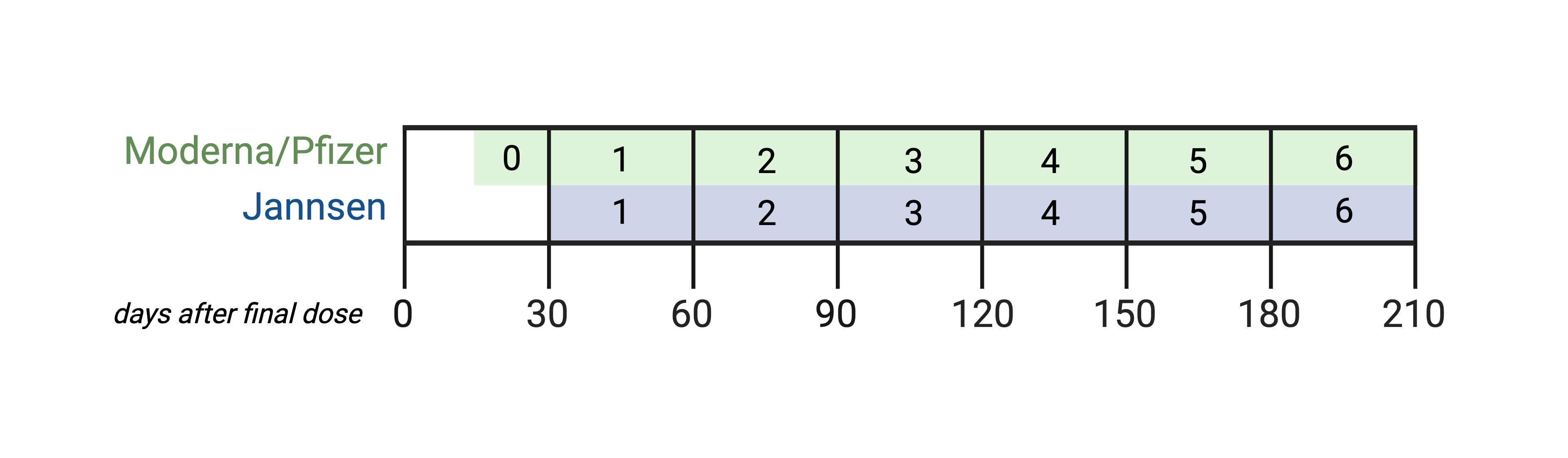

Supplement: S1 Fig — Created with BioRender.com. (PNG) [file pmen.0000410.s001.png]
