# Supplementary material for: The association between depressive symptoms and antibody response following SARS-CoV-2 vaccination among central North Carolina residents
Source: PLOS Ment Health. 2025 Sep 17;2(9):e0000410. doi: 10.1371/journal.pmen.0000410 (PMC12448651; doi:10.1371/journal.pmen.0000410)
Supplement: S1 Table — All covariates are binary. (DOCX) [file pmen.0000410.s002.docx]

**S1 Table.** Parameter estimates for all covariates included in primary analysis model. All covariates are binary.

| **Covariate** | **Estimate** | **95% CI** |
| --- | --- | --- |
| intercept | 19.348 | (10.399, 28.297) |
| months post vaccination "1" | -0.483 | (-5.638, 4.672) |
| months post vaccination "2" | -2.438 | (-6.151, 1.274) |
| months post vaccination "3" | -1.259 | (-5.144, 2.627) |
| months post vaccination "4" | -2.493 | (-6.084, 1.098) |
| months post vaccination "5" | -1.536 | (-5.694, 2.623) |
| months post vaccination "6" | -3.05 | (-7.838, 1.738) |
| depression (ref = "No") | -3.709 | (-12.454, 5.035) |
| months post vaccination "1" * depression | 4.832 | (-2.903, 12.567) |
| months post vaccination "2" * depression | 2.378 | (-3.006, 7.762) |
| months post vaccination "3" * depression | 2.594 | (-4.537, 9.724) |
| months post vaccination "4" * depression | 3.201 | (-2.82, 9.222) |
| months post vaccination "5" * depression | 5.817 | (-2.077, 13.712) |
| months post vaccination "6" * depression | 4.204 | (-4.674, 13.081) |
| sex (ref = "Female") | -1.559 | (-4.275, 1.158) |
| age_cat (ref = "<50") | -1.472 | (-5.06, 2.115) |
| smoke_cur (ref = "No") | -0.847 | (-10.161, 8.467) |
| alc_cur (ref = "No") | -1.628 | (-4.754, 1.498) |
| exercise (ref = "No") | 1.494 | (-4.862, 7.849) |
