# Supplementary material for: The association between depressive symptoms and antibody response following SARS-CoV-2 vaccination among central North Carolina residents
Source: PLOS Ment Health. 2025 Sep 17;2(9):e0000410. doi: 10.1371/journal.pmen.0000410 (PMC12448651; doi:10.1371/journal.pmen.0000410)

Data Dictionary Codebook

Aiello - Chatham County COVID-19 Cohort (C4) (PID: 2317)

08/07/2025 9:40am

Instruments | **Events**

#	Variable / Field Name	Field Label<br>*Field Note*	Field Attributes (Field Type, Validation, Choices, Calculations, etc.)
[redacted]			
**Instrument: C4 Baseline (c4_baseline)** Enabled as survey			
71	[comp_date]	Survey completion date:<br>[survey-date-completed:c4_baseline]	descriptive<br>Field Annotation: @HIDDEN-SURVEY
72	[fname]	What is your first name? / Cual es su nombre preferido?	text
73	[lname]	What is your last name? / Cual es su appelido?	text
74	[dob]	What is your date of birth? / Cual es su fecha del nacimiento?	text (date_mdy), Required
75	[age]	Participant age:	calc<br>Calculation: rounddown(datediff([dob], 'today', 'y'))
76	[sex]	What is your sex? / Cual es su sexo?	radio, Required<br>1 Female / Mujer<br>2 Male / Hombre<br>3 Prefer not to answer / Prefiero no responder
77	[language]	Would you prefer to take this survey in English or Spanish? / Preferie responder esta encuesta en ingles o espanol?	radio<br>1 English / ingles<br>2 Spanish / espanol
78	[race_e]<br><br>Show the field ONLY if:<br>[language] = '1'	What is your race?<br>*Select all that apply*	checkbox, Required<br>1 race_e___1 American Indian or Alaska Native<br>2 race_e___2 Asian<br>3 race_e___3 Black or African American<br>4 race_e___4 Native Hawaiian or Pacific Islander<br>5 race_e___5 White<br>6 race_e___6 Other<br>7 race_e___7 Prefer not to answer
79	[ethn_e]<br><br>Show the field ONLY if:<br>[language] = '1'	What is your ethnicity?	radio, Required<br>1 Hispanic or Latino<br>2 Not Hispanic or Latino<br>3 Other<br>4 Prefer not to answer

80	[edu_e]<br><br>Show the field ONLY if:<br>[language] = '1'	What is the highest level of education or schooling you have completed?	radio<br>1 never attended school<br>2 kindergarten - 8th grade<br>3 some high school<br>4 high school equivalency (GED)<br>5 high school graduate<br>6 some college<br>7 college graduate<br>8 graduate school or more
81	[smoke_cur_e]<br><br>Show the field ONLY if:<br>[language] = '1'	Do you currently smoke cigarettes, cigars, or a pipe on a daily basis?	yesno<br>1 Yes<br>0 No
82	[smoke_cur2_e]<br><br>Show the field ONLY if:<br>[language] = '1' and [smoke_cur_e] = '1'	How old were you when you first started to smoke fairly regularly?	text (number, Min: 0, Max: 100)
83	[smoke_cur3_e]<br><br>Show the field ONLY if:<br>[language] = '1' and [smoke_cur_e] = '1'	What is the average number of cigarettes, cigars, and/or pipes smoked per day since you began smoking?	radio<br>0 none<br>1 0-10<br>2 11-20<br>3 21-30<br>4 31-40<br>5 41-50<br>6 51-60<br>7 61 or more
84	[smoke_past_e]<br><br>Show the field ONLY if:<br>[language] = '1' and [smoke_cur_e] = '0'	Did you previously smoke cigarettes, cigars, or a pipe on a daily basis?	yesno<br>1 Yes<br>0 No
85	[smoke_past2_e]<br><br>Show the field ONLY if:<br>[language] = '1' and [smoke_past_e] = '1'	How old were you when you first started to smoke fairly regularly?	text (number, Min: 0, Max: 100)
86	[smoke_past3_e]<br><br>Show the field ONLY if:<br>[language] = '1' and [smoke_past_e] = '1'	How many years has it been since you quit smoking?	text (number, Min: 0, Max: 100)
87	[smoke_past4_e]<br><br>Show the field ONLY if:<br>[language] = '1' and [smoke_past_e] = '1'	What was the average number of cigarettes, cigars, and/or pipes smoked per day when you were smoking?	radio<br>0 none<br>1 0-10<br>2 11-20<br>3 21-30<br>4 31-40<br>5 41-50

			6 51-60<br>7 61 or more
88	[vape_cur_e]<br>Show the field ONLY if:<br>[language] = '1'	Do you currently use electronic cigarettes (e-cigarettes, vaping)?	yesno<br>1 Yes<br>0 No
89	[vape_cur2_e]<br>Show the field ONLY if:<br>[language] = '1' and [vape_cur_e] = '1'	How old were you when you first started to use electronic cigarettes fairly regularly?	text (number, Min: 0, Max: 100)
90	[vape_cur3_e]<br>Show the field ONLY if:<br>[language] = '1' and [vape_cur_e] = '1'	What is the average number of e-cigarette (or other vaping product) puffs you inhale per day?	radio<br>0 0-25<br>1 26-50<br>2 51-75<br>3 76-100<br>4 101-125<br>5 126-150<br>6 151-175<br>7 176-200<br>8 201-225<br>9 226-250<br>10 251 or more
91	[vape_past_e]<br>Show the field ONLY if:<br>[language] = '1' and [vape_cur_e] = '0'	Did you previously use electronic cigarettes (e-cigarettes, vaping)?	yesno<br>1 Yes<br>0 No
92	[vape_past2_e]<br>Show the field ONLY if:<br>[language] = '1' and [vape_past_e] = '1'	How old were you when you first started to use electronic cigarettes fairly regularly?	text (number, Min: 0, Max: 100)
93	[vape_past3_e]<br>Show the field ONLY if:<br>[language] = '1' and [vape_past_e] = '1'	How many years has it been since you quit using electronic cigarettes?	text (number, Min: 0, Max: 100)
94	[vape_past4_e]<br>Show the field ONLY if:<br>[language] = '1' and [vape_past_e] = '1'	What was the average number of e-cigarette (or other vaping product) puffs you inhale per day?	radio<br>0 0-25<br>1 26-50<br>2 51-75<br>3 76-100<br>4 101-125<br>5 126-150<br>6 151-175<br>7 176-200<br>8 201-225<br>9 226-250

			10 251 or more
95	[alc_cur_e]<br><br>Show the field ONLY if:<br>[language] = '1'	Do you currently drink alcohol at least once a week?	yesno<br>1 Yes<br>0 No
96	[alc_cur2_e]<br><br>Show the field ONLY if:<br>[language] = '1' and [alc_cur_e] = '1'	How old were you when you first started to drink alcohol fairly regularly?	text (number, Min: 0, Max: 100)
97	[alc_cur3_e]<br><br>Show the field ONLY if:<br>[language] = '1' and [alc_cur_e] = '1'	Think specifically about the last 30 days, including today. During the last 30 days, on how many days did you drink one or more drinks of an alcoholic beverage?	text (number, Min: 0, Max: 30)
98	[alc_cur4_e]<br><br>Show the field ONLY if:<br>[language] = '1' and [alc_cur_e] = '1' and [alc_cur3_e] > '0'	On the days that you drank during the past 30 days, how many drinks did you usually have each day?<br>*One drink is equal to 5 ounces of wine (one glass of wine), 12 ounces of beer (one can or bottle of beer), or 1.5 ounces of liquor (one shot or mixed drink)*	text (number, Min: 0, Max: 30)
99	[alc_past_e]<br><br>Show the field ONLY if:<br>[language] = '1' and [alc_cur_e] = '0'	Did you previously drink alcohol at least once a week?	yesno<br>1 Yes<br>0 No
100	[alc_past2_e]<br><br>Show the field ONLY if:<br>[language] = '1' and [alc_past_e] = '1'	How old were you when you first started to drink alcohol fairly regularly?	text (number, Min: 0, Max: 100)
101	[alc_past3_e]<br><br>Show the field ONLY if:<br>[language] = '1' and [alc_past_e] = '1'	How many years has it been since you quit drinking alcohol?	text (number, Min: 0, Max: 100)
102	[alc_past4_e]<br><br>Show the field ONLY if:<br>[language] = '1' and [alc_past_e] = '1'	During a normal 30 day period, on how many days did you drink one or more drinks of an alcoholic beverage?	text (number, Min: 0, Max: 30)
103	[alc_past5_e]<br><br>Show the field ONLY if:<br>[language] = '1' and [alc_past_e] = '1' and [alc_past4_e] > '0'	On the days that you drank, how many drinks did you usually have each day?<br>*One drink is equal to 5 ounces of wine (one glass of wine), 12 ounces of beer (one can or bottle of beer), or 1.5 ounces of liquor (one shot or mixed drink)*	text (number, Min: 0, Max: 30)
104	[exercise_e]<br><br>Show the field ONLY if:<br>[language] = '1'	At least once a week, do you engage in regular activity like brisk walking, jogging, bicycling, swimming, etc. long enough to work up a sweat, get your heart thumping, or get out of breath?	yesno<br>1 Yes<br>0 No
105	[exercise2_e]<br><br>Show the field ONLY if:<br>[language] = '1' and [exercise_e] = "1"	On average, how many days per week do you engage in this kind of exercise?	radio<br>1 1<br>2 2<br>3 3<br>4 4<br>5 5

			6 6<br>7 7
106	[exercise3_e]<br><br>Show the field ONLY if:<br>[language] = '1' and [exercise_e] = "1"	On average, how many minutes per day do you engage in this kind of exercise?	radio<br>0 0-20<br>1 21-40<br>2 41-60<br>3 61 or more
107	[exercise4_e]<br><br>Show the field ONLY if:<br>[language] = '1' and [exercise_e] = "1"	When you are exercising in your usual fashion, how would you rate your average level of exertion (degree of effort)?	radio<br>0 Easy / Warm-up<br>1 Medium (can hold a conversation) / Aerobic Development<br>2 Hard (but you can push yourself to continue) / Aerobic Endurance<br>3 Very Hard (cannot hold a conversation) / Anaerobic Endurance<br>4 Extremely Hard (out of breath, your body wants to stop) / Speed, Power
108	[work_e]<br><br>Show the field ONLY if:<br>[language] = '1'	Before the COVID-19 pandemic began in North Carolina, which of the following best fit your work situation?	radio<br>1 worked full time<br>2 worked part time<br>3 was looking for work/employment<br>4 retired<br>5 homemaker<br>6 student<br>7 on maternity/paternity leave<br>8 on illness/sick leave<br>9 on disability<br>10 other
109	[work2_e]<br><br>Show the field ONLY if:<br>[language] = '1'	Before the COVID-19 pandemic began in North Carolina, did you consider yourself self-employed (including as an independent contractor or gig-economy worker)?	radio<br>1 yes<br>0 no<br>2 don't know
110	[work3_e]<br><br>Show the field ONLY if:<br>[language] = '1' and ([work_e] = '1' or [work_e] = '2' or [work2_e] = '1')	Of the job (or jobs) that you held before the COVID-19 pandemic in North Carolina, which description best described your main job (i.e. the job you spent the most hours at, or the job at which you had worked the longest)?	radio<br>1 managerial<br>2 professional<br>3 administrative support<br>4 service<br>5 farming/forestry/fishing<br>6 precision production/craft/repair<br>7 operators/fabricators/laborers<br>8 military<br>9 student<br>10 not working

			11 other
111	[work4_e]<br>Show the field ONLY if:<br>[language] = '1' and ([work_e] = '1' or [work_e] = '2' or [work2_e] = '1')	How many years had you spent at your main job?	text (number, Min: 0, Max: 70)
112	[work5_e]<br>Show the field ONLY if:<br>[language] = '1' and ([work_e] = '1' or [work_e] = '2' or [work2_e] = '1')	In your main job before the COVID-19 pandemic, did your employer offer you any of the following benefits?<br>*Select all that apply.*	checkbox<br>1 work5_e___1 paid sick leave<br>2 work5_e___2 paid vacation/personal leave<br>3 work5_e___3 health insurance<br>4 work5_e___4 disability insurance<br>5 work5_e___5 retirement plan<br>6 work5_e___6 other
113	[work6_e]<br>Show the field ONLY if:<br>[language] = '1' and ([work_e] = '1' or [work_e] = '2' or [work2_e] = '1')	Has your work situation changed since the COVID-19 pandemic began in North Carolina?	yesno<br>1 Yes<br>0 No
114	[work7_e]<br>Show the field ONLY if:<br>[language] = '1' and [work6_e] = '1'	Which of the following best fits your current work situation?	radio<br>1 works full time<br>2 works part time<br>3 is looking for work/employment<br>4 retired<br>5 homemaker<br>6 student<br>7 on maternity/paternity leave<br>8 on illness/sick leave<br>9 on disability<br>10 other
115	[work8_e]<br>Show the field ONLY if:<br>[language] = '1' and [work6_e] = '1'	Do you currently consider yourself self-employed (including as an independent contractor or gig-economy worker)?	radio<br>1 yes<br>0 no<br>2 don't know
116	[work9_e]<br>Show the field ONLY if:<br>[language] = '1' and [work6_e] = '1'	Of the job (or jobs) that you currently hold, which description best describes your main job (i.e. the job you spend the most hours at, or the job at which you have worked the longest)?	radio<br>1 managerial<br>2 professional<br>3 administrative support<br>4 service<br>5 farming/forestry/fishing<br>6 precision production/craft/repair<br>7 operators/fabricators/laborers<br>8 military<br>9 student

			10 not working<br>11 other
117	[work10_e]<br><br>Show the field ONLY if:<br>[language] = '1' and [work6_e] = '1'	How many months have you spent at your current main job?	text (number, Min: 0, Max: 70)
118	[work11_e]<br><br>Show the field ONLY if:<br>[language] = '1' and [work6_e] = '1'	In your current main job, does your employer offer you any of the following benefits?<br>*Select all that apply.*	radio<br>1 paid sick leave<br>2 paid vacation/personal leave<br>3 health insurance<br>4 disability insurance<br>5 retirement plan<br>6 other
119	[work12_e]<br><br>Show the field ONLY if:<br>[language] = '1' and ([work_e] = '1' or [work_e] = '2' or [work2_e] = '1') and [work6_e] = '0'	On a scale of 0 (definitely not going to happen) to 10 (definitely going to happen), how likely is it that you will lose your job because of the COVID-19 pandemic?	text (number, Min: 0, Max: 10)
120	[work13_e]<br><br>Show the field ONLY if:<br>[language] = '1' and ([work_e] = '1' or [work_e] = '2' or [work2_e] = '1') and [work6_e] = '0'	On a scale of 0 (definitely not going to happen) to 10 (definitely going to happen), how likely is it that you will receive fewer work hours at your job because of the COVID-19 pandemic?	text (number, Min: 0, Max: 10)
121	[work14_e]<br><br>Show the field ONLY if:<br>[language] = '1' and ([work_e] = '1' or [work_e] = '2' or [work2_e] = '1')	How often were you required to work from outside of the home before the COVID-19 pandemic in North Carolina?	radio (Matrix)<br>1 always (100%)<br>2 most of the time (75%)<br>3 half of the time (50%)<br>4 less than half of the time (25%)<br>5 never (0%)
122	[work15_e]<br><br>Show the field ONLY if:<br>[language] = '1' and ([work14_e] = '1' or [work14_e] = '2' or [work14_e] = '3' or [work14_e] = '4')	Before the COVID-19 pandemic, how regularly were you in close physical contact with co-workers during your work outside of the home?	radio (Matrix)<br>1 always (100%)<br>2 most of the time (75%)<br>3 half of the time (50%)<br>4 less than half of the time (25%)<br>5 never (0%)
123	[work16_e]<br><br>Show the field ONLY if:<br>[language] = '1' and ([work14_e] = '1' or [work14_e] = '2' or [work14_e] = '3' or [work14_e] = '4')	Before the COVID-19 pandemic, how regularly were you in close physical contact with clients during your work outside of the home?	radio (Matrix)<br>1 always (100%)<br>2 most of the time (75%)<br>3 half of the time (50%)<br>4 less than half of the time (25%)<br>5 never (0%)

124	[work17_e]<br><br>Show the field ONLY if: [language] = '1' and ([work14_e] = '1' or [work14_e] = '2' or [work14_e] = '3' or [work14_e] = '4')	Before the COVID-19 pandemic, how often did you have access to disposable gloves during your work outside of the home?	radio (Matrix)<br>1 always (100%)<br>2 most of the time (75%)<br>3 half of the time (50%)<br>4 less than half of the time (25%)<br>5 never (0%)
125	[work18_e]<br><br>Show the field ONLY if: [language] = '1' and ([work14_e] = '1' or [work14_e] = '2' or [work14_e] = '3' or [work14_e] = '4')	Before the COVID-19 pandemic, how often did you have access to a face mask during your work outside of the home?	radio (Matrix)<br>1 always (100%)<br>2 most of the time (75%)<br>3 half of the time (50%)<br>4 less than half of the time (25%)<br>5 never (0%)
126	[work19_e]<br><br>Show the field ONLY if: [language] = '1' and ([work14_e] = '1' or [work14_e] = '2' or [work14_e] = '3' or [work14_e] = '4')	Before the COVID-19 pandemic, how often did you use disposable gloves during your work outside of the home?	radio (Matrix)<br>1 always (100%)<br>2 most of the time (75%)<br>3 half of the time (50%)<br>4 less than half of the time (25%)<br>5 never (0%)
127	[work20_e]<br><br>Show the field ONLY if: [language] = '1' and ([work14_e] = '1' or [work14_e] = '2' or [work14_e] = '3' or [work14_e] = '4')	Before the COVID-19 pandemic, how often did you use a face mask during your work outside of the home?	radio (Matrix)<br>1 always (100%)<br>2 most of the time (75%)<br>3 half of the time (50%)<br>4 less than half of the time (25%)<br>5 never (0%)
128	[work21_e]<br><br>Show the field ONLY if: [language] = '1' and ([work14_e] = '1' or [work14_e] = '2' or [work14_e] = '3' or [work14_e] = '4')	Before the COVID-19 pandemic, how often did you wash your hands with soap and water during your work outside of the home?	radio (Matrix)<br>1 always (100%)<br>2 most of the time (75%)<br>3 half of the time (50%)<br>4 less than half of the time (25%)<br>5 never (0%)
129	[work22_e]<br><br>Show the field ONLY if: [language] = '1' and ([work14_e] = '1' or [work14_e] = '2' or [work14_e] = '3' or [work14_e] = '4')	Before the COVID-19 pandemic, how often did you sanitize your hands with hand sanitizer during your work outside of the home?	radio (Matrix)<br>1 always (100%)<br>2 most of the time (75%)<br>3 half of the time (50%)<br>4 less than half of the time (25%)<br>5 never (0%)
130	[work23_e]<br><br>Show the field ONLY if: [language] = '1' and ([work14_e] = '1' or [work14_e] = '2' or [work14_e] = '3' or [work14_e] = '4')	Before the COVID-19 pandemic, how worried were you that you would be exposed to COVID-19 during your work outside of the home?	radio (Matrix)<br>1 always (100%)<br>2 most of the time (75%)<br>3 half of the time (50%)<br>4 less than half of the time (25%)<br>5 never (0%)

131	[work24_e]<br>Show the field ONLY if:<br>[language] = '1'	How often are you required to work from outside of the home currently?	radio (Matrix)<br>1 always (100%)<br>2 most of the time (75%)<br>3 half of the time (50%)<br>4 less than half of the time (25%)<br>5 never (0%)
132	[work25_e]<br>Show the field ONLY if:<br>[language] = '1' and ([work24_e] = '1' or [work24_e] = '2' or [work24_e] = '3' or [work24_e] = '4')	How regularly are you in close physical contact with co-workers during your work outside of the home currently?	radio (Matrix)<br>1 always (100%)<br>2 most of the time (75%)<br>3 half of the time (50%)<br>4 less than half of the time (25%)<br>5 never (0%)
133	[work26_e]<br>Show the field ONLY if:<br>[language] = '1' and ([work24_e] = '1' or [work24_e] = '2' or [work24_e] = '3' or [work24_e] = '4')	How regularly are you in close physical contact with clients during your work outside of the home currently?	radio (Matrix)<br>1 always (100%)<br>2 most of the time (75%)<br>3 half of the time (50%)<br>4 less than half of the time (25%)<br>5 never (0%)
134	[work27_e]<br>Show the field ONLY if:<br>[language] = '1' and ([work24_e] = '1' or [work24_e] = '2' or [work24_e] = '3' or [work24_e] = '4')	How often do you have access to disposable gloves during your work outside of the home currently?	radio (Matrix)<br>1 always (100%)<br>2 most of the time (75%)<br>3 half of the time (50%)<br>4 less than half of the time (25%)<br>5 never (0%)
135	[work28_e]<br>Show the field ONLY if:<br>[language] = '1' and ([work24_e] = '1' or [work24_e] = '2' or [work24_e] = '3' or [work24_e] = '4')	How often do you have access to a face mask during your work outside of the home currently?	radio (Matrix)<br>1 always (100%)<br>2 most of the time (75%)<br>3 half of the time (50%)<br>4 less than half of the time (25%)<br>5 never (0%)
136	[work29_e]<br>Show the field ONLY if:<br>[language] = '1' and ([work24_e] = '1' or [work24_e] = '2' or [work24_e] = '3' or [work24_e] = '4')	How often do you use disposable gloves during your work outside of the home currently?	radio (Matrix)<br>1 always (100%)<br>2 most of the time (75%)<br>3 half of the time (50%)<br>4 less than half of the time (25%)<br>5 never (0%)
137	[work30_e]<br>Show the field ONLY if:<br>[language] = '1' and ([work24_e] = '1' or [work24_e] = '2' or [work24_e] = '3' or [work24_e] = '4')	How often do you use a face mask during your work outside of the home currently?	radio (Matrix)<br>1 always (100%)<br>2 most of the time (75%)<br>3 half of the time (50%)<br>4 less than half of the time (25%)<br>5 never (0%)

138	[work31_e]<br><br>Show the field ONLY if:<br>[language] = '1' and ([work24_e] = '1' or [work24_e] = '2' or [work24_e] = '3' or [work24_e] = '4')	How often do you wash your hands with soap and water during your work outside of the home currently?	radio (Matrix)<br>1 always (100%)<br>2 most of the time (75%)<br>3 half of the time (50%)<br>4 less than half of the time (25%)<br>5 never (0%)
139	[work32_e]<br><br>Show the field ONLY if:<br>[language] = '1' and ([work24_e] = '1' or [work24_e] = '2' or [work24_e] = '3' or [work24_e] = '4')	How often do you sanitize your hands with hand sanitizer during your work outside of the home currently?	radio (Matrix)<br>1 always (100%)<br>2 most of the time (75%)<br>3 half of the time (50%)<br>4 less than half of the time (25%)<br>5 never (0%)
140	[work33_e]<br><br>Show the field ONLY if:<br>[language] = '1' and ([work24_e] = '1' or [work24_e] = '2' or [work24_e] = '3' or [work24_e] = '4')	How worried are you that you will be exposed to COVID-19 during your work outside of the home currently?	radio (Matrix)<br>1 always (100%)<br>2 most of the time (75%)<br>3 half of the time (50%)<br>4 less than half of the time (25%)<br>5 never (0%)
141	[work34_e]<br><br>Show the field ONLY if:<br>[language] = '1' and ([work_e] = '1' or [work_e] = '2' or [work2_e] = '1')	Do you currently work in any of the following high-risk settings for COVID-19 transmission?	checkbox<br>1 work34_e___1 healthcare setting (hospital, clinic, urgent care, etc.)<br>2 work34_e___2 dense residential setting (nursing home, other long-term care facility)<br>3 work34_e___3 prison or jail<br>4 work34_e___4 meatpacking facility<br>5 work34_e___5 shipping or distribution facility<br>6 work34_e___6 high-volume retail facility (grocery store, etc.)
142	[height_e]<br><br>Show the field ONLY if:<br>[language] = '1'	What is your height?<br>*Record your height in feet and inches (example: 5'10)*	text
143	[weight_e]<br><br>Show the field ONLY if:<br>[language] = '1'	What is your weight?<br>*Record your weight in pounds (example: 145)*	text
144	[insur_e]<br><br>Show the field ONLY if:<br>[language] = '1'	Are you covered by any type of medical or health insurance (including private insurance, insurance your purchased, Medicare, Medicaid, or any other health insurance program)?	radio<br>1 yes<br>0 no<br>2 don't know
145	[insur2_e]<br><br>Show the field ONLY if:<br>[language] = '1' and [insur_e] = '1'	What is the primary health insurance coverage that you have?<br>*Select one (your primary insurance).*	radio<br>1 Private health insurance through a job or school

			2 Insurance purchased through a state or federal health insurance exchange, such as healthcare.gov<br>3 Insurance purchased directly through a health plan or insurance company<br>4 Medicare<br>5 Medi-Gap<br>6 Medicaid<br>7 Military health care (TRICARE, VA, CHAMP-VA, etc.)<br>8 Indian Health Service<br>9 Other
146	[insur3_e]<br>Show the field ONLY if:<br>[language] = '1' and [insur2_e] = '9'	Please specify your other source of health insurance	text
147	[allergies_e]<br>Show the field ONLY if:<br>[language] = '1'	Section Header: *Have you ever been given a diagnosis of any of the following?*<br>seasonal allergies	radio (Matrix)<br>1 yes<br>0 no
148	[asthma_e]<br>Show the field ONLY if:<br>[language] = '1'	asthma	radio (Matrix)<br>1 yes<br>0 no
149	[diabetes_e]<br>Show the field ONLY if:<br>[language] = '1'	diabetes	radio (Matrix)<br>1 yes<br>0 no
150	[htn_e]<br>Show the field ONLY if:<br>[language] = '1'	hypertension	radio (Matrix)<br>1 yes<br>0 no
151	[cvd_e]<br>Show the field ONLY if:<br>[language] = '1'	cardiovascular disease (heart attack, heart failure, angina, etc.)	radio (Matrix)<br>1 yes<br>0 no
152	[cancer_e]<br>Show the field ONLY if:<br>[language] = '1'	cancer	radio (Matrix)<br>1 yes<br>0 no
153	[crd_e]<br>Show the field ONLY if:<br>[language] = '1'	chronic lung or respiratory disease (COPD, emphysema, bronchitis, etc.)	radio (Matrix)<br>1 yes<br>0 no
154	[kidney_e]<br>Show the field ONLY if:<br>[language] = '1'	chronic kidney disease	radio (Matrix)<br>1 yes<br>0 no
155	[liver_e]<br>Show the field ONLY if:<br>[language] = '1'	chronic liver disease (cirrhosis, etc.)	radio (Matrix)<br>1 yes<br>0 no

156	[immune_e]<br><br>Show the field ONLY if:<br>[language] = '1'	weakened immune system (HIV, chronic corticosteroid treatment, organ transplant, etc.)	radio (Matrix)<br>1 yes<br>0 no
157	[other_chronic_e]<br><br>Show the field ONLY if:<br>[language] = '1'	other chronic condition	radio (Matrix)<br>1 yes<br>0 no
158	[other_chronic2_e]<br><br>Show the field ONLY if:<br>[language] = '1' and [other_chronic_e] = '1'	Please identify what other chronic medical condition you have been diagnosed with.	text
159	[pregnant_e]<br><br>Show the field ONLY if:<br>[language] = '1' and [sex] = '1'	Are you currently pregnant?	yesno<br>1 Yes<br>0 No
160	[prev_covid_e]<br><br>Show the field ONLY if:<br>[language] = '1'	Have you previously been diagnosed with COVID-19?	radio<br>1 Yes<br>0 No<br><br>Field Annotation: @HIDDEN
161	[prev_covid2_e]<br><br>Show the field ONLY if:<br>[language] = '1'	When were you previously diagnosed with COVID-19?<br>*Please provide your best guess as to your date of testing, or if not tested for COVID-19, then your best guess as to the date when you were diagnosed by a clinician.*	text (date_mdy)<br>Field Annotation: @HIDDEN
162	[srh_e]<br><br>Show the field ONLY if:<br>[language] = '1'	In general, how would you have rated your health before the COVID-19 pandemic?	radio (Matrix)<br>1 excellent<br>2 very good<br>3 good<br>4 fair<br>5 poor
163	[srh2_e]<br><br>Show the field ONLY if:<br>[language] = '1'	In general, how would you rate your health over the last two weeks?	radio (Matrix)<br>1 excellent<br>2 very good<br>3 good<br>4 fair<br>5 poor
164	[fluvaccine_e]<br><br>Show the field ONLY if:<br>[language] = '1'	Did you receive a flu vaccine this flu season (2019-2020)?	radio<br>1 yes<br>0 no<br>2 don't know
165	[fluvaccine2_e]<br><br>Show the field ONLY if:<br>[language] = '1'	How often do you get a flu vaccine?	radio<br>1 every flu season<br>2 most flu seasons<br>3 half of the flu seasons<br>4 less than half of the flu seasons<br>5 never

166	[prevent_e]<br><br>Show the field ONLY if:<br>[language] = '1'	Section Header: *How often have you done the following things to protect yourself from infection since the beginning of the COVID-19 pandemic in North Carolina?*<br><br>Worn a face mask	radio (Matrix)<br>1 always (100%)<br>2 most of the time (75%)<br>3 half of the time (50%)<br>4 less than half of the time (25%)<br>5 never (0%)
167	[prevent2_e]<br><br>Show the field ONLY if:<br>[language] = '1'	Washed hands and/or used sanitizer frequently	radio (Matrix)<br>1 always (100%)<br>2 most of the time (75%)<br>3 half of the time (50%)<br>4 less than half of the time (25%)<br>5 never (0%)
168	[prevent3_e]<br><br>Show the field ONLY if:<br>[language] = '1'	Stayed at least 6 feet away from others	radio (Matrix)<br>1 always (100%)<br>2 most of the time (75%)<br>3 half of the time (50%)<br>4 less than half of the time (25%)<br>5 never (0%)
169	[prevent4_e]<br><br>Show the field ONLY if:<br>[language] = '1'	Avoided large gatherings, public spaces, or crowds	radio (Matrix)<br>1 always (100%)<br>2 most of the time (75%)<br>3 half of the time (50%)<br>4 less than half of the time (25%)<br>5 never (0%)
170	[prevent5_e]<br><br>Show the field ONLY if:<br>[language] = '1'	Avoided contact with people who could be high risk	radio (Matrix)<br>1 always (100%)<br>2 most of the time (75%)<br>3 half of the time (50%)<br>4 less than half of the time (25%)<br>5 never (0%)
171	[prevent6_e]<br><br>Show the field ONLY if:<br>[language] = '1'	Avoided food from restaurants, including takeout	radio (Matrix)<br>1 always (100%)<br>2 most of the time (75%)<br>3 half of the time (50%)<br>4 less than half of the time (25%)<br>5 never (0%)
172	[prevent7_e]<br><br>Show the field ONLY if:<br>[language] = '1'	Worked or studied at home instead of going into an office/classroom	radio (Matrix)<br>1 always (100%)<br>2 most of the time (75%)<br>3 half of the time (50%)<br>4 less than half of the time (25%)<br>5 never (0%)

173	[prevent8_e]<br><br>Show the field ONLY if:<br>[language] = '1'	Avoided shaking hands or touching people	radio (Matrix)<br>1 always (100%)<br>2 most of the time (75%)<br>3 half of the time (50%)<br>4 less than half of the time (25%)<br>5 never (0%)
174	[prevent9_e]<br><br>Show the field ONLY if:<br>[language] = '1'	Stayed home when you were sick	radio (Matrix)<br>1 always (100%)<br>2 most of the time (75%)<br>3 half of the time (50%)<br>4 less than half of the time (25%)<br>5 never (0%)
175	[prevent10_e]<br><br>Show the field ONLY if:<br>[language] = '1'	Wiped down surfaces with disinfectant	radio (Matrix)<br>1 always (100%)<br>2 most of the time (75%)<br>3 half of the time (50%)<br>4 less than half of the time (25%)<br>5 never (0%)
176	[prevent11_e]<br><br>Show the field ONLY if:<br>[language] = '1'	Cancelled or postponed planned travel for work	radio (Matrix)<br>1 always (100%)<br>2 most of the time (75%)<br>3 half of the time (50%)<br>4 less than half of the time (25%)<br>5 never (0%)
177	[prevent12_e]<br><br>Show the field ONLY if:<br>[language] = '1'	Cancelled or postponed travel for pleasure	radio (Matrix)<br>1 always (100%)<br>2 most of the time (75%)<br>3 half of the time (50%)<br>4 less than half of the time (25%)<br>5 never (0%)
178	[prevent13_e]<br><br>Show the field ONLY if:<br>[language] = '1'	Cancelled or postponed personal or social activities	radio (Matrix)<br>1 always (100%)<br>2 most of the time (75%)<br>3 half of the time (50%)<br>4 less than half of the time (25%)<br>5 never (0%)
179	[prevent14_e]<br><br>Show the field ONLY if:<br>[language] = '1'	Cancelled a doctor's appointment	radio (Matrix)<br>1 always (100%)<br>2 most of the time (75%)<br>3 half of the time (50%)<br>4 less than half of the time (25%)<br>5 never (0%)

180	[prevent15_e]<br><br>Show the field ONLY if:<br>[language] = '1'	Stockpiled food or water	radio (Matrix)<br>1 always (100%)<br>2 most of the time (75%)<br>3 half of the time (50%)<br>4 less than half of the time (25%)<br>5 never (0%)
181	[prevent16_e]<br><br>Show the field ONLY if:<br>[language] = '1'	Followed government guidelines or rules to shelter in place (staying at home, limiting contacts with other people, etc.)	radio (Matrix)<br>1 always (100%)<br>2 most of the time (75%)<br>3 half of the time (50%)<br>4 less than half of the time (25%)<br>5 never (0%)
182	[receivedcovidvaccine_e]<br><br>Show the field ONLY if:<br>[language] = '1'	Have you received a COVID vaccine outside of a clinical trial?	yesno<br>1 Yes<br>0 No
183	[receivedvaccine_where_e]<br><br>Show the field ONLY if:<br>[language] = '1' and [receivedcovidvaccine_e] = '1'	Where did you receive the COVID vaccine?	radio<br>1 Doctors Office<br>2 Work/Employment<br>3 Retail (e.g. Walgreens, CVS)<br>5 Vaccine site<br>4 Other
184	[receivedvaccine_where_oth_e]<br><br>Show the field ONLY if:<br>[language] = '1' and [receivedcovidvaccine_e] = '1' and [receivedvaccine_where_e] = '4'	Please specify where you received the COVID vaccine.	text
185	[receivedvaccine_site_e]<br><br>Show the field ONLY if:<br>[language] = '1' and [receivedcovidvaccine_e] = '1' and [receivedvaccine_where_e] = '5'	Please specify the city/town in NC of the vaccine site:	text
186	[receievedvaccine_site2_e]<br><br>Show the field ONLY if:<br>[language] = '1' and [receivedcovidvaccine_e] = '1' and [receivedvaccine_where_e] = '5'	Please specify who was the hosting organization/institution of the vaccine site:	text
187	[receivedvaccine_name_e]<br><br>Show the field ONLY if:<br>[language] = '1' and [receivedcovidvaccine_e] = '1'	Which COVID vaccine did you receive?	radio<br>1 Pfizer<br>2 Moderna<br>3 AstraZeneca

			4 Novavax<br>6 Johnson & Johnson<br>5 Other<br>9 Unsure/Unknown
188	[receivedvaccine_name_oth_e]<br><br>Show the field ONLY if: [language] = '1' and [receivedcovidvaccine_e] = '1' and [receivedvaccine_name_e] = '5'	Please specify which other COVID vaccine you received.	text
189	[receivedvaccine_dose_e]<br><br>Show the field ONLY if: [language] = '1' and [receivedcovidvaccine_e] = '1'	How many doses of the vaccine have you received?	radio<br>1 1<br>2 2
190	[receivedvaccine_first_dt_e]<br><br>Show the field ONLY if: [receivedvaccine_dose_e] = '1' OR [receivedvaccine_dose_e] = '2'	What day did you receive the first dose of the vaccine?	text (date_mdy), Required
191	[receivedvaccine_second_dt_e]<br><br>Show the field ONLY if: [language] = '1' and [receivedcovidvaccine_e] = '1' and [receivedvaccine_dose_e] = '2'	What day did you receive the second dose of the vaccine?	text (date_mdy), Required
192	[vacc_pain_e]<br><br>Show the field ONLY if: [language] = '1' and [receivedcovidvaccine_e] = '1'	Section Header: *Did you experience any of the following side effects after vaccination?*<br><br>pain at or around the injection site	radio (Matrix)<br>0 No<br>1 Mild (you notice symptoms, but they aren't a problem)<br>2 Moderate (symptoms limit your normal daily activities)<br>3 Severe (symptoms make normal daily activities difficult or impossible)<br><br>Question number: 1
193	[vacc_redness_e]<br><br>Show the field ONLY if: [language] = '1' and [receivedcovidvaccine_e] = '1'	redness at or around the injection site	radio (Matrix)<br>0 No<br>1 Mild (you notice symptoms, but they aren't a problem)<br>2 Moderate (symptoms limit your normal daily activities)<br>3 Severe (symptoms make normal daily activities difficult or impossible)<br><br>Question number: 2
194	[vacc_swell_e]<br><br>Show the field ONLY if:	swelling at or around the injection site	radio (Matrix)<br>0 No

	[language] = '1' and [receivedcovidvaccine_e] = '1'		1 Mild (you notice symptoms, but they aren't a problem)<br>2 Moderate (symptoms limit your normal daily activities)<br>3 Severe (symptoms make normal daily activities difficult or impossible)<br><br>Question number: 3
195	[vacc_rash_e]<br><br>Show the field ONLY if:<br>[language] = '1' and [receivedcovidvaccine_e] = '1'	rash at or around the injection site	radio (Matrix)<br>0 No<br>1 Mild (you notice symptoms, but they aren't a problem)<br>2 Moderate (symptoms limit your normal daily activities)<br>3 Severe (symptoms make normal daily activities difficult or impossible)<br><br>Question number: 4
196	[vacc_headache_e]<br><br>Show the field ONLY if:<br>[language] = '1' and [receivedcovidvaccine_e] = '1'	headache	radio (Matrix)<br>0 No<br>1 Mild (you notice symptoms, but they aren't a problem)<br>2 Moderate (symptoms limit your normal daily activities)<br>3 Severe (symptoms make normal daily activities difficult or impossible)<br><br>Question number: 5
197	[vacc_fatigue_e]<br><br>Show the field ONLY if:<br>[language] = '1' and [receivedcovidvaccine_e] = '1'	fatigue	radio (Matrix)<br>0 No<br>1 Mild (you notice symptoms, but they aren't a problem)<br>2 Moderate (symptoms limit your normal daily activities)<br>3 Severe (symptoms make normal daily activities difficult or impossible)<br><br>Question number: 6
198	[vacc_fever_e]<br><br>Show the field ONLY if:<br>[language] = '1' and [receivedcovidvaccine_e] = '1'	fever (temperature >100.4°F or >38°C)	radio (Matrix)<br>0 No<br>1 Mild (you notice symptoms, but they aren't a problem)<br>2 Moderate (symptoms limit your normal daily activities)<br>3 Severe (symptoms make normal daily activities difficult or impossible)<br><br>Question number: 7
199	[vacc_chills_e]<br><br>Show the field ONLY if:	chills	radio (Matrix)<br>0 No

	[language] = '1' and [receivedcovidvaccine_e] = '1'		1 Mild (you notice symptoms, but they aren't a problem)<br>2 Moderate (symptoms limit your normal daily activities)<br>3 Severe (symptoms make normal daily activities difficult or impossible)<br><br>Question number: 8
200	[vacc_joint_e]<br><br>Show the field ONLY if:<br>[language] = '1' and [receivedcovidvaccine_e] = '1'	joint pain	radio (Matrix)<br>0 No<br>1 Mild (you notice symptoms, but they aren't a problem)<br>2 Moderate (symptoms limit your normal daily activities)<br>3 Severe (symptoms make normal daily activities difficult or impossible)<br><br>Question number: 9
201	[vacc_muscle_e]	muscle pain	radio (Matrix)<br>0 No<br>1 Mild (you notice symptoms, but they aren't a problem)<br>2 Moderate (symptoms limit your normal daily activities)<br>3 Severe (symptoms make normal daily activities difficult or impossible)<br><br>Question number: 10
202	[vacc_nausea_e]<br><br>Show the field ONLY if:<br>[language] = '1' and [receivedcovidvaccine_e] = '1'	nausea	radio (Matrix)<br>0 No<br>1 Mild (you notice symptoms, but they aren't a problem)<br>2 Moderate (symptoms limit your normal daily activities)<br>3 Severe (symptoms make normal daily activities difficult or impossible)<br><br>Question number: 11
203	[vacc_effects_e]<br><br>Show the field ONLY if:<br>[vacc_pain_e]>0 OR [vacc_redness_e]>0 OR [vacc_swell_e]>0 OR [vacc_rash_e]>0 OR [vacc_headache_e]>0 OR [vacc_fatigue_e]>0 OR [vacc_fever_e]>0 OR [vacc_chills_e]>0 OR [vacc_joint_e]>0 OR [vacc_muscle_e]>0 OR [vacc_nausea_e]>0	How long did these side effects last?	radio<br>1 Less than 12 hours<br>2 12 to 24 hours<br>3 more than 24 hours
204	[vacc_med_e]<br><br>Show the field ONLY if:	Did you take any medication for these side effects?	radio<br>1 Yes

	[vacc_pain_e]>0 OR [vacc_redness_e]>0 OR [vacc_swell_e]>0 OR [vacc_rash_e]>0 OR [vacc_headache_e]>0 OR [vacc_fatigue_e]>0 OR [vacc_fever_e]>0 OR [vacc_chills_e]>0 OR [vacc_joint_e]>0 OR [vacc_muscle_e]>0 OR [vacc_nausea_e]>0		0 No
205	[vacc_med_list_e]<br><br>Show the field ONLY if:<br>[vacc_med_e] = '1'	What medication(s) did you take for the side effects?<br>*Please list all medications.*	text
206	[vacc_effects_consult_e]<br><br>Show the field ONLY if:<br>[vacc_pain_e]>0 OR [vacc_redness_e]>0 OR [vacc_swell_e]>0 OR [vacc_rash_e]>0 OR [vacc_headache_e]>0 OR [vacc_fatigue_e]>0 OR [vacc_fever_e]>0 OR [vacc_chills_e]>0 OR [vacc_joint_e]>0 OR [vacc_muscle_e]>0 OR [vacc_nausea_e]>0	Did you consult a physician or other health care provider for the side effects?	radio<br>1 Yes<br>0 No
207	[vacc_effects_dose_e]<br><br>Show the field ONLY if:<br>[vacc_pain_e]>0 OR [vacc_redness_e]>0 OR [vacc_swell_e]>0 OR [vacc_rash_e]>0 OR [vacc_headache_e]>0 OR [vacc_fatigue_e]>0 OR [vacc_fever_e]>0 OR [vacc_chills_e]>0 OR [vacc_joint_e]>0 OR [vacc_muscle_e]>0 OR [vacc_nausea_e]>0 OR [vacc_effects_e]>0 AND [receivedvaccine_dose_e(2)] = '1'	How did you experience the side effects after the second dose of the vaccination as compared to those after the first dose of the vaccination?	radio<br>3 More severe<br>2 Less severe<br>1 Equally severe<br>0 Not applicable/Haven't received second dose yet
208	[covidvaccine_e]<br><br>Show the field ONLY if:<br>[language] = '1' and [receivedcovidvaccine_e] = '0'	Do you plan to get a vaccine for COVID-19 if one becomes available?	radio<br>1 yes<br>0 no<br>2 don't know
209	[vaccine_hesitancy_e]<br><br>Show the field ONLY if:<br>[covidvaccine_e]= '0' or [covidvaccine_e]= '2'	Which of the following, if any, are reasons that you answered "no" or "don't know" about getting a COVID-19 vaccine?<br>*select all that apply*	checkbox<br>1 vaccine_hesitancy_e___1 I am concerned [illegible] the side effects [illegible] safety of the CO[illegible] vaccine<br>2 vaccine_hesitancy_e___2 I have an underl[illegible] condition and th[illegible] not enough rese[illegible] to make me feel comfortable gett[illegible] vaccine at this ti[illegible]

3	vaccine_hesitancy_e___3	I am concerned t the COVID-19 va is being develop fast	
4	vaccine_hesitancy_e___4	I plan to wait an it is safe and ma later	
5	vaccine_hesitancy_e___5	I do not trust the government	
6	vaccine_hesitancy_e___6	I plan to use mas and other precau instead	
7	vaccine_hesitancy_e___7	I am not a memb any group that is high risk from CO 19	
8	vaccine_hesitancy_e___8	I do not believe C 19 is a serious ill	
9	vaccine_hesitancy_e___9	I believe the vacc can give me COV	
10	vaccine_hesitancy_e___10	I do not think the COVID-19 vaccin work	
11	vaccine_hesitancy_e___11	I do not like nee	
12	vaccine_hesitancy_e___12	I already had CO and believe that should be immu	
13	vaccine_hesitancy_e___13	My doctor has n recommended a COVID-19 vaccin me	
14	vaccine_hesitancy_e___14	I did not know I needed a vaccin against COVID-1	
15	vaccine_hesitancy_e___15	I am concerned a the costs associa with the vaccine as office visit cos vaccine administ fees)	
16	vaccine_hesitancy_e___16	I am not yet eligi (under NC phase guidelines or du other health conditions/proce	
17	vaccine_hesitancy_e___17	I do not have acc	
18	vaccine_hesitancy_e___18	Something else	

210	[vaccine_hesitancy_oth_e]<br>Show the field ONLY if: [vaccine_hesitancy_e(18)] = '1'	Please specify what other reason you may or may not be receiving a COVID-19 vaccine:	text
211	[fever_e]	Section Header: *During the last two weeks, have you experienced any of the following symptoms?*	radio (Matrix)

	Show the field ONLY if: [language] = '1'	Fever (measured by thermometer or self-diagnosed)	1 yes<br>0 no
212	[cough_e]<br><br>Show the field ONLY if: [language] = '1'	Cough (new or worsening)	radio (Matrix)<br>1 yes<br>0 no
213	[sob_e]<br><br>Show the field ONLY if: [language] = '1'	Shortness of breath (new or worsening)	radio (Matrix)<br>1 yes<br>0 no
214	[fatigue_e]<br><br>Show the field ONLY if: [language] = '1'	Fatigue (new tiredness doing normal activities)	radio (Matrix)<br>1 yes<br>0 no
215	[bodyache_e]<br><br>Show the field ONLY if: [language] = '1'	Body aches	radio (Matrix)<br>1 yes<br>0 no
216	[headache_e]<br><br>Show the field ONLY if: [language] = '1'	Headache	radio (Matrix)<br>1 yes<br>0 no
217	[diarrhea_e]<br><br>Show the field ONLY if: [language] = '1'	Diarrhea	radio (Matrix)<br>1 yes<br>0 no
218	[pharyngitis_e]<br><br>Show the field ONLY if: [language] = '1'	Sore throat	radio (Matrix)<br>1 yes<br>0 no
219	[eye_e]<br><br>Show the field ONLY if: [language] = '1'	Itchy, pink, or painful eyes	radio (Matrix)<br>1 yes<br>0 no
220	[congest_e]<br><br>Show the field ONLY if: [language] = '1'	Runny nose or congestion	radio (Matrix)<br>1 yes<br>0 no
221	[sensory_e]<br><br>Show the field ONLY if: [language] = '1'	Changes in your sense of smell or taste	radio (Matrix)<br>1 yes<br>0 no
222	[rash_e]<br><br>Show the field ONLY if: [language] = '1'	New rash	radio (Matrix)<br>1 yes<br>0 no
223	[chills_e]<br><br>Show the field ONLY if: [language] = '1'	Repeated shaking with chills	radio (Matrix)<br>1 yes<br>0 no
224	[covid_symp_e]<br><br>Show the field ONLY if: [language] = '1' and ([fever_e] = '1' or [cough_e] =	When did the symptoms reported above first start?	text (date_mdy)

	'1' or [sob_e] = '1' or [fatigue_e] = '1' or [bodyache_e] = '1' or [headache_e] = '1' or [diarrhea_e] = '1' or [pharyngitis_e] = '1' or [eye_e] = '1' or [congest_e] = '1' or [sensory_e] = '1' or [rash_e] = '1' or [chills_e] = '1')		
225	[covid_symp2_e]<br><br>Show the field ONLY if: [language] = '1' and ([fever_e] = '1' or [cough_e] = '1' or [sob_e] = '1' or [fatigue_e] = '1' or [bodyache_e] = '1' or [headache_e] = '1' or [diarrhea_e] = '1' or [pharyngitis_e] = '1' or [eye_e] = '1' or [congest_e] = '1' or [sensory_e] = '1' or [rash_e] = '1' or [chills_e] = '1')	Were you worried that you may have COVID-19 due to the symptoms you reported?	radio<br>1 yes<br>0 no<br>2 don't know
226	[covid_symp3_e]<br><br>Show the field ONLY if: [language] = '1' and ([fever_e] = '1' or [cough_e] = '1' or [sob_e] = '1' or [fatigue_e] = '1' or [bodyache_e] = '1' or [headache_e] = '1' or [diarrhea_e] = '1' or [pharyngitis_e] = '1' or [eye_e] = '1' or [congest_e] = '1' or [sensory_e] = '1' or [rash_e] = '1' or [chills_e] = '1')	Did you experience any bias or discrimination due to the symptoms you reported?	radio<br>1 yes<br>0 no<br>2 don't know
227	[prevent17_e]<br><br>Show the field ONLY if: [language] = '1' and ([fever_e] = '1' or [cough_e] = '1' or [sob_e] = '1' or [fatigue_e] = '1' or [bodyache_e] = '1' or [headache_e] = '1' or [diarrhea_e] = '1' or [pharyngitis_e] = '1' or [eye_e] = '1' or [congest_e] = '1' or [sensory_e] = '1' or [rash_e] = '1' or [chills_e] = '1')	Which of the following did you do to protect your friends and family after your symptoms began?	checkbox<br>1 prevent17_e___1 wore a mask more frequently<br>2 prevent17_e___2 washed your hands with soap and water more frequently<br>3 prevent17_e___3 used hand sanitizer more frequently<br>4 prevent17_e___4 isolated yourself in your home more frequently<br>5 prevent17_e___5 stayed home more frequently<br>6 prevent17_e___6 wore disposable gloves more frequently
228	[covid_symp4_e]<br><br>Show the field ONLY if: [language] = '1' and ([fever_e] = '1' or [cough_e] = '1' or [sob_e] = '1' or [fatigue_e] = '1' or [bodyache_	What did you do in response to the symptoms reported above?<br>*Select all that apply.*	checkbox<br>0 covid_symp4_e___0 nothing<br>1 covid_symp4_e___1 took over the counter medication (ibuprofen,

	e] = '1' or [headache_e] = '1' or [diarrhea_e] = '1' or [pharyngitis_e] = '1' or [eye_e] = '1' or [congest_e] = '1' or [sensory_e] = '1' or [rash_e] = '1' or [chills_e] = '1')		acetaminophen, etc.)<br>2 covid_symp4_e___2 communicated with a health care provider over the phone<br>3 covid_symp4_e___3 visited a health care provider's office<br>4 covid_symp4_e___4 visited a retail clinic or pharmacy<br>5 covid_symp4_e___5 visited urgent care (FASTMed, etc.)<br>6 covid_symp4_e___6 visited the emergency room<br>7 covid_symp4_e___7 was admitted to the hospital<br>8 covid_symp4_e___8 other
229	[covid_symp5_e]<br>Show the field ONLY if: [language] = '1' and [covid_symp4_e(8)] = '1'	Please specify what other action you took in response to your symptoms.	text
230	[covid_symp6_e]<br>Show the field ONLY if: [language] = '1' and ([covid_symp4_e(2)] = '1' or [covid_symp4_e(3)] = '1' or [covid_symp4_e(4)] = '1' or [covid_symp4_e(5)] = '1' or [covid_symp4_e(6)] = '1' or [covid_symp4_e(7)] = '1' or [covid_symp4_e(8)] = '1')	If you were able to talk with a health care provider, were you told that you may have COVID-19?	radio<br>1 yes<br>0 no<br>2 don't know
231	[covid_test_e]<br>Show the field ONLY if: [language] = '1' and ([fever_e] = '1' or [cough_e] = '1' or [sob_e] = '1' or [fatigue_e] = '1' or [bodyache_e] = '1' or [headache_e] = '1' or [diarrhea_e] = '1' or [pharyngitis_e] = '1' or [eye_e] = '1' or [congest_e] = '1' or [sensory_e] = '1' or [rash_e] = '1' or [chills_e] = '1')	If you received a COVID-19 test due to the symptoms you reported, what was the result?	radio<br>1 pending<br>2 positive<br>3 negative<br>4 inconclusive<br>5 did not receive a test
232	[covid_admit_e]<br>Show the field ONLY if: [language] = '1' and [covid_symp4_e(7)] = '1'	How many days were you admitted to the hospital?	text (number, Min: 0)
233	[covid_admit2_e]<br>Show the field ONLY if: [language] = '1' and [covid_symp4_e(7)] = '1'	Did you receive the following interventions during your hospital admission?	checkbox<br>1 covid_admit2_e___1 extra oxygen in your nose<br>2 covid_admit2_e___2 treatment in the intensive care unit

			(ICU)<br>3 | covid_admit2_e___3 | mechanical ventilation (intubation or a breathing tube)
234	[covid_symp7_e]<br>Show the field ONLY if: [language] = '1' and ([fever_e] = '1' or [cough_e] = '1' or [sob_e] = '1' or [fatigue_e] = '1' or [bodyache_e] = '1' or [headache_e] = '1' or [diarrhea_e] = '1' or [pharyngitis_e] = '1' or [eye_e] = '1' or [congest_e] = '1' or [sensory_e] = '1' or [rash_e] = '1' or [chills_e] = '1')	Have you returned to your normal health at this time?	radio<br>1 | yes<br>0 | no<br>2 | don't know
235	[address_e]<br>Show the field ONLY if: [language] = '1'	Section Header: *Please provide the following information about your household.*<br>What is your permanent address?	text
236	[address2_e]<br>Show the field ONLY if: [language] = '1'	How long have you lived at this address?	radio<br>1 | 0-3 years<br>2 | 4-6 years<br>3 | 7-10 years<br>4 | more than 10 years
237	[hhcount_e]<br>Show the field ONLY if: [language] = '1'	How many additional people (not including yourself) live or spend a significant amount of time (greater than 40 hours a week) in this household?	radio<br>0 | 0<br>1 | 1<br>2 | 2<br>3 | 3<br>4 | 4<br>5 | 5<br>6 | 6<br>7 | 7<br>8 | 8<br>9 | 9<br>10 | 10<br>11 | 11<br>12 | 12
238	[hhcount2_e]<br>Show the field ONLY if: [language] = '1' and [hhcount_e] > 0	How many of the people in your household are below the age of 18?	radio<br>0 | 0<br>1 | 1<br>2 | 2<br>3 | 3<br>4 | 4<br>5 | 5

			6 6<br>7 7<br>8 8<br>9 9<br>10 10<br>11 11<br>12 12
239	[hhcount3_e]<br><br>Show the field ONLY if:<br>[language] = '1' and [hhcount2_e] > 0	How difficult has it been for your household to adjust to changes in child care or having to home school?	radio<br>1 not difficult<br>2 somewhat difficult<br>3 very difficult<br>4 extremely difficult
240	[hhlang_e]<br><br>Show the field ONLY if:<br>[language] = '1'	What is the primary language spoken in your household?	radio<br>1 English<br>2 Spanish<br>3 Other
241	[hhlang2_e]<br><br>Show the field ONLY if:<br>[language] = '1' and [hhlang_e] = '3'	What other language is the primary language of your household?	text
242	[caregive_e]<br><br>Show the field ONLY if:<br>[language] = '1'	Before the COVID-19 pandemic, did you have regular caregiving responsibilities for someone who didn't live in your household (ex. elderly parent or sibling who you regularly visited and supported, etc.)?	yesno<br>1 Yes<br>0 No
243	[caregive2_e]<br><br>Show the field ONLY if:<br>[language] = '1' and [caregive_e] = '1'	How difficult has it been to continue your caregiving responsibilities due to the COVID-19 pandemic?	radio<br>1 not difficult<br>2 somewhat difficult<br>3 very difficult<br>4 extremely difficult
244	[isolate_e]<br><br>Show the field ONLY if:<br>[language] = '1' and [hhcount_e] > 0	If someone in the household became sick with COVID-19, how well would the household be able to isolate them from other household members (let them stay in their own room and limit contact with them)?	radio<br>1 not very well<br>2 pretty well<br>3 very well<br>4 extremely well
245	[income_e]<br><br>Show the field ONLY if:<br>[language] = '1'	What was your approximate total household income last year from all sources, before taxes?	radio<br>1 less than $10,000<br>2 $10,000-$19,999<br>3 $20,000-$29,999<br>4 $30,000-$49,999<br>5 $50,000-$74,999<br>6 $75,000 or more
246	[income2_e]<br><br>Show the field ONLY if:	How do you think your total household income will change this year due to the COVID-19 crisis?	radio<br>1 decrease significantly

	[language] = '1'		2 decrease slightly<br>3 stay the same<br>4 increase slightly<br>5 increase significantly
247	[runout_e]<br><br>Show the field ONLY if:<br>[language] = '1'	On a scale of 0 (definitely not going to happen) to 10 (definitely going to happen), how likely do you think it is that your household will run out of money in the next 3 months?	text (number, Min: 0, Max: 10)
248	[errands_e]<br><br>Show the field ONLY if:<br>[language] = '1'	How often are you or your household getting help with running necessary errands, such as getting groceries or medications?	radio<br>1 always (100%)<br>2 most of the time (75%)<br>3 half of the time (50%)<br>4 less than half of the time (25%)<br>5 never (0%)
249	[isolate2_e]<br><br>Show the field ONLY if:<br>[language] = '1'	How often have you or your household been staying at home and avoiding interacting with others, aside from getting groceries?	radio<br>1 always (100%)<br>2 most of the time (75%)<br>3 half of the time (50%)<br>4 less than half of the time (25%)<br>5 never (0%)
250	[hh1_relationship_e]<br><br>Show the field ONLY if:<br>[language] = '1' and [hhcount_e] > 0 and [hhcount_e] < 13	Section Header: *For each additional person in your household, please provide the following information.*<br><br>Person 1: What is your relationship to this person?	radio<br>1 partner or spouse<br>2 child<br>3 parent<br>4 sibling<br>5 other family member<br>6 in-home childcare provider or other caregiver<br>7 other
251	[hh1_relationship2_e]<br><br>Show the field ONLY if:<br>[language] = '1' and [hh1_relationship_e] = '7'	Person 1: Please specify your relationship with this person.	text
252	[hh1_age_e]<br><br>Show the field ONLY if:<br>[language] = '1' and [hhcount_e] > 0 and [hhcount_e] < 13	Person 1: What is this person's age?<br>*Please specify their age in years*	text (number, Min: 0, Max: 110)
253	[hh1_sex_e]<br><br>Show the field ONLY if:<br>[language] = '1' and [hhcount_e] > 0 and [hhcount_e] < 13	Person 1: What is this person's sex?	radio<br>1 Female<br>2 Male<br>3 Other
254	[hh1_race_e]<br><br>Show the field ONLY if:	Person 1: What is this person's race?<br>*Select all that apply.*	checkbox<br>1 hh1_race_e___1 American Indian or Alaska Native

	[language] = '1' and [hhcount_e] > 0 and [hhcount_e] < 13		2 hh1_race_e___2 Asian<br>3 hh1_race_e___3 Black or African American<br>4 hh1_race_e___4 Native Hawaiian or Pacific Islander<br>5 hh1_race_e___5 White<br>6 hh1_race_e___6 Other<br>7 hh1_race_e___7 don't know
255	[hh1_ethn_e]<br>Show the field ONLY if: [language] = '1' and [hhcount_e] > 0 and [hhcount_e] < 13	Person 1: What is this person's ethnicity?	radio<br>1 Hispanic or Latino<br>2 Not Hispanic or Latino<br>3 Other<br>4 don't know
256	[hh1_edu_e]<br>Show the field ONLY if: [language] = '1' and [hhcount_e] > 0 and [hhcount_e] < 13	Person 1: What is the highest level of education or schooling this person has completed?	radio<br>1 never attended school<br>2 kindergarten - 8th grade<br>3 some high school<br>4 high school equivalency (GED)<br>5 high school graduate<br>6 some college<br>7 college graduate<br>8 graduate school or more<br>9 don't know
257	[hh1_work_e]<br>Show the field ONLY if: [language] = '1' and [hhcount_e] > 0 and [hhcount_e] < 13	Person 1: Which of the following best fit this person's current work situation?	radio<br>1 works full time<br>2 works part time<br>3 is looking for work/employment<br>4 retired<br>5 homemaker<br>6 student<br>7 on maternity/paternity leave<br>8 on illness/sick leave<br>9 on disability<br>10 other<br>11 don't know
258	[hh1_work2_e]<br>Show the field ONLY if: [language] = '1' and [hhcount_e] > 0 and [hhcount_e] < 13	Person 1: Does this person currently consider themselves self-employed (including as an independent contractor or gig-economy worker)?	radio<br>1 yes<br>0 no<br>2 don't know
259	[hh1_work3_e]<br>Show the field ONLY if: [language] = '1' and [hhcount_e] > 0 and [hhcount_	Person 1: Does this person currently work in any of the following high-risk settings for COVID-19 transmission?	checkbox<br>1 hh1_work3_e___1 healthcare setting (hospital, clinic, urgent care, etc.)

	e] < 13 and ([hh1_work_e] = '1' or [hh1_work_e] = '2' or [hh1_work2_e] = '1')		2 | hh1_work3_e___2 | dense residential setting (nursing home, other long-term care facility)<br>3 | hh1_work3_e___3 | prison or jail<br>4 | hh1_work3_e___4 | meatpacking facility<br>5 | hh1_work3_e___5 | shipping or distribution facility<br>6 | hh1_work3_e___6 | high-volume retail facility (grocery store, etc.)<br>7 | hh1_work3_e___7 | don't know
260	[hh1_work4_e]<br><br>Show the field ONLY if:<br>[language] = '1' and [hhcount_e] > 0 and [hhcount_e] < 13 and ([hh1_work_e] = '1' or [hh1_work_e] = '2' or [hh1_work2_e] = '1')	Person 1: Does this person's employer offer them any of the following benefits at their current main job?<br>*Select all that apply.*	checkbox<br>1 | hh1_work4_e___1 | paid sick leave<br>2 | hh1_work4_e___2 | paid vacation/personal leave<br>3 | hh1_work4_e___3 | health insurance<br>4 | hh1_work4_e___4 | disability insurance<br>5 | hh1_work4_e___5 | retirement plan<br>6 | hh1_work4_e___6 | other<br>7 | hh1_work4_e___7 | don't know
261	[hh1_work5_e]<br><br>Show the field ONLY if:<br>[language] = '1' and [hhcount_e] > 0 and [hhcount_e] < 13 and ([hh1_work_e] = '1' or [hh1_work_e] = '2' or [hh1_work2_e] = '1')	Person 1: On a scale of 0 (definitely not going to happen) to 10 (definitely going to happen), how likely is it that this person will lose their job because of the COVID-19 pandemic?	text (number, Min: 0, Max: 10)
262	[hh1_work6_e]<br><br>Show the field ONLY if:<br>[language] = '1' and [hhcount_e] > 0 and [hhcount_e] < 13 and ([hh1_work_e] = '1' or [hh1_work_e] = '2' or [hh1_work2_e] = '1')	Person 1: On a scale of 0 (definitely not going to happen) to 10 (definitely going to happen), how likely is it that this person will receive fewer work hours at their job because of the COVID-19 pandemic?	text (number, Min: 0, Max: 10)
263	[hh1_work7_e]<br><br>Show the field ONLY if:<br>[language] = '1' and [hhcount_e] > 0 and [hhcount_e] < 13 and ([hh1_work_e] = '1' or [hh1_work_e] = '2' or [hh1_work2_e] = '1')	Person 1: How often is this person required to work from outside of the home currently?	radio (Matrix)<br>1 | always (100%)<br>2 | most of the time (75%)<br>3 | half of the time (50%)<br>4 | less than half of the time (25%)<br>5 | never (0%)<br>6 | don't know
264	[hh1_work8_e]<br><br>Show the field ONLY if:<br>[language] = '1' and ([hh1_work7_e] = '1' or [hh1_work7_e] = '2' or [hh1_wor	Person 1: How regularly is this person in close physical contact with co-workers during their work outside of the home currently?	radio (Matrix)<br>1 | always (100%)<br>2 | most of the time (75%)<br>3 | half of the time (50%)<br>4 | less than half of the time (25%)

	k7_e] = '3' or [hh1_work7_e] = '4')		5 never (0%)<br>6 don't know
265	[hh1_work9_e]<br><br>Show the field ONLY if: [language] = '1' and ([hh1_work7_e] = '1' or [hh1_work7_e] = '2' or [hh1_work7_e] = '3' or [hh1_work7_e] = '4')	Person 1: How regularly is this person in close physical contact with clients during their work outside of the home currently?	radio (Matrix)<br>1 always (100%)<br>2 most of the time (75%)<br>3 half of the time (50%)<br>4 less than half of the time (25%)<br>5 never (0%)<br>6 don't know
266	[hh1_covidvaccine_e]<br><br>Show the field ONLY if: [language] = '1' and [hhcount_e] > 0 and [hhcount_e] < 13	Person 1: Does this person plan to get a vaccine for COVID-19 when one becomes available?	radio<br>1 yes<br>0 no<br>2 don't know
267	[hh1_covidsymp_e]<br><br>Show the field ONLY if: [language] = '1' and [hhcount_e] > 0 and [hhcount_e] < 13	Person 1: Has this person had any symptoms (cough, fever, difficulty breathing, fatigue, body aches, diarrhea, runny nose, loss of smell or taste) consistent with COVID-19 in the last two weeks?	radio<br>1 yes<br>0 no<br>2 don't know
268	[hh1_covidsymp2_e]<br><br>Show the field ONLY if: [language] = '1' and [hh1_covidsymp_e] = '1'	Person 1: When did this person's symptoms begin?	text (date_mdy)
269	[hh1_covidsymp3_e]<br><br>Show the field ONLY if: [language] = '1' and [hh1_covidsymp_e] = '1'	Person 1: Is this person worried that they may have had COVID-19 because of their symptoms?	radio<br>1 yes<br>0 no<br>2 don't know
270	[hh1_covidsymp4_e]<br><br>Show the field ONLY if: [language] = '1' and [hh1_covidsymp_e] = '1'	Person 1: Did this person experience any bias or discrimination because of their symptoms?	radio<br>1 yes<br>0 no<br>2 don't know
271	[hh1_covidsymp5_e]<br><br>Show the field ONLY if: [language] = '1' and [hh1_covidsymp_e] = '1'	Person 1: What did this person do in response to their symptoms?<br>*Select all that apply.*	checkbox<br>0 hh1_covidsymp5_e___0 nothing<br>1 hh1_covidsymp5_e___1 took over the counter medication (ibuprofen, acetaminophen, etc.)<br>2 hh1_covidsymp5_e___2 communicated with a health care provider over the phone<br>3 hh1_covidsymp5_e___3 visited a health care provider's office<br>4 hh1_covidsymp5_e___4 visited a retail clinic or

			pharmacy<br>5 hh1_covidsymp5_e___5 visited urgent care (FASTMed, etc.)<br>6 hh1_covidsymp5_e___6 visited the emergency room<br>7 hh1_covidsymp5_e___7 was admitted to the hospital<br>8 hh1_covidsymp5_e___8 other<br>9 hh1_covidsymp5_e___9 don't know
272	[hh1_covidsymp6_e]<br>Show the field ONLY if:<br>[language] = '1' and [hh1_covidsymp5_e(8)] = '1'	Person 1: Please specify what other action this person took in response to their symptoms.	text
273	[hh1_covidsymp7_e]<br>Show the field ONLY if:<br>[language] = '1' and ([hh1_covidsymp5_e(2)] = '1' or [hh1_covidsymp5_e(3)] = '1' or [hh1_covidsymp5_e(4)] = '1' or [hh1_covidsymp5_e(5)] = '1' or [hh1_covidsymp5_e(6)] = '1' or [hh1_covidsymp5_e(7)] = '1' or [hh1_covidsymp5_e(8)] = '1')	Person 1: Did a health care provider tell this person that they may have COVID-19?	radio<br>1 yes<br>0 no<br>2 don't know
274	[hh1_covid_test_e]<br>Show the field ONLY if:<br>[language] = '1' and [hh1_covidsymp_e] = '1'	Person 1: If this person received a COVID-19 test due to their symptoms, what was the result?	radio<br>1 pending<br>2 positive<br>3 negative<br>4 inconclusive<br>5 did not receive a test<br>6 don't know
275	[hh1_covid_admit_e]<br>Show the field ONLY if:<br>[language] = '1' and [hh1_covidsymp5_e(7)] = '1'	Person 1: How many days was this person admitted to the hospital?	text (number, Min: 0)
276	[hh1_covid_admit2_e]<br>Show the field ONLY if:<br>[language] = '1' and [hh1_covidsymp5_e(7)] = '1'	Person 1: Did this person receive any of the following interventions during their hospital admission?	checkbox<br>1 hh1_covid_admit2_e___1 extra oxygen in your nose<br>2 hh1_covid_admit2_e___2 treatment in the intensive care unit (ICU)<br>3 hh1_covid_admit2_e___3 mechanical ventilation (intubation or a breathing tube)<br>4 hh1_covid_admit2_e___4 don't know

277	[hh1_covidsymp8_e]<br><br>Show the field ONLY if:<br>[language] = '1' and [hh1_covidsymp_e] = '1'	Person 1: Has this person returned to their normal health at this time?	radio<br>1 yes<br>0 no<br>2 don't know
278	[hh1_prevent_e]<br><br>Show the field ONLY if:<br>[language] = '1' and [hh1_covidsymp_e] = '1'	Person 1: Which of the following did this person do to protect their friends and family after their symptoms began?	checkbox<br>1 hh1_prevent_e___1 wore a mask more frequently<br>2 hh1_prevent_e___2 washed your hands with soap and water more frequently<br>3 hh1_prevent_e___3 used hand sanitizer more frequently<br>4 hh1_prevent_e___4 isolated yourself in your home more frequently<br>5 hh1_prevent_e___5 stayed home more frequently<br>6 hh1_prevent_e___6 wore disposable gloves more frequently<br>7 hh1_prevent_e___7 don't know
279	[hh2_relationship_e]<br><br>Show the field ONLY if:<br>[language] = '1' and [hhcount_e] > 1 and [hhcount_e] < 13	Section Header: *For each additional person in the your household, please provide the following information.*<br><br>Person 2: What is your relationship to this person?	radio<br>1 partner or spouse<br>2 child<br>3 parent<br>4 sibling<br>5 other family member<br>6 in-home childcare provider or other caregiver<br>7 other
280	[hh2_relationship2_e]<br><br>Show the field ONLY if:<br>[language] = '1' and [hh2_relationship_e] = '7'	Person 2: Please specify your relationship with this person.	text
281	[hh2_age_e]<br><br>Show the field ONLY if:<br>[language] = '1' and [hhcount_e] > 1 and [hhcount_e] < 13	Person 2: What is this person's age?<br>*Please specify their age in years*	text (number, Min: 0, Max: 110)
282	[hh2_sex_e]<br><br>Show the field ONLY if:<br>[language] = '1' and [hhcount_e] > 1 and [hhcount_e] < 13	Person 2: What is this person's sex?	radio<br>1 Female<br>2 Male<br>3 Other
283	[hh2_race_e]<br><br>Show the field ONLY if:<br>[language] = '1' and [hhcount_e] > 1 and [hhcount_e] < 13	Person 2: What is this person's race?<br>*Select all that apply.*	checkbox<br>1 hh2_race_e___1 American Indian or Alaska Native<br>2 hh2_race_e___2 Asian

			3 hh2_race_e___3 Black or African American<br>4 hh2_race_e___4 Native Hawaiian or Pacific Islander<br>5 hh2_race_e___5 White<br>6 hh2_race_e___6 Other<br>7 hh2_race_e___7 don't know
284	[hh2_ethn_e]<br><br>Show the field ONLY if:<br>[language] = '1' and [hhcount_e] > 1 and [hhcount_e] < 13	Person 2: What is this person's ethnicity?	radio<br>1 Hispanic or Latino<br>2 Not Hispanic or Latino<br>3 Other<br>4 don't know
285	[hh2_edu_e]<br><br>Show the field ONLY if:<br>[language] = '1' and [hhcount_e] > 1 and [hhcount_e] < 13	Person 2: What is the highest level of education or schooling this person has completed?	radio<br>1 never attended school<br>2 kindergarten - 8th grade<br>3 some high school<br>4 high school equivalency (GED)<br>5 high school graduate<br>6 some college<br>7 college graduate<br>8 graduate school or more<br>9 don't know
286	[hh2_work_e]<br><br>Show the field ONLY if:<br>[language] = '1' and [hhcount_e] > 1 and [hhcount_e] < 13	Person 2: Which of the following best fit this person's current work situation?	radio<br>1 works full time<br>2 works part time<br>3 is looking for work/employment<br>4 retired<br>5 homemaker<br>6 student<br>7 on maternity/paternity leave<br>8 on illness/sick leave<br>9 on disability<br>10 other<br>11 don't know
287	[hh2_work2_e]<br><br>Show the field ONLY if:<br>[language] = '1' and [hhcount_e] > 1 and [hhcount_e] < 13	Person 2: Does this person currently consider themselves self-employed (including as an independent contractor or gig-economy worker)?	radio<br>1 yes<br>0 no<br>2 don't know
288	[hh2_work3_e]<br><br>Show the field ONLY if:<br>[language] = '1' and [hhcount_e] > 1 and [hhcount_e] < 13 and ([hh2_work_e]	Person 2: Does this person currently work in any of the following high-risk settings for COVID-19 transmission?	checkbox<br>1 hh2_work3_e___1 healthcare setting (hospital, clinic, urgent care, etc.)

	= '1' or [hh2_work_e] = '2' or [hh2_work2_e] = '1')		2 hh2_work3_e___2 dense residential setting (nursing home, other long-term care facility)<br>3 hh2_work3_e___3 prison or jail<br>4 hh2_work3_e___4 meatpacking facility<br>5 hh2_work3_e___5 shipping or distribution facility<br>6 hh2_work3_e___6 high-volume retail facility (grocery store, etc.)<br>7 hh2_work3_e___7 don't know
289	[hh2_work4_e]<br>Show the field ONLY if: [language] = '1' and [hhcount_e] > 1 and [hhcount_e] < 13 and ([hh2_work_e] = '1' or [hh2_work_e] = '2' or [hh2_work2_e] = '1')	Person 2: Does this person's employer offer them any of the following benefits at their current main job?<br>*Select all that apply.*	checkbox<br>1 hh2_work4_e___1 paid sick leave<br>2 hh2_work4_e___2 paid vacation/personal leave<br>3 hh2_work4_e___3 health insurance<br>4 hh2_work4_e___4 disability insurance<br>5 hh2_work4_e___5 retirement plan<br>6 hh2_work4_e___6 other<br>7 hh2_work4_e___7 don't know
290	[hh2_work5_e]<br>Show the field ONLY if: [language] = '1' and [hhcount_e] > 1 and [hhcount_e] < 13 and ([hh2_work_e] = '1' or [hh2_work_e] = '2' or [hh2_work2_e] = '1')	Person 2: On a scale of 0 (definitely not going to happen) to 10 (definitely going to happen), how likely is it that this person will lose their job because of the COVID-19 pandemic?	text (number, Min: 0, Max: 10)
291	[hh2_work6_e]<br>Show the field ONLY if: [language] = '1' and [hhcount_e] > 1 and [hhcount_e] < 13 and ([hh2_work_e] = '1' or [hh2_work_e] = '2' or [hh2_work2_e] = '1')	Person 2: On a scale of 0 (definitely not going to happen) to 10 (definitely going to happen), how likely is it that this person will receive fewer work hours at their job because of the COVID-19 pandemic?	text (number, Min: 0, Max: 10)
292	[hh2_work7_e]<br>Show the field ONLY if: [language] = '1' and [hhcount_e] > 1 and [hhcount_e] < 13 and ([hh2_work_e] = '1' or [hh2_work_e] = '2' or [hh2_work2_e] = '1')	Person 2: How often is this person required to work from outside of the home currently?	radio (Matrix)<br>1 always (100%)<br>2 most of the time (75%)<br>3 half of the time (50%)<br>4 less than half of the time (25%)<br>5 never (0%)<br>6 don't know
293	[hh2_work8_e]<br>Show the field ONLY if: [language] = '1' and ([hh2_work7_e] = '1' or [hh2_work7_e] = '2' or [hh2_wor	Person 2: How regularly is this person in close physical contact with co-workers during their work outside of the home currently?	radio (Matrix)<br>1 always (100%)<br>2 most of the time (75%)<br>3 half of the time (50%)<br>4 less than half of the time (25%)

	k7_e] = '3' or [hh2_work7_e] = '4')		5 never (0%)<br>6 don't know
294	[hh2_work9_e]<br><br>Show the field ONLY if:<br>[language] = '1' and ([hh2_work7_e] = '1' or [hh2_work7_e] = '2' or [hh2_work7_e] = '3' or [hh2_work7_e] = '4')	Person 2: How regularly is this person in close physical contact with clients during their work outside of the home currently?	radio (Matrix)<br>1 always (100%)<br>2 most of the time (75%)<br>3 half of the time (50%)<br>4 less than half of the time (25%)<br>5 never (0%)<br>6 don't know
295	[hh2_covidvaccine_e]<br><br>Show the field ONLY if:<br>[language] = '1' and [hhcount_e] > 1 and [hhcount_e] < 13	Person 2: Does this person plan to get a vaccine for COVID-19 when one becomes available?	radio<br>1 yes<br>0 no<br>2 don't know
296	[hh2_covidsymp_e]<br><br>Show the field ONLY if:<br>[language] = '1' and [hhcount_e] > 1 and [hhcount_e] < 13	Person 2: Has this person had any symptoms (cough, fever, difficulty breathing, fatigue, body aches, diarrhea, runny nose, loss of smell or taste) consistent with COVID-19 in the last two weeks?	radio<br>1 yes<br>0 no<br>2 don't know
297	[hh2_covidsymp2_e]<br><br>Show the field ONLY if:<br>[language] = '1' and [hh2_covidsymp_e] = '1'	Person 2: When did this person's symptoms begin?	text (date_mdy)
298	[hh2_covidsymp3_e]<br><br>Show the field ONLY if:<br>[language] = '1' and [hh2_covidsymp_e] = '1'	Person 2: Is this person worried that they may have had COVID-19 because of their symptoms?	radio<br>1 yes<br>0 no<br>2 don't know
299	[hh2_covidsymp4_e]<br><br>Show the field ONLY if:<br>[language] = '1' and [hh2_covidsymp_e] = '1'	Person 2: Did this person experience any bias or discrimination because of their symptoms?	radio<br>1 yes<br>0 no<br>2 don't know
300	[hh2_covidsymp5_e]<br><br>Show the field ONLY if:<br>[language] = '1' and [hh2_covidsymp_e] = '1'	Person 2: What did this person do in response to their symptoms?<br>*Select all that apply.*	checkbox<br>0 hh2_covidsymp5_e___0 nothing<br>1 hh2_covidsymp5_e___1 took over the counter medication (ibuprofen, acetaminophen, etc.)<br>2 hh2_covidsymp5_e___2 communicated with a health care provider over the phone<br>3 hh2_covidsymp5_e___3 visited a health care provider's office<br>4 hh2_covidsymp5_e___4 visited a retail clinic or

			pharmacy<br>5 | hh2_covidsymp5_e___5 | visited urgent care (FASTMed, etc.)<br>6 | hh2_covidsymp5_e___6 | visited the emergency room<br>7 | hh2_covidsymp5_e___7 | was admitted to the hospital<br>8 | hh2_covidsymp5_e___8 | other<br>9 | hh2_covidsymp5_e___9 | don't know
301	[hh2_covidsymp6_e]<br><br>Show the field ONLY if: [language] = '1' and [hh2_covidsymp5_e(8)] = '1'	Person 2: Please specify what other action this person took in response to their symptoms.	text
302	[hh2_covidsymp7_e]<br><br>Show the field ONLY if: [language] = '1' and ([hh2_covidsymp5_e(2)] = '1' or [hh2_covidsymp5_e(3)] = '1' or [hh2_covidsymp5_e(4)] = '1' or [hh2_covidsymp5_e(5)] = '1' or [hh2_covidsymp5_e(6)] = '1' or [hh2_covidsymp5_e(7)] = '1' or [hh2_covidsymp5_e(8)] = '1')	Person 2: Did a health care provider tell this person that they may have COVID-19?	radio<br>1 | yes<br>0 | no<br>2 | don't know
303	[hh2_covid_test_e]<br><br>Show the field ONLY if: [language] = '1' and [hh2_covidsymp_e] = '1'	Person 2: If this person received a COVID-19 test due to their symptoms, what was the result?	radio<br>1 | pending<br>2 | positive<br>3 | negative<br>4 | inconclusive<br>5 | did not receive a test<br>6 | don't know
304	[hh2_covid_admit_e]<br><br>Show the field ONLY if: [language] = '1' and [hh2_covidsymp5_e(7)] = '1'	Person 2: How many days was this person admitted to the hospital?	text (number, Min: 0)
305	[hh2_covid_admit2_e]<br><br>Show the field ONLY if: [language] = '1' and [hh2_covidsymp5_e(7)] = '1'	Person 2: Did this person receive any of the following interventions during their hospital admission?	checkbox<br>1 | hh2_covid_admit2_e___1 | extra oxygen in your nose<br>2 | hh2_covid_admit2_e___2 | treatment in the intensive care unit (ICU)<br>3 | hh2_covid_admit2_e___3 | mechanical ventilation (intubation or a breathing tube)<br>4 | hh2_covid_admit2_e___4 | don't know

306	[hh2_covidsymp8_e]<br><br>Show the field ONLY if:<br>[language] = '1' and [hh2_covidsymp_e] = '1'	Person 2: Has this person returned to their normal health at this time?	radio<br>1 yes<br>0 no<br>2 don't know
307	[hh2_prevent_e]<br><br>Show the field ONLY if:<br>[language] = '1' and [hh2_covidsymp_e] = '1'	Person 2: Which of the following did this person do to protect their friends and family after their symptoms began?	checkbox<br>1 hh2_prevent_e___1 wore a mask more frequently<br>2 hh2_prevent_e___2 washed your hands with soap and water more frequently<br>3 hh2_prevent_e___3 used hand sanitizer more frequently<br>4 hh2_prevent_e___4 isolated yourself in your home more frequently<br>5 hh2_prevent_e___5 stayed home more frequently<br>6 hh2_prevent_e___6 wore disposable gloves more frequently<br>7 hh2_prevent_e___7 don't know
308	[hh3_relationship_e]<br><br>Show the field ONLY if:<br>[language] = '1' and [hhcount_e] > 2 and [hhcount_e] < 13	Section Header: *For each additional person in the your household, please provide the following information.*<br><br>Person 3: What is your relationship to this person?	radio<br>1 partner or spouse<br>2 child<br>3 parent<br>4 sibling<br>5 other family member<br>6 in-home childcare provider or other caregiver<br>7 other
309	[hh3_relationship2_e]<br><br>Show the field ONLY if:<br>[language] = '1' and [hh3_relationship_e] = '7'	Person 3: Please specify your relationship with this person.	text
310	[hh3_age_e]<br><br>Show the field ONLY if:<br>[language] = '1' and [hhcount_e] > 2 and [hhcount_e] < 13	Person 3: What is this person's age?<br>*Please specify their age in years*	text (number, Min: 0, Max: 110)
311	[hh3_sex_e]<br><br>Show the field ONLY if:<br>[language] = '1' and [hhcount_e] > 2 and [hhcount_e] < 13	Person 3: What is this person's sex?	radio<br>1 Female<br>2 Male<br>3 Other
312	[hh3_race_e]<br><br>Show the field ONLY if:<br>[language] = '1' and [hhcount_e] > 2 and [hhcount_e] < 13	Person 3: What is this person's race?<br>*Select all that apply.*	checkbox<br>1 hh3_race_e___1 American Indian or Alaska Native<br>2 hh3_race_e___2 Asian

			3 hh3_race_e___3 Black or African American<br>4 hh3_race_e___4 Native Hawaiian or Pacific Islander<br>5 hh3_race_e___5 White<br>6 hh3_race_e___6 Other<br>7 hh3_race_e___7 don't know
313	[hh3_ethn_e]<br><br>Show the field ONLY if:<br>[language] = '1' and [hhcount_e] > 2 and [hhcount_e] < 13	Person 3: What is this person's ethnicity?	radio<br>1 Hispanic or Latino<br>2 Not Hispanic or Latino<br>3 Other<br>4 don't know
314	[hh3_edu_e]<br><br>Show the field ONLY if:<br>[language] = '1' and [hhcount_e] > 2 and [hhcount_e] < 13	Person 3: What is the highest level of education or schooling this person has completed?	radio<br>1 never attended school<br>2 kindergarten - 8th grade<br>3 some high school<br>4 high school equivalency (GED)<br>5 high school graduate<br>6 some college<br>7 college graduate<br>8 graduate school or more<br>9 don't know
315	[hh3_work_e]<br><br>Show the field ONLY if:<br>[language] = '1' and [hhcount_e] > 2 and [hhcount_e] < 13	Person 3: Which of the following best fit this person's current work situation?	radio<br>1 works full time<br>2 works part time<br>3 is looking for work/employment<br>4 retired<br>5 homemaker<br>6 student<br>7 on maternity/paternity leave<br>8 on illness/sick leave<br>9 on disability<br>10 other<br>11 don't know
316	[hh3_work2_e]<br><br>Show the field ONLY if:<br>[language] = '1' and [hhcount_e] > 2 and [hhcount_e] < 13	Person 3: Does this person currently consider themselves self-employed (including as an independent contractor or gig-economy worker)?	radio<br>1 yes<br>0 no<br>2 don't know
317	[hh3_work3_e]<br><br>Show the field ONLY if:<br>[language] = '1' and [hhcount_e] > 2 and [hhcount_e] < 13 and ([hh3_work_e]	Person 3: Does this person currently work in any of the following high-risk settings for COVID-19 transmission?	checkbox<br>1 hh3_work3_e___1 healthcare setting (hospital, clinic, urgent care, etc.)

	= '1' or [hh3_work_e] = '2' or [hh3_work2_e] = '1')		2 | hh3_work3_e___2 | dense residential setting (nursing home, other long-term care facility)<br>3 | hh3_work3_e___3 | prison or jail<br>4 | hh3_work3_e___4 | meatpacking facility<br>5 | hh3_work3_e___5 | shipping or distribution facility<br>6 | hh3_work3_e___6 | high-volume retail facility (grocery store, etc.)<br>7 | hh3_work3_e___7 | don't know
318	[hh3_work4_e]<br><br>Show the field ONLY if: [language] = '1' and [hhcount_e] > 2 and [hhcount_e] < 13 and ([hh3_work_e] = '1' or [hh3_work_e] = '2' or [hh3_work2_e] = '1')	Person 3: Does this person's employer offer them any of the following benefits at their current main job?<br>*Select all that apply.*	checkbox<br>1 | hh3_work4_e___1 | paid sick leave<br>2 | hh3_work4_e___2 | paid vacation/personal leave<br>3 | hh3_work4_e___3 | health insurance<br>4 | hh3_work4_e___4 | disability insurance<br>5 | hh3_work4_e___5 | retirement plan<br>6 | hh3_work4_e___6 | other<br>7 | hh3_work4_e___7 | don't know
319	[hh3_work5_e]<br><br>Show the field ONLY if: [language] = '1' and [hhcount_e] > 2 and [hhcount_e] < 13 and ([hh3_work_e] = '1' or [hh3_work_e] = '2' or [hh3_work2_e] = '1')	Person 3: On a scale of 0 (definitely not going to happen) to 10 (definitely going to happen), how likely is it that this person will lose their job because of the COVID-19 pandemic?	text (number, Min: 0, Max: 10)
320	[hh3_work6_e]<br><br>Show the field ONLY if: [language] = '1' and [hhcount_e] > 2 and [hhcount_e] < 13 and ([hh3_work_e] = '1' or [hh3_work_e] = '2' or [hh3_work2_e] = '1')	Person 3: On a scale of 0 (definitely not going to happen) to 10 (definitely going to happen), how likely is it that this person will receive fewer work hours at their job because of the COVID-19 pandemic?	text (number, Min: 0, Max: 10)
321	[hh3_work7_e]<br><br>Show the field ONLY if: [language] = '1' and [hhcount_e] > 2 and [hhcount_e] < 13 and ([hh3_work_e] = '1' or [hh3_work_e] = '2' or [hh3_work2_e] = '1')	Person 3: How often is this person required to work from outside of the home currently?	radio (Matrix)<br>1 | always (100%)<br>2 | most of the time (75%)<br>3 | half of the time (50%)<br>4 | less than half of the time (25%)<br>5 | never (0%)<br>6 | don't know
322	[hh3_work8_e]<br><br>Show the field ONLY if: [language] = '1' and ([hh3_work7_e] = '1' or [hh3_work7_e] = '2' or [hh3_wor	Person 3: How regularly is this person in close physical contact with co-workers during their work outside of the home currently?	radio (Matrix)<br>1 | always (100%)<br>2 | most of the time (75%)<br>3 | half of the time (50%)<br>4 | less than half of the time (25%)

	k7_e] = '3' or [hh3_work7_e] = '4')		5 never (0%)<br>6 don't know
323	[hh3_work9_e]<br><br>Show the field ONLY if:<br>[language] = '1' and ([hh3_work7_e] = '1' or [hh3_work7_e] = '2' or [hh3_work7_e] = '3' or [hh3_work7_e] = '4')	Person 3: How regularly is this person in close physical contact with clients during their work outside of the home currently?	radio (Matrix)<br>1 always (100%)<br>2 most of the time (75%)<br>3 half of the time (50%)<br>4 less than half of the time (25%)<br>5 never (0%)<br>6 don't know
324	[hh3_covidvaccine_e]<br><br>Show the field ONLY if:<br>[language] = '1' and [hhcount_e] > 2 and [hhcount_e] < 13	Person 3: Does this person plan to get a vaccine for COVID-19 when one becomes available?	radio<br>1 yes<br>0 no<br>2 don't know
325	[hh3_covidsymp_e]<br><br>Show the field ONLY if:<br>[language] = '1' and [hhcount_e] > 2 and [hhcount_e] < 13	Person 3: Has this person had any symptoms (cough, fever, difficulty breathing, fatigue, body aches, diarrhea, runny nose, loss of smell or taste) consistent with COVID-19 in the last two weeks?	radio<br>1 yes<br>0 no<br>2 don't know
326	[hh3_covidsymp2_e]<br><br>Show the field ONLY if:<br>[language] = '1' and [hh3_covidsymp_e] = '1'	Person 3: When did this person's symptoms begin?	text (date_mdy)
327	[hh3_covidsymp3_e]<br><br>Show the field ONLY if:<br>[language] = '1' and [hh3_covidsymp_e] = '1'	Person 3: Is this person worried that they may have had COVID-19 because of their symptoms?	radio<br>1 yes<br>0 no<br>2 don't know
328	[hh3_covidsymp4_e]<br><br>Show the field ONLY if:<br>[language] = '1' and [hh3_covidsymp_e] = '1'	Person 3: Did this person experience any bias or discrimination because of their symptoms?	radio<br>1 yes<br>0 no<br>2 don't know
329	[hh3_covidsymp5_e]<br><br>Show the field ONLY if:<br>[language] = '1' and [hh3_covidsymp_e] = '1'	Person 3: What did this person do in response to their symptoms?<br>*Select all that apply.*	checkbox<br>0 hh3_covidsymp5_e___0 nothing<br>1 hh3_covidsymp5_e___1 took over the counter medication (ibuprofen, acetaminophen, etc.)<br>2 hh3_covidsymp5_e___2 communicated with a health care provider over the phone<br>3 hh3_covidsymp5_e___3 visited a health care provider's office<br>4 hh3_covidsymp5_e___4 visited a retail clinic or

			pharmacy<br>5 hh3_covidsymp5_e___5 visited urgent care (FASTMed, etc.)<br>6 hh3_covidsymp5_e___6 visited the emergency room<br>7 hh3_covidsymp5_e___7 was admitted to the hospital<br>8 hh3_covidsymp5_e___8 other<br>9 hh3_covidsymp5_e___9 don't know
330	[hh3_covidsymp6_e]<br><br>Show the field ONLY if:<br>[language] = '1' and [hh3_covidsymp5_e(8)] = '1'	Person 3: Please specify what other action this person took in response to their symptoms.	text
331	[hh3_covidsymp7_e]<br><br>Show the field ONLY if:<br>[language] = '1' and ([hh3_covidsymp5_e(2)] = '1' or [hh3_covidsymp5_e(3)] = '1' or [hh3_covidsymp5_e(4)] = '1' or [hh3_covidsymp5_e(5)] = '1' or [hh3_covidsymp5_e(6)] = '1' or [hh3_covidsymp5_e(7)] = '1' or [hh3_covidsymp5_e(8)] = '1')	Person 3: Did a health care provider tell this person that they may have COVID-19?	radio<br>1 yes<br>0 no<br>2 don't know
332	[hh3_covid_test_e]<br><br>Show the field ONLY if:<br>[language] = '1' and [hh3_covidsymp_e] = '1'	Person 3: If this person received a COVID-19 test due to their symptoms, what was the result?	radio<br>1 pending<br>2 positive<br>3 negative<br>4 inconclusive<br>5 did not receive a test<br>6 don't know
333	[hh3_covid_admit_e]<br><br>Show the field ONLY if:<br>[language] = '1' and [hh3_covidsymp5_e(7)] = '1'	Person 3: How many days was this person admitted to the hospital?	text (number, Min: 0)
334	[hh3_covid_admit2_e]<br><br>Show the field ONLY if:<br>[language] = '1' and [hh3_covidsymp5_e(7)] = '1'	Person 3: Did this person receive any of the following interventions during their hospital admission?	checkbox<br>1 hh3_covid_admit2_e___1 extra oxygen in your nose<br>2 hh3_covid_admit2_e___2 treatment in the intensive care unit (ICU)<br>3 hh3_covid_admit2_e___3 mechanical ventilation (intubation or a breathing tube)<br>4 hh3_covid_admit2_e___4 don't know

335	[hh3_covidsymp8_e]<br><br>Show the field ONLY if:<br>[language] = '1' and [hh3_covidsymp_e] = '1'	Person 3: Has this person returned to their normal health at this time?	radio<br>1 yes<br>0 no<br>2 don't know
336	[hh3_prevent_e]<br><br>Show the field ONLY if:<br>[language] = '1' and [hh3_covidsymp_e] = '1'	Person 3: Which of the following did this person do to protect their friends and family after their symptoms began?	checkbox<br>1 hh3_prevent_e___1 wore a mask more frequently<br>2 hh3_prevent_e___2 washed your hands with soap and water more frequently<br>3 hh3_prevent_e___3 used hand sanitizer more frequently<br>4 hh3_prevent_e___4 isolated yourself in your home more frequently<br>5 hh3_prevent_e___5 stayed home more frequently<br>6 hh3_prevent_e___6 wore disposable gloves more frequently<br>7 hh3_prevent_e___7 don't know
337	[hh4_relationship_e]<br><br>Show the field ONLY if:<br>[language] = '1' and [hhcount_e] > 3 and [hhcount_e] < 13	Section Header: *For each additional person in the your household, please provide the following information.*<br><br>Person 4: What is your relationship to this person?	radio<br>1 partner or spouse<br>2 child<br>3 parent<br>4 sibling<br>5 other family member<br>6 in-home childcare provider or other caregiver<br>7 other
338	[hh4_relationship2_e]<br><br>Show the field ONLY if:<br>[language] = '1' and [hh4_relationship_e] = '7'	Person 4: Please specify your relationship with this person.	text
339	[hh4_age_e]<br><br>Show the field ONLY if:<br>[language] = '1' and [hhcount_e] > 3 and [hhcount_e] < 13	Person 4: What is this person's age?<br>*Please specify their age in years*	text (number, Min: 0, Max: 110)
340	[hh4_sex_e]<br><br>Show the field ONLY if:<br>[language] = '1' and [hhcount_e] > 3 and [hhcount_e] < 13	Person 4: What is this person's sex?	radio<br>1 Female<br>2 Male<br>3 Other
341	[hh4_race_e]<br><br>Show the field ONLY if:<br>[language] = '1' and [hhcount_e] > 3 and [hhcount_e] < 13	Person 4: What is this person's race?<br>*Select all that apply.*	checkbox<br>1 hh4_race_e___1 American Indian or Alaska Native<br>2 hh4_race_e___2 Asian

			3 hh4_race_e___3 Black or African American<br>4 hh4_race_e___4 Native Hawaiian or Pacific Islander<br>5 hh4_race_e___5 White<br>6 hh4_race_e___6 Other<br>7 hh4_race_e___7 don't know
342	[hh4_ethn_e]<br>Show the field ONLY if:<br>[language] = '1' and [hhcount_e] > 3 and [hhcount_e] < 13	Person 4: What is this person's ethnicity?	radio<br>1 Hispanic or Latino<br>2 Not Hispanic or Latino<br>3 Other<br>4 don't know
343	[hh4_edu_e]<br>Show the field ONLY if:<br>[language] = '1' and [hhcount_e] > 3 and [hhcount_e] < 13	Person 4: What is the highest level of education or schooling this person has completed?	radio<br>1 never attended school<br>2 kindergarten - 8th grade<br>3 some high school<br>4 high school equivalency (GED)<br>5 high school graduate<br>6 some college<br>7 college graduate<br>8 graduate school or more<br>9 don't know
344	[hh4_work_e]<br>Show the field ONLY if:<br>[language] = '1' and [hhcount_e] > 3 and [hhcount_e] < 13	Person 4: Which of the following best fit this person's current work situation?	radio<br>1 works full time<br>2 works part time<br>3 is looking for work/employment<br>4 retired<br>5 homemaker<br>6 student<br>7 on maternity/paternity leave<br>8 on illness/sick leave<br>9 on disability<br>10 other<br>11 don't know
345	[hh4_work2_e]<br>Show the field ONLY if:<br>[language] = '1' and [hhcount_e] > 3 and [hhcount_e] < 13	Person 4: Does this person currently consider themselves self-employed (including as an independent contractor or gig-economy worker)?	radio<br>1 yes<br>0 no<br>2 don't know
346	[hh4_work3_e]<br>Show the field ONLY if:<br>[language] = '1' and [hhcount_e] > 3 and [hhcount_e] < 13 and ([hh4_work_e]	Person 4: Does this person currently work in any of the following high-risk settings for COVID-19 transmission?	checkbox<br>1 hh4_work3_e___1 healthcare setting (hospital, clinic, urgent care, etc.)

	= '1' or [hh4_work_e] = '2' or [hh4_work2_e] = '1')		2 hh4_work3_e___2 dense residential setting (nursing home, other long-term care facility)<br>3 hh4_work3_e___3 prison or jail<br>4 hh4_work3_e___4 meatpacking facility<br>5 hh4_work3_e___5 shipping or distribution facility<br>6 hh4_work3_e___6 high-volume retail facility (grocery store, etc.)<br>7 hh4_work3_e___7 don't know
347	[hh4_work4_e]<br><br>Show the field ONLY if: [language] = '1' and [hhcount_e] > 3 and [hhcount_e] < 13 and ([hh4_work_e] = '1' or [hh4_work_e] = '2' or [hh4_work2_e] = '1')	Person 4: Does this person's employer offer them any of the following benefits at their current main job?<br>*Select all that apply.*	checkbox<br>1 hh4_work4_e___1 paid sick leave<br>2 hh4_work4_e___2 paid vacation/personal leave<br>3 hh4_work4_e___3 health insurance<br>4 hh4_work4_e___4 disability insurance<br>5 hh4_work4_e___5 retirement plan<br>6 hh4_work4_e___6 other<br>7 hh4_work4_e___7 don't know
348	[hh4_work5_e]<br><br>Show the field ONLY if: [language] = '1' and [hhcount_e] > 3 and [hhcount_e] < 13 and ([hh4_work_e] = '1' or [hh4_work_e] = '2' or [hh4_work2_e] = '1')	Person 4: On a scale of 0 (definitely not going to happen) to 10 (definitely going to happen), how likely is it that this person will lose their job because of the COVID-19 pandemic?	text (number, Min: 0, Max: 10)
349	[hh4_work6_e]<br><br>Show the field ONLY if: [language] = '1' and [hhcount_e] > 3 and [hhcount_e] < 13 and ([hh4_work_e] = '1' or [hh4_work_e] = '2' or [hh4_work2_e] = '1')	Person 4: On a scale of 0 (definitely not going to happen) to 10 (definitely going to happen), how likely is it that this person will receive fewer work hours at their job because of the COVID-19 pandemic?	text (number, Min: 0, Max: 10)
350	[hh4_work7_e]<br><br>Show the field ONLY if: [language] = '1' and [hhcount_e] > 3 and [hhcount_e] < 13 and ([hh4_work_e] = '1' or [hh4_work_e] = '2' or [hh4_work2_e] = '1')	Person 4: How often is this person required to work from outside of the home currently?	radio (Matrix)<br>1 always (100%)<br>2 most of the time (75%)<br>3 half of the time (50%)<br>4 less than half of the time (25%)<br>5 never (0%)<br>6 don't know
351	[hh4_work8_e]<br><br>Show the field ONLY if: [language] = '1' and ([hh4_work7_e] = '1' or [hh4_work7_e] = '2' or [hh4_wor	Person 4: How regularly is this person in close physical contact with co-workers during their work outside of the home currently?	radio (Matrix)<br>1 always (100%)<br>2 most of the time (75%)<br>3 half of the time (50%)<br>4 less than half of the time (25%)

	k7_e] = '3' or [hh4_work7_e] = '4')		5 never (0%)<br>6 don't know
352	[hh4_work9_e]<br><br>Show the field ONLY if:<br>[language] = '1' and ([hh4_work7_e] = '1' or [hh4_work7_e] = '2' or [hh4_work7_e] = '3' or [hh4_work7_e] = '4')	Person 4: How regularly is this person in close physical contact with clients during their work outside of the home currently?	radio (Matrix)<br>1 always (100%)<br>2 most of the time (75%)<br>3 half of the time (50%)<br>4 less than half of the time (25%)<br>5 never (0%)<br>6 don't know
353	[hh4_covidvaccine_e]<br><br>Show the field ONLY if:<br>[language] = '1' and [hhcount_e] > 3 and [hhcount_e] < 13	Person 4: Does this person plan to get a vaccine for COVID-19 when one becomes available?	radio<br>1 yes<br>0 no<br>2 don't know
354	[hh4_covidsymp_e]<br><br>Show the field ONLY if:<br>[language] = '1' and [hhcount_e] > 3 and [hhcount_e] < 13	Person 4: Has this person had any symptoms (cough, fever, difficulty breathing, fatigue, body aches, diarrhea, runny nose, loss of smell or taste) consistent with COVID-19 in the last two weeks?	radio<br>1 yes<br>0 no<br>2 don't know
355	[hh4_covidsymp2_e]<br><br>Show the field ONLY if:<br>[language] = '1' and [hh4_covidsymp_e] = '1'	Person 4: When did this person's symptoms begin?	text (date_mdy)
356	[hh4_covidsymp3_e]<br><br>Show the field ONLY if:<br>[language] = '1' and [hh4_covidsymp_e] = '1'	Person 4: Is this person worried that they may have had COVID-19 because of their symptoms?	radio<br>1 yes<br>0 no<br>2 don't know
357	[hh4_covidsymp4_e]<br><br>Show the field ONLY if:<br>[language] = '1' and [hh4_covidsymp_e] = '1'	Person 4: Did this person experience any bias or discrimination because of their symptoms?	radio<br>1 yes<br>0 no<br>2 don't know
358	[hh4_covidsymp5_e]<br><br>Show the field ONLY if:<br>[language] = '1' and [hh4_covidsymp_e] = '1'	Person 4: What did this person do in response to their symptoms?<br>*Select all that apply.*	checkbox<br>0 hh4_covidsymp5_e___0 nothing<br>1 hh4_covidsymp5_e___1 took over the counter medication (ibuprofen, acetaminophen, etc.)<br>2 hh4_covidsymp5_e___2 communicated with a health care provider over the phone<br>3 hh4_covidsymp5_e___3 visited a health care provider's office<br>4 hh4_covidsymp5_e___4 visited a retail clinic or

			pharmacy<br>5 hh4_covidsymp5_e___5 visited urgent care (FASTMed, etc.)<br>6 hh4_covidsymp5_e___6 visited the emergency room<br>7 hh4_covidsymp5_e___7 was admitted to the hospital<br>8 hh4_covidsymp5_e___8 other<br>9 hh4_covidsymp5_e___9 don't know
359	[hh4_covidsymp6_e]<br>Show the field ONLY if:<br>[language] = '1' and [hh4_covidsymp5_e(8)] = '1'	Person 4: Please specify what other action this person took in response to their symptoms.	text
360	[hh4_covidsymp7_e]<br>Show the field ONLY if:<br>[language] = '1' and ([hh4_covidsymp5_e(2)] = '1' or [hh4_covidsymp5_e(3)] = '1' or [hh4_covidsymp5_e(4)] = '1' or [hh4_covidsymp5_e(5)] = '1' or [hh4_covidsymp5_e(6)] = '1' or [hh4_covidsymp5_e(7)] = '1' or [hh4_covidsymp5_e(8)] = '1')	Person 4: Did a health care provider tell this person that they may have COVID-19?	radio<br>1 yes<br>0 no<br>2 don't know
361	[hh4_covid_test_e]<br>Show the field ONLY if:<br>[language] = '1' and [hh4_covidsymp_e] = '1'	Person 4: If this person received a COVID-19 test due to their symptoms, what was the result?	radio<br>1 pending<br>2 positive<br>3 negative<br>4 inconclusive<br>5 did not receive a test<br>6 don't know
362	[hh4_covid_admit_e]<br>Show the field ONLY if:<br>[language] = '1' and [hh4_covidsymp5_e(7)] = '1'	Person 4: How many days was this person admitted to the hospital?	text (number, Min: 0)
363	[hh4_covid_admit2_e]<br>Show the field ONLY if:<br>[language] = '1' and [hh4_covidsymp5_e(7)] = '1'	Person 4: Did this person receive any of the following interventions during their hospital admission?	checkbox<br>1 hh4_covid_admit2_e___1 extra oxygen in your nose<br>2 hh4_covid_admit2_e___2 treatment in the intensive care unit (ICU)<br>3 hh4_covid_admit2_e___3 mechanical ventilation (intubation or a breathing tube)<br>4 hh4_covid_admit2_e___4 don't know

364	[hh4_covidsymp8_e]<br><br>Show the field ONLY if:<br>[language] = '1' and [hh4_covidsymp_e] = '1'	Person 4: Has this person returned to their normal health at this time?	radio<br>1 yes<br>0 no<br>2 don't know
365	[hh4_prevent_e]<br><br>Show the field ONLY if:<br>[language] = '1' and [hh4_covidsymp_e] = '1'	Person 4: Which of the following did this person do to protect their friends and family after their symptoms began?	checkbox<br>1 hh4_prevent_e___1 wore a mask more frequently<br>2 hh4_prevent_e___2 washed your hands with soap and water more frequently<br>3 hh4_prevent_e___3 used hand sanitizer more frequently<br>4 hh4_prevent_e___4 isolated yourself in your home more frequently<br>5 hh4_prevent_e___5 stayed home more frequently<br>6 hh4_prevent_e___6 wore disposable gloves more frequently<br>7 hh4_prevent_e___7 don't know
366	[hh5_relationship_e]<br><br>Show the field ONLY if:<br>[language] = '1' and [hhcount_e] > 4 and [hhcount_e] < 13	Section Header: *For each additional person in the your household, please provide the following information.*<br><br>Person 5: What is your relationship to this person?	radio<br>1 partner or spouse<br>2 child<br>3 parent<br>4 sibling<br>5 other family member<br>6 in-home childcare provider or other caregiver<br>7 other
367	[hh5_relationship2_e]<br><br>Show the field ONLY if:<br>[language] = '1' and [hh5_relationship_e] = '7'	Person 5: Please specify your relationship with this person.	text
368	[hh5_age_e]<br><br>Show the field ONLY if:<br>[language] = '1' and [hhcount_e] > 4 and [hhcount_e] < 13	Person 5: What is this person's age?<br>*Please specify their age in years*	text (number, Min: 0, Max: 110)
369	[hh5_sex_e]<br><br>Show the field ONLY if:<br>[language] = '1' and [hhcount_e] > 4 and [hhcount_e] < 13	Person 5: What is this person's sex?	radio<br>1 Female<br>2 Male<br>3 Other
370	[hh5_race_e]<br><br>Show the field ONLY if:<br>[language] = '1' and [hhcount_e] > 4 and [hhcount_e] < 13	Person 5: What is this person's race?<br>*Select all that apply.*	checkbox<br>1 hh5_race_e___1 American Indian or Alaska Native<br>2 hh5_race_e___2 Asian

			3 hh5_race_e___3 Black or African American<br>4 hh5_race_e___4 Native Hawaiian or Pacific Islander<br>5 hh5_race_e___5 White<br>6 hh5_race_e___6 Other<br>7 hh5_race_e___7 don't know
371	[hh5_ethn_e]<br><br>Show the field ONLY if:<br>[language] = '1' and [hhcount_e] > 4 and [hhcount_e] < 13	Person 5: What is this person's ethnicity?	radio<br>1 Hispanic or Latino<br>2 Not Hispanic or Latino<br>3 Other<br>4 don't know
372	[hh5_edu_e]<br><br>Show the field ONLY if:<br>[language] = '1' and [hhcount_e] > 4 and [hhcount_e] < 13	Person 5: What is the highest level of education or schooling this person has completed?	radio<br>1 never attended school<br>2 kindergarten - 8th grade<br>3 some high school<br>4 high school equivalency (GED)<br>5 high school graduate<br>6 some college<br>7 college graduate<br>8 graduate school or more<br>9 don't know
373	[hh5_work_e]<br><br>Show the field ONLY if:<br>[language] = '1' and [hhcount_e] > 4 and [hhcount_e] < 13	Person 5: Which of the following best fit this person's current work situation?	radio<br>1 works full time<br>2 works part time<br>3 is looking for work/employment<br>4 retired<br>5 homemaker<br>6 student<br>7 on maternity/paternity leave<br>8 on illness/sick leave<br>9 on disability<br>10 other<br>11 don't know
374	[hh5_work2_e]<br><br>Show the field ONLY if:<br>[language] = '1' and [hhcount_e] > 4 and [hhcount_e] < 13	Person 5: Does this person currently consider themselves self-employed (including as an independent contractor or gig-economy worker)?	radio<br>1 yes<br>0 no<br>2 don't know
375	[hh5_work3_e]<br><br>Show the field ONLY if:<br>[language] = '1' and [hhcount_e] > 4 and [hhcount_e] < 13 and ([hh5_work_e]	Person 5: Does this person currently work in any of the following high-risk settings for COVID-19 transmission?	checkbox<br>1 hh5_work3_e___1 healthcare setting (hospital, clinic, urgent care, etc.)

	= '1' or [hh5_work_e] = '2' or [hh5_work2_e] = '1')		2 hh5_work3_e___2 dense residential setting (nursing home, other long-term care facility)<br>3 hh5_work3_e___3 prison or jail<br>4 hh5_work3_e___4 meatpacking facility<br>5 hh5_work3_e___5 shipping or distribution facility<br>6 hh5_work3_e___6 high-volume retail facility (grocery store, etc.)<br>7 hh5_work3_e___7 don't know
376	[hh5_work4_e]<br>Show the field ONLY if: [language] = '1' and [hhcount_e] > 4 and [hhcount_e] < 13 and ([hh5_work_e] = '1' or [hh5_work_e] = '2' or [hh5_work2_e] = '1')	Person 5: Does this person's employer offer them any of the following benefits at their current main job?<br>*Select all that apply.*	checkbox<br>1 hh5_work4_e___1 paid sick leave<br>2 hh5_work4_e___2 paid vacation/personal leave<br>3 hh5_work4_e___3 health insurance<br>4 hh5_work4_e___4 disability insurance<br>5 hh5_work4_e___5 retirement plan<br>6 hh5_work4_e___6 other<br>7 hh5_work4_e___7 don't know
377	[hh5_work5_e]<br>Show the field ONLY if: [language] = '1' and [hhcount_e] > 4 and [hhcount_e] < 13 and ([hh5_work_e] = '1' or [hh5_work_e] = '2' or [hh5_work2_e] = '1')	Person 5: On a scale of 0 (definitely not going to happen) to 10 (definitely going to happen), how likely is it that this person will lose their job because of the COVID-19 pandemic?	text (number, Min: 0, Max: 10)
378	[hh5_work6_e]<br>Show the field ONLY if: [language] = '1' and [hhcount_e] > 4 and [hhcount_e] < 13 and ([hh5_work_e] = '1' or [hh5_work_e] = '2' or [hh5_work2_e] = '1')	Person 5: On a scale of 0 (definitely not going to happen) to 10 (definitely going to happen), how likely is it that this person will receive fewer work hours at their job because of the COVID-19 pandemic?	text (number, Min: 0, Max: 10)
379	[hh5_work7_e]<br>Show the field ONLY if: [language] = '1' and [hhcount_e] > 4 and [hhcount_e] < 13 and ([hh5_work_e] = '1' or [hh5_work_e] = '2' or [hh5_work2_e] = '1')	Person 5: How often is this person required to work from outside of the home currently?	radio (Matrix)<br>1 always (100%)<br>2 most of the time (75%)<br>3 half of the time (50%)<br>4 less than half of the time (25%)<br>5 never (0%)<br>6 don't know
380	[hh5_work8_e]<br>Show the field ONLY if: [language] = '1' and ([hh5_work7_e] = '1' or [hh5_work7_e] = '2' or [hh5_wor	Person 5: How regularly is this person in close physical contact with co-workers during their work outside of the home currently?	radio (Matrix)<br>1 always (100%)<br>2 most of the time (75%)<br>3 half of the time (50%)<br>4 less than half of the time (25%)

	k7_e] = '3' or [hh5_work7_e] = '4')		5 never (0%)<br>6 don't know
381	[hh5_work9_e]<br><br>Show the field ONLY if:<br>[language] = '1' and ([hh5_work7_e] = '1' or [hh5_work7_e] = '2' or [hh5_work7_e] = '3' or [hh5_work7_e] = '4')	Person 5: How regularly is this person in close physical contact with clients during their work outside of the home currently?	radio (Matrix)<br>1 always (100%)<br>2 most of the time (75%)<br>3 half of the time (50%)<br>4 less than half of the time (25%)<br>5 never (0%)<br>6 don't know
382	[hh5_covidvaccine_e]<br><br>Show the field ONLY if:<br>[language] = '1' and [hhcount_e] > 4 and [hhcount_e] < 13	Person 5: Does this person plan to get a vaccine for COVID-19 when one becomes available?	radio<br>1 yes<br>0 no<br>2 don't know
383	[hh5_covidsymp_e]<br><br>Show the field ONLY if:<br>[language] = '1' and [hhcount_e] > 4 and [hhcount_e] < 13	Person 5: Has this person had any symptoms (cough, fever, difficulty breathing, fatigue, body aches, diarrhea, runny nose, loss of smell or taste) consistent with COVID-19 in the last two weeks?	radio<br>1 yes<br>0 no<br>2 don't know
384	[hh5_covidsymp2_e]<br><br>Show the field ONLY if:<br>[language] = '1' and [hh5_covidsymp_e] = '1'	Person 5: When did this person's symptoms begin?	text (date_mdy)
385	[hh5_covidsymp3_e]<br><br>Show the field ONLY if:<br>[language] = '1' and [hh5_covidsymp_e] = '1'	Person 5: Is this person worried that they may have had COVID-19 because of their symptoms?	radio<br>1 yes<br>0 no<br>2 don't know
386	[hh5_covidsymp4_e]<br><br>Show the field ONLY if:<br>[language] = '1' and [hh5_covidsymp_e] = '1'	Person 5: Did this person experience any bias or discrimination because of their symptoms?	radio<br>1 yes<br>0 no<br>2 don't know
387	[hh5_covidsymp5_e]<br><br>Show the field ONLY if:<br>[language] = '1' and [hh5_covidsymp_e] = '1'	Person 5: What did this person do in response to their symptoms?<br>*Select all that apply.*	checkbox<br>0 hh5_covidsymp5_e___0 nothing<br>1 hh5_covidsymp5_e___1 took over the counter medication (ibuprofen, acetaminophen, etc.)<br>2 hh5_covidsymp5_e___2 communicated with a health care provider over the phone<br>3 hh5_covidsymp5_e___3 visited a health care provider's office<br>4 hh5_covidsymp5_e___4 visited a retail clinic or

			pharmacy<br>5 hh5_covidsymp5_e___5 visited urgent care (FASTMed, etc.)<br>6 hh5_covidsymp5_e___6 visited the emergency room<br>7 hh5_covidsymp5_e___7 was admitted to the hospital<br>8 hh5_covidsymp5_e___8 other<br>9 hh5_covidsymp5_e___9 don't know
388	[hh5_covidsymp6_e]<br><br>Show the field ONLY if: [language] = '1' and [hh5_covidsymp5_e(8)] = '1'	Person 5: Please specify what other action this person took in response to their symptoms.	text
389	[hh5_covidsymp7_e]<br><br>Show the field ONLY if: [language] = '1' and ([hh5_covidsymp5_e(2)] = '1' or [hh5_covidsymp5_e(3)] = '1' or [hh5_covidsymp5_e(4)] = '1' or [hh5_covidsymp5_e(5)] = '1' or [hh5_covidsymp5_e(6)] = '1' or [hh5_covidsymp5_e(7)] = '1' or [hh5_covidsymp5_e(8)] = '1')	Person 5: Did a health care provider tell this person that they may have COVID-19?	radio<br>1 yes<br>0 no<br>2 don't know
390	[hh5_covid_test_e]<br><br>Show the field ONLY if: [language] = '1' and [hh5_covidsymp_e] = '1'	Person 5: If this person received a COVID-19 test due to their symptoms, what was the result?	radio<br>1 pending<br>2 positive<br>3 negative<br>4 inconclusive<br>5 did not receive a test<br>6 don't know
391	[hh5_covid_admit_e]<br><br>Show the field ONLY if: [language] = '1' and [hh5_covidsymp5_e(7)] = '1'	Person 5: How many days was this person admitted to the hospital?	text (number, Min: 0)
392	[hh5_covid_admit2_e]<br><br>Show the field ONLY if: [language] = '1' and [hh5_covidsymp5_e(7)] = '1'	Person 5: Did this person receive any of the following interventions during their hospital admission?	checkbox<br>1 hh5_covid_admit2_e___1 extra oxygen in your nose<br>2 hh5_covid_admit2_e___2 treatment in the intensive care unit (ICU)<br>3 hh5_covid_admit2_e___3 mechanical ventilation (intubation or a breathing tube)<br>4 hh5_covid_admit2_e___4 don't know

<table>
<tr><td>393</td><td>[hh5_covidsymp8_e]

Show the field ONLY if:
[language] = '1' and [hh5_covidsymp_e] = '1'</td><td>Person 5: Has this person returned to their normal health at this time?</td><td>radio
1 yes
0 no
2 don't know</td></tr>
<tr><td>394</td><td>[hh5_prevent_e]

Show the field ONLY if:
[language] = '1' and [hh5_covidsymp_e] = '1'</td><td>Person 5: Which of the following did this person do to protect their friends and family after their symptoms began?</td><td>checkbox
1 hh5_prevent_e___1 wore a mask more frequently
2 hh5_prevent_e___2 washed your hands with soap and water more frequently
3 hh5_prevent_e___3 used hand sanitizer more frequently
4 hh5_prevent_e___4 isolated yourself in your home more frequently
5 hh5_prevent_e___5 stayed home more frequently
6 hh5_prevent_e___6 wore disposable gloves more frequently
7 hh5_prevent_e___7 don't know</td></tr>
<tr><td>395</td><td>[hh6_relationship_e]

Show the field ONLY if:
[language] = '1' and [hhcount_e] > 5 and [hhcount_e] < 13</td><td>Section Header: For each additional person in the your household, please provide the following information.

Person 6: What is your relationship to this person?</td><td>radio
1 partner or spouse
2 child
3 parent
4 sibling
5 other family member
6 in-home childcare provider or other caregiver
7 other</td></tr>
<tr><td>396</td><td>[hh6_relationship2_e]

Show the field ONLY if:
[language] = '1' and [hh6_relationship_e] = '7'</td><td>Person 6: Please specify your relationship with this person.</td><td>text</td></tr>
<tr><td>397</td><td>[hh6_age_e]

Show the field ONLY if:
[language] = '1' and [hhcount_e] > 5 and [hhcount_e] < 13</td><td>Person 6: What is this person's age?
Please specify their age in years</td><td>text (number, Min: 0, Max: 110)</td></tr>
<tr><td>398</td><td>[hh6_sex_e]

Show the field ONLY if:
[language] = '1' and [hhcount_e] > 5 and [hhcount_e] < 13</td><td>Person 6: What is this person's sex?</td><td>radio
1 Female
2 Male
3 Other</td></tr>
<tr><td>399</td><td>[hh6_race_e]

Show the field ONLY if:
[language] = '1' and [hhcount_e] > 5 and [hhcount_e] < 13</td><td>Person 6: What is this person's race?
Select all that apply.</td><td>checkbox
1 hh6_race_e___1 American Indian or Alaska Native
2 hh6_race_e___2 Asian</td></tr>
</table>

			3 | hh6_race_e___3 | Black or African American<br>4 | hh6_race_e___4 | Native Hawaiian or Pacific Islander<br>5 | hh6_race_e___5 | White<br>6 | hh6_race_e___6 | Other<br>7 | hh6_race_e___7 | don't know
400	[hh6_ethn_e]<br><br>Show the field ONLY if:<br>[language] = '1' and [hhcount_e] > 5 and [hhcount_e] < 13	Person 6: What is this person's ethnicity?	radio<br>1 | Hispanic or Latino<br>2 | Not Hispanic or Latino<br>3 | Other<br>4 | don't know
401	[hh6_edu_e]<br><br>Show the field ONLY if:<br>[language] = '1' and [hhcount_e] > 5 and [hhcount_e] < 13	Person 6: What is the highest level of education or schooling this person has completed?	radio<br>1 | never attended school<br>2 | kindergarten - 8th grade<br>3 | some high school<br>4 | high school equivalency (GED)<br>5 | high school graduate<br>6 | some college<br>7 | college graduate<br>8 | graduate school or more<br>9 | don't know
402	[hh6_work_e]<br><br>Show the field ONLY if:<br>[language] = '1' and [hhcount_e] > 5 and [hhcount_e] < 13	Person 6: Which of the following best fit this person's current work situation?	radio<br>1 | works full time<br>2 | works part time<br>3 | is looking for work/employment<br>4 | retired<br>5 | homemaker<br>6 | student<br>7 | on maternity/paternity leave<br>8 | on illness/sick leave<br>9 | on disability<br>10 | other<br>11 | don't know
403	[hh6_work2_e]<br><br>Show the field ONLY if:<br>[language] = '1' and [hhcount_e] > 5 and [hhcount_e] < 13	Person 6: Does this person currently consider themselves self-employed (including as an independent contractor or gig-economy worker)?	radio<br>1 | yes<br>0 | no<br>2 | don't know
404	[hh6_work3_e]<br><br>Show the field ONLY if:<br>[language] = '1' and [hhcount_e] > 5 and [hhcount_e] < 13 and ([hh6_work_e]	Person 6: Does this person currently work in any of the following high-risk settings for COVID-19 transmission?	checkbox<br>1 | hh6_work3_e___1 | healthcare setting (hospital, clinic, urgent care, etc.)

	= '1' or [hh6_work_e] = '2' or [hh6_work2_e] = '1')		2 hh6_work3_e___2 dense residential setting (nursing home, other long-term care facility)<br>3 hh6_work3_e___3 prison or jail<br>4 hh6_work3_e___4 meatpacking facility<br>5 hh6_work3_e___5 shipping or distribution facility<br>6 hh6_work3_e___6 high-volume retail facility (grocery store, etc.)<br>7 hh6_work3_e___7 don't know
405	[hh6_work4_e]<br><br>Show the field ONLY if: [language] = '1' and [hhcount_e] > 5 and [hhcount_e] < 13 and ([hh6_work_e] = '1' or [hh6_work_e] = '2' or [hh6_work2_e] = '1')	Person 6: Does this person's employer offer them any of the following benefits at their current main job?<br>*Select all that apply.*	checkbox<br>1 hh6_work4_e___1 paid sick leave<br>2 hh6_work4_e___2 paid vacation/personal leave<br>3 hh6_work4_e___3 health insurance<br>4 hh6_work4_e___4 disability insurance<br>5 hh6_work4_e___5 retirement plan<br>6 hh6_work4_e___6 other<br>7 hh6_work4_e___7 don't know
406	[hh6_work5_e]<br><br>Show the field ONLY if: [language] = '1' and [hhcount_e] > 5 and [hhcount_e] < 13 and ([hh6_work_e] = '1' or [hh6_work_e] = '2' or [hh6_work2_e] = '1')	Person 6: On a scale of 0 (definitely not going to happen) to 10 (definitely going to happen), how likely is it that this person will lose their job because of the COVID-19 pandemic?	text (number, Min: 0, Max: 10)
407	[hh6_work6_e]<br><br>Show the field ONLY if: [language] = '1' and [hhcount_e] > 5 and [hhcount_e] < 13 and ([hh6_work_e] = '1' or [hh6_work_e] = '2' or [hh6_work2_e] = '1')	Person 6: On a scale of 0 (definitely not going to happen) to 10 (definitely going to happen), how likely is it that this person will receive fewer work hours at their job because of the COVID-19 pandemic?	text (number, Min: 0, Max: 10)
408	[hh6_work7_e]<br><br>Show the field ONLY if: [language] = '1' and [hhcount_e] > 5 and [hhcount_e] < 13 and ([hh6_work_e] = '1' or [hh6_work_e] = '2' or [hh6_work2_e] = '1')	Person 6: How often is this person required to work from outside of the home currently?	radio (Matrix)<br>1 always (100%)<br>2 most of the time (75%)<br>3 half of the time (50%)<br>4 less than half of the time (25%)<br>5 never (0%)<br>6 don't know
409	[hh6_work8_e]<br><br>Show the field ONLY if: [language] = '1' and ([hh6_work7_e] = '1' or [hh6_work7_e] = '2' or [hh6_wor	Person 6: How regularly is this person in close physical contact with co-workers during their work outside of the home currently?	radio (Matrix)<br>1 always (100%)<br>2 most of the time (75%)<br>3 half of the time (50%)<br>4 less than half of the time (25%)

	k7_e] = '3' or [hh6_work7_e] = '4')		5 never (0%)<br>6 don't know
410	[hh6_work9_e]<br><br>Show the field ONLY if:<br>[language] = '1' and ([hh6_work7_e] = '1' or [hh6_work7_e] = '2' or [hh6_work7_e] = '3' or [hh6_work7_e] = '4')	Person 6: How regularly is this person in close physical contact with clients during their work outside of the home currently?	radio (Matrix)<br>1 always (100%)<br>2 most of the time (75%)<br>3 half of the time (50%)<br>4 less than half of the time (25%)<br>5 never (0%)<br>6 don't know
411	[hh6_covidvaccine_e]<br><br>Show the field ONLY if:<br>[language] = '1' and [hhcount_e] > 5 and [hhcount_e] < 13	Person 6: Does this person plan to get a vaccine for COVID-19 when one becomes available?	radio<br>1 yes<br>0 no<br>2 don't know
412	[hh6_covidsymp_e]<br><br>Show the field ONLY if:<br>[language] = '1' and [hhcount_e] > 5 and [hhcount_e] < 13	Person 6: Has this person had any symptoms (cough, fever, difficulty breathing, fatigue, body aches, diarrhea, runny nose, loss of smell or taste) consistent with COVID-19 in the last two weeks?	radio<br>1 yes<br>0 no<br>2 don't know
413	[hh6_covidsymp2_e]<br><br>Show the field ONLY if:<br>[language] = '1' and [hh6_covidsymp_e] = '1'	Person 6: When did this person's symptoms begin?	text (date_mdy)
414	[hh6_covidsymp3_e]<br><br>Show the field ONLY if:<br>[language] = '1' and [hh6_covidsymp_e] = '1'	Person 6: Is this person worried that they may have had COVID-19 because of their symptoms?	radio<br>1 yes<br>0 no<br>2 don't know
415	[hh6_covidsymp4_e]<br><br>Show the field ONLY if:<br>[language] = '1' and [hh6_covidsymp_e] = '1'	Person 6: Did this person experience any bias or discrimination because of their symptoms?	radio<br>1 yes<br>0 no<br>2 don't know
416	[hh6_covidsymp5_e]<br><br>Show the field ONLY if:<br>[language] = '1' and [hh6_covidsymp_e] = '1'	Person 6: What did this person do in response to their symptoms?<br>*Select all that apply.*	checkbox<br>0 hh6_covidsymp5_e___0 nothing<br>1 hh6_covidsymp5_e___1 took over the counter medication (ibuprofen, acetaminophen, etc.)<br>2 hh6_covidsymp5_e___2 communicated with a health care provider over the phone<br>3 hh6_covidsymp5_e___3 visited a health care provider's office<br>4 hh6_covidsymp5_e___4 visited a retail clinic or

			pharmacy<br>5 hh6_covidsymp5_e___5 visited urgent care (FASTMed, etc.)<br>6 hh6_covidsymp5_e___6 visited the emergency room<br>7 hh6_covidsymp5_e___7 was admitted to the hospital<br>8 hh6_covidsymp5_e___8 other<br>9 hh6_covidsymp5_e___9 don't know
417	[hh6_covidsymp6_e]<br>Show the field ONLY if:<br>[language] = '1' and [hh6_covidsymp5_e(8)] = '1'	Person 6: Please specify what other action this person took in response to their symptoms.	text
418	[hh6_covidsymp7_e]<br>Show the field ONLY if:<br>[language] = '1' and ([hh6_covidsymp5_e(2)] = '1' or [hh6_covidsymp5_e(3)] = '1' or [hh6_covidsymp5_e(4)] = '1' or [hh6_covidsymp5_e(5)] = '1' or [hh6_covidsymp5_e(6)] = '1' or [hh6_covidsymp5_e(7)] = '1' or [hh6_covidsymp5_e(8)] = '1')	Person 6: Did a health care provider tell this person that they may have COVID-19?	radio<br>1 yes<br>0 no<br>2 don't know
419	[hh6_covid_test_e]<br>Show the field ONLY if:<br>[language] = '1' and [hh6_covidsymp_e] = '1'	Person 6: If this person received a COVID-19 test due to their symptoms, what was the result?	radio<br>1 pending<br>2 positive<br>3 negative<br>4 inconclusive<br>5 did not receive a test<br>6 don't know
420	[hh6_covid_admit_e]<br>Show the field ONLY if:<br>[language] = '1' and [hh6_covidsymp5_e(7)] = '1'	Person 6: How many days was this person admitted to the hospital?	text (number, Min: 0)
421	[hh6_covid_admit2_e]<br>Show the field ONLY if:<br>[language] = '1' and [hh6_covidsymp5_e(7)] = '1'	Person 6: Did this person receive any of the following interventions during their hospital admission?	checkbox<br>1 hh6_covid_admit2_e___1 extra oxygen in your nose<br>2 hh6_covid_admit2_e___2 treatment in the intensive care unit (ICU)<br>3 hh6_covid_admit2_e___3 mechanical ventilation (intubation or a breathing tube)<br>4 hh6_covid_admit2_e___4 don't know

422	[hh6_covidsymp8_e]<br><br>Show the field ONLY if:<br>[language] = '1' and [hh6_covidsymp_e] = '1'	Person 6: Has this person returned to their normal health at this time?	radio<br>1 yes<br>0 no<br>2 don't know
423	[hh6_prevent_e]<br><br>Show the field ONLY if:<br>[language] = '1' and [hh6_covidsymp_e] = '1'	Person 6: Which of the following did this person do to protect their friends and family after their symptoms began?	checkbox<br>1 hh6_prevent_e___1 wore a mask more frequently<br>2 hh6_prevent_e___2 washed your hands with soap and water more frequently<br>3 hh6_prevent_e___3 used hand sanitizer more frequently<br>4 hh6_prevent_e___4 isolated yourself in your home more frequently<br>5 hh6_prevent_e___5 stayed home more frequently<br>6 hh6_prevent_e___6 wore disposable gloves more frequently<br>7 hh6_prevent_e___7 don't know
424	[hh7_relationship_e]<br><br>Show the field ONLY if:<br>[language] = '1' and [hhcount_e] > 6 and [hhcount_e] < 13	Section Header: *For each additional person in the your household, please provide the following information.*<br><br>Person 7: What is your relationship to this person?	radio<br>1 partner or spouse<br>2 child<br>3 parent<br>4 sibling<br>5 other family member<br>6 in-home childcare provider or other caregiver<br>7 other
425	[hh7_relationship2_e]<br><br>Show the field ONLY if:<br>[language] = '1' and [hh7_relationship_e] = '7'	Person 7: Please specify your relationship with this person.	text
426	[hh7_age_e]<br><br>Show the field ONLY if:<br>[language] = '1' and [hhcount_e] > 6 and [hhcount_e] < 13	Person 7: What is this person's age?<br>*Please specify their age in years*	text (number, Min: 0, Max: 110)
427	[hh7_sex_e]<br><br>Show the field ONLY if:<br>[language] = '1' and [hhcount_e] > 6 and [hhcount_e] < 13	Person 7: What is this person's sex?	radio<br>1 Female<br>2 Male<br>3 Other
428	[hh7_race_e]<br><br>Show the field ONLY if:<br>[language] = '1' and [hhcount_e] > 6 and [hhcount_e] < 13	Person 7: What is this person's race?<br>*Select all that apply.*	checkbox<br>1 hh7_race_e___1 American Indian or Alaska Native<br>2 hh7_race_e___2 Asian

			3 hh7_race_e___3 Black or African American<br>4 hh7_race_e___4 Native Hawaiian or Pacific Islander<br>5 hh7_race_e___5 White<br>6 hh7_race_e___6 Other<br>7 hh7_race_e___7 don't know
429	[hh7_ethn_e]<br><br>Show the field ONLY if:<br>[language] = '1' and [hhcount_e] > 6 and [hhcount_e] < 13	Person 7: What is this person's ethnicity?	radio<br>1 Hispanic or Latino<br>2 Not Hispanic or Latino<br>3 Other<br>4 don't know
430	[hh7_edu_e]<br><br>Show the field ONLY if:<br>[language] = '1' and [hhcount_e] > 6 and [hhcount_e] < 13	Person 7: What is the highest level of education or schooling this person has completed?	radio<br>1 never attended school<br>2 kindergarten - 8th grade<br>3 some high school<br>4 high school equivalency (GED)<br>5 high school graduate<br>6 some college<br>7 college graduate<br>8 graduate school or more<br>9 don't know
431	[hh7_work_e]<br><br>Show the field ONLY if:<br>[language] = '1' and [hhcount_e] > 6 and [hhcount_e] < 13	Person 7: Which of the following best fit this person's current work situation?	radio<br>1 works full time<br>2 works part time<br>3 is looking for work/employment<br>4 retired<br>5 homemaker<br>6 student<br>7 on maternity/paternity leave<br>8 on illness/sick leave<br>9 on disability<br>10 other<br>11 don't know
432	[hh7_work2_e]<br><br>Show the field ONLY if:<br>[language] = '1' and [hhcount_e] > 6 and [hhcount_e] < 13	Person 7: Does this person currently consider themselves self-employed (including as an independent contractor or gig-economy worker)?	radio<br>1 yes<br>0 no<br>2 don't know
433	[hh7_work3_e]<br><br>Show the field ONLY if:<br>[language] = '1' and [hhcount_e] > 6 and [hhcount_e] < 13 and ([hh7_work_e]	Person 7: Does this person currently work in any of the following high-risk settings for COVID-19 transmission?	checkbox<br>1 hh7_work3_e___1 healthcare setting (hospital, clinic, urgent care, etc.)

	= '1' or [hh7_work_e] = '2' or [hh7_work2_e] = '1')		2 hh7_work3_e___2 dense residential setting (nursing home, other long-term care facility)<br>3 hh7_work3_e___3 prison or jail<br>4 hh7_work3_e___4 meatpacking facility<br>5 hh7_work3_e___5 shipping or distribution facility<br>6 hh7_work3_e___6 high-volume retail facility (grocery store, etc.)<br>7 hh7_work3_e___7 don't know
434	[hh7_work4_e]<br><br>Show the field ONLY if: [language] = '1' and [hhcount_e] > 6 and [hhcount_e] < 13 and ([hh7_work_e] = '1' or [hh7_work_e] = '2' or [hh7_work2_e] = '1')	Person 7: Does this person's employer offer them any of the following benefits at their current main job?<br>*Select all that apply.*	checkbox<br>1 hh7_work4_e___1 paid sick leave<br>2 hh7_work4_e___2 paid vacation/personal leave<br>3 hh7_work4_e___3 health insurance<br>4 hh7_work4_e___4 disability insurance<br>5 hh7_work4_e___5 retirement plan<br>6 hh7_work4_e___6 other<br>7 hh7_work4_e___7 don't know
435	[hh7_work5_e]<br><br>Show the field ONLY if: [language] = '1' and [hhcount_e] > 6 and [hhcount_e] < 13 and ([hh7_work_e] = '1' or [hh7_work_e] = '2' or [hh7_work2_e] = '1')	Person 7: On a scale of 0 (definitely not going to happen) to 10 (definitely going to happen), how likely is it that this person will lose their job because of the COVID-19 pandemic?	text (number, Min: 0, Max: 10)
436	[hh7_work6_e]<br><br>Show the field ONLY if: [language] = '1' and [hhcount_e] > 6 and [hhcount_e] < 13 and ([hh7_work_e] = '1' or [hh7_work_e] = '2' or [hh7_work2_e] = '1')	Person 7: On a scale of 0 (definitely not going to happen) to 10 (definitely going to happen), how likely is it that this person will receive fewer work hours at their job because of the COVID-19 pandemic?	text (number, Min: 0, Max: 10)
437	[hh7_work7_e]<br><br>Show the field ONLY if: [language] = '1' and [hhcount_e] > 6 and [hhcount_e] < 13 and ([hh7_work_e] = '1' or [hh7_work_e] = '2' or [hh7_work2_e] = '1')	Person 7: How often is this person required to work from outside of the home currently?	radio (Matrix)<br>1 always (100%)<br>2 most of the time (75%)<br>3 half of the time (50%)<br>4 less than half of the time (25%)<br>5 never (0%)<br>6 don't know
438	[hh7_work8_e]<br><br>Show the field ONLY if: [language] = '1' and ([hh7_work7_e] = '1' or [hh7_work7_e] = '2' or [hh7_wor	Person 7: How regularly is this person in close physical contact with co-workers during their work outside of the home currently?	radio (Matrix)<br>1 always (100%)<br>2 most of the time (75%)<br>3 half of the time (50%)<br>4 less than half of the time (25%)

	k7_e] = '3' or [hh7_work7_e] = '4')		5 never (0%)<br>6 don't know
439	[hh7_work9_e]<br><br>Show the field ONLY if:<br>[language] = '1' and ([hh7_work7_e] = '1' or [hh7_work7_e] = '2' or [hh7_work7_e] = '3' or [hh7_work7_e] = '4')	Person 7: How regularly is this person in close physical contact with clients during their work outside of the home currently?	radio (Matrix)<br>1 always (100%)<br>2 most of the time (75%)<br>3 half of the time (50%)<br>4 less than half of the time (25%)<br>5 never (0%)<br>6 don't know
440	[hh7_covidvaccine_e]<br><br>Show the field ONLY if:<br>[language] = '1' and [hhcount_e] > 6 and [hhcount_e] < 13	Person 7: Does this person plan to get a vaccine for COVID-19 when one becomes available?	radio<br>1 yes<br>0 no<br>2 don't know
441	[hh7_covidsymp_e]<br><br>Show the field ONLY if:<br>[language] = '1' and [hhcount_e] > 6 and [hhcount_e] < 13	Person 7: Has this person had any symptoms (cough, fever, difficulty breathing, fatigue, body aches, diarrhea, runny nose, loss of smell or taste) consistent with COVID-19 in the last two weeks?	radio<br>1 yes<br>0 no<br>2 don't know
442	[hh7_covidsymp2_e]<br><br>Show the field ONLY if:<br>[language] = '1' and [hh7_covidsymp_e] = '1'	Person 7: When did this person's symptoms begin?	text (date_mdy)
443	[hh7_covidsymp3_e]<br><br>Show the field ONLY if:<br>[language] = '1' and [hh7_covidsymp_e] = '1'	Person 7: Is this person worried that they may have had COVID-19 because of their symptoms?	radio<br>1 yes<br>0 no<br>2 don't know
444	[hh7_covidsymp4_e]<br><br>Show the field ONLY if:<br>[language] = '1' and [hh7_covidsymp_e] = '1'	Person 7: Did this person experience any bias or discrimination because of their symptoms?	radio<br>1 yes<br>0 no<br>2 don't know
445	[hh7_covidsymp5_e]<br><br>Show the field ONLY if:<br>[language] = '1' and [hh7_covidsymp_e] = '1'	Person 7: What did this person do in response to their symptoms?<br>*Select all that apply.*	checkbox<br>0 hh7_covidsymp5_e___0 nothing<br>1 hh7_covidsymp5_e___1 took over the counter medication (ibuprofen, acetaminophen, etc.)<br>2 hh7_covidsymp5_e___2 communicated with a health care provider over the phone<br>3 hh7_covidsymp5_e___3 visited a health care provider's office<br>4 hh7_covidsymp5_e___4 visited a retail clinic or

			pharmacy<br>5 hh7_covidsymp5_e___5 visited urgent care (FASTMed, etc.)<br>6 hh7_covidsymp5_e___6 visited the emergency room<br>7 hh7_covidsymp5_e___7 was admitted to the hospital<br>8 hh7_covidsymp5_e___8 other<br>9 hh7_covidsymp5_e___9 don't know
446	[hh7_covidsymp6_e]<br><br>Show the field ONLY if:<br>[language] = '1' and [hh7_covidsymp5_e(8)] = '1'	Person 7: Please specify what other action this person took in response to their symptoms.	text
447	[hh7_covidsymp7_e]<br><br>Show the field ONLY if:<br>[language] = '1' and ([hh7_covidsymp5_e(2)] = '1' or [hh7_covidsymp5_e(3)] = '1' or [hh7_covidsymp5_e(4)] = '1' or [hh7_covidsymp5_e(5)] = '1' or [hh7_covidsymp5_e(6)] = '1' or [hh7_covidsymp5_e(7)] = '1' or [hh7_covidsymp5_e(8)] = '1')	Person 7: Did a health care provider tell this person that they may have COVID-19?	radio<br>1 yes<br>0 no<br>2 don't know
448	[hh7_covid_test_e]<br><br>Show the field ONLY if:<br>[language] = '1' and [hh7_covidsymp_e] = '1'	Person 7: If this person received a COVID-19 test due to their symptoms, what was the result?	radio<br>1 pending<br>2 positive<br>3 negative<br>4 inconclusive<br>5 did not receive a test<br>6 don't know
449	[hh7_covid_admit_e]<br><br>Show the field ONLY if:<br>[language] = '1' and [hh7_covidsymp5_e(7)] = '1'	Person 7: How many days was this person admitted to the hospital?	text (number, Min: 0)
450	[hh7_covid_admit2_e]<br><br>Show the field ONLY if:<br>[language] = '1' and [hh7_covidsymp5_e(7)] = '1'	Person 7: Did this person receive any of the following interventions during their hospital admission?	checkbox<br>1 hh7_covid_admit2_e___1 extra oxygen in your nose<br>2 hh7_covid_admit2_e___2 treatment in the intensive care unit (ICU)<br>3 hh7_covid_admit2_e___3 mechanical ventilation (intubation or a breathing tube)<br>4 hh7_covid_admit2_e___4 don't know

451	[hh7_covidsymp8_e]<br>Show the field ONLY if:<br>[language] = '1' and [hh7_covidsymp_e] = '1'	Person 7: Has this person returned to their normal health at this time?	radio<br>1 yes<br>0 no<br>2 don't know
452	[hh7_prevent_e]<br>Show the field ONLY if:<br>[language] = '1' and [hh7_covidsymp_e] = '1'	Person 7: Which of the following did this person do to protect their friends and family after their symptoms began?	checkbox<br>1 hh7_prevent_e___1 wore a mask more frequently<br>2 hh7_prevent_e___2 washed your hands with soap and water more frequently<br>3 hh7_prevent_e___3 used hand sanitizer more frequently<br>4 hh7_prevent_e___4 isolated yourself in your home more frequently<br>5 hh7_prevent_e___5 stayed home more frequently<br>6 hh7_prevent_e___6 wore disposable gloves more frequently<br>7 hh7_prevent_e___7 don't know
453	[hh8_relationship_e]<br>Show the field ONLY if:<br>[language] = '1' and [hhcount_e] > 7 and [hhcount_e] < 13	Section Header: *For each additional person in the your household, please provide the following information.*<br>Person 8: What is your relationship to this person?	radio<br>1 partner or spouse<br>2 child<br>3 parent<br>4 sibling<br>5 other family member<br>6 in-home childcare provider or other caregiver<br>7 other
454	[hh8_relationship2_e]<br>Show the field ONLY if:<br>[language] = '1' and [hh8_relationship_e] = '7'	Person 8: Please specify your relationship with this person.	text
455	[hh8_age_e]<br>Show the field ONLY if:<br>[language] = '1' and [hhcount_e] > 7 and [hhcount_e] < 13	Person 8: What is this person's age?<br>*Please specify their age in years*	text (number, Min: 0, Max: 110)
456	[hh8_sex_e]<br>Show the field ONLY if:<br>[language] = '1' and [hhcount_e] > 7 and [hhcount_e] < 13	Person 8: What is this person's sex?	radio<br>1 Female<br>2 Male<br>3 Other
457	[hh8_race_e]<br>Show the field ONLY if:<br>[language] = '1' and [hhcount_e] > 7 and [hhcount_e] < 13	Person 8: What is this person's race?<br>*Select all that apply.*	checkbox<br>1 hh8_race_e___1 American Indian or Alaska Native<br>2 hh8_race_e___2 Asian

			3 | hh8_race_e___3 | Black or African American<br>4 | hh8_race_e___4 | Native Hawaiian or Pacific Islander<br>5 | hh8_race_e___5 | White<br>6 | hh8_race_e___6 | Other<br>7 | hh8_race_e___7 | don't know
458	[hh8_ethn_e]<br><br>Show the field ONLY if:<br>[language] = '1' and [hhcount_e] > 7 and [hhcount_e] < 13	Person 8: What is this person's ethnicity?	radio<br>1 | Hispanic or Latino<br>2 | Not Hispanic or Latino<br>3 | Other<br>4 | don't know
459	[hh8_edu_e]<br><br>Show the field ONLY if:<br>[language] = '1' and [hhcount_e] > 7 and [hhcount_e] < 13	Person 8: What is the highest level of education or schooling this person has completed?	radio<br>1 | never attended school<br>2 | kindergarten - 8th grade<br>3 | some high school<br>4 | high school equivalency (GED)<br>5 | high school graduate<br>6 | some college<br>7 | college graduate<br>8 | graduate school or more<br>9 | don't know
460	[hh8_work_e]<br><br>Show the field ONLY if:<br>[language] = '1' and [hhcount_e] > 7 and [hhcount_e] < 13	Person 8: Which of the following best fit this person's current work situation?	radio<br>1 | works full time<br>2 | works part time<br>3 | is looking for work/employment<br>4 | retired<br>5 | homemaker<br>6 | student<br>7 | on maternity/paternity leave<br>8 | on illness/sick leave<br>9 | on disability<br>10 | other<br>11 | don't know
461	[hh8_work2_e]<br><br>Show the field ONLY if:<br>[language] = '1' and [hhcount_e] > 7 and [hhcount_e] < 13	Person 8: Does this person currently consider themselves self-employed (including as an independent contractor or gig-economy worker)?	radio<br>1 | yes<br>0 | no<br>2 | don't know
462	[hh8_work3_e]<br><br>Show the field ONLY if:<br>[language] = '1' and [hhcount_e] > 7 and [hhcount_e] < 13 and ([hh8_work_e]	Person 8: Does this person currently work in any of the following high-risk settings for COVID-19 transmission?	checkbox<br>1 | hh8_work3_e___1 | healthcare setting (hospital, clinic, urgent care, etc.)

	= '1' or [hh8_work_e] = '2' or [hh8_work2_e] = '1')		2 | hh8_work3_e___2 | dense residential setting (nursing home, other long-term care facility)<br>3 | hh8_work3_e___3 | prison or jail<br>4 | hh8_work3_e___4 | meatpacking facility<br>5 | hh8_work3_e___5 | shipping or distribution facility<br>6 | hh8_work3_e___6 | high-volume retail facility (grocery store, etc.)<br>7 | hh8_work3_e___7 | don't know
463	[hh8_work4_e]<br><br>Show the field ONLY if: [language] = '1' and [hhcount_e] > 7 and [hhcount_e] < 13 and ([hh8_work_e] = '1' or [hh8_work_e] = '2' or [hh8_work2_e] = '1')	Person 8: Does this person's employer offer them any of the following benefits at their current main job?<br>*Select all that apply.*	checkbox<br>1 | hh8_work4_e___1 | paid sick leave<br>2 | hh8_work4_e___2 | paid vacation/personal leave<br>3 | hh8_work4_e___3 | health insurance<br>4 | hh8_work4_e___4 | disability insurance<br>5 | hh8_work4_e___5 | retirement plan<br>6 | hh8_work4_e___6 | other<br>7 | hh8_work4_e___7 | don't know
464	[hh8_work5_e]<br><br>Show the field ONLY if: [language] = '1' and [hhcount_e] > 7 and [hhcount_e] < 13 and ([hh8_work_e] = '1' or [hh8_work_e] = '2' or [hh8_work2_e] = '1')	Person 8: On a scale of 0 (definitely not going to happen) to 10 (definitely going to happen), how likely is it that this person will lose their job because of the COVID-19 pandemic?	text (number, Min: 0, Max: 10)
465	[hh8_work6_e]<br><br>Show the field ONLY if: [language] = '1' and [hhcount_e] > 7 and [hhcount_e] < 13 and ([hh8_work_e] = '1' or [hh8_work_e] = '2' or [hh8_work2_e] = '1')	Person 8: On a scale of 0 (definitely not going to happen) to 10 (definitely going to happen), how likely is it that this person will receive fewer work hours at their job because of the COVID-19 pandemic?	text (number, Min: 0, Max: 10)
466	[hh8_work7_e]<br><br>Show the field ONLY if: [language] = '1' and [hhcount_e] > 7 and [hhcount_e] < 13 and ([hh8_work_e] = '1' or [hh8_work_e] = '2' or [hh8_work2_e] = '1')	Person 8: How often is this person required to work from outside of the home currently?	radio (Matrix)<br>1 | always (100%)<br>2 | most of the time (75%)<br>3 | half of the time (50%)<br>4 | less than half of the time (25%)<br>5 | never (0%)<br>6 | don't know
467	[hh8_work8_e]<br><br>Show the field ONLY if: [language] = '1' and ([hh8_work7_e] = '1' or [hh8_work7_e] = '2' or [hh8_wor	Person 8: How regularly is this person in close physical contact with co-workers during their work outside of the home currently?	radio (Matrix)<br>1 | always (100%)<br>2 | most of the time (75%)<br>3 | half of the time (50%)<br>4 | less than half of the time (25%)

	k7_e] = '3' or [hh8_work7_e] = '4')		5 never (0%)<br>6 don't know
468	[hh8_work9_e]<br><br>Show the field ONLY if: [language] = '1' and ([hh8_work7_e] = '1' or [hh8_work7_e] = '2' or [hh8_work7_e] = '3' or [hh8_work7_e] = '4')	Person 8: How regularly is this person in close physical contact with clients during their work outside of the home currently?	radio (Matrix)<br>1 always (100%)<br>2 most of the time (75%)<br>3 half of the time (50%)<br>4 less than half of the time (25%)<br>5 never (0%)<br>6 don't know
469	[hh8_covidvaccine_e]<br><br>Show the field ONLY if: [language] = '1' and [hhcount_e] > 7 and [hhcount_e] < 13	Person 8: Does this person plan to get a vaccine for COVID-19 when one becomes available?	radio<br>1 yes<br>0 no<br>2 don't know
470	[hh8_covidsymp_e]<br><br>Show the field ONLY if: [language] = '1' and [hhcount_e] > 7 and [hhcount_e] < 13	Person 8: Has this person had any symptoms (cough, fever, difficulty breathing, fatigue, body aches, diarrhea, runny nose, loss of smell or taste) consistent with COVID-19 in the last two weeks?	radio<br>1 yes<br>0 no<br>2 don't know
471	[hh8_covidsymp2_e]<br><br>Show the field ONLY if: [language] = '1' and [hh8_covidsymp_e] = '1'	Person 8: When did this person's symptoms begin?	text (date_mdy)
472	[hh8_covidsymp3_e]<br><br>Show the field ONLY if: [language] = '1' and [hh8_covidsymp_e] = '1'	Person 8: Is this person worried that they may have had COVID-19 because of their symptoms?	radio<br>1 yes<br>0 no<br>2 don't know
473	[hh8_covidsymp4_e]<br><br>Show the field ONLY if: [language] = '1' and [hh8_covidsymp_e] = '1'	Person 8: Did this person experience any bias or discrimination because of their symptoms?	radio<br>1 yes<br>0 no<br>2 don't know
474	[hh8_covidsymp5_e]<br><br>Show the field ONLY if: [language] = '1' and [hh8_covidsymp_e] = '1'	Person 8: What did this person do in response to their symptoms?<br>*Select all that apply.*	checkbox<br>0 hh8_covidsymp5_e___0 nothing<br>1 hh8_covidsymp5_e___1 took over the counter medication (ibuprofen, acetaminophen, etc.)<br>2 hh8_covidsymp5_e___2 communicated with a health care provider over the phone<br>3 hh8_covidsymp5_e___3 visited a health care provider's office<br>4 hh8_covidsymp5_e___4 visited a retail clinic or

			pharmacy<br>5 hh8_covidsymp5_e___5 visited urgent care (FASTMed, etc.)<br>6 hh8_covidsymp5_e___6 visited the emergency room<br>7 hh8_covidsymp5_e___7 was admitted to the hospital<br>8 hh8_covidsymp5_e___8 other<br>9 hh8_covidsymp5_e___9 don't know
475	[hh8_covidsymp6_e]<br>Show the field ONLY if: [language] = '1' and [hh8_covidsymp5_e(8)] = '1'	Person 8: Please specify what other action this person took in response to their symptoms.	text
476	[hh8_covidsymp7_e]<br>Show the field ONLY if: [language] = '1' and ([hh8_covidsymp5_e(2)] = '1' or [hh8_covidsymp5_e(3)] = '1' or [hh8_covidsymp5_e(4)] = '1' or [hh8_covidsymp5_e(5)] = '1' or [hh8_covidsymp5_e(6)] = '1' or [hh8_covidsymp5_e(7)] = '1' or [hh8_covidsymp5_e(8)] = '1')	Person 8: Did a health care provider tell this person that they may have COVID-19?	radio<br>1 yes<br>0 no<br>2 don't know
477	[hh8_covid_test_e]<br>Show the field ONLY if: [language] = '1' and [hh8_covidsymp_e] = '1'	Person 8: If this person received a COVID-19 test due to their symptoms, what was the result?	radio<br>1 pending<br>2 positive<br>3 negative<br>4 inconclusive<br>5 did not receive a test<br>6 don't know
478	[hh8_covid_admit_e]<br>Show the field ONLY if: [language] = '1' and [hh8_covidsymp5_e(7)] = '1'	Person 8: How many days was this person admitted to the hospital?	text (number, Min: 0)
479	[hh8_covid_admit2_e]<br>Show the field ONLY if: [language] = '1' and [hh8_covidsymp5_e(7)] = '1'	Person 8: Did this person receive any of the following interventions during their hospital admission?	checkbox<br>1 hh8_covid_admit2_e___1 extra oxygen in your nose<br>2 hh8_covid_admit2_e___2 treatment in the intensive care unit (ICU)<br>3 hh8_covid_admit2_e___3 mechanical ventilation (intubation or a breathing tube)<br>4 hh8_covid_admit2_e___4 don't know

480	[hh8_covidsymp8_e]<br>Show the field ONLY if:<br>[language] = '1' and [hh8_covidsymp_e] = '1'	Person 8: Has this person returned to their normal health at this time?	radio<br>1 yes<br>0 no<br>2 don't know
481	[hh8_prevent_e]<br>Show the field ONLY if:<br>[language] = '1' and [hh8_covidsymp_e] = '1'	Person 8: Which of the following did this person do to protect their friends and family after their symptoms began?	checkbox<br>1 hh8_prevent_e___1 wore a mask more frequently<br>2 hh8_prevent_e___2 washed your hands with soap and water more frequently<br>3 hh8_prevent_e___3 used hand sanitizer more frequently<br>4 hh8_prevent_e___4 isolated yourself in your home more frequently<br>5 hh8_prevent_e___5 stayed home more frequently<br>6 hh8_prevent_e___6 wore disposable gloves more frequently<br>7 hh8_prevent_e___7 don't know
482	[hh9_relationship_e]<br>Show the field ONLY if:<br>[language] = '1' and [hhcount_e] > 8 and [hhcount_e] < 13	Section Header: *For each additional person in the your household, please provide the following information.*<br>Person 9: What is your relationship to this person?	radio<br>1 partner or spouse<br>2 child<br>3 parent<br>4 sibling<br>5 other family member<br>6 in-home childcare provider or other caregiver<br>7 other
483	[hh9_relationship2_e]<br>Show the field ONLY if:<br>[language] = '1' and [hh9_relationship_e] = '7'	Person 9: Please specify your relationship with this person.	text
484	[hh9_age_e]<br>Show the field ONLY if:<br>[language] = '1' and [hhcount_e] > 8 and [hhcount_e] < 13	Person 9: What is this person's age?<br>*Please specify their age in years*	text (number, Min: 0, Max: 110)
485	[hh9_sex_e]<br>Show the field ONLY if:<br>[language] = '1' and [hhcount_e] > 8 and [hhcount_e] < 13	Person 9: What is this person's sex?	radio<br>1 Female<br>2 Male<br>3 Other
486	[hh9_race_e]<br>Show the field ONLY if:<br>[language] = '1' and [hhcount_e] > 8 and [hhcount_e] < 13	Person 9: What is this person's race?<br>*Select all that apply.*	checkbox<br>1 hh9_race_e___1 American Indian or Alaska Native<br>2 hh9_race_e___2 Asian

			3 hh9_race_e___3 Black or African American<br>4 hh9_race_e___4 Native Hawaiian or Pacific Islander<br>5 hh9_race_e___5 White<br>6 hh9_race_e___6 Other<br>7 hh9_race_e___7 don't know
487	[hh9_ethn_e]<br><br>Show the field ONLY if:<br>[language] = '1' and [hhcount_e] > 8 and [hhcount_e] < 13	Person 9: What is this person's ethnicity?	radio<br>1 Hispanic or Latino<br>2 Not Hispanic or Latino<br>3 Other<br>4 don't know
488	[hh9_edu_e]<br><br>Show the field ONLY if:<br>[language] = '1' and [hhcount_e] > 8 and [hhcount_e] < 13	Person 9: What is the highest level of education or schooling this person has completed?	radio<br>1 never attended school<br>2 kindergarten - 8th grade<br>3 some high school<br>4 high school equivalency (GED)<br>5 high school graduate<br>6 some college<br>7 college graduate<br>8 graduate school or more<br>9 don't know
489	[hh9_work_e]<br><br>Show the field ONLY if:<br>[language] = '1' and [hhcount_e] > 8 and [hhcount_e] < 13	Person 9: Which of the following best fit this person's current work situation?	radio<br>1 works full time<br>2 works part time<br>3 is looking for work/employment<br>4 retired<br>5 homemaker<br>6 student<br>7 on maternity/paternity leave<br>8 on illness/sick leave<br>9 on disability<br>10 other<br>11 don't know
490	[hh9_work2_e]<br><br>Show the field ONLY if:<br>[language] = '1' and [hhcount_e] > 8 and [hhcount_e] < 13	Person 9: Does this person currently consider themselves self-employed (including as an independent contractor or gig-economy worker)?	radio<br>1 yes<br>0 no<br>2 don't know
491	[hh9_work3_e]<br><br>Show the field ONLY if:<br>[language] = '1' and [hhcount_e] > 8 and [hhcount_e] < 13 and ([hh9_work_e]	Person 9: Does this person currently work in any of the following high-risk settings for COVID-19 transmission?	checkbox<br>1 hh9_work3_e___1 healthcare setting (hospital, clinic, urgent care, etc.)

	= '1' or [hh9_work_e] = '2' or [hh9_work2_e] = '1')		2 hh9_work3_e___2 dense residential setting (nursing home, other long-term care facility)<br>3 hh9_work3_e___3 prison or jail<br>4 hh9_work3_e___4 meatpacking facility<br>5 hh9_work3_e___5 shipping or distribution facility<br>6 hh9_work3_e___6 high-volume retail facility (grocery store, etc.)<br>7 hh9_work3_e___7 don't know
492	[hh9_work4_e]<br><br>Show the field ONLY if: [language] = '1' and [hhcount_e] > 8 and [hhcount_e] < 13 and ([hh9_work_e] = '1' or [hh9_work_e] = '2' or [hh9_work2_e] = '1')	Person 9: Does this person's employer offer them any of the following benefits at their current main job?<br>*Select all that apply.*	checkbox<br>1 hh9_work4_e___1 paid sick leave<br>2 hh9_work4_e___2 paid vacation/personal leave<br>3 hh9_work4_e___3 health insurance<br>4 hh9_work4_e___4 disability insurance<br>5 hh9_work4_e___5 retirement plan<br>6 hh9_work4_e___6 other<br>7 hh9_work4_e___7 don't know
493	[hh9_work5_e]<br><br>Show the field ONLY if: [language] = '1' and [hhcount_e] > 8 and [hhcount_e] < 13 and ([hh9_work_e] = '1' or [hh9_work_e] = '2' or [hh9_work2_e] = '1')	Person 9: On a scale of 0 (definitely not going to happen) to 10 (definitely going to happen), how likely is it that this person will lose their job because of the COVID-19 pandemic?	text (number, Min: 0, Max: 10)
494	[hh9_work6_e]<br><br>Show the field ONLY if: [language] = '1' and [hhcount_e] > 8 and [hhcount_e] < 13 and ([hh9_work_e] = '1' or [hh9_work_e] = '2' or [hh9_work2_e] = '1')	Person 9: On a scale of 0 (definitely not going to happen) to 10 (definitely going to happen), how likely is it that this person will receive fewer work hours at their job because of the COVID-19 pandemic?	text (number, Min: 0, Max: 10)
495	[hh9_work7_e]<br><br>Show the field ONLY if: [language] = '1' and [hhcount_e] > 8 and [hhcount_e] < 13 and ([hh9_work_e] = '1' or [hh9_work_e] = '2' or [hh9_work2_e] = '1')	Person 9: How often is this person required to work from outside of the home currently?	radio (Matrix)<br>1 always (100%)<br>2 most of the time (75%)<br>3 half of the time (50%)<br>4 less than half of the time (25%)<br>5 never (0%)<br>6 don't know
496	[hh9_work8_e]<br><br>Show the field ONLY if: [language] = '1' and ([hh9_work7_e] = '1' or [hh9_work7_e] = '2' or [hh9_wor	Person 9: How regularly is this person in close physical contact with co-workers during their work outside of the home currently?	radio (Matrix)<br>1 always (100%)<br>2 most of the time (75%)<br>3 half of the time (50%)<br>4 less than half of the time (25%)

	k7_e] = '3' or [hh9_work7_e] = '4')		5 never (0%)<br>6 don't know
497	[hh9_work9_e]<br><br>Show the field ONLY if:<br>[language] = '1' and ([hh9_work7_e] = '1' or [hh9_work7_e] = '2' or [hh9_work7_e] = '3' or [hh9_work7_e] = '4')	Person 9: How regularly is this person in close physical contact with clients during their work outside of the home currently?	radio (Matrix)<br>1 always (100%)<br>2 most of the time (75%)<br>3 half of the time (50%)<br>4 less than half of the time (25%)<br>5 never (0%)<br>6 don't know
498	[hh9_covidvaccine_e]<br><br>Show the field ONLY if:<br>[language] = '1' and [hhcount_e] > 8 and [hhcount_e] < 13	Person 9: Does this person plan to get a vaccine for COVID-19 when one becomes available?	radio<br>1 yes<br>0 no<br>2 don't know
499	[hh9_covidsymp_e]<br><br>Show the field ONLY if:<br>[language] = '1' and [hhcount_e] > 8 and [hhcount_e] < 13	Person 9: Has this person had any symptoms (cough, fever, difficulty breathing, fatigue, body aches, diarrhea, runny nose, loss of smell or taste) consistent with COVID-19 in the last two weeks?	radio<br>1 yes<br>0 no<br>2 don't know
500	[hh9_covidsymp2_e]<br><br>Show the field ONLY if:<br>[language] = '1' and [hh9_covidsymp_e] = '1'	Person 9: When did this person's symptoms begin?	text (date_mdy)
501	[hh9_covidsymp3_e]<br><br>Show the field ONLY if:<br>[language] = '1' and [hh9_covidsymp_e] = '1'	Person 9: Is this person worried that they may have had COVID-19 because of their symptoms?	radio<br>1 yes<br>0 no<br>2 don't know
502	[hh9_covidsymp4_e]<br><br>Show the field ONLY if:<br>[language] = '1' and [hh9_covidsymp_e] = '1'	Person 9: Did this person experience any bias or discrimination because of their symptoms?	radio<br>1 yes<br>0 no<br>2 don't know
503	[hh9_covidsymp5_e]<br><br>Show the field ONLY if:<br>[language] = '1' and [hh9_covidsymp_e] = '1'	Person 9: What did this person do in response to their symptoms?<br>*Select all that apply.*	checkbox<br>0 hh9_covidsymp5_e___0 nothing<br>1 hh9_covidsymp5_e___1 took over the counter medication (ibuprofen, acetaminophen, etc.)<br>2 hh9_covidsymp5_e___2 communicated with a health care provider over the phone<br>3 hh9_covidsymp5_e___3 visited a health care provider's office<br>4 hh9_covidsymp5_e___4 visited a retail clinic or

			pharmacy<br>5 hh9_covidsymp5_e___5 visited urgent care (FASTMed, etc.)<br>6 hh9_covidsymp5_e___6 visited the emergency room<br>7 hh9_covidsymp5_e___7 was admitted to the hospital<br>8 hh9_covidsymp5_e___8 other<br>9 hh9_covidsymp5_e___9 don't know
504	[hh9_covidsymp6_e]<br><br>Show the field ONLY if:<br>[language] = '1' and [hh9_covidsymp5_e(8)] = '1'	Person 9: Please specify what other action this person took in response to their symptoms.	text
505	[hh9_covidsymp7_e]<br><br>Show the field ONLY if:<br>[language] = '1' and ([hh9_covidsymp5_e(2)] = '1' or [hh9_covidsymp5_e(3)] = '1' or [hh9_covidsymp5_e(4)] = '1' or [hh9_covidsymp5_e(5)] = '1' or [hh9_covidsymp5_e(6)] = '1' or [hh9_covidsymp5_e(7)] = '1' or [hh9_covidsymp5_e(8)] = '1')	Person 9: Did a health care provider tell this person that they may have COVID-19?	radio<br>1 yes<br>0 no<br>2 don't know
506	[hh9_covid_test_e]<br><br>Show the field ONLY if:<br>[language] = '1' and [hh9_covidsymp_e] = '1'	Person 9: If this person received a COVID-19 test due to their symptoms, what was the result?	radio<br>1 pending<br>2 positive<br>3 negative<br>4 inconclusive<br>5 did not receive a test<br>6 don't know
507	[hh9_covid_admit_e]<br><br>Show the field ONLY if:<br>[language] = '1' and [hh9_covidsymp5_e(7)] = '1'	Person 9: How many days was this person admitted to the hospital?	text (number, Min: 0)
508	[hh9_covid_admit2_e]<br><br>Show the field ONLY if:<br>[language] = '1' and [hh9_covidsymp5_e(7)] = '1'	Person 9: Did this person receive any of the following interventions during their hospital admission?	checkbox<br>1 hh9_covid_admit2_e___1 extra oxygen in your nose<br>2 hh9_covid_admit2_e___2 treatment in the intensive care unit (ICU)<br>3 hh9_covid_admit2_e___3 mechanical ventilation (intubation or a breathing tube)<br>4 hh9_covid_admit2_e___4 don't know

509	[hh9_covidsymp8_e]<br><br>Show the field ONLY if:<br>[language] = '1' and [hh9_covidsymp_e] = '1'	Person 9: Has this person returned to their normal health at this time?	radio<br>1 yes<br>0 no<br>2 don't know
510	[hh9_prevent_e]<br><br>Show the field ONLY if:<br>[language] = '1' and [hh9_covidsymp_e] = '1'	Person 9: Which of the following did this person do to protect their friends and family after their symptoms began?	checkbox<br>1 hh9_prevent_e___1 wore a mask more frequently<br>2 hh9_prevent_e___2 washed your hands with soap and water more frequently<br>3 hh9_prevent_e___3 used hand sanitizer more frequently<br>4 hh9_prevent_e___4 isolated yourself in your home more frequently<br>5 hh9_prevent_e___5 stayed home more frequently<br>6 hh9_prevent_e___6 wore disposable gloves more frequently<br>7 hh9_prevent_e___7 don't know
511	[hh10_relationship_e]<br><br>Show the field ONLY if:<br>[language] = '1' and [hhcount_e] > 9 and [hhcount_e] < 13	Section Header: *For each additional person in the your household, please provide the following information.*<br><br>Person 10: What is your relationship to this person?	radio<br>1 partner or spouse<br>2 child<br>3 parent<br>4 sibling<br>5 other family member<br>6 in-home childcare provider or other caregiver<br>7 other
512	[hh10_relationship2_e]<br><br>Show the field ONLY if:<br>[language] = '1' and [hh10_relationship_e] = '7'	Person 10: Please specify your relationship with this person.	text
513	[hh10_age_e]<br><br>Show the field ONLY if:<br>[language] = '1' and [hhcount_e] > 9 and [hhcount_e] < 13	Person 10: What is this person's age?<br>*Please specify their age in years*	text (number, Min: 0, Max: 110)
514	[hh10_sex_e]<br><br>Show the field ONLY if:<br>[language] = '1' and [hhcount_e] > 9 and [hhcount_e] < 13	Person 10: What is this person's sex?	radio<br>1 Female<br>2 Male<br>3 Other
515	[hh10_race_e]<br><br>Show the field ONLY if:<br>[language] = '1' and [hhcount_e] > 9 and [hhcount_	Person 10: What is this person's race?<br>*Select all that apply.*	checkbox<br>1 hh10_race_e___1 American Indian or Alaska Native

	e] < 13		2 hh10_race_e___2 Asian<br>3 hh10_race_e___3 Black or African American<br>4 hh10_race_e___4 Native Hawaiian or Pacific Islander<br>5 hh10_race_e___5 White<br>6 hh10_race_e___6 Other<br>7 hh10_race_e___7 don't know
516	[hh10_ethn_e]<br>Show the field ONLY if:<br>[language] = '1' and [hhcount_e] > 9 and [hhcount_e] < 13	Person 10: What is this person's ethnicity?	radio<br>1 Hispanic or Latino<br>2 Not Hispanic or Latino<br>3 Other<br>4 don't know
517	[hh10_edu_e]<br>Show the field ONLY if:<br>[language] = '1' and [hhcount_e] > 9 and [hhcount_e] < 13	Person 10: What is the highest level of education or schooling this person has completed?	radio<br>1 never attended school<br>2 kindergarten - 8th grade<br>3 some high school<br>4 high school equivalency (GED)<br>5 high school graduate<br>6 some college<br>7 college graduate<br>8 graduate school or more<br>9 don't know
518	[hh10_work_e]<br>Show the field ONLY if:<br>[language] = '1' and [hhcount_e] > 9 and [hhcount_e] < 13	Person 10: Which of the following best fit this person's current work situation?	radio<br>1 works full time<br>2 works part time<br>3 is looking for work/employment<br>4 retired<br>5 homemaker<br>6 student<br>7 on maternity/paternity leave<br>8 on illness/sick leave<br>9 on disability<br>10 other<br>11 don't know
519	[hh10_work2_e]<br>Show the field ONLY if:<br>[language] = '1' and [hhcount_e] > 9 and [hhcount_e] < 13	Person 10: Does this person currently consider themselves self-employed (including as an independent contractor or gig-economy worker)?	radio<br>1 yes<br>0 no<br>2 don't know
520	[hh10_work3_e]<br>Show the field ONLY if:<br>[language] = '1' and [hhcount_e] > 9 and [hhcount_	Person 10: Does this person currently work in any of the following high-risk settings for COVID-19 transmission?	checkbox<br>1 hh10_work3_e___1 healthcare setting (hospital, clinic, urgent care, etc.)

	e] < 13 and ([hh10_work_e] = '1' or [hh10_work_e] = '2' or [hh10_work2_e] = '1')		2 hh10_work3_e___2 dense residential setting (nursing home, other long-term care facility)<br>3 hh10_work3_e___3 prison or jail<br>4 hh10_work3_e___4 meatpacking facility<br>5 hh10_work3_e___5 shipping or distribution facility<br>6 hh10_work3_e___6 high-volume retail facility (grocery store, etc.)<br>7 hh10_work3_e___7 don't know
521	[hh10_work4_e]<br>Show the field ONLY if: [language] = '1' and [hhcount_e] > 9 and [hhcount_e] < 13 and ([hh10_work_e] = '1' or [hh10_work_e] = '2' or [hh10_work2_e] = '1')	Person 10: Does this person's employer offer them any of the following benefits at their current main job? *Select all that apply.*	checkbox<br>1 hh10_work4_e___1 paid sick leave<br>2 hh10_work4_e___2 paid vacation/personal leave<br>3 hh10_work4_e___3 health insurance<br>4 hh10_work4_e___4 disability insurance<br>5 hh10_work4_e___5 retirement plan<br>6 hh10_work4_e___6 other<br>7 hh10_work4_e___7 don't know
522	[hh10_work5_e]<br>Show the field ONLY if: [language] = '1' and [hhcount_e] > 9 and [hhcount_e] < 13 and ([hh10_work_e] = '1' or [hh10_work_e] = '2' or [hh10_work2_e] = '1')	Person 10: On a scale of 0 (definitely not going to happen) to 10 (definitely going to happen), how likely is it that this person will lose their job because of the COVID-19 pandemic?	text (number, Min: 0, Max: 10)
523	[hh10_work6_e]<br>Show the field ONLY if: [language] = '1' and [hhcount_e] > 9 and [hhcount_e] < 13 and ([hh10_work_e] = '1' or [hh10_work_e] = '2' or [hh10_work2_e] = '1')	Person 10: On a scale of 0 (definitely not going to happen) to 10 (definitely going to happen), how likely is it that this person will receive fewer work hours at their job because of the COVID-19 pandemic?	text (number, Min: 0, Max: 10)
524	[hh10_work7_e]<br>Show the field ONLY if: [language] = '1' and [hhcount_e] > 9 and [hhcount_e] < 13 and ([hh10_work_e] = '1' or [hh10_work_e] = '2' or [hh10_work2_e] = '1')	Person 10: How often is this person required to work from outside of the home currently?	radio (Matrix)<br>1 always (100%)<br>2 most of the time (75%)<br>3 half of the time (50%)<br>4 less than half of the time (25%)<br>5 never (0%)<br>6 don't know
525	[hh10_work8_e]<br>Show the field ONLY if: [language] = '1' and ([hh10_work7_e] = '1' or [hh10	Person 10: How regularly is this person in close physical contact with co-workers during their work outside of the home currently?	radio (Matrix)<br>1 always (100%)<br>2 most of the time (75%)

	_work7_e] = '2' or [hh10_work7_e] = '3' or [hh10_work7_e] = '4')		3 half of the time (50%)<br>4 less than half of the time (25%)<br>5 never (0%)<br>6 don't know
526	[hh10_work9_e]<br><br>Show the field ONLY if:<br>[language] = '1' and ([hh10_work7_e] = '1' or [hh10_work7_e] = '2' or [hh10_work7_e] = '3' or [hh10_work7_e] = '4')	Person 10: How regularly is this person in close physical contact with clients during their work outside of the home currently?	radio (Matrix)<br>1 always (100%)<br>2 most of the time (75%)<br>3 half of the time (50%)<br>4 less than half of the time (25%)<br>5 never (0%)<br>6 don't know
527	[hh10_covidvaccine_e]<br><br>Show the field ONLY if:<br>[language] = '1' and [hhcount_e] > 9 and [hhcount_e] < 13	Person 10: Does this person plan to get a vaccine for COVID-19 when one becomes available?	radio<br>1 yes<br>0 no<br>2 don't know
528	[hh10_covidsymp_e]<br><br>Show the field ONLY if:<br>[language] = '1' and [hhcount_e] > 9 and [hhcount_e] < 13	Person 10: Has this person had any symptoms (cough, fever, difficulty breathing, fatigue, body aches, diarrhea, runny nose, loss of smell or taste) consistent with COVID-19 in the last two weeks?	radio<br>1 yes<br>0 no<br>2 don't know
529	[hh10_covidsymp2_e]<br><br>Show the field ONLY if:<br>[language] = '1' and [hh10_covidsymp_e] = '1'	Person 10: When did this person's symptoms begin?	text (date_mdy)
530	[hh10_covidsymp3_e]<br><br>Show the field ONLY if:<br>[language] = '1' and [hh10_covidsymp_e] = '1'	Person 10: Is this person worried that they may have had COVID-19 because of their symptoms?	radio<br>1 yes<br>0 no<br>2 don't know
531	[hh10_covidsymp4_e]<br><br>Show the field ONLY if:<br>[language] = '1' and [hh10_covidsymp_e] = '1'	Person 10: Did this person experience any bias or discrimination because of their symptoms?	radio<br>1 yes<br>0 no<br>2 don't know
532	[hh10_covidsymp5_e]<br><br>Show the field ONLY if:<br>[language] = '1' and [hh10_covidsymp_e] = '1'	Person 10: What did this person do in response to their symptoms?<br>*Select all that apply.*	checkbox<br>0 hh10_covidsymp5_e___0 nothing<br>1 hh10_covidsymp5_e___1 took over the counter medication (ibuprofen, acetaminophen, etc.)<br>2 hh10_covidsymp5_e___2 communicated with a health care provider over the phone<br>3 hh10_covidsymp5_e___3 visited a health care provider's office

			4 hh10_covidsymp5_e___4 visited a retail clinic or pharmacy<br>5 hh10_covidsymp5_e___5 visited urgent care (FASTMed, etc.)<br>6 hh10_covidsymp5_e___6 visited the emergency room<br>7 hh10_covidsymp5_e___7 was admitted to the hospital<br>8 hh10_covidsymp5_e___8 other<br>9 hh10_covidsymp5_e___9 don't know
533	[hh10_covidsymp6_e]<br><br>Show the field ONLY if: [language] = '1' and [hh10_covidsymp5_e(8)] = '1'	Person 10: Please specify what other action this person took in response to their symptoms.	text
534	[hh10_covidsymp7_e]<br><br>Show the field ONLY if: [language] = '1' and ([hh10_covidsymp5_e(2)] = '1' or [hh10_covidsymp5_e(3)] = '1' or [hh10_covidsymp5_e(4)] = '1' or [hh10_covidsymp5_e(5)] = '1' or [hh10_covidsymp5_e(6)] = '1' or [hh10_covidsymp5_e(7)] = '1' or [hh10_covidsymp5_e(8)] = '1')	Person 10: Did a health care provider tell this person that they may have COVID-19?	radio<br>1 yes<br>0 no<br>2 don't know
535	[hh10_covid_test_e]<br><br>Show the field ONLY if: [language] = '1' and [hh10_covidsymp_e] = '1'	Person 10: If this person received a COVID-19 test due to their symptoms, what was the result?	radio<br>1 pending<br>2 positive<br>3 negative<br>4 inconclusive<br>5 did not receive a test<br>6 don't know
536	[hh10_covid_admit_e]<br><br>Show the field ONLY if: [language] = '1' and [hh10_covidsymp5_e(7)] = '1'	Person 10: How many days was this person admitted to the hospital?	text (number, Min: 0)
537	[hh10_covid_admit2_e]<br><br>Show the field ONLY if: [language] = '1' and [hh10_covidsymp5_e(7)] = '1'	Person 10: Did this person receive any of the following interventions during their hospital admission?	checkbox<br>1 hh10_covid_admit2_e___1 extra oxygen in your nose<br>2 hh10_covid_admit2_e___2 treatment in the intensive care unit (ICU)<br>3 hh10_covid_admit2_e___3 mechanical ventilation (intubation or

			a breathing tube)<br>4 | hh10_covid_admit2_e___4 | don't know
538	[hh10_covidsymp8_e]<br>Show the field ONLY if: [language] = '1' and [hh10_covidsymp_e] = '1'	Person 10: Has this person returned to their normal health at this time?	radio<br>1 yes<br>0 no<br>2 don't know
539	[hh10_prevent_e]<br>Show the field ONLY if: [language] = '1' and [hh10_covidsymp_e] = '1'	Person 10: Which of the following did this person do to protect their friends and family after their symptoms began?	checkbox<br>1 hh10_prevent_e___1 wore a mask more frequently<br>2 hh10_prevent_e___2 washed your hands with soap and water more frequently<br>3 hh10_prevent_e___3 used hand sanitizer more frequently<br>4 hh10_prevent_e___4 isolated yourself in your home more frequently<br>5 hh10_prevent_e___5 stayed home more frequently<br>6 hh10_prevent_e___6 wore disposable gloves more frequently<br>7 hh10_prevent_e___7 don't know
540	[hh11_relationship_e]<br>Show the field ONLY if: [language] = '1' and [hhcount_e] > 10 and [hhcount_e] < 13	Section Header: *For each additional person in the your household, please provide the following information.*<br>Person 11: What is your relationship to this person?	radio<br>1 partner or spouse<br>2 child<br>3 parent<br>4 sibling<br>5 other family member<br>6 in-home childcare provider or other caregiver<br>7 other
541	[hh11_relationship2_e]<br>Show the field ONLY if: [language] = '1' and [hh11_relationship_e] = '7'	Person 11: Please specify your relationship with this person.	text
542	[hh11_age_e]<br>Show the field ONLY if: [language] = '1' and [hhcount_e] > 10 and [hhcount_e] < 13	Person 11: What is this person's age?<br>*Please specify their age in years*	text (number, Min: 0, Max: 110)
543	[hh11_sex_e]<br>Show the field ONLY if: [language] = '1' and [hhcount_e] > 10 and [hhcount	Person 11: What is this person's sex?	radio<br>1 Female<br>2 Male

	_e] < 13		3 Other
544	[hh11_race_e]<br><br>Show the field ONLY if:<br>[language] = '1' and [hhcount_e] > 10 and [hhcount_e] < 13	Person 11: What is this person's race?<br>*Select all that apply.*	checkbox<br>1 hh11_race_e___1 American Indian or Alaska Native<br>2 hh11_race_e___2 Asian<br>3 hh11_race_e___3 Black or African American<br>4 hh11_race_e___4 Native Hawaiian or Pacific Islander<br>5 hh11_race_e___5 White<br>6 hh11_race_e___6 Other<br>7 hh11_race_e___7 don't know
545	[hh11_ethn_e]<br><br>Show the field ONLY if:<br>[language] = '1' and [hhcount_e] > 10 and [hhcount_e] < 13	Person 11: What is this person's ethnicity?	radio<br>1 Hispanic or Latino<br>2 Not Hispanic or Latino<br>3 Other<br>4 don't know
546	[hh11_edu_e]<br><br>Show the field ONLY if:<br>[language] = '1' and [hhcount_e] > 10 and [hhcount_e] < 13	Person 11: What is the highest level of education or schooling this person has completed?	radio<br>1 never attended school<br>2 kindergarten - 8th grade<br>3 some high school<br>4 high school equivalency (GED)<br>5 high school graduate<br>6 some college<br>7 college graduate<br>8 graduate school or more<br>9 don't know
547	[hh11_work_e]<br><br>Show the field ONLY if:<br>[language] = '1' and [hhcount_e] > 10 and [hhcount_e] < 13	Person 11: Which of the following best fit this person's current work situation?	radio<br>1 works full time<br>2 works part time<br>3 is looking for work/employment<br>4 retired<br>5 homemaker<br>6 student<br>7 on maternity/paternity leave<br>8 on illness/sick leave<br>9 on disability<br>10 other<br>11 don't know

548	[hh11_work2_e]<br><br>Show the field ONLY if:<br>[language] = '1' and [hhcount_e] > 10 and [hhcount_e] < 13	Person 11: Does this person currently consider themselves self-employed (including as an independent contractor or gig-economy worker)?	radio<br>1 yes<br>0 no<br>2 don't know
549	[hh11_work3_e]<br><br>Show the field ONLY if:<br>[language] = '1' and [hhcount_e] > 10 and [hhcount_e] < 13 and ([hh11_work_e] = '1' or [hh11_work_e] = '2' or [hh11_work2_e] = '1')	Person 11: Does this person currently work in any of the following high-risk settings for COVID-19 transmission?	checkbox<br>1 hh11_work3_e___1 healthcare setting (hospital, clinic, urgent care, etc.)<br>2 hh11_work3_e___2 dense residential setting (nursing home, other long-term care facility)<br>3 hh11_work3_e___3 prison or jail<br>4 hh11_work3_e___4 meatpacking facility<br>5 hh11_work3_e___5 shipping or distribution facility<br>6 hh11_work3_e___6 high-volume retail facility (grocery store, etc.)<br>7 hh11_work3_e___7 don't know
550	[hh11_work4_e]<br><br>Show the field ONLY if:<br>[language] = '1' and [hhcount_e] > 10 and [hhcount_e] < 13 and ([hh11_work_e] = '1' or [hh11_work_e] = '2' or [hh11_work2_e] = '1')	Person 11: Does this person's employer offer them any of the following benefits at their current main job?<br>*Select all that apply.*	checkbox<br>1 hh11_work4_e___1 paid sick leave<br>2 hh11_work4_e___2 paid vacation/personal leave<br>3 hh11_work4_e___3 health insurance<br>4 hh11_work4_e___4 disability insurance<br>5 hh11_work4_e___5 retirement plan<br>6 hh11_work4_e___6 other<br>7 hh11_work4_e___7 don't know
551	[hh11_work5_e]<br><br>Show the field ONLY if:<br>[language] = '1' and [hhcount_e] > 10 and [hhcount_e] < 13 and ([hh11_work_e] = '1' or [hh11_work_e] = '2' or [hh11_work2_e] = '1')	Person 11: On a scale of 0 (definitely not going to happen) to 10 (definitely going to happen), how likely is it that this person will lose their job because of the COVID-19 pandemic?	text (number, Min: 0, Max: 10)
552	[hh11_work6_e]<br><br>Show the field ONLY if:<br>[language] = '1' and [hhcount_e] > 10 and [hhcount_e] < 13 and ([hh11_work_e] = '1' or [hh11_work_e] = '2' or [hh11_work2_e] = '1')	Person 11: On a scale of 0 (definitely not going to happen) to 10 (definitely going to happen), how likely is it that this person will receive fewer work hours at their job because of the COVID-19 pandemic?	text (number, Min: 0, Max: 10)
553	[hh11_work7_e]<br><br>Show the field ONLY if:<br>[language] = '1' and [hhcount_e] > 10 and [hhcount	Person 11: How often is this person required to work from outside of the home currently?	radio (Matrix)<br>1 always (100%)<br>2 most of the time (75%)

	_e] < 13 and ([hh11_work_e] = '1' or [hh11_work_e] = '2' or [hh11_work2_e] = '1')		3 half of the time (50%)<br>4 less than half of the time (25%)<br>5 never (0%)<br>6 don't know
554	[hh11_work8_e]<br><br>Show the field ONLY if:<br>[language] = '1' and ([hh11_work7_e] = '1' or [hh11_work7_e] = '2' or [hh11_work7_e] = '3' or [hh11_work7_e] = '4')	Person 11: How regularly is this person in close physical contact with co-workers during their work outside of the home currently?	radio (Matrix)<br>1 always (100%)<br>2 most of the time (75%)<br>3 half of the time (50%)<br>4 less than half of the time (25%)<br>5 never (0%)<br>6 don't know
555	[hh11_work9_e]<br><br>Show the field ONLY if:<br>[language] = '1' and ([hh11_work7_e] = '1' or [hh11_work7_e] = '2' or [hh11_work7_e] = '3' or [hh11_work7_e] = '4')	Person 11: How regularly is this person in close physical contact with clients during their work outside of the home currently?	radio (Matrix)<br>1 always (100%)<br>2 most of the time (75%)<br>3 half of the time (50%)<br>4 less than half of the time (25%)<br>5 never (0%)<br>6 don't know
556	[hh11_covidvaccine_e]<br><br>Show the field ONLY if:<br>[language] = '1' and [hhcount_e] > 10 and [hhcount_e] < 13	Person 11: Does this person plan to get a vaccine for COVID-19 when one becomes available?	radio<br>1 yes<br>0 no<br>2 don't know
557	[hh11_covidsymp_e]<br><br>Show the field ONLY if:<br>[language] = '1' and [hhcount_e] > 10 and [hhcount_e] < 13	Person 11: Has this person had any symptoms (cough, fever, difficulty breathing, fatigue, body aches, diarrhea, runny nose, loss of smell or taste) consistent with COVID-19 in the last two weeks?	radio<br>1 yes<br>0 no<br>2 don't know
558	[hh11_covidsymp2_e]<br><br>Show the field ONLY if:<br>[language] = '1' and [hh11_covidsymp_e] = '1'	Person 11: When did this person's symptoms begin?	text (date_mdy)
559	[hh11_covidsymp3_e]<br><br>Show the field ONLY if:<br>[language] = '1' and [hh11_covidsymp_e] = '1'	Person 11: Is this person worried that they may have had COVID-19 because of their symptoms?	radio<br>1 yes<br>0 no<br>2 don't know
560	[hh11_covidsymp4_e]<br><br>Show the field ONLY if:<br>[language] = '1' and [hh11_covidsymp_e] = '1'	Person 11: Did this person experience any bias or discrimination because of their symptoms?	radio<br>1 yes<br>0 no<br>2 don't know
561	[hh11_covidsymp5_e]<br><br>Show the field ONLY if:<br>[language] = '1' and [hh11_covidsymp_e] = '1'	Person 11: What did this person do in response to their symptoms?<br>*Select all that apply.*	checkbox<br>0 hh11_covidsymp5_e___0 nothing<br>1 hh11_covidsymp5_e___1 took over the counter medication

			(ibuprofen, acetaminophen, etc.)<br>2 | hh11_covidsymp5_e___2 | communicated with a health care provider over the phone<br>3 | hh11_covidsymp5_e___3 | visited a health care provider's office<br>4 | hh11_covidsymp5_e___4 | visited a retail clinic or pharmacy<br>5 | hh11_covidsymp5_e___5 | visited urgent care (FASTMed, etc.)<br>6 | hh11_covidsymp5_e___6 | visited the emergency room<br>7 | hh11_covidsymp5_e___7 | was admitted to the hospital<br>8 | hh11_covidsymp5_e___8 | other<br>9 | hh11_covidsymp5_e___9 | don't know
562	[hh11_covidsymp6_e]<br><br>Show the field ONLY if: [language] = '1' and [hh11_covidsymp5_e(8)] = '1'	Person 11: Please specify what other action this person took in response to their symptoms.	text
563	[hh11_covidsymp7_e]<br><br>Show the field ONLY if: [language] = '1' and ([hh11_covidsymp5_e(2)] = '1' or [hh11_covidsymp5_e(3)] = '1' or [hh11_covidsymp5_e(4)] = '1' or [hh11_covidsymp5_e(5)] = '1' or [hh11_covidsymp5_e(6)] = '1' or [hh11_covidsymp5_e(7)] = '1' or [hh11_covidsymp5_e(8)] = '1')	Person 11: Did a health care provider tell this person that they may have COVID-19?	radio<br>1 | yes<br>0 | no<br>2 | don't know
564	[hh11_covid_test_e]<br><br>Show the field ONLY if: [language] = '1' and [hh11_covidsymp_e] = '1'	Person 11: If this person received a COVID-19 test due to their symptoms, what was the result?	radio<br>1 | pending<br>2 | positive<br>3 | negative<br>4 | inconclusive<br>5 | did not receive a test<br>6 | don't know
565	[hh11_covid_admit_e]<br><br>Show the field ONLY if: [language] = '1' and [hh11_covidsymp5_e(7)] = '1'	Person 11: How many days was this person admitted to the hospital?	text (number, Min: 0)

566	[hh11_covid_admit2_e]<br>Show the field ONLY if:<br>[language] = '1' and [hh11_covidsymp5_e(7)] = '1'	Person 11: Did this person receive any of the following interventions during their hospital admission?	checkbox<br>1 hh11_covid_admit2_e___1 extra oxygen in your nose<br>2 hh11_covid_admit2_e___2 treatment in the intensive care unit (ICU)<br>3 hh11_covid_admit2_e___3 mechanical ventilation (intubation or a breathing tube)<br>4 hh11_covid_admit2_e___4 don't know
567	[hh11_covidsymp8_e]<br>Show the field ONLY if:<br>[language] = '1' and [hh11_covidsymp_e] = '1'	Person 11: Has this person returned to their normal health at this time?	radio<br>1 yes<br>0 no<br>2 don't know
568	[hh11_prevent_e]<br>Show the field ONLY if:<br>[language] = '1' and [hh11_covidsymp_e] = '1'	Person 11: Which of the following did this person do to protect their friends and family after their symptoms began?	checkbox<br>1 hh11_prevent_e___1 wore a mask more frequently<br>2 hh11_prevent_e___2 washed your hands with soap and water more frequently<br>3 hh11_prevent_e___3 used hand sanitizer more frequently<br>4 hh11_prevent_e___4 isolated yourself in your home more frequently<br>5 hh11_prevent_e___5 stayed home more frequently<br>6 hh11_prevent_e___6 wore disposable gloves more frequently<br>7 hh11_prevent_e___7 don't know
569	[hh12_relationship_e]<br>Show the field ONLY if:<br>[language] = '1' and [hhcount_e] > 11 and [hhcount_e] < 13	Section Header: *For each additional person in the your household, please provide the following information.*<br>Person 12: What is your relationship to this person?	radio<br>1 partner or spouse<br>2 child<br>3 parent<br>4 sibling<br>5 other family member<br>6 in-home childcare provider or other caregiver<br>7 other
570	[hh12_relationship2_e]<br>Show the field ONLY if:	Person 12: Please specify your relationship with this person.	text

	[language] = '1' and [hh12_relationship_e] = '7'		
571	[hh12_age_e]<br><br>Show the field ONLY if:<br>[language] = '1' and [hhcount_e] > 11 and [hhcount_e] < 13	Person 12: What is this person's age?<br>*Please specify their age in years*	text (number, Min: 0, Max: 110)
572	[hh12_sex_e]<br><br>Show the field ONLY if:<br>[language] = '1' and [hhcount_e] > 11 and [hhcount_e] < 13	Person 12: What is this person's sex?	radio<br>1 Female<br>2 Male<br>3 Other
573	[hh12_race_e]<br><br>Show the field ONLY if:<br>[language] = '1' and [hhcount_e] > 11 and [hhcount_e] < 13	Person 12: What is this person's race?<br>*Select all that apply.*	checkbox<br>1 hh12_race_e___1 American Indian or Alaska Native<br>2 hh12_race_e___2 Asian<br>3 hh12_race_e___3 Black or African American<br>4 hh12_race_e___4 Native Hawaiian or Pacific Islander<br>5 hh12_race_e___5 White<br>6 hh12_race_e___6 Other<br>7 hh12_race_e___7 don't know
574	[hh12_ethn_e]<br><br>Show the field ONLY if:<br>[language] = '1' and [hhcount_e] > 11 and [hhcount_e] < 13	Person 12: What is this person's ethnicity?	radio<br>1 Hispanic or Latino<br>2 Not Hispanic or Latino<br>3 Other<br>4 don't know
575	[hh12_edu_e]<br><br>Show the field ONLY if:<br>[language] = '1' and [hhcount_e] > 11 and [hhcount_e] < 13	Person 12: What is the highest level of education or schooling this person has completed?	radio<br>1 never attended school<br>2 kindergarten - 8th grade<br>3 some high school<br>4 high school equivalency (GED)<br>5 high school graduate<br>6 some college<br>7 college graduate<br>8 graduate school or more<br>9 don't know
576	[hh12_work_e]<br><br>Show the field ONLY if:<br>[language] = '1' and [hhcount_e] > 11 and [hhcount_e] < 13	Person 12: Which of the following best fit this person's current work situation?	radio<br>1 works full time<br>2 works part time<br>3 is looking for work/employment<br>4 retired<br>5 homemaker<br>6 student

			7 on maternity/paternity leave<br>8 on illness/sick leave<br>9 on disability<br>10 other<br>11 don't know
577	[hh12_work2_e]<br><br>Show the field ONLY if:<br>[language] = '1' and [hhcount_e] > 11 and [hhcount_e] < 13	Person 12: Does this person currently consider themselves self-employed (including as an independent contractor or gig-economy worker)?	radio<br>1 yes<br>0 no<br>2 don't know
578	[hh12_work3_e]<br><br>Show the field ONLY if:<br>[language] = '1' and [hhcount_e] > 11 and [hhcount_e] < 13 and ([hh12_work_e] = '1' or [hh12_work_e] = '2' or [hh12_work2_e] = '1')	Person 12: Does this person currently work in any of the following high-risk settings for COVID-19 transmission?	checkbox<br>1 hh12_work3_e___1 healthcare setting (hospital, clinic, urgent care, etc.)<br>2 hh12_work3_e___2 dense residential setting (nursing home, other long-term care facility)<br>3 hh12_work3_e___3 prison or jail<br>4 hh12_work3_e___4 meatpacking facility<br>5 hh12_work3_e___5 shipping or distribution facility<br>6 hh12_work3_e___6 high-volume retail facility (grocery store, etc.)<br>7 hh12_work3_e___7 don't know
579	[hh12_work4_e]<br><br>Show the field ONLY if:<br>[language] = '1' and [hhcount_e] > 11 and [hhcount_e] < 13 and ([hh12_work_e] = '1' or [hh12_work_e] = '2' or [hh12_work2_e] = '1')	Person 12: Does this person's employer offer them any of the following benefits at their current main job?<br>*Select all that apply.*	checkbox<br>1 hh12_work4_e___1 paid sick leave<br>2 hh12_work4_e___2 paid vacation/personal leave<br>3 hh12_work4_e___3 health insurance<br>4 hh12_work4_e___4 disability insurance<br>5 hh12_work4_e___5 retirement plan<br>6 hh12_work4_e___6 other<br>7 hh12_work4_e___7 don't know
580	[hh12_work5_e]<br><br>Show the field ONLY if:<br>[language] = '1' and [hhcount_e] > 11 and [hhcount_e] < 13 and ([hh12_work_e] = '1' or [hh12_work_e] = '2' or [hh12_work2_e] = '1')	Person 12: On a scale of 0 (definitely not going to happen) to 10 (definitely going to happen), how likely is it that this person will lose their job because of the COVID-19 pandemic?	text (number, Min: 0, Max: 10)
581	[hh12_work6_e]<br><br>Show the field ONLY if:<br>[language] = '1' and [hhcount_e] > 11 and [hhcount_e] < 13 and ([hh12_work	Person 12: On a scale of 0 (definitely not going to happen) to 10 (definitely going to happen), how likely is it that this person will receive fewer work hours at their job because of the COVID-19 pandemic?	text (number, Min: 0, Max: 10)

	_e] = '1' or [hh12_work_e] = '2' or [hh12_work2_e] = '1')		
582	[hh12_work7_e]<br>Show the field ONLY if: [language] = '1' and [hhcount_e] > 11 and [hhcount_e] < 13 and ([hh12_work_e] = '1' or [hh12_work_e] = '2' or [hh12_work2_e] = '1')	Person 12: How often is this person required to work from outside of the home currently?	radio (Matrix)<br>1 always (100%)<br>2 most of the time (75%)<br>3 half of the time (50%)<br>4 less than half of the time (25%)<br>5 never (0%)<br>6 don't know
583	[hh12_work8_e]<br>Show the field ONLY if: [language] = '1' and ([hh12_work7_e] = '1' or [hh12_work7_e] = '2' or [hh12_work7_e] = '3' or [hh12_work7_e] = '4')	Person 12: How regularly is this person in close physical contact with co-workers during their work outside of the home currently?	radio (Matrix)<br>1 always (100%)<br>2 most of the time (75%)<br>3 half of the time (50%)<br>4 less than half of the time (25%)<br>5 never (0%)<br>6 don't know
584	[hh12_work9_e]<br>Show the field ONLY if: [language] = '1' and ([hh12_work7_e] = '1' or [hh12_work7_e] = '2' or [hh12_work7_e] = '3' or [hh12_work7_e] = '4')	Person 12: How regularly is this person in close physical contact with clients during their work outside of the home currently?	radio (Matrix)<br>1 always (100%)<br>2 most of the time (75%)<br>3 half of the time (50%)<br>4 less than half of the time (25%)<br>5 never (0%)<br>6 don't know
585	[hh12_covidvaccine_e]<br>Show the field ONLY if: [language] = '1' and [hhcount_e] > 11 and [hhcount_e] < 13	Person 12: Does this person plan to get a vaccine for COVID-19 when one becomes available?	radio<br>1 yes<br>0 no<br>2 don't know
586	[hh12_covidsymp_e]<br>Show the field ONLY if: [language] = '1' and [hhcount_e] > 11 and [hhcount_e] < 13	Person 12: Has this person had any symptoms (cough, fever, difficulty breathing, fatigue, body aches, diarrhea, runny nose, loss of smell or taste) consistent with COVID-19 in the last two weeks?	radio<br>1 yes<br>0 no<br>2 don't know
587	[hh12_covidsymp2_e]<br>Show the field ONLY if: [language] = '1' and [hh12_covidsymp_e] = '1'	Person 12: When did this person's symptoms begin?	text (date_mdy)
588	[hh12_covidsymp3_e]<br>Show the field ONLY if: [language] = '1' and [hh12_covidsymp_e] = '1'	Person 12: Is this person worried that they may have had COVID-19 because of their symptoms?	radio<br>1 yes<br>0 no<br>2 don't know
589	[hh12_covidsymp4_e]<br>Show the field ONLY if: [language] = '1' and [hh12_covidsymp_e] = '1'	Person 12: Did this person experience any bias or discrimination because of their symptoms?	radio<br>1 yes<br>0 no

			2 don't know
590	[hh12_covidsymp5_e]<br>Show the field ONLY if:<br>[language] = '1' and [hh12_covidsymp_e] = '1'	Person 12: What did this person do in response to their symptoms?<br>*Select all that apply.*	checkbox<br>0 hh12_covidsymp5_e___0 nothing<br>1 hh12_covidsymp5_e___1 took over the counter medication (ibuprofen, acetaminophen, etc.)<br>2 hh12_covidsymp5_e___2 communicated with a health care provider over the phone<br>3 hh12_covidsymp5_e___3 visited a health care provider's office<br>4 hh12_covidsymp5_e___4 visited a retail clinic or pharmacy<br>5 hh12_covidsymp5_e___5 visited urgent care (FASTMed, etc.)<br>6 hh12_covidsymp5_e___6 visited the emergency room<br>7 hh12_covidsymp5_e___7 was admitted to the hospital<br>8 hh12_covidsymp5_e___8 other<br>9 hh12_covidsymp5_e___9 don't know
591	[hh12_covidsymp6_e]<br>Show the field ONLY if:<br>[language] = '1' and [hh12_covidsymp5_e(8)] = '1'	Person 12: Please specify what other action this person took in response to their symptoms.	text
592	[hh12_covidsymp7_e]<br>Show the field ONLY if:<br>[language] = '1' and ([hh12_covidsymp5_e(2)] = '1' or [hh12_covidsymp5_e(3)] = '1' or [hh12_covidsymp5_e(4)] = '1' or [hh12_covidsymp5_e(5)] = '1' or [hh12_covidsymp5_e(6)] = '1' or [hh12_covidsymp5_e(7)] = '1' or [hh12_covidsymp5_e(8)] = '1')	Person 12: Did a health care provider tell this person that they may have COVID-19?	radio<br>1 yes<br>0 no<br>2 don't know
593	[hh12_covid_test_e]<br>Show the field ONLY if:<br>[language] = '1' and [hh12_covidsymp_e] = '1'	Person 12: If this person received a COVID-19 test due to their symptoms, what was the result?	radio<br>1 pending<br>2 positive<br>3 negative<br>4 inconclusive<br>5 did not receive a test

			6 don't know
594	[hh12_covid_admit_e]<br><br>Show the field ONLY if:<br>[language] = '1' and [hh12_covidsymp5_e(7)] = '1'	Person 12: How many days was this person admitted to the hospital?	text (number, Min: 0)
595	[hh12_covid_admit2_e]<br><br>Show the field ONLY if:<br>[language] = '1' and [hh12_covidsymp5_e(7)] = '1'	Person 12: Did this person receive any of the following interventions during their hospital admission?	checkbox<br>1 hh12_covid_admit2_e___1 extra oxygen in your nose<br>2 hh12_covid_admit2_e___2 treatment in the intensive care unit (ICU)<br>3 hh12_covid_admit2_e___3 mechanical ventilation (intubation or a breathing tube)<br>4 hh12_covid_admit2_e___4 don't know
596	[hh12_covidsymp8_e]<br><br>Show the field ONLY if:<br>[language] = '1' and [hh12_covidsymp_e] = '1'	Person 12: Has this person returned to their normal health at this time?	radio<br>1 yes<br>0 no<br>2 don't know
597	[hh12_prevent_e]<br><br>Show the field ONLY if:<br>[language] = '1' and [hh12_covidsymp_e] = '1'	Person 12: Which of the following did this person do to protect their friends and family after their symptoms began?	checkbox<br>1 hh12_prevent_e___1 wore a mask more frequently<br>2 hh12_prevent_e___2 washed your hands with soap and water more frequently<br>3 hh12_prevent_e___3 used hand sanitizer more frequently<br>4 hh12_prevent_e___4 isolated yourself in your home more frequently<br>5 hh12_prevent_e___5 stayed home more frequently<br>6 hh12_prevent_e___6 wore disposable gloves more frequently<br>7 hh12_prevent_e___7 don't know
598	[serious1_e]<br><br>Show the field ONLY if:<br>[language] = '1'	Section Header: *Please provide the following information about your mental health and wellness.*<br><br>How serious a problem would you say the COVID-19 pandemic is for you personally right now?	radio<br>1 very serious<br>2 somewhat serious<br>3 not too serious<br>4 not at all serious
599	[serious2_e]<br><br>Show the field ONLY if:<br>[language] = '1'	How serious a problem would you say the COVID-19 pandemic is for people in your community right now?	radio<br>1 very serious<br>2 somewhat serious

			3 not too serious<br>4 not at all serious
600	[serious3_e]<br><br>Show the field ONLY if:<br>[language] = '1'	How serious a problem would you say the COVID-19 pandemic is for people in the United States right now?	radio<br>1 very serious<br>2 somewhat serious<br>3 not too serious<br>4 not at all serious
601	[serious4_e]<br><br>Show the field ONLY if:<br>[language] = '1'	How serious a problem would you say the COVID-19 pandemic is for people around the world right now?	radio<br>1 very serious<br>2 somewhat serious<br>3 not too serious<br>4 not at all serious
602	[concern1_e]<br><br>Show the field ONLY if:<br>[language] = '1'	Section Header: *The COVID-19 pandemic may cause challenges for some people regardless of whether they are infected. How concerned are you about each of the following things?*<br>Getting the healthcare you need (including care for mental health)	radio (Matrix)<br>1 not at all concerned<br>2 not too concerned<br>3 somewhat concerned<br>4 very concerned
603	[concern2_e]<br><br>Show the field ONLY if:<br>[language] = '1'	Having a place to live	radio (Matrix)<br>1 not at all concerned<br>2 not too concerned<br>3 somewhat concerned<br>4 very concerned
604	[concern3_e]<br><br>Show the field ONLY if:<br>[language] = '1'	Being able to interact with other people	radio (Matrix)<br>1 not at all concerned<br>2 not too concerned<br>3 somewhat concerned<br>4 very concerned
605	[concern4_e]<br><br>Show the field ONLY if:<br>[language] = '1'	Getting food, water, and other household supplies	radio (Matrix)<br>1 not at all concerned<br>2 not too concerned<br>3 somewhat concerned<br>4 very concerned
606	[concern5_e]<br><br>Show the field ONLY if:<br>[language] = '1'	Getting medication	radio (Matrix)<br>1 not at all concerned<br>2 not too concerned<br>3 somewhat concerned<br>4 very concerned
607	[concern6_e]<br><br>Show the field ONLY if:<br>[language] = '1'	Having transportation to get where you need to go	radio (Matrix)<br>1 not at all concerned<br>2 not too concerned<br>3 somewhat concerned

			4 very concerned
608	[concern7_e]<br><br>Show the field ONLY if:<br>[language] = '1'	Caring for your family and friends	radio (Matrix)<br>1 not at all concerned<br>2 not too concerned<br>3 somewhat concerned<br>4 very concerned
609	[gad7_1_e]<br><br>Show the field ONLY if:<br>[language] = '1'	Section Header: *During the last two weeks, how often have you been bothered by the following problems?*<br><br>Feeling nervous, anxious, or on edge	radio (Matrix)<br>0 not at all<br>1 several days<br>2 more than half of the days<br>3 nearly every day
610	[gad7_2_e]<br><br>Show the field ONLY if:<br>[language] = '1'	Not being able to stop or control worrying	radio (Matrix)<br>0 not at all<br>1 several days<br>2 more than half of the days<br>3 nearly every day
611	[gad7_3_e]<br><br>Show the field ONLY if:<br>[language] = '1'	Worrying too much about different things	radio (Matrix)<br>0 not at all<br>1 several days<br>2 more than half of the days<br>3 nearly every day
612	[gad7_4_e]<br><br>Show the field ONLY if:<br>[language] = '1'	Trouble relaxing	radio (Matrix)<br>0 not at all<br>1 several days<br>2 more than half of the days<br>3 nearly every day
613	[gad7_5_e]<br><br>Show the field ONLY if:<br>[language] = '1'	Being so restless that it's hard to sit still	radio (Matrix)<br>0 not at all<br>1 several days<br>2 more than half of the days<br>3 nearly every day
614	[gad7_6_e]<br><br>Show the field ONLY if:<br>[language] = '1'	Becoming easily annoyed or irritable	radio (Matrix)<br>0 not at all<br>1 several days<br>2 more than half of the days<br>3 nearly every day
615	[gad7_7_e]<br><br>Show the field ONLY if:<br>[language] = '1'	Feeling afraid as if something awful might happen	radio (Matrix)<br>0 not at all<br>1 several days<br>2 more than half of the days<br>3 nearly every day

616	[gad7_8_e]<br><br>Show the field ONLY if:<br>[language] = '1' and ([gad7_1_e] > 0 or [gad7_2_e] > 0 or [gad7_3_e] > 0 or [gad7_4_e] > 0 or [gad7_5_e] > 0 or [gad7_6_e] > 0 or [gad7_7_e] > 0)	How difficult have these problems made it for you to do your work, take care of things at home, or get along with other people?	radio<br>0 not difficult<br>1 somewhat difficult<br>2 very difficult<br>3 extremely difficult
617	[cesd_1_e_b]<br><br>Show the field ONLY if:<br>[language] = '1'	Section Header: *During the last two weeks, how often have you experienced the following behaviors and feelings?*<br><br>I was bothered by things that usually don't bother me.	radio (Matrix)<br>0 Rarely or none of the time (less than 1 day)<br>1 Some or a little of the time (1-2 days)<br>2 Occasionally or a moderate amount of the time (3-4 days)<br>3 Most or all of the time (5-7 days)
618	[cesd_2_e_b]<br><br>Show the field ONLY if:<br>[language] = '1'	I did not feel like eating; my appetite was poor.	radio (Matrix)<br>0 Rarely or none of the time (less than 1 day)<br>1 Some or a little of the time (1-2 days)<br>2 Occasionally or a moderate amount of the time (3-4 days)<br>3 Most or all of the time (5-7 days)
619	[cesd_3_e_b]<br><br>Show the field ONLY if:<br>[language] = '1'	I felt that I could not shake off the blues even with help from my family or friends.	radio (Matrix)<br>0 Rarely or none of the time (less than 1 day)<br>1 Some or a little of the time (1-2 days)<br>2 Occasionally or a moderate amount of the time (3-4 days)<br>3 Most or all of the time (5-7 days)
620	[cesd_4_e_b]<br><br>Show the field ONLY if:<br>[language] = '1'	I felt I was just as good as other people.	radio (Matrix)<br>0 Rarely or none of the time (less than 1 day)<br>1 Some or a little of the time (1-2 days)<br>2 Occasionally or a moderate amount of the time (3-4 days)<br>3 Most or all of the time (5-7 days)
621	[cesd_5_e_b]<br><br>Show the field ONLY if:<br>[language] = '1'	I had trouble keeping my mind on what I was doing.	radio (Matrix)<br>0 Rarely or none of the time (less than 1 day)<br>1 Some or a little of the time (1-2 days)<br>2 Occasionally or a moderate amount of the time (3-4 days)<br>3 Most or all of the time (5-7 days)
622	[cesd_6_e_b]<br><br>Show the field ONLY if:<br>[language] = '1'	I felt depressed.	radio (Matrix)<br>0 Rarely or none of the time (less than 1 day)<br>1 Some or a little of the time (1-2 days)

			2 Occasionally or a moderate amount of the time (3-4 days)<br>3 Most or all of the time (5-7 days)
623	[cesd_7_e_b]<br>Show the field ONLY if:<br>[language] = '1'	I felt that everything I did was an effort.	radio (Matrix)<br>0 Rarely or none of the time (less than 1 day)<br>1 Some or a little of the time (1-2 days)<br>2 Occasionally or a moderate amount of the time (3-4 days)<br>3 Most or all of the time (5-7 days)
624	[cesd_8_e_b]<br>Show the field ONLY if:<br>[language] = '1'	I felt hopeful about the future.	radio (Matrix)<br>0 Rarely or none of the time (less than 1 day)<br>1 Some or a little of the time (1-2 days)<br>2 Occasionally or a moderate amount of the time (3-4 days)<br>3 Most or all of the time (5-7 days)
625	[cesd_9_e_b]<br>Show the field ONLY if:<br>[language] = '1'	I thought my life had been a failure.	radio (Matrix)<br>0 Rarely or none of the time (less than 1 day)<br>1 Some or a little of the time (1-2 days)<br>2 Occasionally or a moderate amount of the time (3-4 days)<br>3 Most or all of the time (5-7 days)
626	[cesd_10_e_b]<br>Show the field ONLY if:<br>[language] = '1'	I felt fearful.	radio (Matrix)<br>0 Rarely or none of the time (less than 1 day)<br>1 Some or a little of the time (1-2 days)<br>2 Occasionally or a moderate amount of the time (3-4 days)<br>3 Most or all of the time (5-7 days)
627	[cesd_11_e_b]<br>Show the field ONLY if:<br>[language] = '1'	My sleep was restless.	radio (Matrix)<br>0 Rarely or none of the time (less than 1 day)<br>1 Some or a little of the time (1-2 days)<br>2 Occasionally or a moderate amount of the time (3-4 days)<br>3 Most or all of the time (5-7 days)
628	[cesd_12_e_b]<br>Show the field ONLY if:<br>[language] = '1'	I was happy.	radio (Matrix)<br>0 Rarely or none of the time (less than 1 day)<br>1 Some or a little of the time (1-2 days)<br>2 Occasionally or a moderate amount of the time (3-4 days)<br>3 Most or all of the time (5-7 days)
629	[cesd_13_e_b]	I talked less than usual.	radio (Matrix)

	Show the field ONLY if: [language] = '1'		0 Rarely or none of the time (less than 1 day)<br>1 Some or a little of the time (1-2 days)<br>2 Occasionally or a moderate amount of the time (3-4 days)<br>3 Most or all of the time (5-7 days)
630	[cesd_14_e_b]<br>Show the field ONLY if: [language] = '1'	I felt lonely.	radio (Matrix)<br>0 Rarely or none of the time (less than 1 day)<br>1 Some or a little of the time (1-2 days)<br>2 Occasionally or a moderate amount of the time (3-4 days)<br>3 Most or all of the time (5-7 days)
631	[cesd_15_e_b]<br>Show the field ONLY if: [language] = '1'	People were unfriendly.	radio (Matrix)<br>0 Rarely or none of the time (less than 1 day)<br>1 Some or a little of the time (1-2 days)<br>2 Occasionally or a moderate amount of the time (3-4 days)<br>3 Most or all of the time (5-7 days)
632	[cesd_16_e_b]<br>Show the field ONLY if: [language] = '1'	I enjoyed life.	radio (Matrix)<br>0 Rarely or none of the time (less than 1 day)<br>1 Some or a little of the time (1-2 days)<br>2 Occasionally or a moderate amount of the time (3-4 days)<br>3 Most or all of the time (5-7 days)
633	[cesd_17_e_b]<br>Show the field ONLY if: [language] = '1'	I had crying spells.	radio (Matrix)<br>0 Rarely or none of the time (less than 1 day)<br>1 Some or a little of the time (1-2 days)<br>2 Occasionally or a moderate amount of the time (3-4 days)<br>3 Most or all of the time (5-7 days)
634	[cesd_18_e_b]<br>Show the field ONLY if: [language] = '1'	I felt sad.	radio (Matrix)<br>0 Rarely or none of the time (less than 1 day)<br>1 Some or a little of the time (1-2 days)<br>2 Occasionally or a moderate amount of the time (3-4 days)<br>3 Most or all of the time (5-7 days)
635	[cesd_19_e_b]<br>Show the field ONLY if: [language] = '1'	I felt that people disliked me.	radio (Matrix)<br>0 Rarely or none of the time (less than 1 day)<br>1 Some or a little of the time (1-2 days)

			2 Occasionally or a moderate amount of the time (3-4 days)<br>3 Most or all of the time (5-7 days)
636	[cesd_20_e_b]<br><br>Show the field ONLY if:<br>[language] = '1'	I could not "get going".	radio (Matrix)<br>0 Rarely or none of the time (less than 1 day)<br>1 Some or a little of the time (1-2 days)<br>2 Occasionally or a moderate amount of the time (3-4 days)<br>3 Most or all of the time (5-7 days)
637	[sample_e]<br><br>Show the field ONLY if:<br>[language] = '1'	Are you willing to provide a painless nasal swab and painless blood sample for this study of COVID-19 in Chatham county?	yesno<br>1 Yes<br>0 No
638	[survey_e]<br><br>Show the field ONLY if:<br>[language] = '1'	How did you complete this survey?	radio<br>1 on a computer (laptop or desktop)<br>2 on a mobile device (cell phone or tablet)<br>3 on a phone with an interviewer<br>4 other
639	[race_s]<br><br>Show the field ONLY if:<br>[language] = '2'	¿Cuál es su raza?<br>*Seleccione todo lo que corresponda.*	checkbox, Required<br>1 race_s___1 Indio americano o nativo de Alaska<br>2 race_s___2 Asiático<br>3 race_s___3 Negro o afroestadounidense<br>4 race_s___4 Nativo de Hawái o de las islas del Pacifico<br>5 race_s___5 Blanco<br>6 race_s___6 Otra<br>7 race_s___7 Prefiero no responder
640	[ethn_s]<br><br>Show the field ONLY if:<br>[language] = '2'	¿Cuál es su identidad étnica?	radio, Required<br>1 Hispano o latino<br>2 Ni hispano ni latino<br>3 Otra<br>4 Prefiero no responder
641	[edu_s]<br><br>Show the field ONLY if:<br>[language] = '2'	¿Qué nivel de educación tiene usted?	radio<br>1 No tiene educación formal<br>2 Kinder a 8° grado<br>3 Estudios de educación secundaria<br>4 Equivalencia de educación secundaria<br>5 Diploma de educación secundaria<br>6 Estudios de educación superior<br>7 Título universitario<br>8 Estudios de posgrado o más
642	[smoke_cur_s]	¿Fuma usted cigarillos, cigarros o pipa a diario?	radio

	Show the field ONLY if: [language] = '2'		1 Sí<br>0 No
643	[smoke_cur2_s]<br><br>Show the field ONLY if: [language] = '2' and [smoke_cur_s] = '1'	¿A qué edad comenzó usted a fumar con regularidad?	text (number, Min: 0, Max: 100)
644	[smoke_cur3_s]<br><br>Show the field ONLY if: [language] = '2' and [smoke_cur_s] = '1'	En promedio ¿cuántos cigarrillos, cigarros o pipa fuma usted al día desde que comenzó a fumar?	radio<br>0 0<br>1 1-10<br>2 11-20<br>3 21-30<br>4 31-40<br>5 41-50<br>6 51-60<br>7 61 o más
645	[smoke_past_s]<br><br>Show the field ONLY if: [language] = '2' and [smoke_cur_s] = '0'	¿Fumaba usted cigarrillos, cigarros o pipas a diario antes?	radio<br>1 Sí<br>0 No
646	[smoke_past2_s]<br><br>Show the field ONLY if: [language] = '2' and [smoke_past_s] = '1'	¿A qué edad comenzó usted a fumar con regularidad?	text (number, Min: 0, Max: 100)
647	[smoke_past3_s]<br><br>Show the field ONLY if: [language] = '2' and [smoke_past_s] = '1'	¿Hace cuántos años que dejó de fumar?	text (number, Min: 0, Max: 100)
648	[smoke_past4_s]<br><br>Show the field ONLY if: [language] = '2' and [smoke_past_s] = '1'	En promedio ¿cuántos cigarrillos, cigarros o pipas fumaba al día cuando fumaba?	radio<br>0 0<br>1 1-10<br>2 11-20<br>3 21-30<br>4 31-40<br>5 41-50<br>6 51-60<br>7 61 o más
649	[vape_cur_s]<br><br>Show the field ONLY if: [language] = '2'	¿Fuma usted cigarrillos electrónicos o de vapor?	radio<br>1 Sí<br>0 No
650	[vape_cur2_s]<br><br>Show the field ONLY if: [language] = '2' and [vape_cur_s] = '1'	¿A qué edad comenzó usted a fumar cigarrillos electrónicos con regularidad?	text (number, Min: 0, Max: 100)
651	[vape_cur3_s]<br><br>Show the field ONLY if:	En promedio ¿cuántas bocanadas de cigarrillo electrónico o de vapor inhala usted al día?	radio<br>0 0-25

	[language] = '2' and [vape_cur_s] = '1'		1 26-50<br>2 51-75<br>3 76-100<br>4 101-125<br>5 126-150<br>6 151-175<br>7 176-200<br>8 201-225<br>9 226-250<br>10 251 o más
652	[vape_past_s]<br><br>Show the field ONLY if: [language] = '2' and [vape_cur_s] = '0'	¿Fumaba usted cigarrillos electrónicos o de vapor antes?	radio<br>1 Sí<br>0 No
653	[vape_past2_s]<br><br>Show the field ONLY if: [language] = '2' and [vape_past_s] = '1'	¿A qué edad comenzó usted a fumar cigarrillos electrónicos con regularidad?	text (number, Min: 0, Max: 100)
654	[vape_past3_s]<br><br>Show the field ONLY if: [language] = '2' and [vape_past_s] = '1'	¿Hace cuántos años que dejó de fumar cigarrillos electrónicos?	text (number, Min: 0, Max: 100)
655	[vape_past4_s]<br><br>Show the field ONLY if: [language] = '2' and [vape_past_s] = '1'	En promedio ¿cuántas bocanadas de cigarrillo electrónico (o de otros productos de vapor) inhalaba usted al día?	radio<br>0 0-25<br>1 26-50<br>2 51-75<br>3 76-100<br>4 101-125<br>5 126-150<br>6 151-175<br>7 176-200<br>8 201-225<br>9 226-250<br>10 251 o más
656	[alc_cur_s]<br><br>Show the field ONLY if: [language] = '2'	¿Toma usted bebidas alcohólicas al menos una vez a la semana?	yesno<br>1 Yes<br>0 No
657	[alc_cur2_s]<br><br>Show the field ONLY if: [language] = '2' and [alc_cur_s] = '1'	¿A qué edad comenzó usted a tomar bebidas alcohólicas con regularidad?	text (number, Min: 0, Max: 100)
658	[alc_cur3_s]<br><br>Show the field ONLY if: [language] = '2' and [alc_cur_s] = '1'	En los últimos 30 días, incluido el día de hoy ¿cuántos días tomó usted una o varias bebidas alcohólicas?	text (number, Min: 0, Max: 30)

659	[alc_cur4_s]<br><br>Show the field ONLY if:<br>[language] = '2' and [alc_cur_s] = '1' and [alc_cur3_s] > '0'	En los últimos 30 días, ¿cuántas bebidas alcohólicas tomó por lo general cada día que bebió?<br>*(Una bebida es igual a 5 onzas de vino (una copa de vino), 12 onzas de cerveza (una lata o una botella de cerveza) o 1.5 onzas de licor (un trago o un coctel).)*	text (number, Min: 0, Max: 30)
660	[alc_past_s]<br><br>Show the field ONLY if:<br>[language] = '2' and [alc_cur_s] = '0'	¿Tomaba usted antes bebidas alcohólicas al menos una vez a la semana?	radio<br>1 Sí<br>0 No
661	[alc_past2_s]<br><br>Show the field ONLY if:<br>[language] = '2' and [alc_past_s] = '1'	¿A qué edad comenzó usted a tomar bebidas alcohólicas con regularidad?	text (number, Min: 0, Max: 100)
662	[alc_past3_s]<br><br>Show the field ONLY if:<br>[language] = '2' and [alc_past_s] = '1'	¿Hace cuántos años que dejó de tomar bebidas alcohólicas?	text (number, Min: 0, Max: 100)
663	[alc_past4_s]<br><br>Show the field ONLY if:<br>[language] = '2' and [alc_past_s] = '1'	Durante un periodo normal de 30 dias, ¿cuántos días tomaba usted una o varias bebidas alcohólicas?	text (number, Min: 0, Max: 30)
664	[alc_past5_s]<br><br>Show the field ONLY if:<br>[language] = '2' and [alc_past_s] = '1' and [alc_past4_s] > '0'	Durante un período normal de 30 días, ¿cuántos días tomaba usted una o varias bebidas alcohólicas? ¿Cuántas bebidas alcohólicas tomaba usted por lo general cada día que bebía?<br>*(Una bebida es igual a 5 onzas de vino (una copa de vino), 12 onzas de cerveza (una lata o una botella de cerveza) o 1.5 onzas de licor (un trago o un coctel).)*	text (number, Min: 0, Max: 30)
665	[exercise_s]<br><br>Show the field ONLY if:<br>[language] = '2'	¿Hace usted al menos una vez a la semana actividades físicas tales como caminar rápido, trotar, andar en bicicleta, nadar, etc. hasta sudar, acelerar el pulso o jadear?	radio<br>1 Sí<br>0 No
666	[exercise2_s]<br><br>Show the field ONLY if:<br>[language] = '2' and [exercise_s] = "1"	En promedio, ¿cuántos días a la semana hace usted este tipo de actividades?	radio<br>1 1<br>2 2<br>3 3<br>4 4<br>5 5<br>6 6<br>7 7
667	[exercise3_s]<br><br>Show the field ONLY if:<br>[language] = '2' and [exercise_s] = "1"	En promedio, ¿cuántos minutos al día hace usted este tipo de actividades?	radio<br>0 0-20<br>1 21-40<br>2 41-60<br>3 61 o más
668	[exercise4_s]<br><br>Show the field ONLY if:<br>[language] = '2' and [exercise_s] = "1"	Cuando hace ejercicio normalmente, ¿cómo calificaría usted su nivel promedio de esfuerzo?	radio<br>0 Leve / calentamiento<br>1 Medio (puede conversar) / desarrollo aeróbico

			2 Intenso (pero puede esforzarse para continuar) / resistencia aeróbica<br>3 Muy intenso (no puede conversar) / resistencia anaeróbica<br>4 ™ Extremadamente intenso (jadeo, su cuerpo quiere parar) / velocidad, potencia
669	[work_s]<br>Show the field ONLY if:<br>[language] = '2'	¿Cuál de las siguientes es la mejor descripción de su situación laboral antes de que comenzara la pandemia de COVID-19 en Carolina del Norte?	radio<br>1 Empleo a tiempo completo<br>2 Empleo a tiempo parcial<br>3 En busca de empleo<br>4 Jubilado<br>5 Ama de casa<br>6 Estudiante<br>7 Permiso de maternidad o paternidad<br>8 Permiso por enfermedad<br>9 Desempleado por discapacidad<br>10 Otra
670	[work2_s]<br>Show the field ONLY if:<br>[language] = '2'	Antes de que comenzara la pandemia de COVID-19 en Carolina del Norte, ¿se consideraba usted empleado por cuenta propia (contratista independiente, trabajador esporádico (gig), etc.)?	radio<br>1 Sí<br>0 No<br>2 No sabe
671	[work3_s]<br>Show the field ONLY if:<br>[language] = '2' and ([work_s] = '1' or [work_s] = '2' or [work2_s] = '1')	¿Cuál de las siguientes es la mejor descripción de su empleo principal (empleo en el que pasaba la mayor parte del tiempo o que había tenido por más tiempo) antes de la pandemia de COVID-19 en Carolina del Norte?	radio<br>1 Administrativo<br>2 Profesional<br>3 Apoyo administrativo<br>4 Servicio<br>5 Agricultura, silvicultura o pesca<br>6 Produccion de precisión, artesania o reparación<br>7 Operario, fabricante u obrero<br>8 Militar<br>9 Estudiante<br>10 Desempleado<br>11 Otra
672	[work4_s]<br>Show the field ONLY if:<br>[language] = '2' and ([work_s] = '1' or [work_s] = '2' or [work2_s] = '1')	¿Cuántos años llevaba usted en su empleo principal?	text (number, Min: 0, Max: 70)
673	[work5_s]<br>Show the field ONLY if:<br>[language] = '2' and ([work_s] = '1' or [work_s] = '2' or [work2_s] = '1')	En su empleo principal antes de la pandemia de COVID-19, ¿ofrecía su empleador alguno de los siguientes beneficios?<br>*Seleccione todo lo que corresponda.*	checkbox<br>1 work5_s___1 Permiso por enfermedad con goce de sueldo<br>2 work5_s___2 Vacaciones o permiso personal con goce de

			sueldo<br>3 work5_s___3 Seguro de salud<br>4 work5_s___4 Seguro de discapacidad<br>5 work5_s___5 Plan de jubilación<br>6 work5_s___6 Otro
674	[work6_s]<br><br>Show the field ONLY if: [language] = '2' and ([work_s] = '1' or [work_s] = '2' or [work2_s] = '1')	¿Ha cambiado su situación laboral desde que comenzó la pandemia de COVID-19 en Carolina del Norte?	radio<br>1 Sí<br>0 No
675	[work7_s]<br><br>Show the field ONLY if: [language] = '2' and [work6_s] = '1'	¿Cuál de las siguientes es la mejor descripción de su situación laboral actual?	radio<br>1 Empleo a tiempo completo<br>2 Empleo a tiempo parcial<br>3 En busca de empleo<br>4 Jubilado<br>5 Ama de casa<br>6 Estudiante<br>7 Permiso de maternidad o paternidad<br>8 Permiso por enfermedad<br>9 Desempleado por discapacidad<br>10 Otra
676	[work8_s]<br><br>Show the field ONLY if: [language] = '2' and [work6_s] = '1'	¿Se considera usted actualmente empleado por cuenta propia (contratista independiente, trabajador esporádico (gig), etc.)?	radio<br>1 Sí<br>0 No<br>2 no sabe
677	[work9_s]<br><br>Show the field ONLY if: [language] = '2' and [work6_s] = '1'	Del trabajo (o trabajos) que desempena actualmente, cual de las siguientes opciones describe mejor su trabajo principal (el trabajo en que pasa mas horas, o el trabajo en que ha trabajado mas tiempo?	radio<br>1 Administrativo<br>2 Profesional<br>3 Apoyo administrativo<br>4 Servicio<br>5 Agricultura, silvicultura o pesca<br>6 Produccion de precisión, artesania o reparación<br>7 Operario, fabricante u obrero<br>8 Militar<br>9 Estudiante<br>10 Desempleado<br>11 Otra
678	[work10_s]<br><br>Show the field ONLY if: [language] = '2' and [work6_s] = '1'	¿Cuántos meses lleva usted en su empleo principal actual?	text (number, Min: 0, Max: 70)

679	[work11_s]<br><br>Show the field ONLY if:<br>[language] = '2' and [work6_s] = '1'	En su empleo principal actual, ¿ofrece su empleador alguno de los siguientes beneficios?<br>*Seleccione todo lo que corresponda.*	radio<br>1 Permiso por enfermedad con goce de sueldo<br>2 Vacaciones o permiso personal con goce de sueldo<br>3 Seguro de salud<br>4 Seguro de discapacidad<br>5 Plan de jubilación<br>6 Otro
680	[work12_s]<br><br>Show the field ONLY if:<br>[language] = '2' and ([work_s] = '1' or [work_s] = '2' or [work2_s] = '1') and [work6_s] = '0'	En una escala de 0 (definativamente no va a suceder) a 10 (definativamente va a suceder), ¿qué tan probable es que usted pierda su empleo debido a la pandemia de COVID-19?	text (number, Min: 0, Max: 10)
681	[work13_s]<br><br>Show the field ONLY if:<br>[language] = '2' and ([work_s] = '1' or [work_s] = '2' or [work2_s] = '1') and [work6_s] = '0'	En una escala de 0 (definativamente no va a suceder) a 10 (definativamente va a suceder), ¿qué tan probable es que le asignen menos horas de trabajo debido a la pandemia de COVID-19?	text (number, Min: 0, Max: 10)
682	[work14_s]<br><br>Show the field ONLY if:<br>[language] = '2' and ([work_s] = '1' or [work_s] = '2' or [work2_s] = '1') and [work6_s] = '0'	Antes de la pandemia de COVID-19 en Carolina del Norte, ¿con qué frecuencia se le exigía que trabajara fuera de su residencia?	radio (Matrix)<br>1 Todo el tiempo (100%)<br>2 La mayor parte del tiempo (75%)<br>3 La mitad del tiempo (50%)<br>4 Menos de la mitad del tiempo (25%)<br>5 Nunca (0%)
683	[work15_s]<br><br>Show the field ONLY if:<br>[language] = '2' and ([work14_s] = '1' or [work14_s] = '2' or [work14_s] = '3' or [work14_s] = '4')	Antes de la pandemia de COVID-19, ¿con qué frecuencia se encontraba usted físicamente cerca de sus compañeros de trabajo mientras trabajaba fuera de su residencia?	radio (Matrix)<br>1 Todo el tiempo (100%)<br>2 La mayor parte del tiempo (75%)<br>3 La mitad del tiempo (50%)<br>4 Menos de la mitad del tiempo (25%)<br>5 Nunca (0%)
684	[work16_s]<br><br>Show the field ONLY if:<br>[language] = '2' and ([work14_s] = '1' or [work14_s] = '2' or [work14_s] = '3' or [work14_s] = '4')	Antes de la pandemia de COVID-19, ¿con qué frecuencia se encontraba usted físicamente cerca de los clientes mientras trabajaba fuera de su residencia?	radio (Matrix)<br>1 Todo el tiempo (100%)<br>2 La mayor parte del tiempo (75%)<br>3 La mitad del tiempo (50%)<br>4 Menos de la mitad del tiempo (25%)<br>5 Nunca (0%)
685	[work17_s]<br><br>Show the field ONLY if:<br>[language] = '2' and ([work14_s] = '1' or [work14_s] = '2' or [work14_s] = '3' or [work14_s] = '4')	Antes de la pandemia de COVID-19, ¿con qué frecuencia tenía acceso a guantes desechables mientras trabajaba fuera de su residencia?	radio (Matrix)<br>1 Todo el tiempo (100%)<br>2 La mayor parte del tiempo (75%)<br>3 La mitad del tiempo (50%)<br>4 Menos de la mitad del tiempo (25%)<br>5 Nunca (0%)

686	[work18_s]<br><br>Show the field ONLY if:<br>[language] = '2' and ([work14_s] = '1' or [work14_s] = '2' or [work14_s] = '3' or [work14_s] = '4')	Antes de la pandemia de COVID-19, ¿con qué frecuencia tenía acceso a una mascarilla mientras trabajaba fuera de su residencia?	radio (Matrix)<br>1 Todo el tiempo (100%)<br>2 La mayor parte del tiempo (75%)<br>3 La mitad del tiempo (50%)<br>4 Menos de la mitad del tiempo (25%)<br>5 Nunca (0%)
687	[work19_s]<br><br>Show the field ONLY if:<br>[language] = '2' and ([work14_s] = '1' or [work14_s] = '2' or [work14_s] = '3' or [work14_s] = '4')	Antes de la pandemia de COVID-19, ¿con qué frecuencia llevaba usted guantes desechables mientras trabajaba fuera de su residencia?	radio (Matrix)<br>1 Todo el tiempo (100%)<br>2 La mayor parte del tiempo (75%)<br>3 La mitad del tiempo (50%)<br>4 Menos de la mitad del tiempo (25%)<br>5 Nunca (0%)
688	[work20_s]<br><br>Show the field ONLY if:<br>[language] = '2' and ([work14_s] = '1' or [work14_s] = '2' or [work14_s] = '3' or [work14_s] = '4')	Antes de la pandemia de COVID-19, ¿con qué frecuencia llevaba usted una mascarilla mientras trabajaba fuera de su residencia?	radio (Matrix)<br>1 Todo el tiempo (100%)<br>2 La mayor parte del tiempo (75%)<br>3 La mitad del tiempo (50%)<br>4 Menos de la mitad del tiempo (25%)<br>5 Nunca (0%)
689	[work21_s]<br><br>Show the field ONLY if:<br>[language] = '2' and ([work14_s] = '1' or [work14_s] = '2' or [work14_s] = '3' or [work14_s] = '4')	Antes de la pandemia de COVID-19,¿con qué frecuencia se lavaba las manos con agua y jabón mientras trabajaba fuera de su residencia?	radio (Matrix)<br>1 Todo el tiempo (100%)<br>2 La mayor parte del tiempo (75%)<br>3 La mitad del tiempo (50%)<br>4 Menos de la mitad del tiempo (25%)<br>5 Nunca (0%)
690	[work22_s]<br><br>Show the field ONLY if:<br>[language] = '2' and ([work14_s] = '1' or [work14_s] = '2' or [work14_s] = '3' or [work14_s] = '4')	Antes de la pandemia de COVID-19, ¿con qué frecuencia se desinfectaba las manos con desinfectante para manos mientras trabajaba fuera de su residencia?	radio (Matrix)<br>1 Todo el tiempo (100%)<br>2 La mayor parte del tiempo (75%)<br>3 La mitad del tiempo (50%)<br>4 Menos de la mitad del tiempo (25%)<br>5 Nunca (0%)
691	[work23_s]<br><br>Show the field ONLY if:<br>[language] = '2' and ([work14_s] = '1' or [work14_s] = '2' or [work14_s] = '3' or [work14_s] = '4')	Antes de la pandemia de COVID-19, ¿cuánto le preocupaba exponerse a la COVID-19 mientras trabajaba fuera de su residencia?	radio (Matrix)<br>1 Todo el tiempo (100%)<br>2 La mayor parte del tiempo (75%)<br>3 La mitad del tiempo (50%)<br>4 Menos de la mitad del tiempo (25%)<br>5 Nunca (0%)
692	[work24_s]<br><br>Show the field ONLY if:<br>[language] = '2'	Actualmente, ¿con qué frecuencia se le exige que trabaje fuera de su residencia?	radio (Matrix)<br>1 Todo el tiempo (100%)<br>2 La mayor parte del tiempo (75%)<br>3 La mitad del tiempo (50%)<br>4 Menos de la mitad del tiempo (25%)<br>5 Nunca (0%)

693	[work25_s]<br><br>Show the field ONLY if: [language] = '2' and ([work24_s] = '1' or [work24_s] = '2' or [work24_s] = '3' or [work24_s] = '4')	Actualmente, ¿con qué frecuencia se encuentra usted físicamente cerca de sus compañeros de trabajo mientras trabaja fuera de su residencia?	radio (Matrix)<br>1 Todo el tiempo (100%)<br>2 La mayor parte del tiempo (75%)<br>3 La mitad del tiempo (50%)<br>4 Menos de la mitad del tiempo (25%)<br>5 Nunca (0%)
694	[work26_s]<br><br>Show the field ONLY if: [language] = '2' and ([work24_s] = '1' or [work24_s] = '2' or [work24_s] = '3' or [work24_s] = '4')	Actualmente, ¿con qué frecuencia se encuentra usted físicamente cerca de los clientes mientras trabaja fuera de su residencia?	radio (Matrix)<br>1 Todo el tiempo (100%)<br>2 La mayor parte del tiempo (75%)<br>3 La mitad del tiempo (50%)<br>4 Menos de la mitad del tiempo (25%)<br>5 Nunca (0%)
695	[work27_s]<br><br>Show the field ONLY if: [language] = '2' and ([work24_s] = '1' or [work24_s] = '2' or [work24_s] = '3' or [work24_s] = '4')	Actualmente, ¿con qué frecuencia tiene acceso a guantes desechables mientras trabaja fuera de su residencia?	radio (Matrix)<br>1 Todo el tiempo (100%)<br>2 La mayor parte del tiempo (75%)<br>3 La mitad del tiempo (50%)<br>4 Menos de la mitad del tiempo (25%)<br>5 Nunca (0%)
696	[work28_s]<br><br>Show the field ONLY if: [language] = '2' and ([work24_s] = '1' or [work24_s] = '2' or [work24_s] = '3' or [work24_s] = '4')	Actualmente, ¿con qué frecuencia tiene acceso a una mascarilla mientras trabaja fuera de su residencia?	radio (Matrix)<br>1 Todo el tiempo (100%)<br>2 La mayor parte del tiempo (75%)<br>3 La mitad del tiempo (50%)<br>4 Menos de la mitad del tiempo (25%)<br>5 Nunca (0%)
697	[work29_s]<br><br>Show the field ONLY if: [language] = '2' and ([work24_s] = '1' or [work24_s] = '2' or [work24_s] = '3' or [work24_s] = '4')	Actualmente, ¿con qué frecuencia lleva usted guantes desechables mientras trabaja fuera de su residencia?	radio (Matrix)<br>1 Todo el tiempo (100%)<br>2 La mayor parte del tiempo (75%)<br>3 La mitad del tiempo (50%)<br>4 Menos de la mitad del tiempo (25%)<br>5 Nunca (0%)
698	[work30_s]<br><br>Show the field ONLY if: [language] = '2' and ([work24_s] = '1' or [work24_s] = '2' or [work24_s] = '3' or [work24_s] = '4')	Actualmente, ¿con qué frecuencia lleva usted una mascarilla mientras trabaja fuera de su residencia?	radio (Matrix)<br>1 Todo el tiempo (100%)<br>2 La mayor parte del tiempo (75%)<br>3 La mitad del tiempo (50%)<br>4 Menos de la mitad del tiempo (25%)<br>5 Nunca (0%)
699	[work31_s]<br><br>Show the field ONLY if: [language] = '2' and ([work24_s] = '1' or [work24_s] = '2' or [work24_s] = '3' or [work24_s] = '4')	Actualmente, ¿con qué frecuencia se lava las manos con agua y jabón mientras trabaja fuera de su residencia?	radio (Matrix)<br>1 Todo el tiempo (100%)<br>2 La mayor parte del tiempo (75%)<br>3 La mitad del tiempo (50%)<br>4 Menos de la mitad del tiempo (25%)<br>5 Nunca (0%)

700	[work32_s]<br><br>Show the field ONLY if:<br>[language] = '2' and ([work24_s] = '1' or [work24_s] = '2' or [work24_s] = '3' or [work24_s] = '4')	Actualmente, ¿con qué frecuencia se desinfecta las manos con desinfectante de manos mientras trabaja fuera de su residencia?	radio (Matrix)<br>1 Todo el tiempo (100%)<br>2 La mayor parte del tiempo (75%)<br>3 La mitad del tiempo (50%)<br>4 Menos de la mitad del tiempo (25%)<br>5 Nunca (0%)
701	[work33_s]<br><br>Show the field ONLY if:<br>[language] = '2' and ([work24_s] = '1' or [work24_s] = '2' or [work24_s] = '3' or [work24_s] = '4')	Actualmente, ¿cuánto le preocupa exponerse a la COVID-19 mientras trabaja fuera de su residencia?	radio (Matrix)<br>1 Todo el tiempo (100%)<br>2 La mayor parte del tiempo (75%)<br>3 La mitad del tiempo (50%)<br>4 Menos de la mitad del tiempo (25%)<br>5 Nunca (0%)
702	[work34_s]<br><br>Show the field ONLY if:<br>[language] = '2' and ([work_s] = '1' or [work_s] = '2' or [work2_s] = '1')	¿Trabaja usted actualmente en alguno de los siguientes ambientes de alto riesgo de contagio de COVID-19?	checkbox<br>1 work34_s___1 Ambiente de asistencia médica (hospital, clínica, centro de urgencias)<br>2 work34_s___2 Ambiente residencial denso (hogar de ancianos, otro centro de asistencia de larga duración)<br>3 work34_s___3 Prisión o cárcel<br>4 work34_s___4 Establecimiento de envasado de carne<br>5 work34_s___5 Establecimiento de envío o distribución<br>6 work34_s___6 Establecimiento minorista de alto volumen (tienda de provisiones, etc.)
703	[height_s]<br><br>Show the field ONLY if:<br>[language] = '2'	¿Cuánto mide usted?<br>*(Dé su estatura en pies y pulgadas (p. ej.,5'10))*	text
704	[weight_s]<br><br>Show the field ONLY if:<br>[language] = '2'	¿Cuánto pesa usted?<br>*(Dé su peso en libras (p. ej.,145))*	text
705	[insur_s]<br><br>Show the field ONLY if:<br>[language] = '2'	¿Tiene usted seguro médico o de salud (seguro privado, seguro que usted ha comprado, Medicare, Medicaid o algún otro tipo de seguro)?	radio<br>1 Sí<br>0 No<br>2 No sabe
706	[insur2_s]<br><br>Show the field ONLY if:<br>[language] = '2' and [insur_s] = '1'	¿Cuál es su principal seguro de salud?<br>*Seleccione uno (su seguro principal).*	radio<br>1 Seguro médico privado por empleo o establecimiento educacional<br>2 Seguro comprado en un intercambio de seguros de salud estatal o federal, tal como healthcare.gov

			3 Seguro comprado directamente a una campañia de seguros<br>4 Medicare<br>5 Medi-Gap<br>6 Medicaid<br>7 Asistencia médica militar (TRICARE, VA, CHAMP-VA, etc.)<br>8 Servicio de Salud Indígena<br>9 Otro
707	[insur3_s]<br>Show the field ONLY if: [language] = '2' and [insur2_s] = '9'	Si marcó "Otro", especifique la fuente de seguro médico.	text
708	[allergies_s]<br>Show the field ONLY if: [language] = '2'	Section Header: *Alguna vez le han dado un diagnostico de alguno de los siguientes?*<br>Alergias estacionales	radio (Matrix)<br>1 Sí<br>0 No
709	[asthma_s]<br>Show the field ONLY if: [language] = '2'	Asma	radio (Matrix)<br>1 Sí<br>0 No
710	[diabetes_s]<br>Show the field ONLY if: [language] = '2'	Diabetes	radio (Matrix)<br>1 Sí<br>0 No
711	[htn_s]<br>Show the field ONLY if: [language] = '2'	Hipertensión	radio (Matrix)<br>1 Sí<br>0 No
712	[cvd_s]<br>Show the field ONLY if: [language] = '2'	Enfermedad cardiovascular (ataque al corazón, insuficiencia cardíaca, angina, etc.)	radio (Matrix)<br>1 Sí<br>0 No
713	[cancer_s]<br>Show the field ONLY if: [language] = '2'	Cáncer	radio (Matrix)<br>1 Sí<br>0 No
714	[crd_s]<br>Show the field ONLY if: [language] = '2'	Enfermedad pulmonar o respiratoria crónica (COPD enfisema, bronquitis, etc.)	radio (Matrix)<br>1 Sí<br>0 No
715	[kidney_s]<br>Show the field ONLY if: [language] = '2'	Enfermedad crónica de los riñones	radio (Matrix)<br>1 Sí<br>0 No
716	[liver_s]<br>Show the field ONLY if: [language] = '2'	Enfermedad crónica del hígado (cirrosis, etc.)	radio (Matrix)<br>1 Sí<br>0 No
717	[immune_s]<br>Show the field ONLY if: [language] = '2'	Debilitamiento del sistema inmunitario (VIH, tratamiento crónico con corticosteroides, trasplante de órganos, etc.)	radio (Matrix)<br>1 Sí<br>0 No

718	[other_chronic_s]<br><br>Show the field ONLY if:<br>[language] = '2'	Otra afección crónica	radio (Matrix)<br>1 Sí<br>0 No
719	[other_chronic2_s]<br><br>Show the field ONLY if:<br>[language] = '2' and [other_chronic_s] = '1'	¿Qué otra afección crónica le han diagnosticado?	text
720	[pregnant_s]<br><br>Show the field ONLY if:<br>[language] = '2' and [sex] = '1'	¿Está usted actualmente embarazada?	radio<br>1 Sí<br>0 No
721	[prev_covid_s]<br><br>Show the field ONLY if:<br>[language] = '2'	¿Le han diagnosticado COVID-19?	radio<br>1 Sí<br>0 No<br><br>Field Annotation: @HIDDEN
722	[prev_covid2_s]<br><br>Show the field ONLY if:<br>[language] = '2'	¿Cuándo le diagnosticaron COVID-19?<br>*Si le hicieron la prueba, dé la fecha exacta o más aproximada posible. Si no le hicieron la prueba, dé la fecha exacta o más aproximada posible en que un médico le diagnosticó la COVID-19.*	text (date_mdy)<br>Field Annotation: @HIDDEN
723	[srh_s]<br><br>Show the field ONLY if:<br>[language] = '2'	En general, ¿cómo habría calificado usted su salud antes de la pandemia de COVID-19?	radio (Matrix)<br>1 Excelente<br>2 Muy buena<br>3 Buena<br>4 Regular<br>5 Mala
724	[srh2_s]<br><br>Show the field ONLY if:<br>[language] = '2'	En general, ¿cómo calificaría usted su salud en las últimas dos semanas?	radio (Matrix)<br>1 Excelente<br>2 Muy buena<br>3 Buena<br>4 Regular<br>5 Mala
725	[fluvaccine_s]<br><br>Show the field ONLY if:<br>[language] = '2'	¿Ha sido usted vacunado contra la influenza esta temporada (2019-2020)?	radio<br>1 Sí<br>0 No<br>2 No sabe
726	[fluvaccine2_s]<br><br>Show the field ONLY if:<br>[language] = '2'	¿Con qué frecuencia se vacuna usted contra la influenza?	radio<br>1 Cada temporada de influenza<br>2 La mayoria de las temporadas de influenza<br>3 La mitad de las temporadas de influenza<br>4 Menos de la mitad de las temporadas de influenza<br>5 Nunca
727	[prevent_s]<br><br>Show the field ONLY if:	Section Header: *Con que frequencia ha hecho lo siguiente desde el inicio de la pandemia de COVID-19 en Carolina del Norte para protegerse contra la infeccion?*	radio (Matrix)<br>1 Siempre (100%)

	[language] = '2'	Llevar mascarilla	2 La mayor parte del tiempo (75%)<br>3 La mitad del tiempo (50%)<br>4 Menos de la mitad del tiempo (25%)<br>5 Nunca (0%)
728	[prevent2_s]<br><br>Show the field ONLY if:<br>[language] = '2'	Lavarse las manos y/o limpiárselas con desinfectante a menudo	radio (Matrix)<br>1 Siempre (100%)<br>2 La mayor parte del tiempo (75%)<br>3 La mitad del tiempo (50%)<br>4 Menos de la mitad del tiempo (25%)<br>5 Nunca (0%)
729	[prevent3_s]<br><br>Show the field ONLY if:<br>[language] = '2'	Mantenerse al menos a 6 pies de distancia de los demas	radio (Matrix)<br>1 Siempre (100%)<br>2 La mayor parte del tiempo (75%)<br>3 La mitad del tiempo (50%)<br>4 Menos de la mitad del tiempo (25%)<br>5 Nunca (0%)
730	[prevent4_s]<br><br>Show the field ONLY if:<br>[language] = '2'	Evitar grandes reuniones, espacios públicos o multitudes	radio (Matrix)<br>1 Siempre (100%)<br>2 La mayor parte del tiempo (75%)<br>3 La mitad del tiempo (50%)<br>4 Menos de la mitad del tiempo (25%)<br>5 Nunca (0%)
731	[prevent5_s]<br><br>Show the field ONLY if:<br>[language] = '2'	Evitar el contacto con personas de alto riesgo	radio (Matrix)<br>1 Siempre (100%)<br>2 La mayor parte del tiempo (75%)<br>3 La mitad del tiempo (50%)<br>4 Menos de la mitad del tiempo (25%)<br>5 Nunca (0%)
732	[prevent6_s]<br><br>Show the field ONLY if:<br>[language] = '2'	No comer en restaurante, ni siquiera comprar comida para llevar	radio (Matrix)<br>1 Siempre (100%)<br>2 La mayor parte del tiempo (75%)<br>3 La mitad del tiempo (50%)<br>4 Menos de la mitad del tiempo (25%)<br>5 Nunca (0%)
733	[prevent7_s]<br><br>Show the field ONLY if:<br>[language] = '2'	Trabajar o estudiar en casa en lugar de ir a una oficina o un aula	radio (Matrix)<br>1 Siempre (100%)<br>2 La mayor parte del tiempo (75%)<br>3 La mitad del tiempo (50%)<br>4 Menos de la mitad del tiempo (25%)<br>5 Nunca (0%)
734	[prevent8_s]<br><br>Show the field ONLY if:	No dar la mano ni tocar a otras personas.	radio (Matrix)<br>1 Siempre (100%)

	[language] = '2'		2 La mayor parte del tiempo (75%)<br>3 La mitad del tiempo (50%)<br>4 Menos de la mitad del tiempo (25%)<br>5 Nunca (0%)
735	[prevent9_s]<br><br>Show the field ONLY if:<br>[language] = '2'	Quedarse en casa cuando se está enfermo	radio (Matrix)<br>1 Siempre (100%)<br>2 La mayor parte del tiempo (75%)<br>3 La mitad del tiempo (50%)<br>4 Menos de la mitad del tiempo (25%)<br>5 Nunca (0%)
736	[prevent10_s]<br><br>Show the field ONLY if:<br>[language] = '2'	Limpiar las superficies con desinfectante	radio (Matrix)<br>1 Siempre (100%)<br>2 La mayor parte del tiempo (75%)<br>3 La mitad del tiempo (50%)<br>4 Menos de la mitad del tiempo (25%)<br>5 Nunca (0%)
737	[prevent11_s]<br><br>Show the field ONLY if:<br>[language] = '2'	Cancelar o postergar los viajes de trabajo	radio (Matrix)<br>1 Siempre (100%)<br>2 La mayor parte del tiempo (75%)<br>3 La mitad del tiempo (50%)<br>4 Menos de la mitad del tiempo (25%)<br>5 Nunca (0%)
738	[prevent12_s]<br><br>Show the field ONLY if:<br>[language] = '2'	Cancelar o postergar los viajes de placer	radio (Matrix)<br>1 Siempre (100%)<br>2 La mayor parte del tiempo (75%)<br>3 La mitad del tiempo (50%)<br>4 Menos de la mitad del tiempo (25%)<br>5 Nunca (0%)
739	[prevent13_s]<br><br>Show the field ONLY if:<br>[language] = '2'	Cancelar o postergar las actividades personales o sociales	radio (Matrix)<br>1 Siempre (100%)<br>2 La mayor parte del tiempo (75%)<br>3 La mitad del tiempo (50%)<br>4 Menos de la mitad del tiempo (25%)<br>5 Nunca (0%)
740	[prevent14_s]<br><br>Show the field ONLY if:<br>[language] = '2'	Cancelar las consultas médicas en persona	radio (Matrix)<br>1 Siempre (100%)<br>2 La mayor parte del tiempo (75%)<br>3 La mitad del tiempo (50%)<br>4 Menos de la mitad del tiempo (25%)<br>5 Nunca (0%)
741	[prevent15_s]<br><br>Show the field ONLY if:	Almacenar comida o agua	radio (Matrix)<br>1 Siempre (100%)

	[language] = '2'		2 La mayor parte del tiempo (75%)<br>3 La mitad del tiempo (50%)<br>4 Menos de la mitad del tiempo (25%)<br>5 Nunca (0%)
742	[prevent16_s]<br><br>Show the field ONLY if:<br>[language] = '2'	Seguir las recomendaciones o reglas del gobierno sobre refugiarse donde se esté (es decir, quedarse en casa, limitar el contacto con otras personas, etc.)	radio (Matrix)<br>1 Siempre (100%)<br>2 La mayor parte del tiempo (75%)<br>3 La mitad del tiempo (50%)<br>4 Menos de la mitad del tiempo (25%)<br>5 Nunca (0%)
743	[receivedcovidvaccine_s]<br><br>Show the field ONLY if:<br>[language] = '2'	¿Ha recibido una vacuna de COVID-19 fuera de un ensayo clínico?	radio<br>1 Sí<br>0 No
744	[receivedvaccine_where_s]<br><br>Show the field ONLY if:<br>[language] = '2' and [receivedcovidvaccine_s] = '1'	¿Dónde recibió la vacuna de COVID-19?	radio<br>1 Oficina del doctor/medico<br>2 Trabajo/Empleo<br>3 Minorista (por ejemplo Walgreens, CVS)<br>5 Sitio de la vacuna<br>4 Otro
745	[receivedvaccine_where_oth_s]<br><br>Show the field ONLY if:<br>[language] = '2' and [receivedcovidvaccine_s] = '1' and [receivedvaccine_where_s] = '4'	Especifique donde recibió la vacuna de COVID-19.	text
746	[receivedvaccine_site_s]<br><br>Show the field ONLY if:<br>[language] = '2' and [receivedcovidvaccine_s] = '1' and [receivedvaccine_where_s] = '5'	Especifique la cuidad/el pueblo en Carolina del Norte del sitio de la vacuna:	text
747	[receivedvaccine_site2_s]<br><br>Show the field ONLY if:<br>[language] = '2' and [receivedcovidvaccine_s] = '1' and [receivedvaccine_where_s] = '5'	Especifique quien fue la organización o instituto que organizo el sitio de la vacuna:	text
748	[receivedvaccine_name_s]<br><br>Show the field ONLY if:<br>[language] = '2' and [receivedcovidvaccine_s] = '1'	¿Cuál vacuna de COVID-19 recibió?	radio<br>1 Pfizer<br>2 Moderna<br>3 AstraZeneca<br>4 Novavax<br>6 Johnson & Johnson

			5 Otro<br>9 No sabe
749	[receivedvaccine_name_oth_s]<br>Show the field ONLY if:<br>[language] = '2' and [receivedcovidvaccine_s] = '1' and [receivedvaccine_name_s] = '5'	Especifique que otra vacuna de COVID recibió.	text
750	[receivedvaccine_doses_s]<br>Show the field ONLY if:<br>[language] = '2' and [receivedcovidvaccine_s] = '1'	¿Cuántas dosis de la vacuna ha recibido?	radio<br>1 1<br>2 2
751	[receivedvaccine_first_dt_s]<br>Show the field ONLY if:<br>[language] = '2' and [receivedcovidvaccine_s] = '1' OR [receivedvaccine_doses_s] = '1' OR [receivedvaccine_doses_s] = '2'	¿Qué día recibió la primera dosis de la vacuna?	text (date_mdy)
752	[receivedvaccine_second_dt_s]<br>Show the field ONLY if:<br>[language] = '2' and [receivedcovidvaccine_s] = '1' and [receivedvaccine_doses_s] = '2'	¿Qué día recibió la segunda dosis de la vacuna?	text (date_mdy)
753	[vacc_pain_s]<br>Show the field ONLY if:<br>[language] = '2' and [receivedcovidvaccine_s] = '1'	Section Header: *¿Usted sintió alguno de los siguientes efectos secundarios después de la vacunación?*<br>dolor en o alrededor del lugar de la inyección	radio (Matrix)<br>0 No<br>1 Leve (nota síntomas, pero no son un problema)<br>2 Moderado (los síntomas limitan sus actividades diarias normales)<br>3 Grave (los síntomas hacen que las actividades normales sean difícil o imposible)<br>Question number: 1
754	[vacc_redness_s]<br>Show the field ONLY if:<br>[language] = '2' and [receivedcovidvaccine_s] = '1'	enrojecimiento en o alrededor del lugar de la inyección	radio (Matrix)<br>0 No<br>1 Leve (nota síntomas, pero no son un problema)<br>2 Moderado (los síntomas limitan sus actividades diarias normales)<br>3 Grave (los síntomas hacen que las actividades normales sean difícil o imposible)<br>Question number: 2
755	[vacc_swell_s]	hinchazón en o alrededor del lugar de la inyección	radio (Matrix)

			0 No<br>1 Leve (nota síntomas, pero no son un problema)<br>2 Moderado (los síntomas limitan sus actividades diarias normales)<br>3 Grave (los síntomas hacen que las actividades normales sean difícil o imposible)<br><br>Question number: 3
756	[vacc_rash_s]<br><br>Show the field ONLY if:<br>[language] = '2' and [receivedcovidvaccine_s] = '1'	erupción en o alrededor del lugar de la inyección	radio (Matrix)<br>0 No<br>1 Leve (nota síntomas, pero no son un problema)<br>2 Moderado (los síntomas limitan sus actividades diarias normales)<br>3 Grave (los síntomas hacen que las actividades normales sean difícil o imposible)<br><br>Question number: 4
757	[vacc_headache_s]<br><br>Show the field ONLY if:<br>[language] = '2' and [receivedcovidvaccine_s] = '1'	dolor de cabeza	radio (Matrix)<br>0 No<br>1 Leve (nota síntomas, pero no son un problema)<br>2 Moderado (los síntomas limitan sus actividades diarias normales)<br>3 Grave (los síntomas hacen que las actividades normales sean difícil o imposible)<br><br>Question number: 5
758	[vacc_fatigue_s]<br><br>Show the field ONLY if:<br>[language] = '2' and [receivedcovidvaccine_s] = '1'	fatiga/cansancio	radio (Matrix)<br>0 No<br>1 Leve (nota síntomas, pero no son un problema)<br>2 Moderado (los síntomas limitan sus actividades diarias normales)<br>3 Grave (los síntomas hacen que las actividades normales sean difícil o imposible)<br><br>Question number: 6
759	[vacc_fever_s]<br><br>Show the field ONLY if:<br>[language] = '2' and [receivedcovidvaccine_s] = '1'	fiebre (temperatura >100.4°F o >38°C)	radio (Matrix)<br>0 No<br>1 Leve (nota síntomas, pero no son un problema)<br>2 Moderado (los síntomas limitan sus actividades diarias normales)<br>3 Grave (los síntomas hacen que las actividades normales sean difícil o

			imposible)<br><br>Question number: 7
760	[vacc_chills_s]<br><br>Show the field ONLY if: [language] = '2' and [receivedcovidvaccine_s] = '1'	escalofríos	radio (Matrix)<br>0 No<br>1 Leve (nota síntomas, pero no son un problema)<br>2 Moderado (los síntomas limitan sus actividades diarias normales)<br>3 Grave (los síntomas hacen que las actividades normales sean difícil o imposible)<br><br>Question number: 8
761	[vacc_joint_s]<br><br>Show the field ONLY if: [language] = '2' and [receivedcovidvaccine_s] = '1'	dolor en las articulaciones	radio (Matrix)<br>0 No<br>1 Leve (nota síntomas, pero no son un problema)<br>2 Moderado (los síntomas limitan sus actividades diarias normales)<br>3 Grave (los síntomas hacen que las actividades normales sean difícil o imposible)<br><br>Question number: 9
762	[vacc_muscle_s]<br><br>Show the field ONLY if: [language] = '2' and [receivedcovidvaccine_s] = '1'	dolor muscular	radio (Matrix)<br>0 No<br>1 Leve (nota síntomas, pero no son un problema)<br>2 Moderado (los síntomas limitan sus actividades diarias normales)<br>3 Grave (los síntomas hacen que las actividades normales sean difícil o imposible)<br><br>Question number: 10
763	[vacc_nausea_s]<br><br>Show the field ONLY if: [language] = '2' and [receivedcovidvaccine_s] = '1'	nausea	radio (Matrix)<br>0 No<br>1 Leve (nota síntomas, pero no son un problema)<br>2 Moderado (los síntomas limitan sus actividades diarias normales)<br>3 Grave (los síntomas hacen que las actividades normales sean difícil o imposible)<br><br>Question number: 11
764	[vacc_effects_s]<br><br>Show the field ONLY if: [vacc_pain_s]>0 OR [vacc_redness_s]>0 OR [vacc_s	¿Cuánto tiempo duraron estos efectos secundarios?	radio<br>1 menos de 12 horas<br>2 12 a 24 horas

	well_s]>0 OR [vacc_rash_s]>0 OR [vacc_headache_s]>0 OR [vacc_fatigue_s]>0 OR [vacc_fever_s]>0 OR [vacc_chills_s]>0 OR [vacc_joint_s]>0 OR [vacc_muscle_s]>0 OR [vacc_nausea_s]>0		3 más de 24 horas
765	[vacc_med_s]<br><br>Show the field ONLY if:<br>[vacc_pain_s]>0 OR [vacc_redness_s]>0 OR [vacc_swell_s]>0 OR [vacc_rash_s]>0 OR [vacc_headache_s]>0 OR [vacc_fatigue_s]>0 OR [vacc_fever_s]>0 OR [vacc_chills_s]>0 OR [vacc_joint_s]>0 OR [vacc_muscle_s]>0 OR [vacc_nausea_s]>0	¿Tomo algún medicamento para estos efectos secundarios?	radio<br>1 Sí<br>0 No
766	[vacc_med_list_s]<br><br>Show the field ONLY if:<br>[vacc_med_s] = '1'	¿Que medicamento(s) tomo para los efectos secundarios?<br>*Por favor enumere todos los medicamentos.*	text
767	[vacc_effect_consult_s]<br><br>Show the field ONLY if:<br>[vacc_pain_s]>0 OR [vacc_redness_s]>0 OR [vacc_swell_s]>0 OR [vacc_rash_s]>0 OR [vacc_headache_s]>0 OR [vacc_fatigue_s]>0 OR [vacc_fever_s]>0 OR [vacc_chills_s]>0 OR [vacc_joint_s]>0 OR [vacc_muscle_s]>0 OR [vacc_nausea_s]>0	¿Consultó a un médico u otro proveedor de atención médica para los efectos secundarios?	radio<br>1 Sí<br>0 No
768	[vacc_effects_dose_s]<br><br>Show the field ONLY if:<br>[vacc_pain_s]>0 OR [vacc_redness_s]>0 OR [vacc_swell_s]>0 OR [vacc_rash_s]>0 OR [vacc_headache_s]>0 OR [vacc_fatigue_s]>0 OR [vacc_fever_s]>0 OR [vacc_chills_s]>0 OR [vacc_joint_s]>0 OR [vacc_muscle_s]>0 OR [vacc_nausea_s]>0 AND [receivedvaccine_dose_e(2)] = '1'	¿Como experimento los efectos secundarios después de la secunda dosis de la vacuna en comparación con los efectos secundarios después de la primera dosis de la vacuna?	radio<br>3 más grave<br>2 menos grave<br>1 igualmente grave<br>0 no corresponde/aún no he recibido la segunda dosis
769	[covidvaccine_s]<br><br>Show the field ONLY if:<br>[language] = '2' and [receivedcovidvaccine_s] = '0'	¿Piensa usted vacunarse contra la COVID-19 si se ofrece una vacuna?	radio<br>1 Sí<br>0 No<br>2 No sabe
770	[vaccine_hesitancy_s]<br><br>Show the field ONLY if:	Cuáles de las siguientes, si alguna, son razones por las que respondió "no" o "no se" acerca de recibir la vacuna de COVID-19?	checkbox<br>1 vaccine_hesitancy_s___1 Me preocupan lo secundarios y la

	[covidvaccine_s]='0' or [covidvaccine_s]='2'	*Marque todo lo que corresponda*	

		de la vacuna de
2	vaccine_hesitancy_s___2	Tengo una condi subyacente y no suficientes inves que me hagan se cómodo al recibi de COVID-19 en momento
3	vaccine_hesitancy_s___3	Me preocupa qu de COVID-19 se desarrollando de rápido
4	vaccine_hesitancy_s___4	Tengo la intenció esperar y ver si l segura y recibirl adelante
5	vaccine_hesitancy_s___5	No confió en el
6	vaccine_hesitancy_s___6	Tengo la intenció máscaras y otras precauciones en recibir la vacuna
7	vaccine_hesitancy_s___7	No soy miembro grupo que este riesgo de COVID
8	vaccine_hesitancy_s___8	No creo que CO una enfermedad
9	vaccine_hesitancy_s___9	Creo que la vac darme COVID-1
10	vaccine_hesitancy_s___10	No creo que la v COVID-19 funci
11	vaccine_hesitancy_s___11	No me gustan la
12	vaccine_hesitancy_s___12	Ya tuve COVID-1 debería ser inm
13	vaccine_hesitancy_s___13	Mi doctor no me recomendado la COVID-19
14	vaccine_hesitancy_s___14	No sabía que ne una vacuna con 19
15	vaccine_hesitancy_s___15	Me preocupan l asociados con la (como los costos visitas al consult tarifas de admin la vacuna)
16	vaccine_hesitancy_s___16	Todavía no soy (según las pauta fases en NC o d otras condiciones/pr de salud)
17	vaccine_hesitancy_s___17	No tengo acceso
18	vaccine_hesitancy_s___18	Otra cosa

771	[vaccine_hesitancy_oth_s]<br><br>Show the field ONLY if:<br>[vaccine_hesitancy_s(18)] = '1'	Especifique que otro motivo tiene usted para o no recibir la vacuna de COVID-19	text
772	[fever_s]<br><br>Show the field ONLY if:<br>[language] = '2'	Section Header: *Durante los ultimos dos semanas, ha tenido alguno de los siguientes sintomas?*<br><br>Fiebre (medida por termómetro o autodiagnóstico)	radio (Matrix)<br>1 Sí<br>0 No
773	[cough_s]<br><br>Show the field ONLY if:<br>[language] = '2'	Tos (nueva o que empeora)	radio (Matrix)<br>1 Sí<br>0 No
774	[sob_s]<br><br>Show the field ONLY if:<br>[language] = '2'	Dificultad para respirar (nueva o que empeora)	radio (Matrix)<br>1 Sí<br>0 No
775	[fatigue_s]<br><br>Show the field ONLY if:<br>[language] = '2'	Fatiga (nuevo cansancio al hacer actividades normales)	radio (Matrix)<br>1 Sí<br>0 No
776	[bodyache_s]<br><br>Show the field ONLY if:<br>[language] = '2'	Dolores de cuerpo	radio (Matrix)<br>1 Sí<br>0 No
777	[headache_s]<br><br>Show the field ONLY if:<br>[language] = '2'	Dolor de cabeza	radio (Matrix)<br>1 Sí<br>0 No
778	[diarrhea_s]<br><br>Show the field ONLY if:<br>[language] = '2'	Diarrea	radio (Matrix)<br>1 Sí<br>0 No
779	[pharyngitis_s]<br><br>Show the field ONLY if:<br>[language] = '2'	Dolor de garganta	radio (Matrix)<br>1 Sí<br>0 No
780	[eye_s]<br><br>Show the field ONLY if:<br>[language] = '2'	Picazón, enrojecimiento o dolor de ojos	radio (Matrix)<br>1 Sí<br>0 No
781	[congest_s]<br><br>Show the field ONLY if:<br>[language] = '2'	Goteo o congestión nasal	radio (Matrix)<br>1 Sí<br>0 No
782	[sensory_s]<br><br>Show the field ONLY if:<br>[language] = '2'	Cambios en el sentido del olfato o del gusto	radio (Matrix)<br>1 Sí<br>0 No
783	[rash_s]<br><br>Show the field ONLY if:<br>[language] = '2'	Nuevo sarpullido	radio (Matrix)<br>1 Sí<br>0 No
784	[chills_s]	Temblores y escalofríos reiterados	radio (Matrix)

	Show the field ONLY if: [language] = '2'		1 Sí<br>0 No
785	[covid_symp_s]<br><br>Show the field ONLY if: [language] = '2' and ([fever_s] = '1' or [cough_s] = '1' or [sob_s] = '1' or [fatigue_s] = '1' or [bodyache_s] = '1' or [headache_s] = '1' or [diarrhea_s] = '1' or [pharyngitis_s] = '1' or [eye_s] = '1' or [congest_s] = '1' or [sensory_s] = '1' or [rash_s] = '1' or [chills_s] = '1')	¿Cuándo comenzaron estos síntomas?	text (date_mdy)
786	[covid_symp2_s]<br><br>Show the field ONLY if: [language] = '2' and ([fever_s] = '1' or [cough_s] = '1' or [sob_s] = '1' or [fatigue_s] = '1' or [bodyache_s] = '1' or [headache_s] = '1' or [diarrhea_s] = '1' or [pharyngitis_s] = '1' or [eye_s] = '1' or [congest_s] = '1' or [sensory_s] = '1' or [rash_s] = '1' or [chills_s] = '1')	En vista de los síntomas que declaró, ¿le preocupaba a usted la posibilidad de tener COVID-19?	radio<br>1 Sí<br>0 No<br>2 No sabe
787	[covid_symp3_s]<br><br>Show the field ONLY if: [language] = '2' and ([fever_s] = '1' or [cough_s] = '1' or [sob_s] = '1' or [fatigue_s] = '1' or [bodyache_s] = '1' or [headache_s] = '1' or [diarrhea_s] = '1' or [pharyngitis_s] = '1' or [eye_s] = '1' or [congest_s] = '1' or [sensory_s] = '1' or [rash_s] = '1' or [chills_s] = '1')	¿Fue usted objeto de prejuicio o discriminación debido a sus síntomas?	radio<br>1 Sí<br>0 No<br>2 No sabe
788	[prevent17_s]<br><br>Show the field ONLY if: [language] = '2' and ([fever_s] = '1' or [cough_s] = '1' or [sob_s] = '1' or [fatigue_s] = '1' or [bodyache_s] = '1' or [headache_s] = '1' or [diarrhea_s] = '1' or [pharyngitis_s] = '1' or [eye_s] = '1' or [congest_s] = '1' or [sensory_s] = '1' or [rash_s] = '1' or [chills_s] = '1')	¿Cuáles de las siguientes medidas tomó usted para proteger a sus amigos y familiares después de que comenzaron sus síntomas?	checkbox<br>1 prevent17_s___1 Ponerse mascarilla con mayor frecuencia<br>2 prevent17_s___2 Lavarse las manos con agua y jabón con mayor frecuencia<br>3 prevent17_s___3 Limpiarse las manos con desinfectante con mayor frecuencia<br>4 prevent17_s___4 Aislarse en su casa con mayor frecuencia<br>5 prevent17_s___5 Quedarse en su casa con mayor frecuencia<br>6 prevent17_s___6 Ponerse guantes desechables con mayor frecuencia

789	[covid_symp4_s]<br><br>Show the field ONLY if:<br>[language] = '2' and ([fever_s] = '1' or [cough_s] = '1' or [sob_s] = '1' or [fatigue_s] = '1' or [bodyache_s] = '1' or [headache_s] = '1' or [diarrhea_s] = '1' or [pharyngitis_s] = '1' or [eye_s] = '1' or [congest_s] = '1' or [sensory_s] = '1' or [rash_s] = '1' or [chills_s] = '1')	¿Qué hizo usted en vista de sus síntomas?<br>*Seleccione todo lo que corresponda.*	checkbox<br>0 covid_symp4_s___0 Nada<br>1 covid_symp4_s___1 Tomó medicamentos sin receta (ibuprofeno, acetaminofén, etc.)<br>2 covid_symp4_s___2 Consultó por teléfono a un proveedor de asistencia médica<br>3 covid_symp4_s___3 Fue al consultorio de un proveedor de atencion médica<br>4 covid_symp4_s___4 Fue a una clínica o una farmacia minorista<br>5 covid_symp4_s___5 Fue a un centro de urgencias (FASTMed, etc.)<br>6 covid_symp4_s___6 Fue a la sala de emergencias<br>7 covid_symp4_s___7 Fue ingresado al hospital<br>8 covid_symp4_s___8 Otra cosa<br>9 covid_symp4_s___9 No sabe
790	[covid_symp5_s]<br><br>Show the field ONLY if:<br>[language] = '2' and [covid_symp4_s(8)] = '1'	¿Qué otra cosa hizo usted en vista de sus síntomas?	text
791	[covid_symp6_s]<br><br>Show the field ONLY if:<br>[language] = '2' and ([covid_symp4_s(2)] = '1' or [covid_symp4_s(3)] = '1' or [covid_symp4_s(4)] = '1' or [covid_symp4_s(5)] = '1' or [covid_symp4_s(6)] = '1' or [covid_symp4_s(7)] = '1' or [covid_symp4_s(8)] = '1')	Si pudo hablar con un proveedor de asistencia médica, ¿le dijo el proveedor que era posible que tuviera COVID-19?	radio<br>1 Sí<br>0 No<br>2 No sabe
792	[covid_test_s]<br><br>Show the field ONLY if:<br>[language] = '2' and ([fever_s] = '1' or [cough_s] = '1' or [sob_s] = '1' or [fatigue_s] = '1' or [bodyache_s] = '1' or [headache_s] = '1' or [diarrhea_s] = '1' or [pharyngitis_s] = '1' or [eye_s] = '1' or [congest_s] = '1' or [sensory_s] = '1' or [rash_s] = '1' or [chills_s] = '1')	Si le hicieron una prueba de COVID-19 en vista de los síntomas que declaró, ¿cuál fue el resultado?	radio<br>1 Pendiente<br>2 Positivo<br>3 Negativo<br>4 No concluyente<br>5 No se hizo la prueba
793	[covid_admit_s]	¿Cuántos días estuvo usted hospitalizado?	text (number, Min: 0)

	Show the field ONLY if: [language] = '2' and [covid_symp4_s(7)] = '1'		
794	[covid_admit2_s]<br><br>Show the field ONLY if: [language] = '2' and [covid_symp4_s(7)] = '1'	¿Le hicieron a usted las siguientes intervenciones durante su hospitalización?	checkbox<br>1 covid_admit2_s___1 Oxígeno adicional por la nariz<br>2 covid_admit2_s___2 Tratamiento en la Unidad de Cuidados Intensivos (Intensive Care Unit, ICU)<br>3 covid_admit2_s___3 Ventilación mecánica (intubación o tubo de respiración)
795	[covid_symp7_s]<br><br>Show the field ONLY if: [language] = '2' and ([fever_s] = '1' or [cough_s] = '1' or [sob_s] = '1' or [fatigue_s] = '1' or [bodyache_s] = '1' or [headache_s] = '1' or [diarrhea_s] = '1' or [pharyngitis_s] = '1' or [eye_s] = '1' or [congest_s] = '1' or [sensory_s] = '1' or [rash_s] = '1' or [chills_s] = '1')	¿Ha vuelto usted a su salud normal?	radio<br>1 Sí<br>0 No<br>2 No sabe
796	[address_s]<br><br>Show the field ONLY if: [language] = '2'	Section Header: *Proporcione la siguiente informacion sobre su hogar.*<br><br>¿Cuál es su dirección permanente?	text
797	[address2_s]<br><br>Show the field ONLY if: [language] = '2'	¿Cuanto tiempo ha vivido en esta dirección?	radio<br>1 0-3 años<br>2 4-6 años<br>3 7-10 años<br>4 Más de 10 años
798	[hhcount_s]<br><br>Show the field ONLY if: [language] = '2'	¿Cuántas personas (sin contarse usted) viven o pasan una cantidad considerable de tiempo en esta vivienda (más de 40 horas por semana)?	radio<br>0 0<br>1 1<br>2 2<br>3 3<br>4 4<br>5 5<br>6 6<br>7 7<br>8 8<br>9 9<br>10 10<br>11 11<br>12 12

799	[hhcount2_s]<br><br>Show the field ONLY if:<br>[language] = '2' and [hhcount_s] > 0	¿Cuántas personas de su grupo familiar son menores de 18 años?	radio<br>0 0<br>1 1<br>2 2<br>3 3<br>4 4<br>5 5<br>6 6<br>7 7<br>8 8<br>9 9<br>10 10<br>11 11<br>12 12
800	[hhcount3_s]<br><br>Show the field ONLY if:<br>[language] = '2' and [hhcount2_s] > 0	¿Qué tan difícil ha sido para su grupo familiar adaptarse a los cambios en el cuidado infantil o la educación de los niños en casa?	radio<br>1 Nada dificil<br>2 Un poco dificil<br>3 Muy dificil<br>4 Extremadamente dificil
801	[hhlang_s]<br><br>Show the field ONLY if:<br>[language] = '2'	¿Cuál es el idioma principal que se habla en su grupo familiar?	radio<br>1 Inglés<br>2 Español<br>3 Otro
802	[hhlang2_s]<br><br>Show the field ONLY if:<br>[language] = '2' and [hhlang_s] = '3'	¿Qué otro idioma principal se habla en su grupo familiar?	text
803	[caregive_s]<br><br>Show the field ONLY if:<br>[language] = '2'	Antes de la pandemia de COVID-19, ¿tenía usted responsabilidades habituales de cuidado de una persona que no vivía con su grupo familiar (por ejemplo, padre o hermano de edad a quien usted visitaba y apoyaba con regularidad, etc.)?	radio<br>1 Sí<br>0 No
804	[caregive2_s]<br><br>Show the field ONLY if:<br>[language] = '2' and [caregive_s] = '1'	¿Qué tan difícil ha sido continuar con sus responsabilidades de cuidado debido a la pandemia de COVID-19?	radio<br>1 Nada dificil<br>2 Un poco dificil<br>3 Muy dificil<br>4 Extremadamente dificil
805	[isolate_s]<br><br>Show the field ONLY if:<br>[language] = '2' and [hhcount_s] > 0	Si a un integrante del grupo familiar le da COVID-19, ¿qué tan bien podría el grupo familiar aislarlo de otros integrantes del grupo familiar (asignarle su propia habitación y limitar el contacto con el enfermo)?	radio<br>1 No muy bien<br>2 Bastante bien<br>3 Muy bien<br>4 Extremadamente bien
806	[income_s]<br><br>Show the field ONLY if:	Antes de descontar los impuestos, ¿cuál fue el monto total aproximado de todas las fuentes de ingresos del	radio<br>1 Menos que $10,000

	[language] = '2'	grupo familiar el año pasado?	2 $10,000-$19,999<br>3 $20,000-$29,999<br>4 $30,000-$49,999<br>5 $50,000-$74,999<br>6 $75,000 o más
807	[income2_s]<br>Show the field ONLY if:<br>[language] = '2'	En su opinión, ¿qué efecto va a tener este año la crisis de COVID-19 en el monto total de ingresos de su grupo familiar?	radio<br>1 Disminución considerable<br>2 Disminución ligera<br>3 Ningún efecto<br>4 Aumento considerable<br>5 Aumento ligero
808	[runout_s]<br>Show the field ONLY if:<br>[language] = '2'	En su opinión ¿qué tan probable es que su grupo familiar se quede sin dinero en los próximos 3 meses? Dé su respuesta en una escala de 0 (definitivamente no va a suceder) a 10 (definitivamente va a suceder).	text (number, Min: 1, Max: 10)
809	[errands_s]<br>Show the field ONLY if:<br>[language] = '2'	¿Con qué frecuencia recibe usted o su grupo familiar asistencia para hacer mandados tales como comprar provisiones o medicamentos?	radio<br>1 Todo el tiempo (100%)<br>2 La mayor parte del tiempo (75%)<br>3 La mitad del tiempo (50%)<br>4 Menos de la mitad del tiempo (25%)<br>5 Nunca (0%)
810	[isolate2_s]<br>Show the field ONLY if:<br>[language] = '2'	¿Con qué frecuencia se ha quedado usted o su familia en su casa y ha evitado interactuar con otras personas, exceptuando las salidas para comprar provisiones?	radio<br>1 Todo el tiempo (100%)<br>2 La mayor parte del tiempo (75%)<br>3 La mitad del tiempo (50%)<br>4 Menos de la mitad del tiempo (25%)<br>5 Nunca (0%)
811	[hh1_relationship_s]<br>Show the field ONLY if:<br>[language] = '2' and [hhcount_s] > 0 and [hhcount_s] < 13	Section Header: *Por cada persona adicional en su hogar, proporcione la siguiente informacion.*<br>Persona 1: ¿Cuál es su relación con esta persona?	radio<br>1 Pareja o cónyuge<br>2 Hijo o hija<br>3 Padre o madre<br>4 Hermano o hermana<br>5 Otra familiar<br>6 Proveedor de cuidado infantil u otros cuidados en casa<br>7 Otra
812	[hh1_relationship2_s]<br>Show the field ONLY if:<br>[language] = '2' and [hh1_relationship_s] = '7'	Persona 1: Especifique su relación con esta persona.	text

813	[hh1_age_s]<br><br>Show the field ONLY if:<br>[language] = '2' and [hhcount_s] > 0 and [hhcount_s] < 13	Persona 1: ¿Qué edad tiene esta persona?<br>*(Especifique la edad en años)*	text (number, Min: 0, Max: 110)
814	[hh1_sex_s]<br><br>Show the field ONLY if:<br>[language] = '2' and [hhcount_s] > 0 and [hhcount_s] < 13	Persona 1: ¿Cuál es el sexo de esta persona?	radio<br>1 Femenino<br>2 Masculino
815	[hh1_race_s]<br><br>Show the field ONLY if:<br>[language] = '2' and [hhcount_s] > 0 and [hhcount_s] < 13	Persona 1: ¿Cuál es la raza de esta persona?<br>*(Seleccione todo lo que corresponda.)*	checkbox<br>1 hh1_race_s___1 Indio americano o nativo de Alaska<br>2 hh1_race_s___2 Asiático<br>3 hh1_race_s___3 Negro o afroestadounidense<br>4 hh1_race_s___4 Nativo de Hawái o de las islas del Pacífico<br>5 hh1_race_s___5 Blanco<br>6 hh1_race_s___6 Otro<br>7 hh1_race_s___7 No sabe
816	[hh1_ethn_s]<br><br>Show the field ONLY if:<br>[language] = '2' and [hhcount_s] > 0 and [hhcount_s] < 13	Persona 1: ¿Cuál es la identidad étnica de esta persona?	radio<br>1 Hispano o latino<br>2 Ni hispano ni latino<br>3 Otra<br>4 No sabe
817	[hh1_edu_s]<br><br>Show the field ONLY if:<br>[language] = '2' and [hhcount_s] > 0 and [hhcount_s] < 13	Persona 1: ¿Cuál es el nivel de educación de esta persona?	radio<br>1 No tiene educación formal<br>2 Kínder a 8° grado<br>3 Estudios de educación secundaria<br>4 Equivalencia de educación secundaria (GED)<br>5 Diploma de educación secundaria<br>6 Estudios de educación superior<br>7 Título universitario<br>8 Estudios de posgrado o más<br>9 No sabe
818	[hh1_work_s]<br><br>Show the field ONLY if:<br>[language] = '2' and [hhcount_s] > 0 and [hhcount_s] < 13	Persona 1: ¿Cuál de las siguientes es la mejor descripción de la situación laboral actual de esta persona?	radio<br>1 Empleo a tiempo completo<br>2 Empleo a tiempo parcial<br>3 En busca de empleo<br>4 Jubilado<br>5 Ama de casa<br>6 Estudiante<br>7 Permiso de maternidad o paternidad

			8 Permiso por enfermedad<br>9 Desempleado por discapacidad<br>10 Otra<br>11 No sabe
819	[hh1_work2_s]<br><br>Show the field ONLY if:<br>[language] = '2' and [hhcount_s] > 0 and [hhcount_s] < 13	Persona 1: ¿Se considera esta persona actualmente empleado por cuenta propia (contratista independiente, trabajador esporádico (gig), etc.)?	radio<br>1 Sí<br>0 No<br>2 No sabe
820	[hh1_work3_s]<br><br>Show the field ONLY if:<br>[language] = '2' and [hhcount_s] > 0 and [hhcount_s] < 13 and ([hh1_work_s] = '1' or [hh1_work_s] = '2' or [hh1_work2_s] = '1')	Persona 1: ¿Trabaja esta persona actualmente en alguno de los siguientes ambientes de alto riesgo de contagio de COVID-19?	checkbox<br>1 hh1_work3_s___1 Ambiente de asistencia médica (hospital, clínica, centro de urgencias, etc.)<br>2 hh1_work3_s___2 Ambiente residencial denso (hogar de ancianos, otro centro de asistencia de larga duración)<br>3 hh1_work3_s___3 Prisión o cárcel<br>4 hh1_work3_s___4 Establecimiento de envasado de carne<br>5 hh1_work3_s___5 Establecimiento de envío o distribución<br>6 hh1_work3_s___6 Establecimiento minorista de alto volumen (tienda de provisiones, etc.)<br>7 hh1_work3_s___7 No sabe
821	[hh1_work4_s]<br><br>Show the field ONLY if:<br>[language] = '2' and [hhcount_s] > 0 and [hhcount_s] < 13 and ([hh1_work_s] = '1' or [hh1_work_s] = '2' or [hh1_work2_s] = '1')	Persona 1: ¿Le ofrece el empleador a esta persona alguno de los siguientes beneficios en su empleo principal actual?<br>*(Seleccione todo lo que corresponda.)*	checkbox<br>1 hh1_work4_s___1 Permiso por enfermedad con goce de sueldo<br>2 hh1_work4_s___2 Vacaciones o permiso personal con goce de sueldo<br>3 hh1_work4_s___3 Seguro de salud<br>4 hh1_work4_s___4 Seguro de discapacidad<br>5 hh1_work4_s___5 Plan de jubilación<br>6 hh1_work4_s___6 Otro<br>7 hh1_work4_s___7 No sabe
822	[hh1_work5_s]<br><br>Show the field ONLY if:<br>[language] = '2' and [hhcount_s] > 0 and [hhcount_s] < 13 and ([hh1_work_s] = '1' or [hh1_work_s] = '2' or [hh1_work2_s] = '1')	Persona 1: En una escala de 0 (definitivamente no va a suceder) a 10 (definitivamente va a suceder), ¿qué tan probable es que esta persona pierda su empleo debido a la pandemia de COVID-19?	text (number, Min: 0, Max: 10)

823	[hh1_work6_s]<br><br>Show the field ONLY if:<br>[language] = '2' and [hhcount_s] > 0 and [hhcount_s] < 13 and ([hh1_work_s] = '1' or [hh1_work_s] = '2' or [hh1_work2_s] = '1')	Persona 1: En una escala de 0 (definitivamente no va a suceder) a 10 (definitivamente va a suceder), ¿qué tan probable es que a esta persona se le asignen menos horas de trabajo debido a la pandemia de COVID-19?	text (number, Min: 0, Max: 10)
824	[hh1_work7_s]<br><br>Show the field ONLY if:<br>[language] = '2' and [hhcount_s] > 0 and [hhcount_s] < 13 and ([hh1_work_s] = '1' or [hh1_work_s] = '2' or [hh1_work2_s] = '1')	Persona 1: Actualmente ¿con qué frecuencia se le exige a esta persona que trabaje fuera de su residencia?	radio (Matrix)<br>1 Todo el tiempo (100%)<br>2 la mayor parte del tiempo (75%)<br>3 La mitad del tiempo (50%)<br>4 Menos de la mitad del tiempo (25%)<br>5 Nunca (0%)
825	[hh1_work8_s]<br><br>Show the field ONLY if:<br>[language] = '2' and ([hh1_work7_s] = '1' or [hh1_work7_s] = '2' or [hh1_work7_s] = '3' or [hh1_work7_s] = '4')	Persona 1: Actualmente ¿con qué frecuencia se encuentra esta persona físicamente cerca de sus compañeros de trabajo mientras trabaja fuera de su residencia?	radio (Matrix)<br>1 Todo el tiempo (100%)<br>2 la mayor parte del tiempo (75%)<br>3 La mitad del tiempo (50%)<br>4 Menos de la mitad del tiempo (25%)<br>5 Nunca (0%)
826	[hh1_work9_s]<br><br>Show the field ONLY if:<br>[language] = '2' and ([hh1_work7_s] = '1' or [hh1_work7_s] = '2' or [hh1_work7_s] = '3' or [hh1_work7_s] = '4')	Persona 1: Actualmente ¿con qué frecuencia se encuentra esta persona físicamente cerca de sus clientes mientras trabaja fuera de su residencia?	radio (Matrix)<br>1 Todo el tiempo (100%)<br>2 la mayor parte del tiempo (75%)<br>3 La mitad del tiempo (50%)<br>4 Menos de la mitad del tiempo (25%)<br>5 Nunca (0%)
827	[hh1_covidvaccine_s]<br><br>Show the field ONLY if:<br>[language] = '2' and [hhcount_s] > 0 and [hhcount_s] < 13	Persona 1: ¿Piensa esta persona vacunarse contra la COVID-19 cuando se ofrezca una vacuna?	radio<br>1 Sí<br>0 No<br>2 No sabe
828	[hh1_covidsymp_s]<br><br>Show the field ONLY if:<br>[language] = '2' and [hhcount_s] > 0 and [hhcount_s] < 13	Persona 1: En las últimas dos semanas, ¿ha tenido esta persona algún síntoma de COVID-19 (tos, fiebre, dificultad para respirar, fatiga, dolores de cuerpo, diarrea, goteo nasal o pérdida del sentido del olfato o del gusto)?	radio<br>1 Sí<br>0 No<br>2 No sabe
829	[hh1_covidsymp2_s]<br><br>Show the field ONLY if:<br>[language] = '2' and [hh1_covidsymp_s] = '1'	Persona 1: ¿Cuándo le comenzaron los síntomas de COVID-19 a esta persona?	text (date_mdy)
830	[hh1_covidsymp3_s]<br><br>Show the field ONLY if:<br>[language] = '2' and [hh1_covidsymp_s] = '1'	Persona 1: En vista de sus síntomas, ¿le preocupa a esta persona la posibilidad de tener COVID-19?	radio<br>1 Sí<br>0 No<br>2 No sabe

831	[hh1_covidsymp4_s]<br>Show the field ONLY if:<br>[language] = '2' and [hh1_covidsymp_s] = '1'	Persona 1: ¿Fue esta persona objeto de prejuicio o discriminación debido a sus síntomas?	radio<br>1 Sí<br>0 No<br>2 No sabe
832	[hh1_covidsymp5_s]<br>Show the field ONLY if:<br>[language] = '2' and [hh1_covidsymp_s] = '1'	Persona 1: ¿Qué hizo esta persona en vista de sus síntomas?<br>*(Seleccione todo lo que corresponda.)*	checkbox<br>0 hh1_covidsymp5_s___0 Nada<br>1 hh1_covidsymp5_s___1 Tomó medicamentos sin receta (ibuprofeno, acetaminofén, etc.)<br>2 hh1_covidsymp5_s___2 Consultó por teléfono a un proveedor de asistencia médica<br>3 hh1_covidsymp5_s___3 Fue al consultorio de un proveedor de asistencia médica<br>4 hh1_covidsymp5_s___4 Fue a una clínica o una farmacia minorista<br>5 hh1_covidsymp5_s___5 Fue a un centro de urgencias (FASTMed, etc.)<br>6 hh1_covidsymp5_s___6 Fue a la sala de emergencias<br>7 hh1_covidsymp5_s___7 Fue ingresado al hospital<br>8 hh1_covidsymp5_s___8 Otra cosa<br>9 hh1_covidsymp5_s___9 No sabe
833	[hh1_covidsymp6_s]<br>Show the field ONLY if:<br>[language] = '2' and [hh1_covidsymp5_s(8)] = '1'	Persona 1: ¿Qué otra cosa hizo esta persona en vista de sus síntomas?	text
834	[hh1_covidsymp7_s]<br>Show the field ONLY if:<br>[language] = '2' and ([hh1_covidsymp5_s(2)] = '1' or [hh1_covidsymp5_s(3)] = '1' or [hh1_covidsymp5_s(4)] = '1' or [hh1_covidsymp5_s(5)] = '1' or [hh1_covidsymp5_s(6)] = '1' or [hh1_covidsymp5_s(7)] = '1' or [hh1_covidsymp5_s(8)] = '1')	Persona 1: ¿Le dijo un proveedor de asistencia médica a esta persona que era posible que tuviera COVID-19?	radio<br>1 Sí<br>0 No<br>2 No sabe
835	[hh1_covid_test_s]<br>Show the field ONLY if:	Persona 1: Si a esta persona le hicieron una prueba de COVID-19 en vista de sus síntomas, ¿cuál fue el resultado?	radio<br>1 Pendiente

	[language] = '2' and [hh1_covidsymp_s] = '1'		2 Positivo<br>3 Negativo<br>4 No concluyente<br>5 No se hizo la prueba<br>6 No sabe
836	[hh1_covid_admit_s]<br><br>Show the field ONLY if: [language] = '2' and [hh1_covidsymp5_s(7)] = '1'	Persona 1: ¿Cuántos días estuvo hospitalizada esta persona?	text (number, Min: 0)
837	[hh1_covid_admit2_s]<br><br>Show the field ONLY if: [language] = '2' and [hh1_covidsymp5_s(7)] = '1'	Persona 1: ¿Se le hicieron a esta persona las siguientes intervenciones durante su hospitalización?	checkbox<br>1 hh1_covid_admit2_s___1 Oxígeno adicional por la nariz<br>2 hh1_covid_admit2_s___2 Tratamiento en la Unidad de Cuidados Intensivos (Intensive Care Unit, ICU)<br>3 hh1_covid_admit2_s___3 Ventilación mecánica (intubación o tubo de respiración)<br>4 hh1_covid_admit2_s___4 No sabe
838	[hh1_covidsymp8_s]<br><br>Show the field ONLY if: [language] = '2' and [hh1_covidsymp_s] = '1'	Persona 1: ¿Ha vuelto esta persona a su salud normal?	radio<br>1 Sí<br>0 No<br>2 No sabe

839	[hh1_prevent_s]<br>Show the field ONLY if:<br>[language] = '2' and [hh1_covidsymp_s] = '1'	Persona 1: ¿Cuáles de las siguientes medidas tomó esta persona para proteger a sus amigos y familiares después de que comenzaron sus síntomas?	checkbox<br>1 hh1_prevent_s___1 Ponerse mascarilla con mayor frecuencia<br>2 hh1_prevent_s___2 Lavarse las manos con agua y jabón con mayor frecuencia<br>3 hh1_prevent_s___3 Limpiarse las manos con desinfectante con mayor frecuencia<br>4 hh1_prevent_s___4 Aislarse en su casa con mayor frecuencia<br>5 hh1_prevent_s___5 Quedarse en casa con mayor frecuencia<br>6 hh1_prevent_s___6 Ponerse guantes desechables con mayor frecuencia<br>7 hh1_prevent_s___7 No sabe
840	[hh2_relationship_s]<br>Show the field ONLY if:<br>[language] = '2' and [hhcount_s] > 1 and [hhcount_s] < 13	Section Header: *Por cada persona adicional en su hogar, proporcione la siguiente informacion.*<br>Persona 2: ¿Cuál es su relación con esta persona?	radio<br>1 Pareja o cónyuge<br>2 Hijo o hija<br>3 Padre o madre<br>4 Hermano o hermana<br>5 Otra familiar<br>6 Proveedor de cuidado infantil u otros cuidados en casa<br>7 Otra
841	[hh2_relationship2_s]<br>Show the field ONLY if:<br>[language] = '2' and [hh2_relationship_s] = '7'	Persona 2: Especifique su relación con esta persona.	text
842	[hh2_age_s]<br>Show the field ONLY if:<br>[language] = '2' and [hhcount_s] > 1 and [hhcount_s] < 13	Persona 2: ¿Qué edad tiene esta persona?<br>*(Especifique la edad en años)*	text (number, Min: 0, Max: 110)
843	[hh2_sex_s]<br>Show the field ONLY if:<br>[language] = '2' and [hhcount_s] > 1 and [hhcount_s] < 13	Persona 2: ¿Cuál es el sexo de esta persona?	radio<br>1 Femenino<br>2 Masculino
844	[hh2_race_s]<br>Show the field ONLY if:<br>[language] = '2' and [hhcount_s] > 1 and [hhcount_s] < 13	Persona 2: ¿Cuál es la raza de esta persona?<br>*(Seleccione todo lo que corresponda.)*	checkbox<br>1 hh2_race_s___1 Indio americano o nativo de Alaska<br>2 hh2_race_s___2 Asiático

			3 hh2_race_s___3 Negro o afroestadounidense<br>4 hh2_race_s___4 Nativo de Hawái o de las islas del Pacífico<br>5 hh2_race_s___5 Blanco<br>6 hh2_race_s___6 Otro<br>7 hh2_race_s___7 No sabe
845	[hh2_ethn_s]<br><br>Show the field ONLY if:<br>[language] = '2' and [hhcount_s] > 1 and [hhcount_s] < 13	Persona 2: ¿Cuál es la identidad étnica de esta persona?	radio<br>1 Hispano o latino<br>2 Ni hispano ni latino<br>3 Otra<br>4 No sabe
846	[hh2_edu_s]<br><br>Show the field ONLY if:<br>[language] = '2' and [hhcount_s] > 1 and [hhcount_s] < 13	Persona 2: ¿Cuál es el nivel de educación de esta persona?	radio<br>1 No tiene educación formal<br>2 Kínder a 8° grado<br>3 Estudios de educación secundaria<br>4 Equivalencia de educación secundaria (GED)<br>5 Diploma de educación secundaria<br>6 Estudios de educación superior<br>7 Título universitario<br>8 Estudios de posgrado o más<br>9 No sabe
847	[hh2_work_s]<br><br>Show the field ONLY if:<br>[language] = '2' and [hhcount_s] > 1 and [hhcount_s] < 13	Persona 2: ¿Cuál de las siguientes es la mejor descripción de la situación laboral actual de esta persona?	radio<br>1 Empleo a tiempo completo<br>2 Empleo a tiempo parcial<br>3 En busca de empleo<br>4 Jubilado<br>5 Ama de casa<br>6 Estudiante<br>7 Permiso de maternidad o paternidad<br>8 Permiso por enfermedad<br>9 Desempleado por discapacidad<br>10 Otra<br>11 No sabe
848	[hh2_work2_s]<br><br>Show the field ONLY if:<br>[language] = '2' and [hhcount_s] > 1 and [hhcount_s] < 13	Persona 2: ¿Se considera esta persona actualmente empleado por cuenta propia (contratista independiente, trabajador esporádico (gig), etc.)?	radio<br>1 Sí<br>0 No<br>2 No sabe
849	[hh2_work3_s]<br><br>Show the field ONLY if:<br>[language] = '2' and [hhcount_s] > 1 and [hhcount_s] < 13 and ([hh2_work_s]	Persona 2: ¿Trabaja esta persona actualmente en alguno de los siguientes ambientes de alto riesgo de contagio de COVID-19?	checkbox<br>1 hh2_work3_s___1 Ambiente de asistencia médica (hospital, clínica,

	= '1' or [hh2_work_s] = '2' or [hh2_work2_s] = '1')		1 | … | centro de urgencias, etc.)<br>2 | hh2_work3_s___2 | Ambiente residencial denso (hogar de ancianos, otro centro de asistencia de larga duración)<br>3 | hh2_work3_s___3 | Prisión o cárcel<br>4 | hh2_work3_s___4 | Establecimiento de envasado de carne<br>5 | hh2_work3_s___5 | Establecimiento de envío o distribución<br>6 | hh2_work3_s___6 | Establecimiento minorista de alto volumen (tienda de provisiones, etc.)<br>7 | hh2_work3_s___7 | No sabe
850	[hh2_work4_s]<br><br>Show the field ONLY if: [language] = '2' and [hhcount_s] > 1 and [hhcount_s] < 13 and ([hh2_work_s] = '1' or [hh2_work_s] = '2' or [hh2_work2_s] = '1')	Persona 2: ¿Le ofrece el empleador a esta persona alguno de los siguientes beneficios en su empleo principal actual?<br>*(Seleccione todo lo que corresponda.)*	checkbox<br>1 | hh2_work4_s___1 | Permiso por enfermedad con goce de sueldo<br>2 | hh2_work4_s___2 | Vacaciones o permiso personal con goce de sueldo<br>3 | hh2_work4_s___3 | Seguro de salud<br>4 | hh2_work4_s___4 | Seguro de discapacidad<br>5 | hh2_work4_s___5 | Plan de jubilación<br>6 | hh2_work4_s___6 | Otro<br>7 | hh2_work4_s___7 | No sabe
851	[hh2_work5_s]<br><br>Show the field ONLY if: [language] = '2' and [hhcount_s] > 1 and [hhcount_s] < 13 and ([hh2_work_s] = '1' or [hh2_work_s] = '2' or [hh2_work2_s] = '1')	Persona 2: En una escala de 0 (definitivamente no va a suceder) a 10 (definitivamente va a suceder), ¿qué tan probable es que esta persona pierda su empleo debido a la pandemia de COVID-19?	text (number, Min: 0, Max: 10)
852	[hh2_work6_s]<br><br>Show the field ONLY if: [language] = '2' and [hhcount_s] > 1 and [hhcount_s] < 13 and ([hh2_work_s] = '1' or [hh2_work_s] = '2' or [hh2_work2_s] = '1')	Persona 2: En una escala de 0 (definitivamente no va a suceder) a 10 (definitivamente va a suceder), ¿qué tan probable es que a esta persona se le asignen menos horas de trabajo debido a la pandemia de COVID-19?	text (number, Min: 0, Max: 10)
853	[hh2_work7_s]<br><br>Show the field ONLY if: [language] = '2' and [hhcount_s] > 1 and [hhcount_s] < 13 and ([hh2_work_s] = '1' or [hh2_work_s] = '2' or [hh2_work2_s] = '1')	Persona 2: Actualmente ¿con qué frecuencia se le exige a esta persona que trabaje fuera de su residencia?	radio (Matrix)<br>1 | Todo el tiempo (100%)<br>2 | la mayor parte del tiempo (75%)<br>3 | La mitad del tiempo (50%)<br>4 | Menos de la mitad del tiempo (25%)<br>5 | Nunca (0%)

854	[hh2_work8_s]<br><br>Show the field ONLY if:<br>[language] = '2' and ([hh2_work7_s] = '1' or [hh2_work7_s] = '2' or [hh2_work7_s] = '3' or [hh2_work7_s] = '4')	Persona 2: Actualmente ¿con qué frecuencia se encuentra esta persona físicamente cerca de sus compañeros de trabajo mientras trabaja fuera de su residencia?	radio (Matrix)<br>1 Todo el tiempo (100%)<br>2 la mayor parte del tiempo (75%)<br>3 La mitad del tiempo (50%)<br>4 Menos de la mitad del tiempo (25%)<br>5 Nunca (0%)
855	[hh2_work9_s]<br><br>Show the field ONLY if:<br>[language] = '2' and ([hh2_work7_s] = '1' or [hh2_work7_s] = '2' or [hh2_work7_s] = '3' or [hh2_work7_s] = '4')	Persona 2: Actualmente ¿con qué frecuencia se encuentra esta persona físicamente cerca de sus clientes mientras trabaja fuera de su residencia?	radio (Matrix)<br>1 Todo el tiempo (100%)<br>2 la mayor parte del tiempo (75%)<br>3 La mitad del tiempo (50%)<br>4 Menos de la mitad del tiempo (25%)<br>5 Nunca (0%)
856	[hh2_covidvaccine_s]<br><br>Show the field ONLY if:<br>[language] = '2' and [hhcount_s] > 1 and [hhcount_s] < 13	Persona 2: ¿Piensa esta persona vacunarse contra la COVID-19 cuando se ofrezca una vacuna?	radio<br>1 Sí<br>0 No<br>2 No sabe
857	[hh2_covidsymp_s]<br><br>Show the field ONLY if:<br>[language] = '2' and [hhcount_s] > 1 and [hhcount_s] < 13	Persona 2: En las últimas dos semanas, ¿ha tenido esta persona algún síntoma de COVID-19 (tos, fiebre, dificultad para respirar, fatiga, dolores de cuerpo, diarrea, goteo nasal o pérdida del sentido del olfato o del gusto)?	radio<br>1 Sí<br>0 No<br>2 No sabe
858	[hh2_covidsymp2_s]<br><br>Show the field ONLY if:<br>[language] = '2' and [hh2_covidsymp_s] = '1'	Persona 2: ¿Cuándo le comenzaron los síntomas de COVID-19 a esta persona?	text (date_mdy)
859	[hh2_covidsymp3_s]<br><br>Show the field ONLY if:<br>[language] = '2' and [hh2_covidsymp_s] = '1'	Persona 2: En vista de sus síntomas, ¿le preocupa a esta persona la posibilidad de tener COVID-19?	radio<br>1 Sí<br>0 No<br>2 No sabe
860	[hh2_covidsymp4_s]<br><br>Show the field ONLY if:<br>[language] = '2' and [hh2_covidsymp_s] = '1'	Persona 2: ¿Fue esta persona objeto de prejuicio o discriminación debido a sus síntomas?	radio<br>1 Sí<br>0 No<br>2 No sabe
861	[hh2_covidsymp5_s]<br><br>Show the field ONLY if:<br>[language] = '2' and [hh2_covidsymp_s] = '1'	Persona 2: ¿Qué hizo esta persona en vista de sus síntomas?<br>*(Seleccione todo lo que corresponda.)*	checkbox<br>0 hh2_covidsymp5_s___0 Nada<br>1 hh2_covidsymp5_s___1 Tomó medicamentos sin receta (ibuprofeno, acetaminofén, etc.)<br>2 hh2_covidsymp5_s___2 Consultó por teléfono a un proveedor de asistencia médica

			3 | hh2_covidsymp5_s___3 | Fue al consultorio de un proveedor de asistencia médica<br>4 | hh2_covidsymp5_s___4 | Fue a una clínica o una farmacia minorista<br>5 | hh2_covidsymp5_s___5 | Fue a un centro de urgencias (FASTMed, etc.)<br>6 | hh2_covidsymp5_s___6 | Fue a la sala de emergencias<br>7 | hh2_covidsymp5_s___7 | Fue ingresado al hospital<br>8 | hh2_covidsymp5_s___8 | Otra cosa<br>9 | hh2_covidsymp5_s___9 | No sabe
862	[hh2_covidsymp6_s]<br><br>Show the field ONLY if: [language] = '2' and [hh2_covidsymp5_s(8)] = '1'	Persona 2: ¿Qué otra cosa hizo esta persona en vista de sus síntomas?	text
863	[hh2_covidsymp7_s]<br><br>Show the field ONLY if: [language] = '2' and ([hh2_covidsymp5_s(2)] = '1' or [hh2_covidsymp5_s(3)] = '1' or [hh2_covidsymp5_s(4)] = '1' or [hh2_covidsymp5_s(5)] = '1' or [hh2_covidsymp5_s(6)] = '1' or [hh2_covidsymp5_s(7)] = '1' or [hh2_covidsymp5_s(8)] = '1')	Persona 2: ¿Le dijo un proveedor de asistencia médica a esta persona que era posible que tuviera COVID-19?	radio<br>1 | Sí<br>0 | No<br>2 | No sabe
864	[hh2_covid_test_s]<br><br>Show the field ONLY if: [language] = '2' and [hh2_covidsymp_s] = '1'	Persona 2: Si a esta persona le hicieron una prueba de COVID-19 en vista de sus síntomas, ¿cuál fue el resultado?	radio<br>1 | Pendiente<br>2 | Positivo<br>3 | Negativo<br>4 | No concluyente<br>5 | No se hizo la prueba<br>6 | No sabe
865	[hh2_covid_admit_s]<br><br>Show the field ONLY if: [language] = '2' and [hh2_covidsymp5_s(7)] = '1'	Persona 2: ¿Cuántos días estuvo hospitalizada esta persona?	text (number, Min: 0)
866	[hh2_covid_admit2_s]<br><br>Show the field ONLY if: [language] = '2' and [hh2_covidsymp5_s(7)] = '1'	Persona 2: ¿Se le hicieron a esta persona las siguientes intervenciones durante su hospitalización?	checkbox<br>1 | hh2_covid_admit2_s___1 | Oxígeno adicional por la nariz<br>2 | hh2_covid_admit2_s___2 | Tratamiento en la Unidad de Cuidados

			Intensivos (Intensive Care Unit, ICU)<br>3 | hh2_covid_admit2_s___3 | Ventilación mecánica (intubación o tubo de respiración)<br>4 | hh2_covid_admit2_s___4 | No sabe
867	[hh2_covidsymp8_s]<br>Show the field ONLY if: [language] = '2' and [hh2_covidsymp_s] = '1'	Persona 2: ¿Ha vuelto esta persona a su salud normal?	radio<br>1 | Sí<br>0 | No<br>2 | No sabe
868	[hh2_prevent_s]<br>Show the field ONLY if: [language] = '2' and [hh2_covidsymp_s] = '1'	Persona 2: ¿Cuáles de las siguientes medidas tomó esta persona para proteger a sus amigos y familiares después de que comenzaron sus síntomas?	checkbox<br>1 | hh2_prevent_s___1 | Ponerse mascarilla con mayor frecuencia<br>2 | hh2_prevent_s___2 | Lavarse las manos con agua y jabón con mayor frecuencia<br>3 | hh2_prevent_s___3 | Limpiarse las manos con desinfectante con mayor frecuencia<br>4 | hh2_prevent_s___4 | Aislarse en su casa con mayor frecuencia<br>5 | hh2_prevent_s___5 | Quedarse en casa con mayor frecuencia<br>6 | hh2_prevent_s___6 | Ponerse guantes desechables con mayor frecuencia<br>7 | hh2_prevent_s___7 | No sabe
869	[hh3_relationship_s]<br>Show the field ONLY if: [language] = '2' and [hhcount_s] > 2 and [hhcount_s] < 13	Section Header: *Por cada persona adicional en su hogar, proporcione la siguiente informacion.*<br>Persona 3: ¿Cuál es su relación con esta persona?	radio<br>1 | Pareja o cónyuge<br>2 | Hijo o hija<br>3 | Padre o madre<br>4 | Hermano o hermana<br>5 | Otra familiar<br>6 | Proveedor de cuidado infantil u otros cuidados en casa<br>7 | Otra
870	[hh3_relationship2_s]<br>Show the field ONLY if: [language] = '2' and [hh3_relationship_s] = '7'	Persona 3: Especifique su relación con esta persona.	text
871	[hh3_age_s]	Persona 3: ¿Qué edad tiene esta persona? *(Especifique la edad en años)*	text (number, Min: 0, Max: 110)

	Show the field ONLY if: [language] = '2' and [hhcount_s] > 2 and [hhcount_s] < 13		
872	[hh3_sex_s]<br><br>Show the field ONLY if: [language] = '2' and [hhcount_s] > 2 and [hhcount_s] < 13	Persona 3: ¿Cuál es el sexo de esta persona?	radio<br>1 Femenino<br>2 Masculino
873	[hh3_race_s]<br><br>Show the field ONLY if: [language] = '2' and [hhcount_s] > 2 and [hhcount_s] < 13	Persona 3: ¿Cuál es la raza de esta persona?<br>*(Seleccione todo lo que corresponda.)*	checkbox<br>1 hh3_race_s___1 Indio americano o nativo de Alaska<br>2 hh3_race_s___2 Asiático<br>3 hh3_race_s___3 Negro o afroestadounidense<br>4 hh3_race_s___4 Nativo de Hawái o de las islas del Pacífico<br>5 hh3_race_s___5 Blanco<br>6 hh3_race_s___6 Otro<br>7 hh3_race_s___7 No sabe
874	[hh3_ethn_s]<br><br>Show the field ONLY if: [language] = '2' and [hhcount_s] > 2 and [hhcount_s] < 13	Persona 3: ¿Cuál es la identidad étnica de esta persona?	radio<br>1 Hispano o latino<br>2 Ni hispano ni latino<br>3 Otra<br>4 No sabe
875	[hh3_edu_s]<br><br>Show the field ONLY if: [language] = '2' and [hhcount_s] > 2 and [hhcount_s] < 13	Persona 3: ¿Cuál es el nivel de educación de esta persona?	radio<br>1 No tiene educación formal<br>2 Kínder a 8° grado<br>3 Estudios de educación secundaria<br>4 Equivalencia de educación secundaria (GED)<br>5 Diploma de educación secundaria<br>6 Estudios de educación superior<br>7 Título universitario<br>8 Estudios de posgrado o más<br>9 No sabe
876	[hh3_work_s]<br><br>Show the field ONLY if: [language] = '2' and [hhcount_s] > 2 and [hhcount_s] < 13	Persona 3: ¿Cuál de las siguientes es la mejor descripción de la situación laboral actual de esta persona?	radio<br>1 Empleo a tiempo completo<br>2 Empleo a tiempo parcial<br>3 En busca de empleo<br>4 Jubilado<br>5 Ama de casa<br>6 Estudiante<br>7 Permiso de maternidad o paternidad<br>8 Permiso por enfermedad

			9 Desempleado por discapacidad<br>10 Otra<br>11 No sabe
877	[hh3_work2_s]<br><br>Show the field ONLY if:<br>[language] = '2' and [hhcount_s] > 2 and [hhcount_s] < 13	Persona 3: ¿Se considera esta persona actualmente empleado por cuenta propia (contratista independiente, trabajador esporádico (gig), etc.)?	radio<br>1 Sí<br>0 No<br>2 No sabe
878	[hh3_work3_s]<br><br>Show the field ONLY if:<br>[language] = '2' and [hhcount_s] > 2 and [hhcount_s] < 13 and ([hh3_work_s] = '1' or [hh3_work_s] = '2' or [hh3_work2_s] = '1')	Persona 3: ¿Trabaja esta persona actualmente en alguno de los siguientes ambientes de alto riesgo de contagio de COVID-19?	checkbox<br>1 hh3_work3_s___1 Ambiente de asistencia médica (hospital, clínica, centro de urgencias, etc.)<br>2 hh3_work3_s___2 Ambiente residencial denso (hogar de ancianos, otro centro de asistencia de larga duración)<br>3 hh3_work3_s___3 Prisión o cárcel<br>4 hh3_work3_s___4 Establecimiento de envasado de carne<br>5 hh3_work3_s___5 Establecimiento de envío o distribución<br>6 hh3_work3_s___6 Establecimiento minorista de alto volumen (tienda de provisiones, etc.)<br>7 hh3_work3_s___7 No sabe
879	[hh3_work4_s]<br><br>Show the field ONLY if:<br>[language] = '2' and [hhcount_s] > 2 and [hhcount_s] < 13 and ([hh3_work_s] = '1' or [hh3_work_s] = '2' or [hh3_work2_s] = '1')	Persona 3: ¿Le ofrece el empleador a esta persona alguno de los siguientes beneficios en su empleo principal actual?<br>*(Seleccione todo lo que corresponda.)*	checkbox<br>1 hh3_work4_s___1 Permiso por enfermedad con goce de sueldo<br>2 hh3_work4_s___2 Vacaciones o permiso personal con goce de sueldo<br>3 hh3_work4_s___3 Seguro de salud<br>4 hh3_work4_s___4 Seguro de discapacidad<br>5 hh3_work4_s___5 Plan de jubilación<br>6 hh3_work4_s___6 Otro<br>7 hh3_work4_s___7 No sabe
880	[hh3_work5_s]<br><br>Show the field ONLY if:<br>[language] = '2' and [hhcount_s] > 2 and [hhcount_s] < 13 and ([hh3_work_s] = '1' or [hh3_work_s] = '2' or [hh3_work2_s] = '1')	Persona 3: En una escala de 0 (definitivamente no va a suceder) a 10 (definitivamente va a suceder), ¿qué tan probable es que esta persona pierda su empleo debido a la pandemia de COVID-19?	text (number, Min: 0, Max: 10)
881	[hh3_work6_s]	Persona 3: En una escala de 0 (definitivamente no va a suceder) a 10 (definitivamente va a suceder), ¿qué tan	text (number, Min: 0, Max: 10)

	Show the field ONLY if: [language] = '2' and [hhcount_s] > 2 and [hhcount_s] < 13 and ([hh3_work_s] = '1' or [hh3_work_s] = '2' or [hh3_work2_s] = '1')	probable es que a esta persona se le asignen menos horas de trabajo debido a la pandemia de COVID-19?	
882	[hh3_work7_s]<br><br>Show the field ONLY if: [language] = '2' and [hhcount_s] > 2 and [hhcount_s] < 13 and ([hh3_work_s] = '1' or [hh3_work_s] = '2' or [hh3_work2_s] = '1')	Persona 3: Actualmente ¿con qué frecuencia se le exige a esta persona que trabaje fuera de su residencia?	radio (Matrix)<br>1 Todo el tiempo (100%)<br>2 la mayor parte del tiempo (75%)<br>3 La mitad del tiempo (50%)<br>4 Menos de la mitad del tiempo (25%)<br>5 Nunca (0%)
883	[hh3_work8_s]<br><br>Show the field ONLY if: [language] = '2' and ([hh3_work7_s] = '1' or [hh3_work7_s] = '2' or [hh3_work7_s] = '3' or [hh3_work7_s] = '4')	Persona 3: Actualmente ¿con qué frecuencia se encuentra esta persona físicamente cerca de sus compañeros de trabajo mientras trabaja fuera de su residencia?	radio (Matrix)<br>1 Todo el tiempo (100%)<br>2 la mayor parte del tiempo (75%)<br>3 La mitad del tiempo (50%)<br>4 Menos de la mitad del tiempo (25%)<br>5 Nunca (0%)
884	[hh3_work9_s]<br><br>Show the field ONLY if: [language] = '2' and ([hh3_work7_s] = '1' or [hh3_work7_s] = '2' or [hh3_work7_s] = '3' or [hh3_work7_s] = '4')	Persona 3: Actualmente ¿con qué frecuencia se encuentra esta persona físicamente cerca de sus clientes mientras trabaja fuera de su residencia?	radio (Matrix)<br>1 Todo el tiempo (100%)<br>2 la mayor parte del tiempo (75%)<br>3 La mitad del tiempo (50%)<br>4 Menos de la mitad del tiempo (25%)<br>5 Nunca (0%)
885	[hh3_covidvaccine_s]<br><br>Show the field ONLY if: [language] = '2' and [hhcount_s] > 2 and [hhcount_s] < 13	Persona 3: ¿Piensa esta persona vacunarse contra la COVID-19 cuando se ofrezca una vacuna?	radio<br>1 Sí<br>0 No<br>2 No sabe
886	[hh3_covidsymp_s]<br><br>Show the field ONLY if: [language] = '2' and [hhcount_s] > 2 and [hhcount_s] < 13	Persona 3: En las últimas dos semanas, ¿ha tenido esta persona algún síntoma de COVID-19 (tos, fiebre, dificultad para respirar, fatiga, dolores de cuerpo, diarrea, goteo nasal o pérdida del sentido del olfato o del gusto)?	radio<br>1 Sí<br>0 No<br>2 No sabe
887	[hh3_covidsymp2_s]<br><br>Show the field ONLY if: [language] = '2' and [hh3_covidsymp_s] = '1'	Persona 3: ¿Cuándo le comenzaron los síntomas de COVID-19 a esta persona?	text (date_mdy)
888	[hh3_covidsymp3_s]<br><br>Show the field ONLY if: [language] = '2' and [hh3_covidsymp_s] = '1'	Persona 3: En vista de sus síntomas, ¿le preocupa a esta persona la posibilidad de tener COVID-19?	radio<br>1 Sí<br>0 No<br>2 No sabe
889	[hh3_covidsymp4_s]<br><br>Show the field ONLY if: [language] = '2' and [hh3_covidsymp_s] = '1'	Persona 3: ¿Fue esta persona objeto de prejuicio o discriminación debido a sus síntomas?	radio<br>1 Sí<br>0 No<br>2 No sabe

890	[hh3_covidsymp5_s]<br><br>Show the field ONLY if:<br>[language] = '2' and [hh3_covidsymp_s] = '1'	Persona 3: ¿Qué hizo esta persona en vista de sus síntomas?<br>*(Seleccione todo lo que corresponda.)*	checkbox<br>0 hh3_covidsymp5_s___0 Nada<br>1 hh3_covidsymp5_s___1 Tomó medicamentos sin receta (ibuprofeno, acetaminofén, etc.)<br>2 hh3_covidsymp5_s___2 Consultó por teléfono a un proveedor de asistencia médica<br>3 hh3_covidsymp5_s___3 Fue al consultorio de un proveedor de asistencia médica<br>4 hh3_covidsymp5_s___4 Fue a una clínica o una farmacia minorista<br>5 hh3_covidsymp5_s___5 Fue a un centro de urgencias (FASTMed, etc.)<br>6 hh3_covidsymp5_s___6 Fue a la sala de emergencias<br>7 hh3_covidsymp5_s___7 Fue ingresado al hospital<br>8 hh3_covidsymp5_s___8 Otra cosa<br>9 hh3_covidsymp5_s___9 No sabe
891	[hh3_covidsymp6_s]<br><br>Show the field ONLY if:<br>[language] = '2' and [hh3_covidsymp5_s(8)] = '1'	Persona 3: ¿Qué otra cosa hizo esta persona en vista de sus síntomas?	text
892	[hh3_covidsymp7_s]<br><br>Show the field ONLY if:<br>[language] = '2' and ([hh3_covidsymp5_s(2)] = '1' or [hh3_covidsymp5_s(3)] = '1' or [hh3_covidsymp5_s(4)] = '1' or [hh3_covidsymp5_s(5)] = '1' or [hh3_covidsymp5_s(6)] = '1' or [hh3_covidsymp5_s(7)] = '1' or [hh3_covidsymp5_s(8)] = '1')	Persona 3: ¿Le dijo un proveedor de asistencia médica a esta persona que era posible que tuviera COVID-19?	radio<br>1 Sí<br>0 No<br>2 No sabe
893	[hh3_covid_test_s]<br><br>Show the field ONLY if:<br>[language] = '2' and [hh3_covidsymp_s] = '1'	Persona 3: Si a esta persona le hicieron una prueba de COVID-19 en vista de sus síntomas, ¿cuál fue el resultado?	radio<br>1 Pendiente<br>2 Positivo<br>3 Negativo<br>4 No concluyente<br>5 No se hizo la prueba

			6 No sabe
894	[hh3_covid_admit_s]<br>Show the field ONLY if:<br>[language] = '2' and [hh3_covidsymp5_s(7)] = '1'	Persona 3: ¿Cuántos días estuvo hospitalizada esta persona?	text (number, Min: 0)
895	[hh3_covid_admit2_s]<br>Show the field ONLY if:<br>[language] = '2' and [hh3_covidsymp5_s(7)] = '1'	Persona 3: ¿Se le hicieron a esta persona las siguientes intervenciones durante su hospitalización?	checkbox<br>1 hh3_covid_admit2_s___1 Oxígeno adicional por la nariz<br>2 hh3_covid_admit2_s___2 Tratamiento en la Unidad de Cuidados Intensivos (Intensive Care Unit, ICU)<br>3 hh3_covid_admit2_s___3 Ventilación mecánica (intubación o tubo de respiración)<br>4 hh3_covid_admit2_s___4 No sabe
896	[hh3_covidsymp8_s]<br>Show the field ONLY if:<br>[language] = '2' and [hh3_covidsymp_s] = '1'	Persona 3: ¿Ha vuelto esta persona a su salud normal?	radio<br>1 Sí<br>0 No<br>2 No sabe
897	[hh3_prevent_s]<br>Show the field ONLY if:<br>[language] = '2' and [hh3_covidsymp_s] = '1'	Persona 3: ¿Cuáles de las siguientes medidas tomó esta persona para proteger a sus amigos y familiares después de que comenzaron sus síntomas?	checkbox<br>1 hh3_prevent_s___1 Ponerse mascarilla con mayor frecuencia<br>2 hh3_prevent_s___2 Lavarse las manos con agua y jabón con mayor frecuencia<br>3 hh3_prevent_s___3 Limpiarse las manos con desinfectante con mayor frecuencia<br>4 hh3_prevent_s___4 Aislarse en su casa con mayor frecuencia<br>5 hh3_prevent_s___5 Quedarse en casa con mayor frecuencia<br>6 hh3_prevent_s___6 Ponerse guantes desechables con mayor frecuencia<br>7 hh3_prevent_s___7 No sabe

<table>
<tr><td>898</td><td>[hh4_relationship_s]

Show the field ONLY if:
[language] = '2' and [hhcount_s] > 3 and [hhcount_s] < 13</td><td>Section Header: Por cada persona adicional en su hogar, proporcione la siguiente informacion.

Persona 4: ¿Cuál es su relación con esta persona?</td><td>radio
1 Pareja o cónyuge
2 Hijo o hija
3 Padre o madre
4 Hermano o hermana
5 Otra familiar
6 Proveedor de cuidado infantil u otros cuidados en casa
7 Otra</td></tr>
<tr><td>899</td><td>[hh4_relationship2_s]

Show the field ONLY if:
[language] = '2' and [hh4_relationship_s] = '7'</td><td>Persona 4: Especifique su relación con esta persona.</td><td>text</td></tr>
<tr><td>900</td><td>[hh4_age_s]

Show the field ONLY if:
[language] = '2' and [hhcount_s] > 3 and [hhcount_s] < 13</td><td>Persona 4: ¿Qué edad tiene esta persona?
(Especifique la edad en años)</td><td>text (number, Min: 0, Max: 110)</td></tr>
<tr><td>901</td><td>[hh4_sex_s]

Show the field ONLY if:
[language] = '2' and [hhcount_s] > 3 and [hhcount_s] < 13</td><td>Persona 4: ¿Cuál es el sexo de esta persona?</td><td>radio
1 Femenino
2 Masculino</td></tr>
<tr><td>902</td><td>[hh4_race_s]

Show the field ONLY if:
[language] = '2' and [hhcount_s] > 3 and [hhcount_s] < 13</td><td>Persona 4: ¿Cuál es la raza de esta persona?
(Seleccione todo lo que corresponda.)</td><td>checkbox
1 hh4_race_s___1 Indio americano o nativo de Alaska
2 hh4_race_s___2 Asiático
3 hh4_race_s___3 Negro o afroestadounidense
4 hh4_race_s___4 Nativo de Hawái o de las islas del Pacífico
5 hh4_race_s___5 Blanco
6 hh4_race_s___6 Otro
7 hh4_race_s___7 No sabe</td></tr>
<tr><td>903</td><td>[hh4_ethn_s]

Show the field ONLY if:
[language] = '2' and [hhcount_s] > 3 and [hhcount_s] < 13</td><td>Persona 4: ¿Cuál es la identidad étnica de esta persona?</td><td>radio
1 Hispano o latino
2 Ni hispano ni latino
3 Otra
4 No sabe</td></tr>
<tr><td>904</td><td>[hh4_edu_s]

Show the field ONLY if:
[language] = '2' and [hhcount_s] > 3 and [hhcount_s] < 13</td><td>Persona 4: ¿Cuál es el nivel de educación de esta persona?</td><td>radio
1 No tiene educación formal
2 Kínder a 8° grado
3 Estudios de educación secundaria
4 Equivalencia de educación secundaria (GED)
5 Diploma de educación secundaria</td></tr>
</table>

			6 Estudios de educación superior<br>7 Título universitario<br>8 Estudios de posgrado o más<br>9 No sabe
905	[hh4_work_s]<br><br>Show the field ONLY if:<br>[language] = '2' and [hhcount_s] > 3 and [hhcount_s] < 13	Persona 4: ¿Cuál de las siguientes es la mejor descripción de la situación laboral actual de esta persona?	radio<br>1 Empleo a tiempo completo<br>2 Empleo a tiempo parcial<br>3 En busca de empleo<br>4 Jubilado<br>5 Ama de casa<br>6 Estudiante<br>7 Permiso de maternidad o paternidad<br>8 Permiso por enfermedad<br>9 Desempleado por discapacidad<br>10 Otra<br>11 No sabe
906	[hh4_work2_s]<br><br>Show the field ONLY if:<br>[language] = '2' and [hhcount_s] > 3 and [hhcount_s] < 13	Persona 4: ¿Se considera esta persona actualmente empleado por cuenta propia (contratista independiente, trabajador esporádico (gig), etc.)?	radio<br>1 Sí<br>0 No<br>2 No sabe
907	[hh4_work3_s]<br><br>Show the field ONLY if:<br>[language] = '2' and [hhcount_s] > 3 and [hhcount_s] < 13 and ([hh4_work_s] = '1' or [hh4_work_s] = '2' or [hh4_work2_s] = '1')	Persona 4: ¿Trabaja esta persona actualmente en alguno de los siguientes ambientes de alto riesgo de contagio de COVID-19?	checkbox<br>1 hh4_work3_s___1 Ambiente de asistencia médica (hospital, clínica, centro de urgencias, etc.)<br>2 hh4_work3_s___2 Ambiente residencial denso (hogar de ancianos, otro centro de asistencia de larga duración)<br>3 hh4_work3_s___3 Prisión o cárcel<br>4 hh4_work3_s___4 Establecimiento de envasado de carne<br>5 hh4_work3_s___5 Establecimiento de envío o distribución<br>6 hh4_work3_s___6 Establecimiento minorista de alto volumen (tienda de provisiones, etc.)<br>7 hh4_work3_s___7 No sabe

908	[hh4_work4_s]<br><br>Show the field ONLY if: [language] = '2' and [hhcount_s] > 3 and [hhcount_s] < 13 and ([hh4_work_s] = '1' or [hh4_work_s] = '2' or [hh4_work2_s] = '1')	Persona 4: ¿Le ofrece el empleador a esta persona alguno de los siguientes beneficios en su empleo principal actual?<br>*(Seleccione todo lo que corresponda.)*	checkbox<br>1 hh4_work4_s___1 Permiso por enfermedad con goce de sueldo<br>2 hh4_work4_s___2 Vacaciones o permiso personal con goce de sueldo<br>3 hh4_work4_s___3 Seguro de salud<br>4 hh4_work4_s___4 Seguro de discapacidad<br>5 hh4_work4_s___5 Plan de jubilación<br>6 hh4_work4_s___6 Otro<br>7 hh4_work4_s___7 No sabe
909	[hh4_work5_s]<br><br>Show the field ONLY if: [language] = '2' and [hhcount_s] > 3 and [hhcount_s] < 13 and ([hh4_work_s] = '1' or [hh4_work_s] = '2' or [hh4_work2_s] = '1')	Persona 4: En una escala de 0 (definitivamente no va a suceder) a 10 (definitivamente va a suceder), ¿qué tan probable es que esta persona pierda su empleo debido a la pandemia de COVID-19?	text (number, Min: 0, Max: 10)
910	[hh4_work6_s]<br><br>Show the field ONLY if: [language] = '2' and [hhcount_s] > 3 and [hhcount_s] < 13 and ([hh4_work_s] = '1' or [hh4_work_s] = '2' or [hh4_work2_s] = '1')	Persona 4: En una escala de 0 (definitivamente no va a suceder) a 10 (definitivamente va a suceder), ¿qué tan probable es que a esta persona se le asignen menos horas de trabajo debido a la pandemia de COVID-19?	text (number, Min: 0, Max: 10)
911	[hh4_work7_s]<br><br>Show the field ONLY if: [language] = '2' and [hhcount_s] > 3 and [hhcount_s] < 13 and ([hh4_work_s] = '1' or [hh4_work_s] = '2' or [hh4_work2_s] = '1')	Persona 4: Actualmente ¿con qué frecuencia se le exige a esta persona que trabaje fuera de su residencia?	radio (Matrix)<br>1 Todo el tiempo (100%)<br>2 la mayor parte del tiempo (75%)<br>3 La mitad del tiempo (50%)<br>4 Menos de la mitad del tiempo (25%)<br>5 Nunca (0%)
912	[hh4_work8_s]<br><br>Show the field ONLY if: [language] = '2' and ([hh4_work7_s] = '1' or [hh4_work7_s] = '2' or [hh4_work7_s] = '3' or [hh4_work7_s] = '4')	Persona 4: Actualmente ¿con qué frecuencia se encuentra esta persona físicamente cerca de sus compañeros de trabajo mientras trabaja fuera de su residencia?	radio (Matrix)<br>1 Todo el tiempo (100%)<br>2 la mayor parte del tiempo (75%)<br>3 La mitad del tiempo (50%)<br>4 Menos de la mitad del tiempo (25%)<br>5 Nunca (0%)
913	[hh4_work9_s]<br><br>Show the field ONLY if: [language] = '2' and ([hh4_work7_s] = '1' or [hh4_work7_s] = '2' or [hh4_work7_s] = '3' or [hh4_work7_s] = '4')	Persona 4: Actualmente ¿con qué frecuencia se encuentra esta persona físicamente cerca de sus clientes mientras trabaja fuera de su residencia?	radio (Matrix)<br>1 Todo el tiempo (100%)<br>2 la mayor parte del tiempo (75%)<br>3 La mitad del tiempo (50%)<br>4 Menos de la mitad del tiempo (25%)<br>5 Nunca (0%)
914	[hh4_covidvaccine_s]<br><br>Show the field ONLY if:	Persona 4: ¿Piensa esta persona vacunarse contra la COVID-19 cuando se ofrezca una vacuna?	radio<br>1 Sí

	[language] = '2' and [hhcount_s] > 3 and [hhcount_s] < 13		0 No<br>2 No sabe
915	[hh4_covidsymp_s]<br><br>Show the field ONLY if:<br>[language] = '2' and [hhcount_s] > 3 and [hhcount_s] < 13	Persona 4: En las últimas dos semanas, ¿ha tenido esta persona algún síntoma de COVID-19 (tos, fiebre, dificultad para respirar, fatiga, dolores de cuerpo, diarrea, goteo nasal o pérdida del sentido del olfato o del gusto)?	radio<br>1 Sí<br>0 No<br>2 No sabe
916	[hh4_covidsymp2_s]<br><br>Show the field ONLY if:<br>[language] = '2' and [hh4_covidsymp_s] = '1'	Persona 4: ¿Cuándo le comenzaron los síntomas de COVID-19 a esta persona?	text (date_mdy)
917	[hh4_covidsymp3_s]<br><br>Show the field ONLY if:<br>[language] = '2' and [hh4_covidsymp_s] = '1'	Persona 4: En vista de sus síntomas, ¿le preocupa a esta persona la posibilidad de tener COVID-19?	radio<br>1 Sí<br>0 No<br>2 No sabe
918	[hh4_covidsymp4_s]<br><br>Show the field ONLY if:<br>[language] = '2' and [hh4_covidsymp_s] = '1'	Persona 4: ¿Fue esta persona objeto de prejuicio o discriminación debido a sus síntomas?	radio<br>1 Sí<br>0 No<br>2 No sabe
919	[hh4_covidsymp5_s]<br><br>Show the field ONLY if:<br>[language] = '2' and [hh4_covidsymp_s] = '1'	Persona 4: ¿Qué hizo esta persona en vista de sus síntomas?<br>*(Seleccione todo lo que corresponda.)*	checkbox<br>0 hh4_covidsymp5_s___0 Nada<br>1 hh4_covidsymp5_s___1 Tomó medicamentos sin receta (ibuprofeno, acetaminofén, etc.)<br>2 hh4_covidsymp5_s___2 Consultó por teléfono a un proveedor de asistencia médica<br>3 hh4_covidsymp5_s___3 Fue al consultorio de un proveedor de asistencia médica<br>4 hh4_covidsymp5_s___4 Fue a una clínica o una farmacia minorista<br>5 hh4_covidsymp5_s___5 Fue a un centro de urgencias (FASTMed, etc.)<br>6 hh4_covidsymp5_s___6 Fue a la sala de emergencias<br>7 hh4_covidsymp5_s___7 Fue ingresado al hospital<br>8 hh4_covidsymp5_s___8 Otra cosa<br>9 hh4_covidsymp5_s___9 No sabe

920	[hh4_covidsymp6_s]<br><br>Show the field ONLY if:<br>[language] = '2' and [hh4_covidsymp5_s(8)] = '1'	Persona 4: ¿Qué otra cosa hizo esta persona en vista de sus síntomas?	text
921	[hh4_covidsymp7_s]<br><br>Show the field ONLY if:<br>[language] = '2' and ([hh4_covidsymp5_s(2)] = '1' or [hh4_covidsymp5_s(3)] = '1' or [hh4_covidsymp5_s(4)] = '1' or [hh4_covidsymp5_s(5)] = '1' or [hh4_covidsymp5_s(6)] = '1' or [hh4_covidsymp5_s(7)] = '1' or [hh4_covidsymp5_s(8)] = '1')	Persona 4: ¿Le dijo un proveedor de asistencia médica a esta persona que era posible que tuviera COVID-19?	radio<br>1 Sí<br>0 No<br>2 No sabe
922	[hh4_covid_test_s]<br><br>Show the field ONLY if:<br>[language] = '2' and [hh4_covidsymp_s] = '1'	Persona 4: Si a esta persona le hicieron una prueba de COVID-19 en vista de sus síntomas, ¿cuál fue el resultado?	radio<br>1 Pendiente<br>2 Positivo<br>3 Negativo<br>4 No concluyente<br>5 No se hizo la prueba<br>6 No sabe
923	[hh4_covid_admit_s]<br><br>Show the field ONLY if:<br>[language] = '2' and [hh4_covidsymp5_s(7)] = '1'	Persona 4: ¿Cuántos días estuvo hospitalizada esta persona?	text (number, Min: 0)
924	[hh4_covid_admit2_s]<br><br>Show the field ONLY if:<br>[language] = '2' and [hh4_covidsymp5_s(7)] = '1'	Persona 4: ¿Se le hicieron a esta persona las siguientes intervenciones durante su hospitalización?	checkbox<br>1 hh4_covid_admit2_s___1 Oxígeno adicional por la nariz<br>2 hh4_covid_admit2_s___2 Tratamiento en la Unidad de Cuidados Intensivos (Intensive Care Unit, ICU)<br>3 hh4_covid_admit2_s___3 Ventilación mecánica (intubación o tubo de respiración)<br>4 hh4_covid_admit2_s___4 No sabe
925	[hh4_covidsymp8_s]<br><br>Show the field ONLY if:<br>[language] = '2' and [hh4_covidsymp_s] = '1'	Persona 4: ¿Ha vuelto esta persona a su salud normal?	radio<br>1 Sí<br>0 No<br>2 No sabe
926	[hh4_prevent_s]<br><br>Show the field ONLY if:<br>[language] = '2' and [hh4_covidsymp_s] = '1'	Persona 4: ¿Cuáles de las siguientes medidas tomó esta persona para proteger a sus amigos y familiares después de que comenzaron sus síntomas?	checkbox<br>1 hh4_prevent_s___1 Ponerse mascarilla con mayor frecuencia

			2 hh4_prevent_s___2 Lavarse las manos con agua y jabón con mayor frecuencia<br>3 hh4_prevent_s___3 Limpiarse las manos con desinfectante con mayor frecuencia<br>4 hh4_prevent_s___4 Aislarse en su casa con mayor frecuencia<br>5 hh4_prevent_s___5 Quedarse en casa con mayor frecuencia<br>6 hh4_prevent_s___6 Ponerse guantes desechables con mayor frecuencia<br>7 hh4_prevent_s___7 No sabe
927	[hh5_relationship_s]<br><br>Show the field ONLY if:<br>[language] = '2' and [hhcount_s] > 4 and [hhcount_s] < 13	Section Header: *Por cada persona adicional en su hogar, proporcione la siguiente informacion.*<br><br>Persona 5: ¿Cuál es su relación con esta persona?	radio<br>1 Pareja o cónyuge<br>2 Hijo o hija<br>3 Padre o madre<br>4 Hermano o hermana<br>5 Otra familiar<br>6 Proveedor de cuidado infantil u otros cuidados en casa<br>7 Otra
928	[hh5_relationship2_s]<br><br>Show the field ONLY if:<br>[language] = '2' and [hh5_relationship_s] = '7'	Persona 5: Especifique su relación con esta persona.	text
929	[hh5_age_s]<br><br>Show the field ONLY if:<br>[language] = '2' and [hhcount_s] > 4 and [hhcount_s] < 13	Persona 5: ¿Qué edad tiene esta persona?<br>*(Especifique la edad en años)*	text (number, Min: 0, Max: 110)
930	[hh5_sex_s]<br><br>Show the field ONLY if:<br>[language] = '2' and [hhcount_s] > 4 and [hhcount_s] < 13	Persona 5: ¿Cuál es el sexo de esta persona?	radio<br>1 Femenino<br>2 Masculino
931	[hh5_race_s]<br><br>Show the field ONLY if:<br>[language] = '2' and [hhcount_s] > 4 and [hhcount_s] < 13	Persona 5: ¿Cuál es la raza de esta persona?<br>*(Seleccione todo lo que corresponda.)*	checkbox<br>1 hh5_race_s___1 Indio americano o nativo de Alaska<br>2 hh5_race_s___2 Asiático<br>3 hh5_race_s___3 Negro o afroestadounidense<br>4 hh5_race_s___4 Nativo de Hawái o de las islas del Pacífico<br>5 hh5_race_s___5 Blanco

			6 hh5_race_s___6 Otro<br>7 hh5_race_s___7 No sabe
932	[hh5_ethn_s]<br>Show the field ONLY if:<br>[language] = '2' and [hhcount_s] > 4 and [hhcount_s] < 13	Persona 5: ¿Cuál es la identidad étnica de esta persona?	radio<br>1 Hispano o latino<br>2 Ni hispano ni latino<br>3 Otra<br>4 No sabe
933	[hh5_edu_s]<br>Show the field ONLY if:<br>[language] = '2' and [hhcount_s] > 4 and [hhcount_s] < 13	Persona 5: ¿Cuál es el nivel de educación de esta persona?	radio<br>1 No tiene educación formal<br>2 Kínder a 8° grado<br>3 Estudios de educación secundaria<br>4 Equivalencia de educación secundaria (GED)<br>5 Diploma de educación secundaria<br>6 Estudios de educación superior<br>7 Título universitario<br>8 Estudios de posgrado o más<br>9 No sabe
934	[hh5_work_s]<br>Show the field ONLY if:<br>[language] = '2' and [hhcount_s] > 4 and [hhcount_s] < 13	Persona 5: ¿Cuál de las siguientes es la mejor descripción de la situación laboral actual de esta persona?	radio<br>1 Empleo a tiempo completo<br>2 Empleo a tiempo parcial<br>3 En busca de empleo<br>4 Jubilado<br>5 Ama de casa<br>6 Estudiante<br>7 Permiso de maternidad o paternidad<br>8 Permiso por enfermedad<br>9 Desempleado por discapacidad<br>10 Otra<br>11 No sabe
935	[hh5_work2_s]<br>Show the field ONLY if:<br>[language] = '2' and [hhcount_s] > 4 and [hhcount_s] < 13	Persona 5: ¿Se considera esta persona actualmente empleado por cuenta propia (contratista independiente, trabajador esporádico (gig), etc.)?	radio<br>1 Sí<br>0 No<br>2 No sabe
936	[hh5_work3_s]<br>Show the field ONLY if:<br>[language] = '2' and [hhcount_s] > 4 and [hhcount_s] < 13 and ([hh5_work_s] = '1' or [hh5_work_s] = '2' or [hh5_work2_s] = '1')	Persona 5: ¿Trabaja esta persona actualmente en alguno de los siguientes ambientes de alto riesgo de contagio de COVID-19?	checkbox<br>1 hh5_work3_s___1 Ambiente de asistencia médica (hospital, clínica, centro de urgencias, etc.)<br>2 hh5_work3_s___2 Ambiente residencial denso (hogar de ancianos, otro centro de asistencia de larga duración)

			3 hh5_work3_s___3 Prisión o cárcel<br>4 hh5_work3_s___4 Establecimiento de envasado de carne<br>5 hh5_work3_s___5 Establecimiento de envío o distribución<br>6 hh5_work3_s___6 Establecimiento minorista de alto volumen (tienda de provisiones, etc.)<br>7 hh5_work3_s___7 No sabe
937	[hh5_work4_s]<br><br>Show the field ONLY if: [language] = '2' and [hhcount_s] > 4 and [hhcount_s] < 13 and ([hh5_work_s] = '1' or [hh5_work_s] = '2' or [hh5_work2_s] = '1')	Persona 5: ¿Le ofrece el empleador a esta persona alguno de los siguientes beneficios en su empleo principal actual?<br>*(Seleccione todo lo que corresponda.)*	checkbox<br>1 hh5_work4_s___1 Permiso por enfermedad con goce de sueldo<br>2 hh5_work4_s___2 Vacaciones o permiso personal con goce de sueldo<br>3 hh5_work4_s___3 Seguro de salud<br>4 hh5_work4_s___4 Seguro de discapacidad<br>5 hh5_work4_s___5 Plan de jubilación<br>6 hh5_work4_s___6 Otro<br>7 hh5_work4_s___7 No sabe
938	[hh5_work5_s]<br><br>Show the field ONLY if: [language] = '2' and [hhcount_s] > 4 and [hhcount_s] < 13 and ([hh5_work_s] = '1' or [hh5_work_s] = '2' or [hh5_work2_s] = '1')	Persona 5: En una escala de 0 (definitivamente no va a suceder) a 10 (definitivamente va a suceder), ¿qué tan probable es que esta persona pierda su empleo debido a la pandemia de COVID-19?	text (number, Min: 0, Max: 10)
939	[hh5_work6_s]<br><br>Show the field ONLY if: [language] = '2' and [hhcount_s] > 4 and [hhcount_s] < 13 and ([hh5_work_s] = '1' or [hh5_work_s] = '2' or [hh5_work2_s] = '1')	Persona 5: En una escala de 0 (definitivamente no va a suceder) a 10 (definitivamente va a suceder), ¿qué tan probable es que a esta persona se le asignen menos horas de trabajo debido a la pandemia de COVID-19?	text (number, Min: 0, Max: 10)
940	[hh5_work7_s]<br><br>Show the field ONLY if: [language] = '2' and [hhcount_s] > 4 and [hhcount_s] < 13 and ([hh5_work_s] = '1' or [hh5_work_s] = '2' or [hh5_work2_s] = '1')	Persona 5: Actualmente ¿con qué frecuencia se le exige a esta persona que trabaje fuera de su residencia?	radio (Matrix)<br>1 Todo el tiempo (100%)<br>2 la mayor parte del tiempo (75%)<br>3 La mitad del tiempo (50%)<br>4 Menos de la mitad del tiempo (25%)<br>5 Nunca (0%)
941	[hh5_work8_s]<br><br>Show the field ONLY if: [language] = '2' and ([hh5_work7_s] = '1' or [hh5_work7_s] = '2' or [hh5_work7_s] = '3' or [hh5_work7_s] = '4')	Persona 5: Actualmente ¿con qué frecuencia se encuentra esta persona físicamente cerca de sus compañeros de trabajo mientras trabaja fuera de su residencia?	radio (Matrix)<br>1 Todo el tiempo (100%)<br>2 la mayor parte del tiempo (75%)<br>3 La mitad del tiempo (50%)<br>4 Menos de la mitad del tiempo (25%)

			5 Nunca (0%)
942	[hh5_work9_s]<br><br>Show the field ONLY if:<br>[language] = '2' and ([hh5_work7_s] = '1' or [hh5_work7_s] = '2' or [hh5_work7_s] = '3' or [hh5_work7_s] = '4')	Persona 5: Actualmente ¿con qué frecuencia se encuentra esta persona físicamente cerca de sus clientes mientras trabaja fuera de su residencia?	radio (Matrix)<br>1 Todo el tiempo (100%)<br>2 la mayor parte del tiempo (75%)<br>3 La mitad del tiempo (50%)<br>4 Menos de la mitad del tiempo (25%)<br>5 Nunca (0%)
943	[hh5_covidvaccine_s]<br><br>Show the field ONLY if:<br>[language] = '2' and [hhcount_s] > 4 and [hhcount_s] < 13	Persona 5: ¿Piensa esta persona vacunarse contra la COVID-19 cuando se ofrezca una vacuna?	radio<br>1 Sí<br>0 No<br>2 No sabe
944	[hh5_covidsymp_s]<br><br>Show the field ONLY if:<br>[language] = '2' and [hhcount_s] > 4 and [hhcount_s] < 13	Persona 5: En las últimas dos semanas, ¿ha tenido esta persona algún síntoma de COVID-19 (tos, fiebre, dificultad para respirar, fatiga, dolores de cuerpo, diarrea, goteo nasal o pérdida del sentido del olfato o del gusto)?	radio<br>1 Sí<br>0 No<br>2 No sabe
945	[hh5_covidsymp2_s]<br><br>Show the field ONLY if:<br>[language] = '2' and [hh5_covidsymp_s] = '1'	Persona 5: ¿Cuándo le comenzaron los síntomas de COVID-19 a esta persona?	text (date_mdy)
946	[hh5_covidsymp3_s]<br><br>Show the field ONLY if:<br>[language] = '2' and [hh5_covidsymp_s] = '1'	Persona 5: En vista de sus síntomas, ¿le preocupa a esta persona la posibilidad de tener COVID-19?	radio<br>1 Sí<br>0 No<br>2 No sabe
947	[hh5_covidsymp4_s]<br><br>Show the field ONLY if:<br>[language] = '2' and [hh5_covidsymp_s] = '1'	Persona 5: ¿Fue esta persona objeto de prejuicio o discriminación debido a sus síntomas?	radio<br>1 Sí<br>0 No<br>2 No sabe
948	[hh5_covidsymp5_s]<br><br>Show the field ONLY if:<br>[language] = '2' and [hh5_covidsymp_s] = '1'	Persona 5: ¿Qué hizo esta persona en vista de sus síntomas?<br>*(Seleccione todo lo que corresponda.)*	checkbox<br>0 hh5_covidsymp5_s___0 Nada<br>1 hh5_covidsymp5_s___1 Tomó medicamentos sin receta (ibuprofeno, acetaminofén, etc.)<br>2 hh5_covidsymp5_s___2 Consultó por teléfono a un proveedor de asistencia médica<br>3 hh5_covidsymp5_s___3 Fue al consultorio de un proveedor de asistencia médica<br>4 hh5_covidsymp5_s___4 Fue a una clínica o una farmacia

			minorista<br>5 | hh5_covidsymp5_s___5 | Fue a un centro de urgencias (FASTMed, etc.)<br>6 | hh5_covidsymp5_s___6 | Fue a la sala de emergencias<br>7 | hh5_covidsymp5_s___7 | Fue ingresado al hospital<br>8 | hh5_covidsymp5_s___8 | Otra cosa<br>9 | hh5_covidsymp5_s___9 | No sabe
949	[hh5_covidsymp6_s]<br><br>Show the field ONLY if:<br>[language] = '2' and [hh5_covidsymp5_s(8)] = '1'	Persona 5: ¿Qué otra cosa hizo esta persona en vista de sus síntomas?	text
950	[hh5_covidsymp7_s]<br><br>Show the field ONLY if:<br>[language] = '2' and ([hh5_covidsymp5_s(2)] = '1' or [hh5_covidsymp5_s(3)] = '1' or [hh5_covidsymp5_s(4)] = '1' or [hh5_covidsymp5_s(5)] = '1' or [hh5_covidsymp5_s(6)] = '1' or [hh5_covidsymp5_s(7)] = '1' or [hh5_covidsymp5_s(8)] = '1')	Persona 5: ¿Le dijo un proveedor de asistencia médica a esta persona que era posible que tuviera COVID-19?	radio<br>1 | Sí<br>0 | No<br>2 | No sabe
951	[hh5_covid_test_s]<br><br>Show the field ONLY if:<br>[language] = '2' and [hh5_covidsymp_s] = '1'	Persona 5: Si a esta persona le hicieron una prueba de COVID-19 en vista de sus síntomas, ¿cuál fue el resultado?	radio<br>1 | Pendiente<br>2 | Positivo<br>3 | Negativo<br>4 | No concluyente<br>5 | No se hizo la prueba<br>6 | No sabe
952	[hh5_covid_admit_s]<br><br>Show the field ONLY if:<br>[language] = '2' and [hh5_covidsymp5_s(7)] = '1'	Persona 5: ¿Cuántos días estuvo hospitalizada esta persona?	text (number, Min: 0)
953	[hh5_covid_admit2_s]<br><br>Show the field ONLY if:<br>[language] = '2' and [hh5_covidsymp5_s(7)] = '1'	Persona 5: ¿Se le hicieron a esta persona las siguientes intervenciones durante su hospitalización?	checkbox<br>1 | hh5_covid_admit2_s___1 | Oxígeno adicional por la nariz<br>2 | hh5_covid_admit2_s___2 | Tratamiento en la Unidad de Cuidados Intensivos (Intensive Care Unit, ICU)

			3 hh5_covid_admit2_s___3 Ventilación mecánica (intubación o tubo de respiración)<br>4 hh5_covid_admit2_s___4 No sabe
954	[hh5_covidsymp8_s]<br>Show the field ONLY if: [language] = '2' and [hh5_covidsymp_s] = '1'	Persona 5: ¿Ha vuelto esta persona a su salud normal?	radio<br>1 Sí<br>0 No<br>2 No sabe
955	[hh5_prevent_s]<br>Show the field ONLY if: [language] = '2' and [hh5_covidsymp_s] = '1'	Persona 5: ¿Cuáles de las siguientes medidas tomó esta persona para proteger a sus amigos y familiares después de que comenzaron sus síntomas?	checkbox<br>1 hh5_prevent_s___1 Ponerse mascarilla con mayor frecuencia<br>2 hh5_prevent_s___2 Lavarse las manos con agua y jabón con mayor frecuencia<br>3 hh5_prevent_s___3 Limpiarse las manos con desinfectante con mayor frecuencia<br>4 hh5_prevent_s___4 Aislarse en su casa con mayor frecuencia<br>5 hh5_prevent_s___5 Quedarse en casa con mayor frecuencia<br>6 hh5_prevent_s___6 Ponerse guantes desechables con mayor frecuencia<br>7 hh5_prevent_s___7 No sabe
956	[hh6_relationship_s]<br>Show the field ONLY if: [language] = '2' and [hhcount_s] > 5 and [hhcount_s] < 13	Section Header: *Por cada persona adicional en su hogar, proporcione la siguiente informacion.*<br>Persona 6: ¿Cuál es su relación con esta persona?	radio<br>1 Pareja o cónyuge<br>2 Hijo o hija<br>3 Padre o madre<br>4 Hermano o hermana<br>5 Otra familiar<br>6 Proveedor de cuidado infantil u otros cuidados en casa<br>7 Otra
957	[hh6_relationship2_s]<br>Show the field ONLY if: [language] = '2' and [hh6_relationship_s] = '7'	Persona 6: Especifique su relación con esta persona.	text

958	[hh6_age_s]<br><br>Show the field ONLY if:<br>[language] = '2' and [hhcount_s] > 5 and [hhcount_s] < 13	Persona 6: ¿Qué edad tiene esta persona?<br>*(Especifique la edad en años)*	text (number, Min: 0, Max: 110)
959	[hh6_sex_s]<br><br>Show the field ONLY if:<br>[language] = '2' and [hhcount_s] > 5 and [hhcount_s] < 13	Persona 6: ¿Cuál es el sexo de esta persona?	radio<br>1 Femenino<br>2 Masculino
960	[hh6_race_s]<br><br>Show the field ONLY if:<br>[language] = '2' and [hhcount_s] > 5 and [hhcount_s] < 13	Persona 6: ¿Cuál es la raza de esta persona?<br>*(Seleccione todo lo que corresponda.)*	checkbox<br>1 hh6_race_s___1 Indio americano o nativo de Alaska<br>2 hh6_race_s___2 Asiático<br>3 hh6_race_s___3 Negro o afroestadounidense<br>4 hh6_race_s___4 Nativo de Hawái o de las islas del Pacífico<br>5 hh6_race_s___5 Blanco<br>6 hh6_race_s___6 Otro<br>7 hh6_race_s___7 No sabe
961	[hh6_ethn_s]<br><br>Show the field ONLY if:<br>[language] = '2' and [hhcount_s] > 5 and [hhcount_s] < 13	Persona 6: ¿Cuál es la identidad étnica de esta persona?	radio<br>1 Hispano o latino<br>2 Ni hispano ni latino<br>3 Otra<br>4 No sabe
962	[hh6_edu_s]<br><br>Show the field ONLY if:<br>[language] = '2' and [hhcount_s] > 5 and [hhcount_s] < 13	Persona 6: ¿Cuál es el nivel de educación de esta persona?	radio<br>1 No tiene educación formal<br>2 Kínder a 8° grado<br>3 Estudios de educación secundaria<br>4 Equivalencia de educación secundaria (GED)<br>5 Diploma de educación secundaria<br>6 Estudios de educación superior<br>7 Título universitario<br>8 Estudios de posgrado o más<br>9 No sabe
963	[hh6_work_s]<br><br>Show the field ONLY if:<br>[language] = '2' and [hhcount_s] > 5 and [hhcount_s] < 13	Persona 6: ¿Cuál de las siguientes es la mejor descripción de la situación laboral actual de esta persona?	radio<br>1 Empleo a tiempo completo<br>2 Empleo a tiempo parcial<br>3 En busca de empleo<br>4 Jubilado<br>5 Ama de casa<br>6 Estudiante<br>7 Permiso de maternidad o paternidad

			8 Permiso por enfermedad<br>9 Desempleado por discapacidad<br>10 Otra<br>11 No sabe
964	[hh6_work2_s]<br><br>Show the field ONLY if:<br>[language] = '2' and [hhcount_s] > 5 and [hhcount_s] < 13	Persona 6: ¿Se considera esta persona actualmente empleado por cuenta propia (contratista independiente, trabajador esporádico (gig), etc.)?	radio<br>1 Sí<br>0 No<br>2 No sabe
965	[hh6_work3_s]<br><br>Show the field ONLY if:<br>[language] = '2' and [hhcount_s] > 5 and [hhcount_s] < 13 and ([hh6_work_s] = '1' or [hh6_work_s] = '2' or [hh6_work2_s] = '1')	Persona 6: ¿Trabaja esta persona actualmente en alguno de los siguientes ambientes de alto riesgo de contagio de COVID-19?	checkbox<br>1 hh6_work3_s___1 Ambiente de asistencia médica (hospital, clínica, centro de urgencias, etc.)<br>2 hh6_work3_s___2 Ambiente residencial denso (hogar de ancianos, otro centro de asistencia de larga duración)<br>3 hh6_work3_s___3 Prisión o cárcel<br>4 hh6_work3_s___4 Establecimiento de envasado de carne<br>5 hh6_work3_s___5 Establecimiento de envío o distribución<br>6 hh6_work3_s___6 Establecimiento minorista de alto volumen (tienda de provisiones, etc.)<br>7 hh6_work3_s___7 No sabe
966	[hh6_work4_s]<br><br>Show the field ONLY if:<br>[language] = '2' and [hhcount_s] > 5 and [hhcount_s] < 13 and ([hh6_work_s] = '1' or [hh6_work_s] = '2' or [hh6_work2_s] = '1')	Persona 6: ¿Le ofrece el empleador a esta persona alguno de los siguientes beneficios en su empleo principal actual?<br>*(Seleccione todo lo que corresponda.)*	checkbox<br>1 hh6_work4_s___1 Permiso por enfermedad con goce de sueldo<br>2 hh6_work4_s___2 Vacaciones o permiso personal con goce de sueldo<br>3 hh6_work4_s___3 Seguro de salud<br>4 hh6_work4_s___4 Seguro de discapacidad<br>5 hh6_work4_s___5 Plan de jubilación<br>6 hh6_work4_s___6 Otro<br>7 hh6_work4_s___7 No sabe
967	[hh6_work5_s]<br><br>Show the field ONLY if:<br>[language] = '2' and [hhcount_s] > 5 and [hhcount_s] < 13 and ([hh6_work_s] = '1' or [hh6_work_s] = '2' or [hh6_work2_s] = '1')	Persona 6: En una escala de 0 (definitivamente no va a suceder) a 10 (definitivamente va a suceder), ¿qué tan probable es que esta persona pierda su empleo debido a la pandemia de COVID-19?	text (number, Min: 0, Max: 10)

968	[hh6_work6_s]<br><br>Show the field ONLY if:<br>[language] = '2' and [hhcount_s] > 5 and [hhcount_s] < 13 and ([hh6_work_s] = '1' or [hh6_work_s] = '2' or [hh6_work2_s] = '1')	Persona 6: En una escala de 0 (definitivamente no va a suceder) a 10 (definitivamente va a suceder), ¿qué tan probable es que a esta persona se le asignen menos horas de trabajo debido a la pandemia de COVID-19?	text (number, Min: 0, Max: 10)
969	[hh6_work7_s]<br><br>Show the field ONLY if:<br>[language] = '2' and [hhcount_s] > 5 and [hhcount_s] < 13 and ([hh6_work_s] = '1' or [hh6_work_s] = '2' or [hh6_work2_s] = '1')	Persona 6: Actualmente ¿con qué frecuencia se le exige a esta persona que trabaje fuera de su residencia?	radio (Matrix)<br>1 Todo el tiempo (100%)<br>2 la mayor parte del tiempo (75%)<br>3 La mitad del tiempo (50%)<br>4 Menos de la mitad del tiempo (25%)<br>5 Nunca (0%)
970	[hh6_work8_s]<br><br>Show the field ONLY if:<br>[language] = '2' and ([hh6_work7_s] = '1' or [hh6_work7_s] = '2' or [hh6_work7_s] = '3' or [hh6_work7_s] = '4')	Persona 6: Actualmente ¿con qué frecuencia se encuentra esta persona físicamente cerca de sus compañeros de trabajo mientras trabaja fuera de su residencia?	radio (Matrix)<br>1 Todo el tiempo (100%)<br>2 la mayor parte del tiempo (75%)<br>3 La mitad del tiempo (50%)<br>4 Menos de la mitad del tiempo (25%)<br>5 Nunca (0%)
971	[hh6_work9_s]<br><br>Show the field ONLY if:<br>[language] = '2' and ([hh6_work7_s] = '1' or [hh6_work7_s] = '2' or [hh6_work7_s] = '3' or [hh6_work7_s] = '4')	Persona 6: Actualmente ¿con qué frecuencia se encuentra esta persona físicamente cerca de sus clientes mientras trabaja fuera de su residencia?	radio (Matrix)<br>1 Todo el tiempo (100%)<br>2 la mayor parte del tiempo (75%)<br>3 La mitad del tiempo (50%)<br>4 Menos de la mitad del tiempo (25%)<br>5 Nunca (0%)
972	[hh6_covidvaccine_s]<br><br>Show the field ONLY if:<br>[language] = '2' and [hhcount_s] > 5 and [hhcount_s] < 13	Persona 6: ¿Piensa esta persona vacunarse contra la COVID-19 cuando se ofrezca una vacuna?	radio<br>1 Sí<br>0 No<br>2 No sabe
973	[hh6_covidsymp_s]<br><br>Show the field ONLY if:<br>[language] = '2' and [hhcount_s] > 5 and [hhcount_s] < 13	Persona 6: En las últimas dos semanas, ¿ha tenido esta persona algún síntoma de COVID-19 (tos, fiebre, dificultad para respirar, fatiga, dolores de cuerpo, diarrea, goteo nasal o pérdida del sentido del olfato o del gusto)?	radio<br>1 Sí<br>0 No<br>2 No sabe
974	[hh6_covidsymp2_s]<br><br>Show the field ONLY if:<br>[language] = '2' and [hh6_covidsymp_s] = '1'	Persona 6: ¿Cuándo le comenzaron los síntomas de COVID-19 a esta persona?	text (date_mdy)
975	[hh6_covidsymp3_s]<br><br>Show the field ONLY if:<br>[language] = '2' and [hh6_covidsymp_s] = '1'	Persona 6: En vista de sus síntomas, ¿le preocupa a esta persona la posibilidad de tener COVID-19?	radio<br>1 Sí<br>0 No<br>2 No sabe

976	[hh6_covidsymp4_s]<br><br>Show the field ONLY if:<br>[language] = '2' and [hh6_covidsymp_s] = '1'	Persona 6: ¿Fue esta persona objeto de prejuicio o discriminación debido a sus síntomas?	radio<br>1 Sí<br>0 No<br>2 No sabe
977	[hh6_covidsymp5_s]<br><br>Show the field ONLY if:<br>[language] = '2' and [hh6_covidsymp_s] = '1'	Persona 6: ¿Qué hizo esta persona en vista de sus síntomas?<br>*(Seleccione todo lo que corresponda.)*	checkbox<br>0 hh6_covidsymp5_s___0 Nada<br>1 hh6_covidsymp5_s___1 Tomó medicamentos sin receta (ibuprofeno, acetaminofén, etc.)<br>2 hh6_covidsymp5_s___2 Consultó por teléfono a un proveedor de asistencia médica<br>3 hh6_covidsymp5_s___3 Fue al consultorio de un proveedor de asistencia médica<br>4 hh6_covidsymp5_s___4 Fue a una clínica o una farmacia minorista<br>5 hh6_covidsymp5_s___5 Fue a un centro de urgencias (FASTMed, etc.)<br>6 hh6_covidsymp5_s___6 Fue a la sala de emergencias<br>7 hh6_covidsymp5_s___7 Fue ingresado al hospital<br>8 hh6_covidsymp5_s___8 Otra cosa<br>9 hh6_covidsymp5_s___9 No sabe
978	[hh6_covidsymp6_s]<br><br>Show the field ONLY if:<br>[language] = '2' and [hh6_covidsymp5_s(8)] = '1'	Persona 6 ¿Qué otra cosa hizo esta persona en vista de sus síntomas?	text
979	[hh6_covidsymp7_s]<br><br>Show the field ONLY if:<br>[language] = '2' and ([hh6_covidsymp5_s(2)] = '1' or [hh6_covidsymp5_s(3)] = '1' or [hh6_covidsymp5_s(4)] = '1' or [hh6_covidsymp5_s(5)] = '1' or [hh6_covidsymp5_s(6)] = '1' or [hh6_covidsymp5_s(7)] = '1' or [hh6_covidsymp5_s(8)] = '1')	Persona 6: ¿Le dijo un proveedor de asistencia médica a esta persona que era posible que tuviera COVID-19?	radio<br>1 Sí<br>0 No<br>2 No sabe
980	[hh6_covid_test_s]<br><br>Show the field ONLY if:	Persona 6: Si a esta persona le hicieron una prueba de COVID-19 en vista de sus síntomas, ¿cuál fue el resultado?	radio<br>1 Pendiente

	[language] = '2' and [hh6_covidsymp_s] = '1'		2 Positivo<br>3 Negativo<br>4 No concluyente<br>5 No se hizo la prueba<br>6 No sabe
981	[hh6_covid_admit_s]<br><br>Show the field ONLY if: [language] = '2' and [hh6_covidsymp5_s(7)] = '1'	Persona 6: ¿Cuántos días estuvo hospitalizada esta persona?	text (number, Min: 0)
982	[hh6_covid_admit2_s]<br><br>Show the field ONLY if: [language] = '2' and [hh6_covidsymp5_s(7)] = '1'	Persona 6: ¿Se le hicieron a esta persona las siguientes intervenciones durante su hospitalización?	checkbox<br>1 hh6_covid_admit2_s___1 Oxígeno adicional por la nariz<br>2 hh6_covid_admit2_s___2 Tratamiento en la Unidad de Cuidados Intensivos (Intensive Care Unit, ICU)<br>3 hh6_covid_admit2_s___3 Ventilación mecánica (intubación o tubo de respiración)<br>4 hh6_covid_admit2_s___4 No sabe
983	[hh6_covidsymp8_s]<br><br>Show the field ONLY if: [language] = '2' and [hh6_covidsymp_s] = '1'	Persona 6: ¿Ha vuelto esta persona a su salud normal?	radio<br>1 Sí<br>0 No<br>2 No sabe

984	[hh6_prevent_s]<br><br>Show the field ONLY if:<br>[language] = '2' and [hh6_covidsymp_s] = '1'	Persona 6: ¿Cuáles de las siguientes medidas tomó esta persona para proteger a sus amigos y familiares después de que comenzaron sus síntomas?	checkbox<br>1 hh6_prevent_s___1 Ponerse mascarilla con mayor frecuencia<br>2 hh6_prevent_s___2 Lavarse las manos con agua y jabón con mayor frecuencia<br>3 hh6_prevent_s___3 Limpiarse las manos con desinfectante con mayor frecuencia<br>4 hh6_prevent_s___4 Aislarse en su casa con mayor frecuencia<br>5 hh6_prevent_s___5 Quedarse en casa con mayor frecuencia<br>6 hh6_prevent_s___6 Ponerse guantes desechables con mayor frecuencia<br>7 hh6_prevent_s___7 No sabe
985	[hh7_relationship_s]<br><br>Show the field ONLY if:<br>[language] = '2' and [hhcount_s] > 6 and [hhcount_s] < 13	Section Header: *Por cada persona adicional en su hogar, proporcione la siguiente informacion.*<br><br>Persona 7: ¿Cuál es su relación con esta persona?	radio<br>1 Pareja o cónyuge<br>2 Hijo o hija<br>3 Padre o madre<br>4 Hermano o hermana<br>5 Otra familiar<br>6 Proveedor de cuidado infantil u otros cuidados en casa<br>7 Otra
986	[hh7_relationship2_s]<br><br>Show the field ONLY if:<br>[language] = '2' and [hh7_relationship_s] = '7'	Persona 7: Especifique su relación con esta persona.	text
987	[hh7_age_s]<br><br>Show the field ONLY if:<br>[language] = '2' and [hhcount_s] > 6 and [hhcount_s] < 13	Persona 7: ¿Qué edad tiene esta persona?<br>*(Especifique la edad en años)*	text (number, Min: 0, Max: 110)
988	[hh7_sex_s]<br><br>Show the field ONLY if:<br>[language] = '2' and [hhcount_s] > 6 and [hhcount_s] < 13	Persona 7: ¿Cuál es el sexo de esta persona?	radio<br>1 Femenino<br>2 Masculino
989	[hh7_race_s]<br><br>Show the field ONLY if:<br>[language] = '2' and [hhcount_s] > 6 and [hhcount_s] < 13	Persona 7: ¿Cuál es la raza de esta persona?<br>*(Seleccione todo lo que corresponda.)*	checkbox<br>1 hh7_race_s___1 Indio americano o nativo de Alaska<br>2 hh7_race_s___2 Asiático

			3 hh7_race_s___3 Negro o afroestadounidense<br>4 hh7_race_s___4 Nativo de Hawái o de las islas del Pacífico<br>5 hh7_race_s___5 Blanco<br>6 hh7_race_s___6 Otro<br>7 hh7_race_s___7 No sabe
990	[hh7_ethn_s]<br>Show the field ONLY if:<br>[language] = '2' and [hhcount_s] > 6 and [hhcount_s] < 13	Persona 7: ¿Cuál es la identidad étnica de esta persona?	radio<br>1 Hispano o latino<br>2 Ni hispano ni latino<br>3 Otra<br>4 No sabe
991	[hh7_edu_s]<br>Show the field ONLY if:<br>[language] = '2' and [hhcount_s] > 6 and [hhcount_s] < 13	Persona 7: ¿Cuál es el nivel de educación de esta persona?	radio<br>1 No tiene educación formal<br>2 Kínder a 8° grado<br>3 Estudios de educación secundaria<br>4 Equivalencia de educación secundaria (GED)<br>5 Diploma de educación secundaria<br>6 Estudios de educación superior<br>7 Título universitario<br>8 Estudios de posgrado o más<br>9 No sabe
992	[hh7_work_s]<br>Show the field ONLY if:<br>[language] = '2' and [hhcount_s] > 6 and [hhcount_s] < 13	Persona 7: ¿Cuál de las siguientes es la mejor descripción de la situación laboral actual de esta persona?	radio<br>1 Empleo a tiempo completo<br>2 Empleo a tiempo parcial<br>3 En busca de empleo<br>4 Jubilado<br>5 Ama de casa<br>6 Estudiante<br>7 Permiso de maternidad o paternidad<br>8 Permiso por enfermedad<br>9 Desempleado por discapacidad<br>10 Otra<br>11 No sabe
993	[hh7_work2_s]<br>Show the field ONLY if:<br>[language] = '2' and [hhcount_s] > 6 and [hhcount_s] < 13	Persona 7: ¿Se considera esta persona actualmente empleado por cuenta propia (contratista independiente, trabajador esporádico (gig), etc.)?	radio<br>1 Sí<br>0 No<br>2 No sabe
994	[hh7_work3_s]<br>Show the field ONLY if:<br>[language] = '2' and [hhcount_s] > 6 and [hhcount_s] < 13 and ([hh7_work_s]	Persona 7: ¿Trabaja esta persona actualmente en alguno de los siguientes ambientes de alto riesgo de contagio de COVID-19?	checkbox<br>1 hh7_work3_s___1 Ambiente de asistencia médica (hospital, clínica,

	= '1' or [hh7_work_s] = '2' or [hh7_work2_s] = '1')		(continued) centro de urgencias, etc.)<br>2 | hh7_work3_s___2 | Ambiente residencial denso (hogar de ancianos, otro centro de asistencia de larga duración)<br>3 | hh7_work3_s___3 | Prisión o cárcel<br>4 | hh7_work3_s___4 | Establecimiento de envasado de carne<br>5 | hh7_work3_s___5 | Establecimiento de envío o distribución<br>6 | hh7_work3_s___6 | Establecimiento minorista de alto volumen (tienda de provisiones, etc.)<br>7 | hh7_work3_s___7 | No sabe
995	[hh7_work4_s]<br><br>Show the field ONLY if: [language] = '2' and [hhcount_s] > 6 and [hhcount_s] < 13 and ([hh7_work_s] = '1' or [hh7_work_s] = '2' or [hh7_work2_s] = '1')	Persona 7: ¿Le ofrece el empleador a esta persona alguno de los siguientes beneficios en su empleo principal actual?<br>*(Seleccione todo lo que corresponda.)*	checkbox<br>1 | hh7_work4_s___1 | Permiso por enfermedad con goce de sueldo<br>2 | hh7_work4_s___2 | Vacaciones o permiso personal con goce de sueldo<br>3 | hh7_work4_s___3 | Seguro de salud<br>4 | hh7_work4_s___4 | Seguro de discapacidad<br>5 | hh7_work4_s___5 | Plan de jubilación<br>6 | hh7_work4_s___6 | Otro<br>7 | hh7_work4_s___7 | No sabe
996	[hh7_work5_s]<br><br>Show the field ONLY if: [language] = '2' and [hhcount_s] > 6 and [hhcount_s] < 13 and ([hh7_work_s] = '1' or [hh7_work_s] = '2' or [hh7_work2_s] = '1')	Persona 7: En una escala de 0 (definitivamente no va a suceder) a 10 (definitivamente va a suceder), ¿qué tan probable es que esta persona pierda su empleo debido a la pandemia de COVID-19?	text (number, Min: 0, Max: 10)
997	[hh7_work6_s]<br><br>Show the field ONLY if: [language] = '2' and [hhcount_s] > 6 and [hhcount_s] < 13 and ([hh7_work_s] = '1' or [hh7_work_s] = '2' or [hh7_work2_s] = '1')	Persona 7: En una escala de 0 (definitivamente no va a suceder) a 10 (definitivamente va a suceder), ¿qué tan probable es que a esta persona se le asignen menos horas de trabajo debido a la pandemia de COVID-19?	text (number, Min: 0, Max: 10)
998	[hh7_work7_s]<br><br>Show the field ONLY if: [language] = '2' and [hhcount_s] > 6 and [hhcount_s] < 13 and ([hh7_work_s] = '1' or [hh7_work_s] = '2' or [hh7_work2_s] = '1')	Persona 7: Actualmente ¿con qué frecuencia se le exige a esta persona que trabaje fuera de su residencia?	radio (Matrix)<br>1 | Todo el tiempo (100%)<br>2 | la mayor parte del tiempo (75%)<br>3 | La mitad del tiempo (50%)<br>4 | Menos de la mitad del tiempo (25%)<br>5 | Nunca (0%)

999	[hh7_work8_s]<br><br>Show the field ONLY if:<br>[language] = '2' and ([hh7_work7_s] = '1' or [hh7_work7_s] = '2' or [hh7_work7_s] = '3' or [hh7_work7_s] = '4')	Persona 7: Actualmente ¿con qué frecuencia se encuentra esta persona físicamente cerca de sus compañeros de trabajo mientras trabaja fuera de su residencia?	radio (Matrix)<br>1 Todo el tiempo (100%)<br>2 la mayor parte del tiempo (75%)<br>3 La mitad del tiempo (50%)<br>4 Menos de la mitad del tiempo (25%)<br>5 Nunca (0%)
1000	[hh7_work9_s]<br><br>Show the field ONLY if:<br>[language] = '2' and ([hh7_work7_s] = '1' or [hh7_work7_s] = '2' or [hh7_work7_s] = '3' or [hh7_work7_s] = '4')	Persona 7: Actualmente ¿con qué frecuencia se encuentra esta persona físicamente cerca de sus clientes mientras trabaja fuera de su residencia?	radio (Matrix)<br>1 Todo el tiempo (100%)<br>2 la mayor parte del tiempo (75%)<br>3 La mitad del tiempo (50%)<br>4 Menos de la mitad del tiempo (25%)<br>5 Nunca (0%)
1001	[hh7_covidvaccine_s]<br><br>Show the field ONLY if:<br>[language] = '2' and [hhcount_s] > 6 and [hhcount_s] < 13	Persona 7: ¿Piensa esta persona vacunarse contra la COVID-19 cuando se ofrezca una vacuna?	radio<br>1 Sí<br>0 No<br>2 No sabe
1002	[hh7_covidsymp_s]<br><br>Show the field ONLY if:<br>[language] = '2' and [hhcount_s] > 6 and [hhcount_s] < 13	Persona 7: En las últimas dos semanas, ¿ha tenido esta persona algún síntoma de COVID-19 (tos, fiebre, dificultad para respirar, fatiga, dolores de cuerpo, diarrea, goteo nasal o pérdida del sentido del olfato o del gusto)?	radio<br>1 Sí<br>0 No<br>2 No sabe
1003	[hh7_covidsymp2_s]<br><br>Show the field ONLY if:<br>[language] = '2' and [hh7_covidsymp_s] = '1'	Persona 7: ¿Cuándo le comenzaron los síntomas de COVID-19 a esta persona?	text (date_mdy)
1004	[hh7_covidsymp3_s]<br><br>Show the field ONLY if:<br>[language] = '2' and [hh7_covidsymp_s] = '1'	Persona 7: En vista de sus síntomas, ¿le preocupa a esta persona la posibilidad de tener COVID-19?	radio<br>1 Sí<br>0 No<br>2 No sabe
1005	[hh7_covidsymp4_s]<br><br>Show the field ONLY if:<br>[language] = '2' and [hh7_covidsymp_s] = '1'	Persona 7: ¿Fue esta persona objeto de prejuicio o discriminación debido a sus síntomas?	radio<br>1 Sí<br>0 No<br>2 No sabe
1006	[hh7_covidsymp5_s]<br><br>Show the field ONLY if:<br>[language] = '2' and [hh7_covidsymp_s] = '1'	Persona 7: ¿Qué hizo esta persona en vista de sus síntomas?<br>*(Seleccione todo lo que corresponda.)*	checkbox<br>0 hh7_covidsymp5_s___0 Nada<br>1 hh7_covidsymp5_s___1 Tomó medicamentos sin receta (ibuprofeno, acetaminofén, etc.)<br>2 hh7_covidsymp5_s___2 Consultó por teléfono a un proveedor de asistencia médica

			3 | hh7_covidsymp5_s___3 | Fue al consultorio de un proveedor de asistencia médica<br>4 | hh7_covidsymp5_s___4 | Fue a una clínica o una farmacia minorista<br>5 | hh7_covidsymp5_s___5 | Fue a un centro de urgencias (FASTMed, etc.)<br>6 | hh7_covidsymp5_s___6 | Fue a la sala de emergencias<br>7 | hh7_covidsymp5_s___7 | Fue ingresado al hospital<br>8 | hh7_covidsymp5_s___8 | Otra cosa<br>9 | hh7_covidsymp5_s___9 | No sabe
1007	[hh7_covidsymp6_s]<br><br>Show the field ONLY if:<br>[language] = '2' and [hh7_covidsymp5_s(8)] = '1'	Persona 7 ¿Qué otra cosa hizo esta persona en vista de sus síntomas?	text
1008	[hh7_covidsymp7_s]<br><br>Show the field ONLY if:<br>[language] = '2' and ([hh7_covidsymp5_s(2)] = '1' or [hh7_covidsymp5_s(3)] = '1' or [hh7_covidsymp5_s(4)] = '1' or [hh7_covidsymp5_s(5)] = '1' or [hh7_covidsymp5_s(6)] = '1' or [hh7_covidsymp5_s(7)] = '1' or [hh7_covidsymp5_s(8)] = '1')	Persona 7: ¿Le dijo un proveedor de asistencia médica a esta persona que era posible que tuviera COVID-19?	radio<br>1 | Sí<br>0 | No<br>2 | No sabe
1009	[hh7_covid_test_s]<br><br>Show the field ONLY if:<br>[language] = '2' and [hh7_covidsymp_s] = '1'	Persona 7: Si a esta persona le hicieron una prueba de COVID-19 en vista de sus síntomas, ¿cuál fue el resultado?	radio<br>1 | Pendiente<br>2 | Positivo<br>3 | Negativo<br>4 | No concluyente<br>5 | No se hizo la prueba<br>6 | No sabe
1010	[hh7_covid_admit_s]<br><br>Show the field ONLY if:<br>[language] = '2' and [hh7_covidsymp5_s(7)] = '1'	Persona 7: ¿Cuántos días estuvo hospitalizada esta persona?	text (number, Min: 0)
1011	[hh7_covid_admit2_s]<br><br>Show the field ONLY if:<br>[language] = '2' and [hh7_covidsymp5_s(7)] = '1'	Persona 7: ¿Se le hicieron a esta persona las siguientes intervenciones durante su hospitalización?	checkbox<br>1 | hh7_covid_admit2_s___1 | Oxígeno adicional por la nariz<br>2 | hh7_covid_admit2_s___2 | Tratamiento en la Unidad de Cuidados

			Intensivos (Intensive Care Unit, ICU)<br>3 | hh7_covid_admit2_s___3 | Ventilación mecánica (intubación o tubo de respiración)<br>4 | hh7_covid_admit2_s___4 | No sabe
1012	[hh7_covidsymp8_s]<br><br>Show the field ONLY if:<br>[language] = '2' and [hh7_covidsymp_s] = '1'	Persona 7: ¿Ha vuelto esta persona a su salud normal?	radio<br>1 | Sí<br>0 | No<br>2 | No sabe
1013	[hh7_prevent_s]<br><br>Show the field ONLY if:<br>[language] = '2' and [hh7_covidsymp_s] = '1'	Persona 7: ¿Cuáles de las siguientes medidas tomó esta persona para proteger a sus amigos y familiares después de que comenzaron sus síntomas?	checkbox<br>1 | hh7_prevent_s___1 | Ponerse mascarilla con mayor frecuencia<br>2 | hh7_prevent_s___2 | Lavarse las manos con agua y jabón con mayor frecuencia<br>3 | hh7_prevent_s___3 | Limpiarse las manos con desinfectante con mayor frecuencia<br>4 | hh7_prevent_s___4 | Aislarse en su casa con mayor frecuencia<br>5 | hh7_prevent_s___5 | Quedarse en casa con mayor frecuencia<br>6 | hh7_prevent_s___6 | Ponerse guantes desechables con mayor frecuencia<br>7 | hh7_prevent_s___7 | No sabe
1014	[hh8_relationship_s]<br><br>Show the field ONLY if:<br>[language] = '2' and [hhcount_s] > 7 and [hhcount_s] < 13	Section Header: *Por cada persona adicional en su hogar, proporcione la siguiente informacion.*<br><br>Persona 8: ¿Cuál es su relación con esta persona?	radio<br>1 | Pareja o cónyuge<br>2 | Hijo o hija<br>3 | Padre o madre<br>4 | Hermano o hermana<br>5 | Otra familiar<br>6 | Proveedor de cuidado infantil u otros cuidados en casa<br>7 | Otra
1015	[hh8_relationship2_s]<br><br>Show the field ONLY if:<br>[language] = '2' and [hh8_relationship_s] = '7'	Persona 8: Especifique su relación con esta persona.	text
1016	[hh8_age_s]	Persona 8: ¿Qué edad tiene esta persona?<br>*(Especifique la edad en años)*	text (number, Min: 0, Max: 110)

	Show the field ONLY if: [language] = '2' and [hhcount_s] > 7 and [hhcount_s] < 13		
1017	[hh8_sex_s] Show the field ONLY if: [language] = '2' and [hhcount_s] > 7 and [hhcount_s] < 13	Persona 8: ¿Cuál es el sexo de esta persona?	radio<br>1 Femenino<br>2 Masculino
1018	[hh8_race_s] Show the field ONLY if: [language] = '2' and [hhcount_s] > 7 and [hhcount_s] < 13	Persona 8: ¿Cuál es la raza de esta persona? *(Seleccione todo lo que corresponda.)*	checkbox<br>1 hh8_race_s___1 Indio americano o nativo de Alaska<br>2 hh8_race_s___2 Asiático<br>3 hh8_race_s___3 Negro o afroestadounidense<br>4 hh8_race_s___4 Nativo de Hawái o de las islas del Pacífico<br>5 hh8_race_s___5 Blanco<br>6 hh8_race_s___6 Otro<br>7 hh8_race_s___7 No sabe
1019	[hh8_ethn_s] Show the field ONLY if: [language] = '2' and [hhcount_s] > 7 and [hhcount_s] < 13	Persona 8: ¿Cuál es la identidad étnica de esta persona?	radio<br>1 Hispano o latino<br>2 Ni hispano ni latino<br>3 Otra<br>4 No sabe
1020	[hh8_edu_s] Show the field ONLY if: [language] = '2' and [hhcount_s] > 7 and [hhcount_s] < 13	Persona 8: ¿Cuál es el nivel de educación de esta persona?	radio<br>1 No tiene educación formal<br>2 Kínder a 8° grado<br>3 Estudios de educación secundaria<br>4 Equivalencia de educación secundaria (GED)<br>5 Diploma de educación secundaria<br>6 Estudios de educación superior<br>7 Título universitario<br>8 Estudios de posgrado o más<br>9 No sabe
1021	[hh8_work_s] Show the field ONLY if: [language] = '2' and [hhcount_s] > 7 and [hhcount_s] < 13	Persona 8: ¿Cuál de las siguientes es la mejor descripción de la situación laboral actual de esta persona?	radio<br>1 Empleo a tiempo completo<br>2 Empleo a tiempo parcial<br>3 En busca de empleo<br>4 Jubilado<br>5 Ama de casa<br>6 Estudiante<br>7 Permiso de maternidad o paternidad<br>8 Permiso por enfermedad

			9 Desempleado por discapacidad<br>10 Otra<br>11 No sabe
1022	[hh8_work2_s]<br>Show the field ONLY if: [language] = '2' and [hhcount_s] > 7 and [hhcount_s] < 13	Persona 8: ¿Se considera esta persona actualmente empleado por cuenta propia (contratista independiente, trabajador esporádico (gig), etc.)?	radio<br>1 Sí<br>0 No<br>2 No sabe
1023	[hh8_work3_s]<br>Show the field ONLY if: [language] = '2' and [hhcount_s] > 7 and [hhcount_s] < 13 and ([hh8_work_s] = '1' or [hh8_work_s] = '2' or [hh8_work2_s] = '1')	Persona 8: ¿Trabaja esta persona actualmente en alguno de los siguientes ambientes de alto riesgo de contagio de COVID-19?	checkbox<br>1 hh8_work3_s___1 Ambiente de asistencia médica (hospital, clínica, centro de urgencias, etc.)<br>2 hh8_work3_s___2 Ambiente residencial denso (hogar de ancianos, otro centro de asistencia de larga duración)<br>3 hh8_work3_s___3 Prisión o cárcel<br>4 hh8_work3_s___4 Establecimiento de envasado de carne<br>5 hh8_work3_s___5 Establecimiento de envío o distribución<br>6 hh8_work3_s___6 Establecimiento minorista de alto volumen (tienda de provisiones, etc.)<br>7 hh8_work3_s___7 No sabe
1024	[hh8_work4_s]<br>Show the field ONLY if: [language] = '2' and [hhcount_s] > 7 and [hhcount_s] < 13 and ([hh8_work_s] = '1' or [hh8_work_s] = '2' or [hh8_work2_s] = '1')	Persona 8: ¿Le ofrece el empleador a esta persona alguno de los siguientes beneficios en su empleo principal actual?<br>*(Seleccione todo lo que corresponda.)*	checkbox<br>1 hh8_work4_s___1 Permiso por enfermedad con goce de sueldo<br>2 hh8_work4_s___2 Vacaciones o permiso personal con goce de sueldo<br>3 hh8_work4_s___3 Seguro de salud<br>4 hh8_work4_s___4 Seguro de discapacidad<br>5 hh8_work4_s___5 Plan de jubilación<br>6 hh8_work4_s___6 Otro<br>7 hh8_work4_s___7 No sabe
1025	[hh8_work5_s]<br>Show the field ONLY if: [language] = '2' and [hhcount_s] > 7 and [hhcount_s] < 13 and ([hh8_work_s] = '1' or [hh8_work_s] = '2' or [hh8_work2_s] = '1')	Persona 8: En una escala de 0 (definitivamente no va a suceder) a 10 (definitivamente va a suceder), ¿qué tan probable es que esta persona pierda su empleo debido a la pandemia de COVID-19?	text (number, Min: 0, Max: 10)
1026	[hh8_work6_s]	Persona 8: En una escala de 0 (definitivamente no va a suceder) a 10 (definitivamente va a suceder), ¿qué tan	text (number, Min: 0, Max: 10)

	Show the field ONLY if: [language] = '2' and [hhcount_s] > 7 and [hhcount_s] < 13 and ([hh8_work_s] = '1' or [hh8_work_s] = '2' or [hh8_work2_s] = '1')	probable es que a esta persona se le asignen menos horas de trabajo debido a la pandemia de COVID-19?	
1027	[hh8_work7_s]<br><br>Show the field ONLY if: [language] = '2' and [hhcount_s] > 7 and [hhcount_s] < 13 and ([hh8_work_s] = '1' or [hh8_work_s] = '2' or [hh8_work2_s] = '1')	Persona 8: Actualmente ¿con qué frecuencia se le exige a esta persona que trabaje fuera de su residencia?	radio (Matrix)<br>1 Todo el tiempo (100%)<br>2 la mayor parte del tiempo (75%)<br>3 La mitad del tiempo (50%)<br>4 Menos de la mitad del tiempo (25%)<br>5 Nunca (0%)
1028	[hh8_work8_s]<br><br>Show the field ONLY if: [language] = '2' and ([hh8_work7_s] = '1' or [hh8_work7_s] = '2' or [hh8_work7_s] = '3' or [hh8_work7_s] = '4')	Persona 8: Actualmente ¿con qué frecuencia se encuentra esta persona físicamente cerca de sus compañeros de trabajo mientras trabaja fuera de su residencia?	radio (Matrix)<br>1 Todo el tiempo (100%)<br>2 la mayor parte del tiempo (75%)<br>3 La mitad del tiempo (50%)<br>4 Menos de la mitad del tiempo (25%)<br>5 Nunca (0%)
1029	[hh8_work9_s]<br><br>Show the field ONLY if: [language] = '2' and ([hh8_work7_s] = '1' or [hh8_work7_s] = '2' or [hh8_work7_s] = '3' or [hh8_work7_s] = '4')	Persona 8: Actualmente ¿con qué frecuencia se encuentra esta persona físicamente cerca de sus clientes mientras trabaja fuera de su residencia?	radio (Matrix)<br>1 Todo el tiempo (100%)<br>2 la mayor parte del tiempo (75%)<br>3 La mitad del tiempo (50%)<br>4 Menos de la mitad del tiempo (25%)<br>5 Nunca (0%)
1030	[hh8_covidvaccine_s]<br><br>Show the field ONLY if: [language] = '2' and [hhcount_s] > 7 and [hhcount_s] < 13	Persona 8: ¿Piensa esta persona vacunarse contra la COVID-19 cuando se ofrezca una vacuna?	radio<br>1 Sí<br>0 No<br>2 No sabe
1031	[hh8_covidsymp_s]<br><br>Show the field ONLY if: [language] = '2' and [hhcount_s] > 7 and [hhcount_s] < 13	Persona 8: En las últimas dos semanas, ¿ha tenido esta persona algún síntoma de COVID-19 (tos, fiebre, dificultad para respirar, fatiga, dolores de cuerpo, diarrea, goteo nasal o pérdida del sentido del olfato o del gusto)?	radio<br>1 Sí<br>0 No<br>2 No sabe
1032	[hh8_covidsymp2_s]<br><br>Show the field ONLY if: [language] = '2' and [hh8_covidsymp_s] = '1'	Persona 8: ¿Cuándo le comenzaron los síntomas de COVID-19 a esta persona?	text (date_mdy)
1033	[hh8_covidsymp3_s]<br><br>Show the field ONLY if: [language] = '2' and [hh8_covidsymp_s] = '1'	Persona 8: En vista de sus síntomas, ¿le preocupa a esta persona la posibilidad de tener COVID-19?	radio<br>1 Sí<br>0 No<br>2 No sabe
1034	[hh8_covidsymp4_s]<br><br>Show the field ONLY if: [language] = '2' and [hh8_covidsymp_s] = '1'	Persona 8: ¿Fue esta persona objeto de prejuicio o discriminación debido a sus síntomas?	radio<br>1 Sí<br>0 No<br>2 No sabe

1035	[hh8_covidsymp5_s]<br><br>Show the field ONLY if:<br>[language] = '2' and [hh8_covidsymp_s] = '1'	Persona 8: ¿Qué hizo esta persona en vista de sus síntomas?<br>*(Seleccione todo lo que corresponda.)*	checkbox<br>0 | hh8_covidsymp5_s___0 | Nada<br>1 | hh8_covidsymp5_s___1 | Tomó medicamentos sin receta (ibuprofeno, acetaminofén, etc.)<br>2 | hh8_covidsymp5_s___2 | Consultó por teléfono a un proveedor de asistencia médica<br>3 | hh8_covidsymp5_s___3 | Fue al consultorio de un proveedor de asistencia médica<br>4 | hh8_covidsymp5_s___4 | Fue a una clínica o una farmacia minorista<br>5 | hh8_covidsymp5_s___5 | Fue a un centro de urgencias (FASTMed, etc.)<br>6 | hh8_covidsymp5_s___6 | Fue a la sala de emergencias<br>7 | hh8_covidsymp5_s___7 | Fue ingresado al hospital<br>8 | hh8_covidsymp5_s___8 | Otra cosa<br>9 | hh8_covidsymp5_s___9 | No sabe
1036	[hh8_covidsymp6_s]<br><br>Show the field ONLY if:<br>[language] = '2' and [hh8_covidsymp5_s(8)] = '1'	Persona 8 ¿Qué otra cosa hizo esta persona en vista de sus síntomas?	text
1037	[hh8_covidsymp7_s]<br><br>Show the field ONLY if:<br>[language] = '2' and ([hh8_covidsymp5_s(2)] = '1' or [hh8_covidsymp5_s(3)] = '1' or [hh8_covidsymp5_s(4)] = '1' or [hh8_covidsymp5_s(5)] = '1' or [hh8_covidsymp5_s(6)] = '1' or [hh8_covidsymp5_s(7)] = '1' or [hh8_covidsymp5_s(8)] = '1')	Persona 8: ¿Le dijo un proveedor de asistencia médica a esta persona que era posible que tuviera COVID-19?	radio<br>1 | Sí<br>0 | No<br>2 | No sabe
1038	[hh8_covid_test_s]<br><br>Show the field ONLY if:<br>[language] = '2' and [hh8_covidsymp_s] = '1'	Persona 8: Si a esta persona le hicieron una prueba de COVID-19 en vista de sus síntomas, ¿cuál fue el resultado?	radio<br>1 | Pendiente<br>2 | Positivo<br>3 | Negativo<br>4 | No concluyente<br>5 | No se hizo la prueba

			6 No sabe
1039	[hh8_covid_admit_s]<br><br>Show the field ONLY if:<br>[language] = '2' and [hh8_covidsymp5_s(7)] = '1'	Persona 8: ¿Cuántos días estuvo hospitalizada esta persona?	text (number, Min: 0)
1040	[hh8_covid_admit2_s]<br><br>Show the field ONLY if:<br>[language] = '2' and [hh8_covidsymp5_s(7)] = '1'	Persona 8: ¿Se le hicieron a esta persona las siguientes intervenciones durante su hospitalización?	checkbox<br>1 hh8_covid_admit2_s___1 Oxígeno adicional por la nariz<br>2 hh8_covid_admit2_s___2 Tratamiento en la Unidad de Cuidados Intensivos (Intensive Care Unit, ICU)<br>3 hh8_covid_admit2_s___3 Ventilación mecánica (intubación o tubo de respiración)<br>4 hh8_covid_admit2_s___4 No sabe
1041	[hh8_covidsymp8_s]<br><br>Show the field ONLY if:<br>[language] = '2' and [hh8_covidsymp_s] = '1'	Persona 8: ¿Ha vuelto esta persona a su salud normal?	radio<br>1 Sí<br>0 No<br>2 No sabe
1042	[hh8_prevent_s]<br><br>Show the field ONLY if:<br>[language] = '2' and [hh8_covidsymp_s] = '1'	Persona 8: ¿Cuáles de las siguientes medidas tomó esta persona para proteger a sus amigos y familiares después de que comenzaron sus síntomas?	checkbox<br>1 hh8_prevent_s___1 Ponerse mascarilla con mayor frecuencia<br>2 hh8_prevent_s___2 Lavarse las manos con agua y jabón con mayor frecuencia<br>3 hh8_prevent_s___3 Limpiarse las manos con desinfectante con mayor frecuencia<br>4 hh8_prevent_s___4 Aislarse en su casa con mayor frecuencia<br>5 hh8_prevent_s___5 Quedarse en casa con mayor frecuencia<br>6 hh8_prevent_s___6 Ponerse guantes desechables con mayor frecuencia<br>7 hh8_prevent_s___7 No sabe

1043	[hh9_relationship_s]<br><br>Show the field ONLY if:<br>[language] = '2' and [hhcount_s] > 8 and [hhcount_s] < 13	Section Header: *Por cada persona adicional en su hogar, proporcione la siguiente informacion.*<br><br>Persona 9: ¿Cuál es su relación con esta persona?	radio<br>1 Pareja o cónyuge<br>2 Hijo o hija<br>3 Padre o madre<br>4 Hermano o hermana<br>5 Otra familiar<br>6 Proveedor de cuidado infantil u otros cuidados en casa<br>7 Otra
1044	[hh9_relationship2_s]<br><br>Show the field ONLY if:<br>[language] = '2' and [hh9_relationship_s] = '7'	Persona 9: Especifique su relación con esta persona.	text
1045	[hh9_age_s]<br><br>Show the field ONLY if:<br>[language] = '2' and [hhcount_s] > 8 and [hhcount_s] < 13	Persona 9: ¿Qué edad tiene esta persona?<br>*(Especifique la edad en años)*	text (number, Min: 0, Max: 110)
1046	[hh9_sex_s]<br><br>Show the field ONLY if:<br>[language] = '2' and [hhcount_s] > 8 and [hhcount_s] < 13	Persona 9: ¿Cuál es el sexo de esta persona?	radio<br>1 Femenino<br>2 Masculino
1047	[hh9_race_s]<br><br>Show the field ONLY if:<br>[language] = '2' and [hhcount_s] > 8 and [hhcount_s] < 13	Persona 9: ¿Cuál es la raza de esta persona?<br>*(Seleccione todo lo que corresponda.)*	checkbox<br>1 hh9_race_s___1 Indio americano o nativo de Alaska<br>2 hh9_race_s___2 Asiático<br>3 hh9_race_s___3 Negro o afroestadounidense<br>4 hh9_race_s___4 Nativo de Hawái o de las islas del Pacífico<br>5 hh9_race_s___5 Blanco<br>6 hh9_race_s___6 Otro<br>7 hh9_race_s___7 No sabe
1048	[hh9_ethn_s]<br><br>Show the field ONLY if:<br>[language] = '2' and [hhcount_s] > 8 and [hhcount_s] < 13	Persona 9: ¿Cuál es la identidad étnica de esta persona?	radio<br>1 Hispano o latino<br>2 Ni hispano ni latino<br>3 Otra<br>4 No sabe
1049	[hh9_edu_s]<br><br>Show the field ONLY if:<br>[language] = '2' and [hhcount_s] > 8 and [hhcount_s] < 13	Persona 9: ¿Cuál es el nivel de educación de esta persona?	radio<br>1 No tiene educación formal<br>2 Kínder a 8° grado<br>3 Estudios de educación secundaria<br>4 Equivalencia de educación secundaria (GED)<br>5 Diploma de educación secundaria

			6 Estudios de educación superior<br>7 Título universitario<br>8 Estudios de posgrado o más<br>9 No sabe
1050	[hh9_work_s]<br>Show the field ONLY if:<br>[language] = '2' and [hhcount_s] > 8 and [hhcount_s] < 13	Persona 9: ¿Cuál de las siguientes es la mejor descripción de la situación laboral actual de esta persona?	radio<br>1 Empleo a tiempo completo<br>2 Empleo a tiempo parcial<br>3 En busca de empleo<br>4 Jubilado<br>5 Ama de casa<br>6 Estudiante<br>7 Permiso de maternidad o paternidad<br>8 Permiso por enfermedad<br>9 Desempleado por discapacidad<br>10 Otra<br>11 No sabe
1051	[hh9_work2_s]<br>Show the field ONLY if:<br>[language] = '2' and [hhcount_s] > 8 and [hhcount_s] < 13	Persona 9: ¿Se considera esta persona actualmente empleado por cuenta propia (contratista independiente, trabajador esporádico (gig), etc.)?	radio<br>1 Sí<br>0 No<br>2 No sabe
1052	[hh9_work3_s]<br>Show the field ONLY if:<br>[language] = '2' and [hhcount_s] > 8 and [hhcount_s] < 13 and ([hh9_work_s] = '1' or [hh9_work_s] = '2' or [hh9_work2_s] = '1')	Persona 9: ¿Trabaja esta persona actualmente en alguno de los siguientes ambientes de alto riesgo de contagio de COVID-19?	checkbox<br>1 hh9_work3_s___1 Ambiente de asistencia médica (hospital, clínica, centro de urgencias, etc.)<br>2 hh9_work3_s___2 Ambiente residencial denso (hogar de ancianos, otro centro de asistencia de larga duración)<br>3 hh9_work3_s___3 Prisión o cárcel<br>4 hh9_work3_s___4 Establecimiento de envasado de carne<br>5 hh9_work3_s___5 Establecimiento de envío o distribución<br>6 hh9_work3_s___6 Establecimiento minorista de alto volumen (tienda de provisiones, etc.)<br>7 hh9_work3_s___7 No sabe

1053	[hh9_work4_s]<br><br>Show the field ONLY if:<br>[language] = '2' and [hhcount_s] > 8 and [hhcount_s] < 13 and ([hh9_work_s] = '1' or [hh9_work_s] = '2' or [hh9_work2_s] = '1')	Persona 9: ¿Le ofrece el empleador a esta persona alguno de los siguientes beneficios en su empleo principal actual?<br>*(Seleccione todo lo que corresponda.)*	checkbox<br>1 hh9_work4_s___1 Permiso por enfermedad con goce de sueldo<br>2 hh9_work4_s___2 Vacaciones o permiso personal con goce de sueldo<br>3 hh9_work4_s___3 Seguro de salud<br>4 hh9_work4_s___4 Seguro de discapacidad<br>5 hh9_work4_s___5 Plan de jubilación<br>6 hh9_work4_s___6 Otro<br>7 hh9_work4_s___7 No sabe
1054	[hh9_work5_s]<br><br>Show the field ONLY if:<br>[language] = '2' and [hhcount_s] > 8 and [hhcount_s] < 13 and ([hh9_work_s] = '1' or [hh9_work_s] = '2' or [hh9_work2_s] = '1')	Persona 9: En una escala de 0 (definitivamente no va a suceder) a 10 (definitivamente va a suceder), ¿qué tan probable es que esta persona pierda su empleo debido a la pandemia de COVID-19?	text (number, Min: 0, Max: 10)
1055	[hh9_work6_s]<br><br>Show the field ONLY if:<br>[language] = '2' and [hhcount_s] > 8 and [hhcount_s] < 13 and ([hh9_work_s] = '1' or [hh9_work_s] = '2' or [hh9_work2_s] = '1')	Persona 9: En una escala de 0 (definitivamente no va a suceder) a 10 (definitivamente va a suceder), ¿qué tan probable es que a esta persona se le asignen menos horas de trabajo debido a la pandemia de COVID-19?	text (number, Min: 0, Max: 10)
1056	[hh9_work7_s]<br><br>Show the field ONLY if:<br>[language] = '2' and [hhcount_s] > 8 and [hhcount_s] < 13 and ([hh9_work_s] = '1' or [hh9_work_s] = '2' or [hh9_work2_s] = '1')	Persona 9: Actualmente ¿con qué frecuencia se le exige a esta persona que trabaje fuera de su residencia?	radio (Matrix)<br>1 Todo el tiempo (100%)<br>2 la mayor parte del tiempo (75%)<br>3 La mitad del tiempo (50%)<br>4 Menos de la mitad del tiempo (25%)<br>5 Nunca (0%)
1057	[hh9_work8_s]<br><br>Show the field ONLY if:<br>[language] = '2' and ([hh9_work7_s] = '1' or [hh9_work7_s] = '2' or [hh9_work7_s] = '3' or [hh9_work7_s] = '4')	Persona 9: Actualmente ¿con qué frecuencia se encuentra esta persona físicamente cerca de sus compañeros de trabajo mientras trabaja fuera de su residencia?	radio (Matrix)<br>1 Todo el tiempo (100%)<br>2 la mayor parte del tiempo (75%)<br>3 La mitad del tiempo (50%)<br>4 Menos de la mitad del tiempo (25%)<br>5 Nunca (0%)
1058	[hh9_work9_s]<br><br>Show the field ONLY if:<br>[language] = '2' and ([hh9_work7_s] = '1' or [hh9_work7_s] = '2' or [hh9_work7_s] = '3' or [hh9_work7_s] = '4')	Persona 9: Actualmente ¿con qué frecuencia se encuentra esta persona físicamente cerca de sus clientes mientras trabaja fuera de su residencia?	radio (Matrix)<br>1 Todo el tiempo (100%)<br>2 la mayor parte del tiempo (75%)<br>3 La mitad del tiempo (50%)<br>4 Menos de la mitad del tiempo (25%)<br>5 Nunca (0%)
1059	[hh9_covidvaccine_s]<br><br>Show the field ONLY if:	Persona 9: ¿Piensa esta persona vacunarse contra la COVID-19 cuando se ofrezca una vacuna?	radio<br>1 Sí

	[language] = '2' and [hhcount_s] > 8 and [hhcount_s] < 13		0 No<br>2 No sabe
1060	[hh9_covidsymp_s]<br><br>Show the field ONLY if:<br>[language] = '2' and [hhcount_s] > 8 and [hhcount_s] < 13	Persona 9: En las últimas dos semanas, ¿ha tenido esta persona algún síntoma de COVID-19 (tos, fiebre, dificultad para respirar, fatiga, dolores de cuerpo, diarrea, goteo nasal o pérdida del sentido del olfato o del gusto)?	radio<br>1 Sí<br>0 No<br>2 No sabe
1061	[hh9_covidsymp2_s]<br><br>Show the field ONLY if:<br>[language] = '2' and [hh9_covidsymp_s] = '1'	Persona 9: ¿Cuándo le comenzaron los síntomas de COVID-19 a esta persona?	text (date_mdy)
1062	[hh9_covidsymp3_s]<br><br>Show the field ONLY if:<br>[language] = '2' and [hh9_covidsymp_s] = '1'	Persona 9: En vista de sus síntomas, ¿le preocupa a esta persona la posibilidad de tener COVID-19?	radio<br>1 Sí<br>0 No<br>2 No sabe
1063	[hh9_covidsymp4_s]<br><br>Show the field ONLY if:<br>[language] = '2' and [hh9_covidsymp_s] = '1'	Persona 9: ¿Fue esta persona objeto de prejuicio o discriminación debido a sus síntomas?	radio<br>1 Sí<br>0 No<br>2 No sabe
1064	[hh9_covidsymp5_s]<br><br>Show the field ONLY if:<br>[language] = '2' and [hh9_covidsymp_s] = '1'	Persona 9: ¿Qué hizo esta persona en vista de sus síntomas?<br>*(Seleccione todo lo que corresponda.)*	checkbox<br>0 hh9_covidsymp5_s___0 Nada<br>1 hh9_covidsymp5_s___1 Tomó medicamentos sin receta (ibuprofeno, acetaminofén, etc.)<br>2 hh9_covidsymp5_s___2 Consultó por teléfono a un proveedor de asistencia médica<br>3 hh9_covidsymp5_s___3 Fue al consultorio de un proveedor de asistencia médica<br>4 hh9_covidsymp5_s___4 Fue a una clínica o una farmacia minorista<br>5 hh9_covidsymp5_s___5 Fue a un centro de urgencias (FASTMed, etc.)<br>6 hh9_covidsymp5_s___6 Fue a la sala de emergencias<br>7 hh9_covidsymp5_s___7 Fue ingresado al hospital<br>8 hh9_covidsymp5_s___8 Otra cosa<br>9 hh9_covidsymp5_s___9 No sabe

1065	[hh9_covidsymp6_s]<br><br>Show the field ONLY if:<br>[language] = '2' and [hh9_covidsymp5_s(8)] = '1'	Persona 9 ¿Qué otra cosa hizo esta persona en vista de sus síntomas?	text
1066	[hh9_covidsymp7_s]<br><br>Show the field ONLY if:<br>[language] = '2' and ([hh9_covidsymp5_s(2)] = '1' or [hh9_covidsymp5_s(3)] = '1' or [hh9_covidsymp5_s(4)] = '1' or [hh9_covidsymp5_s(5)] = '1' or [hh9_covidsymp5_s(6)] = '1' or [hh9_covidsymp5_s(7)] = '1' or [hh9_covidsymp5_s(8)] = '1')	Persona 9: ¿Le dijo un proveedor de asistencia médica a esta persona que era posible que tuviera COVID-19?	radio<br>1 Sí<br>0 No<br>2 No sabe
1067	[hh9_covid_test_s]<br><br>Show the field ONLY if:<br>[language] = '2' and [hh9_covidsymp_s] = '1'	Persona 9: Si a esta persona le hicieron una prueba de COVID-19 en vista de sus síntomas, ¿cuál fue el resultado?	radio<br>1 Pendiente<br>2 Positivo<br>3 Negativo<br>4 No concluyente<br>5 No se hizo la prueba<br>6 No sabe
1068	[hh9_covid_admit_s]<br><br>Show the field ONLY if:<br>[language] = '2' and [hh9_covidsymp5_s(7)] = '1'	Persona 9: ¿Cuántos días estuvo hospitalizada esta persona?	text (number, Min: 0)
1069	[hh9_covid_admit2_s]<br><br>Show the field ONLY if:<br>[language] = '2' and [hh9_covidsymp5_s(7)] = '1'	Persona 9: ¿Se le hicieron a esta persona las siguientes intervenciones durante su hospitalización?	checkbox<br>1 hh9_covid_admit2_s___1 Oxígeno adicional por la nariz<br>2 hh9_covid_admit2_s___2 Tratamiento en la Unidad de Cuidados Intensivos (Intensive Care Unit, ICU)<br>3 hh9_covid_admit2_s___3 Ventilación mecánica (intubación o tubo de respiración)<br>4 hh9_covid_admit2_s___4 No sabe
1070	[hh9_covidsymp8_s]<br><br>Show the field ONLY if:<br>[language] = '2' and [hh9_covidsymp_s] = '1'	Persona 9: ¿Ha vuelto esta persona a su salud normal?	radio<br>1 Sí<br>0 No<br>2 No sabe
1071	[hh9_prevent_s]<br><br>Show the field ONLY if:<br>[language] = '2' and [hh9_covidsymp_s] = '1'	Persona 9: ¿Cuáles de las siguientes medidas tomó esta persona para proteger a sus amigos y familiares después de que comenzaron sus síntomas?	checkbox<br>1 hh9_prevent_s___1 Ponerse mascarilla con mayor frecuencia

			2 | hh9_prevent_s___2 | Lavarse las manos con agua y jabón con mayor frecuencia<br>3 | hh9_prevent_s___3 | Limpiarse las manos con desinfectante con mayor frecuencia<br>4 | hh9_prevent_s___4 | Aislarse en su casa con mayor frecuencia<br>5 | hh9_prevent_s___5 | Quedarse en casa con mayor frecuencia<br>6 | hh9_prevent_s___6 | Ponerse guantes desechables con mayor frecuencia<br>7 | hh9_prevent_s___7 | No sabe
1072	[hh10_relationship_s]<br>Show the field ONLY if: [language] = '2' and [hhcount_s] > 9 and [hhcount_s] < 13	Section Header: *Por cada persona adicional en su hogar, proporcione la siguiente informacion.*<br>Persona 10: ¿Cuál es su relación con esta persona?	radio<br>1 | Pareja o cónyuge<br>2 | Hijo o hija<br>3 | Padre o madre<br>4 | Hermano o hermana<br>5 | Otra familiar<br>6 | Proveedor de cuidado infantil u otros cuidados en casa<br>7 | Otra
1073	[hh10_relationship2_s]<br>Show the field ONLY if: [language] = '2' and [hh10_relationship_s] = '7'	Persona 10: Especifique su relación con esta persona.	text
1074	[hh10_age_s]<br>Show the field ONLY if: [language] = '2' and [hhcount_s] > 9 and [hhcount_s] < 13	Persona 10: ¿Qué edad tiene esta persona? *(Especifique la edad en años)*	text (number, Min: 0, Max: 110)
1075	[hh10_sex_s]<br>Show the field ONLY if: [language] = '2' and [hhcount_s] > 9 and [hhcount_s] < 13	Persona 10: ¿Cuál es el sexo de esta persona?	radio<br>1 | Femenino<br>2 | Masculino
1076	[hh10_race_s]<br>Show the field ONLY if: [language] = '2' and [hhcount_s] > 9 and [hhcount_s] < 13	Persona 10: ¿Cuál es la raza de esta persona? *(Seleccione todo lo que corresponda.)*	checkbox<br>1 | hh10_race_s___1 | Indio americano o nativo de Alaska<br>2 | hh10_race_s___2 | Asiático<br>3 | hh10_race_s___3 | Negro o afroestadounidense<br>4 | hh10_race_s___4 | Nativo de Hawái o de las islas del Pacífico

			5 hh10_race_s___5 Blanco<br>6 hh10_race_s___6 Otro<br>7 hh10_race_s___7 No sabe
1077	[hh10_ethn_s]<br>Show the field ONLY if:<br>[language] = '2' and [hhcount_s] > 9 and [hhcount_s] < 13	Persona 10: ¿Cuál es la identidad étnica de esta persona?	radio<br>1 Hispano o latino<br>2 Ni hispano ni latino<br>3 Otra<br>4 No sabe
1078	[hh10_edu_s]<br>Show the field ONLY if:<br>[language] = '2' and [hhcount_s] > 9 and [hhcount_s] < 13	Persona 10: ¿Cuál es el nivel de educación de esta persona?	radio<br>1 No tiene educación formal<br>2 Kínder a 8° grado<br>3 Estudios de educación secundaria<br>4 Equivalencia de educación secundaria (GED)<br>5 Diploma de educación secundaria<br>6 Estudios de educación superior<br>7 Título universitario<br>8 Estudios de posgrado o más<br>9 No sabe
1079	[hh10_work_s]<br>Show the field ONLY if:<br>[language] = '2' and [hhcount_s] > 9 and [hhcount_s] < 13	Persona 10: ¿Cuál de las siguientes es la mejor descripción de la situación laboral actual de esta persona?	radio<br>1 Empleo a tiempo completo<br>2 Empleo a tiempo parcial<br>3 En busca de empleo<br>4 Jubilado<br>5 Ama de casa<br>6 Estudiante<br>7 Permiso de maternidad o paternidad<br>8 Permiso por enfermedad<br>9 Desempleado por discapacidad<br>10 Otra<br>11 No sabe
1080	[hh10_work2_s]<br>Show the field ONLY if:<br>[language] = '2' and [hhcount_s] > 9 and [hhcount_s] < 13	Persona 10: ¿Se considera esta persona actualmente empleado por cuenta propia (contratista independiente, trabajador esporádico (gig), etc.)?	radio<br>1 Sí<br>0 No<br>2 No sabe
1081	[hh10_work3_s]<br>Show the field ONLY if:<br>[language] = '2' and [hhcount_s] > 9 and [hhcount_s] < 13 and ([hh10_work_s] = '1' or [hh10_work_s] = '2' or [hh10_work2_s] = '1')	Persona 10: ¿Trabaja esta persona actualmente en alguno de los siguientes ambientes de alto riesgo de contagio de COVID-19?	checkbox<br>1 hh10_work3_s___1 Ambiente de asistencia médica (hospital, clínica, centro de urgencias, etc.)<br>2 hh10_work3_s___2 Ambiente residencial denso (hogar de ancianos, otro

			centro de asistencia de larga duración)<br>3 hh10_work3_s___3 Prisión o cárcel<br>4 hh10_work3_s___4 Establecimiento de envasado de carne<br>5 hh10_work3_s___5 Establecimiento de envío o distribución<br>6 hh10_work3_s___6 Establecimiento minorista de alto volumen (tienda de provisiones, etc.)<br>7 hh10_work3_s___7 No sabe
1082	[hh10_work4_s]<br>Show the field ONLY if:<br>[language] = '2' and [hhcount_s] > 9 and [hhcount_s] < 13 and ([hh10_work_s] = '1' or [hh10_work_s] = '2' or [hh10_work2_s] = '1')	Persona 10: ¿Le ofrece el empleador a esta persona alguno de los siguientes beneficios en su empleo principal actual?<br>*(Seleccione todo lo que corresponda.)*	checkbox<br>1 hh10_work4_s___1 Permiso por enfermedad con goce de sueldo<br>2 hh10_work4_s___2 Vacaciones o permiso personal con goce de sueldo<br>3 hh10_work4_s___3 Seguro de salud<br>4 hh10_work4_s___4 Seguro de discapacidad<br>5 hh10_work4_s___5 Plan de jubilación<br>6 hh10_work4_s___6 Otro<br>7 hh10_work4_s___7 No sabe
1083	[hh10_work5_s]<br>Show the field ONLY if:<br>[language] = '2' and [hhcount_s] > 9 and [hhcount_s] < 13 and ([hh10_work_s] = '1' or [hh10_work_s] = '2' or [hh10_work2_s] = '1')	Persona 10: En una escala de 0 (definitivamente no va a suceder) a 10 (definitivamente va a suceder), ¿qué tan probable es que esta persona pierda su empleo debido a la pandemia de COVID-19?	text (number, Min: 0, Max: 10)
1084	[hh10_work6_s]<br>Show the field ONLY if:<br>[language] = '2' and [hhcount_s] > 9 and [hhcount_s] < 13 and ([hh10_work_s] = '1' or [hh10_work_s] = '2' or [hh10_work2_s] = '1')	Persona 10: En una escala de 0 (definitivamente no va a suceder) a 10 (definitivamente va a suceder), ¿qué tan probable es que a esta persona se le asignen menos horas de trabajo debido a la pandemia de COVID-19?	text (number, Min: 0, Max: 10)
1085	[hh10_work7_s]<br>Show the field ONLY if:<br>[language] = '2' and [hhcount_s] > 9 and [hhcount_s] < 13 and ([hh10_work_s] = '1' or [hh10_work_s] = '2' or [hh10_work2_s] = '1')	Persona 10: Actualmente ¿con qué frecuencia se le exige a esta persona que trabaje fuera de su residencia?	radio (Matrix)<br>1 Todo el tiempo (100%)<br>2 la mayor parte del tiempo (75%)<br>3 La mitad del tiempo (50%)<br>4 Menos de la mitad del tiempo (25%)<br>5 Nunca (0%)
1086	[hh10_work8_s]<br>Show the field ONLY if:	Persona 10: Actualmente ¿con qué frecuencia se encuentra esta persona físicamente cerca de sus	radio (Matrix)<br>1 Todo el tiempo (100%)

	[language] = '2' and ([hh10_work7_s] = '1' or [hh10_work7_s] = '2' or [hh10_work7_s] = '3' or [hh10_work7_s] = '4')	compañeros de trabajo mientras trabaja fuera de su residencia?	2 la mayor parte del tiempo (75%)<br>3 La mitad del tiempo (50%)<br>4 Menos de la mitad del tiempo (25%)<br>5 Nunca (0%)
1087	[hh10_work9_s]<br><br>Show the field ONLY if: [language] = '2' and ([hh10_work7_s] = '1' or [hh10_work7_s] = '2' or [hh10_work7_s] = '3' or [hh10_work7_s] = '4')	Persona 10: Actualmente ¿con qué frecuencia se encuentra esta persona físicamente cerca de sus clientes mientras trabaja fuera de su residencia?	radio (Matrix)<br>1 Todo el tiempo (100%)<br>2 la mayor parte del tiempo (75%)<br>3 La mitad del tiempo (50%)<br>4 Menos de la mitad del tiempo (25%)<br>5 Nunca (0%)
1088	[hh10_covidvaccine_s]<br><br>Show the field ONLY if: [language] = '2' and [hhcount_s] > 9 and [hhcount_s] < 13	Persona 10: ¿Piensa esta persona vacunarse contra la COVID-19 cuando se ofrezca una vacuna?	radio<br>1 Sí<br>0 No<br>2 No sabe
1089	[hh10_covidsymp_s]<br><br>Show the field ONLY if: [language] = '2' and [hhcount_s] > 9 and [hhcount_s] < 13	Persona 10: En las últimas dos semanas, ¿ha tenido esta persona algún síntoma de COVID-19 (tos, fiebre, dificultad para respirar, fatiga, dolores de cuerpo, diarrea, goteo nasal o pérdida del sentido del olfato o del gusto)?	radio<br>1 Sí<br>0 No<br>2 No sabe
1090	[hh10_covidsymp2_s]<br><br>Show the field ONLY if: [language] = '2' and [hh10_covidsymp_s] = '1'	Persona 10: ¿Cuándo le comenzaron los síntomas de COVID-19 a esta persona?	text (date_mdy)
1091	[hh10_covidsymp3_s]<br><br>Show the field ONLY if: [language] = '2' and [hh10_covidsymp_s] = '1'	Persona 10: En vista de sus síntomas, ¿le preocupa a esta persona la posibilidad de tener COVID-19?	radio<br>1 Sí<br>0 No<br>2 No sabe
1092	[hh10_covidsymp4_s]<br><br>Show the field ONLY if: [language] = '2' and [hh10_covidsymp_s] = '1'	Persona 10: ¿Fue esta persona objeto de prejuicio o discriminación debido a sus síntomas?	radio<br>1 Sí<br>0 No<br>2 No sabe
1093	[hh10_covidsymp5_s]<br><br>Show the field ONLY if: [language] = '2' and [hh10_covidsymp_s] = '1'	Persona 10: ¿Qué hizo esta persona en vista de sus síntomas?<br>*(Seleccione todo lo que corresponda.)*	checkbox<br>0 hh10_covidsymp5_s___0 Nada<br>1 hh10_covidsymp5_s___1 Tomó medicamentos sin receta (ibuprofeno, acetaminofén, etc.)<br>2 hh10_covidsymp5_s___2 Consultó por teléfono a un proveedor de asistencia médica<br>3 hh10_covidsymp5_s___3 Fue al consultorio de un proveedor

			de asistencia médica
		4 hh10_covidsymp5_s___4	Fue a una clínica o una farmacia minorista
		5 hh10_covidsymp5_s___5	Fue a un centro de urgencias (FASTMed, etc.)
		6 hh10_covidsymp5_s___6	Fue a la sala de emergencias
		7 hh10_covidsymp5_s___7	Fue ingresado al hospital
		8 hh10_covidsymp5_s___8	Otra cosa
		9 hh10_covidsymp5_s___9	No sabe
1094	[hh10_covidsymp6_s]<br>Show the field ONLY if: [language] = '2' and [hh10_covidsymp5_s(8)] = '1'	Persona 10 ¿Qué otra cosa hizo esta persona en vista de sus síntomas?	text
1095	[hh10_covidsymp7_s]<br>Show the field ONLY if: [language] = '2' and ([hh10_covidsymp5_s(2)] = '1' or [hh10_covidsymp5_s(3)] = '1' or [hh10_covidsymp5_s(4)] = '1' or [hh10_covidsymp5_s(5)] = '1' or [hh10_covidsymp5_s(6)] = '1' or [hh10_covidsymp5_s(7)] = '1' or [hh10_covidsymp5_s(8)] = '1')	Persona 10: ¿Le dijo un proveedor de asistencia médica a esta persona que era posible que tuviera COVID-19?	radio<br>1 Sí<br>0 No<br>2 No sabe
1096	[hh10_covid_test_s]<br>Show the field ONLY if: [language] = '2' and [hh10_covidsymp_s] = '1'	Persona 10: Si a esta persona le hicieron una prueba de COVID-19 en vista de sus síntomas, ¿cuál fue el resultado?	radio<br>1 Pendiente<br>2 Positivo<br>3 Negativo<br>4 No concluyente<br>5 No se hizo la prueba<br>6 No sabe
1097	[hh10_covid_admit_s]<br>Show the field ONLY if: [language] = '2' and [hh10_covidsymp5_s(7)] = '1'	Persona 10: ¿Cuántos días estuvo hospitalizada esta persona?	text (number, Min: 0)
1098	[hh10_covid_admit2_s]<br>Show the field ONLY if: [language] = '2' and [hh10_covidsymp5_s(7)] = '1'	Persona 10: ¿Se le hicieron a esta persona las siguientes intervenciones durante su hospitalización?	checkbox<br>1 hh10_covid_admit2_s___1 Oxígeno adicional por la nariz<br>2 hh10_covid_admit2_s___2 Tratamiento en la Unidad de Cuidados Intensivos (Intensive

			Care Unit, ICU)<br>3 hh10_covid_admit2_s___3 Ventilación mecánica (intubación o tubo de respiración)<br>4 hh10_covid_admit2_s___4 No sabe
1099	[hh10_covidsymp8_s]<br>Show the field ONLY if: [language] = '2' and [hh10_covidsymp_s] = '1'	Persona 10: ¿Ha vuelto esta persona a su salud normal?	radio<br>1 Sí<br>0 No<br>2 No sabe
1100	[hh10_prevent_s]<br>Show the field ONLY if: [language] = '2' and [hh10_covidsymp_s] = '1'	Persona 10: ¿Cuáles de las siguientes medidas tomó esta persona para proteger a sus amigos y familiares después de que comenzaron sus síntomas?	checkbox<br>1 hh10_prevent_s___1 Ponerse mascarilla con mayor frecuencia<br>2 hh10_prevent_s___2 Lavarse las manos con agua y jabón con mayor frecuencia<br>3 hh10_prevent_s___3 Limpiarse las manos con desinfectante con mayor frecuencia<br>4 hh10_prevent_s___4 Aislarse en su casa con mayor frecuencia<br>5 hh10_prevent_s___5 Quedarse en casa con mayor frecuencia<br>6 hh10_prevent_s___6 Ponerse guantes desechables con mayor frecuencia<br>7 hh10_prevent_s___7 No sabe
1101	[hh11_relationship_s]<br>Show the field ONLY if: [language] = '2' and [hhcount_s] > 10 and [hhcount_s] < 13	Section Header: *Por cada persona adicional en su hogar, proporcione la siguiente informacion.*<br>Persona 11: ¿Cuál es su relación con esta persona?	radio<br>1 Pareja o cónyuge<br>2 Hijo o hija<br>3 Padre o madre<br>4 Hermano o hermana<br>5 Otra familiar<br>6 Proveedor de cuidado infantil u otros cuidados en casa<br>7 Otra
1102	[hh11_relationship2_s]<br>Show the field ONLY if: [language] = '2' and [hh11_relationship_s] = '7'	Persona 11: Especifique su relación con esta persona.	text
1103	[hh11_age_s]	Persona 11: ¿Qué edad tiene esta persona?<br>*(Especifique la edad en años)*	text (number, Min: 0, Max: 110)

	Show the field ONLY if: [language] = '2' and [hhcount_s] > 10 and [hhcount_s] < 13		
1104	[hh11_sex_s] Show the field ONLY if: [language] = '2' and [hhcount_s] > 10 and [hhcount_s] < 13	Persona 11: ¿Cuál es el sexo de esta persona?	radio<br>1 Femenino<br>2 Masculino
1105	[hh11_race_s] Show the field ONLY if: [language] = '2' and [hhcount_s] > 10 and [hhcount_s] < 13	Persona 11: ¿Cuál es la raza de esta persona? *(Seleccione todo lo que corresponda.)*	checkbox<br>1 hh11_race_s___1 Indio americano o nativo de Alaska<br>2 hh11_race_s___2 Asiático<br>3 hh11_race_s___3 Negro o afroestadounidense<br>4 hh11_race_s___4 Nativo de Hawái o de las islas del Pacífico<br>5 hh11_race_s___5 Blanco<br>6 hh11_race_s___6 Otro<br>7 hh11_race_s___7 No sabe
1106	[hh11_ethn_s] Show the field ONLY if: [language] = '2' and [hhcount_s] > 10 and [hhcount_s] < 13	Persona 11: ¿Cuál es la identidad étnica de esta persona?	radio<br>1 Hispano o latino<br>2 Ni hispano ni latino<br>3 Otra<br>4 No sabe
1107	[hh11_edu_s] Show the field ONLY if: [language] = '2' and [hhcount_s] > 10 and [hhcount_s] < 13	Persona 11: ¿Cuál es el nivel de educación de esta persona?	radio<br>1 No tiene educación formal<br>2 Kínder a 8° grado<br>3 Estudios de educación secundaria<br>4 Equivalencia de educación secundaria (GED)<br>5 Diploma de educación secundaria<br>6 Estudios de educación superior<br>7 Título universitario<br>8 Estudios de posgrado o más<br>9 No sabe
1108	[hh11_work_s] Show the field ONLY if: [language] = '2' and [hhcount_s] > 10 and [hhcount_s] < 13	Persona 11: ¿Cuál de las siguientes es la mejor descripción de la situación laboral actual de esta persona?	radio<br>1 Empleo a tiempo completo<br>2 Empleo a tiempo parcial<br>3 En busca de empleo<br>4 Jubilado<br>5 Ama de casa<br>6 Estudiante<br>7 Permiso de maternidad o paternidad<br>8 Permiso por enfermedad

			9 Desempleado por discapacidad<br>10 Otra<br>11 No sabe
1109	[hh11_work2_s]<br><br>Show the field ONLY if: [language] = '2' and [hhcount_s] > 10 and [hhcount_s] < 13	Persona 11: ¿Se considera esta persona actualmente empleado por cuenta propia (contratista independiente, trabajador esporádico (gig), etc.)?	radio<br>1 Sí<br>0 No<br>2 No sabe
1110	[hh11_work3_s]<br><br>Show the field ONLY if: [language] = '2' and [hhcount_s] > 10 and [hhcount_s] < 13 and ([hh11_work_s] = '1' or [hh11_work_s] = '2' or [hh11_work2_s] = '1')	Persona 11: ¿Trabaja esta persona actualmente en alguno de los siguientes ambientes de alto riesgo de contagio de COVID-19?	checkbox<br>1 hh11_work3_s___1 Ambiente de asistencia médica (hospital, clínica, centro de urgencias, etc.)<br>2 hh11_work3_s___2 Ambiente residencial denso (hogar de ancianos, otro centro de asistencia de larga duración)<br>3 hh11_work3_s___3 Prisión o cárcel<br>4 hh11_work3_s___4 Establecimiento de envasado de carne<br>5 hh11_work3_s___5 Establecimiento de envío o distribución<br>6 hh11_work3_s___6 Establecimiento minorista de alto volumen (tienda de provisiones, etc.)<br>7 hh11_work3_s___7 No sabe
1111	[hh11_work4_s]<br><br>Show the field ONLY if: [language] = '2' and [hhcount_s] > 10 and [hhcount_s] < 13 and ([hh11_work_s] = '1' or [hh11_work_s] = '2' or [hh11_work2_s] = '1')	Persona 11: ¿Le ofrece el empleador a esta persona alguno de los siguientes beneficios en su empleo principal actual?<br>*(Seleccione todo lo que corresponda.)*	checkbox<br>1 hh11_work4_s___1 Permiso por enfermedad con goce de sueldo<br>2 hh11_work4_s___2 Vacaciones o permiso personal con goce de sueldo<br>3 hh11_work4_s___3 Seguro de salud<br>4 hh11_work4_s___4 Seguro de discapacidad<br>5 hh11_work4_s___5 Plan de jubilación<br>6 hh11_work4_s___6 Otro<br>7 hh11_work4_s___7 No sabe
1112	[hh11_work5_s]<br><br>Show the field ONLY if: [language] = '2' and [hhcount_s] > 10 and [hhcount_s] < 13 and ([hh11_work_s] = '1' or [hh11_work_s] = '2' or [hh11_work2_s] = '1')	Persona 11: En una escala de 0 (definitivamente no va a suceder) a 10 (definitivamente va a suceder), ¿qué tan probable es que esta persona pierda su empleo debido a la pandemia de COVID-19?	text (number, Min: 0, Max: 10)

1113	[hh11_work6_s]<br><br>Show the field ONLY if: [language] = '2' and [hhcount_s] > 10 and [hhcount_s] < 13 and ([hh11_work_s] = '1' or [hh11_work_s] = '2' or [hh11_work2_s] = '1')	Persona 11: En una escala de 0 (definitivamente no va a suceder) a 10 (definitivamente va a suceder), ¿qué tan probable es que a esta persona se le asignen menos horas de trabajo debido a la pandemia de COVID-19?	text (number, Min: 0, Max: 10)
1114	[hh11_work7_s]<br><br>Show the field ONLY if: [language] = '2' and [hhcount_s] > 10 and [hhcount_s] < 13 and ([hh11_work_s] = '1' or [hh11_work_s] = '2' or [hh11_work2_s] = '1')	Persona 11: Actualmente ¿con qué frecuencia se le exige a esta persona que trabaje fuera de su residencia?	radio (Matrix)<br>1 Todo el tiempo (100%)<br>2 la mayor parte del tiempo (75%)<br>3 La mitad del tiempo (50%)<br>4 Menos de la mitad del tiempo (25%)<br>5 Nunca (0%)
1115	[hh11_work8_s]<br><br>Show the field ONLY if: [language] = '2' and ([hh11_work7_s] = '1' or [hh11_work7_s] = '2' or [hh11_work7_s] = '3' or [hh11_work7_s] = '4')	Persona 11: Actualmente ¿con qué frecuencia se encuentra esta persona físicamente cerca de sus compañeros de trabajo mientras trabaja fuera de su residencia?	radio (Matrix)<br>1 Todo el tiempo (100%)<br>2 la mayor parte del tiempo (75%)<br>3 La mitad del tiempo (50%)<br>4 Menos de la mitad del tiempo (25%)<br>5 Nunca (0%)
1116	[hh11_work9_s]<br><br>Show the field ONLY if: [language] = '2' and ([hh11_work7_s] = '1' or [hh11_work7_s] = '2' or [hh11_work7_s] = '3' or [hh11_work7_s] = '4')	Persona 11: Actualmente ¿con qué frecuencia se encuentra esta persona físicamente cerca de sus clientes mientras trabaja fuera de su residencia?	radio (Matrix)<br>1 Todo el tiempo (100%)<br>2 la mayor parte del tiempo (75%)<br>3 La mitad del tiempo (50%)<br>4 Menos de la mitad del tiempo (25%)<br>5 Nunca (0%)
1117	[hh11_covidvaccine_s]<br><br>Show the field ONLY if: [language] = '2' and [hhcount_s] > 10 and [hhcount_s] < 13	Persona 11: ¿Piensa esta persona vacunarse contra la COVID-19 cuando se ofrezca una vacuna?	radio<br>1 Sí<br>0 No<br>2 No sabe
1118	[hh11_covidsymp_s]<br><br>Show the field ONLY if: [language] = '2' and [hhcount_s] > 10 and [hhcount_s] < 13	Persona 11: En las últimas dos semanas, ¿ha tenido esta persona algún síntoma de COVID-19 (tos, fiebre, dificultad para respirar, fatiga, dolores de cuerpo, diarrea, goteo nasal o pérdida del sentido del olfato o del gusto)?	radio<br>1 Sí<br>0 No<br>2 No sabe
1119	[hh11_covidsymp2_s]<br><br>Show the field ONLY if: [language] = '2' and [hh11_covidsymp_s] = '1'	Persona 11: ¿Cuándo le comenzaron los síntomas de COVID-19 a esta persona?	text (date_mdy)
1120	[hh11_covidsymp3_s]<br><br>Show the field ONLY if: [language] = '2' and [hh11_covidsymp_s] = '1'	Persona 11: En vista de sus síntomas, ¿le preocupa a esta persona la posibilidad de tener COVID-19?	radio<br>1 Sí<br>0 No<br>2 No sabe
1121	[hh11_covidsymp4_s]<br><br>Show the field ONLY if:	Persona 11: ¿Fue esta persona objeto de prejuicio o discriminación debido a sus síntomas?	radio<br>1 Sí

	[language] = '2' and [hh11_covidsymp_s] = '1'		0 No<br>2 No sabe
1122	[hh11_covidsymp5_s]<br><br>Show the field ONLY if:<br>[language] = '2' and [hh11_covidsymp_s] = '1'	Persona 11: ¿Qué hizo esta persona en vista de sus síntomas?<br>*(Seleccione todo lo que corresponda.)*	checkbox<br>0 hh11_covidsymp5_s___0 Nada<br>1 hh11_covidsymp5_s___1 Tomó medicamentos sin receta (ibuprofeno, acetaminofén, etc.)<br>2 hh11_covidsymp5_s___2 Consultó por teléfono a un proveedor de asistencia médica<br>3 hh11_covidsymp5_s___3 Fue al consultorio de un proveedor de asistencia médica<br>4 hh11_covidsymp5_s___4 Fue a una clínica o una farmacia minorista<br>5 hh11_covidsymp5_s___5 Fue a un centro de urgencias (FASTMed, etc.)<br>6 hh11_covidsymp5_s___6 Fue a la sala de emergencias<br>7 hh11_covidsymp5_s___7 Fue ingresado al hospital<br>8 hh11_covidsymp5_s___8 Otra cosa<br>9 hh11_covidsymp5_s___9 No sabe
1123	[hh11_covidsymp6_s]<br><br>Show the field ONLY if:<br>[language] = '2' and [hh11_covidsymp5_s(8)] = '1'	Persona 11 ¿Qué otra cosa hizo esta persona en vista de sus síntomas?	text
1124	[hh11_covidsymp7_s]<br><br>Show the field ONLY if:<br>[language] = '2' and ([hh11_covidsymp5_s(2)] = '1' or [hh11_covidsymp5_s(3)] = '1' or [hh11_covidsymp5_s(4)] = '1' or [hh11_covidsymp5_s(5)] = '1' or [hh11_covidsymp5_s(6)] = '1' or [hh11_covidsymp5_s(7)] = '1' or [hh11_covidsymp5_s(8)] = '1')	Persona 11: ¿Le dijo un proveedor de asistencia médica a esta persona que era posible que tuviera COVID-19?	radio<br>1 Sí<br>0 No<br>2 No sabe
1125	[hh11_covid_test_s]<br><br>Show the field ONLY if:	Persona 11: Si a esta persona le hicieron una prueba de COVID-19 en vista de sus síntomas, ¿cuál fue el resultado?	radio<br>1 Pendiente<br>2 Positivo

	[language] = '2' and [hh11_covidsymp_s] = '1'		3 Negativo<br>4 No concluyente<br>5 No se hizo la prueba<br>6 No sabe
1126	[hh11_covid_admit_s]<br><br>Show the field ONLY if: [language] = '2' and [hh11_covidsymp5_s(7)] = '1'	Persona 11: ¿Cuántos días estuvo hospitalizada esta persona?	text (number, Min: 0)
1127	[hh11_covid_admit2_s]<br><br>Show the field ONLY if: [language] = '2' and [hh11_covidsymp5_s(7)] = '1'	Persona 11: ¿Se le hicieron a esta persona las siguientes intervenciones durante su hospitalización?	checkbox<br>1 hh11_covid_admit2_s___1 Oxígeno adicional por la nariz<br>2 hh11_covid_admit2_s___2 Tratamiento en la Unidad de Cuidados Intensivos (Intensive Care Unit, ICU)<br>3 hh11_covid_admit2_s___3 Ventilación mecánica (intubación o tubo de respiración)<br>4 hh11_covid_admit2_s___4 No sabe
1128	[hh11_covidsymp8_s]<br><br>Show the field ONLY if: [language] = '2' and [hh11_covidsymp_s] = '1'	Persona 11: ¿Ha vuelto esta persona a su salud normal?	radio<br>1 Sí<br>0 No<br>2 No sabe
1129	[hh11_prevent_s]<br><br>Show the field ONLY if: [language] = '2' and [hh11_covidsymp_s] = '1'	Persona 11: ¿Cuáles de las siguientes medidas tomó esta persona para proteger a sus amigos y familiares después de que comenzaron sus síntomas?	checkbox<br>1 hh11_prevent_s___1 Ponerse mascarilla con mayor frecuencia<br>2 hh11_prevent_s___2 Lavarse las manos con agua y jabón con mayor frecuencia<br>3 hh11_prevent_s___3 Limpiarse las manos con desinfectante con mayor frecuencia<br>4 hh11_prevent_s___4 Aislarse en su casa con mayor frecuencia<br>5 hh11_prevent_s___5 Quedarse en casa con mayor frecuencia<br>6 hh11_prevent_s___6 Ponerse guantes desechables con mayor frecuencia<br>7 hh11_prevent_s___7 No sabe

1130	[hh12_relationship_s]<br>Show the field ONLY if:<br>[language] = '2' and [hhcount_s] > 11 and [hhcount_s] < 13	Section Header: *Por cada persona adicional en su hogar, proporcione la siguiente informacion.*<br>Persona 12: ¿Cuál es su relación con esta persona?	radio<br>1 Pareja o cónyuge<br>2 Hijo o hija<br>3 Padre o madre<br>4 Hermano o hermana<br>5 Otra familiar<br>6 Proveedor de cuidado infantil u otros cuidados en casa<br>7 Otra
1131	[hh12_relationship2_s]<br>Show the field ONLY if:<br>[language] = '2' and [hh12_relationship_s] = '7'	Persona 12: Especifique su relación con esta persona.	text
1132	[hh12_age_s]<br>Show the field ONLY if:<br>[language] = '2' and [hhcount_s] > 11 and [hhcount_s] < 13	Persona 12: ¿Qué edad tiene esta persona?<br>*(Especifique la edad en años)*	text (number, Min: 0, Max: 110)
1133	[hh12_sex_s]<br>Show the field ONLY if:<br>[language] = '2' and [hhcount_s] > 11 and [hhcount_s] < 13	Persona 12: ¿Cuál es el sexo de esta persona?	radio<br>1 Femenino<br>2 Masculino
1134	[hh12_race_s]<br>Show the field ONLY if:<br>[language] = '2' and [hhcount_s] > 11 and [hhcount_s] < 13	Persona 12: ¿Cuál es la raza de esta persona?<br>*(Seleccione todo lo que corresponda.)*	checkbox<br>1 hh12_race_s___1 Indio americano o nativo de Alaska<br>2 hh12_race_s___2 Asiático<br>3 hh12_race_s___3 Negro o afroestadounidense<br>4 hh12_race_s___4 Nativo de Hawái o de las islas del Pacífico<br>5 hh12_race_s___5 Blanco<br>6 hh12_race_s___6 Otro<br>7 hh12_race_s___7 No sabe
1135	[hh12_ethn_s]<br>Show the field ONLY if:<br>[language] = '2' and [hhcount_s] > 11 and [hhcount_s] < 13	Persona 12: ¿Cuál es la identidad étnica de esta persona?	radio<br>1 Hispano o latino<br>2 Ni hispano ni latino<br>3 Otra<br>4 No sabe
1136	[hh12_edu_s]<br>Show the field ONLY if:<br>[language] = '2' and [hhcount_s] > 11 and [hhcount_s] < 13	Persona 12: ¿Cuál es el nivel de educación de esta persona?	radio<br>1 No tiene educación formal<br>2 Kínder a 8° grado<br>3 Estudios de educación secundaria<br>4 Equivalencia de educación secundaria (GED)

			5 Diploma de educación secundaria<br>6 Estudios de educación superior<br>7 Título universitario<br>8 Estudios de posgrado o más<br>9 No sabe
1137	[hh12_work_s]<br><br>Show the field ONLY if:<br>[language] = '2' and [hhcount_s] > 11 and [hhcount_s] < 13	Persona 12: ¿Cuál de las siguientes es la mejor descripción de la situación laboral actual de esta persona?	radio<br>1 Empleo a tiempo completo<br>2 Empleo a tiempo parcial<br>3 En busca de empleo<br>4 Jubilado<br>5 Ama de casa<br>6 Estudiante<br>7 Permiso de maternidad o paternidad<br>8 Permiso por enfermedad<br>9 Desempleado por discapacidad<br>10 Otra<br>11 No sabe
1138	[hh12_work2_s]<br><br>Show the field ONLY if:<br>[language] = '2' and [hhcount_s] > 11 and [hhcount_s] < 13	Persona 12: ¿Se considera esta persona actualmente empleado por cuenta propia (contratista independiente, trabajador esporádico (gig), etc.)?	radio<br>1 Sí<br>0 No<br>2 No sabe
1139	[hh12_work3_s]<br><br>Show the field ONLY if:<br>[language] = '2' and [hhcount_s] > 11 and [hhcount_s] < 13 and ([hh12_work_s] = '1' or [hh12_work_s] = '2' or [hh12_work2_s] = '1')	Persona 12: ¿Trabaja esta persona actualmente en alguno de los siguientes ambientes de alto riesgo de contagio de COVID-19?	checkbox<br>1 hh12_work3_s___1 Ambiente de asistencia médica (hospital, clínica, centro de urgencias, etc.)<br>2 hh12_work3_s___2 Ambiente residencial denso (hogar de ancianos, otro centro de asistencia de larga duración)<br>3 hh12_work3_s___3 Prisión o cárcel<br>4 hh12_work3_s___4 Establecimiento de envasado de carne<br>5 hh12_work3_s___5 Establecimiento de envío o distribución<br>6 hh12_work3_s___6 Establecimiento minorista de alto volumen (tienda de provisiones, etc.)<br>7 hh12_work3_s___7 No sabe
1140	[hh12_work4_s]<br><br>Show the field ONLY if:<br>[language] = '2' and [hhcount_s] > 11 and [hhcount_s] < 13 and ([hh12_work	Persona 12: ¿Le ofrece el empleador a esta persona alguno de los siguientes beneficios en su empleo principal actual?<br>*(Seleccione todo lo que corresponda.)*	checkbox<br>1 hh12_work4_s___1 Permiso por enfermedad con goce de sueldo

	_s] = '1' or [hh12_work_s] = '2' or [hh12_work2_s] = '1')		2 hh12_work4_s___2 Vacaciones o permiso personal con goce de sueldo<br>3 hh12_work4_s___3 Seguro de salud<br>4 hh12_work4_s___4 Seguro de discapacidad<br>5 hh12_work4_s___5 Plan de jubilación<br>6 hh12_work4_s___6 Otro<br>7 hh12_work4_s___7 No sabe
1141	[hh12_work5_s]<br><br>Show the field ONLY if: [language] = '2' and [hhcount_s] > 11 and [hhcount_s] < 13 and ([hh12_work_s] = '1' or [hh12_work_s] = '2' or [hh12_work2_s] = '1')	Persona 12: En una escala de 0 (definitivamente no va a suceder) a 10 (definitivamente va a suceder), ¿qué tan probable es que esta persona pierda su empleo debido a la pandemia de COVID-19?	text (number, Min: 0, Max: 10)
1142	[hh12_work6_s]<br><br>Show the field ONLY if: [language] = '2' and [hhcount_s] > 11 and [hhcount_s] < 13 and ([hh12_work_s] = '1' or [hh12_work_s] = '2' or [hh12_work2_s] = '1')	Persona 12: En una escala de 0 (definitivamente no va a suceder) a 10 (definitivamente va a suceder), ¿qué tan probable es que a esta persona se le asignen menos horas de trabajo debido a la pandemia de COVID-19?	text (number, Min: 0, Max: 10)
1143	[hh12_work7_s]<br><br>Show the field ONLY if: [language] = '2' and [hhcount_s] > 11 and [hhcount_s] < 13 and ([hh12_work_s] = '1' or [hh12_work_s] = '2' or [hh12_work2_s] = '1')	Persona 12: Actualmente ¿con qué frecuencia se le exige a esta persona que trabaje fuera de su residencia?	radio (Matrix)<br>1 Todo el tiempo (100%)<br>2 la mayor parte del tiempo (75%)<br>3 La mitad del tiempo (50%)<br>4 Menos de la mitad del tiempo (25%)<br>5 Nunca (0%)
1144	[hh12_work8_s]<br><br>Show the field ONLY if: [language] = '2' and ([hh12_work7_s] = '1' or [hh12_work7_s] = '2' or [hh12_work7_s] = '3' or [hh12_work7_s] = '4')	Persona 12: Actualmente ¿con qué frecuencia se encuentra esta persona físicamente cerca de sus compañeros de trabajo mientras trabaja fuera de su residencia?	radio (Matrix)<br>1 Todo el tiempo (100%)<br>2 la mayor parte del tiempo (75%)<br>3 La mitad del tiempo (50%)<br>4 Menos de la mitad del tiempo (25%)<br>5 Nunca (0%)
1145	[hh12_work9_s]<br><br>Show the field ONLY if: [language] = '2' and ([hh12_work7_s] = '1' or [hh12_work7_s] = '2' or [hh12_work7_s] = '3' or [hh12_work7_s] = '4')	Persona 12: Actualmente ¿con qué frecuencia se encuentra esta persona físicamente cerca de sus clientes mientras trabaja fuera de su residencia?	radio (Matrix)<br>1 Todo el tiempo (100%)<br>2 la mayor parte del tiempo (75%)<br>3 La mitad del tiempo (50%)<br>4 Menos de la mitad del tiempo (25%)<br>5 Nunca (0%)

1146	[hh12_covidvaccine_s]<br><br>Show the field ONLY if:<br>[language] = '2' and [hhcount_s] > 11 and [hhcount_s] < 13	Persona 12: ¿Piensa esta persona vacunarse contra la COVID-19 cuando se ofrezca una vacuna?	radio<br>1 Sí<br>0 No<br>2 No sabe
1147	[hh12_covidsymp_s]<br><br>Show the field ONLY if:<br>[language] = '2' and [hhcount_s] > 11 and [hhcount_s] < 13	Persona 12: En las últimas dos semanas, ¿ha tenido esta persona algún síntoma de COVID-19 (tos, fiebre, dificultad para respirar, fatiga, dolores de cuerpo, diarrea, goteo nasal o pérdida del sentido del olfato o del gusto)?	radio<br>1 Sí<br>0 No<br>2 No sabe
1148	[hh12_covidsymp2_s]<br><br>Show the field ONLY if:<br>[language] = '2' and [hh12_covidsymp_s] = '1'	Persona 12: ¿Cuándo le comenzaron los síntomas de COVID-19 a esta persona?	text (date_mdy)
1149	[hh12_covidsymp3_s]<br><br>Show the field ONLY if:<br>[language] = '2' and [hh12_covidsymp_s] = '1'	Persona 12: En vista de sus síntomas, ¿le preocupa a esta persona la posibilidad de tener COVID-19?	radio<br>1 Sí<br>0 No<br>2 No sabe
1150	[hh12_covidsymp4_s]<br><br>Show the field ONLY if:<br>[language] = '2' and [hh12_covidsymp_s] = '1'	Persona 12: ¿Fue esta persona objeto de prejuicio o discriminación debido a sus síntomas?	radio<br>1 Sí<br>0 No<br>2 No sabe
1151	[hh12_covidsymp5_s]<br><br>Show the field ONLY if:<br>[language] = '2' and [hh12_covidsymp_s] = '1'	Persona 12: ¿Qué hizo esta persona en vista de sus síntomas?<br>*(Seleccione todo lo que corresponda.)*	checkbox<br>0 hh12_covidsymp5_s___0 Nada<br>1 hh12_covidsymp5_s___1 Tomó medicamentos sin receta (ibuprofeno, acetaminofén, etc.)<br>2 hh12_covidsymp5_s___2 Consultó por teléfono a un proveedor de asistencia médica<br>3 hh12_covidsymp5_s___3 Fue al consultorio de un proveedor de asistencia médica<br>4 hh12_covidsymp5_s___4 Fue a una clínica o una farmacia minorista<br>5 hh12_covidsymp5_s___5 Fue a un centro de urgencias (FASTMed, etc.)<br>6 hh12_covidsymp5_s___6 Fue a la sala de emergencias

			7 hh12_covidsymp5_s___7 Fue ingresado al hospital<br>8 hh12_covidsymp5_s___8 Otra cosa<br>9 hh12_covidsymp5_s___9 No sabe
1152	[hh12_covidsymp6_s]<br>Show the field ONLY if:<br>[language] = '2' and [hh12_covidsymp5_s(8)] = '1'	Persona 12 ¿Qué otra cosa hizo esta persona en vista de sus síntomas?	text
1153	[hh12_covidsymp7_s]<br>Show the field ONLY if:<br>[language] = '2' and ([hh12_covidsymp5_s(2)] = '1' or [hh12_covidsymp5_s(3)] = '1' or [hh12_covidsymp5_s(4)] = '1' or [hh12_covidsymp5_s(5)] = '1' or [hh12_covidsymp5_s(6)] = '1' or [hh12_covidsymp5_s(7)] = '1' or [hh12_covidsymp5_s(8)] = '1')	Persona 12: ¿Le dijo un proveedor de asistencia médica a esta persona que era posible que tuviera COVID-19?	radio<br>1 Sí<br>0 No<br>2 No sabe
1154	[hh12_covid_test_s]<br>Show the field ONLY if:<br>[language] = '2' and [hh12_covidsymp_s] = '1'	Persona 12: Si a esta persona le hicieron una prueba de COVID-19 en vista de sus síntomas, ¿cuál fue el resultado?	radio<br>1 Pendiente<br>2 Positivo<br>3 Negativo<br>4 No concluyente<br>5 No se hizo la prueba<br>6 No sabe
1155	[hh12_covid_admit_s]<br>Show the field ONLY if:<br>[language] = '2' and [hh12_covidsymp5_s(7)] = '1'	Persona 12: ¿Cuántos días estuvo hospitalizada esta persona?	text (number, Min: 0)
1156	[hh12_covid_admit2_s]<br>Show the field ONLY if:<br>[language] = '2' and [hh12_covidsymp5_s(7)] = '1'	Persona 12: ¿Se le hicieron a esta persona las siguientes intervenciones durante su hospitalización?	checkbox<br>1 hh12_covid_admit2_s___1 Oxígeno adicional por la nariz<br>2 hh12_covid_admit2_s___2 Tratamiento en la Unidad de Cuidados Intensivos (Intensive Care Unit, ICU)<br>3 hh12_covid_admit2_s___3 Ventilación mecánica (intubación o tubo de respiración)<br>4 hh12_covid_admit2_s___4 No sabe
1157	[hh12_covidsymp8_s]<br>Show the field ONLY if:	Persona 12: ¿Ha vuelto esta persona a su salud normal?	radio<br>1 Sí<br>0 No

	[language] = '2' and [hh12_covidsymp_s] = '1'		2 No sabe
1158	[hh12_prevent_s]<br>Show the field ONLY if:<br>[language] = '2' and [hh12_covidsymp_s] = '1'	Persona 12: ¿Cuáles de las siguientes medidas tomó esta persona para proteger a sus amigos y familiares después de que comenzaron sus síntomas?	checkbox<br>1 hh12_prevent_s___1 Ponerse mascarilla con mayor frecuencia<br>2 hh12_prevent_s___2 Lavarse las manos con agua y jabón con mayor frecuencia<br>3 hh12_prevent_s___3 Limpiarse las manos con desinfectante con mayor frecuencia<br>4 hh12_prevent_s___4 Aislarse en su casa con mayor frecuencia<br>5 hh12_prevent_s___5 Quedarse en casa con mayor frecuencia<br>6 hh12_prevent_s___6 Ponerse guantes desechables con mayor frecuencia<br>7 hh12_prevent_s___7 No sabe
1159	[serious1_s]<br>Show the field ONLY if:<br>[language] = '2'	Section Header: *Complete la siguiente informacion sobre su salud mental y bienestar*<br>A su juicio, ¿qué tan grave es para usted personalmente la pandemia de COVID-19 en este momento?	radio<br>1 Muy grave<br>2 Más o menos grave<br>3 No demasiado grave<br>4 Nada grave
1160	[serious2_s]<br>Show the field ONLY if:<br>[language] = '2'	A su juicio, ¿qué tan grave es actualmente la pandemia de COVID-19 para los integrantes de su comunidad?	radio<br>1 Muy grave<br>2 Más o menos grave<br>3 No demasiado grave<br>4 Nada grave
1161	[serious3_s]<br>Show the field ONLY if:<br>[language] = '2'	A su juicio, ¿qué tan grave es actualmente la pandemia de COVID-19 para los habitantes de Estados Unidos?	radio<br>1 Muy grave<br>2 Más o menos grave<br>3 No demasiado grave<br>4 Nada grave
1162	[serious4_s]<br>Show the field ONLY if:<br>[language] = '2'	A su juicio, ¿qué tan grave es actualmente la pandemia de COVID-19 para los habitantes de todo el mundo?	radio<br>1 Muy grave<br>2 Más o menos grave<br>3 No demasiado grave<br>4 Nada grave
1163	[concern1_s]<br>Show the field ONLY if:<br>[language] = '2'	Section Header: *La pandemia de COVID-19 puede cuasar desafios por algunas personas sin importar a que esten infectados. Que tan preocupado/a esta usted sobre cada una de la siguiente?*	radio (Matrix)<br>1 Nada<br>2 No mucho

		Recibir la asistencia médica que necesita (incluyendo la asistencia de salud mental)	3 Un poco<br>4 Mucho
1164	[concern2_s]<br>Show the field ONLY if:<br>[language] = '2'	Tener un lugar para vivir	radio (Matrix)<br>1 Nada<br>2 No mucho<br>3 Un poco<br>4 Mucho
1165	[concern3_s]<br>Show the field ONLY if:<br>[language] = '2'	Poder de interactuar con otras personas	radio (Matrix)<br>1 Nada<br>2 No mucho<br>3 Un poco<br>4 Mucho
1166	[concern4_s]<br>Show the field ONLY if:<br>[language] = '2'	Conseguir comida, agua y otros suministros domésticos	radio (Matrix)<br>1 Nada<br>2 No mucho<br>3 Un poco<br>4 Mucho
1167	[concern5_s]<br>Show the field ONLY if:<br>[language] = '2'	Conseguir medicamentos	radio (Matrix)<br>1 Nada<br>2 No mucho<br>3 Un poco<br>4 Mucho
1168	[concern6_s]<br>Show the field ONLY if:<br>[language] = '2'	Tener transporte para llegar adonde tiene que ir	radio (Matrix)<br>1 Nada<br>2 No mucho<br>3 Un poco<br>4 Mucho
1169	[concern7_s]<br>Show the field ONLY if:<br>[language] = '2'	Cuidar de su familia y a sus amigos	radio (Matrix)<br>1 Nada<br>2 No mucho<br>3 Un poco<br>4 Mucho
1170	[gad7_1_s]<br>Show the field ONLY if:<br>[language] = '2'	Section Header: *Durante las ultimas 2 semanas, que tan seguido ha tenido molestias debido a los siguientes problemas?*<br>Nerviosisimo o ansiedad	radio (Matrix)<br>0 Nunca<br>1 Varios días<br>2 Más de la mitad de los días<br>3 Casi todos los días
1171	[gad7_2_s]<br>Show the field ONLY if:<br>[language] = '2'	Incapacidad de dejar de preocuparse o controlar las preocupaciones	radio (Matrix)<br>0 Nunca<br>1 Varios días<br>2 Más de la mitad de los días

			3 Casi todos los días
1172	[gad7_3_s]<br>Show the field ONLY if:<br>[language] = '2'	Exceso de preocupación por diferentes cosas	radio (Matrix)<br>0 Nunca<br>1 Varios días<br>2 Más de la mitad de los días<br>3 Casi todos los días
1173	[gad7_4_s]<br>Show the field ONLY if:<br>[language] = '2'	Dificultad para tranquilizarse	radio (Matrix)<br>0 Nunca<br>1 Varios días<br>2 Más de la mitad de los días<br>3 Casi todos los días
1174	[gad7_5_s]<br>Show the field ONLY if:<br>[language] = '2'	Tanta inquietud que le es difícil estarse quieto	radio (Matrix)<br>0 Nunca<br>1 Varios días<br>2 Más de la mitad de los días<br>3 Casi todos los días
1175	[gad7_6_s]<br>Show the field ONLY if:<br>[language] = '2'	Facilidad para molestarse o irritarse	radio (Matrix)<br>0 Nunca<br>1 Varios días<br>2 Más de la mitad de los días<br>3 Casi todos los días
1176	[gad7_7_s]<br>Show the field ONLY if:<br>[language] = '2'	Temor de que algo horrible podría pasar	radio (Matrix)<br>0 Nunca<br>1 Varios días<br>2 Más de la mitad de los días<br>3 Casi todos los días
1177	[gad7_8_s]<br>Show the field ONLY if:<br>[language] = '2' and ([gad7_1_s] > 0 or [gad7_2_s] > 0 or [gad7_3_s] > 0 or [gad7_4_s] > 0 or [gad7_5_s] > 0 or [gad7_6_s] > 0 or [gad7_7_s] > 0)	¿Cuánta dificultad le han causado estos problemas para hacer su trabajo, ocuparse de las cosas de su casa o llevarse bien con los demás?	radio<br>0 Nada de dificultad<br>1 Un poco de dificultad<br>2 Bastante dificultad<br>3 Mucha dificultad
1178	[cesd_1_s_b]<br>Show the field ONLY if:<br>[language] = '2'	Section Header: *Durante las ultimas 2 semanas, qu_ tan seguido ha tenido los siguentes sentimientos y comportamientos?*<br>Me ha molestado lo que normalmente no me molesta.	radio (Matrix)<br>0 Rara vez o nunca (menos de 1 día)<br>1 Pocas veces (1-2 días)<br>2 De vez en cuando (3-4 días)<br>3 La mayor parte del tiempo o todo el tiempo (5-7 días)
1179	[cesd_2_s_b]<br>Show the field ONLY if:<br>[language] = '2'	No he tenido ganas de comer; he tenido poco apetito.	radio (Matrix)<br>0 Rara vez o nunca (menos de 1 día)<br>1 Pocas veces (1-2 días)<br>2 De vez en cuando (3-4 días)

			3 La mayor parte del tiempo o todo el tiempo (5-7 días)
1180	[cesd_3_s_b]<br>Show the field ONLY if:<br>[language] = '2'	He sentido que no podía librarme de la tristeza, ni siquiera con la ayuda de mis familiares o amigos.	radio (Matrix)<br>0 Rara vez o nunca (menos de 1 día)<br>1 Pocas veces (1-2 días)<br>2 De vez en cuando (3-4 días)<br>3 La mayor parte del tiempo o todo el tiempo (5-7 días)
1181	[cesd_4_s_b]<br>Show the field ONLY if:<br>[language] = '2'	He sentido que yo soy tan bueno como otras personas.	radio (Matrix)<br>0 Rara vez o nunca (menos de 1 día)<br>1 Pocas veces (1-2 días)<br>2 De vez en cuando (3-4 días)<br>3 La mayor parte del tiempo o todo el tiempo (5-7 días)
1182	[cesd_5_s_b]<br>Show the field ONLY if:<br>[language] = '2'	Me ha costado concentrarme en lo que hacía.	radio (Matrix)<br>0 Rara vez o nunca (menos de 1 día)<br>1 Pocas veces (1-2 días)<br>2 De vez en cuando (3-4 días)<br>3 La mayor parte del tiempo o todo el tiempo (5-7 días)
1183	[cesd_6_s_b]<br>Show the field ONLY if:<br>[language] = '2'	Me he sentido deprimido.	radio (Matrix)<br>0 Rara vez o nunca (menos de 1 día)<br>1 Pocas veces (1-2 días)<br>2 De vez en cuando (3-4 días)<br>3 La mayor parte del tiempo o todo el tiempo (5-7 días)
1184	[cesd_7_s_b]<br>Show the field ONLY if:<br>[language] = '2'	He sentido que todo lo que hacía implicaba un gran esfuerzo.	radio (Matrix)<br>0 Rara vez o nunca (menos de 1 día)<br>1 Pocas veces (1-2 días)<br>2 De vez en cuando (3-4 días)<br>3 La mayor parte del tiempo o todo el tiempo (5-7 días)
1185	[cesd_8_s_b]<br>Show the field ONLY if:<br>[language] = '2'	He tenido esperanza para el futuro.	radio (Matrix)<br>0 Rara vez o nunca (menos de 1 día)<br>1 Pocas veces (1-2 días)<br>2 De vez en cuando (3-4 días)<br>3 La mayor parte del tiempo o todo el tiempo (5-7 días)
1186	[cesd_9_s_b]<br>Show the field ONLY if:<br>[language] = '2'	He pensado que mi vida es un fracaso.	radio (Matrix)<br>0 Rara vez o nunca (menos de 1 día)<br>1 Pocas veces (1-2 días)<br>2 De vez en cuando (3-4 días)<br>3 La mayor parte del tiempo o todo el tiempo (5-7 días)

1187	[cesd_10_s_b]<br>Show the field ONLY if:<br>[language] = '2'	He tenido miedo.	radio (Matrix)<br>0 Rara vez o nunca (menos de 1 día)<br>1 Pocas veces (1-2 días)<br>2 De vez en cuando (3-4 días)<br>3 La mayor parte del tiempo o todo el tiempo (5-7 días)
1188	[cesd_11_s_b]<br>Show the field ONLY if:<br>[language] = '2'	No he dormido bien.	radio (Matrix)<br>0 Rara vez o nunca (menos de 1 día)<br>1 Pocas veces (1-2 días)<br>2 De vez en cuando (3-4 días)<br>3 La mayor parte del tiempo o todo el tiempo (5-7 días)
1189	[cesd_12_s_b]<br>Show the field ONLY if:<br>[language] = '2'	He sido feliz.	radio (Matrix)<br>0 Rara vez o nunca (menos de 1 día)<br>1 Pocas veces (1-2 días)<br>2 De vez en cuando (3-4 días)<br>3 La mayor parte del tiempo o todo el tiempo (5-7 días)
1190	[cesd_13_s_b]<br>Show the field ONLY if:<br>[language] = '2'	He hablado menos de lo habitual.	radio (Matrix)<br>0 Rara vez o nunca (menos de 1 día)<br>1 Pocas veces (1-2 días)<br>2 De vez en cuando (3-4 días)<br>3 La mayor parte del tiempo o todo el tiempo (5-7 días)
1191	[cesd_14_s_b]<br>Show the field ONLY if:<br>[language] = '2'	Me he sentido solo.	radio (Matrix)<br>0 Rara vez o nunca (menos de 1 día)<br>1 Pocas veces (1-2 días)<br>2 De vez en cuando (3-4 días)<br>3 La mayor parte del tiempo o todo el tiempo (5-7 días)
1192	[cesd_15_s_b]<br>Show the field ONLY if:<br>[language] = '2'	Las personas han sido poco amistosas.	radio (Matrix)<br>0 Rara vez o nunca (menos de 1 día)<br>1 Pocas veces (1-2 días)<br>2 De vez en cuando (3-4 días)<br>3 La mayor parte del tiempo o todo el tiempo (5-7 días)
1193	[cesd_16_s_b]<br>Show the field ONLY if:<br>[language] = '2'	He disfrutado de la vida.	radio (Matrix)<br>0 Rara vez o nunca (menos de 1 día)<br>1 Pocas veces (1-2 días)<br>2 De vez en cuando (3-4 días)<br>3 La mayor parte del tiempo o todo el tiempo (5-7 días)
1194	[cesd_17_s_b]<br>Show the field ONLY if:	He tenido episodios de llanto.	radio (Matrix)<br>0 Rara vez o nunca (menos de 1 día)

	[language] = '2'		1 Pocas veces (1-2 días)<br>2 De vez en cuando (3-4 días)<br>3 La mayor parte del tiempo o todo el tiempo (5-7 días)
1195	[cesd_18_s_b]<br>Show the field ONLY if:<br>[language] = '2'	Me he sentido triste.	radio (Matrix)<br>0 Rara vez o nunca (menos de 1 día)<br>1 Pocas veces (1-2 días)<br>2 De vez en cuando (3-4 días)<br>3 La mayor parte del tiempo o todo el tiempo (5-7 días)
1196	[cesd_19_s_b]<br>Show the field ONLY if:<br>[language] = '2'	He sentido que no les agrado a los demás.	radio (Matrix)<br>0 Rara vez o nunca (menos de 1 día)<br>1 Pocas veces (1-2 días)<br>2 De vez en cuando (3-4 días)<br>3 La mayor parte del tiempo o todo el tiempo (5-7 días)
1197	[cesd_20_s_b]<br>Show the field ONLY if:<br>[language] = '2'	No he podido "ponerme en marcha".	radio (Matrix)<br>0 Rara vez o nunca (menos de 1 día)<br>1 Pocas veces (1-2 días)<br>2 De vez en cuando (3-4 días)<br>3 La mayor parte del tiempo o todo el tiempo (5-7 días)
1198	[sample_s]<br>Show the field ONLY if:<br>[language] = '2'	¿Está usted dispuesto a dar una muestra nasal con hisopo indolora y una muestra de sangre indolora para este estudio de COVID-19 en el condado de Chatham?	radio<br>1 Sí<br>0 No
1199	[survey_s]<br>Show the field ONLY if:<br>[language] = '2'	¿Cómo llenó esta encuesta?	radio<br>1 En una computadora (portátil o de escritorio)<br>2 En un dispositivo móvil (tableta o teléfono celular)<br>3 Por teléfono con un entrevistador<br>4 Otra manera
1200	[c4_baseline_complete]	Section Header: *Form Status*<br>Complete?	dropdown<br>0 Incomplete<br>1 Unverified<br>2 Complete

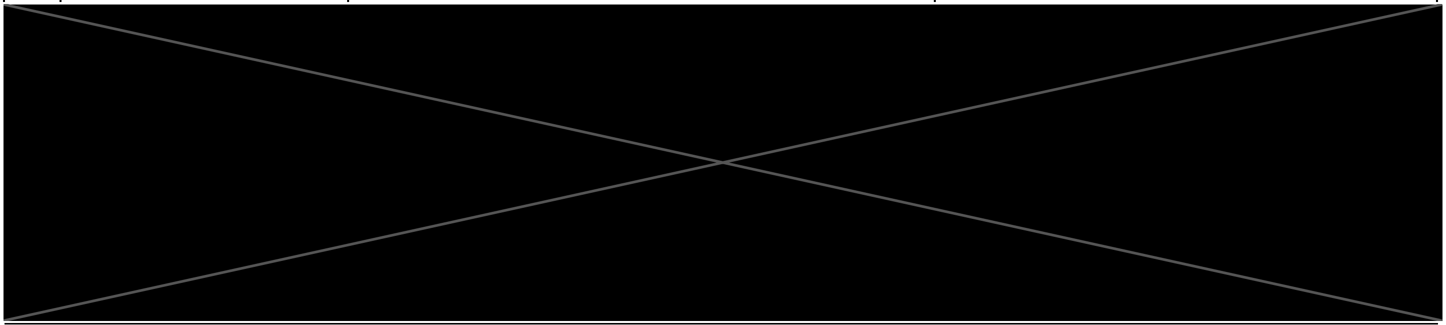

Supplement: S1 File — (PDF) [file pmen.0000410.s003.pdf]
