# Supplementary material for: The association between depressive symptoms and antibody response following SARS-CoV-2 vaccination among central North Carolina residents
Source: PLOS Ment Health. 2025 Sep 17;2(9):e0000410. doi: 10.1371/journal.pmen.0000410 (PMC12448651; doi:10.1371/journal.pmen.0000410)

Data Dictionary Codebook

Aiello - Chatham County COVID-19 Cohort (C4) (PID: 2317)

08/07/2025 9:40am

Instruments	Events

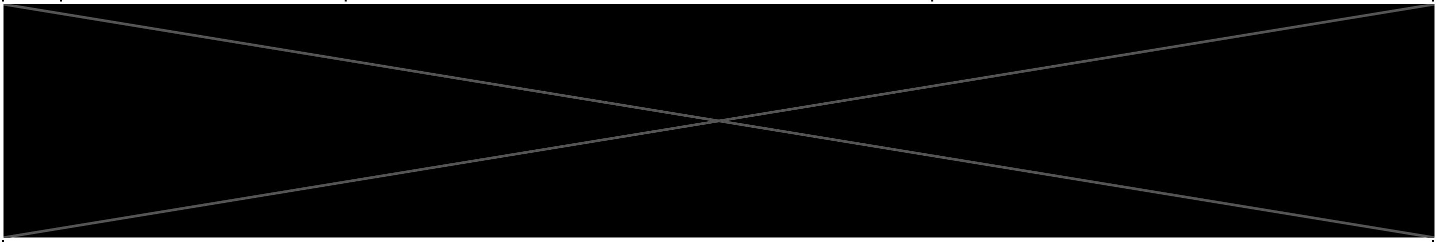

#	Variable / Field Name	Field Label<br>*Field Note*	Field Attributes (Field Type, Validation, Choices, Calculations, etc.)
**Instrument: C4 Q2weeks (c4_q2weeks) Enabled as survey**			
1201	[sruvey_date]	Date of survey completion / Fetcha de finalización de la encuesta:	text (date_mdy)<br>Field Annotation: @NOW
1202	[language_q2]	Would you prefer to take this survey in English or Spanish? / Preferie responder esta encuesta en ingles o espanol?	radio<br>1 English / ingles<br>2 Spanish / espanol<br><br>Field Annotation: @DEFAULT="[language_q2]"
1203	[work7_e_q2]<br><br>Show the field ONLY if:<br>[language_q2] = '1'	Which of the following best fits your current work situation?	radio<br>1 works full time<br>2 works part time<br>3 is looking for work/employment<br>4 retired<br>5 homemaker<br>6 student<br>7 on maternity/paternity leave<br>8 on illness/sick leave<br>9 on disability<br>10 other<br><br>Field Annotation: @DEFAULT="[work7_e_q2]"
1204	[work8_e_q2]<br><br>Show the field ONLY if:<br>[language_q2] = '1'	Do you currently consider yourself self-employed (including as an independent contractor or gig-economy worker)?	radio<br>1 yes<br>0 no<br>2 don't know<br><br>Field Annotation: @DEFAULT="[work8_e_q2]"

1205	[work12_e_q2]<br><br>Show the field ONLY if:<br>[language_q2] = '1' and ([work7_e_q2] = '1' or [work7_e_q2] = '2' or [work8_e_q2] = '1')	On a scale of 0 (definitely not going to happen) to 10 (definitely going to happen), how likely is it that you will lose your job because of the COVID-19 pandemic?	text (number, Min: 0, Max: 10)
1206	[work13_e_q2]<br><br>Show the field ONLY if:<br>[language_q2] = '1' and ([work7_e_q2] = '1' or [work7_e_q2] = '2' or [work8_e_q2] = '1')	On a scale of 0 (definitely not going to happen) to 10 (definitely going to happen), how likely is it that you will receive fewer work hours at your job because of the COVID-19 pandemic?	text (number, Min: 0, Max: 10)
1207	[runout_e_q2]<br><br>Show the field ONLY if:<br>[language_q2] = '1'	On a scale of 0 (definitely not going to happen) to 10 (definitely going to happen), how likely do you think it is that your household will run out of money in the next 3 months?	text (number, Min: 0, Max: 10)
1208	[work24_e_q2]<br><br>Show the field ONLY if:<br>[language_q2] = '1' and ([work7_e_q2] = '1' or [work7_e_q2] = '2' or [work8_e_q2] = '1')	How often are you required to work from outside of the home currently?	radio (Matrix)<br>1 always (100%)<br>2 most of the time (75%)<br>3 half of the time (50%)<br>4 less than half of the time (25%)<br>5 never (0%)
1209	[work25_e_q2]<br><br>Show the field ONLY if:<br>[language_q2] = '1' and ([work24_e_q2] = '1' or [work24_e_q2] = '2' or [work24_e_q2] = '3' or [work24_e_q2] = '4')	How regularly are you in close physical contact with co-workers during your work outside of the home currently?	radio (Matrix)<br>1 always (100%)<br>2 most of the time (75%)<br>3 half of the time (50%)<br>4 less than half of the time (25%)<br>5 never (0%)
1210	[work26_e_q2]<br><br>Show the field ONLY if:<br>[language_q2] = '1' and ([work24_e_q2] = '1' or [work24_e_q2] = '2' or [work24_e_q2] = '3' or [work24_e_q2] = '4')	How regularly are you in close physical contact with clients during your work outside of the home currently?	radio (Matrix)<br>1 always (100%)<br>2 most of the time (75%)<br>3 half of the time (50%)<br>4 less than half of the time (25%)<br>5 never (0%)
1211	[work27_e_q2]<br><br>Show the field ONLY if:<br>[language_q2] = '1' and ([work24_e_q2] = '1' or [work24_e_q2] = '2' or [work24_e_q2] = '3' or [work24_e_q2] = '4')	How often do you have access to disposable gloves during your work outside of the home currently?	radio (Matrix)<br>1 always (100%)<br>2 most of the time (75%)<br>3 half of the time (50%)<br>4 less than half of the time (25%)<br>5 never (0%)
1212	[work28_e_q2]<br><br>Show the field ONLY if:	How often do you have access to a face mask during your work outside of the home currently?	radio (Matrix)<br>1 always (100%)

	[language_q2] = '1' and ([work24_e_q2] = '1' or [work24_e_q2] = '2' or [work24_e_q2] = '3' or [work24_e_q2] = '4')		2 most of the time (75%)<br>3 half of the time (50%)<br>4 less than half of the time (25%)<br>5 never (0%)
1213	[work29_e_q2]<br>Show the field ONLY if:<br>[language_q2] = '1' and ([work24_e_q2] = '1' or [work24_e_q2] = '2' or [work24_e_q2] = '3' or [work24_e_q2] = '4')	How often do you use disposable gloves during your work outside of the home currently?	radio (Matrix)<br>1 always (100%)<br>2 most of the time (75%)<br>3 half of the time (50%)<br>4 less than half of the time (25%)<br>5 never (0%)
1214	[work30_e_q2]<br>Show the field ONLY if:<br>[language_q2] = '1' and ([work24_e_q2] = '1' or [work24_e_q2] = '2' or [work24_e_q2] = '3' or [work24_e_q2] = '4')	How often do you use a face mask during your work outside of the home currently?	radio (Matrix)<br>1 always (100%)<br>2 most of the time (75%)<br>3 half of the time (50%)<br>4 less than half of the time (25%)<br>5 never (0%)
1215	[work31_e_q2]<br>Show the field ONLY if:<br>[language_q2] = '1' and ([work24_e_q2] = '1' or [work24_e_q2] = '2' or [work24_e_q2] = '3' or [work24_e_q2] = '4')	How often do you wash your hands with soap and water during your work outside of the home currently?	radio (Matrix)<br>1 always (100%)<br>2 most of the time (75%)<br>3 half of the time (50%)<br>4 less than half of the time (25%)<br>5 never (0%)
1216	[work32_e_q2]<br>Show the field ONLY if:<br>[language_q2] = '1' and ([work24_e_q2] = '1' or [work24_e_q2] = '2' or [work24_e_q2] = '3' or [work24_e_q2] = '4')	How often do you sanitize your hands with hand sanitizer during your work outside of the home currently?	radio (Matrix)<br>1 always (100%)<br>2 most of the time (75%)<br>3 half of the time (50%)<br>4 less than half of the time (25%)<br>5 never (0%)
1217	[work33_e_q2]<br>Show the field ONLY if:<br>[language_q2] = '1' and ([work24_e_q2] = '1' or [work24_e_q2] = '2' or [work24_e_q2] = '3' or [work24_e_q2] = '4')	How worried are you that you will be exposed to COVID-19 during your work outside of the home currently?	radio (Matrix)<br>1 always (100%)<br>2 most of the time (75%)<br>3 half of the time (50%)<br>4 less than half of the time (25%)<br>5 never (0%)
1218	[work34_e_q2]<br>Show the field ONLY if:<br>[language_q2] = '1' and ([work7_e_q2] = '1' or [work7_e_q2] = '2' or [work8_e_q2] = '1')	Do you currently work in any of the following high-risk settings for COVID-19 transmission?	checkbox<br>1 work34_e_q2___1 healthcare setting (hospital, clinic, urgent care, etc.)<br>2 work34_e_q2___2 dense residential setting (nursing

			home, other long-term care facility)<br>3 | work34_e_q2___3 | prison or jail<br>4 | work34_e_q2___4 | meatpacking facility<br>5 | work34_e_q2___5 | shipping or distribution facility<br>6 | work34_e_q2___6 | high-volume retail facility (grocery store, etc.)<br><br>Field Annotation: @DEFAULT="[work34_e_q2]"
1219	[insur_e_q2]<br><br>Show the field ONLY if:<br>[language_q2] = '1'	Are you covered by any type of medical or health insurance (including private insurance, insurance your purchased, Medicare, Medicaid, or any other health insurance program)?	radio<br>1 | yes<br>0 | no<br>2 | don't know<br><br>Field Annotation: @DEFAULT="[insur_e_q2]"
1220	[insur2_e_q2]<br><br>Show the field ONLY if:<br>[language_q2] = '1' and [insur_e_q2] = '1'	What is the primary health insurance coverage that you have?<br>*Select one (your primary insurance).*	radio<br>1 | Private health insurance through a job or school<br>2 | Insurance purchased through a state or federal health insurance exchange, such as healthcare.gov<br>3 | Insurance purchased directly through a health plan or insurance company<br>4 | Medicare<br>5 | Medi-Gap<br>6 | Medicaid<br>7 | Military health care (TRICARE, VA, CHAMP-VA, etc.)<br>8 | Indian Health Service<br>9 | Other<br><br>Field Annotation: @DEFAULT="[insur2_e_q2]"
1221	[insur3_e_q2]<br><br>Show the field ONLY if:<br>[language_q2] = '1' and [insur2_e_q2] = '9'	Please specify your other source of health insurance	text<br>Field Annotation: @DEFAULT="[insur3_e_q2]"

1222	[pregnant_e_q2]<br><br>Show the field ONLY if:<br>[language_q2] = '1' and [sex] = '1'	Are you currently pregnant?	yesno<br>1 Yes<br>0 No
1223	[srh2_e_q2]<br><br>Show the field ONLY if:<br>[language_q2] = '1'	In general, how would you rate your health over the last two weeks?	radio (Matrix)<br>1 excellent<br>2 very good<br>3 good<br>4 fair<br>5 poor
1224	[prevent_e_q2]<br><br>Show the field ONLY if:<br>[language_q2] = '1'	Section Header: *How often have you done the following things to protect yourself from infection during the last two weeks?*<br>Worn a face mask	radio (Matrix)<br>1 always (100%)<br>2 most of the time (75%)<br>3 half of the time (50%)<br>4 less than half of the time (25%)<br>5 never (0%)
1225	[prevent2_e_q2]<br><br>Show the field ONLY if:<br>[language_q2] = '1'	Washed hands and/or used sanitizer frequently	radio (Matrix)<br>1 always (100%)<br>2 most of the time (75%)<br>3 half of the time (50%)<br>4 less than half of the time (25%)<br>5 never (0%)
1226	[prevent3_e_q2]<br><br>Show the field ONLY if:<br>[language_q2] = '1'	Stayed at least 6 feet away from others	radio (Matrix)<br>1 always (100%)<br>2 most of the time (75%)<br>3 half of the time (50%)<br>4 less than half of the time (25%)<br>5 never (0%)
1227	[prevent4_e_q2]<br><br>Show the field ONLY if:<br>[language_q2] = '1'	Avoided large gatherings, public spaces, or crowds	radio (Matrix)<br>1 always (100%)<br>2 most of the time (75%)<br>3 half of the time (50%)<br>4 less than half of the time (25%)<br>5 never (0%)
1228	[prevent5_e_q2]<br><br>Show the field ONLY if:<br>[language_q2] = '1'	Avoided contact with people who could be high risk	radio (Matrix)<br>1 always (100%)<br>2 most of the time (75%)<br>3 half of the time (50%)<br>4 less than half of the time (25%)

			5 never (0%)
1229	[prevent6_e_q2]<br>Show the field ONLY if:<br>[language_q2] = '1'	Avoided food from restaurants, including takeout	radio (Matrix)<br>1 always (100%)<br>2 most of the time (75%)<br>3 half of the time (50%)<br>4 less than half of the time (25%)<br>5 never (0%)
1230	[prevent7_e_q2]<br>Show the field ONLY if:<br>[language_q2] = '1'	Worked or studied at home instead of going into an office/classroom	radio (Matrix)<br>1 always (100%)<br>2 most of the time (75%)<br>3 half of the time (50%)<br>4 less than half of the time (25%)<br>5 never (0%)
1231	[prevent8_e_q2]<br>Show the field ONLY if:<br>[language_q2] = '1'	Avoided shaking hands or touching people	radio (Matrix)<br>1 always (100%)<br>2 most of the time (75%)<br>3 half of the time (50%)<br>4 less than half of the time (25%)<br>5 never (0%)
1232	[prevent9_e_q2]<br>Show the field ONLY if:<br>[language_q2] = '1'	Stayed home when you were sick	radio (Matrix)<br>1 always (100%)<br>2 most of the time (75%)<br>3 half of the time (50%)<br>4 less than half of the time (25%)<br>5 never (0%)
1233	[prevent10_e_q2]<br>Show the field ONLY if:<br>[language_q2] = '1'	Wiped down surfaces with disinfectant	radio (Matrix)<br>1 always (100%)<br>2 most of the time (75%)<br>3 half of the time (50%)<br>4 less than half of the time (25%)<br>5 never (0%)
1234	[prevent11_e_q2]<br>Show the field ONLY if:<br>[language_q2] = '1'	Cancelled or postponed planned travel for work	radio (Matrix)<br>1 always (100%)<br>2 most of the time (75%)<br>3 half of the time (50%)<br>4 less than half of the time (25%)<br>5 never (0%)
1235	[prevent12_e_q2]	Cancelled or postponed travel for pleasure	radio (Matrix)

	Show the field ONLY if: [language_q2] = '1'		1 always (100%)<br>2 most of the time (75%)<br>3 half of the time (50%)<br>4 less than half of the time (25%)<br>5 never (0%)
1236	[prevent13_e_q2]<br><br>Show the field ONLY if: [language_q2] = '1'	Cancelled or postponed personal or social activities	radio (Matrix)<br>1 always (100%)<br>2 most of the time (75%)<br>3 half of the time (50%)<br>4 less than half of the time (25%)<br>5 never (0%)
1237	[prevent14_e_q2]<br><br>Show the field ONLY if: [language_q2] = '1'	Cancelled a doctor's appointment	radio (Matrix)<br>1 always (100%)<br>2 most of the time (75%)<br>3 half of the time (50%)<br>4 less than half of the time (25%)<br>5 never (0%)
1238	[prevent15_e_q2]<br><br>Show the field ONLY if: [language_q2] = '1'	Stockpiled food or water	radio (Matrix)<br>1 always (100%)<br>2 most of the time (75%)<br>3 half of the time (50%)<br>4 less than half of the time (25%)<br>5 never (0%)
1239	[prevent16_e_q2]<br><br>Show the field ONLY if: [language_q2] = '1'	Followed government guidelines or rules to shelter in place (staying at home, limiting contacts with other people, etc.)	radio (Matrix)<br>1 always (100%)<br>2 most of the time (75%)<br>3 half of the time (50%)<br>4 less than half of the time (25%)<br>5 never (0%)
1240	[fever_e_q2]<br><br>Show the field ONLY if: [language_q2] = '1'	Section Header: *During the last two weeks, have you experienced any of the following symptoms?*<br><br>Fever (measured by thermometer or self-diagnosed)	radio (Matrix)<br>1 yes<br>0 no
1241	[cough_e_q2]<br><br>Show the field ONLY if: [language_q2] = '1'	Cough (new or worsening)	radio (Matrix)<br>1 yes<br>0 no
1242	[sob_e_q2]<br><br>Show the field ONLY if: [language_q2] = '1'	Shortness of breath (new or worsening)	radio (Matrix)<br>1 yes<br>0 no

1243	[fatigue_e_q2]<br><br>Show the field ONLY if:<br>[language_q2] = '1'	Fatigue (new tiredness doing normal activities)	radio (Matrix)<br>1 yes<br>0 no
1244	[bodyache_e_q2]<br><br>Show the field ONLY if:<br>[language_q2] = '1'	Body aches	radio (Matrix)<br>1 yes<br>0 no
1245	[headache_e_q2]<br><br>Show the field ONLY if:<br>[language_q2] = '1'	Headache	radio (Matrix)<br>1 yes<br>0 no
1246	[diarrhea_e_q2]<br><br>Show the field ONLY if:<br>[language_q2] = '1'	Diarrhea	radio (Matrix)<br>1 yes<br>0 no
1247	[pharyngitis_e_q2]<br><br>Show the field ONLY if:<br>[language_q2] = '1'	Sore throat	radio (Matrix)<br>1 yes<br>0 no
1248	[eye_e_q2]<br><br>Show the field ONLY if:<br>[language_q2] = '1'	Itchy, pink, or painful eyes	radio (Matrix)<br>1 yes<br>0 no
1249	[congest_e_q2]<br><br>Show the field ONLY if:<br>[language_q2] = '1'	Runny nose or congestion	radio (Matrix)<br>1 yes<br>0 no
1250	[sensory_e_q2]<br><br>Show the field ONLY if:<br>[language_q2] = '1'	Changes in your sense of smell or taste	radio (Matrix)<br>1 yes<br>0 no
1251	[rash_e_q2]<br><br>Show the field ONLY if:<br>[language_q2] = '1'	New rash	radio (Matrix)<br>1 yes<br>0 no
1252	[chills_e_q2]<br><br>Show the field ONLY if:<br>[language_q2] = '1'	Repeated shaking with chills	radio (Matrix)<br>1 yes<br>0 no
1253	[covid_symp_e_q2]<br><br>Show the field ONLY if:<br>[language_q2] = '1' and ([fever_e_q2] = '1' or [cough_e_q2] = '1' or [sob_e_q2] = '1' or [fatigue_e_q2] = '1' or [bodyache_e_q2] = '1' or [headache_e_q2] = '1' or [diarrhea_e_q2] = '1' or [pharyngitis_e_q2] = '1' or [eye_e_q2] = '1' or [conges	When did the symptoms reported above first start?	text (date_mdy)

	t_e_q2] = '1' or [sensory_e_q2] = '1' or [rash_e_q2] = '1' or [chills_e_q2] = '1')		
1254	[covid_symp3_e_q2]<br><br>Show the field ONLY if:<br>[language_q2] = '1' and ([fever_e_q2] = '1' or [cough_e_q2] = '1' or [sob_e_q2] = '1' or [fatigue_e_q2] = '1' or [bodyache_e_q2] = '1' or [headache_e_q2] = '1' or [diarrhea_e_q2] = '1' or [pharyngitis_e_q2] = '1' or [eye_e_q2] = '1' or [congest_e_q2] = '1' or [sensory_e_q2] = '1' or [rash_e_q2] = '1' or [chills_e_q2] = '1')	Did you experience any bias or discrimination due to the symptoms you reported?	radio<br>1 | yes<br>0 | no<br>2 | don't know
1255	[prevent17_e_q2]<br><br>Show the field ONLY if:<br>[language_q2] = '1' and ([fever_e_q2] = '1' or [cough_e_q2] = '1' or [sob_e_q2] = '1' or [fatigue_e_q2] = '1' or [bodyache_e_q2] = '1' or [headache_e_q2] = '1' or [diarrhea_e_q2] = '1' or [pharyngitis_e_q2] = '1' or [eye_e_q2] = '1' or [congest_e_q2] = '1' or [sensory_e_q2] = '1' or [rash_e_q2] = '1' or [chills_e_q2] = '1')	Which of the following did you do to protect your friends and family after your symptoms began?	checkbox<br>1 | prevent17_e_q2___1 | wore a mask more frequently<br>2 | prevent17_e_q2___2 | washed your hands with soap and water more frequently<br>3 | prevent17_e_q2___3 | used hand sanitizer more frequently<br>4 | prevent17_e_q2___4 | isolated yourself in your home more frequently<br>5 | prevent17_e_q2___5 | stayed home more frequently<br>6 | prevent17_e_q2___6 | wore disposable gloves more frequently
1256	[covid_symp4_e_q2]<br><br>Show the field ONLY if:<br>[language_q2] = '1' and ([fever_e_q2] = '1' or [cough_e_q2] = '1' or [sob_e_q2] = '1' or [fatigue_e_q2] = '1' or [bodyache_e_q2] = '1' or [headache_e_q2] = '1' or [diarrh	What did you do in response to the symptoms reported above?<br>*Select all that apply.*	checkbox<br>0 | covid_symp4_e_q2___0 | nothing<br>1 | covid_symp4_e_q2___1 | took over the counter medication (ibuprofen, acetaminophen, etc.)

	ea_e_q2] = '1' or [pharyngitis_e_q2] = '1' or [eye_e_q2] = '1' or [congest_e_q2] = '1' or [sensory_e_q2] = '1' or [rash_e_q2] = '1' or [chills_e_q2] = '1')		2 covid_symp4_e_q2___2 communicated with a health care provider over the phone<br>3 covid_symp4_e_q2___3 visited a health care provider's office<br>4 covid_symp4_e_q2___4 visited a retail clinic or pharmacy<br>5 covid_symp4_e_q2___5 visited urgent care (FASTMed, etc.)<br>6 covid_symp4_e_q2___6 visited the emergency room<br>7 covid_symp4_e_q2___7 was admitted to the hospital<br>8 covid_symp4_e_q2___8 other
1257	[covid_symp5_e_q2]<br>Show the field ONLY if: [language_q2] = '1' and [covid_symp4_e_q2(8)] = '1'	Please specify what other action you took in response to your symptoms.	text
1258	[covid_symp6_e_q2]<br>Show the field ONLY if: [language_q2] = '1' and ([covid_symp4_e_q2(2)] = '1' or [covid_symp4_e_q2(3)] = '1' or [covid_symp4_e_q2(4)] = '1' or [covid_symp4_e_q2(5)] = '1' or [covid_symp4_e_q2(6)] = '1' or [covid_symp4_e_q2(7)] = '1' or [covid_symp4_e_q2(8)] = '1')	If you were able to talk with a health care provider, were you told that you may have COVID-19?	radio<br>1 yes<br>0 no<br>2 don't know
1259	[covid_test_e_q2]<br>Show the field ONLY if: [language_q2] = '1' and ([fever_e_q2] = '1' or [cough_e_q2] = '1' or [sob_e_q2] = '1' or [fatigue_e_q2] = '1' or [bodyache_e_q2] = '1' or [headache_e_q2] = '1' or [diarrhea_e_q2] = '1' or [pharyngitis_e_q2] = '1' or [eye_e_q2] = '1' or [congest_e_q2] = '1' or [sensory_e_q2] = '1' or [rash_e_	If you received a COVID-19 test due to the symptoms you reported, what was the result?	radio<br>1 pending<br>2 positive<br>3 negative<br>4 inconclusive<br>5 did not receive a test

	q2] = '1' or [chills_e_q2] = '1')		
1260	[covid_admit_e_q2]<br><br>Show the field ONLY if: [language_q2] = '1' and [covid_symp4_e_q2(7)] = '1'	How many days were you admitted to the hospital?	text (number, Min: 0)
1261	[covid_admit2_e_q2]<br><br>Show the field ONLY if: [language_q2] = '1' and [covid_symp4_e_q2(7)] = '1'	Did you receive the following interventions during your hospital admission?	checkbox<br>1 covid_admit2_e_q2___1 extra oxygen in your nose<br>2 covid_admit2_e_q2___2 treatment in the intensive care unit (ICU)<br>3 covid_admit2_e_q2___3 mechanical ventilation (intubation or a breathing tube)
1262	[covid_symp7_e_q2]<br><br>Show the field ONLY if: [language_q2] = '1' and ([fever_e_q2] = '1' or [cough_e_q2] = '1' or [sob_e_q2] = '1' or [fatigue_e_q2] = '1' or [bodyache_e_q2] = '1' or [headache_e_q2] = '1' or [diarrhea_e_q2] = '1' or [pharyngitis_e_q2] = '1' or [eye_e_q2] = '1' or [congest_e_q2] = '1' or [sensory_e_q2] = '1' or [rash_e_q2] = '1' or [chills_e_q2] = '1')	Have you returned to your normal health at this time?	radio<br>1 yes<br>0 no<br>2 don't know
1263	[vaccine_trial_e_q2]<br><br>Show the field ONLY if: [language_q2] = '1'	Have you ever been, or are currently, in a COVID vaccine trial?	yesno<br>1 Yes<br>0 No
1264	[vaccine_trial2_e_q2]<br><br>Show the field ONLY if: [language_q2] = '1' and [vaccine_trial_e_q2] = '1'	Where was/is the primary site for the COVID vaccine trial? (For example: UNC, Duke, a specific hospital?)	text
1265	[receivedcovidvaccine_e_q2]<br><br>Show the field ONLY if:	Have you received a COVID vaccine outside of a clinical trial?	yesno<br>1 Yes

	[language_q2] = '1'		0 No
1266	[vacclast2week_e]<br>Show the field ONLY if: [receivedcovidvaccine_e_q2] = '1'	Did you receive the COVID-19 vaccination since filling out your last biweekly survey?	yesno<br>1 Yes<br>0 No
1267	[receivedvaccine_where_e_q2]<br>Show the field ONLY if: [language_q2] = '1' and [vacclast2week_e] = '1'	Where did you receive the COVID vaccine?	radio<br>1 Doctors Office<br>2 Work/Employment<br>3 Retail (e.g. Walgreens, CVS)<br>5 Vaccine site<br>4 Other
1268	[receivedvaccine_where_oth_e_q2]<br>Show the field ONLY if: [language_q2] = '1' and [vacclast2week_e] = '1' and [receivedvaccine_where_e] = '4'	Please specify where you received the COVID vaccine.	text
1269	[receivedvaccine_site_e_q2]<br>Show the field ONLY if: [language_q2] = '1' and [vacclast2week_e] = '1' and [receivedvaccine_where_e_q2] = '5'	Please specify the city/town in NC of the vaccine site:	text
1270	[receivedvaccine_site2_e_q2]<br>Show the field ONLY if: [language_q2] = '1' and [vacclast2week_e] = '1' and [receivedvaccine_where_e_q2] = '5'	Please specify who was the hosting organization/institution of the vaccine site:	text
1271	[receivedvaccine_name_e_q2]<br>Show the field ONLY if: [language_q2] = '1' and [vacclast2week_e] = '1'	Which COVID vaccine did you receive?	radio<br>1 Pfizer<br>2 Moderna<br>3 AstraZeneca<br>4 Novavax<br>6 Johnson & Johnson<br>5 Other<br>9 Unsure/Unknown
1272	[receivedvaccine_name_oth_e_q2]<br>Show the field ONLY if: [language_q2] = '1' AND [receivedvaccine_na	Please specify which other COVID vaccine you received.	text

	me_e_q2] = '5'		
1273	[receivedvaccine_doses_e_q2]<br><br>Show the field ONLY if: [language_q2] = '1' and [vacclast2week_e] = '1'	How many doses of the COVID vaccine have you received?	radio<br>1 | 1<br>2 | 2
1274	[receivedvaccine_first_dt_e_q2]<br><br>Show the field ONLY if: [language_q2] = '1' and [vacclast2week_e] = '1' and [receivedvaccine_doses_e_q2] = '1' OR [receivedvaccine_doses_e_q2] = '2'	Which day did you receive the first dose of the vaccine?	text (date_mdy), Required
1275	[receivedvaccine_second_dt_e_q2]<br><br>Show the field ONLY if: [language_q2] = '1' and [vacclast2week_e] = '1' and [receivedvaccine_doses_e_q2] = '2'	Which day did you receive the second dose of the vaccine?	text (date_mdy), Required
1276	[vacc_pain_e_q2]<br><br>Show the field ONLY if: [language_q2] = '1' and [vacclast2week_e] = '1'	Section Header: *Did you experience any of the following side effects after vaccination?*<br><br>pain at or around the injection site	radio (Matrix)<br>0 | No<br>1 | Mild (you notice symptoms, but they aren't a problem)<br>2 | Moderate (symptoms limit your normal daily activities)<br>3 | Severe (symptoms make normal daily activities difficult or impossible)<br><br>Question number: 1
1277	[vacc_redness_e_q2]<br><br>Show the field ONLY if: [language_q2] = '1' and [vacclast2week_e] = '1'	redness at or around the injection site	radio (Matrix)<br>0 | No<br>1 | Mild (you notice symptoms, but they aren't a problem)<br>2 | Moderate (symptoms limit your normal daily activities)<br>3 | Severe (symptoms make normal daily activities difficult or impossible)<br><br>Question number: 2
1278	[vacc_swell_e_q2]<br><br>Show the field ONLY if: [language_q2] = '1' and [vacclast2week_e] = '1'	swelling at or around the injection site	radio (Matrix)<br>0 | No<br>1 | Mild (you notice symptoms, but they aren't a problem)<br>2 | Moderate (symptoms limit your normal daily activities)

			3 Severe (symptoms make normal daily activities difficult or impossible)<br><br>Question number: 3
1279	[vacc_rash_e_q2]<br><br>Show the field ONLY if: [language_q2] = '1' and [vacclast2week_e] = '1'	rash at or around the injection site	radio (Matrix)<br>0 No<br>1 Mild (you notice symptoms, but they aren't a problem)<br>2 Moderate (symptoms limit your normal daily activities)<br>3 Severe (symptoms make normal daily activities difficult or impossible)<br><br>Question number: 4
1280	[vacc_headache_e_q2]<br><br>Show the field ONLY if: [language_q2] = '1' and [vacclast2week_e] = '1'	headache	radio (Matrix)<br>0 No<br>1 Mild (you notice symptoms, but they aren't a problem)<br>2 Moderate (symptoms limit your normal daily activities)<br>3 Severe (symptoms make normal daily activities difficult or impossible)<br><br>Question number: 5
1281	[vacc_fatigue_e_q2]<br><br>Show the field ONLY if: [language_q2] = '1' and [vacclast2week_e] = '1'	fatigue	radio (Matrix)<br>0 No<br>1 Mild (you notice symptoms, but they aren't a problem)<br>2 Moderate (symptoms limit your normal daily activities)<br>3 Severe (symptoms make normal daily activities difficult or impossible)<br><br>Question number: 6
1282	[vacc_fever_e_q2]<br><br>Show the field ONLY if: [language_q2] = '1' and [vacclast2week_e] = '1'	fever (temperature >100.4°F or >38°C)	radio (Matrix)<br>0 No<br>1 Mild (you notice symptoms, but they aren't a problem)<br>2 Moderate (symptoms limit your normal daily activities)<br>3 Severe (symptoms make normal daily activities difficult or impossible)<br><br>Question number: 7
1283	[vacc_chills_e_q2]<br><br>Show the field ONLY if:	chills	radio (Matrix)<br>0 No

	[language_q2] = '1' and [vacclast2week_e] = '1'		1 Mild (you notice symptoms, but they aren't a problem)<br>2 Moderate (symptoms limit your normal daily activities)<br>3 Severe (symptoms make normal daily activities difficult or impossible)<br><br>Question number: 8
1284	[vacc_joint_e_q2]<br><br>Show the field ONLY if: [language_q2] = '1' and [vacclast2week_e] = '1'	joint pain	radio (Matrix)<br>0 No<br>1 Mild (you notice symptoms, but they aren't a problem)<br>2 Moderate (symptoms limit your normal daily activities)<br>3 Severe (symptoms make normal daily activities difficult or impossible)<br><br>Question number: 9
1285	[vacc_muscle_e_q2]<br><br>Show the field ONLY if: [language_q2] = '1' and [vacclast2week_e] = '1'	muscle pain	radio (Matrix)<br>0 No<br>1 Mild (you notice symptoms, but they aren't a problem)<br>2 Moderate (symptoms limit your normal daily activities)<br>3 Severe (symptoms make normal daily activities difficult or impossible)<br><br>Question number: 10
1286	[vacc_nausea_e_q2]<br><br>Show the field ONLY if: [language_q2] = '1' and [vacclast2week_e] = '1'	nausea	radio (Matrix)<br>0 No<br>1 Mild (you notice symptoms, but they aren't a problem)<br>2 Moderate (symptoms limit your normal daily activities)<br>3 Severe (symptoms make normal daily activities difficult or impossible)<br><br>Question number: 11
1287	[vacc_effects_e_q2]<br><br>Show the field ONLY if: [vacc_pain_e_q2]>0 OR [vacc_redness_e_q2]>0 OR [vacc_swell_e_q2]>0 OR [vacc_rash_e_q2]>0 OR [vacc_headache_e_q2]>0 OR [vacc_fatigue_e_q2]>0 OR [vacc_fever_e_q2]>0 OR [vacc_chil	How long did these side effects last?	radio<br>1 Less than 12 hours<br>2 12 to 24 hours<br>3 more than 24 hours

	ls_e_q2]>0 OR [vacc_joint_e_q2]>0 OR [vacc_muscle_e_q2]>0 OR [vacc_nausea_e_q2]>0		
1288	[vacc_med_e_q2]<br><br>Show the field ONLY if: [vacc_pain_e_q2]>0 OR [vacc_redness_e_q2]>0 OR [vacc_swell_e_q2]>0 OR [vacc_rash_e_q2]>0 OR [vacc_headache_e_q2]>0 OR [vacc_fatigue_e_q2]>0 OR [vacc_fever_e_q2]>0 OR [vacc_chills_e_q2]>0 OR [vacc_joint_e_q2]>0 OR [vacc_muscle_e_q2]>0 OR [vacc_nausea_e_q2]>0	Did you take any medication for these side effects?	radio<br>1 Yes<br>0 No
1289	[vacc_med_list_e_q2]<br><br>Show the field ONLY if: [vacc_med_e_q2] = '1'	What medication(s) did you take for the side effects?<br>*Please list all medications.*	text
1290	[vacc_effects_consult_e_q2]<br><br>Show the field ONLY if: [vacc_pain_e_q2]>0 OR [vacc_redness_e_q2]>0 OR [vacc_swell_e_q2]>0 OR [vacc_rash_e_q2]>0 OR [vacc_headache_e_q2]>0 OR [vacc_fatigue_e_q2]>0 OR [vacc_fever_e_q2]>0 OR [vacc_chills_e_q2]>0 OR [vacc_joint_e_q2]>0 OR [vacc_muscle_e_q2]>0 OR [vacc_nausea_e_q2]>0	Did you consult a physician or other health care provider for the side effects?	radio<br>1 Yes<br>0 No
1291	[vacc_effects_dose_e_q2]<br><br>Show the field ONLY if: [vacc_pain_e_q2]>0 OR [vacc_redness_e_q2]>0 OR [vacc_swell_e_q2]>0 OR [vacc_rash_e_q2]>0 OR [vacc_headache_e_q2]>0 OR [vacc_fatigue_e_q2]>0 OR [vacc_fever_e_q2]>0 OR [vacc_chills_e_q2]>0 OR [vacc_joint_e_q2]>0 OR [vacc_muscle_e_q2]>0 OR [vacc_nausea_e_q2]>0	How did you experience the side effects after the second dose of the vaccination as compared to those after the first dose of the vaccination?	radio<br>3 More severe<br>2 Less severe<br>1 Equally severe<br>0 Not applicable/Haven't received second dose yet

1292	[receive_booster_e_q2]<br>Show the field ONLY if:<br>[language_q2] = '1'	Have you received a booster dose of the COVID-19 vaccine?	yesno<br>1 Yes<br>0 No
1293	[boosterlast2week_e]<br>Show the field ONLY if:<br>[receive_booster_e_q2] = '1'	Did you receive a COVID-19 booster since filling out your last biweekly survey or that you have not documented?	yesno<br>1 Yes<br>0 No
1294	[receive_booster_num_e]<br>Show the field ONLY if:<br>[boosterlast2week_e] = '1'	Please indicate what number booster dose you received<br>*Example: select 2 if you received four mRNA doses or two doses of mRNA and 1 J&J*	dropdown<br>1 1<br>2 2<br>3 3<br>4 4<br>5 5<br>6 6<br>7 7<br>8 8<br>9 9<br>10 10
1295	[receive_booster2_e_q2]<br>Show the field ONLY if:<br>[receive_booster_e_q2] = '0'	Do you plan to get a booster dose for COVID-19?	radio<br>1 Yes<br>2 No<br>3 Don't know
1296	[receive_booster3_e_q2]<br>Show the field ONLY if:<br>[boosterlast2week_e] = '1'	What is the main factor influencing your decision to get the booster dose of the COVID-19 vaccine at this time?<br>*Select all that apply*	checkbox<br>1 receive_booster3_e_q2___1 Was required for my employment<br>2 receive_booster3_e_q2___2 Health condition<br>3 receive_booster3_e_q2___3 Age<br>4 receive_booster3_e_q2___4 Doctor suggestion<br>5 receive_booster3_e_q2___5 Worried that the vaccine I took, did not provide strong protection<br>6 receive_booster3_e_q2___6 Concerns about transmitting virus to others

			7 | receive_booster3_e_q2___7 | Other___
1297	[receive_boosterloc_e_q2]<br><br>Show the field ONLY if: [boosterlast2week_e] = '1'	Where did you receive the booster dose of the COVID-19 vaccine?	text
1298	[receive_booster3oth_e_q2]<br><br>Show the field ONLY if: [boosterlast2week_e] = '1' and [receive_booster3_e_q2(7)] = '1'	Please specify what the main factor influencing your decision to get the booster dose	text
1299	[receive_booster4_e_q2]<br><br>Show the field ONLY if: [receive_booster_e_q2] = '0'	What is the main factor influencing your decision NOT to get the booster dose of the COVID-19 vaccine at this time?<br>*Select all that apply*	checkbox<br>1 | receive_booster4_e_q2___1 | Not available to me<br>2 | receive_booster4_e_q2___2 | Did not think it was necessary for me<br>3 | receive_booster4_e_q2___3 | Did not think it was ethical when others have not received first dose<br>4 | receive_booster4_e_q2___4 | Concerns about safety<br>5 | receive_booster4_e_q2___5 | Just don't want to get it<br>6 | receive_booster4_e_q2___6 | Other reasons ____
1300	[receive_booster4oth_e_q2]<br><br>Show the field ONLY if: [receive_booster_e_q2] = '0' and [receive_booster4_e_q2(6)] = '1'	Please specify what the main factor influencing your decision NOT to get a booster dose	text
1301	[receive_booster5_e_q2]<br><br>Show the field ONLY if: [boosterlast2week_e] = '1'	Which COVID-19 booster dose did you receive?	radio<br>1 | Pfizer<br>2 | Moderna<br>3 | J&J

			4 Other
1302	[receive_booster5oth_e_q2]<br>Show the field ONLY if:<br>[boosterlast2week_e] = '1' and [receive_booster5_e_q2] = '4'	Please specify which other COVID-19 booster shot you received	text
1303	[receive_boosterdt_e_q2]<br>Show the field ONLY if:<br>[boosterlast2week_e] = '1'	What day did you receive your COVID-19 booster shot?	text (date_mdy)
1304	[booster_pain_e_q2]<br>Show the field ONLY if:<br>[boosterlast2week_e] = '1'	Section Header: *Please indicate any of the following symptoms after receiving the booster dose:*<br>Pain at or around the injection site	radio (Matrix)<br>0 No<br>1 Mild (you notice symptoms, but they aren't a problem)<br>2 Moderate (symptoms limit your normal daily activities)<br>3 Severe (symptoms make normal daily activities difficult or impossible)
1305	[booster_redness_e_q2]<br>Show the field ONLY if:<br>[boosterlast2week_e] = '1'	Redness at or around the injection site	radio (Matrix)<br>0 No<br>1 Mild (you notice symptoms, but they aren't a problem)<br>2 Moderate (symptoms limit your normal daily activities)<br>3 Severe (symptoms make normal daily activities difficult or impossible)
1306	[booster_swelling_e_q2]<br>Show the field ONLY if:<br>[boosterlast2week_e] = '1'	Swelling at or around the injection site	radio (Matrix)<br>0 No<br>1 Mild (you notice symptoms, but they aren't a problem)<br>2 Moderate (symptoms limit your normal daily activities)<br>3 Severe (symptoms make normal daily activities difficult or impossible)
1307	[booster_rash_e_q2]<br>Show the field ONLY if:<br>[boosterlast2week_e] = '1'	Rash at or around the injection site	radio (Matrix)<br>0 No<br>1 Mild (you notice symptoms, but they aren't a problem)<br>2 Moderate (symptoms limit your normal daily activities)<br>3 Severe (symptoms make normal daily activities difficult or impossible)

1308	[booster_headache_e_q2]<br><br>Show the field ONLY if: [boosterlast2week_e] = '1'	Headache	radio (Matrix)<br>0 No<br>1 Mild (you notice symptoms, but they aren't a problem)<br>2 Moderate (symptoms limit your normal daily activities)<br>3 Severe (symptoms make normal daily activities difficult or impossible)
1309	[booster_fatigue_e_q2]<br><br>Show the field ONLY if: [boosterlast2week_e] = '1'	Fatigue	radio (Matrix)<br>0 No<br>1 Mild (you notice symptoms, but they aren't a problem)<br>2 Moderate (symptoms limit your normal daily activities)<br>3 Severe (symptoms make normal daily activities difficult or impossible)
1310	[booster_fever_e_q2]<br><br>Show the field ONLY if: [boosterlast2week_e] = '1'	Fever (temperature >100.4°F or >38°C)	radio (Matrix)<br>0 No<br>1 Mild (you notice symptoms, but they aren't a problem)<br>2 Moderate (symptoms limit your normal daily activities)<br>3 Severe (symptoms make normal daily activities difficult or impossible)
1311	[booster_chills_e_q2]<br><br>Show the field ONLY if: [boosterlast2week_e] = '1'	Chills	radio (Matrix)<br>0 No<br>1 Mild (you notice symptoms, but they aren't a problem)<br>2 Moderate (symptoms limit your normal daily activities)<br>3 Severe (symptoms make normal daily activities difficult or impossible)
1312	[booster_joint_e_q2]<br><br>Show the field ONLY if: [boosterlast2week_e] = '1'	Joint pain	radio (Matrix)<br>0 No<br>1 Mild (you notice symptoms, but they aren't a problem)<br>2 Moderate (symptoms limit your normal daily activities)<br>3 Severe (symptoms make normal daily activities difficult or impossible)

1313	[booster_muscle_e_q2]<br><br>Show the field ONLY if: [boosterlast2week_e] = '1'	Muscle pain	radio (Matrix)<br>0 No<br>1 Mild (you notice symptoms, but they aren't a problem)<br>2 Moderate (symptoms limit your normal daily activities)<br>3 Severe (symptoms make normal daily activities difficult or impossible)
1314	[booster_nausea_e_q2]<br><br>Show the field ONLY if: [boosterlast2week_e] = '1'	Nausea	radio (Matrix)<br>0 No<br>1 Mild (you notice symptoms, but they aren't a problem)<br>2 Moderate (symptoms limit your normal daily activities)<br>3 Severe (symptoms make normal daily activities difficult or impossible)
1315	[receive_booster6_e_q2]<br><br>Show the field ONLY if: [booster_pain_e_q2] = '1' or [booster_pain_e_q2] = '2' or [booster_pain_e_q2] = '3' or [booster_redness_e_q2] = '1' or [booster_redness_e_q2] = '2' or [booster_redness_e_q2] = '3' or [booster_swelling_e_q2] = '1' or [booster_swelling_e_q2] = '2' or [booster_swelling_e_q2] = '3' or [booster_rash_e_q2] = '1' or [booster_rash_e_q2] = '2' or [booster_rash_e_q2] = '3' or [booster_headache_e_q2] = '1' or [booster_headache_e_q2] = '2' or [booster_headache_e_q2] = '3' or [booster_fatigue_e_q2] = '1' or [booster_fatigue_e_q2] = '2' or [booster_fatigue_e_q2] = '3' or [booster_fever_e_q2] = '1' or [booster_fever_e_q2] = '2' or [booster_fever_e_q2] = '3' or [booster_chills_e_q2] = '1' or [booster_chills_e_q2] = '2' or [booster_chills_e_q2] = '3' or [booster_joint_e_q2] = '1' or [boost	How long did these side effects last?	radio<br>1 Less than 12 hours<br>2 12 to 24 hours<br>3 More than 24 hours

	er_joint_e_q2] = '2' or [booster_joint_e_q2] = '3' or [booster_muscle_e_q2] = '1' or [booster_muscle_e_q2] = '2' or [booster_muscle_e_q2] = '3' or [booster_nausea_e_q2] = '1' or [booster_nausea_e_q2] = '2' or [booster_nausea_e_q2] = '3'		
1316	[receive_booster7_e_q2]<br><br>Show the field ONLY if: [booster_pain_e_q2] = '1' or [booster_pain_e_q2] = '2' or [booster_pain_e_q2] = '3' or [booster_redness_e_q2] = '1' or [booster_redness_e_q2] = '2' or [booster_redness_e_q2] = '3' or [booster_swelling_e_q2] = '1' or [booster_swelling_e_q2] = '2' or [booster_swelling_e_q2] = '3' or [booster_rash_e_q2] = '1' or [booster_rash_e_q2] = '2' or [booster_rash_e_q2] = '3' or [booster_headache_e_q2] = '1' or [booster_headache_e_q2] = '2' or [booster_headache_e_q2] = '3' or [booster_fatigue_e_q2] = '1' or [booster_fatigue_e_q2] = '2' or [booster_fatigue_e_q2] = '3' or [booster_fever_e_q2] = '1' or [booster_fever_e_q2] = '2' or [booster_fever_e_q2] = '3' or [booster_chills_e_q2] = '1' or [booster_chills_e_q2] = '2' or [booster_chills_e_q2] = '3' or [booster_joint_e_q2] = '1' or [booster_joint_e_q2] = '2' or [booster_joint_e_q2] = '3' or [booster_muscle_e_q2] = '1' or [booster_muscle_e_q2] = '2' or [booster_muscle_e_q2] = '3' or [booster_nausea_e_q2] = '1' or [booster_nausea_e_q2] = '2' or [b	Did you take any medication for these side effects?	yesno<br>1 Yes<br>0 No

	ooster_nausea_e_q2] = '3'		
1317	[receive_booster8_e_q2]<br>Show the field ONLY if: [receive_booster7_e_q2] = '1'	What medication(s) did you take for these side effects?	text
1318	[receive_booster9_e_q2]<br>Show the field ONLY if: [booster_pain_e_q2] = '1' or [booster_pain_e_q2] = '2' or [booster_pain_e_q2] = '3' or [booster_redness_e_q2] = '1' or [booster_redness_e_q2] = '2' or [booster_redness_e_q2] = '3' or [booster_swelling_e_q2] = '1' or [booster_swelling_e_q2] = '2' or [booster_swelling_e_q2] = '3' or [booster_rash_e_q2] = '1' or [booster_rash_e_q2] = '2' or [booster_rash_e_q2] = '3' or [booster_headache_e_q2] = '1' or [booster_headache_e_q2] = '2' or [booster_headache_e_q2] = '3' or [booster_fatigue_e_q2] = '1' or [booster_fatigue_e_q2] = '2' or [booster_fatigue_e_q2] = '3' or [booster_fever_e_q2] = '1' or [booster_fever_e_q2] = '2' or [booster_fever_e_q2] = '3' or [booster_chills_e_q2] = '1' or [booster_chills_e_q2] = '2' or [booster_chills_e_q2] = '3' or [booster_joint_e_q2] = '1' or [booster_joint_e_q2] = '2' or [booster_joint_e_q2] = '3' or [booster_muscle_e_q2] = '1' or [booster_muscle_e_q2] = '2' or [booster_muscle_e_q2] = '3' or [booster_nausea_e_q2] = '1' or [booster_nausea_e_q2] = '2' or [booster_nausea_e_q2] = '3'	Did you consult a physician or other health care provider for these side effects?	yesno<br>1 Yes<br>0 No
1319	[receive_booster10_e_q2]	Were the side effects you experienced after the booster dose of the vaccination more or less	radio

	Show the field ONLY if: [booster_pain_e_q2] = '1' or [booster_pain_e_q2] = '2' or [booster_pain_e_q2] = '3' or [booster_redness_e_q2] = '1' or [booster_redness_e_q2] = '2' or [booster_redness_e_q2] = '3' or [booster_swelling_e_q2] = '1' or [booster_swelling_e_q2] = '2' or [booster_swelling_e_q2] = '3' or [booster_rash_e_q2] = '1' or [booster_rash_e_q2] = '2' or [booster_rash_e_q2] = '3' or [booster_headache_e_q2] = '1' or [booster_headache_e_q2] = '2' or [booster_headache_e_q2] = '3' or [booster_fatigue_e_q2] = '1' or [booster_fatigue_e_q2] = '2' or [booster_fatigue_e_q2] = '3' or [booster_fever_e_q2] = '1' or [booster_fever_e_q2] = '2' or [booster_fever_e_q2] = '3' or [booster_chills_e_q2] = '1' or [booster_chills_e_q2] = '2' or [booster_chills_e_q2] = '3' or [booster_joint_e_q2] = '1' or [booster_joint_e_q2] = '2' or [booster_joint_e_q2] = '3' or [booster_muscle_e_q2] = '1' or [booster_muscle_e_q2] = '2' or [booster_muscle_e_q2] = '3' or [booster_nausea_e_q2] = '1' or [booster_nausea_e_q2] = '2' or [booster_nausea_e_q2] = '3'	severe as compared to the side effects you experienced after the first dose of the vaccination?	1 More severe<br>2 Equally severe<br>3 Less severe<br>4 I did not experience any side effects with the first dose
1320	[receive_booster11_e_q2]<br><br>Show the field ONLY if: [booster_pain_e_q2] = '1' or [booster_pain_e_q2] = '2' or [booster_pain_e_q2] = '3' or [booster_redness_e_q2] = '1' or [booster_redness_e_q2] = '2' or [booster_redness_e_q2] = '3' or [booster_swelling_e_q2] =	Were the side effects you experienced after the booster dose of the vaccination more or less severe as compared to the side effects you experienced after the second dose of the vaccination?	radio<br>1 More severe<br>2 Equally severe<br>3 Less severe<br>4 Not applicable/only received one dose (J&J)<br>5 I did not experience any side effects with the second dose

	'1' or [booster_swelling_e_q2] = '2' or [booster_swelling_e_q2] = '3' or [booster_rash_e_q2] = '1' or [booster_rash_e_q2] = '2' or [booster_rash_e_q2] = '3' or [booster_headache_e_q2] = '1' or [booster_headache_e_q2] = '2' or [booster_headache_e_q2] = '3' or [booster_fatigue_e_q2] = '1' or [booster_fatigue_e_q2] = '2' or [booster_fatigue_e_q2] = '3' or [booster_fever_e_q2] = '1' or [booster_fever_e_q2] = '2' or [booster_fever_e_q2] = '3' or [booster_chills_e_q2] = '1' or [booster_chills_e_q2] = '2' or [booster_chills_e_q2] = '3' or [booster_joint_e_q2] = '1' or [booster_joint_e_q2] = '2' or [booster_joint_e_q2] = '3' or [booster_muscle_e_q2] = '1' or [booster_muscle_e_q2] = '2' or [booster_muscle_e_q2] = '3' or [booster_nausea_e_q2] = '1' or [booster_nausea_e_q2] = '2' or [booster_nausea_e_q2] = '3'		
1321	[address_e_q2]<br><br>Show the field ONLY if: [language_q2] = '1'	Section Header: *Please provide the following information about your household.*<br><br>What is your permanent address?	text<br>Field Annotation: @DEFAULT="[address_e_q2]"
1322	[address2_e_q2]<br><br>Show the field ONLY if: [language_q2] = '1'	How long have you lived at this address?	radio<br>1 | 0-3 years<br>2 | 4-6 years<br>3 | 7-10 years<br>4 | more than 10 years<br><br>Field Annotation: @DEFAULT="[address2_e_q2]"
1323	[hhcount_e_q2]<br><br>Show the field ONLY if: [language_q2] = '1'	How many additional people (not including yourself) live or spend a significant amount of time (greater than 40 hours a week) in this household?	radio<br>0 | 0<br>1 | 1<br>2 | 2<br>3 | 3

			4 4<br>5 5<br>6 6<br>7 7<br>8 8<br>9 9<br>10 10<br>11 11<br>12 12<br><br>Field Annotation: @DEFAULT="[hhcount_e_q2]"
1324	[hhcount2_e_q2]<br><br>Show the field ONLY if: [language_q2] = '1' and [hhcount_e_q2] > 0	How many of the people in your household are below the age of 18?	radio<br>0 0<br>1 1<br>2 2<br>3 3<br>4 4<br>5 5<br>6 6<br>7 7<br>8 8<br>9 9<br>10 10<br>11 11<br>12 12<br><br>Field Annotation: @DEFAULT="[hhcount2_e_q2]"
1325	[hh1_relationship_e_q2]<br><br>Show the field ONLY if: [language_q2] = '1' and [hhcount_e_q2] > 0 and [hhcount_e_q2] < 13	Section Header: *For each additional person in the your household, please provide the following information.*<br><br>Person 1: What is your relationship to this person?	radio<br>1 partner or spouse<br>2 child<br>3 parent<br>4 sibling<br>5 other family member<br>6 in-home childcare provider or other caregiver<br>7 other<br><br>Field Annotation: @DEFAULT="[hh1_relationship_e_q2]"

1326	[hh1_relationship2_e_q2]<br><br>Show the field ONLY if: [language_q2] = '1' and [hh1_relationship_e_q2] = '7'	Person 1: Please specify your relationship with this person.	text<br>Field Annotation: @DEFAULT="[hh1_relationship2_e_q2]"
1327	[hh1_age_e_q2]<br><br>Show the field ONLY if: [language_q2] = '1' and [hhcount_e_q2] > 0 and [hhcount_e_q2] < 13	Person 1: What is this person's age?<br>*Please specify their age in years*	text (number, Min: 0, Max: 110)<br>Field Annotation: @DEFAULT="[hh1_age_e_q2]"
1328	[hh1_sex_e_q2]<br><br>Show the field ONLY if: [language_q2] = '1' and [hhcount_e_q2] > 0 and [hhcount_e_q2] < 13	Person 1: What is this person's sex?	radio<br>1 Female<br>2 Male<br>3 Other<br><br>Field Annotation: @DEFAULT="[hh1_sex_e_q2]"
1329	[hh1_race_e_q2]<br><br>Show the field ONLY if: [language_q2] = '1' and [hhcount_e_q2] > 0 and [hhcount_e_q2] < 13	Person 1: What is this person's race?<br>*Select all that apply.*	checkbox<br>1 hh1_race_e_q2___1 American Indian or Alaska Native<br>2 hh1_race_e_q2___2 Asian<br>3 hh1_race_e_q2___3 Black or African American<br>4 hh1_race_e_q2___4 Native Hawaiian or Pacific Islander<br>5 hh1_race_e_q2___5 White<br>6 hh1_race_e_q2___6 Other<br>7 hh1_race_e_q2___7 don't know<br><br>Field Annotation: @DEFAULT="[hh1_race_e_q2]"
1330	[hh1_e_q2thn_e_q2]<br><br>Show the field ONLY if: [language_q2] = '1' and [hhcount_e_q2] > 0 and [hhcount_e_q2] < 13	Person 1: What is this person's ethnicity?	radio<br>1 Hispanic or Latino<br>2 Not Hispanic or Latino<br>3 Other<br>4 don't know<br><br>Field Annotation: @DEFAULT="[hh1_e_q2thn_e_q2]"
1331	[hh1_e_q2du_e_q2]<br><br>Show the field ONLY if: [language_q2] = '1' and [hhcount_e_q2] > 0 and [hhcount_e_q2] < 13	Person 1: What is the highest level of education or schooling this person has completed?	radio<br>1 never attended school<br>2 kindergarten - 8th grade<br>3 some high school

			4 high school equivalency (GED)<br>5 high school graduate<br>6 some college<br>7 college graduate<br>8 graduate school or more<br>9 don't know<br><br>Field Annotation: @DEFAULT="[hh1_e_q2du_e_q2]"
1332	[hh1_work_e_q2]<br><br>Show the field ONLY if: [language_q2] = '1' and [hhcount_e_q2] > 0 and [hhcount_e_q2] < 13	Person 1: Which of the following best fit this person's current work situation?	radio<br>1 works full time<br>2 works part time<br>3 is looking for work/employment<br>4 retired<br>5 homemaker<br>6 student<br>7 on maternity/paternity leave<br>8 on illness/sick leave<br>9 on disability<br>10 other<br>11 don't know<br><br>Field Annotation: @DEFAULT="[hh1_work_e_q2]"
1333	[hh1_work2_e_q2]<br><br>Show the field ONLY if: [language_q2] = '1' and [hhcount_e_q2] > 0 and [hhcount_e_q2] < 13	Person 1: Does this person currently consider themselves self-employed (including as an independent contractor or gig-economy worker)?	radio<br>1 yes<br>0 no<br>2 don't know<br><br>Field Annotation: @DEFAULT="[hh1_work2_e_q2]"
1334	[hh1_work3_e_q2]<br><br>Show the field ONLY if: [language_q2] = '1' and [hhcount_e_q2] > 0 and [hhcount_e_q2] < 13 and ([hh1_work_e_q2] = '1' or [hh1_work_e_q2] = '2' or [hh1_work2_e_q2] = '1')	Person 1: Does this person currently work in any of the following high-risk settings for COVID-19 transmission?	checkbox<br>1 hh1_work3_e_q2___1 healthcare setting (hospital, clinic, urgent care, etc.)<br>2 hh1_work3_e_q2___2 dense residential setting (nursing home, other long-term care facility)<br>3 hh1_work3_e_q2___3 prison or jail

			4 hh1_work3_e_q2___4 meatpacking facility<br>5 hh1_work3_e_q2___5 shipping or distribution facility<br>6 hh1_work3_e_q2___6 high-volume retail facility (grocery store, etc.)<br>7 hh1_work3_e_q2___7 don't know<br><br>Field Annotation: @DEFAULT="[hh1_work3_e_q2]"
1335	[hh1_work4_e_q2]<br><br>Show the field ONLY if: [language_q2] = '1' and [hhcount_e_q2] > 0 and [hhcount_e_q2] < 13 and ([hh1_work_e_q2] = '1' or [hh1_work_e_q2] = '2' or [hh1_work2_e_q2] = '1')	Person 1: Does this person's employer offer them any of the following benefits at their current main job?<br>*Select all that apply.*	checkbox<br>1 hh1_work4_e_q2___1 paid sick leave<br>2 hh1_work4_e_q2___2 paid vacation/personal leave<br>3 hh1_work4_e_q2___3 health insurance<br>4 hh1_work4_e_q2___4 disability insurance<br>5 hh1_work4_e_q2___5 retirement plan<br>6 hh1_work4_e_q2___6 other<br>7 hh1_work4_e_q2___7 don't know<br><br>Field Annotation: @DEFAULT="[hh1_work4_e_q2]"
1336	[hh1_work5_e_q2]<br><br>Show the field ONLY if: [language_q2] = '1' and [hhcount_e_q2] > 0 and [hhcount_e_q2] < 13 and ([hh1_work_e_q2] = '1' or [hh1_work_e_q2] = '2' or [hh1_work2_e_q2] = '1')	Person 1: On a scale of 0 (definitely not going to happen) to 10 (definitely going to happen), how likely is it that this person will lose their job because of the COVID-19 pandemic?	text (number, Min: 0, Max: 10)<br>Field Annotation: @DEFAULT="[hh1_work5_e_q2]"
1337	[hh1_work6_e_q2]<br><br>Show the field ONLY if: [language_q2] = '1' and [hhcount_e_q2] > 0 and [hhcount_e_q2] < 13 and ([hh1_work_e_q2] = '1' or [hh1_work_e_q2] = '2' or [hh1_work2_e_q2] = '1')	Person 1: On a scale of 0 (definitely not going to happen) to 10 (definitely going to happen), how likely is it that this person will receive fewer work hours at their job because of the COVID-19 pandemic?	text (number, Min: 0, Max: 10)<br>Field Annotation: @DEFAULT="[hh1_work6_e_q2]"
1338	[hh1_work7_e_q2]<br><br>Show the field ONLY if: [language_q2] = '1' and [hhcount_e_q2] > 0 and	Person 1: How often is this person required to work from outside of the home currently?	radio (Matrix)<br>1 always (100%)<br>2 most of the time (75%)

	[hhcount_e_q2] < 13 and ([hh1_work_e_q2] = '1' or [hh1_work_e_q2] = '2' or [hh1_work2_e_q2] = '1')		3 half of the time (50%)<br>4 less than half of the time (25%)<br>5 never (0%)<br>6 don't know
1339	[hh1_work8_e_q2]<br><br>Show the field ONLY if: [language_q2] = '1' and ([hh1_work7_e_q2] = '1' or [hh1_work7_e_q2] = '2' or [hh1_work7_e_q2] = '3' or [hh1_work7_e_q2] = '4')	Person 1: How regularly is this person in close physical contact with co-workers during their work outside of the home currently?	radio (Matrix)<br>1 always (100%)<br>2 most of the time (75%)<br>3 half of the time (50%)<br>4 less than half of the time (25%)<br>5 never (0%)<br>6 don't know
1340	[hh1_work9_e_q2]<br><br>Show the field ONLY if: [language_q2] = '1' and ([hh1_work7_e_q2] = '1' or [hh1_work7_e_q2] = '2' or [hh1_work7_e_q2] = '3' or [hh1_work7_e_q2] = '4')	Person 1: How regularly is this person in close physical contact with clients during their work outside of the home currently?	radio (Matrix)<br>1 always (100%)<br>2 most of the time (75%)<br>3 half of the time (50%)<br>4 less than half of the time (25%)<br>5 never (0%)<br>6 don't know
1341	[hh1_covidvaccine_e_q2]<br><br>Show the field ONLY if: [language_q2] = '1' and [hhcount_e_q2] > 0 and [hhcount_e_q2] < 13	Person 1: Does this person plan to get a vaccine for COVID-19?	radio<br>1 Yes<br>0 No<br>2 Don't know<br>3 This individual has already been vaccinated<br><br>Field Annotation: @DEFAULT=" [hh1_covidvaccine_e_q2]"
1342	[hh1_covidsymp_e_q2]<br><br>Show the field ONLY if: [language_q2] = '1' and [hhcount_e_q2] > 0 and [hhcount_e_q2] < 13	Person 1: Has this person had any symptoms (cough, fever, difficulty breathing, fatigue, body aches, diarrhea, runny nose, loss of smell or taste) consistent with COVID-19 in the last two weeks?	radio<br>1 yes<br>0 no<br>2 don't know
1343	[hh1_covidsymp2_e_q2]<br><br>Show the field ONLY if: [language_q2] = '1' and [hh1_covidsymp_e_q2] = '1'	Person 1: When did this person's symptoms begin?	text (date_mdy)
1344	[hh1_covidsymp3_e_q2]<br><br>Show the field ONLY if:	Person 1: Is this person worried that they may have had COVID-19 because of their symptoms?	radio<br>1 yes<br>0 no

	[language_q2] = '1' and [hh1_covidsymp_e_q2] = '1'		2 don't know
1345	[hh1_covidsymp4_e_q2]<br>Show the field ONLY if: [language_q2] = '1' and [hh1_covidsymp_e_q2] = '1'	Person 1: Did this person experience any bias or discrimination because of their symptoms?	radio<br>1 yes<br>0 no<br>2 don't know
1346	[hh1_covidsymp5_e_q2]<br>Show the field ONLY if: [language_q2] = '1' and [hh1_covidsymp_e_q2] = '1'	Person 1: What did this person do in response to their symptoms?<br>*Select all that apply.*	checkbox<br>0 hh1_covidsymp5_e_q2___0 nothing<br>1 hh1_covidsymp5_e_q2___1 took over th[illegible] counter medication (ibuprofen, acetaminop[illegible] etc.)<br>2 hh1_covidsymp5_e_q2___2 communica[illegible] with a healt[illegible] care provid[illegible] over the ph[illegible]<br>3 hh1_covidsymp5_e_q2___3 visited a he[illegible] care provid[illegible] office<br>4 hh1_covidsymp5_e_q2___4 visited a ret[illegible] clinic or pharmacy<br>5 hh1_covidsymp5_e_q2___5 visited urge[illegible] care (FASTM[illegible] etc.)<br>6 hh1_covidsymp5_e_q2___6 visited the emergency room<br>7 hh1_covidsymp5_e_q2___7 was admitte[illegible] the hospital<br>8 hh1_covidsymp5_e_q2___8 other<br>9 hh1_covidsymp5_e_q2___9 don't know
1347	[hh1_covidsymp6_e_q2]<br>Show the field ONLY if: [language_q2] = '1' and [hh1_covidsymp5_e_q2(8)] = '1'	Person 1: Please specify what other action this person took in response to their symptoms.	text
1348	[hh1_covidsymp7_e_q2]<br>Show the field ONLY if: [language_q2] = '1' and ([hh1_covidsymp5_e_q2(2)] = '1' or [hh1_covid	Person 1: Did a health care provider tell this person that they may have COVID-19?	radio<br>1 yes<br>0 no<br>2 don't know

	symp5_e_q2(3)] = '1' or [hh1_covidsymp5_e_q2(4)] = '1' or [hh1_covidsymp5_e_q2(5)] = '1' or [hh1_covidsymp5_e_q2(6)] = '1' or [hh1_covidsymp5_e_q2(7)] = '1' or [hh1_covidsymp5_e_q2(8)] = '1')		
1349	[hh1_covid_test_e_q2]<br>Show the field ONLY if:<br>[language_q2] = '1' and [hh1_covidsymp_e_q2] = '1'	Person 1: If this person received a COVID-19 test due to their symptoms, what was the result?	radio<br>1 pending<br>2 positive<br>3 negative<br>4 inconclusive<br>5 did not receive a test<br>6 don't know
1350	[hh1_covid_admit_e_q2]<br>Show the field ONLY if:<br>[language_q2] = '1' and [hh1_covidsymp5_e_q2(7)] = '1'	Person 1: How many days was this person admitted to the hospital?	text (number, Min: 0)
1351	[hh1_covid_admit2_e_q2]<br>Show the field ONLY if:<br>[language_q2] = '1' and [hh1_covidsymp5_e_q2(7)] = '1'	Person 1: Did this person receive any of the following interventions during their hospital admission?	checkbox<br>1 hh1_covid_admit2_e_q2___1 extra oxygen in your nose<br>2 hh1_covid_admit2_e_q2___2 treatment in the intensive care unit (ICU)<br>3 hh1_covid_admit2_e_q2___3 mechanical ventilation (intubation or a breathing tube)<br>4 hh1_covid_admit2_e_q2___4 don't know
1352	[hh1_covidsymp8_e_q2]<br>Show the field ONLY if:<br>[language_q2] = '1' and [hh1_covidsymp_e_q2] = '1'	Person 1: Has this person returned to their normal health at this time?	radio<br>1 yes<br>0 no<br>2 don't know
1353	[hh1_prevent_e_q2]<br>Show the field ONLY if:<br>[language_q2] = '1' and [hh1_covidsymp_e_q2] = '1'	Person 1: Which of the following did this person do to protect their friends and family after their symptoms began?	checkbox<br>1 hh1_prevent_e_q2___1 wore a mask more frequently

			2 hh1_prevent_e_q2___2 washed your hands with soap and water more frequently<br>3 hh1_prevent_e_q2___3 used hand sanitizer more frequently<br>4 hh1_prevent_e_q2___4 isolated yourself in your home more frequently<br>5 hh1_prevent_e_q2___5 stayed home more frequently<br>6 hh1_prevent_e_q2___6 wore disposable gloves more frequently<br>7 hh1_prevent_e_q2___7 don't know
1354	[hh2_relationship_e_q2]<br><br>Show the field ONLY if: [language_q2] = '1' and [hhcount_e_q2] > 1 and [hhcount_e_q2] < 13	Section Header: *For each additional person in the your household, please provide the following information.*<br><br>Person 2: What is your relationship to this person?	radio<br>1 partner or spouse<br>2 child<br>3 parent<br>4 sibling<br>5 other family member<br>6 in-home childcare provider or other caregiver<br>7 other<br><br>Field Annotation: @DEFAULT="[hh2_relationship_e_q2]"
1355	[hh2_relationship2_e_q2]<br><br>Show the field ONLY if: [language_q2] = '1' and [hh2_relationship_e_q2] = '7'	Person 2: Please specify your relationship with this person.	text<br>Field Annotation: @DEFAULT="[hh2_relationship2_e_q2]"
1356	[hh2_age_e_q2]<br><br>Show the field ONLY if: [language_q2] = '1' and [hhcount_e_q2] > 1 and [hhcount_e_q2] < 13	Person 2: What is this person's age?<br>*Please specify their age in years*	text (number, Min: 0, Max: 110)<br>Field Annotation: @DEFAULT="[hh2_age_e_q2]"
1357	[hh2_sex_e_q2]<br><br>Show the field ONLY if:	Person 2: What is this person's sex?	radio<br>1 Female

	[language_q2] = '1' and [hhcount_e_q2] > 1 and [hhcount_e_q2] < 13		2 Male<br>3 Other<br><br>Field Annotation: @DEFAULT=" [hh2_sex_e_q2]"
1358	[hh2_race_e_q2]<br><br>Show the field ONLY if: [language_q2] = '1' and [hhcount_e_q2] > 1 and [hhcount_e_q2] < 13	Person 2: What is this person's race?<br>*Select all that apply.*	checkbox<br>1 hh2_race_e_q2___1 American Indian or Alaska Native<br>2 hh2_race_e_q2___2 Asian<br>3 hh2_race_e_q2___3 Black or African American<br>4 hh2_race_e_q2___4 Native Hawaiian or Pacific Islander<br>5 hh2_race_e_q2___5 White<br>6 hh2_race_e_q2___6 Other<br>7 hh2_race_e_q2___7 don't know<br><br>Field Annotation: @DEFAULT=" [hh2_race_e_q2]"
1359	[hh2_e_q2thn_e_q2]<br><br>Show the field ONLY if: [language_q2] = '1' and [hhcount_e_q2] > 1 and [hhcount_e_q2] < 13	Person 2: What is this person's ethnicity?	radio<br>1 Hispanic or Latino<br>2 Not Hispanic or Latino<br>3 Other<br>4 don't know<br><br>Field Annotation: @DEFAULT=" [hh2_e_q2thn_e_q2]"
1360	[hh2_e_q2du_e_q2]<br><br>Show the field ONLY if: [language_q2] = '1' and [hhcount_e_q2] > 1 and [hhcount_e_q2] < 13	Person 2: What is the highest level of education or schooling this person has completed?	radio<br>1 never attended school<br>2 kindergarten - 8th grade<br>3 some high school<br>4 high school equivalency (GED)<br>5 high school graduate<br>6 some college<br>7 college graduate<br>8 graduate school or more<br>9 don't know<br><br>Field Annotation: @DEFAULT=" [hh2_e_q2du_e_q2]"
1361	[hh2_work_e_q2]<br><br>Show the field ONLY if:	Person 2: Which of the following best fit this person's current work situation?	radio<br>1 works full time<br>2 works part time

	[language_q2] = '1' and [hhcount_e_q2] > 1 and [hhcount_e_q2] < 13		3 is looking for work/employment<br>4 retired<br>5 homemaker<br>6 student<br>7 on maternity/paternity leave<br>8 on illness/sick leave<br>9 on disability<br>10 other<br>11 don't know<br><br>Field Annotation: @DEFAULT=" [hh2_work_e_q2]"
1362	[hh2_work2_e_q2]<br><br>Show the field ONLY if: [language_q2] = '1' and [hhcount_e_q2] > 1 and [hhcount_e_q2] < 13	Person 2: Does this person currently consider themselves self-employed (including as an independent contractor or gig-economy worker)?	radio<br>1 yes<br>0 no<br>2 don't know<br><br>Field Annotation: @DEFAULT=" [hh2_work2_e_q2]"
1363	[hh2_work3_e_q2]<br><br>Show the field ONLY if: [language_q2] = '1' and [hhcount_e_q2] > 1 and [hhcount_e_q2] < 13 and ([hh2_work_e_q2] = '1' or [hh2_work_e_q2] = '2' or [hh2_work2_e_q2] = '1')	Person 2: Does this person currently work in any of the following high-risk settings for COVID-19 transmission?	checkbox<br>1 hh2_work3_e_q2___1 healthcare setting (hospital, clinic, urgent care, etc.)<br>2 hh2_work3_e_q2___2 dense residential setting (nursing home, other long-term care facility)<br>3 hh2_work3_e_q2___3 prison or jail<br>4 hh2_work3_e_q2___4 meatpacking facility<br>5 hh2_work3_e_q2___5 shipping or distribution facility<br>6 hh2_work3_e_q2___6 high-volume retail facility (grocery store, etc.)<br>7 hh2_work3_e_q2___7 don't know<br><br>Field Annotation: @DEFAULT=" [hh2_work3_e_q2]"

1364	[hh2_work4_e_q2]<br><br>Show the field ONLY if:<br>[language_q2] = '1' and [hhcount_e_q2] > 1 and [hhcount_e_q2] < 13 and ([hh2_work_e_q2] = '1' or [hh2_work_e_q2] = '2' or [hh2_work2_e_q2] = '1')	Person 2: Does this person's employer offer them any of the following benefits at their current main job?<br>*Select all that apply.*	checkbox<br>1 hh2_work4_e_q2___1 paid sick leave<br>2 hh2_work4_e_q2___2 paid vacation/personal leave<br>3 hh2_work4_e_q2___3 health insurance<br>4 hh2_work4_e_q2___4 disability insurance<br>5 hh2_work4_e_q2___5 retirement plan<br>6 hh2_work4_e_q2___6 other<br>7 hh2_work4_e_q2___7 don't know<br><br>Field Annotation: @DEFAULT=" [hh2_work4_e_q2]"
1365	[hh2_work5_e_q2]<br><br>Show the field ONLY if:<br>[language_q2] = '1' and [hhcount_e_q2] > 1 and [hhcount_e_q2] < 13 and ([hh2_work_e_q2] = '1' or [hh2_work_e_q2] = '2' or [hh2_work2_e_q2] = '1')	Person 2: On a scale of 0 (definitely not going to happen) to 10 (definitely going to happen), how likely is it that this person will lose their job because of the COVID-19 pandemic?	text (number, Min: 0, Max: 10)<br>Field Annotation: @DEFAULT=" [hh2_work5_e_q2]"
1366	[hh2_work6_e_q2]<br><br>Show the field ONLY if:<br>[language_q2] = '1' and [hhcount_e_q2] > 1 and [hhcount_e_q2] < 13 and ([hh2_work_e_q2] = '1' or [hh2_work_e_q2] = '2' or [hh2_work2_e_q2] = '1')	Person 2: On a scale of 0 (definitely not going to happen) to 10 (definitely going to happen), how likely is it that this person will receive fewer work hours at their job because of the COVID-19 pandemic?	text (number, Min: 0, Max: 10)<br>Field Annotation: @DEFAULT=" [hh2_work6_e_q2]"
1367	[hh2_work7_e_q2]<br><br>Show the field ONLY if:<br>[language_q2] = '1' and [hhcount_e_q2] > 1 and [hhcount_e_q2] < 13 and ([hh2_work_e_q2] = '1' or [hh2_work_e_q2] = '2' or [hh2_work2_e_q2] = '1')	Person 2: How often is this person required to work from outside of the home currently?	radio (Matrix)<br>1 always (100%)<br>2 most of the time (75%)<br>3 half of the time (50%)<br>4 less than half of the time (25%)<br>5 never (0%)<br>6 don't know
1368	[hh2_work8_e_q2]<br><br>Show the field ONLY if:<br>[language_q2] = '1' and ([hh2_work7_e_q2] = '1' or [hh2_work7_e_q2] = '2' or [hh2_work7_e_q2] = '3' or [hh2_work7_e_q2] = '4')	Person 2: How regularly is this person in close physical contact with co-workers during their work outside of the home currently?	radio (Matrix)<br>1 always (100%)<br>2 most of the time (75%)<br>3 half of the time (50%)<br>4 less than half of the time (25%)<br>5 never (0%)

			6 don't know
1369	[hh2_work9_e_q2]<br><br>Show the field ONLY if: [language_q2] = '1' and ([hh2_work7_e_q2] = '1' or [hh2_work7_e_q2] = '2' or [hh2_work7_e_q2] = '3' or [hh2_work7_e_q2] = '4')	Person 2: How regularly is this person in close physical contact with clients during their work outside of the home currently?	radio (Matrix)<br>1 always (100%)<br>2 most of the time (75%)<br>3 half of the time (50%)<br>4 less than half of the time (25%)<br>5 never (0%)<br>6 don't know
1370	[hh2_covidvaccine_e_q2]<br><br>Show the field ONLY if: [language_q2] = '1' and [hhcount_e_q2] > 1 and [hhcount_e_q2] < 13	Person 2: Does this person plan to get a vaccine for COVID-19?	radio<br>1 Yes<br>0 No<br>2 Don't know<br>3 This individual has already been vaccinated<br><br>Field Annotation: @DEFAULT="[hh2_covidvaccine_e_q2]"
1371	[hh2_covidsymp_e_q2]<br><br>Show the field ONLY if: [language_q2] = '1' and [hhcount_e_q2] > 1 and [hhcount_e_q2] < 13	Person 2: Has this person had any symptoms (cough, fever, difficulty breathing, fatigue, body aches, diarrhea, runny nose, loss of smell or taste) consistent with COVID-19 in the last two weeks?	radio<br>1 yes<br>0 no<br>2 don't know
1372	[hh2_covidsymp2_e_q2]<br><br>Show the field ONLY if: [language_q2] = '1' and [hh2_covidsymp_e_q2] = '1'	Person 2: When did this person's symptoms begin?	text (date_mdy)
1373	[hh2_covidsymp3_e_q2]<br><br>Show the field ONLY if: [language_q2] = '1' and [hh2_covidsymp_e_q2] = '1'	Person 2: Is this person worried that they may have had COVID-19 because of their symptoms?	radio<br>1 yes<br>0 no<br>2 don't know
1374	[hh2_covidsymp4_e_q2]<br><br>Show the field ONLY if: [language_q2] = '1' and [hh2_covidsymp_e_q2] = '1'	Person 2: Did this person experience any bias or discrimination because of their symptoms?	radio<br>1 yes<br>0 no<br>2 don't know
1375	[hh2_covidsymp5_e_q2]<br><br>Show the field ONLY if: [language_q2] = '1' and [hh2_covidsymp_e_q2]	Person 2: What did this person do in response to their symptoms?<br>*Select all that apply.*	checkbox<br>0 hh2_covidsymp5_e_q2___0 nothing<br>1 hh2_covidsymp5_e_q2___1 took over the counter medication

	= '1'		(ibuprofen, acetaminoph etc.)<br>2 | hh2_covidsymp5_e_q2___2 | communicat with a health care provide over the pho<br>3 | hh2_covidsymp5_e_q2___3 | visited a hea care provide office<br>4 | hh2_covidsymp5_e_q2___4 | visited a reta clinic or pharmacy<br>5 | hh2_covidsymp5_e_q2___5 | visited urger care (FASTM etc.)<br>6 | hh2_covidsymp5_e_q2___6 | visited the emergency room<br>7 | hh2_covidsymp5_e_q2___7 | was admitte the hospital<br>8 | hh2_covidsymp5_e_q2___8 | other<br>9 | hh2_covidsymp5_e_q2___9 | don't know
1376	[hh2_covidsymp6_e_q2]<br><br>Show the field ONLY if: [language_q2] = '1' and [hh2_covidsymp5_e_q2(8)] = '1'	Person 2: Please specify what other action this person took in response to their symptoms.	text
1377	[hh2_covidsymp7_e_q2]<br><br>Show the field ONLY if: [language_q2] = '1' and ([hh2_covidsymp5_e_q2(2)] = '1' or [hh2_covidsymp5_e_q2(3)] = '1' or [hh2_covidsymp5_e_q2(4)] = '1' or [hh2_covidsymp5_e_q2(5)] = '1' or [hh2_covidsymp5_e_q2(6)] = '1' or [hh2_covidsymp5_e_q2(7)] = '1' or [hh2_covidsymp5_e_q2(8)] = '1')	Person 2: Did a health care provider tell this person that they may have COVID-19?	radio<br>1 | yes<br>0 | no<br>2 | don't know
1378	[hh2_covid_test_e_q2]<br><br>Show the field ONLY if: [language_q2] = '1' and [hh2_covidsymp_e_q2] = '1'	Person 2: If this person received a COVID-19 test due to their symptoms, what was the result?	radio<br>1 | pending<br>2 | positive<br>3 | negative<br>4 | inconclusive

			5 did not receive a test<br>6 don't know
1379	[hh2_covid_admit_e_q2]<br><br>Show the field ONLY if: [language_q2] = '1' and [hh2_covidsymp5_e_q2(7)] = '1'	Person 2: How many days was this person admitted to the hospital?	text (number, Min: 0)
1380	[hh2_covid_admit2_e_q2]<br><br>Show the field ONLY if: [language_q2] = '1' and [hh2_covidsymp5_e_q2(7)] = '1'	Person 2: Did this person receive any of the following interventions during their hospital admission?	checkbox<br>1 hh2_covid_admit2_e_q2___1 extra oxygen in your nose<br>2 hh2_covid_admit2_e_q2___2 treatment in the intensive care unit (ICU)<br>3 hh2_covid_admit2_e_q2___3 mechanical ventilation (intubation or a breathing tube)<br>4 hh2_covid_admit2_e_q2___4 don't know
1381	[hh2_covidsymp8_e_q2]<br><br>Show the field ONLY if: [language_q2] = '1' and [hh2_covidsymp_e_q2] = '1'	Person 2: Has this person returned to their normal health at this time?	radio<br>1 yes<br>0 no<br>2 don't know
1382	[hh2_prevent_e_q2]<br><br>Show the field ONLY if: [language_q2] = '1' and [hh2_covidsymp_e_q2] = '1'	Person 2: Which of the following did this person do to protect their friends and family after their symptoms began?	checkbox<br>1 hh2_prevent_e_q2___1 wore a mask more frequently<br>2 hh2_prevent_e_q2___2 washed your hands with soap and water more frequently<br>3 hh2_prevent_e_q2___3 used hand sanitizer more frequently<br>4 hh2_prevent_e_q2___4 isolated yourself in your home more frequently<br>5 hh2_prevent_e_q2___5 stayed home more

			frequently
			6 hh2_prevent_e_q2___6 wore disposable gloves more frequently<br>7 hh2_prevent_e_q2___7 don't know
1383	[hh3_relationship_e_q2]<br><br>Show the field ONLY if: [language_q2] = '1' and [hhcount_e_q2] > 2 and [hhcount_e_q2] < 13	Section Header: *For each additional person in the your household, please provide the following information.*<br><br>Person 3: What is your relationship to this person?	radio<br>1 partner or spouse<br>2 child<br>3 parent<br>4 sibling<br>5 other family member<br>6 in-home childcare provider or other caregiver<br>7 other<br><br>Field Annotation: @DEFAULT="[hh3_relationship_e_q2]"
1384	[hh3_relationship2_e_q2]<br><br>Show the field ONLY if: [language_q2] = '1' and [hh3_relationship_e_q2] = '7'	Person 3: Please specify your relationship with this person.	text<br>Field Annotation: @DEFAULT="[hh3_relationship2_e_q2]"
1385	[hh3_age_e_q2]<br><br>Show the field ONLY if: [language_q2] = '1' and [hhcount_e_q2] > 2 and [hhcount_e_q2] < 13	Person 3: What is this person's age?<br>*Please specify their age in years*	text (number, Min: 0, Max: 110)<br>Field Annotation: @DEFAULT="[hh3_age_e_q2]"
1386	[hh3_sex_e_q2]<br><br>Show the field ONLY if: [language_q2] = '1' and [hhcount_e_q2] > 2 and [hhcount_e_q2] < 13	Person 3: What is this person's sex?	radio<br>1 Female<br>2 Male<br>3 Other<br><br>Field Annotation: @DEFAULT="[hh3_sex_e_q2]"
1387	[hh3_race_e_q2]<br><br>Show the field ONLY if: [language_q2] = '1' and [hhcount_e_q2] > 2 and [hhcount_e_q2] < 13	Person 3: What is this person's race?<br>*Select all that apply.*	checkbox<br>1 hh3_race_e_q2___1 American Indian or Alaska Native<br>2 hh3_race_e_q2___2 Asian<br>3 hh3_race_e_q2___3 Black or African American<br>4 hh3_race_e_q2___4 Native Hawaiian or Pacific Islander<br>5 hh3_race_e_q2___5 White

			6 hh3_race_e_q2___6 Other<br>7 hh3_race_e_q2___7 don't know<br><br>Field Annotation: @DEFAULT="[hh3_race_e_q2]"
1388	[hh3_e_q2thn_e_q2]<br><br>Show the field ONLY if: [language_q2] = '1' and [hhcount_e_q2] > 2 and [hhcount_e_q2] < 13	Person 3: What is this person's ethnicity?	radio<br>1 Hispanic or Latino<br>2 Not Hispanic or Latino<br>3 Other<br>4 don't know<br><br>Field Annotation: @DEFAULT="[hh3_e_q2thn_e_q2]"
1389	[hh3_e_q2du_e_q2]<br><br>Show the field ONLY if: [language_q2] = '1' and [hhcount_e_q2] > 2 and [hhcount_e_q2] < 13	Person 3: What is the highest level of education or schooling this person has completed?	radio<br>1 never attended school<br>2 kindergarten - 8th grade<br>3 some high school<br>4 high school equivalency (GED)<br>5 high school graduate<br>6 some college<br>7 college graduate<br>8 graduate school or more<br>9 don't know<br><br>Field Annotation: @DEFAULT="[hh3_e_q2du_e_q2]"
1390	[hh3_work_e_q2]<br><br>Show the field ONLY if: [language_q2] = '1' and [hhcount_e_q2] > 2 and [hhcount_e_q2] < 13	Person 3: Which of the following best fit this person's current work situation?	radio<br>1 works full time<br>2 works part time<br>3 is looking for work/employment<br>4 retired<br>5 homemaker<br>6 student<br>7 on maternity/paternity leave<br>8 on illness/sick leave<br>9 on disability<br>10 other<br>11 don't know<br><br>Field Annotation: @DEFAULT="[hh3_work_e_q2]"
1391	[hh3_work2_e_q2]	Person 3: Does this person currently consider themselves self-employed (including as an	radio

	Show the field ONLY if: [language_q2] = '1' and [hhcount_e_q2] > 2 and [hhcount_e_q2] < 13	independent contractor or gig-economy worker)?	1 yes<br>0 no<br>2 don't know<br><br>Field Annotation: @DEFAULT=" [hh3_work2_e_q2]"
1392	[hh3_work3_e_q2]<br><br>Show the field ONLY if: [language_q2] = '1' and [hhcount_e_q2] > 2 and [hhcount_e_q2] < 13 and ([hh3_work_e_q2] = '1' or [hh3_work_e_q2] = '2' or [hh3_work2_e_q2] = '1')	Person 3: Does this person currently work in any of the following high-risk settings for COVID-19 transmission?	checkbox<br>1 hh3_work3_e_q2___1 healthcare setting (hospital, clinic, urgent care, etc.)<br>2 hh3_work3_e_q2___2 dense residential setting (nursing home, other long-term care facility)<br>3 hh3_work3_e_q2___3 prison or jail<br>4 hh3_work3_e_q2___4 meatpacking facility<br>5 hh3_work3_e_q2___5 shipping or distribution facility<br>6 hh3_work3_e_q2___6 high-volume retail facility (grocery store, etc.)<br>7 hh3_work3_e_q2___7 don't know<br><br>Field Annotation: @DEFAULT=" [hh3_work3_e_q2]"
1393	[hh3_work4_e_q2]<br><br>Show the field ONLY if: [language_q2] = '1' and [hhcount_e_q2] > 2 and [hhcount_e_q2] < 13 and ([hh3_work_e_q2] = '1' or [hh3_work_e_q2] = '2' or [hh3_work2_e_q2] = '1')	Person 3: Does this person's employer offer them any of the following benefits at their current main job?<br>*Select all that apply.*	checkbox<br>1 hh3_work4_e_q2___1 paid sick leave<br>2 hh3_work4_e_q2___2 paid vacation/personal leave<br>3 hh3_work4_e_q2___3 health insurance<br>4 hh3_work4_e_q2___4 disability insurance<br>5 hh3_work4_e_q2___5 retirement plan<br>6 hh3_work4_e_q2___6 other<br>7 hh3_work4_e_q2___7 don't know<br><br>Field Annotation: @DEFAULT=" [hh3_work4_e_q2]"

1394	[hh3_work5_e_q2]<br><br>Show the field ONLY if: [language_q2] = '1' and [hhcount_e_q2] > 2 and [hhcount_e_q2] < 13 and ([hh3_work_e_q2] = '1' or [hh3_work_e_q2] = '2' or [hh3_work2_e_q2] = '1')	Person 3: On a scale of 0 (definitely not going to happen) to 10 (definitely going to happen), how likely is it that this person will lose their job because of the COVID-19 pandemic?	text (number, Min: 0, Max: 10)<br>Field Annotation: @DEFAULT="[hh3_work5_e_q2]"
1395	[hh3_work6_e_q2]<br><br>Show the field ONLY if: [language_q2] = '1' and [hhcount_e_q2] > 2 and [hhcount_e_q2] < 13 and ([hh3_work_e_q2] = '1' or [hh3_work_e_q2] = '2' or [hh3_work2_e_q2] = '1')	Person 3: On a scale of 0 (definitely not going to happen) to 10 (definitely going to happen), how likely is it that this person will receive fewer work hours at their job because of the COVID-19 pandemic?	text (number, Min: 0, Max: 10)<br>Field Annotation: @DEFAULT="[hh3_work6_e_q2]"
1396	[hh3_work7_e_q2]<br><br>Show the field ONLY if: [language_q2] = '1' and [hhcount_e_q2] > 2 and [hhcount_e_q2] < 13 and ([hh3_work_e_q2] = '1' or [hh3_work_e_q2] = '2' or [hh3_work2_e_q2] = '1')	Person 3: How often is this person required to work from outside of the home currently?	radio (Matrix)<br>1 always (100%)<br>2 most of the time (75%)<br>3 half of the time (50%)<br>4 less than half of the time (25%)<br>5 never (0%)<br>6 don't know
1397	[hh3_work8_e_q2]<br><br>Show the field ONLY if: [language_q2] = '1' and ([hh3_work7_e_q2] = '1' or [hh3_work7_e_q2] = '2' or [hh3_work7_e_q2] = '3' or [hh3_work7_e_q2] = '4')	Person 3: How regularly is this person in close physical contact with co-workers during their work outside of the home currently?	radio (Matrix)<br>1 always (100%)<br>2 most of the time (75%)<br>3 half of the time (50%)<br>4 less than half of the time (25%)<br>5 never (0%)<br>6 don't know
1398	[hh3_work9_e_q2]<br><br>Show the field ONLY if: [language_q2] = '1' and ([hh3_work7_e_q2] = '1' or [hh3_work7_e_q2] = '2' or [hh3_work7_e_q2] = '3' or [hh3_work7_e_q2] = '4')	Person 3: How regularly is this person in close physical contact with clients during their work outside of the home currently?	radio (Matrix)<br>1 always (100%)<br>2 most of the time (75%)<br>3 half of the time (50%)<br>4 less than half of the time (25%)<br>5 never (0%)<br>6 don't know

1399	[hh3_covidvaccine_e_q2]<br><br>Show the field ONLY if: [language_q2] = '1' and [hhcount_e_q2] > 2 and [hhcount_e_q2] < 13	Person 3: Does this person plan to get a vaccine for COVID-19?	radio<br>1 Yes<br>0 No<br>2 Don't know<br>3 This individual has already been vaccinated<br><br>Field Annotation: @DEFAULT="[hh3_covidvaccine_e_q2]"
1400	[hh3_covidsymp_e_q2]<br><br>Show the field ONLY if: [language_q2] = '1' and [hhcount_e_q2] > 2 and [hhcount_e_q2] < 13	Person 3: Has this person had any symptoms (cough, fever, difficulty breathing, fatigue, body aches, diarrhea, runny nose, loss of smell or taste) consistent with COVID-19 in the last two weeks?	radio<br>1 yes<br>0 no<br>2 don't know
1401	[hh3_covidsymp2_e_q2]<br><br>Show the field ONLY if: [language_q2] = '1' and [hh3_covidsymp_e_q2] = '1'	Person 3: When did this person's symptoms begin?	text (date_mdy)
1402	[hh3_covidsymp3_e_q2]<br><br>Show the field ONLY if: [language_q2] = '1' and [hh3_covidsymp_e_q2] = '1'	Person 3: Is this person worried that they may have had COVID-19 because of their symptoms?	radio<br>1 yes<br>0 no<br>2 don't know
1403	[hh3_covidsymp4_e_q2]<br><br>Show the field ONLY if: [language_q2] = '1' and [hh3_covidsymp_e_q2] = '1'	Person 3: Did this person experience any bias or discrimination because of their symptoms?	radio<br>1 yes<br>0 no<br>2 don't know
1404	[hh3_covidsymp5_e_q2]<br><br>Show the field ONLY if: [language_q2] = '1' and [hh3_covidsymp_e_q2] = '1'	Person 3: What did this person do in response to their symptoms?<br>*Select all that apply.*	checkbox<br>0 hh3_covidsymp5_e_q2___0 nothing<br>1 hh3_covidsymp5_e_q2___1 took over the counter medication (ibuprofen, acetaminoph[illegible] etc.)<br>2 hh3_covidsymp5_e_q2___2 communicat[illegible] with a health care provide[illegible] over the pho[illegible]<br>3 hh3_covidsymp5_e_q2___3 visited a hea[illegible] care provide[illegible] office

			4 hh3_covidsymp5_e_q2___4 visited a reta[illegible] clinic or pharmacy<br>5 hh3_covidsymp5_e_q2___5 visited urger[illegible] care (FASTM[illegible] etc.)<br>6 hh3_covidsymp5_e_q2___6 visited the emergency room<br>7 hh3_covidsymp5_e_q2___7 was admitte[illegible] the hospital<br>8 hh3_covidsymp5_e_q2___8 other<br>9 hh3_covidsymp5_e_q2___9 don't know
1405	[hh3_covidsymp6_e_q2]<br><br>Show the field ONLY if: [language_q2] = '1' and [hh3_covidsymp5_e_q2(8)] = '1'	Person 3: Please specify what other action this person took in response to their symptoms.	text
1406	[hh3_covidsymp7_e_q2]<br><br>Show the field ONLY if: [language_q2] = '1' and ([hh3_covidsymp5_e_q2(2)] = '1' or [hh3_covidsymp5_e_q2(3)] = '1' or [hh3_covidsymp5_e_q2(4)] = '1' or [hh3_covidsymp5_e_q2(5)] = '1' or [hh3_covidsymp5_e_q2(6)] = '1' or [hh3_covidsymp5_e_q2(7)] = '1' or [hh3_covidsymp5_e_q2(8)] = '1')	Person 3: Did a health care provider tell this person that they may have COVID-19?	radio<br>1 yes<br>0 no<br>2 don't know
1407	[hh3_covid_test_e_q2]<br><br>Show the field ONLY if: [language_q2] = '1' and [hh3_covidsymp_e_q2] = '1'	Person 3: If this person received a COVID-19 test due to their symptoms, what was the result?	radio<br>1 pending<br>2 positive<br>3 negative<br>4 inconclusive<br>5 did not receive a test<br>6 don't know
1408	[hh3_covid_admit_e_q2]<br><br>Show the field ONLY if: [language_q2] = '1' and [hh3_covidsymp5_e_q2(7)] = '1'	Person 3: How many days was this person admitted to the hospital?	text (number, Min: 0)

1409	[hh3_covid_admit2_e_q2]<br><br>Show the field ONLY if: [language_q2] = '1' and [hh3_covidsymp5_e_q2(7)] = '1'	Person 3: Did this person receive any of the following interventions during their hospital admission?	checkbox<br>1 hh3_covid_admit2_e_q2___1 extra oxygen in your nose<br>2 hh3_covid_admit2_e_q2___2 treatment in the intensive care unit (ICU)<br>3 hh3_covid_admit2_e_q2___3 mechanical ventilation (intubation or a breathing tube)<br>4 hh3_covid_admit2_e_q2___4 don't know
1410	[hh3_covidsymp8_e_q2]<br><br>Show the field ONLY if: [language_q2] = '1' and [hh3_covidsymp_e_q2] = '1'	Person 3: Has this person returned to their normal health at this time?	radio<br>1 yes<br>0 no<br>2 don't know
1411	[hh3_prevent_e_q2]<br><br>Show the field ONLY if: [language_q2] = '1' and [hh3_covidsymp_e_q2] = '1'	Person 3: Which of the following did this person do to protect their friends and family after their symptoms began?	checkbox<br>1 hh3_prevent_e_q2___1 wore a mask more frequently<br>2 hh3_prevent_e_q2___2 washed your hands with soap and water more frequently<br>3 hh3_prevent_e_q2___3 used hand sanitizer more frequently<br>4 hh3_prevent_e_q2___4 isolated yourself in your home more frequently<br>5 hh3_prevent_e_q2___5 stayed home more frequently<br>6 hh3_prevent_e_q2___6 wore disposable gloves more frequently<br>7 hh3_prevent_e_q2___7 don't know
1412	[hh4_relationship_e_q2]	Section Header: *For each additional person in the your household, please provide the following information.*	radio<br>1 partner or spouse

	Show the field ONLY if: [language_q2] = '1' and [hhcount_e_q2] > 3 and [hhcount_e_q2] < 13	Person 4: What is your relationship to this person?	2 child<br>3 parent<br>4 sibling<br>5 other family member<br>6 in-home childcare provider or other caregiver<br>7 other<br><br>Field Annotation: @DEFAULT="[hh4_relationship_e_q2]"
1413	[hh4_relationship2_e_q2]<br><br>Show the field ONLY if: [language_q2] = '1' and [hh4_relationship_e_q2] = '7'	Person 4: Please specify your relationship with this person.	text<br>Field Annotation: @DEFAULT="[hh4_relationship2_e_q2]"
1414	[hh4_age_e_q2]<br><br>Show the field ONLY if: [language_q2] = '1' and [hhcount_e_q2] > 3 and [hhcount_e_q2] < 13	Person 4: What is this person's age?<br>*Please specify their age in years*	text (number, Min: 0, Max: 110)<br>Field Annotation: @DEFAULT="[hh4_age_e_q2]"
1415	[hh4_sex_e_q2]<br><br>Show the field ONLY if: [language_q2] = '1' and [hhcount_e_q2] > 3 and [hhcount_e_q2] < 13	Person 4: What is this person's sex?	radio<br>1 Female<br>2 Male<br>3 Other<br><br>Field Annotation: @DEFAULT="[hh4_sex_e_q2]"
1416	[hh4_race_e_q2]<br><br>Show the field ONLY if: [language_q2] = '1' and [hhcount_e_q2] > 3 and [hhcount_e_q2] < 13	Person 4: What is this person's race?<br>*Select all that apply.*	checkbox<br>1 hh4_race_e_q2___1 American Indian or Alaska Native<br>2 hh4_race_e_q2___2 Asian<br>3 hh4_race_e_q2___3 Black or African American<br>4 hh4_race_e_q2___4 Native Hawaiian or Pacific Islander<br>5 hh4_race_e_q2___5 White<br>6 hh4_race_e_q2___6 Other<br>7 hh4_race_e_q2___7 don't know<br><br>Field Annotation: @DEFAULT="[hh4_race_e_q2]"
1417	[hh4_e_q2thn_e_q2]<br><br>Show the field ONLY if:	Person 4: What is this person's ethnicity?	radio<br>1 Hispanic or Latino

	[language_q2] = '1' and [hhcount_e_q2] > 3 and [hhcount_e_q2] < 13		2 Not Hispanic or Latino<br>3 Other<br>4 don't know<br><br>Field Annotation: @DEFAULT="[hh4_e_q2thn_e_q2]"
1418	[hh4_e_q2du_e_q2]<br><br>Show the field ONLY if: [language_q2] = '1' and [hhcount_e_q2] > 3 and [hhcount_e_q2] < 13	Person 4: What is the highest level of education or schooling this person has completed?	radio<br>1 never attended school<br>2 kindergarten - 8th grade<br>3 some high school<br>4 high school equivalency (GED)<br>5 high school graduate<br>6 some college<br>7 college graduate<br>8 graduate school or more<br>9 don't know<br><br>Field Annotation: @DEFAULT="[hh4_e_q2du_e_q2]"
1419	[hh4_work_e_q2]<br><br>Show the field ONLY if: [language_q2] = '1' and [hhcount_e_q2] > 3 and [hhcount_e_q2] < 13	Person 4: Which of the following best fit this person's current work situation?	radio<br>1 works full time<br>2 works part time<br>3 is looking for work/employment<br>4 retired<br>5 homemaker<br>6 student<br>7 on maternity/paternity leave<br>8 on illness/sick leave<br>9 on disability<br>10 other<br>11 don't know<br><br>Field Annotation: @DEFAULT="[hh4_work_e_q2]"
1420	[hh4_work2_e_q2]<br><br>Show the field ONLY if: [language_q2] = '1' and [hhcount_e_q2] > 3 and [hhcount_e_q2] < 13	Person 4: Does this person currently consider themselves self-employed (including as an independent contractor or gig-economy worker)?	radio<br>1 yes<br>0 no<br>2 don't know<br><br>Field Annotation: @DEFAULT="[hh4_work2_e_q2]"
1421	[hh4_work3_e_q2]	Person 4: Does this person currently work in any of the following high-risk settings for COVID-19	checkbox

	Show the field ONLY if: [language_q2] = '1' and [hhcount_e_q2] > 3 and [hhcount_e_q2] < 13 and ([hh4_work_e_q2] = '1' or [hh4_work_e_q2] = '2' or [hh4_work2_e_q2] = '1')	transmission?	1 hh4_work3_e_q2___1 healthcare setting (hospital, clinic, urgent care, etc.)<br>2 hh4_work3_e_q2___2 dense residential setting (nursing home, other long-term care facility)<br>3 hh4_work3_e_q2___3 prison or jail<br>4 hh4_work3_e_q2___4 meatpacking facility<br>5 hh4_work3_e_q2___5 shipping or distribution facility<br>6 hh4_work3_e_q2___6 high-volume retail facility (grocery store, etc.)<br>7 hh4_work3_e_q2___7 don't know<br><br>Field Annotation: @DEFAULT="[hh4_work3_e_q2]"
1422	[hh4_work4_e_q2]<br><br>Show the field ONLY if: [language_q2] = '1' and [hhcount_e_q2] > 3 and [hhcount_e_q2] < 13 and ([hh4_work_e_q2] = '1' or [hh4_work_e_q2] = '2' or [hh4_work2_e_q2] = '1')	Person 4: Does this person's employer offer them any of the following benefits at their current main job?<br>*Select all that apply.*	checkbox<br>1 hh4_work4_e_q2___1 paid sick leave<br>2 hh4_work4_e_q2___2 paid vacation/personal leave<br>3 hh4_work4_e_q2___3 health insurance<br>4 hh4_work4_e_q2___4 disability insurance<br>5 hh4_work4_e_q2___5 retirement plan<br>6 hh4_work4_e_q2___6 other<br>7 hh4_work4_e_q2___7 don't know<br><br>Field Annotation: @DEFAULT="[hh4_work4_e_q2]"
1423	[hh4_work5_e_q2]<br><br>Show the field ONLY if: [language_q2] = '1' and [hhcount_e_q2] > 3 and [hhcount_e_q2] < 13 and ([hh4_work_e_q2] = '1' or [hh4_work_e_q2] = '2' or [hh4_work2_e_q2] = '1')	Person 4: On a scale of 0 (definitely not going to happen) to 10 (definitely going to happen), how likely is it that this person will lose their job because of the COVID-19 pandemic?	text (number, Min: 0, Max: 10)<br>Field Annotation: @DEFAULT="[hh4_work5_e_q2]"

1424	[hh4_work6_e_q2]<br><br>Show the field ONLY if:<br>[language_q2] = '1' and [hhcount_e_q2] > 3 and [hhcount_e_q2] < 13 and ([hh4_work_e_q2] = '1' or [hh4_work_e_q2] = '2' or [hh4_work2_e_q2] = '1')	Person 4: On a scale of 0 (definitely not going to happen) to 10 (definitely going to happen), how likely is it that this person will receive fewer work hours at their job because of the COVID-19 pandemic?	text (number, Min: 0, Max: 10)<br>Field Annotation: @DEFAULT="[hh4_work6_e_q2]"
1425	[hh4_work7_e_q2]<br><br>Show the field ONLY if:<br>[language_q2] = '1' and [hhcount_e_q2] > 3 and [hhcount_e_q2] < 13 and ([hh4_work_e_q2] = '1' or [hh4_work_e_q2] = '2' or [hh4_work2_e_q2] = '1')	Person 4: How often is this person required to work from outside of the home currently?	radio (Matrix)<br>1 always (100%)<br>2 most of the time (75%)<br>3 half of the time (50%)<br>4 less than half of the time (25%)<br>5 never (0%)<br>6 don't know
1426	[hh4_work8_e_q2]<br><br>Show the field ONLY if:<br>[language_q2] = '1' and ([hh4_work7_e_q2] = '1' or [hh4_work7_e_q2] = '2' or [hh4_work7_e_q2] = '3' or [hh4_work7_e_q2] = '4')	Person 4: How regularly is this person in close physical contact with co-workers during their work outside of the home currently?	radio (Matrix)<br>1 always (100%)<br>2 most of the time (75%)<br>3 half of the time (50%)<br>4 less than half of the time (25%)<br>5 never (0%)<br>6 don't know
1427	[hh4_work9_e_q2]<br><br>Show the field ONLY if:<br>[language_q2] = '1' and ([hh4_work7_e_q2] = '1' or [hh4_work7_e_q2] = '2' or [hh4_work7_e_q2] = '3' or [hh4_work7_e_q2] = '4')	Person 4: How regularly is this person in close physical contact with clients during their work outside of the home currently?	radio (Matrix)<br>1 always (100%)<br>2 most of the time (75%)<br>3 half of the time (50%)<br>4 less than half of the time (25%)<br>5 never (0%)<br>6 don't know
1428	[hh4_covidvaccine_e_q2]<br><br>Show the field ONLY if:<br>[language_q2] = '1' and [hhcount_e_q2] > 3 and [hhcount_e_q2] < 13	Person 4: Does this person plan to get a vaccine for COVID-19?	radio<br>1 Yes<br>0 No<br>2 Don't know<br>3 This individual has already been vaccinated<br><br>Field Annotation: @DEFAULT="[hh4_covidvaccine_e_q2]"
1429	[hh4_covidsymp_e_q2]<br><br>Show the field ONLY if:	Person 4: Has this person had any symptoms (cough, fever, difficulty breathing, fatigue, body aches, diarrhea, runny nose, loss of smell or taste) consistent with COVID-19 in the last two weeks?	radio<br>1 yes<br>0 no

	[language_q2] = '1' and [hhcount_e_q2] > 3 and [hhcount_e_q2] < 13		2 don't know
1430	[hh4_covidsymp2_e_q2]<br><br>Show the field ONLY if: [language_q2] = '1' and [hh4_covidsymp_e_q2] = '1'	Person 4: When did this person's symptoms begin?	text (date_mdy)
1431	[hh4_covidsymp3_e_q2]<br><br>Show the field ONLY if: [language_q2] = '1' and [hh4_covidsymp_e_q2] = '1'	Person 4: Is this person worried that they may have had COVID-19 because of their symptoms?	radio<br>1 yes<br>0 no<br>2 don't know
1432	[hh4_covidsymp4_e_q2]<br><br>Show the field ONLY if: [language_q2] = '1' and [hh4_covidsymp_e_q2] = '1'	Person 4: Did this person experience any bias or discrimination because of their symptoms?	radio<br>1 yes<br>0 no<br>2 don't know
1433	[hh4_covidsymp5_e_q2]<br><br>Show the field ONLY if: [language_q2] = '1' and [hh4_covidsymp_e_q2] = '1'	Person 4: What did this person do in response to their symptoms?<br>*Select all that apply.*	checkbox<br>0 hh4_covidsymp5_e_q2___0 nothing<br>1 hh4_covidsymp5_e_q2___1 took over th counter medication (ibuprofen, acetaminop etc.)<br>2 hh4_covidsymp5_e_q2___2 communica with a healt care provide over the pho<br>3 hh4_covidsymp5_e_q2___3 visited a hea care provide office<br>4 hh4_covidsymp5_e_q2___4 visited a reta clinic or pharmacy<br>5 hh4_covidsymp5_e_q2___5 visited urge care (FASTM etc.)<br>6 hh4_covidsymp5_e_q2___6 visited the emergency room<br>7 hh4_covidsymp5_e_q2___7 was admitte the hospital<br>8 hh4_covidsymp5_e_q2___8 other<br>9 hh4_covidsymp5_e_q2___9 don't know

1434	[hh4_covidsymp6_e_q2]<br><br>Show the field ONLY if:<br>[language_q2] = '1' and [hh4_covidsymp5_e_q2(8)] = '1'	Person 4: Please specify what other action this person took in response to their symptoms.	text
1435	[hh4_covidsymp7_e_q2]<br><br>Show the field ONLY if:<br>[language_q2] = '1' and ([hh4_covidsymp5_e_q2(2)] = '1' or [hh4_covidsymp5_e_q2(3)] = '1' or [hh4_covidsymp5_e_q2(4)] = '1' or [hh4_covidsymp5_e_q2(5)] = '1' or [hh4_covidsymp5_e_q2(6)] = '1' or [hh4_covidsymp5_e_q2(7)] = '1' or [hh4_covidsymp5_e_q2(8)] = '1')	Person 4: Did a health care provider tell this person that they may have COVID-19?	radio<br>1 yes<br>0 no<br>2 don't know
1436	[hh4_covid_test_e_q2]<br><br>Show the field ONLY if:<br>[language_q2] = '1' and [hh4_covidsymp_e_q2] = '1'	Person 4: If this person received a COVID-19 test due to their symptoms, what was the result?	radio<br>1 pending<br>2 positive<br>3 negative<br>4 inconclusive<br>5 did not receive a test<br>6 don't know
1437	[hh4_covid_admit_e_q2]<br><br>Show the field ONLY if:<br>[language_q2] = '1' and [hh4_covidsymp5_e_q2(7)] = '1'	Person 4: How many days was this person admitted to the hospital?	text (number, Min: 0)
1438	[hh4_covid_admit2_e_q2]<br><br>Show the field ONLY if:<br>[language_q2] = '1' and [hh4_covidsymp5_e_q2(7)] = '1'	Person 4: Did this person receive any of the following interventions during their hospital admission?	checkbox<br>1 hh4_covid_admit2_e_q2___1 extra oxygen in your nose<br>2 hh4_covid_admit2_e_q2___2 treatment in the intensive care unit (ICU)<br>3 hh4_covid_admit2_e_q2___3 mechanical ventilation (intubation or a breathing tube)

			4 | hh4_covid_admit2_e_q2___4 | don't know
1439	[hh4_covidsymp8_e_q2]<br>Show the field ONLY if: [language_q2] = '1' and [hh4_covidsymp_e_q2] = '1'	Person 4: Has this person returned to their normal health at this time?	radio<br>1 | yes<br>0 | no<br>2 | don't know
1440	[hh4_prevent_e_q2]<br>Show the field ONLY if: [language_q2] = '1' and [hh4_covidsymp_e_q2] = '1'	Person 4: Which of the following did this person do to protect their friends and family after their symptoms began?	checkbox<br>1 | hh4_prevent_e_q2___1 | wore a mask more frequently<br>2 | hh4_prevent_e_q2___2 | washed your hands with soap and water more frequently<br>3 | hh4_prevent_e_q2___3 | used hand sanitizer more frequently<br>4 | hh4_prevent_e_q2___4 | isolated yourself in your home more frequently<br>5 | hh4_prevent_e_q2___5 | stayed home more frequently<br>6 | hh4_prevent_e_q2___6 | wore disposable gloves more frequently<br>7 | hh4_prevent_e_q2___7 | don't know
1441	[hh5_relationship_e_q2]<br>Show the field ONLY if: [language_q2] = '1' and [hhcount_e_q2] > 4 and [hhcount_e_q2] < 13	Section Header: *For each additional person in the your household, please provide the following information.*<br>Person 5: What is your relationship to this person?	radio<br>1 | partner or spouse<br>2 | child<br>3 | parent<br>4 | sibling<br>5 | other family member<br>6 | in-home childcare provider or other caregiver<br>7 | other<br>Field Annotation: @DEFAULT="[hh5_relationship_e_q2]"
1442	[hh5_relationship2_e_q2]	Person 5: Please specify your relationship with this person.	text<br>Field Annotation: @DEFAULT="[hh5_relationship2_e_q2]"

	Show the field ONLY if: [language_q2] = '1' and [hh5_relationship_e_q2] = '7'		
1443	[hh5_age_e_q2]<br><br>Show the field ONLY if: [language_q2] = '1' and [hhcount_e_q2] > 4 and [hhcount_e_q2] < 13	Person 5: What is this person's age?<br>*Please specify their age in years*	text (number, Min: 0, Max: 110)<br>Field Annotation: @DEFAULT="[hh5_age_e_q2]"
1444	[hh5_sex_e_q2]<br><br>Show the field ONLY if: [language_q2] = '1' and [hhcount_e_q2] > 4 and [hhcount_e_q2] < 13	Person 5: What is this person's sex?	radio<br>1 Female<br>2 Male<br>3 Other<br><br>Field Annotation: @DEFAULT="[hh5_sex_e_q2]"
1445	[hh5_race_e_q2]<br><br>Show the field ONLY if: [language_q2] = '1' and [hhcount_e_q2] > 4 and [hhcount_e_q2] < 13	Person 5: What is this person's race?<br>*Select all that apply.*	checkbox<br>1 hh5_race_e_q2___1 American Indian or Alaska Native<br>2 hh5_race_e_q2___2 Asian<br>3 hh5_race_e_q2___3 Black or African American<br>4 hh5_race_e_q2___4 Native Hawaiian or Pacific Islander<br>5 hh5_race_e_q2___5 White<br>6 hh5_race_e_q2___6 Other<br>7 hh5_race_e_q2___7 don't know<br><br>Field Annotation: @DEFAULT="[hh5_race_e_q2]"
1446	[hh5_e_q2thn_e_q2]<br><br>Show the field ONLY if: [language_q2] = '1' and [hhcount_e_q2] > 4 and [hhcount_e_q2] < 13	Person 5: What is this person's ethnicity?	radio<br>1 Hispanic or Latino<br>2 Not Hispanic or Latino<br>3 Other<br>4 don't know<br><br>Field Annotation: @DEFAULT="[hh5_e_q2thn_e_q2]"
1447	[hh5_e_q2du_e_q2]<br><br>Show the field ONLY if: [language_q2] = '1' and [hhcount_e_q2] > 4 and [hhcount_e_q2] < 13	Person 5: What is the highest level of education or schooling this person has completed?	radio<br>1 never attended school<br>2 kindergarten - 8th grade<br>3 some high school<br>4 high school equivalency (GED)<br>5 high school graduate

			6 some college<br>7 college graduate<br>8 graduate school or more<br>9 don't know<br><br>Field Annotation: @DEFAULT="[hh5_e_q2du_e_q2]"
1448	[hh5_work_e_q2]<br><br>Show the field ONLY if: [language_q2] = '1' and [hhcount_e_q2] > 4 and [hhcount_e_q2] < 13	Person 5: Which of the following best fit this person's current work situation?	radio<br>1 works full time<br>2 works part time<br>3 is looking for work/employment<br>4 retired<br>5 homemaker<br>6 student<br>7 on maternity/paternity leave<br>8 on illness/sick leave<br>9 on disability<br>10 other<br>11 don't know<br><br>Field Annotation: @DEFAULT="[hh5_work_e_q2]"
1449	[hh5_work2_e_q2]<br><br>Show the field ONLY if: [language_q2] = '1' and [hhcount_e_q2] > 4 and [hhcount_e_q2] < 13	Person 5: Does this person currently consider themselves self-employed (including as an independent contractor or gig-economy worker)?	radio<br>1 yes<br>0 no<br>2 don't know<br><br>Field Annotation: @DEFAULT="[hh5_work2_e_q2]"
1450	[hh5_work3_e_q2]<br><br>Show the field ONLY if: [language_q2] = '1' and [hhcount_e_q2] > 4 and [hhcount_e_q2] < 13 and ([hh5_work_e_q2] = '1' or [hh5_work_e_q2] = '2' or [hh5_work2_e_q2] = '1')	Person 5: Does this person currently work in any of the following high-risk settings for COVID-19 transmission?	checkbox<br>1 hh5_work3_e_q2___1 healthcare setting (hospital, clinic, urgent care, etc.)<br>2 hh5_work3_e_q2___2 dense residential setting (nursing home, other long-term care facility)<br>3 hh5_work3_e_q2___3 prison or jail<br>4 hh5_work3_e_q2___4 meatpacking facility

			5 | hh5_work3_e_q2___5 | shipping or distribution facility<br>6 | hh5_work3_e_q2___6 | high-volume retail facility (grocery store, etc.)<br>7 | hh5_work3_e_q2___7 | don't know<br><br>Field Annotation: @DEFAULT="[hh5_work3_e_q2]"
1451	[hh5_work4_e_q2]<br>Show the field ONLY if: [language_q2] = '1' and [hhcount_e_q2] > 4 and [hhcount_e_q2] < 13 and ([hh5_work_e_q2] = '1' or [hh5_work_e_q2] = '2' or [hh5_work2_e_q2] = '1')	Person 5: Does this person's employer offer them any of the following benefits at their current main job?<br>*Select all that apply.*	checkbox<br>1 | hh5_work4_e_q2___1 | paid sick leave<br>2 | hh5_work4_e_q2___2 | paid vacation/personal leave<br>3 | hh5_work4_e_q2___3 | health insurance<br>4 | hh5_work4_e_q2___4 | disability insurance<br>5 | hh5_work4_e_q2___5 | retirement plan<br>6 | hh5_work4_e_q2___6 | other<br>7 | hh5_work4_e_q2___7 | don't know<br><br>Field Annotation: @DEFAULT="[hh5_work4_e_q2]"
1452	[hh5_work5_e_q2]<br>Show the field ONLY if: [language_q2] = '1' and [hhcount_e_q2] > 4 and [hhcount_e_q2] < 13 and ([hh5_work_e_q2] = '1' or [hh5_work_e_q2] = '2' or [hh5_work2_e_q2] = '1')	Person 5: On a scale of 0 (definitely not going to happen) to 10 (definitely going to happen), how likely is it that this person will lose their job because of the COVID-19 pandemic?	text (number, Min: 0, Max: 10)<br>Field Annotation: @DEFAULT="[hh5_work5_e_q2]"
1453	[hh5_work6_e_q2]<br>Show the field ONLY if: [language_q2] = '1' and [hhcount_e_q2] > 4 and [hhcount_e_q2] < 13 and ([hh5_work_e_q2] = '1' or [hh5_work_e_q2] = '2' or [hh5_work2_e_q2] = '1')	Person 5: On a scale of 0 (definitely not going to happen) to 10 (definitely going to happen), how likely is it that this person will receive fewer work hours at their job because of the COVID-19 pandemic?	text (number, Min: 0, Max: 10)<br>Field Annotation: @DEFAULT="[hh5_work6_e_q2]"
1454	[hh5_work7_e_q2]<br>Show the field ONLY if: [language_q2] = '1' and [hhcount_e_q2] > 4 and [hhcount_e_q2] < 13 and ([hh5_work_e_q2] =	Person 5: How often is this person required to work from outside of the home currently?	radio (Matrix)<br>1 | always (100%)<br>2 | most of the time (75%)<br>3 | half of the time (50%)<br>4 | less than half of the time (25%)

	'1' or [hh5_work_e_q2] = '2' or [hh5_work2_e_q2] = '1')		5 never (0%)<br>6 don't know
1455	[hh5_work8_e_q2]<br><br>Show the field ONLY if: [language_q2] = '1' and ([hh5_work7_e_q2] = '1' or [hh5_work7_e_q2] = '2' or [hh5_work7_e_q2] = '3' or [hh5_work7_e_q2] = '4')	Person 5: How regularly is this person in close physical contact with co-workers during their work outside of the home currently?	radio (Matrix)<br>1 always (100%)<br>2 most of the time (75%)<br>3 half of the time (50%)<br>4 less than half of the time (25%)<br>5 never (0%)<br>6 don't know
1456	[hh5_work9_e_q2]<br><br>Show the field ONLY if: [language_q2] = '1' and ([hh5_work7_e_q2] = '1' or [hh5_work7_e_q2] = '2' or [hh5_work7_e_q2] = '3' or [hh5_work7_e_q2] = '4')	Person 5: How regularly is this person in close physical contact with clients during their work outside of the home currently?	radio (Matrix)<br>1 always (100%)<br>2 most of the time (75%)<br>3 half of the time (50%)<br>4 less than half of the time (25%)<br>5 never (0%)<br>6 don't know
1457	[hh5_covidvaccine_e_q2]<br><br>Show the field ONLY if: [language_q2] = '1' and [hhcount_e_q2] > 4 and [hhcount_e_q2] < 13	Person 5: Does this person plan to get a vaccine for COVID-19?	radio<br>1 Yes<br>0 No<br>2 Don't know<br>3 This individual has already been vaccinated<br><br>Field Annotation: @DEFAULT=" [hh5_covidvaccine_e_q2]"
1458	[hh5_covidsymp_e_q2]<br><br>Show the field ONLY if: [language_q2] = '1' and [hhcount_e_q2] > 4 and [hhcount_e_q2] < 13	Person 5: Has this person had any symptoms (cough, fever, difficulty breathing, fatigue, body aches, diarrhea, runny nose, loss of smell or taste) consistent with COVID-19 in the last two weeks?	radio<br>1 yes<br>0 no<br>2 don't know
1459	[hh5_covidsymp2_e_q2]<br><br>Show the field ONLY if: [language_q2] = '1' and [hh5_covidsymp_e_q2] = '1'	Person 5: When did this person's symptoms begin?	text (date_mdy)
1460	[hh5_covidsymp3_e_q2]<br><br>Show the field ONLY if: [language_q2] = '1' and [hh5_covidsymp_e_q2] = '1'	Person 5: Is this person worried that they may have had COVID-19 because of their symptoms?	radio<br>1 yes<br>0 no<br>2 don't know

1461	[hh5_covidsymp4_e_q2]<br><br>Show the field ONLY if: [language_q2] = '1' and [hh5_covidsymp_e_q2] = '1'	Person 5: Did this person experience any bias or discrimination because of their symptoms?	radio<br>1 yes<br>0 no<br>2 don't know
1462	[hh5_covidsymp5_e_q2]<br><br>Show the field ONLY if: [language_q2] = '1' and [hh5_covidsymp_e_q2] = '1'	Person 5: What did this person do in response to their symptoms?<br>*Select all that apply.*	checkbox<br>0 hh5_covidsymp5_e_q2___0 nothing<br>1 hh5_covidsymp5_e_q2___1 took over the counter medication (ibuprofen, acetaminoph[illegible] etc.)<br>2 hh5_covidsymp5_e_q2___2 communicat[illegible] with a health care provide[illegible] over the pho[illegible]<br>3 hh5_covidsymp5_e_q2___3 visited a hea[illegible] care provide[illegible] office<br>4 hh5_covidsymp5_e_q2___4 visited a reta[illegible] clinic or pharmacy<br>5 hh5_covidsymp5_e_q2___5 visited urge[illegible] care (FASTM[illegible] etc.)<br>6 hh5_covidsymp5_e_q2___6 visited the emergency room<br>7 hh5_covidsymp5_e_q2___7 was admitte[illegible] the hospital<br>8 hh5_covidsymp5_e_q2___8 other<br>9 hh5_covidsymp5_e_q2___9 don't know
1463	[hh5_covidsymp6_e_q2]<br><br>Show the field ONLY if: [language_q2] = '1' and [hh5_covidsymp5_e_q2(8)] = '1'	Person 5: Please specify what other action this person took in response to their symptoms.	text
1464	[hh5_covidsymp7_e_q2]<br><br>Show the field ONLY if: [language_q2] = '1' and ([hh5_covidsymp5_e_q2(2)] = '1' or [hh5_covidsymp5_e_q2(3)] = '1' or [hh5_covidsymp5_e_q2(4)] = '1' or [hh5_covidsymp5_e_q2(5)] = '1' or	Person 5: Did a health care provider tell this person that they may have COVID-19?	radio<br>1 yes<br>0 no<br>2 don't know

	[hh5_covidsymp5_e_q2(6)] = '1' or [hh5_covidsymp5_e_q2(7)] = '1' or [hh5_covidsymp5_e_q2(8)] = '1')		
1465	[hh5_covid_test_e_q2]<br><br>Show the field ONLY if:<br>[language_q2] = '1' and [hh5_covidsymp_e_q2] = '1'	Person 5: If this person received a COVID-19 test due to their symptoms, what was the result?	radio<br>1 pending<br>2 positive<br>3 negative<br>4 inconclusive<br>5 did not receive a test<br>6 don't know
1466	[hh5_covid_admit_e_q2]<br><br>Show the field ONLY if:<br>[language_q2] = '1' and [hh5_covidsymp5_e_q2(7)] = '1'	Person 5: How many days was this person admitted to the hospital?	text (number, Min: 0)
1467	[hh5_covid_admit2_e_q2]<br><br>Show the field ONLY if:<br>[language_q2] = '1' and [hh5_covidsymp5_e_q2(7)] = '1'	Person 5: Did this person receive any of the following interventions during their hospital admission?	checkbox<br>1 hh5_covid_admit2_e_q2___1 extra oxygen in your nose<br>2 hh5_covid_admit2_e_q2___2 treatment in the intensive care unit (ICU)<br>3 hh5_covid_admit2_e_q2___3 mechanical ventilation (intubation or a breathing tube)<br>4 hh5_covid_admit2_e_q2___4 don't know
1468	[hh5_covidsymp8_e_q2]<br><br>Show the field ONLY if:<br>[language_q2] = '1' and [hh5_covidsymp_e_q2] = '1'	Person 5: Has this person returned to their normal health at this time?	radio<br>1 yes<br>0 no<br>2 don't know
1469	[hh5_prevent_e_q2]<br><br>Show the field ONLY if:<br>[language_q2] = '1' and [hh5_covidsymp_e_q2] = '1'	Person 5: Which of the following did this person do to protect their friends and family after their symptoms began?	checkbox<br>1 hh5_prevent_e_q2___1 wore a mask more frequently

			2 | hh5_prevent_e_q2___2 | washed your hands with soap and water more frequently<br>3 | hh5_prevent_e_q2___3 | used hand sanitizer more frequently<br>4 | hh5_prevent_e_q2___4 | isolated yourself in your home more frequently<br>5 | hh5_prevent_e_q2___5 | stayed home more frequently<br>6 | hh5_prevent_e_q2___6 | wore disposable gloves more frequently<br>7 | hh5_prevent_e_q2___7 | don't know
1470	[hh6_relationship_e_q2]<br>Show the field ONLY if: [language_q2] = '1' and [hhcount_e_q2] > 5 and [hhcount_e_q2] < 13	Section Header: *For each additional person in the your household, please provide the following information.*<br>Person 6: What is your relationship to this person?	radio<br>1 | partner or spouse<br>2 | child<br>3 | parent<br>4 | sibling<br>5 | other family member<br>6 | in-home childcare provider or other caregiver<br>7 | other<br>Field Annotation: @DEFAULT="[hh6_relationship_e_q2]"
1471	[hh6_relationship2_e_q2]<br>Show the field ONLY if: [language_q2] = '1' and [hh6_relationship_e_q2] = '7'	Person 6: Please specify your relationship with this person.	text<br>Field Annotation: @DEFAULT="[hh6_relationship2_e_q2]"
1472	[hh6_age_e_q2]<br>Show the field ONLY if: [language_q2] = '1' and [hhcount_e_q2] > 5 and [hhcount_e_q2] < 13	Person 6: What is this person's age?<br>*Please specify their age in years*	text (number, Min: 0, Max: 110)<br>Field Annotation: @DEFAULT="[hh6_age_e_q2]"
1473	[hh6_sex_e_q2]<br>Show the field ONLY if:	Person 6: What is this person's sex?	radio<br>1 | Female

	[language_q2] = '1' and [hhcount_e_q2] > 5 and [hhcount_e_q2] < 13		2 Male<br>3 Other<br><br>Field Annotation: @DEFAULT=" [hh6_sex_e_q2]"
1474	[hh6_race_e_q2]<br><br>Show the field ONLY if: [language_q2] = '1' and [hhcount_e_q2] > 5 and [hhcount_e_q2] < 13	Person 6: What is this person's race?<br>*Select all that apply.*	checkbox<br>1 hh6_race_e_q2___1 American Indian or Alaska Native<br>2 hh6_race_e_q2___2 Asian<br>3 hh6_race_e_q2___3 Black or African American<br>4 hh6_race_e_q2___4 Native Hawaiian or Pacific Islander<br>5 hh6_race_e_q2___5 White<br>6 hh6_race_e_q2___6 Other<br>7 hh6_race_e_q2___7 don't know<br><br>Field Annotation: @DEFAULT=" [hh6_race_e_q2]"
1475	[hh6_e_q2thn_e_q2]<br><br>Show the field ONLY if: [language_q2] = '1' and [hhcount_e_q2] > 5 and [hhcount_e_q2] < 13	Person 6: What is this person's ethnicity?	radio<br>1 Hispanic or Latino<br>2 Not Hispanic or Latino<br>3 Other<br>4 don't know<br><br>Field Annotation: @DEFAULT=" [hh6_e_q2thn_e_q2]"
1476	[hh6_e_q2du_e_q2]<br><br>Show the field ONLY if: [language_q2] = '1' and [hhcount_e_q2] > 5 and [hhcount_e_q2] < 13	Person 6: What is the highest level of education or schooling this person has completed?	radio<br>1 never attended school<br>2 kindergarten - 8th grade<br>3 some high school<br>4 high school equivalency (GED)<br>5 high school graduate<br>6 some college<br>7 college graduate<br>8 graduate school or more<br>9 don't know<br><br>Field Annotation: @DEFAULT=" [hh6_e_q2du_e_q2]"
1477	[hh6_work_e_q2]<br><br>Show the field ONLY if:	Person 6: Which of the following best fit this person's current work situation?	radio<br>1 works full time<br>2 works part time

	[language_q2] = '1' and [hhcount_e_q2] > 5 and [hhcount_e_q2] < 13		3 is looking for work/employment<br>4 retired<br>5 homemaker<br>6 student<br>7 on maternity/paternity leave<br>8 on illness/sick leave<br>9 on disability<br>10 other<br>11 don't know<br><br>Field Annotation: @DEFAULT=" [hh6_work_e_q2]"
1478	[hh6_work2_e_q2]<br><br>Show the field ONLY if: [language_q2] = '1' and [hhcount_e_q2] > 5 and [hhcount_e_q2] < 13	Person 6: Does this person currently consider themselves self-employed (including as an independent contractor or gig-economy worker)?	radio<br>1 yes<br>0 no<br>2 don't know<br><br>Field Annotation: @DEFAULT=" [hh6_work2_e_q2]"
1479	[hh6_work3_e_q2]<br><br>Show the field ONLY if: [language_q2] = '1' and [hhcount_e_q2] > 5 and [hhcount_e_q2] < 13 and ([hh6_work_e_q2] = '1' or [hh6_work_e_q2] = '2' or [hh6_work2_e_q2] = '1')	Person 6: Does this person currently work in any of the following high-risk settings for COVID-19 transmission?	checkbox<br>1 hh6_work3_e_q2___1 healthcare setting (hospital, clinic, urgent care, etc.)<br>2 hh6_work3_e_q2___2 dense residential setting (nursing home, other long-term care facility)<br>3 hh6_work3_e_q2___3 prison or jail<br>4 hh6_work3_e_q2___4 meatpacking facility<br>5 hh6_work3_e_q2___5 shipping or distribution facility<br>6 hh6_work3_e_q2___6 high-volume retail facility (grocery store, etc.)<br>7 hh6_work3_e_q2___7 don't know<br><br>Field Annotation: @DEFAULT=" [hh6_work3_e_q2]"

1480	[hh6_work4_e_q2]<br><br>Show the field ONLY if:<br>[language_q2] = '1' and [hhcount_e_q2] > 5 and [hhcount_e_q2] < 13 and ([hh6_work_e_q2] = '1' or [hh6_work_e_q2] = '2' or [hh6_work2_e_q2] = '1')	Person 6: Does this person's employer offer them any of the following benefits at their current main job?<br>*Select all that apply.*	checkbox<br>1 hh6_work4_e_q2___1 paid sick leave<br>2 hh6_work4_e_q2___2 paid vacation/personal leave<br>3 hh6_work4_e_q2___3 health insurance<br>4 hh6_work4_e_q2___4 disability insurance<br>5 hh6_work4_e_q2___5 retirement plan<br>6 hh6_work4_e_q2___6 other<br>7 hh6_work4_e_q2___7 don't know<br><br>Field Annotation: @DEFAULT="[hh6_work4_e_q2]"
1481	[hh6_work5_e_q2]<br><br>Show the field ONLY if:<br>[language_q2] = '1' and [hhcount_e_q2] > 5 and [hhcount_e_q2] < 13 and ([hh6_work_e_q2] = '1' or [hh6_work_e_q2] = '2' or [hh6_work2_e_q2] = '1')	Person 6: On a scale of 0 (definitely not going to happen) to 10 (definitely going to happen), how likely is it that this person will lose their job because of the COVID-19 pandemic?	text (number, Min: 0, Max: 10)<br>Field Annotation: @DEFAULT="[hh6_work5_e_q2]"
1482	[hh6_work6_e_q2]<br><br>Show the field ONLY if:<br>[language_q2] = '1' and [hhcount_e_q2] > 5 and [hhcount_e_q2] < 13 and ([hh6_work_e_q2] = '1' or [hh6_work_e_q2] = '2' or [hh6_work2_e_q2] = '1')	Person 6: On a scale of 0 (definitely not going to happen) to 10 (definitely going to happen), how likely is it that this person will receive fewer work hours at their job because of the COVID-19 pandemic?	text (number, Min: 0, Max: 10)<br>Field Annotation: @DEFAULT="[hh6_work6_e_q2]"
1483	[hh6_work7_e_q2]<br><br>Show the field ONLY if:<br>[language_q2] = '1' and [hhcount_e_q2] > 5 and [hhcount_e_q2] < 13 and ([hh6_work_e_q2] = '1' or [hh6_work_e_q2] = '2' or [hh6_work2_e_q2] = '1')	Person 6: How often is this person required to work from outside of the home currently?	radio (Matrix)<br>1 always (100%)<br>2 most of the time (75%)<br>3 half of the time (50%)<br>4 less than half of the time (25%)<br>5 never (0%)<br>6 don't know
1484	[hh6_work8_e_q2]<br><br>Show the field ONLY if:<br>[language_q2] = '1' and ([hh6_work7_e_q2] = '1' or [hh6_work7_e_q2] = '2' or [hh6_work7_e_q2] = '3' or [hh6_work7_e_q2] = '4')	Person 6: How regularly is this person in close physical contact with co-workers during their work outside of the home currently?	radio (Matrix)<br>1 always (100%)<br>2 most of the time (75%)<br>3 half of the time (50%)<br>4 less than half of the time (25%)<br>5 never (0%)

			6 don't know
1485	[hh6_work9_e_q2]<br><br>Show the field ONLY if: [language_q2] = '1' and ([hh6_work7_e_q2] = '1' or [hh6_work7_e_q2] = '2' or [hh6_work7_e_q2] = '3' or [hh6_work7_e_q2] = '4')	Person 6: How regularly is this person in close physical contact with clients during their work outside of the home currently?	radio (Matrix)<br>1 always (100%)<br>2 most of the time (75%)<br>3 half of the time (50%)<br>4 less than half of the time (25%)<br>5 never (0%)<br>6 don't know
1486	[hh6_covidvaccine_e_q2]<br><br>Show the field ONLY if: [language_q2] = '1' and [hhcount_e_q2] > 5 and [hhcount_e_q2] < 13	Person 6: Does this person plan to get a vaccine for COVID-19?	radio<br>1 Yes<br>0 No<br>2 Don't know<br>3 This individual has already been vaccinated<br><br>Field Annotation: @DEFAULT="[hh6_covidvaccine_e_q2]"
1487	[hh6_covidsymp_e_q2]<br><br>Show the field ONLY if: [language_q2] = '1' and [hhcount_e_q2] > 5 and [hhcount_e_q2] < 13	Person 6: Has this person had any symptoms (cough, fever, difficulty breathing, fatigue, body aches, diarrhea, runny nose, loss of smell or taste) consistent with COVID-19 in the last two weeks?	radio<br>1 yes<br>0 no<br>2 don't know
1488	[hh6_covidsymp2_e_q2]<br><br>Show the field ONLY if: [language_q2] = '1' and [hh6_covidsymp_e_q2] = '1'	Person 6: When did this person's symptoms begin?	text (date_mdy)
1489	[hh6_covidsymp3_e_q2]<br><br>Show the field ONLY if: [language_q2] = '1' and [hh6_covidsymp_e_q2] = '1'	Person 6: Is this person worried that they may have had COVID-19 because of their symptoms?	radio<br>1 yes<br>0 no<br>2 don't know
1490	[hh6_covidsymp4_e_q2]<br><br>Show the field ONLY if: [language_q2] = '1' and [hh6_covidsymp_e_q2] = '1'	Person 6: Did this person experience any bias or discrimination because of their symptoms?	radio<br>1 yes<br>0 no<br>2 don't know
1491	[hh6_covidsymp5_e_q2]<br><br>Show the field ONLY if: [language_q2] = '1' and [hh6_covidsymp_e_q2]	Person 6: What did this person do in response to their symptoms?<br>*Select all that apply.*	checkbox<br>0 hh6_covidsymp5_e_q2___0 nothing<br>1 hh6_covidsymp5_e_q2___1 took over the counter medication

	= '1'		(ibuprofen, acetaminoph etc.)<br>2 | hh6_covidsymp5_e_q2___2 | communicat with a health care provide over the pho<br>3 | hh6_covidsymp5_e_q2___3 | visited a hea care provide office<br>4 | hh6_covidsymp5_e_q2___4 | visited a reta clinic or pharmacy<br>5 | hh6_covidsymp5_e_q2___5 | visited urger care (FASTM etc.)<br>6 | hh6_covidsymp5_e_q2___6 | visited the emergency room<br>7 | hh6_covidsymp5_e_q2___7 | was admitte the hospital<br>8 | hh6_covidsymp5_e_q2___8 | other<br>9 | hh6_covidsymp5_e_q2___9 | don't know
1492	[hh6_covidsymp6_e_q2]<br>Show the field ONLY if: [language_q2] = '1' and [hh6_covidsymp5_e_q2(8)] = '1'	Person 6: Please specify what other action this person took in response to their symptoms.	text
1493	[hh6_covidsymp7_e_q2]<br>Show the field ONLY if: [language_q2] = '1' and ([hh6_covidsymp5_e_q2(2)] = '1' or [hh6_covidsymp5_e_q2(3)] = '1' or [hh6_covidsymp5_e_q2(4)] = '1' or [hh6_covidsymp5_e_q2(5)] = '1' or [hh6_covidsymp5_e_q2(6)] = '1' or [hh6_covidsymp5_e_q2(7)] = '1' or [hh6_covidsymp5_e_q2(8)] = '1')	Person 6: Did a health care provider tell this person that they may have COVID-19?	radio<br>1 | yes<br>0 | no<br>2 | don't know
1494	[hh6_covid_test_e_q2]<br>Show the field ONLY if: [language_q2] = '1' and [hh6_covidsymp_e_q2] = '1'	Person 6: If this person received a COVID-19 test due to their symptoms, what was the result?	radio<br>1 | pending<br>2 | positive<br>3 | negative<br>4 | inconclusive

			5 did not receive a test<br>6 don't know
1495	[hh6_covid_admit_e_q2]<br><br>Show the field ONLY if: [language_q2] = '1' and [hh6_covidsymp5_e_q2(7)] = '1'	Person 6: How many days was this person admitted to the hospital?	text (number, Min: 0)
1496	[hh6_covid_admit2_e_q2]<br><br>Show the field ONLY if: [language_q2] = '1' and [hh6_covidsymp5_e_q2(7)] = '1'	Person 6: Did this person receive any of the following interventions during their hospital admission?	checkbox<br>1 hh6_covid_admit2_e_q2___1 extra oxygen in your nose<br>2 hh6_covid_admit2_e_q2___2 treatment in the intensive care unit (ICU)<br>3 hh6_covid_admit2_e_q2___3 mechanical ventilation (intubation or a breathing tube)<br>4 hh6_covid_admit2_e_q2___4 don't know
1497	[hh6_covidsymp8_e_q2]<br><br>Show the field ONLY if: [language_q2] = '1' and [hh6_covidsymp_e_q2] = '1'	Person 6: Has this person returned to their normal health at this time?	radio<br>1 yes<br>0 no<br>2 don't know
1498	[hh6_prevent_e_q2]<br><br>Show the field ONLY if: [language_q2] = '1' and [hh6_covidsymp_e_q2] = '1'	Person 6: Which of the following did this person do to protect their friends and family after their symptoms began?	checkbox<br>1 hh6_prevent_e_q2___1 wore a mask more frequently<br>2 hh6_prevent_e_q2___2 washed your hands with soap and water more frequently<br>3 hh6_prevent_e_q2___3 used hand sanitizer more frequently<br>4 hh6_prevent_e_q2___4 isolated yourself in your home more frequently<br>5 hh6_prevent_e_q2___5 stayed home more

			6 hh6_prevent_e_q2___6 wore disposable gloves more frequently<br>7 hh6_prevent_e_q2___7 don't know
1499	[hh7_relationship_e_q2]<br><br>Show the field ONLY if:<br>[language_q2] = '1' and [hhcount_e_q2] > 6 and [hhcount_e_q2] < 13	Section Header: *For each additional person in the your household, please provide the following information.*<br><br>Person 7: What is your relationship to this person?	radio<br>1 partner or spouse<br>2 child<br>3 parent<br>4 sibling<br>5 other family member<br>6 in-home childcare provider or other caregiver<br>7 other<br><br>Field Annotation: @DEFAULT="[hh7_relationship_e_q2]"
1500	[hh7_relationship2_e_q2]<br><br>Show the field ONLY if:<br>[language_q2] = '1' and [hh7_relationship_e_q2] = '7'	Person 7: Please specify your relationship with this person.	text<br>Field Annotation: @DEFAULT="[hh7_relationship2_e_q2]"
1501	[hh7_age_e_q2]<br><br>Show the field ONLY if:<br>[language_q2] = '1' and [hhcount_e_q2] > 6 and [hhcount_e_q2] < 13	Person 7: What is this person's age?<br>*Please specify their age in years*	text (number, Min: 0, Max: 110)<br>Field Annotation: @DEFAULT="[hh7_age_e_q2]"
1502	[hh7_sex_e_q2]<br><br>Show the field ONLY if:<br>[language_q2] = '1' and [hhcount_e_q2] > 6 and [hhcount_e_q2] < 13	Person 7: What is this person's sex?	radio<br>1 Female<br>2 Male<br>3 Other<br><br>Field Annotation: @DEFAULT="[hh7_sex_e_q2]"
1503	[hh7_race_e_q2]<br><br>Show the field ONLY if:<br>[language_q2] = '1' and [hhcount_e_q2] > 6 and [hhcount_e_q2] < 13	Person 7: What is this person's race?<br>*Select all that apply.*	checkbox<br>1 hh7_race_e_q2___1 American Indian or Alaska Native<br>2 hh7_race_e_q2___2 Asian<br>3 hh7_race_e_q2___3 Black or African American<br>4 hh7_race_e_q2___4 Native Hawaiian or Pacific Islander<br>5 hh7_race_e_q2___5 White

			6 hh7_race_e_q2___6 Other<br>7 hh7_race_e_q2___7 don't know<br><br>Field Annotation: @DEFAULT="[hh7_race_e_q2]"
1504	[hh7_e_q2thn_e_q2]<br><br>Show the field ONLY if:<br>[language_q2] = '1' and [hhcount_e_q2] > 6 and [hhcount_e_q2] < 13	Person 7: What is this person's ethnicity?	radio<br>1 Hispanic or Latino<br>2 Not Hispanic or Latino<br>3 Other<br>4 don't know<br><br>Field Annotation: @DEFAULT="[hh7_e_q2thn_e_q2]"
1505	[hh7_e_q2du_e_q2]<br><br>Show the field ONLY if:<br>[language_q2] = '1' and [hhcount_e_q2] > 6 and [hhcount_e_q2] < 13	Person 7: What is the highest level of education or schooling this person has completed?	radio<br>1 never attended school<br>2 kindergarten - 8th grade<br>3 some high school<br>4 high school equivalency (GED)<br>5 high school graduate<br>6 some college<br>7 college graduate<br>8 graduate school or more<br>9 don't know<br><br>Field Annotation: @DEFAULT="[hh7_e_q2du_e_q2]"
1506	[hh7_work_e_q2]<br><br>Show the field ONLY if:<br>[language_q2] = '1' and [hhcount_e_q2] > 6 and [hhcount_e_q2] < 13	Person 7: Which of the following best fit this person's current work situation?	radio<br>1 works full time<br>2 works part time<br>3 is looking for work/employment<br>4 retired<br>5 homemaker<br>6 student<br>7 on maternity/paternity leave<br>8 on illness/sick leave<br>9 on disability<br>10 other<br>11 don't know<br><br>Field Annotation: @DEFAULT="[hh7_work_e_q2]"
1507	[hh7_work2_e_q2]	Person 7: Does this person currently consider themselves self-employed (including as an	radio

	Show the field ONLY if: [language_q2] = '1' and [hhcount_e_q2] > 6 and [hhcount_e_q2] < 13	independent contractor or gig-economy worker)?	1 yes<br>0 no<br>2 don't know<br><br>Field Annotation: @DEFAULT=" [hh7_work2_e_q2]"
1508	[hh7_work3_e_q2]<br><br>Show the field ONLY if: [language_q2] = '1' and [hhcount_e_q2] > 6 and [hhcount_e_q2] < 13 an d ([hh7_work_e_q2] = '1' or [hh7_work_e_q2] = '2' or [hh7_work2_e_q 2] = '1')	Person 7: Does this person currently work in any of the following high-risk settings for COVID-19 transmission?	checkbox<br>1 hh7_work3_e_q2___1 healthcare setting (hospital, clinic, urgent care, etc.)<br>2 hh7_work3_e_q2___2 dense residential setting (nursing home, other long-term care facility)<br>3 hh7_work3_e_q2___3 prison or jail<br>4 hh7_work3_e_q2___4 meatpacking facility<br>5 hh7_work3_e_q2___5 shipping or distribution facility<br>6 hh7_work3_e_q2___6 high-volume retail facility (grocery store, etc.)<br>7 hh7_work3_e_q2___7 don't know<br><br>Field Annotation: @DEFAULT=" [hh7_work3_e_q2]"
1509	[hh7_work4_e_q2]<br><br>Show the field ONLY if: [language_q2] = '1' and [hhcount_e_q2] > 6 and [hhcount_e_q2] < 13 an d ([hh7_work_e_q2] = '1' or [hh7_work_e_q2] = '2' or [hh7_work2_e_q 2] = '1')	Person 7: Does this person's employer offer them any of the following benefits at their current main job?<br>*Select all that apply.*	checkbox<br>1 hh7_work4_e_q2___1 paid sick leave<br>2 hh7_work4_e_q2___2 paid vacation/personal leave<br>3 hh7_work4_e_q2___3 health insurance<br>4 hh7_work4_e_q2___4 disability insurance<br>5 hh7_work4_e_q2___5 retirement plan<br>6 hh7_work4_e_q2___6 other<br>7 hh7_work4_e_q2___7 don't know<br><br>Field Annotation: @DEFAULT=" [hh7_work4_e_q2]"

1510	[hh7_work5_e_q2]<br><br>Show the field ONLY if:<br>[language_q2] = '1' and [hhcount_e_q2] > 6 and [hhcount_e_q2] < 13 and ([hh7_work_e_q2] = '1' or [hh7_work_e_q2] = '2' or [hh7_work2_e_q2] = '1')	Person 7: On a scale of 0 (definitely not going to happen) to 10 (definitely going to happen), how likely is it that this person will lose their job because of the COVID-19 pandemic?	text (number, Min: 0, Max: 10)<br>Field Annotation: @DEFAULT="[hh7_work5_e_q2]"
1511	[hh7_work6_e_q2]<br><br>Show the field ONLY if:<br>[language_q2] = '1' and [hhcount_e_q2] > 6 and [hhcount_e_q2] < 13 and ([hh7_work_e_q2] = '1' or [hh7_work_e_q2] = '2' or [hh7_work2_e_q2] = '1')	Person 7: On a scale of 0 (definitely not going to happen) to 10 (definitely going to happen), how likely is it that this person will receive fewer work hours at their job because of the COVID-19 pandemic?	text (number, Min: 0, Max: 10)<br>Field Annotation: @DEFAULT="[hh7_work6_e_q2]"
1512	[hh7_work7_e_q2]<br><br>Show the field ONLY if:<br>[language_q2] = '1' and [hhcount_e_q2] > 6 and [hhcount_e_q2] < 13 and ([hh7_work_e_q2] = '1' or [hh7_work_e_q2] = '2' or [hh7_work2_e_q2] = '1')	Person 7: How often is this person required to work from outside of the home currently?	radio (Matrix)<br>1 always (100%)<br>2 most of the time (75%)<br>3 half of the time (50%)<br>4 less than half of the time (25%)<br>5 never (0%)<br>6 don't know
1513	[hh7_work8_e_q2]<br><br>Show the field ONLY if:<br>[language_q2] = '1' and ([hh7_work7_e_q2] = '1' or [hh7_work7_e_q2] = '2' or [hh7_work7_e_q2] = '3' or [hh7_work7_e_q2] = '4')	Person 7: How regularly is this person in close physical contact with co-workers during their work outside of the home currently?	radio (Matrix)<br>1 always (100%)<br>2 most of the time (75%)<br>3 half of the time (50%)<br>4 less than half of the time (25%)<br>5 never (0%)<br>6 don't know
1514	[hh7_work9_e_q2]<br><br>Show the field ONLY if:<br>[language_q2] = '1' and ([hh7_work7_e_q2] = '1' or [hh7_work7_e_q2] = '2' or [hh7_work7_e_q2] = '3' or [hh7_work7_e_q2] = '4')	Person 7: How regularly is this person in close physical contact with clients during their work outside of the home currently?	radio (Matrix)<br>1 always (100%)<br>2 most of the time (75%)<br>3 half of the time (50%)<br>4 less than half of the time (25%)<br>5 never (0%)<br>6 don't know

1515	[hh7_covidvaccine_e_q2]<br><br>Show the field ONLY if: [language_q2] = '1' and [hhcount_e_q2] > 6 and [hhcount_e_q2] < 13	Person 7: Does this person plan to get a vaccine for COVID-19?	radio<br>1 Yes<br>0 No<br>2 Don't know<br>3 This individual has already been vaccinated<br><br>Field Annotation: @DEFAULT="[hh7_covidvaccine_e_q2]"
1516	[hh7_covidsymp_e_q2]<br><br>Show the field ONLY if: [language_q2] = '1' and [hhcount_e_q2] > 6 and [hhcount_e_q2] < 13	Person 7: Has this person had any symptoms (cough, fever, difficulty breathing, fatigue, body aches, diarrhea, runny nose, loss of smell or taste) consistent with COVID-19 in the last two weeks?	radio<br>1 yes<br>0 no<br>2 don't know
1517	[hh7_covidsymp2_e_q2]<br><br>Show the field ONLY if: [language_q2] = '1' and [hh7_covidsymp_e_q2] = '1'	Person 7: When did this person's symptoms begin?	text (date_mdy)
1518	[hh7_covidsymp3_e_q2]<br><br>Show the field ONLY if: [language_q2] = '1' and [hh7_covidsymp_e_q2] = '1'	Person 7: Is this person worried that they may have had COVID-19 because of their symptoms?	radio<br>1 yes<br>0 no<br>2 don't know
1519	[hh7_covidsymp4_e_q2]<br><br>Show the field ONLY if: [language_q2] = '1' and [hh7_covidsymp_e_q2] = '1'	Person 7: Did this person experience any bias or discrimination because of their symptoms?	radio<br>1 yes<br>0 no<br>2 don't know
1520	[hh7_covidsymp5_e_q2]<br><br>Show the field ONLY if: [language_q2] = '1' and [hh7_covidsymp_e_q2] = '1'	Person 7: What did this person do in response to their symptoms?<br>*Select all that apply.*	checkbox<br>0 hh7_covidsymp5_e_q2___0 nothing<br>1 hh7_covidsymp5_e_q2___1 took over th[illegible] counter medication (ibuprofen, acetaminop[illegible] etc.)<br>2 hh7_covidsymp5_e_q2___2 communica[illegible] with a healt[illegible] care provid[illegible] over the ph[illegible]<br>3 hh7_covidsymp5_e_q2___3 visited a he[illegible] care provid[illegible] office

			4 hh7_covidsymp5_e_q2___4 visited a reta clinic or pharmacy<br>5 hh7_covidsymp5_e_q2___5 visited urger care (FASTM etc.)<br>6 hh7_covidsymp5_e_q2___6 visited the emergency room<br>7 hh7_covidsymp5_e_q2___7 was admitte the hospital<br>8 hh7_covidsymp5_e_q2___8 other<br>9 hh7_covidsymp5_e_q2___9 don't know
1521	[hh7_covidsymp6_e_q2]<br><br>Show the field ONLY if: [language_q2] = '1' and [hh7_covidsymp5_e_q2(8)] = '1'	Person 7: Please specify what other action this person took in response to their symptoms.	text
1522	[hh7_covidsymp7_e_q2]<br><br>Show the field ONLY if: [language_q2] = '1' and ([hh7_covidsymp5_e_q2(2)] = '1' or [hh7_covidsymp5_e_q2(3)] = '1' or [hh7_covidsymp5_e_q2(4)] = '1' or [hh7_covidsymp5_e_q2(5)] = '1' or [hh7_covidsymp5_e_q2(6)] = '1' or [hh7_covidsymp5_e_q2(7)] = '1' or [hh7_covidsymp5_e_q2(8)] = '1')	Person 7: Did a health care provider tell this person that they may have COVID-19?	radio<br>1 yes<br>0 no<br>2 don't know
1523	[hh7_covid_test_e_q2]<br><br>Show the field ONLY if: [language_q2] = '1' and [hh7_covidsymp_e_q2] = '1'	Person 7: If this person received a COVID-19 test due to their symptoms, what was the result?	radio<br>1 pending<br>2 positive<br>3 negative<br>4 inconclusive<br>5 did not receive a test<br>6 don't know
1524	[hh7_covid_admit_e_q2]<br><br>Show the field ONLY if: [language_q2] = '1' and [hh7_covidsymp5_e_q2(7)] = '1'	Person 7: How many days was this person admitted to the hospital?	text (number, Min: 0)

1525	[hh7_covid_admit2_e_q2]<br><br>Show the field ONLY if: [language_q2] = '1' and [hh7_covidsymp5_e_q2(7)] = '1'	Person 7: Did this person receive any of the following interventions during their hospital admission?	checkbox<br>1 hh7_covid_admit2_e_q2___1 extra oxygen in your nose<br>2 hh7_covid_admit2_e_q2___2 treatment in the intensive care unit (ICU)<br>3 hh7_covid_admit2_e_q2___3 mechanical ventilation (intubation or a breathing tube)<br>4 hh7_covid_admit2_e_q2___4 don't know
1526	[hh7_covidsymp8_e_q2]<br><br>Show the field ONLY if: [language_q2] = '1' and [hh7_covidsymp_e_q2] = '1'	Person 7: Has this person returned to their normal health at this time?	radio<br>1 yes<br>0 no<br>2 don't know
1527	[hh7_prevent_e_q2]<br><br>Show the field ONLY if: [language_q2] = '1' and [hh7_covidsymp_e_q2] = '1'	Person 7: Which of the following did this person do to protect their friends and family after their symptoms began?	checkbox<br>1 hh7_prevent_e_q2___1 wore a mask more frequently<br>2 hh7_prevent_e_q2___2 washed your hands with soap and water more frequently<br>3 hh7_prevent_e_q2___3 used hand sanitizer more frequently<br>4 hh7_prevent_e_q2___4 isolated yourself in your home more frequently<br>5 hh7_prevent_e_q2___5 stayed home more frequently<br>6 hh7_prevent_e_q2___6 wore disposable gloves more frequently<br>7 hh7_prevent_e_q2___7 don't know
1528	[hh8_relationship_e_q2]	Section Header: *For each additional person in the your household, please provide the following information.*	radio<br>1 partner or spouse

	Show the field ONLY if: [language_q2] = '1' and [hhcount_e_q2] > 7 and [hhcount_e_q2] < 13	Person 8: What is your relationship to this person?	2 child<br>3 parent<br>4 sibling<br>5 other family member<br>6 in-home childcare provider or other caregiver<br>7 other<br><br>Field Annotation: @DEFAULT="[hh8_relationship_e_q2]"
1529	[hh8_relationship2_e_q2]<br><br>Show the field ONLY if: [language_q2] = '1' and [hh8_relationship_e_q2] = '7'	Person 8: Please specify your relationship with this person.	text<br>Field Annotation: @DEFAULT="[hh8_relationship2_e_q2]"
1530	[hh8_age_e_q2]<br><br>Show the field ONLY if: [language_q2] = '1' and [hhcount_e_q2] > 7 and [hhcount_e_q2] < 13	Person 8: What is this person's age?<br>*Please specify their age in years*	text (number, Min: 0, Max: 110)<br>Field Annotation: @DEFAULT="[hh8_age_e_q2]"
1531	[hh8_sex_e_q2]<br><br>Show the field ONLY if: [language_q2] = '1' and [hhcount_e_q2] > 7 and [hhcount_e_q2] < 13	Person 8: What is this person's sex?	radio<br>1 Female<br>2 Male<br>3 Other<br><br>Field Annotation: @DEFAULT="[hh8_sex_e_q2]"
1532	[hh8_race_e_q2]<br><br>Show the field ONLY if: [language_q2] = '1' and [hhcount_e_q2] > 7 and [hhcount_e_q2] < 13	Person 8: What is this person's race?<br>*Select all that apply.*	checkbox<br>1 hh8_race_e_q2___1 American Indian or Alaska Native<br>2 hh8_race_e_q2___2 Asian<br>3 hh8_race_e_q2___3 Black or African American<br>4 hh8_race_e_q2___4 Native Hawaiian or Pacific Islander<br>5 hh8_race_e_q2___5 White<br>6 hh8_race_e_q2___6 Other<br>7 hh8_race_e_q2___7 don't know<br><br>Field Annotation: @DEFAULT="[hh8_race_e_q2]"
1533	[hh8_e_q2thn_e_q2]<br><br>Show the field ONLY if:	Person 8: What is this person's ethnicity?	radio<br>1 Hispanic or Latino

	[language_q2] = '1' and [hhcount_e_q2] > 7 and [hhcount_e_q2] < 13		2 Not Hispanic or Latino<br>3 Other<br>4 don't know<br><br>Field Annotation: @DEFAULT=" [hh8_e_q2thn_e_q2]"
1534	[hh8_e_q2du_e_q2]<br><br>Show the field ONLY if: [language_q2] = '1' and [hhcount_e_q2] > 7 and [hhcount_e_q2] < 13	Person 8: What is the highest level of education or schooling this person has completed?	radio<br>1 never attended school<br>2 kindergarten - 8th grade<br>3 some high school<br>4 high school equivalency (GED)<br>5 high school graduate<br>6 some college<br>7 college graduate<br>8 graduate school or more<br>9 don't know<br><br>Field Annotation: @DEFAULT=" [hh8_e_q2du_e_q2]"
1535	[hh8_work_e_q2]<br><br>Show the field ONLY if: [language_q2] = '1' and [hhcount_e_q2] > 7 and [hhcount_e_q2] < 13	Person 8: Which of the following best fit this person's current work situation?	radio<br>1 works full time<br>2 works part time<br>3 is looking for work/employment<br>4 retired<br>5 homemaker<br>6 student<br>7 on maternity/paternity leave<br>8 on illness/sick leave<br>9 on disability<br>10 other<br>11 don't know<br><br>Field Annotation: @DEFAULT=" [hh8_work_e_q2]"
1536	[hh8_work2_e_q2]<br><br>Show the field ONLY if: [language_q2] = '1' and [hhcount_e_q2] > 7 and [hhcount_e_q2] < 13	Person 8: Does this person currently consider themselves self-employed (including as an independent contractor or gig-economy worker)?	radio<br>1 yes<br>0 no<br>2 don't know<br><br>Field Annotation: @DEFAULT=" [hh8_work2_e_q2]"
1537	[hh8_work3_e_q2]	Person 8: Does this person currently work in any of the following high-risk settings for COVID-19	checkbox

	Show the field ONLY if: [language_q2] = '1' and [hhcount_e_q2] > 7 and [hhcount_e_q2] < 13 and ([hh8_work_e_q2] = '1' or [hh8_work_e_q2] = '2' or [hh8_work2_e_q2] = '1')	transmission?	1 hh8_work3_e_q2___1 healthcare setting (hospital, clinic, urgent care, etc.)<br>2 hh8_work3_e_q2___2 dense residential setting (nursing home, other long-term care facility)<br>3 hh8_work3_e_q2___3 prison or jail<br>4 hh8_work3_e_q2___4 meatpacking facility<br>5 hh8_work3_e_q2___5 shipping or distribution facility<br>6 hh8_work3_e_q2___6 high-volume retail facility (grocery store, etc.)<br>7 hh8_work3_e_q2___7 don't know<br><br>Field Annotation: @DEFAULT="[hh8_work3_e_q2]"
1538	[hh8_work4_e_q2]<br><br>Show the field ONLY if: [language_q2] = '1' and [hhcount_e_q2] > 7 and [hhcount_e_q2] < 13 and ([hh8_work_e_q2] = '1' or [hh8_work_e_q2] = '2' or [hh8_work2_e_q2] = '1')	Person 8: Does this person's employer offer them any of the following benefits at their current main job?<br>*Select all that apply.*	checkbox<br>1 hh8_work4_e_q2___1 paid sick leave<br>2 hh8_work4_e_q2___2 paid vacation/personal leave<br>3 hh8_work4_e_q2___3 health insurance<br>4 hh8_work4_e_q2___4 disability insurance<br>5 hh8_work4_e_q2___5 retirement plan<br>6 hh8_work4_e_q2___6 other<br>7 hh8_work4_e_q2___7 don't know<br><br>Field Annotation: @DEFAULT="[hh8_work4_e_q2]"
1539	[hh8_work5_e_q2]<br><br>Show the field ONLY if: [language_q2] = '1' and [hhcount_e_q2] > 7 and [hhcount_e_q2] < 13 and ([hh8_work_e_q2] = '1' or [hh8_work_e_q2] = '2' or [hh8_work2_e_q2] = '1')	Person 8: On a scale of 0 (definitely not going to happen) to 10 (definitely going to happen), how likely is it that this person will lose their job because of the COVID-19 pandemic?	text (number, Min: 0, Max: 10)<br>Field Annotation: @DEFAULT="[hh8_work5_e_q2]"

1540	[hh8_work6_e_q2]<br><br>Show the field ONLY if:<br>[language_q2] = '1' and [hhcount_e_q2] > 7 and [hhcount_e_q2] < 13 and ([hh8_work_e_q2] = '1' or [hh8_work_e_q2] = '2' or [hh8_work2_e_q2] = '1')	Person 8: On a scale of 0 (definitely not going to happen) to 10 (definitely going to happen), how likely is it that this person will receive fewer work hours at their job because of the COVID-19 pandemic?	text (number, Min: 0, Max: 10)<br>Field Annotation: @DEFAULT="[hh8_work6_e_q2]"
1541	[hh8_work7_e_q2]<br><br>Show the field ONLY if:<br>[language_q2] = '1' and [hhcount_e_q2] > 7 and [hhcount_e_q2] < 13 and ([hh8_work_e_q2] = '1' or [hh8_work_e_q2] = '2' or [hh8_work2_e_q2] = '1')	Person 8: How often is this person required to work from outside of the home currently?	radio (Matrix)<br>1 always (100%)<br>2 most of the time (75%)<br>3 half of the time (50%)<br>4 less than half of the time (25%)<br>5 never (0%)<br>6 don't know
1542	[hh8_work8_e_q2]<br><br>Show the field ONLY if:<br>[language_q2] = '1' and ([hh8_work7_e_q2] = '1' or [hh8_work7_e_q2] = '2' or [hh8_work7_e_q2] = '3' or [hh8_work7_e_q2] = '4')	Person 8: How regularly is this person in close physical contact with co-workers during their work outside of the home currently?	radio (Matrix)<br>1 always (100%)<br>2 most of the time (75%)<br>3 half of the time (50%)<br>4 less than half of the time (25%)<br>5 never (0%)<br>6 don't know
1543	[hh8_work9_e_q2]<br><br>Show the field ONLY if:<br>[language_q2] = '1' and ([hh8_work7_e_q2] = '1' or [hh8_work7_e_q2] = '2' or [hh8_work7_e_q2] = '3' or [hh8_work7_e_q2] = '4')	Person 8: How regularly is this person in close physical contact with clients during their work outside of the home currently?	radio (Matrix)<br>1 always (100%)<br>2 most of the time (75%)<br>3 half of the time (50%)<br>4 less than half of the time (25%)<br>5 never (0%)<br>6 don't know
1544	[hh8_covidvaccine_e_q2]<br><br>Show the field ONLY if:<br>[language_q2] = '1' and [hhcount_e_q2] > 7 and [hhcount_e_q2] < 13	Person 8: Does this person plan to get a vaccine for COVID-19?	radio<br>1 Yes<br>0 No<br>2 Don't know<br>3 This individual has already been vaccinated<br><br>Field Annotation: @DEFAULT="[hh8_covidvaccine_e_q2]"
1545	[hh8_covidsymp_e_q2]<br><br>Show the field ONLY if:	Person 8: Has this person had any symptoms (cough, fever, difficulty breathing, fatigue, body aches, diarrhea, runny nose, loss of smell or taste) consistent with COVID-19 in the last two weeks?	radio<br>1 yes<br>0 no

	[language_q2] = '1' and [hhcount_e_q2] > 7 and [hhcount_e_q2] < 13		2 don't know
1546	[hh8_covidsymp2_e_q2]<br>Show the field ONLY if: [language_q2] = '1' and [hh8_covidsymp_e_q2] = '1'	Person 8: When did this person's symptoms begin?	text (date_mdy)
1547	[hh8_covidsymp3_e_q2]<br>Show the field ONLY if: [language_q2] = '1' and [hh8_covidsymp_e_q2] = '1'	Person 8: Is this person worried that they may have had COVID-19 because of their symptoms?	radio<br>1 yes<br>0 no<br>2 don't know
1548	[hh8_covidsymp4_e_q2]<br>Show the field ONLY if: [language_q2] = '1' and [hh8_covidsymp_e_q2] = '1'	Person 8: Did this person experience any bias or discrimination because of their symptoms?	radio<br>1 yes<br>0 no<br>2 don't know
1549	[hh8_covidsymp5_e_q2]<br>Show the field ONLY if: [language_q2] = '1' and [hh8_covidsymp_e_q2] = '1'	Person 8: What did this person do in response to their symptoms?<br>*Select all that apply.*	checkbox<br>0 hh8_covidsymp5_e_q2___0 nothing<br>1 hh8_covidsymp5_e_q2___1 took over the counter medication (ibuprofen, acetaminoph[illegible] etc.)<br>2 hh8_covidsymp5_e_q2___2 communicat[illegible] with a health care provide[illegible] over the pho[illegible]<br>3 hh8_covidsymp5_e_q2___3 visited a hea[illegible] care provide[illegible] office<br>4 hh8_covidsymp5_e_q2___4 visited a reta[illegible] clinic or pharmacy<br>5 hh8_covidsymp5_e_q2___5 visited urge[illegible] care (FASTM[illegible] etc.)<br>6 hh8_covidsymp5_e_q2___6 visited the emergency room<br>7 hh8_covidsymp5_e_q2___7 was admitte[illegible] the hospital<br>8 hh8_covidsymp5_e_q2___8 other<br>9 hh8_covidsymp5_e_q2___9 don't know

1550	[hh8_covidsymp6_e_q2]<br><br>Show the field ONLY if: [language_q2] = '1' and [hh8_covidsymp5_e_q2(8)] = '1'	Person 8: Please specify what other action this person took in response to their symptoms.	text
1551	[hh8_covidsymp7_e_q2]<br><br>Show the field ONLY if: [language_q2] = '1' and ([hh8_covidsymp5_e_q2(2)] = '1' or [hh8_covidsymp5_e_q2(3)] = '1' or [hh8_covidsymp5_e_q2(4)] = '1' or [hh8_covidsymp5_e_q2(5)] = '1' or [hh8_covidsymp5_e_q2(6)] = '1' or [hh8_covidsymp5_e_q2(7)] = '1' or [hh8_covidsymp5_e_q2(8)] = '1')	Person 8: Did a health care provider tell this person that they may have COVID-19?	radio<br>1 yes<br>0 no<br>2 don't know
1552	[hh8_covid_test_e_q2]<br><br>Show the field ONLY if: [language_q2] = '1' and [hh8_covidsymp_e_q2] = '1'	Person 8: If this person received a COVID-19 test due to their symptoms, what was the result?	radio<br>1 pending<br>2 positive<br>3 negative<br>4 inconclusive<br>5 did not receive a test<br>6 don't know
1553	[hh8_covid_admit_e_q2]<br><br>Show the field ONLY if: [language_q2] = '1' and [hh8_covidsymp5_e_q2(7)] = '1'	Person 8: How many days was this person admitted to the hospital?	text (number, Min: 0)
1554	[hh8_covid_admit2_e_q2]<br><br>Show the field ONLY if: [language_q2] = '1' and [hh8_covidsymp5_e_q2(7)] = '1'	Person 8: Did this person receive any of the following interventions during their hospital admission?	checkbox<br>1 hh8_covid_admit2_e_q2___1 extra oxygen in your nose<br>2 hh8_covid_admit2_e_q2___2 treatment in the intensive care unit (ICU)<br>3 hh8_covid_admit2_e_q2___3 mechanical ventilation (intubation or a breathing tube)

			4 | hh8_covid_admit2_e_q2___4 | don't know
1555	[hh8_covidsymp8_e_q2]<br><br>Show the field ONLY if:<br>[language_q2] = '1' and<br>[hh8_covidsymp_e_q2]<br>= '1'	Person 8: Has this person returned to their normal health at this time?	radio<br>1 | yes<br>0 | no<br>2 | don't know
1556	[hh8_prevent_e_q2]<br><br>Show the field ONLY if:<br>[language_q2] = '1' and<br>[hh8_covidsymp_e_q2]<br>= '1'	Person 8: Which of the following did this person do to protect their friends and family after their symptoms began?	checkbox<br>1 | hh8_prevent_e_q2___1 | wore a mask more frequently<br>2 | hh8_prevent_e_q2___2 | washed your hands with soap and water more frequently<br>3 | hh8_prevent_e_q2___3 | used hand sanitizer more frequently<br>4 | hh8_prevent_e_q2___4 | isolated yourself in your home more frequently<br>5 | hh8_prevent_e_q2___5 | stayed home more frequently<br>6 | hh8_prevent_e_q2___6 | wore disposable gloves more frequently<br>7 | hh8_prevent_e_q2___7 | don't know
1557	[hh9_relationship_e_q2]<br><br>Show the field ONLY if:<br>[language_q2] = '1' and<br>[hhcount_e_q2] > 8 and<br>[hhcount_e_q2] < 13	Section Header: *For each additional person in the your household, please provide the following information.*<br><br>Person 9: What is your relationship to this person?	radio<br>1 | partner or spouse<br>2 | child<br>3 | parent<br>4 | sibling<br>5 | other family member<br>6 | in-home childcare provider or other caregiver<br>7 | other<br><br>Field Annotation: @DEFAULT="[hh9_relationship_e_q2]"
1558	[hh9_relationship2_e_q2]	Person 9: Please specify your relationship with this person.	text<br>Field Annotation: @DEFAULT="[hh9_relationship2_e_q2]"

	Show the field ONLY if: [language_q2] = '1' and [hh9_relationship_e_q2] = '7'		
1559	[hh9_age_e_q2]<br><br>Show the field ONLY if: [language_q2] = '1' and [hhcount_e_q2] > 8 and [hhcount_e_q2] < 13	Person 9: What is this person's age?<br>*Please specify their age in years*	text (number, Min: 0, Max: 110)<br>Field Annotation: @DEFAULT="[hh9_age_e_q2]"
1560	[hh9_sex_e_q2]<br><br>Show the field ONLY if: [language_q2] = '1' and [hhcount_e_q2] > 8 and [hhcount_e_q2] < 13	Person 9: What is this person's sex?	radio<br>1 Female<br>2 Male<br>3 Other<br><br>Field Annotation: @DEFAULT="[hh9_sex_e_q2]"
1561	[hh9_race_e_q2]<br><br>Show the field ONLY if: [language_q2] = '1' and [hhcount_e_q2] > 8 and [hhcount_e_q2] < 13	Person 9: What is this person's race?<br>*Select all that apply.*	checkbox<br>1 hh9_race_e_q2___1 American Indian or Alaska Native<br>2 hh9_race_e_q2___2 Asian<br>3 hh9_race_e_q2___3 Black or African American<br>4 hh9_race_e_q2___4 Native Hawaiian or Pacific Islander<br>5 hh9_race_e_q2___5 White<br>6 hh9_race_e_q2___6 Other<br>7 hh9_race_e_q2___7 don't know<br><br>Field Annotation: @DEFAULT="[hh9_race_e_q2]"
1562	[hh9_e_q2thn_e_q2]<br><br>Show the field ONLY if: [language_q2] = '1' and [hhcount_e_q2] > 8 and [hhcount_e_q2] < 13	Person 9: What is this person's ethnicity?	radio<br>1 Hispanic or Latino<br>2 Not Hispanic or Latino<br>3 Other<br>4 don't know<br><br>Field Annotation: @DEFAULT="[hh9_e_q2thn_e_q2]"
1563	[hh9_e_q2du_e_q2]<br><br>Show the field ONLY if: [language_q2] = '1' and [hhcount_e_q2] > 8 and [hhcount_e_q2] < 13	Person 9: What is the highest level of education or schooling this person has completed?	radio<br>1 never attended school<br>2 kindergarten - 8th grade<br>3 some high school<br>4 high school equivalency (GED)<br>5 high school graduate

			6 some college<br>7 college graduate<br>8 graduate school or more<br>9 don't know<br><br>Field Annotation: @DEFAULT="[hh9_e_q2du_e_q2]"
1564	[hh9_work_e_q2]<br><br>Show the field ONLY if: [language_q2] = '1' and [hhcount_e_q2] > 8 and [hhcount_e_q2] < 13	Person 9: Which of the following best fit this person's current work situation?	radio<br>1 works full time<br>2 works part time<br>3 is looking for work/employment<br>4 retired<br>5 homemaker<br>6 student<br>7 on maternity/paternity leave<br>8 on illness/sick leave<br>9 on disability<br>10 other<br>11 don't know<br><br>Field Annotation: @DEFAULT="[hh9_work_e_q2]"
1565	[hh9_work2_e_q2]<br><br>Show the field ONLY if: [language_q2] = '1' and [hhcount_e_q2] > 8 and [hhcount_e_q2] < 13	Person 9: Does this person currently consider themselves self-employed (including as an independent contractor or gig-economy worker)?	radio<br>1 yes<br>0 no<br>2 don't know<br><br>Field Annotation: @DEFAULT="[hh9_work2_e_q2]"
1566	[hh9_work3_e_q2]<br><br>Show the field ONLY if: [language_q2] = '1' and [hhcount_e_q2] > 8 and [hhcount_e_q2] < 13 and ([hh9_work_e_q2] = '1' or [hh9_work_e_q2] = '2' or [hh9_work2_e_q2] = '1')	Person 9: Does this person currently work in any of the following high-risk settings for COVID-19 transmission?	checkbox<br>1 hh9_work3_e_q2___1 healthcare setting (hospital, clinic, urgent care, etc.)<br>2 hh9_work3_e_q2___2 dense residential setting (nursing home, other long-term care facility)<br>3 hh9_work3_e_q2___3 prison or jail<br>4 hh9_work3_e_q2___4 meatpacking facility

			5 hh9_work3_e_q2___5 shipping or distribution facility<br>6 hh9_work3_e_q2___6 high-volume retail facility (grocery store, etc.)<br>7 hh9_work3_e_q2___7 don't know<br><br>Field Annotation: @DEFAULT="[hh9_work3_e_q2]"
1567	[hh9_work4_e_q2]<br><br>Show the field ONLY if: [language_q2] = '1' and [hhcount_e_q2] > 8 and [hhcount_e_q2] < 13 and ([hh9_work_e_q2] = '1' or [hh9_work_e_q2] = '2' or [hh9_work2_e_q2] = '1')	Person 9: Does this person's employer offer them any of the following benefits at their current main job?<br>*Select all that apply.*	checkbox<br>1 hh9_work4_e_q2___1 paid sick leave<br>2 hh9_work4_e_q2___2 paid vacation/personal leave<br>3 hh9_work4_e_q2___3 health insurance<br>4 hh9_work4_e_q2___4 disability insurance<br>5 hh9_work4_e_q2___5 retirement plan<br>6 hh9_work4_e_q2___6 other<br>7 hh9_work4_e_q2___7 don't know<br><br>Field Annotation: @DEFAULT="[hh9_work4_e_q2]"
1568	[hh9_work5_e_q2]<br><br>Show the field ONLY if: [language_q2] = '1' and [hhcount_e_q2] > 8 and [hhcount_e_q2] < 13 and ([hh9_work_e_q2] = '1' or [hh9_work_e_q2] = '2' or [hh9_work2_e_q2] = '1')	Person 9: On a scale of 0 (definitely not going to happen) to 10 (definitely going to happen), how likely is it that this person will lose their job because of the COVID-19 pandemic?	text (number, Min: 0, Max: 10)<br>Field Annotation: @DEFAULT="[hh9_work5_e_q2]"
1569	[hh9_work6_e_q2]<br><br>Show the field ONLY if: [language_q2] = '1' and [hhcount_e_q2] > 8 and [hhcount_e_q2] < 13 and ([hh9_work_e_q2] = '1' or [hh9_work_e_q2] = '2' or [hh9_work2_e_q2] = '1')	Person 9: On a scale of 0 (definitely not going to happen) to 10 (definitely going to happen), how likely is it that this person will receive fewer work hours at their job because of the COVID-19 pandemic?	text (number, Min: 0, Max: 10)<br>Field Annotation: @DEFAULT="[hh9_work6_e_q2]"
1570	[hh9_work7_e_q2]<br><br>Show the field ONLY if: [language_q2] = '1' and [hhcount_e_q2] > 8 and [hhcount_e_q2] < 13 and ([hh9_work_e_q2] =	Person 9: How often is this person required to work from outside of the home currently?	radio (Matrix)<br>1 always (100%)<br>2 most of the time (75%)<br>3 half of the time (50%)<br>4 less than half of the time (25%)

	'1' or [hh9_work_e_q2] = '2' or [hh9_work2_e_q2] = '1')		5 never (0%)<br>6 don't know
1571	[hh9_work8_e_q2]<br><br>Show the field ONLY if: [language_q2] = '1' and ([hh9_work7_e_q2] = '1' or [hh9_work7_e_q2] = '2' or [hh9_work7_e_q2] = '3' or [hh9_work7_e_q2] = '4')	Person 9: How regularly is this person in close physical contact with co-workers during their work outside of the home currently?	radio (Matrix)<br>1 always (100%)<br>2 most of the time (75%)<br>3 half of the time (50%)<br>4 less than half of the time (25%)<br>5 never (0%)<br>6 don't know
1572	[hh9_work9_e_q2]<br><br>Show the field ONLY if: [language_q2] = '1' and ([hh9_work7_e_q2] = '1' or [hh9_work7_e_q2] = '2' or [hh9_work7_e_q2] = '3' or [hh9_work7_e_q2] = '4')	Person 9: How regularly is this person in close physical contact with clients during their work outside of the home currently?	radio (Matrix)<br>1 always (100%)<br>2 most of the time (75%)<br>3 half of the time (50%)<br>4 less than half of the time (25%)<br>5 never (0%)<br>6 don't know
1573	[hh9_covidvaccine_e_q2]<br><br>Show the field ONLY if: [language_q2] = '1' and [hhcount_e_q2] > 8 and [hhcount_e_q2] < 13	Person 9: Does this person plan to get a vaccine for COVID-19?	radio<br>1 Yes<br>0 No<br>2 Don't know<br>3 This individual has already been vaccinated<br><br>Field Annotation: @DEFAULT="[hh9_covidvaccine_e_q2]"
1574	[hh9_covidsymp_e_q2]<br><br>Show the field ONLY if: [language_q2] = '1' and [hhcount_e_q2] > 8 and [hhcount_e_q2] < 13	Person 9: Has this person had any symptoms (cough, fever, difficulty breathing, fatigue, body aches, diarrhea, runny nose, loss of smell or taste) consistent with COVID-19 in the last two weeks?	radio<br>1 yes<br>0 no<br>2 don't know
1575	[hh9_covidsymp2_e_q2]<br><br>Show the field ONLY if: [language_q2] = '1' and [hh9_covidsymp_e_q2] = '1'	Person 9: When did this person's symptoms begin?	text (date_mdy)
1576	[hh9_covidsymp3_e_q2]<br><br>Show the field ONLY if: [language_q2] = '1' and [hh9_covidsymp_e_q2] = '1'	Person 9: Is this person worried that they may have had COVID-19 because of their symptoms?	radio<br>1 yes<br>0 no<br>2 don't know

1577	[hh9_covidsymp4_e_q2]<br><br>Show the field ONLY if: [language_q2] = '1' and [hh9_covidsymp_e_q2] = '1'	Person 9: Did this person experience any bias or discrimination because of their symptoms?	radio<br>1 yes<br>0 no<br>2 don't know
1578	[hh9_covidsymp5_e_q2]<br><br>Show the field ONLY if: [language_q2] = '1' and [hh9_covidsymp_e_q2] = '1'	Person 9: What did this person do in response to their symptoms?<br>*Select all that apply.*	checkbox<br>0 hh9_covidsymp5_e_q2___0 nothing<br>1 hh9_covidsymp5_e_q2___1 took over the counter medication (ibuprofen, acetaminoph[illegible] etc.)<br>2 hh9_covidsymp5_e_q2___2 communicat[illegible] with a health care provide[illegible] over the pho[illegible]<br>3 hh9_covidsymp5_e_q2___3 visited a hea[illegible] care provide[illegible] office<br>4 hh9_covidsymp5_e_q2___4 visited a reta[illegible] clinic or pharmacy<br>5 hh9_covidsymp5_e_q2___5 visited urgen[illegible] care (FASTM[illegible] etc.)<br>6 hh9_covidsymp5_e_q2___6 visited the emergency room<br>7 hh9_covidsymp5_e_q2___7 was admitte[illegible] the hospital<br>8 hh9_covidsymp5_e_q2___8 other<br>9 hh9_covidsymp5_e_q2___9 don't know
1579	[hh9_covidsymp6_e_q2]<br><br>Show the field ONLY if: [language_q2] = '1' and [hh9_covidsymp5_e_q2(8)] = '1'	Person 9: Please specify what other action this person took in response to their symptoms.	text
1580	[hh9_covidsymp7_e_q2]<br><br>Show the field ONLY if: [language_q2] = '1' and ([hh9_covidsymp5_e_q2(2)] = '1' or [hh9_covidsymp5_e_q2(3)] = '1' or [hh9_covidsymp5_e_q2(4)] = '1' or [hh9_covidsymp5_e_q2(5)] = '1' or	Person 9: Did a health care provider tell this person that they may have COVID-19?	radio<br>1 yes<br>0 no<br>2 don't know

	[hh9_covidsymp5_e_q2(6)] = '1' or [hh9_covidsymp5_e_q2(7)] = '1' or [hh9_covidsymp5_e_q2(8)] = '1')		
1581	[hh9_covid_test_e_q2]<br><br>Show the field ONLY if:<br>[language_q2] = '1' and [hh9_covidsymp_e_q2] = '1'	Person 9: If this person received a COVID-19 test due to their symptoms, what was the result?	radio<br>1 pending<br>2 positive<br>3 negative<br>4 inconclusive<br>5 did not receive a test<br>6 don't know
1582	[hh9_covid_admit_e_q2]<br><br>Show the field ONLY if:<br>[language_q2] = '1' and [hh9_covidsymp5_e_q2(7)] = '1'	Person 9: How many days was this person admitted to the hospital?	text (number, Min: 0)
1583	[hh9_covid_admit2_e_q2]<br><br>Show the field ONLY if:<br>[language_q2] = '1' and [hh9_covidsymp5_e_q2(7)] = '1'	Person 9: Did this person receive any of the following interventions during their hospital admission?	checkbox<br>1 hh9_covid_admit2_e_q2___1 extra oxygen in your nose<br>2 hh9_covid_admit2_e_q2___2 treatment in the intensive care unit (ICU)<br>3 hh9_covid_admit2_e_q2___3 mechanical ventilation (intubation or a breathing tube)<br>4 hh9_covid_admit2_e_q2___4 don't know
1584	[hh9_covidsymp8_e_q2]<br><br>Show the field ONLY if:<br>[language_q2] = '1' and [hh9_covidsymp_e_q2] = '1'	Person 9: Has this person returned to their normal health at this time?	radio<br>1 yes<br>0 no<br>2 don't know
1585	[hh9_prevent_e_q2]<br><br>Show the field ONLY if:<br>[language_q2] = '1' and [hh9_covidsymp_e_q2] = '1'	Person 9: Which of the following did this person do to protect their friends and family after their symptoms began?	checkbox<br>1 hh9_prevent_e_q2___1 wore a mask more frequently

			2 hh9_prevent_e_q2___2 washed your hands with soap and water more frequently<br>3 hh9_prevent_e_q2___3 used hand sanitizer more frequently<br>4 hh9_prevent_e_q2___4 isolated yourself in your home more frequently<br>5 hh9_prevent_e_q2___5 stayed home more frequently<br>6 hh9_prevent_e_q2___6 wore disposable gloves more frequently<br>7 hh9_prevent_e_q2___7 don't know
1586	[hh10_relationship_e_q2]<br><br>Show the field ONLY if: [language_q2] = '1' and [hhcount_e_q2] > 9 and [hhcount_e_q2] < 13	Section Header: *For each additional person in the your household, please provide the following information.*<br><br>Person 10: What is your relationship to this person?	radio<br>1 partner or spouse<br>2 child<br>3 parent<br>4 sibling<br>5 other family member<br>6 in-home childcare provider or other caregiver<br>7 other<br><br>Field Annotation: @DEFAULT="[hh10_relationship_e_q2]"
1587	[hh10_relationship2_e_q2]<br><br>Show the field ONLY if: [language_q2] = '1' and [hh10_relationship_e_q2] = '7'	Person 10: Please specify your relationship with this person.	text<br>Field Annotation: @DEFAULT="[hh10_relationship2_e_q2]"
1588	[hh10_age_e_q2]<br><br>Show the field ONLY if: [language_q2] = '1' and [hhcount_e_q2] > 9 and [hhcount_e_q2] < 13	Person 10: What is this person's age?<br>*Please specify their age in years*	text (number, Min: 0, Max: 110)<br>Field Annotation: @DEFAULT="[hh10_age_e_q2]"
1589	[hh10_sex_e_q2]<br><br>Show the field ONLY if:	Person 10: What is this person's sex?	radio<br>1 Female

	[language_q2] = '1' and [hhcount_e_q2] > 9 and [hhcount_e_q2] < 13		2 Male<br>3 Other<br><br>Field Annotation: @DEFAULT=" [hh10_sex_e_q2]"
1590	[hh10_race_e_q2]<br><br>Show the field ONLY if: [language_q2] = '1' and [hhcount_e_q2] > 9 and [hhcount_e_q2] < 13	Person 10: What is this person's race?<br>*Select all that apply.*	checkbox<br>1 hh10_race_e_q2___1 American Indian or Alaska Native<br>2 hh10_race_e_q2___2 Asian<br>3 hh10_race_e_q2___3 Black or African American<br>4 hh10_race_e_q2___4 Native Hawaiian or Pacific Islander<br>5 hh10_race_e_q2___5 White<br>6 hh10_race_e_q2___6 Other<br>7 hh10_race_e_q2___7 don't know<br><br>Field Annotation: @DEFAULT=" [hh10_race_e_q2]"
1591	[hh10_e_q2thn_e_q2]<br><br>Show the field ONLY if: [language_q2] = '1' and [hhcount_e_q2] > 9 and [hhcount_e_q2] < 13	Person 10: What is this person's ethnicity?	radio<br>1 Hispanic or Latino<br>2 Not Hispanic or Latino<br>3 Other<br>4 don't know<br><br>Field Annotation: @DEFAULT=" [hh10_e_q2thn_e_q2]"
1592	[hh10_e_q2du_e_q2]<br><br>Show the field ONLY if: [language_q2] = '1' and [hhcount_e_q2] > 9 and [hhcount_e_q2] < 13	Person 10: What is the highest level of education or schooling this person has completed?	radio<br>1 never attended school<br>2 kindergarten - 8th grade<br>3 some high school<br>4 high school equivalency (GED)<br>5 high school graduate<br>6 some college<br>7 college graduate<br>8 graduate school or more<br>9 don't know<br><br>Field Annotation: @DEFAULT=" [hh10_e_q2du_e_q2]"
1593	[hh10_work_e_q2]<br><br>Show the field ONLY if:	Person 10: Which of the following best fit this person's current work situation?	radio<br>1 works full time

	[language_q2] = '1' and [hhcount_e_q2] > 9 and [hhcount_e_q2] < 13		2 works part time<br>3 is looking for work/employment<br>4 retired<br>5 homemaker<br>6 student<br>7 on maternity/paternity leave<br>8 on illness/sick leave<br>9 on disability<br>10 other<br>11 don't know<br><br>Field Annotation: @DEFAULT=" [hh10_work_e_q2]"
1594	[hh10_work2_e_q2]<br><br>Show the field ONLY if: [language_q2] = '1' and [hhcount_e_q2] > 9 and [hhcount_e_q2] < 13	Person 10: Does this person currently consider themselves self-employed (including as an independent contractor or gig-economy worker)?	radio<br>1 yes<br>0 no<br>2 don't know<br><br>Field Annotation: @DEFAULT=" [hh10_work2_e_q2]"
1595	[hh10_work3_e_q2]<br><br>Show the field ONLY if: [language_q2] = '1' and [hhcount_e_q2] > 9 and [hhcount_e_q2] < 13 and ([hh10_work_e_q2] = '1' or [hh10_work_e_q2] = '2' or [hh10_work2_e_q2] = '1')	Person 10: Does this person currently work in any of the following high-risk settings for COVID-19 transmission?	checkbox<br>1 hh10_work3_e_q2___1 healthcare setting (hospital, clinic, urgent care, etc.)<br>2 hh10_work3_e_q2___2 dense residential setting (nursing home, other long-term care facility)<br>3 hh10_work3_e_q2___3 prison or jail<br>4 hh10_work3_e_q2___4 meatpacking facility<br>5 hh10_work3_e_q2___5 shipping or distribution facility<br>6 hh10_work3_e_q2___6 high-volume retail facility (grocery store, etc.)<br>7 hh10_work3_e_q2___7 don't know<br><br>Field Annotation: @DEFAULT=" [hh10_work3_e_q2]"

1596	[hh10_work4_e_q2]<br><br>Show the field ONLY if:<br>[language_q2] = '1' and [hhcount_e_q2] > 9 and [hhcount_e_q2] < 13 and ([hh10_work_e_q2] = '1' or [hh10_work_e_q2] = '2' or [hh10_work2_e_q2] = '1')	Person 10: Does this person's employer offer them any of the following benefits at their current main job?<br>*Select all that apply.*	checkbox<br>1 hh10_work4_e_q2___1 paid sick leave<br>2 hh10_work4_e_q2___2 paid vacation/persona[illegible] leave<br>3 hh10_work4_e_q2___3 health insurance<br>4 hh10_work4_e_q2___4 disability insurance<br>5 hh10_work4_e_q2___5 retirement plan<br>6 hh10_work4_e_q2___6 other<br>7 hh10_work4_e_q2___7 don't know<br><br>Field Annotation: @DEFAULT="[hh10_work4_e_q2]"
1597	[hh10_work5_e_q2]<br><br>Show the field ONLY if:<br>[language_q2] = '1' and [hhcount_e_q2] > 9 and [hhcount_e_q2] < 13 and ([hh10_work_e_q2] = '1' or [hh10_work_e_q2] = '2' or [hh10_work2_e_q2] = '1')	Person 10: On a scale of 0 (definitely not going to happen) to 10 (definitely going to happen), how likely is it that this person will lose their job because of the COVID-19 pandemic?	text (number, Min: 0, Max: 10)<br>Field Annotation: @DEFAULT="[hh10_work5_e_q2]"
1598	[hh10_work6_e_q2]<br><br>Show the field ONLY if:<br>[language_q2] = '1' and [hhcount_e_q2] > 9 and [hhcount_e_q2] < 13 and ([hh10_work_e_q2] = '1' or [hh10_work_e_q2] = '2' or [hh10_work2_e_q2] = '1')	Person 10: On a scale of 0 (definitely not going to happen) to 10 (definitely going to happen), how likely is it that this person will receive fewer work hours at their job because of the COVID-19 pandemic?	text (number, Min: 0, Max: 10)<br>Field Annotation: @DEFAULT="[hh10_work6_e_q2]"
1599	[hh10_work7_e_q2]<br><br>Show the field ONLY if:<br>[language_q2] = '1' and [hhcount_e_q2] > 9 and [hhcount_e_q2] < 13 and ([hh10_work_e_q2] = '1' or [hh10_work_e_q2] = '2' or [hh10_work2_e_q2] = '1')	Person 10: How often is this person required to work from outside of the home currently?	radio (Matrix)<br>1 always (100%)<br>2 most of the time (75%)<br>3 half of the time (50%)<br>4 less than half of the time (25%)<br>5 never (0%)<br>6 don't know
1600	[hh10_work8_e_q2]<br><br>Show the field ONLY if:<br>[language_q2] = '1' and ([hh10_work7_e_q2] = '1' or [hh10_work7_e_q2] = '2' or [hh10_work7_e_q2] = '3' or [hh10_work7_e_q2] = '4')	Person 10: How regularly is this person in close physical contact with co-workers during their work outside of the home currently?	radio (Matrix)<br>1 always (100%)<br>2 most of the time (75%)<br>3 half of the time (50%)<br>4 less than half of the time (25%)<br>5 never (0%)

			6 don't know
1601	[hh10_work9_e_q2]<br><br>Show the field ONLY if: [language_q2] = '1' and ([hh10_work7_e_q2] = '1' or [hh10_work7_e_q2] = '2' or [hh10_work7_e_q2] = '3' or [hh10_work7_e_q2] = '4')	Person 10: How regularly is this person in close physical contact with clients during their work outside of the home currently?	radio (Matrix)<br>1 always (100%)<br>2 most of the time (75%)<br>3 half of the time (50%)<br>4 less than half of the time (25%)<br>5 never (0%)<br>6 don't know
1602	[hh10_covidvaccine_e_q2]<br><br>Show the field ONLY if: [language_q2] = '1' and [hhcount_e_q2] > 9 and [hhcount_e_q2] < 13	Person 10: Does this person plan to get a vaccine for COVID-19?	radio<br>1 Yes<br>0 No<br>2 Don't know<br>3 This individual has already been vaccinated<br><br>Field Annotation: @DEFAULT="[hh10_covidvaccine_e_q2]"
1603	[hh10_covidsymp_e_q2]<br><br>Show the field ONLY if: [language_q2] = '1' and [hhcount_e_q2] > 9 and [hhcount_e_q2] < 13	Person 10: Has this person had any symptoms (cough, fever, difficulty breathing, fatigue, body aches, diarrhea, runny nose, loss of smell or taste) consistent with COVID-19 in the last two weeks?	radio<br>1 yes<br>0 no<br>2 don't know
1604	[hh10_covidsymp2_e_q2]<br><br>Show the field ONLY if: [language_q2] = '1' and [hh10_covidsymp_e_q2] = '1'	Person 10: When did this person's symptoms begin?	text (date_mdy)
1605	[hh10_covidsymp3_e_q2]<br><br>Show the field ONLY if: [language_q2] = '1' and [hh10_covidsymp_e_q2] = '1'	Person 10: Is this person worried that they may have had COVID-19 because of their symptoms?	radio<br>1 yes<br>0 no<br>2 don't know
1606	[hh10_covidsymp4_e_q2]<br><br>Show the field ONLY if: [language_q2] = '1' and [hh10_covidsymp_e_q2] = '1'	Person 10: Did this person experience any bias or discrimination because of their symptoms?	radio<br>1 yes<br>0 no<br>2 don't know
1607	[hh10_covidsymp5_e_q2]<br><br>Show the field ONLY if: [language_q2] = '1' and [hh10_covidsymp_e_q	Person 10: What did this person do in response to their symptoms?<br>*Select all that apply.*	checkbox<br>0 hh10_covidsymp5_e_q2___0 nothing<br>1 hh10_covidsymp5_e_q2___1 took over th counter medicatio

	2] = '1'		(ibuprofen, acetaminop etc.)<br>2 hh10_covidsymp5_e_q2___2 communica with a healt care provid over the ph<br>3 hh10_covidsymp5_e_q2___3 visited a he care provid office<br>4 hh10_covidsymp5_e_q2___4 visited a ret clinic or pharmacy<br>5 hh10_covidsymp5_e_q2___5 visited urge care (FASTM etc.)<br>6 hh10_covidsymp5_e_q2___6 visited the emergency room<br>7 hh10_covidsymp5_e_q2___7 was admitt the hospita<br>8 hh10_covidsymp5_e_q2___8 other<br>9 hh10_covidsymp5_e_q2___9 don't know
1608	[hh10_covidsymp6_e_q2]<br>Show the field ONLY if: [language_q2] = '1' and [hh10_covidsymp5_e_q2(8)] = '1'	Person 10: Please specify what other action this person took in response to their symptoms.	text
1609	[hh10_covidsymp7_e_q2]<br>Show the field ONLY if: [language_q2] = '1' and ([hh10_covidsymp5_e_q2(2)] = '1' or [hh10_covidsymp5_e_q2(3)] = '1' or [hh10_covidsymp5_e_q2(4)] = '1' or [hh10_covidsymp5_e_q2(5)] = '1' or [hh10_covidsymp5_e_q2(6)] = '1' or [hh10_covidsymp5_e_q2(7)] = '1' or [hh10_covidsymp5_e_q2(8)] = '1')	Person 10: Did a health care provider tell this person that they may have COVID-19?	radio<br>1 yes<br>0 no<br>2 don't know
1610	[hh10_covid_test_e_q2]<br>Show the field ONLY if: [language_q2] = '1' and [hh10_covidsymp_e_q2] = '1'	Person 10: If this person received a COVID-19 test due to their symptoms, what was the result?	radio<br>1 pending<br>2 positive<br>3 negative<br>4 inconclusive

			5 did not receive a test<br>6 don't know
1611	[hh10_covid_admit_e_q2]<br><br>Show the field ONLY if:<br>[language_q2] = '1' and [hh10_covidsymp5_e_q2(7)] = '1'	Person 10: How many days was this person admitted to the hospital?	text (number, Min: 0)
1612	[hh10_covid_admit2_e_q2]<br><br>Show the field ONLY if:<br>[language_q2] = '1' and [hh10_covidsymp5_e_q2(7)] = '1'	Person 10: Did this person receive any of the following interventions during their hospital admission?	checkbox<br>1 hh10_covid_admit2_e_q2___1 extra oxygen in your nose<br>2 hh10_covid_admit2_e_q2___2 treatment in the intensive care unit (ICU)<br>3 hh10_covid_admit2_e_q2___3 mechanica[illegible] ventilatio[illegible] (intubatio[illegible] or a breathing tube)<br>4 hh10_covid_admit2_e_q2___4 don't kno[illegible]
1613	[hh10_covidsymp8_e_q2]<br><br>Show the field ONLY if:<br>[language_q2] = '1' and [hh10_covidsymp_e_q2] = '1'	Person 10: Has this person returned to their normal health at this time?	radio<br>1 yes<br>0 no<br>2 don't know
1614	[hh10_prevent_e_q2]<br><br>Show the field ONLY if:<br>[language_q2] = '1' and [hh10_covidsymp_e_q2] = '1'	Person 10: Which of the following did this person do to protect their friends and family after their symptoms began?	checkbox<br>1 hh10_prevent_e_q2___1 wore a mask more frequently<br>2 hh10_prevent_e_q2___2 washed your hands with soap and water more frequently<br>3 hh10_prevent_e_q2___3 used hand sanitizer more frequently<br>4 hh10_prevent_e_q2___4 isolated yourself in your home more frequently

			5 | hh10_prevent_e_q2___5 | stayed home more frequently<br>6 | hh10_prevent_e_q2___6 | wore disposable gloves more frequently<br>7 | hh10_prevent_e_q2___7 | don't know
1615	[hh11_relationship_e_q2]<br><br>Show the field ONLY if:<br>[language_q2] = '1' and [hhcount_e_q2] > 10 and [hhcount_e_q2] < 13	Section Header: *For each additional person in the your household, please provide the following information.*<br><br>Person 11: What is your relationship to this person?	radio<br>1 | partner or spouse<br>2 | child<br>3 | parent<br>4 | sibling<br>5 | other family member<br>6 | in-home childcare provider or other caregiver<br>7 | other<br><br>Field Annotation: @DEFAULT="[hh11_relationship_e_q2]"
1616	[hh11_relationship2_e_q2]<br><br>Show the field ONLY if:<br>[language_q2] = '1' and [hh11_relationship_e_q2] = '7'	Person 11: Please specify your relationship with this person.	text<br>Field Annotation: @DEFAULT="[hh11_relationship2_e_q2]"
1617	[hh11_age_e_q2]<br><br>Show the field ONLY if:<br>[language_q2] = '1' and [hhcount_e_q2] > 10 and [hhcount_e_q2] < 13	Person 11: What is this person's age?<br>*Please specify their age in years*	text (number, Min: 0, Max: 110)<br>Field Annotation: @DEFAULT="[hh11_age_e_q2]"
1618	[hh11_sex_e_q2]<br><br>Show the field ONLY if:<br>[language_q2] = '1' and [hhcount_e_q2] > 10 and [hhcount_e_q2] < 13	Person 11: What is this person's sex?	radio<br>1 | Female<br>2 | Male<br>3 | Other<br><br>Field Annotation: @DEFAULT="[hh11_sex_e_q2]"
1619	[hh11_race_e_q2]<br><br>Show the field ONLY if:<br>[language_q2] = '1' and [hhcount_e_q2] > 10 and [hhcount_e_q2] < 13	Person 11: What is this person's race?<br>*Select all that apply.*	checkbox<br>1 | hh11_race_e_q2___1 | American Indian or Alaska Native<br>2 | hh11_race_e_q2___2 | Asian<br>3 | hh11_race_e_q2___3 | Black or African American

			4 hh11_race_e_q2___4 Native Hawaiian or Pacific Islander<br>5 hh11_race_e_q2___5 White<br>6 hh11_race_e_q2___6 Other<br>7 hh11_race_e_q2___7 don't know<br><br>Field Annotation: @DEFAULT=" [hh11_race_e_q2]"
1620	[hh11_e_q2thn_e_q2]<br><br>Show the field ONLY if: [language_q2] = '1' and [hhcount_e_q2] > 10 and [hhcount_e_q2] < 13	Person 11: What is this person's ethnicity?	radio<br>1 Hispanic or Latino<br>2 Not Hispanic or Latino<br>3 Other<br>4 don't know<br><br>Field Annotation: @DEFAULT=" [hh11_e_q2thn_e_q2]"
1621	[hh11_e_q2du_e_q2]<br><br>Show the field ONLY if: [language_q2] = '1' and [hhcount_e_q2] > 10 and [hhcount_e_q2] < 13	Person 11: What is the highest level of education or schooling this person has completed?	radio<br>1 never attended school<br>2 kindergarten - 8th grade<br>3 some high school<br>4 high school equivalency (GED)<br>5 high school graduate<br>6 some college<br>7 college graduate<br>8 graduate school or more<br>9 don't know<br><br>Field Annotation: @DEFAULT=" [hh11_e_q2du_e_q2]"
1622	[hh11_work_e_q2]<br><br>Show the field ONLY if: [language_q2] = '1' and [hhcount_e_q2] > 10 and [hhcount_e_q2] < 13	Person 11: Which of the following best fit this person's current work situation?	radio<br>1 works full time<br>2 works part time<br>3 is looking for work/employment<br>4 retired<br>5 homemaker<br>6 student<br>7 on maternity/paternity leave<br>8 on illness/sick leave<br>9 on disability<br>10 other<br>11 don't know

			Field Annotation: @DEFAULT="[hh11_work_e_q2]"
1623	[hh11_work2_e_q2]<br>Show the field ONLY if: [language_q2] = '1' and [hhcount_e_q2] > 10 and [hhcount_e_q2] < 13	Person 11: Does this person currently consider themselves self-employed (including as an independent contractor or gig-economy worker)?	radio<br>1 yes<br>0 no<br>2 don't know<br>Field Annotation: @DEFAULT="[hh11_work2_e_q2]"
1624	[hh11_work3_e_q2]<br>Show the field ONLY if: [language_q2] = '1' and [hhcount_e_q2] > 10 and [hhcount_e_q2] < 13 and ([hh11_work_e_q2] = '1' or [hh11_work_e_q2] = '2' or [hh11_work2_e_q2] = '1')	Person 11: Does this person currently work in any of the following high-risk settings for COVID-19 transmission?	checkbox<br>1 hh11_work3_e_q2___1 healthcare setting (hospital, clinic, urgent care, etc.)<br>2 hh11_work3_e_q2___2 dense residential setting (nursing home, other long-term care facility)<br>3 hh11_work3_e_q2___3 prison or jail<br>4 hh11_work3_e_q2___4 meatpacking facility<br>5 hh11_work3_e_q2___5 shipping or distribution facility<br>6 hh11_work3_e_q2___6 high-volume retail facility (grocery store, etc.)<br>7 hh11_work3_e_q2___7 don't know<br>Field Annotation: @DEFAULT="[hh11_work3_e_q2]"
1625	[hh11_work4_e_q2]<br>Show the field ONLY if: [language_q2] = '1' and [hhcount_e_q2] > 10 and [hhcount_e_q2] < 13 and ([hh11_work_e_q2] = '1' or [hh11_work_e_q2] = '2' or [hh11_work2_e_q2] = '1')	Person 11: Does this person's employer offer them any of the following benefits at their current main job?<br>*Select all that apply.*	checkbox<br>1 hh11_work4_e_q2___1 paid sick leave<br>2 hh11_work4_e_q2___2 paid vacation/persona leave<br>3 hh11_work4_e_q2___3 health insurance<br>4 hh11_work4_e_q2___4 disability insurance<br>5 hh11_work4_e_q2___5 retirement plan<br>6 hh11_work4_e_q2___6 other<br>7 hh11_work4_e_q2___7 don't know

			Field Annotation: @DEFAULT="[hh11_work4_e_q2]"
1626	[hh11_work5_e_q2]<br>Show the field ONLY if: [language_q2] = '1' and [hhcount_e_q2] > 10 and [hhcount_e_q2] < 13 and ([hh11_work_e_q2] = '1' or [hh11_work_e_q2] = '2' or [hh11_work2_e_q2] = '1')	Person 11: On a scale of 0 (definitely not going to happen) to 10 (definitely going to happen), how likely is it that this person will lose their job because of the COVID-19 pandemic?	text (number, Min: 0, Max: 10)<br>Field Annotation: @DEFAULT="[hh11_work5_e_q2]"
1627	[hh11_work6_e_q2]<br>Show the field ONLY if: [language_q2] = '1' and [hhcount_e_q2] > 10 and [hhcount_e_q2] < 13 and ([hh11_work_e_q2] = '1' or [hh11_work_e_q2] = '2' or [hh11_work2_e_q2] = '1')	Person 11: On a scale of 0 (definitely not going to happen) to 10 (definitely going to happen), how likely is it that this person will receive fewer work hours at their job because of the COVID-19 pandemic?	text (number, Min: 0, Max: 10)<br>Field Annotation: @DEFAULT="[hh11_work6_e_q2]"
1628	[hh11_work7_e_q2]<br>Show the field ONLY if: [language_q2] = '1' and [hhcount_e_q2] > 10 and [hhcount_e_q2] < 13 and ([hh11_work_e_q2] = '1' or [hh11_work_e_q2] = '2' or [hh11_work2_e_q2] = '1')	Person 11: How often is this person required to work from outside of the home currently?	radio (Matrix)<br>1 always (100%)<br>2 most of the time (75%)<br>3 half of the time (50%)<br>4 less than half of the time (25%)<br>5 never (0%)<br>6 don't know
1629	[hh11_work8_e_q2]<br>Show the field ONLY if: [language_q2] = '1' and ([hh11_work7_e_q2] = '1' or [hh11_work7_e_q2] = '2' or [hh11_work7_e_q2] = '3' or [hh11_work7_e_q2] = '4')	Person 11: How regularly is this person in close physical contact with co-workers during their work outside of the home currently?	radio (Matrix)<br>1 always (100%)<br>2 most of the time (75%)<br>3 half of the time (50%)<br>4 less than half of the time (25%)<br>5 never (0%)<br>6 don't know
1630	[hh11_work9_e_q2]<br>Show the field ONLY if: [language_q2] = '1' and ([hh11_work7_e_q2] = '1' or [hh11_work7_e_q2] = '2' or [hh11_work7_e_q2] = '3' or [hh11_work7_e_q2] = '4')	Person 11: How regularly is this person in close physical contact with clients during their work outside of the home currently?	radio (Matrix)<br>1 always (100%)<br>2 most of the time (75%)<br>3 half of the time (50%)<br>4 less than half of the time (25%)<br>5 never (0%)<br>6 don't know
1631	[hh11_covidvaccine_e_q2]	Person 11: Does this person plan to get a vaccine for COVID-19?	radio<br>1 Yes

	Show the field ONLY if: [language_q2] = '1' and [hhcount_e_q2] > 10 and [hhcount_e_q2] < 13		0 No<br>2 Don't know<br>3 This individual has already been vaccinated<br><br>Field Annotation: @DEFAULT="[hh11_covidvaccine_e_q2]"
1632	[hh11_covidsymp_e_q2]<br><br>Show the field ONLY if: [language_q2] = '1' and [hhcount_e_q2] > 10 and [hhcount_e_q2] < 13	Person 11: Has this person had any symptoms (cough, fever, difficulty breathing, fatigue, body aches, diarrhea, runny nose, loss of smell or taste) consistent with COVID-19 in the last two weeks?	radio<br>1 yes<br>0 no<br>2 don't know
1633	[hh11_covidsymp2_e_q2]<br><br>Show the field ONLY if: [language_q2] = '1' and [hh11_covidsymp_e_q2] = '1'	Person 11: When did this person's symptoms begin?	text (date_mdy)
1634	[hh11_covidsymp3_e_q2]<br><br>Show the field ONLY if: [language_q2] = '1' and [hh11_covidsymp_e_q2] = '1'	Person 11: Is this person worried that they may have had COVID-19 because of their symptoms?	radio<br>1 yes<br>0 no<br>2 don't know
1635	[hh11_covidsymp4_e_q2]<br><br>Show the field ONLY if: [language_q2] = '1' and [hh11_covidsymp_e_q2] = '1'	Person 11: Did this person experience any bias or discrimination because of their symptoms?	radio<br>1 yes<br>0 no<br>2 don't know
1636	[hh11_covidsymp5_e_q2]<br><br>Show the field ONLY if: [language_q2] = '1' and [hh11_covidsymp_e_q2] = '1'	Person 11: What did this person do in response to their symptoms?<br>*Select all that apply.*	checkbox<br>0 hh11_covidsymp5_e_q2___0 nothing<br>1 hh11_covidsymp5_e_q2___1 took over th[illegible] counter medication (ibuprofen, acetaminop[illegible] etc.)<br>2 hh11_covidsymp5_e_q2___2 communica[illegible] with a healt[illegible] care provid[illegible] over the ph[illegible]<br>3 hh11_covidsymp5_e_q2___3 visited a he[illegible] care provid[illegible] office<br>4 hh11_covidsymp5_e_q2___4 visited a ret[illegible] clinic or pharmacy

			5 hh11_covidsymp5_e_q2___5 visited urge care (FASTM etc.)<br>6 hh11_covidsymp5_e_q2___6 visited the emergency room<br>7 hh11_covidsymp5_e_q2___7 was admitt the hospita<br>8 hh11_covidsymp5_e_q2___8 other<br>9 hh11_covidsymp5_e_q2___9 don't know
1637	[hh11_covidsymp6_e_q2]<br>Show the field ONLY if: [language_q2] = '1' and [hh11_covidsymp5_e_q2(8)] = '1'	Person 11: Please specify what other action this person took in response to their symptoms.	text
1638	[hh11_covidsymp7_e_q2]<br>Show the field ONLY if: [language_q2] = '1' and ([hh11_covidsymp5_e_q2(2)] = '1' or [hh11_covidsymp5_e_q2(3)] = '1' or [hh11_covidsymp5_e_q2(4)] = '1' or [hh11_covidsymp5_e_q2(5)] = '1' or [hh11_covidsymp5_e_q2(6)] = '1' or [hh11_covidsymp5_e_q2(7)] = '1' or [hh11_covidsymp5_e_q2(8)] = '1')	Person 11: Did a health care provider tell this person that they may have COVID-19?	radio<br>1 yes<br>0 no<br>2 don't know
1639	[hh11_covid_test_e_q2]<br>Show the field ONLY if: [language_q2] = '1' and [hh11_covidsymp_e_q2] = '1'	Person 11: If this person received a COVID-19 test due to their symptoms, what was the result?	radio<br>1 pending<br>2 positive<br>3 negative<br>4 inconclusive<br>5 did not receive a test<br>6 don't know
1640	[hh11_covid_admit_e_q2]<br>Show the field ONLY if: [language_q2] = '1' and [hh11_covidsymp5_e_q2(7)] = '1'	Person 11: How many days was this person admitted to the hospital?	text (number, Min: 0)
1641	[hh11_covid_admit2_e_q2]<br>Show the field ONLY if:	Person 11: Did this person receive any of the following interventions during their hospital admission?	checkbox<br>1 hh11_covid_admit2_e_q2___1 extra oxygen in your nose

	[language_q2] = '1' and [hh11_covidsymp5_e_q2(7)] = '1'		2 hh11_covid_admit2_e_q2___2 treatment in the intensive care unit (ICU)<br>3 hh11_covid_admit2_e_q2___3 mechanica ventilation (intubation or a breathing tube)<br>4 hh11_covid_admit2_e_q2___4 don't know
1642	[hh11_covidsymp8_e_q2]<br>Show the field ONLY if: [language_q2] = '1' and [hh11_covidsymp_e_q2] = '1'	Person 11: Has this person returned to their normal health at this time?	radio<br>1 yes<br>0 no<br>2 don't know
1643	[hh11_prevent_e_q2]<br>Show the field ONLY if: [language_q2] = '1' and [hh11_covidsymp_e_q2] = '1'	Person 11: Which of the following did this person do to protect their friends and family after their symptoms began?	checkbox<br>1 hh11_prevent_e_q2___1 wore a mask more frequently<br>2 hh11_prevent_e_q2___2 washed your hands with soap and water more frequently<br>3 hh11_prevent_e_q2___3 used hand sanitizer more frequently<br>4 hh11_prevent_e_q2___4 isolated yourself in your home more frequently<br>5 hh11_prevent_e_q2___5 stayed home more frequently<br>6 hh11_prevent_e_q2___6 wore disposable gloves more frequently<br>7 hh11_prevent_e_q2___7 don't know
1644	[hh12_relationship_e_q2]<br>Show the field ONLY if: [language_q2] = '1' and [hhcount_e_q2] > 11 an	Section Header: *For each additional person in the your household, please provide the following information.*<br>Person 12: What is your relationship to this person?	radio<br>1 partner or spouse<br>2 child<br>3 parent

	d [hhcount_e_q2] < 13		4 sibling<br>5 other family member<br>6 in-home childcare provider or other caregiver<br>7 other<br><br>Field Annotation: @DEFAULT="[hh12_relationship_e_q2]"
1645	[hh12_relationship2_e_q2]<br><br>Show the field ONLY if: [language_q2] = '1' and [hh12_relationship_e_q2] = '7'	Person 12: Please specify your relationship with this person.	text<br>Field Annotation: @DEFAULT="[hh12_relationship2_e_q2]"
1646	[hh12_age_e_q2]<br><br>Show the field ONLY if: [language_q2] = '1' and [hhcount_e_q2] > 11 and [hhcount_e_q2] < 13	Person 12: What is this person's age?<br>*Please specify their age in years*	text (number, Min: 0, Max: 110)<br>Field Annotation: @DEFAULT="[hh12_age_e_q2]"
1647	[hh12_sex_e_q2]<br><br>Show the field ONLY if: [language_q2] = '1' and [hhcount_e_q2] > 11 and [hhcount_e_q2] < 13	Person 12: What is this person's sex?	radio<br>1 Female<br>2 Male<br>3 Other<br><br>Field Annotation: @DEFAULT="[hh12_sex_e_q2]"
1648	[hh12_race_e_q2]<br><br>Show the field ONLY if: [language_q2] = '1' and [hhcount_e_q2] > 11 and [hhcount_e_q2] < 13	Person 12: What is this person's race?<br>*Select all that apply.*	checkbox<br>1 hh12_race_e_q2___1 American Indian or Alaska Native<br>2 hh12_race_e_q2___2 Asian<br>3 hh12_race_e_q2___3 Black or African American<br>4 hh12_race_e_q2___4 Native Hawaiian or Pacific Islander<br>5 hh12_race_e_q2___5 White<br>6 hh12_race_e_q2___6 Other<br>7 hh12_race_e_q2___7 don't know<br><br>Field Annotation: @DEFAULT="[hh12_race_e_q2]"
1649	[hh12_e_q2thn_e_q2]<br><br>Show the field ONLY if: [language_q2] = '1' and [hhcount_e_q2] > 11 and [hhcount_e_q2] < 13	Person 12: What is this person's ethnicity?	radio<br>1 Hispanic or Latino<br>2 Not Hispanic or Latino<br>3 Other

			4 don't know<br><br>Field Annotation: @DEFAULT="[hh12_e_q2thn_e_q2]"
1650	[hh12_e_q2du_e_q2]<br><br>Show the field ONLY if: [language_q2] = '1' and [hhcount_e_q2] > 11 and [hhcount_e_q2] < 13	Person 12: What is the highest level of education or schooling this person has completed?	radio<br>1 never attended school<br>2 kindergarten - 8th grade<br>3 some high school<br>4 high school equivalency (GED)<br>5 high school graduate<br>6 some college<br>7 college graduate<br>8 graduate school or more<br>9 don't know<br><br>Field Annotation: @DEFAULT="[hh12_e_q2du_e_q2]"
1651	[hh12_work_e_q2]<br><br>Show the field ONLY if: [language_q2] = '1' and [hhcount_e_q2] > 11 and [hhcount_e_q2] < 13	Person 12: Which of the following best fit this person's current work situation?	radio<br>1 works full time<br>2 works part time<br>3 is looking for work/employment<br>4 retired<br>5 homemaker<br>6 student<br>7 on maternity/paternity leave<br>8 on illness/sick leave<br>9 on disability<br>10 other<br>11 don't know<br><br>Field Annotation: @DEFAULT="[hh12_work_e_q2]"
1652	[hh12_work2_e_q2]<br><br>Show the field ONLY if: [language_q2] = '1' and [hhcount_e_q2] > 11 and [hhcount_e_q2] < 13	Person 12: Does this person currently consider themselves self-employed (including as an independent contractor or gig-economy worker)?	radio<br>1 yes<br>0 no<br>2 don't know<br><br>Field Annotation: @DEFAULT="[hh12_work2_e_q2]"
1653	[hh12_work3_e_q2]<br><br>Show the field ONLY if: [language_q2] = '1' and [hhcount_e_q2] > 11 an	Person 12: Does this person currently work in any of the following high-risk settings for COVID-19 transmission?	checkbox<br>1 hh12_work3_e_q2___1 healthcare setting (hospital,

	d [hhcount_e_q2] < 13 and ([hh12_work_e_q2] = '1' or [hh12_work_e_q2] = '2' or [hh12_work2_e_q2] = '1')		clinic, urgent care, etc.)<br>2 hh12_work3_e_q2___2 dense residential setting (nursing home, other long-term care facility)<br>3 hh12_work3_e_q2___3 prison or jail<br>4 hh12_work3_e_q2___4 meatpacking facility<br>5 hh12_work3_e_q2___5 shipping or distribution facility<br>6 hh12_work3_e_q2___6 high-volume retail facility (grocery store, etc.)<br>7 hh12_work3_e_q2___7 don't know<br><br>Field Annotation: @DEFAULT=[q2_2_arm_1][hh12_work3_e_q2:value] @DEFAULT="[hh12_work3_e_q2]"
1654	[hh12_work4_e_q2]<br><br>Show the field ONLY if: [language_q2] = '1' and [hhcount_e_q2] > 11 and [hhcount_e_q2] < 13 and ([hh12_work_e_q2] = '1' or [hh12_work_e_q2] = '2' or [hh12_work2_e_q2] = '1')	Person 12: Does this person's employer offer them any of the following benefits at their current main job?<br>*Select all that apply.*	checkbox<br>1 hh12_work4_e_q2___1 paid sick leave<br>2 hh12_work4_e_q2___2 paid vacation/personal leave<br>3 hh12_work4_e_q2___3 health insurance<br>4 hh12_work4_e_q2___4 disability insurance<br>5 hh12_work4_e_q2___5 retirement plan<br>6 hh12_work4_e_q2___6 other<br>7 hh12_work4_e_q2___7 don't know<br><br>Field Annotation: @DEFAULT="[hh12_work4_e_q2]"
1655	[hh12_work5_e_q2]<br><br>Show the field ONLY if: [language_q2] = '1' and [hhcount_e_q2] > 11 and [hhcount_e_q2] < 13 and ([hh12_work_e_q2] = '1' or [hh12_work_e_q2] = '2' or [hh12_work2_e_q2] = '1')	Person 12: On a scale of 0 (definitely not going to happen) to 10 (definitely going to happen), how likely is it that this person will lose their job because of the COVID-19 pandemic?	text (number, Min: 0, Max: 10)<br>Field Annotation: @DEFAULT="[hh12_work5_e_q2]"
1656	[hh12_work6_e_q2]<br><br>Show the field ONLY if:	Person 12: On a scale of 0 (definitely not going to happen) to 10 (definitely going to happen), how	text (number, Min: 0, Max: 10)<br>Field Annotation: @DEFAULT="

	[language_q2] = '1' and [hhcount_e_q2] > 11 and [hhcount_e_q2] < 13 and ([hh12_work_e_q2] = '1' or [hh12_work_e_q2] = '2' or [hh12_work2_e_q2] = '1')	likely is it that this person will receive fewer work hours at their job because of the COVID-19 pandemic?	[hh12_work6_e_q2]"
1657	[hh12_work7_e_q2]<br><br>Show the field ONLY if: [language_q2] = '1' and [hhcount_e_q2] > 11 and [hhcount_e_q2] < 13 and ([hh12_work_e_q2] = '1' or [hh12_work_e_q2] = '2' or [hh12_work2_e_q2] = '1')	Person 12: How often is this person required to work from outside of the home currently?	radio (Matrix)<br>1 always (100%)<br>2 most of the time (75%)<br>3 half of the time (50%)<br>4 less than half of the time (25%)<br>5 never (0%)<br>6 don't know
1658	[hh12_work8_e_q2]<br><br>Show the field ONLY if: [language_q2] = '1' and ([hh12_work7_e_q2] = '1' or [hh12_work7_e_q2] = '2' or [hh12_work7_e_q2] = '3' or [hh12_work7_e_q2] = '4')	Person 12: How regularly is this person in close physical contact with co-workers during their work outside of the home currently?	radio (Matrix)<br>1 always (100%)<br>2 most of the time (75%)<br>3 half of the time (50%)<br>4 less than half of the time (25%)<br>5 never (0%)<br>6 don't know
1659	[hh12_work9_e_q2]<br><br>Show the field ONLY if: [language_q2] = '1' and ([hh12_work7_e_q2] = '1' or [hh12_work7_e_q2] = '2' or [hh12_work7_e_q2] = '3' or [hh12_work7_e_q2] = '4')	Person 12: How regularly is this person in close physical contact with clients during their work outside of the home currently?	radio (Matrix)<br>1 always (100%)<br>2 most of the time (75%)<br>3 half of the time (50%)<br>4 less than half of the time (25%)<br>5 never (0%)<br>6 don't know
1660	[hh12_covidvaccine_e_q2]<br><br>Show the field ONLY if: [language_q2] = '1' and [hhcount_e_q2] > 11 and [hhcount_e_q2] < 13	Person 12: Does this person plan to get a vaccine for COVID-19?	radio<br>1 Yes<br>0 No<br>2 Don't know<br>3 This individual has already been vaccinated<br><br>Field Annotation: @DEFAULT="[hh12_covidvaccine_e_q2]"
1661	[hh12_covidsymp_e_q2]<br><br>Show the field ONLY if: [language_q2] = '1' and [hhcount_e_q2] > 11 and [hhcount_e_q2] < 13	Person 12: Has this person had any symptoms (cough, fever, difficulty breathing, fatigue, body aches, diarrhea, runny nose, loss of smell or taste) consistent with COVID-19 in the last two weeks?	radio<br>1 yes<br>0 no<br>2 don't know

1662	[hh12_covidsymp2_e_q2]<br><br>Show the field ONLY if: [language_q2] = '1' and [hh12_covidsymp_e_q2] = '1'	Person 12: When did this person's symptoms begin?	text (date_mdy)
1663	[hh12_covidsymp3_e_q2]<br><br>Show the field ONLY if: [language_q2] = '1' and [hh12_covidsymp_e_q2] = '1'	Person 12: Is this person worried that they may have had COVID-19 because of their symptoms?	radio<br>1 yes<br>0 no<br>2 don't know
1664	[hh12_covidsymp4_e_q2]<br><br>Show the field ONLY if: [language_q2] = '1' and [hh12_covidsymp_e_q2] = '1'	Person 12: Did this person experience any bias or discrimination because of their symptoms?	radio<br>1 yes<br>0 no<br>2 don't know
1665	[hh12_covidsymp5_e_q2]<br><br>Show the field ONLY if: [language_q2] = '1' and [hh12_covidsymp_e_q2] = '1'	Person 12: What did this person do in response to their symptoms?<br>*Select all that apply.*	checkbox<br>0 hh12_covidsymp5_e_q2___0 nothing<br>1 hh12_covidsymp5_e_q2___1 took over th counter medication (ibuprofen, acetaminop etc.)<br>2 hh12_covidsymp5_e_q2___2 communica with a healt care provid over the ph<br>3 hh12_covidsymp5_e_q2___3 visited a he care provid office<br>4 hh12_covidsymp5_e_q2___4 visited a ret clinic or pharmacy<br>5 hh12_covidsymp5_e_q2___5 visited urge care (FASTM etc.)<br>6 hh12_covidsymp5_e_q2___6 visited the emergency room<br>7 hh12_covidsymp5_e_q2___7 was admitt the hospita<br>8 hh12_covidsymp5_e_q2___8 other<br>9 hh12_covidsymp5_e_q2___9 don't know
1666	[hh12_covidsymp6_e_q2]<br><br>Show the field ONLY if:	Person 12: Please specify what other action this person took in response to their symptoms.	text

	[language_q2] = '1' and [hh12_covidsymp5_e_q2(8)] = '1'		
1667	[hh12_covidsymp7_e_q2]<br><br>Show the field ONLY if: [language_q2] = '1' and ([hh12_covidsymp5_e_q2(2)] = '1' or [hh12_covidsymp5_e_q2(3)] = '1' or [hh12_covidsymp5_e_q2(4)] = '1' or [hh12_covidsymp5_e_q2(5)] = '1' or [hh12_covidsymp5_e_q2(6)] = '1' or [hh12_covidsymp5_e_q2(7)] = '1' or [hh12_covidsymp5_e_q2(8)] = '1')	Person 12: Did a health care provider tell this person that they may have COVID-19?	radio<br>1 | yes<br>0 | no<br>2 | don't know
1668	[hh12_covid_test_e_q2]<br><br>Show the field ONLY if: [language_q2] = '1' and [hh12_covidsymp_e_q2] = '1'	Person 12: If this person received a COVID-19 test due to their symptoms, what was the result?	radio<br>1 | pending<br>2 | positive<br>3 | negative<br>4 | inconclusive<br>5 | did not receive a test<br>6 | don't know
1669	[hh12_covid_admit_e_q2]<br><br>Show the field ONLY if: [language_q2] = '1' and [hh12_covidsymp5_e_q2(7)] = '1'	Person 12: How many days was this person admitted to the hospital?	text (number, Min: 0)
1670	[hh12_covid_admit2_e_q2]<br><br>Show the field ONLY if: [language_q2] = '1' and [hh12_covidsymp5_e_q2(7)] = '1'	Person 12: Did this person receive any of the following interventions during their hospital admission?	checkbox<br>1 | hh12_covid_admit2_e_q2___1 | extra oxygen i[illegible] your nose<br>2 | hh12_covid_admit2_e_q2___2 | treatment in the intensive care unit (ICU)<br>3 | hh12_covid_admit2_e_q2___3 | mechanic[illegible] ventilatio[illegible] (intubatio[illegible] or a breathing tube)<br>4 | hh12_covid_admit2_e_q2___4 | don't kno[illegible]
1671	[hh12_covidsymp8_e_q2]	Person 12: Has this person returned to their normal health at this time?	radio<br>1 | yes

	Show the field ONLY if: [language_q2] = '1' and [hh12_covidsymp_e_q2] = '1'		0 no<br>2 don't know
1672	[hh12_prevent_e_q2]<br><br>Show the field ONLY if: [language_q2] = '1' and [hh12_covidsymp_e_q2] = '1'	Person 12: Which of the following did this person do to protect their friends and family after their symptoms began?	checkbox<br>1 hh12_prevent_e_q2___1 wore a mask more frequently<br>2 hh12_prevent_e_q2___2 washed your hands with soap and water more frequently<br>3 hh12_prevent_e_q2___3 used hand sanitizer more frequently<br>4 hh12_prevent_e_q2___4 isolated yourself in your home more frequently<br>5 hh12_prevent_e_q2___5 stayed home more frequently<br>6 hh12_prevent_e_q2___6 wore disposable gloves more frequently<br>7 hh12_prevent_e_q2___7 don't know
1673	[serious1_e_q2]<br><br>Show the field ONLY if: [language_q2] = '1'	Section Header: *Please provide the following information about your mental health and wellness.*<br><br>How serious a problem would you say the COVID-19 pandemic is for you personally right now?	radio<br>1 very serious<br>2 somewhat serious<br>3 not too serious<br>4 not at all serious
1674	[serious2_e_q2]<br><br>Show the field ONLY if: [language_q2] = '1'	How serious a problem would you say the COVID-19 pandemic is for people in your community right now?	radio<br>1 very serious<br>2 somewhat serious<br>3 not too serious<br>4 not at all serious
1675	[serious3_e_q2]<br><br>Show the field ONLY if: [language_q2] = '1'	How serious a problem would you say the COVID-19 pandemic is for people in the United States right now?	radio<br>1 very serious<br>2 somewhat serious<br>3 not too serious<br>4 not at all serious

1676	[serious4_e_q2]<br><br>Show the field ONLY if:<br>[language_q2] = '1'	How serious a problem would you say the COVID-19 pandemic is for people around the world right now?	radio<br>1 very serious<br>2 somewhat serious<br>3 not too serious<br>4 not at all serious
1677	[racism1_e_q2]<br><br>Show the field ONLY if:<br>[language_q2] = '1'	In the last two weeks, how often have you encountered news media, a viral/trending event, online videos, online discussions, or photos in which someone like you was threatened or harmed?	radio<br>1 Never<br>2 About once a month<br>3 About once a week<br>4 About once a day<br>5 Several times a day
1678	[racism2_e_q2]<br><br>Show the field ONLY if:<br>[language_q2] = '1'	In the last two weeks, how often have you encountered news media, a viral/trending event, online videos, online discussions, or photos in which negative stereotypes of people like you were promoted as if they were true?	radio<br>1 Never<br>2 About once a month<br>3 About once a week<br>4 About once a day<br>5 Several times a day
1679	[concern1_e_q2]<br><br>Show the field ONLY if:<br>[language_q2] = '1'	Section Header: *The COVID-19 pandemic may cause challenges for some people regardless of whether they are infected. How concerned are you about each of the following things?*<br><br>Getting the healthcare you need (including care for mental health)	radio (Matrix)<br>1 not at all concerned<br>2 not too concerned<br>3 somewhat concerned<br>4 very concerned
1680	[concern2_e_q2]<br><br>Show the field ONLY if:<br>[language_q2] = '1'	Having a place to live	radio (Matrix)<br>1 not at all concerned<br>2 not too concerned<br>3 somewhat concerned<br>4 very concerned
1681	[concern3_e_q2]<br><br>Show the field ONLY if:<br>[language_q2] = '1'	Being able to interact with other people	radio (Matrix)<br>1 not at all concerned<br>2 not too concerned<br>3 somewhat concerned<br>4 very concerned
1682	[concern4_e_q2]<br><br>Show the field ONLY if:<br>[language_q2] = '1'	Getting food, water, and other household supplies	radio (Matrix)<br>1 not at all concerned<br>2 not too concerned<br>3 somewhat concerned<br>4 very concerned
1683	[concern5_e_q2]	Getting medication	radio (Matrix)

	Show the field ONLY if: [language_q2] = '1'		1 not at all concerned<br>2 not too concerned<br>3 somewhat concerned<br>4 very concerned
1684	[concern6_e_q2]<br><br>Show the field ONLY if: [language_q2] = '1'	Having transportation to get where you need to go	radio (Matrix)<br>1 not at all concerned<br>2 not too concerned<br>3 somewhat concerned<br>4 very concerned
1685	[concern7_e_q2]<br><br>Show the field ONLY if: [language_q2] = '1'	Caring for your family and friends	radio (Matrix)<br>1 not at all concerned<br>2 not too concerned<br>3 somewhat concerned<br>4 very concerned
1686	[gad7_1_e_q2]<br><br>Show the field ONLY if: [language_q2] = '1'	Section Header: *During the last two weeks, how often have you been bothered by the following problems?*<br><br>Feeling nervous, anxious, or on edge	radio (Matrix)<br>0 not at all<br>1 several days<br>2 more than half of the days<br>3 nearly every day
1687	[gad7_2_e_q2]<br><br>Show the field ONLY if: [language_q2] = '1'	Not being able to stop or control worrying	radio (Matrix)<br>0 not at all<br>1 several days<br>2 more than half of the days<br>3 nearly every day
1688	[gad7_3_e_q2]<br><br>Show the field ONLY if: [language_q2] = '1'	Worrying too much about different things	radio (Matrix)<br>0 not at all<br>1 several days<br>2 more than half of the days<br>3 nearly every day
1689	[gad7_4_e_q2]<br><br>Show the field ONLY if: [language_q2] = '1'	Trouble relaxing	radio (Matrix)<br>0 not at all<br>1 several days<br>2 more than half of the days<br>3 nearly every day
1690	[gad7_5_e_q2]<br><br>Show the field ONLY if: [language_q2] = '1'	Being so restless that it's hard to sit still	radio (Matrix)<br>0 not at all<br>1 several days<br>2 more than half of the days

			3 nearly every day
1691	[gad7_6_e_q2]<br>Show the field ONLY if:<br>[language_q2] = '1'	Becoming easily annoyed or irritable	radio (Matrix)<br>0 not at all<br>1 several days<br>2 more than half of the days<br>3 nearly every day
1692	[gad7_7_e_q2]<br>Show the field ONLY if:<br>[language_q2] = '1'	Feeling afraid as if something awful might happen	radio (Matrix)<br>0 not at all<br>1 several days<br>2 more than half of the days<br>3 nearly every day
1693	[gad7_8_e_q2]<br>Show the field ONLY if:<br>[language_q2] = '1' and ([gad7_1_e_q2] > 0 or [gad7_2_e_q2] > 0 or [gad7_3_e_q2] > 0 or [gad7_4_e_q2] > 0 or [gad7_5_e_q2] > 0 or [gad7_6_e_q2] > 0 or [gad7_7_e_q2] > 0)	How difficult have these problems made it for you to do your work, take care of things at home, or get along with other people?	radio<br>0 not difficult<br>1 somewhat difficult<br>2 very difficult<br>3 extremely difficult
1694	[cesd_1_e_q2]<br>Show the field ONLY if:<br>[language_q2] = '1'	Section Header: *In the last two weeks, how often have you experienced the following feelings and behaviors?*<br>I was bothered by things that usually don't bother me.	radio (Matrix)<br>0 Rarely or none of the time (< 1 day)<br>1 Some or a little of the time (1-2 days)<br>2 Occasionally or a moderate amount of the time (3-4 days)<br>3 Most or all of the time (5-7 days)
1695	[cesd_2_e_q2]<br>Show the field ONLY if:<br>[language_q2] = '1'	I did not feel like eating; my appetite was poor.	radio (Matrix)<br>0 Rarely or none of the time (< 1 day)<br>1 Some or a little of the time (1-2 days)<br>2 Occasionally or a moderate amount of the time (3-4 days)<br>3 Most or all of the time (5-7 days)
1696	[cesd_3_e_q2]<br>Show the field ONLY if:<br>[language_q2] = '1'	I felt that I could not shake off the blues even with the help from my family and friends.	radio (Matrix)<br>0 Rarely or none of the time (< 1 day)<br>1 Some or a little of the time (1-2 days)<br>2 Occasionally or a moderate amount of the time (3-4 days)<br>3 Most or all of the time (5-7 days)
1697	[cesd_4_e_q2]<br>Show the field ONLY if:<br>[language_q2] = '1'	I felt that I was not as good as other people.	radio (Matrix)<br>0 Rarely or none of the time (< 1 day)<br>1 Some or a little of the time (1-2 days)

			2 Occasionally or a moderate amount of the time (3-4 days)<br>3 Most or all of the time (5-7 days)
1698	[cesd_5_e_q2]<br>Show the field ONLY if:<br>[language_q2] = '1'	I had trouble keeping my mind on what I was doing.	radio (Matrix)<br>0 Rarely or none of the time (< 1 day)<br>1 Some or a little of the time (1-2 days)<br>2 Occasionally or a moderate amount of the time (3-4 days)<br>3 Most or all of the time (5-7 days)
1699	[cesd_6_e_q2]<br>Show the field ONLY if:<br>[language_q2] = '1'	I felt depressed.	radio (Matrix)<br>0 Rarely or none of the time (< 1 day)<br>1 Some or a little of the time (1-2 days)<br>2 Occasionally or a moderate amount of the time (3-4 days)<br>3 Most or all of the time (5-7 days)
1700	[cesd_7_e_q2]<br>Show the field ONLY if:<br>[language_q2] = '1'	I felt that everything I did was an effort.	radio (Matrix)<br>0 Rarely or none of the time (< 1 day)<br>1 Some or a little of the time (1-2 days)<br>2 Occasionally or a moderate amount of the time (3-4 days)<br>3 Most or all of the time (5-7 days)
1701	[cesd_8_e_q2]<br>Show the field ONLY if:<br>[language_q2] = '1'	I felt hopeless about the future.	radio (Matrix)<br>0 Rarely or none of the time (< 1 day)<br>1 Some or a little of the time (1-2 days)<br>2 Occasionally or a moderate amount of the time (3-4 days)<br>3 Most or all of the time (5-7 days)
1702	[cesd_9_e_q2]<br>Show the field ONLY if:<br>[language_q2] = '1'	I thought my life had been a failure.	radio (Matrix)<br>0 Rarely or none of the time (< 1 day)<br>1 Some or a little of the time (1-2 days)<br>2 Occasionally or a moderate amount of the time (3-4 days)<br>3 Most or all of the time (5-7 days)
1703	[cesd_10_e_q2]<br>Show the field ONLY if:<br>[language_q2] = '1'	I felt fearful.	radio (Matrix)<br>0 Rarely or none of the time (< 1 day)<br>1 Some or a little of the time (1-2 days)<br>2 Occasionally or a moderate amount of the time (3-4 days)<br>3 Most or all of the time (5-7 days)
1704	[cesd_11_e_q2]<br>Show the field ONLY if:	My sleep was restless.	radio (Matrix)<br>0 Rarely or none of the time (< 1 day)

	[language_q2] = '1'		1 Some or a little of the time (1-2 days)<br>2 Occasionally or a moderate amount of the time (3-4 days)<br>3 Most or all of the time (5-7 days)
1705	[cesd_12_e_q2]<br>Show the field ONLY if:<br>[language_q2] = '1'	I was unhappy.	radio (Matrix)<br>0 Rarely or none of the time (< 1 day)<br>1 Some or a little of the time (1-2 days)<br>2 Occasionally or a moderate amount of the time (3-4 days)<br>3 Most or all of the time (5-7 days)
1706	[cesd_13_e_q2]<br>Show the field ONLY if:<br>[language_q2] = '1'	I talked less than usual.	radio (Matrix)<br>0 Rarely or none of the time (< 1 day)<br>1 Some or a little of the time (1-2 days)<br>2 Occasionally or a moderate amount of the time (3-4 days)<br>3 Most or all of the time (5-7 days)
1707	[cesd_14_e_q2]<br>Show the field ONLY if:<br>[language_q2] = '1'	I felt lonely.	radio (Matrix)<br>0 Rarely or none of the time (< 1 day)<br>1 Some or a little of the time (1-2 days)<br>2 Occasionally or a moderate amount of the time (3-4 days)<br>3 Most or all of the time (5-7 days)
1708	[cesd_15_e_q2]<br>Show the field ONLY if:<br>[language_q2] = '1'	People were unfriendly.	radio (Matrix)<br>0 Rarely or none of the time (< 1 day)<br>1 Some or a little of the time (1-2 days)<br>2 Occasionally or a moderate amount of the time (3-4 days)<br>3 Most or all of the time (5-7 days)
1709	[cesd_16_e_q2]<br>Show the field ONLY if:<br>[language_q2] = '1'	I did not enjoy life.	radio (Matrix)<br>0 Rarely or none of the time (< 1 day)<br>1 Some or a little of the time (1-2 days)<br>2 Occasionally or a moderate amount of the time (3-4 days)<br>3 Most or all of the time (5-7 days)
1710	[cesd_17_e_q2]<br>Show the field ONLY if:<br>[language_q2] = '1'	I had crying spells.	radio (Matrix)<br>0 Rarely or none of the time (< 1 day)<br>1 Some or a little of the time (1-2 days)<br>2 Occasionally or a moderate amount of the time (3-4 days)<br>3 Most or all of the time (5-7 days)
1711	[cesd_18_e_q2]	I felt sad.	radio (Matrix)

	Show the field ONLY if: [language_q2] = '1'		0 Rarely or none of the time (< 1 day)<br>1 Some or a little of the time (1-2 days)<br>2 Occasionally or a moderate amount of the time (3-4 days)<br>3 Most or all of the time (5-7 days)
1712	[cesd_19_e_q2]<br>Show the field ONLY if: [language_q2] = '1'	I felt that people disliked me.	radio (Matrix)<br>0 Rarely or none of the time (< 1 day)<br>1 Some or a little of the time (1-2 days)<br>2 Occasionally or a moderate amount of the time (3-4 days)<br>3 Most or all of the time (5-7 days)
1713	[cesd_20_e_q2]<br>Show the field ONLY if: [language_q2] = '1'	I could not get "going".	radio (Matrix)<br>0 Rarely or none of the time (< 1 day)<br>1 Some or a little of the time (1-2 days)<br>2 Occasionally or a moderate amount of the time (3-4 days)<br>3 Most or all of the time (5-7 days)
1714	[survey_e_q2]<br>Show the field ONLY if: [language_q2] = '1'	How did you complete this survey?	radio<br>1 on a computer (laptop or desktop)<br>2 on a mobile device (cell phone or tablet)<br>3 on a phone with an interviewer<br>4 other
1715	[work7_s_q2]<br>Show the field ONLY if: [language_q2] = '2'	¿Cuál de las siguientes es la mejor descripción de su situación laboral actual?	radio<br>1 Empleo a tiempo completo<br>2 Empleo a tiempo parcial<br>3 En busca de empleo<br>4 Jubilado<br>5 Ama de casa<br>6 Estudiante<br>7 Permiso de maternidad o paternidad<br>8 Permiso por enfermedad<br>9 Desempleado por discapacidad<br>10 Otra<br><br>Field Annotation: @DEFAULT="[work7_s_q2]"
1716	[work8_s_q2]<br>Show the field ONLY if: [language_q2] = '2'	¿Se considera usted actualmente empleado por cuenta propia (contratista independiente, trabajador esporádico (gig), etc.)?	radio<br>1 Sí<br>0 No

			2 No sabe<br><br>Field Annotation: @DEFAULT="[work8_s_q2]"
1717	[work12_s_q2]<br><br>Show the field ONLY if: [language_q2] = '2' and ([work7_s_q2] = '1' or [work7_s_q2] = '2' or [work8_s_q2] = '1')	En una escala de 0 (definitivamente no va a suceder) a 10 (definitivamente va a suceder), ¿qué tan probable es que usted pierda su empleo debido a la pandemia de COVID-19?	text (number, Min: 0, Max: 10)
1718	[work13_s_q2]<br><br>Show the field ONLY if: [language_q2] = '2' and ([work7_s_q2] = '1' or [work7_s_q2] = '2' or [work8_s_q2] = '1')	En una escala de 0 (definitivamente no va a suceder) a 10 (definitivamente va a suceder), ¿qué tan probable es que le asignen menos horas de trabajo debido a la pandemia de COVID-19?	text (number, Min: 0, Max: 10)
1719	[runout_s_q2]<br><br>Show the field ONLY if: [language_q2] = '2'	En su opinión ¿qué tan probable es que su grupo familiar se quede sin dinero en los próximos 3 meses? Dé su respuesta en una escala de 0 (definitivamente no va a suceder) a 10 (definitivamente va a suceder).	text (number, Min: 0, Max: 10)
1720	[work24_s_q2]<br><br>Show the field ONLY if: [language_q2] = '2' and ([work7_s_q2] = '1' or [work7_s_q2] = '2' or [work8_s_q2] = '1')	Actualmente, ¿con qué frecuencia se le exige que trabaje fuera de su residencia?	radio (Matrix)<br>1 Todo el tiempo (100%)<br>2 La mayor parte del tiempo (75%)<br>3 La mitad del tiempo (50%)<br>4 Menos de la mitad del tiempo (25%)<br>5 Nunca (0%)
1721	[work25_s_q2]<br><br>Show the field ONLY if: [language_q2] = '2' and ([work24_s_q2] = '1' or [work24_s_q2] = '2' or [work24_s_q2] = '3' or [work24_s_q2] = '4')	Actualmente, ¿con qué frecuencia se encuentra usted físicamente cerca de sus compañeros de trabajo mientras trabaja fuera de su residencia?	radio (Matrix)<br>1 Todo el tiempo (100%)<br>2 La mayor parte del tiempo (75%)<br>3 La mitad del tiempo (50%)<br>4 Menos de la mitad del tiempo (25%)<br>5 Nunca (0%)
1722	[work26_s_q2]<br><br>Show the field ONLY if: [language_q2] = '2' and ([work24_s_q2] = '1' or [work24_s_q2] = '2' or [work24_s_q2] = '3' or [work24_s_q2] = '4')	Actualmente, ¿con qué frecuencia se encuentra usted físicamente cerca de los clientes mientras trabaja fuera de su residencia?	radio (Matrix)<br>1 Todo el tiempo (100%)<br>2 La mayor parte del tiempo (75%)<br>3 La mitad del tiempo (50%)<br>4 Menos de la mitad del tiempo (25%)<br>5 Nunca (0%)
1723	[work27_s_q2]<br><br>Show the field ONLY if: [language_q2] = '2' and ([work24_s_q2] = '1' or [work24_s_q2] = '2' or	Actualmente, ¿con qué frecuencia tiene acceso a guantes desechables mientras trabaja fuera de su residencia?	radio (Matrix)<br>1 Todo el tiempo (100%)<br>2 La mayor parte del tiempo (75%)<br>3 La mitad del tiempo (50%)

	[work24_s_q2] = '3' or<br>[work24_s_q2] = '4')		4 Menos de la mitad del tiempo (25%)<br>5 Nunca (0%)
1724	[work28_s_q2]<br><br>Show the field ONLY if:<br>[language_q2] = '2' and<br>([work24_s_q2] = '1' or<br>[work24_s_q2] = '2' or<br>[work24_s_q2] = '3' or<br>[work24_s_q2] = '4')	Actualmente, ¿con qué frecuencia tiene acceso a una mascarilla mientras trabaja fuera de su residencia?	radio (Matrix)<br>1 Todo el tiempo (100%)<br>2 La mayor parte del tiempo (75%)<br>3 La mitad del tiempo (50%)<br>4 Menos de la mitad del tiempo (25%)<br>5 Nunca (0%)
1725	[work29_s_q2]<br><br>Show the field ONLY if:<br>[language_q2] = '2' and<br>([work24_s_q2] = '1' or<br>[work24_s_q2] = '2' or<br>[work24_s_q2] = '3' or<br>[work24_s_q2] = '4')	Actualmente, ¿con qué frecuencia lleva usted guantes desechables mientras trabaja fuera de su residencia?	radio (Matrix)<br>1 Todo el tiempo (100%)<br>2 La mayor parte del tiempo (75%)<br>3 La mitad del tiempo (50%)<br>4 Menos de la mitad del tiempo (25%)<br>5 Nunca (0%)
1726	[work30_s_q2]<br><br>Show the field ONLY if:<br>[language_q2] = '2' and<br>([work24_s_q2] = '1' or<br>[work24_s_q2] = '2' or<br>[work24_s_q2] = '3' or<br>[work24_s_q2] = '4')	Actualmente, ¿con qué frecuencia lleva usted mascarilla mientras trabaja fuera de su residencia?	radio (Matrix)<br>1 Todo el tiempo (100%)<br>2 La mayor parte del tiempo (75%)<br>3 La mitad del tiempo (50%)<br>4 Menos de la mitad del tiempo (25%)<br>5 Nunca (0%)
1727	[work31_s_q2]<br><br>Show the field ONLY if:<br>[language_q2] = '2' and<br>([work24_s_q2] = '1' or<br>[work24_s_q2] = '2' or<br>[work24_s_q2] = '3' or<br>[work24_s_q2] = '4')	Actualmente, ¿con qué frecuencia se lava las manos con agua y jabón mientras trabaja fuera de su residencia?	radio (Matrix)<br>1 Todo el tiempo (100%)<br>2 La mayor parte del tiempo (75%)<br>3 La mitad del tiempo (50%)<br>4 Menos de la mitad del tiempo (25%)<br>5 Nunca (0%)
1728	[work32_s_q2]<br><br>Show the field ONLY if:<br>[language_q2] = '2' and<br>([work24_s_q2] = '1' or<br>[work24_s_q2] = '2' or<br>[work24_s_q2] = '3' or<br>[work24_s_q2] = '4')	Actualmente, ¿con qué frecuencia se desinfecta las manos con desinfectante de manos mientras trabaja fuera de su residencia?	radio (Matrix)<br>1 Todo el tiempo (100%)<br>2 La mayor parte del tiempo (75%)<br>3 La mitad del tiempo (50%)<br>4 Menos de la mitad del tiempo (25%)<br>5 Nunca (0%)
1729	[work33_s_q2]<br><br>Show the field ONLY if:<br>[language_q2] = '2' and<br>([work24_s_q2] = '1' or<br>[work24_s_q2] = '2' or<br>[work24_s_q2] = '3' or<br>[work24_s_q2] = '4')	Actualmente, ¿cuánto le preocupa exponerse a la COVID-19 mientras trabaja fuera de su residencia?	radio (Matrix)<br>1 Todo el tiempo (100%)<br>2 La mayor parte del tiempo (75%)<br>3 La mitad del tiempo (50%)<br>4 Menos de la mitad del tiempo (25%)<br>5 Nunca (0%)

1730	[work34_s_q2]<br><br>Show the field ONLY if: [language_q2] = '2' and ([work7_s_q2] = '1' or [work7_s_q2] = '2' or [work8_s_q2] = '1')	¿Trabaja usted actualmente en alguno de los siguientes ambientes de alto riesgo de contagio de COVID-19?	checkbox<br>1 | work34_s_q2___1 | Ambiente de asistencia médica (hospital, clínica, centro de urgencias)<br>2 | work34_s_q2___2 | Ambiente residencial denso (hogar de ancianos, otro centro de asistencia de larga duración)<br>3 | work34_s_q2___3 | Prisión o cárcel<br>4 | work34_s_q2___4 | Establecimiento de envasado de carne<br>5 | work34_s_q2___5 | Establecimiento de envío o distribución<br>6 | work34_s_q2___6 | Establecimiento minorista de alto volumen (tienda de provisiones, etc.)
1731	[insur_s_q2]<br><br>Show the field ONLY if: [language_q2] = '2'	¿Tiene usted seguro médico o de salud (seguro privado, seguro que usted ha comprado, Medicare, Medicaid o algún otro tipo de seguro)?	radio<br>1 | Sí<br>0 | No<br>2 | No sabe<br><br>Field Annotation: @DEFAULT=" [insur_s_q2]"
1732	[insur2_s_q2]<br><br>Show the field ONLY if: [language_q2] = '2' and [insur_s_q2] = '1'	¿Cuál es su principal seguro de salud?<br>*(Seleccione uno (su seguro principal).)*	radio<br>1 | Seguro médico privado por empleo o establecimiento educacional<br>2 | Seguro comprado en un intercambio de seguros de salud estatal o federal, tal como healthcare.gov<br>3 | Seguro comprado directamente a una compañía de seguros.<br>4 | Medicare<br>5 | Medi-Gap<br>6 | Medicaid<br>7 | Asistencia médica militar (TRICARE, VA, CHAMP-VA, etc.)<br>8 | Servicio de Salud Indígena<br>9 | Otro

			Field Annotation: @DEFAULT="[insur2_s_q2]"
1733	[insur3_s_q2]<br>Show the field ONLY if: [language_q2] = '2' and [insur2_s_q2] = '9'	Si marcó "Otro", especifique la fuente de seguro médico.	text<br>Field Annotation: @DEFAULT="[insur3_s_q2]"
1734	[pregnant_s_q2]<br>Show the field ONLY if: [language_q2] = '2' and [sex] = '1'	¿Está usted actualmente embarazada?	radio<br>1 Sí<br>0 No
1735	[srh2_s_q2]<br>Show the field ONLY if: [language_q2] = '2'	En general, ¿cómo calificaría usted su salud en las últimas dos semanas?	radio (Matrix)<br>1 Excelente<br>2 Muy buena<br>3 Buena<br>4 Regular<br>5 Mala
1736	[prevent_s_q2]<br>Show the field ONLY if: [language_q2] = '2'	Section Header: *Con que frequencia ha hecho lo siguiente durante las ultimas dos semanas para protegerse contra la infeccion?*<br>Llevar mascarilla	radio (Matrix)<br>1 Siempre (100%)<br>2 La mayor parte del tiempo (75%)<br>3 La mitad del tiempo (50%)<br>4 Menos de la mitad del tiempo (25%)<br>5 Nunca (0%)
1737	[prevent2_s_q2]<br>Show the field ONLY if: [language_q2] = '2'	Lavarse las manos y/o limpiárselas con desinfectante a menudo	radio (Matrix)<br>1 Siempre (100%)<br>2 La mayor parte del tiempo (75%)<br>3 La mitad del tiempo (50%)<br>4 Menos de la mitad del tiempo (25%)<br>5 Nunca (0%)
1738	[prevent3_s_q2]<br>Show the field ONLY if: [language_q2] = '2'	Mantenerse al menos a 6 pies de distancia de los demás	radio (Matrix)<br>1 Siempre (100%)<br>2 La mayor parte del tiempo (75%)<br>3 La mitad del tiempo (50%)<br>4 Menos de la mitad del tiempo (25%)<br>5 Nunca (0%)

1739	[prevent4_s_q2]<br>Show the field ONLY if:<br>[language_q2] = '2'	Evitar grandes reuniones, espacios públicos o multitudes	radio (Matrix)<br>1 Siempre (100%)<br>2 La mayor parte del tiempo (75%)<br>3 La mitad del tiempo (50%)<br>4 Menos de la mitad del tiempo (25%)<br>5 Nunca (0%)
1740	[prevent5_s_q2]<br>Show the field ONLY if:<br>[language_q2] = '2'	Evitar el contacto con personas de alto riesgo	radio (Matrix)<br>1 Siempre (100%)<br>2 La mayor parte del tiempo (75%)<br>3 La mitad del tiempo (50%)<br>4 Menos de la mitad del tiempo (25%)<br>5 Nunca (0%)
1741	[prevent6_s_q2]<br>Show the field ONLY if:<br>[language_q2] = '2'	No comer en restaurante, ni siquiera comprar comida para llevar	radio (Matrix)<br>1 Siempre (100%)<br>2 La mayor parte del tiempo (75%)<br>3 La mitad del tiempo (50%)<br>4 Menos de la mitad del tiempo (25%)<br>5 Nunca (0%)
1742	[prevent7_s_q2]<br>Show the field ONLY if:<br>[language_q2] = '2'	Trabajar o estudiar en casa en lugar de ir a una oficina o a un aula	radio (Matrix)<br>1 Siempre (100%)<br>2 La mayor parte del tiempo (75%)<br>3 La mitad del tiempo (50%)<br>4 Menos de la mitad del tiempo (25%)<br>5 Nunca (0%)
1743	[prevent8_s_q2]<br>Show the field ONLY if:<br>[language_q2] = '2'	No dar la mano ni tocar a otras personas.	radio (Matrix)<br>1 Siempre (100%)<br>2 La mayor parte del tiempo (75%)<br>3 La mitad del tiempo (50%)<br>4 Menos de la mitad del tiempo (25%)<br>5 Nunca (0%)
1744	[prevent9_s_q2]<br>Show the field ONLY if:<br>[language_q2] = '2'	Quedarse en casa cuando se está enfermo	radio (Matrix)<br>1 Siempre (100%)<br>2 La mayor parte del tiempo (75%)<br>3 La mitad del tiempo (50%)<br>4 Menos de la mitad del tiempo (25%)<br>5 Nunca (0%)
1745	[prevent10_s_q2]<br>Show the field ONLY if:<br>[language_q2] = '2'	Limpiar las superficies con desinfectante	radio (Matrix)<br>1 Siempre (100%)<br>2 La mayor parte del tiempo (75%)

			3 La mitad del tiempo (50%)<br>4 Menos de la mitad del tiempo (25%)<br>5 Nunca (0%)
1746	[prevent11_s_q2]<br><br>Show the field ONLY if:<br>[language_q2] = '2'	Cancelar o postergar viajes de trabajo	radio (Matrix)<br>1 Siempre (100%)<br>2 La mayor parte del tiempo (75%)<br>3 La mitad del tiempo (50%)<br>4 Menos de la mitad del tiempo (25%)<br>5 Nunca (0%)
1747	[prevent12_s_q2]<br><br>Show the field ONLY if:<br>[language_q2] = '2'	Cancelar o postergar viajes de placer	radio (Matrix)<br>1 Siempre (100%)<br>2 La mayor parte del tiempo (75%)<br>3 La mitad del tiempo (50%)<br>4 Menos de la mitad del tiempo (25%)<br>5 Nunca (0%)
1748	[prevent13_s_q2]<br><br>Show the field ONLY if:<br>[language_q2] = '2'	Cancelar o postergar viajes las actividades personales o sociales	radio (Matrix)<br>1 Siempre (100%)<br>2 La mayor parte del tiempo (75%)<br>3 La mitad del tiempo (50%)<br>4 Menos de la mitad del tiempo (25%)<br>5 Nunca (0%)
1749	[prevent14_s_q2]<br><br>Show the field ONLY if:<br>[language_q2] = '2'	Cancelar las consultas médicas en persona	radio (Matrix)<br>1 Siempre (100%)<br>2 La mayor parte del tiempo (75%)<br>3 La mitad del tiempo (50%)<br>4 Menos de la mitad del tiempo (25%)<br>5 Nunca (0%)
1750	[prevent15_s_q2]<br><br>Show the field ONLY if:<br>[language_q2] = '2'	Almacenar comida o agua	radio (Matrix)<br>1 Siempre (100%)<br>2 La mayor parte del tiempo (75%)<br>3 La mitad del tiempo (50%)<br>4 Menos de la mitad del tiempo (25%)<br>5 Nunca (0%)
1751	[prevent16_s_q2]<br><br>Show the field ONLY if:<br>[language_q2] = '2'	Seguir las recomendaciones o reglas del gobierno sobre refugiarse donde se esté (es decir, quedarse en casa, limitar el contacto con otras personas, etc.)	radio (Matrix)<br>1 Siempre (100%)<br>2 La mayor parte del tiempo (75%)<br>3 La mitad del tiempo (50%)<br>4 Menos de la mitad del tiempo (25%)

			5 Nunca (0%)
1752	[fever_s_q2]<br>Show the field ONLY if:<br>[language_q2] = '2'	Section Header: *Durante los ultimos dos semanas, ha tenido alguno de los siguientes sintomas?*<br>Fiebre (medida por termómetro o autodiagnóstico)	radio (Matrix)<br>1 Sí<br>0 No
1753	[cough_s_q2]<br>Show the field ONLY if:<br>[language_q2] = '2'	Tos (nueva o que empeora)	radio (Matrix)<br>1 Sí<br>0 No
1754	[sob_s_q2]<br>Show the field ONLY if:<br>[language_q2] = '2'	Dificultad para respirar (nueva o que empeora)	radio (Matrix)<br>1 Sí<br>0 No
1755	[fatigue_s_q2]<br>Show the field ONLY if:<br>[language_q2] = '2'	Fatiga (nuevo cansancio al hacer actividades normales)	radio (Matrix)<br>1 Sí<br>0 No
1756	[bodyache_s_q2]<br>Show the field ONLY if:<br>[language_q2] = '2'	Dolores de cuerpo	radio (Matrix)<br>1 Sí<br>0 No
1757	[headache_s_q2]<br>Show the field ONLY if:<br>[language_q2] = '2'	Dolor de cabeza	radio (Matrix)<br>1 Sí<br>0 No
1758	[diarrhea_s_q2]<br>Show the field ONLY if:<br>[language_q2] = '2'	Diarrea	radio (Matrix)<br>1 Sí<br>0 No
1759	[pharyngitis_s_q2]<br>Show the field ONLY if:<br>[language_q2] = '2'	Dolor de garganta	radio (Matrix)<br>1 Sí<br>0 No
1760	[eye_s_q2]<br>Show the field ONLY if:<br>[language_q2] = '2'	Picazón, enrojecimiento o dolor de ojos	radio (Matrix)<br>1 Sí<br>0 No
1761	[congest_s_q2]<br>Show the field ONLY if:<br>[language_q2] = '2'	Goteo o congestión nasal	radio (Matrix)<br>1 Sí<br>0 No
1762	[sensory_s_q2]<br>Show the field ONLY if:<br>[language_q2] = '2'	Cambios en el sentido del olfato o del gusto	radio (Matrix)<br>1 Sí<br>0 No
1763	[rash_s_q2]<br>Show the field ONLY if:<br>[language_q2] = '2'	Nuevo sarpullido	radio (Matrix)<br>1 Sí<br>0 No
1764	[chills_s_q2]	Temblores y escalofríos reiterados	radio (Matrix)

	Show the field ONLY if: [language_q2] = '2'		1 Sí<br>0 No
1765	[covid_symp_s_q2]<br><br>Show the field ONLY if: [language_q2] = '2' and ([fever_s_q2] = '1' or [cough_s_q2] = '1' or [sob_s_q2] = '1' or [fatigue_s_q2] = '1' or [bodyache_s_q2] = '1' or [headache_s_q2] = '1' or [diarrhea_s_q2] = '1' or [pharyngitis_s_q2] = '1' or [eye_s_q2] = '1' or [congest_s_q2] = '1' or [sensory_s_q2] = '1' or [rash_s_q2] = '1' or [chills_s_q2] = '1')	¿Cuándo comenzaron estos síntomas?	text (date_mdy)
1766	[covid_symp3_s_q2]<br><br>Show the field ONLY if: [language_q2] = '2' and ([fever_s_q2] = '1' or [cough_s_q2] = '1' or [sob_s_q2] = '1' or [fatigue_s_q2] = '1' or [bodyache_s_q2] = '1' or [headache_s_q2] = '1' or [diarrhea_s_q2] = '1' or [pharyngitis_s_q2] = '1' or [eye_s_q2] = '1' or [congest_s_q2] = '1' or [sensory_s_q2] = '1' or [rash_s_q2] = '1' or [chills_s_q2] = '1')	¿Fue usted objeto de prejuicio o discriminación debido a sus síntomas?	radio<br>1 Sí<br>0 No<br>2 No sabe
1767	[prevent17_s_q2]<br><br>Show the field ONLY if: [language_q2] = '2' and ([fever_s_q2] = '1' or [cough_s_q2] = '1' or [sob_s_q2] = '1' or [fatigue_s_q2] = '1' or [bodyache_s_q2] = '1' or [headache_s_q2] = '1' or [diarrhea_s_q2] = '1' or [pharyngitis_s_q2] = '1' or [eye_s_q2] = '1' or [congest_s_q2] = '1' or [sensory_s_q2] = '1' or [rash_s_q2] = '1' or [chills_s_q2] = '1')	¿Cuáles de las siguientes medidas tomó usted para proteger a sus amigos y familiares después de que comenzaron sus síntomas?	checkbox<br>1 prevent17_s_q2___1 Ponerse mascarilla con mayor frecuencia<br>2 prevent17_s_q2___2 Lavarse las manos con agua y jabón con mayor frecuencia<br>3 prevent17_s_q2___3 Limpiarse las manos con desinfectante con mayor frecuencia<br>4 prevent17_s_q2___4 Aislarse en su casa con mayor frecuencia

			5 | prevent17_s_q2___5 | Quedarse en casa con mayor frecuencia<br>6 | prevent17_s_q2___6 | Ponerse guantes desechables con mayor frecuencia
1768	[covid_symp4_s_q2]<br><br>Show the field ONLY if: [language_q2] = '2' and ([fever_s_q2] = '1' or [cough_s_q2] = '1' or [sob_s_q2] = '1' or [fatigue_s_q2] = '1' or [bodyache_s_q2] = '1' or [headache_s_q2] = '1' or [diarrhea_s_q2] = '1' or [pharyngitis_s_q2] = '1' or [eye_s_q2] = '1' or [congest_s_q2] = '1' or [sensory_s_q2] = '1' or [rash_s_q2] = '1' or [chills_s_q2] = '1')	¿Qué hizo usted en vista de sus síntomas?<br>*(Seleccione todo lo que corresponda.)*	checkbox<br>0 | covid_symp4_s_q2___0 | Nada<br>1 | covid_symp4_s_q2___1 | Tomó medicamentos sin receta (ibuprofeno, acetaminofén, etc.)<br>2 | covid_symp4_s_q2___2 | Consultó por teléfono a un proveedor de asistencia médica<br>3 | covid_symp4_s_q2___3 | Fue al consultorio de un proveedor de asistencia<br>4 | covid_symp4_s_q2___4 | Fue a una clínica o una farmacia minorista<br>5 | covid_symp4_s_q2___5 | Fue a un centro de urgencias (FASTmed, etc.)<br>6 | covid_symp4_s_q2___6 | Fue a la sala de emergencias<br>7 | covid_symp4_s_q2___7 | Fue ingresado al hospital<br>8 | covid_symp4_s_q2___8 | Otra cosa
1769	[covid_symp5_s_q2]<br><br>Show the field ONLY if: [language_q2] = '2' and [covid_symp4_s_q2(8)] = '1'	¿Qué otra cosa hizo usted en vista de sus síntomas?	text
1770	[covid_symp6_s_q2]<br><br>Show the field ONLY if: [language_q2] = '2' and ([covid_symp4_s_q2(2)] = '1' or [covid_symp4_s	Si pudo hablar con un proveedor de asistencia médica, ¿le dijo el proveedor que era posible que tuviera COVID-19?	radio<br>1 | Sí<br>0 | No<br>2 | No sabe

	_q2(3)] = '1' or [covid_symp4_s_q2(4)] = '1' or [covid_symp4_s_q2(5)] = '1' or [covid_symp4_s_q2(6)] = '1' or [covid_symp4_s_q2(7)] = '1' or [covid_symp4_s_q2(8)] = '1')		
1771	[covid_test_s_q2]<br><br>Show the field ONLY if:<br>[language_q2] = '2' and ([fever_s_q2] = '1' or [cough_s_q2] = '1' or [sob_s_q2] = '1' or [fatigue_s_q2] = '1' or [bodyache_s_q2] = '1' or [headache_s_q2] = '1' or [diarrhea_s_q2] = '1' or [pharyngitis_s_q2] = '1' or [eye_s_q2] = '1' or [congest_s_q2] = '1' or [sensory_s_q2] = '1' or [rash_s_q2] = '1' or [chills_s_q2] = '1')	Si le hicieron una prueba de COVID-19 en vista de los síntomas que declaró, ¿cuál fue el resultado?	radio<br>1 Pendiente<br>2 Positivo<br>3 Negativo<br>4 No concluyente<br>5 No se hizo la prueba
1772	[covid_admit_s_q2]<br><br>Show the field ONLY if:<br>[language_q2] = '2' and [covid_symp4_s_q2(7)] = '1'	¿Cuántos días estuvo usted hospitalizado?	text (number, Min: 0)
1773	[covid_admit2_s_q2]<br><br>Show the field ONLY if:<br>[language_q2] = '2' and [covid_symp4_s_q2(7)] = '1'	¿Le hicieron a usted las siguientes intervenciones durante su hospitalización?	checkbox<br>1 covid_admit2_s_q2___1 Oxígeno adicional por la nariz<br>2 covid_admit2_s_q2___2 Tratamiento en la Unidad de Cuidados Intensivos (Intensive Care Unit, ICU)<br>3 covid_admit2_s_q2___3 Ventilación mecánica (intubación o tubo de respiración)
1774	[covid_symp7_s_q2]<br><br>Show the field ONLY if:<br>[language_q2] = '2' and ([fever_s_q2] = '1' or [cough_s_q2] = '1' or [sob_s_q2] = '1' or [fatigue_s_q2] = '1' or [bodyache_s_q2] = '1' or [headache_s_q2] = '1' or [diarrhea	¿Ha vuelto usted a su salud normal?	radio<br>1 Sí<br>0 No<br>2 No sabe

	_s_q2] = '1' or [pharyngitis_s_q2] = '1' or [eye_s_q2] = '1' or [congest_s_q2] = '1' or [sensory_s_q2] = '1' or [rash_s_q2] = '1' or [chills_s_q2] = '1')		
1775	[vaccine_trial_s_q2]<br>Show the field ONLY if: [language_q2] = '2'	¿Ha participado o está participando actualmente en un ensayo de la vacuna de COVID?	yesno<br>1 Yes<br>0 No
1776	[vaccine_trial2_s_q2]<br>Show the field ONLY if: [language_q2] = '2' and [vaccine_trial_s_q2] = '1'	¿Dónde fue o es el sitio principal para el ensayo de la vacuna de COVID? (Por ejemplo, ¿UNC, Duke o un hospital específico?)	text
1777	[receivedcovidvaccine_s_q2]<br>Show the field ONLY if: [language_q2] = '2'	¿Ha recibido una vacuna de COVID-19 fuera de un ensayo clínico?	radio<br>1 Sí<br>0 No
1778	[vacclast2week_s]<br>Show the field ONLY if: [receivedcovidvaccine_s_q2] = '1'	¿Recibió la vacuna COVID-19 desde que llenó su última encuesta quincenal?	radio<br>0 No<br>1 Sí
1779	[receivedvaccine_where_s_q2]<br>Show the field ONLY if: [language_q2] = '2' and [vacclast2week_s] = '1'	¿Dónde recibió la vacuna de COVID-19?	radio<br>1 Oficina del doctor/medico<br>2 Trabajo/Empleo<br>3 Minorista (por ejemplo Walgreens, CVS)<br>5 Sitio de la vacuna<br>4 Otro
1780	[receivedvaccine_where_oth_s_q2]<br>Show the field ONLY if: [language_q2] = '2' and [vacclast2week_s] = '1' and [receivedvaccine_where_s_q2] = '4'	Especifique donde recibió la vacuna de COVID-19.	text
1781	[receivedvaccine_site_s_q2]<br>Show the field ONLY if: [language_q2] = '2' and [vacclast2week_s] = '1' and [receivedvaccine_where_s_q2] = '5'	Especifique la cuidad/el pueblo en Carolina del Norte del sitio de la vacuna:	text
1782	[receivedvaccine_site2_s_q2]	Especifique quien fue la organización o instituto que organizo el sitio de la vacuna:	text

	Show the field ONLY if: [language_q2] = '2' and [vacclast2week_s] = '1' and [receivedvaccine_where_s_q2] = '5'		
1783	[receivedvaccine_name_s_q2]<br><br>Show the field ONLY if: [language_q2] = '2' and [vacclast2week_s] = '1'	¿Cuál vacuna de COVID-19 recibió?	radio<br>1 Pfizer<br>2 Moderna<br>3 AstraZeneca<br>4 Novavax<br>6 Johnson & Johnson<br>5 Otro<br>9 No sabe
1784	[receivedvaccine_name_oth_s_q2]<br><br>Show the field ONLY if: [language_q2] = '2' AND [vacclast2week_s] = '1' AND [receivedvaccine_name_s_q2] = '5'	Especifique que otra vacuna de COVID recibió.	text
1785	[receivedvaccine_doses_s_q2]<br><br>Show the field ONLY if: [language_q2] = '2' and [vacclast2week_s] = '1'	¿Cuántas dosis de la vacuna ha recibido?	radio<br>1 1<br>2 2
1786	[receivedvaccine_when_s_q2]<br><br>Show the field ONLY if: [language_q2] = '2' and [vacclast2week_s] = '1' and [receivedvaccine_doses_s_q2] = '1' or [receivedvaccine_doses_s_q2]='2'	¿Qué día recibió la primera dosis de la vacuna?	text (date_mdy), Required
1787	[receivedvaccine_second_dt_s_q2]<br><br>Show the field ONLY if: [language_q2] = '2' and [vacclast2week_s] = '1' and [receivedvaccine_doses_s_q2] = '2'	¿Qué día recibió la segunda dosis de la vacuna?	text (date_mdy), Required
1788	[vacc_pain_s_q2]<br><br>Show the field ONLY if: [language_q2] = '2' and [vacclast2week_s] = '1'	Section Header: *Did you experience any of the following side effects after vaccination?*<br><br>dolor en o alrededor del lugar de la inyección	radio (Matrix)<br>0 No<br>1 Leve (nota síntomas, pero no son un problema)<br>2 Moderado (los síntomas limitan sus actividades diarias normales)

			3 Grave (los síntomas hacen que las actividades normales sean difícil o imposible)<br><br>Question number: 1
1789	[vacc_redness_s_q2]<br><br>Show the field ONLY if: [language_q2] = '2' and [vacclast2week_s] = '1'	enrojecimiento en o alrededor del lugar de la inyección	radio (Matrix)<br>0 No<br>1 Leve (nota síntomas, pero no son un problema)<br>2 Moderado (los síntomas limitan sus actividades diarias normales)<br>3 Grave (los síntomas hacen que las actividades normales sean difícil o imposible)<br><br>Question number: 2
1790	[vacc_swell_s_q2]<br><br>Show the field ONLY if: [language_q2] = '2' and [vacclast2week_s] = '1'	hinchazón en o alrededor del lugar de la inyección	radio (Matrix)<br>0 No<br>1 Leve (nota síntomas, pero no son un problema)<br>2 Moderado (los síntomas limitan sus actividades diarias normales)<br>3 Grave (los síntomas hacen que las actividades normales sean difícil o imposible)<br><br>Question number: 3
1791	[vacc_rash_s_q2]<br><br>Show the field ONLY if: [language_q2] = '2' and [vacclast2week_s] = '1'	erupción en o alrededor del lugar de la inyección	radio (Matrix)<br>0 No<br>1 Leve (nota síntomas, pero no son un problema)<br>2 Moderado (los síntomas limitan sus actividades diarias normales)<br>3 Grave (los síntomas hacen que las actividades normales sean difícil o imposible)<br><br>Question number: 4
1792	[vacc_headache_s_q2]<br><br>Show the field ONLY if: [language_q2] = '2' and [vacclast2week_s] = '1'	dolor de cabeza	radio (Matrix)<br>0 No<br>1 Leve (nota síntomas, pero no son un problema)<br>2 Moderado (los síntomas limitan sus actividades diarias normales)<br>3 Grave (los síntomas hacen que las actividades normales sean difícil o imposible)

			Question number: 5
1793	[vacc_fatigue_s_q2]<br>Show the field ONLY if:<br>[language_q2] = '2' and<br>[vacclast2week_s] = '1'	fatiga/cansancio	radio (Matrix)<br>0 No<br>1 Leve (nota síntomas, pero no son un problema)<br>2 Moderado (los síntomas limitan sus actividades diarias normales)<br>3 Grave (los síntomas hacen que las actividades normales sean difícil o imposible)<br>Question number: 6
1794	[vacc_fever_s_q2]<br>Show the field ONLY if:<br>[language_q2] = '2' and<br>[vacclast2week_s] = '1'	fiebre (temperatura >100.4°F o >38°C)	radio (Matrix)<br>0 No<br>1 Leve (nota síntomas, pero no son un problema)<br>2 Moderado (los síntomas limitan sus actividades diarias normales)<br>3 Grave (los síntomas hacen que las actividades normales sean difícil o imposible)<br>Question number: 7
1795	[vacc_chills_s_q2]<br>Show the field ONLY if:<br>[language_q2] = '2' and<br>[vacclast2week_s] = '1'	escalofríos	radio (Matrix)<br>0 No<br>1 Leve (nota síntomas, pero no son un problema)<br>2 Moderado (los síntomas limitan sus actividades diarias normales)<br>3 Grave (los síntomas hacen que las actividades normales sean difícil o imposible)<br>Question number: 8
1796	[vacc_joint_s_q2]<br>Show the field ONLY if:<br>[language_q2] = '2' and<br>[vacclast2week_s] = '1'	dolor en las articulaciones	radio (Matrix)<br>0 No<br>1 Leve (nota síntomas, pero no son un problema)<br>2 Moderado (los síntomas limitan sus actividades diarias normales)<br>3 Grave (los síntomas hacen que las actividades normales sean difícil o imposible)<br>Question number: 9
1797	[vacc_muscle_s_q2]	dolor muscular	radio (Matrix)

	Show the field ONLY if: [language_q2] = '2' and [vacclast2week_s] = '1'		0 No<br>1 Leve (nota síntomas, pero no son un problema)<br>2 Moderado (los síntomas limitan sus actividades diarias normales)<br>3 Grave (los síntomas hacen que las actividades normales sean difícil o imposible)<br><br>Question number: 10
1798	[vacc_nausea_s_q2]<br><br>Show the field ONLY if: [language_q2] = '2' and [vacclast2week_s] = '1'	nausea	radio (Matrix)<br>0 No<br>1 Leve (nota síntomas, pero no son un problema)<br>2 Moderado (los síntomas limitan sus actividades diarias normales)<br>3 Grave (los síntomas hacen que las actividades normales sean difícil o imposible)<br><br>Question number: 11
1799	[vacc_effects_s_q2]<br><br>Show the field ONLY if: [vacc_pain_s_q2]>0 OR [vacc_redness_s_q2]>0 OR [vacc_swell_s_q2]>0 OR [vacc_rash_s_q2]>0 OR [vacc_headache_s_q2]>0 OR [vacc_fatigue_s_q2]>0 OR [vacc_fever_s_q2]>0 OR [vacc_chills_s_q2]>0 OR [vacc_joint_s_q2]>0 OR [vacc_muscle_s_q2]>0 OR [vacc_nausea_s_q2]>0	¿Cuánto tiempo duraron estos efectos secundarios?	radio<br>1 menos de 12 horas<br>2 12 a 24 horas<br>3 más de 24 horas
1800	[vacc_med_s_q2]<br><br>Show the field ONLY if: [vacc_pain_s_q2]>0 OR [vacc_redness_s_q2]>0 OR [vacc_swell_s_q2]>0 OR [vacc_rash_s_q2]>0 OR [vacc_headache_s_q2]>0 OR [vacc_fatigue_s_q2]>0 OR [vacc_fever_s_q2]>0 OR [vacc_chills_s_q2]>0 OR [vacc_joint_s_q2]>0 OR [vacc_muscle_s_q2]>0 OR [vacc_nausea_s_q2]>0	¿Tomo algún medicamento para estos efectos secundarios?	radio<br>1 Sí<br>0 No
1801	[vacc_med_list_s_q2]	¿Que medicamento(s) tomo para los efectos secundarios?	text

	Show the field ONLY if: [vacc_med_s_q2] = '1'	*Por favor enumere todos los medicamentos.*	
1802	[vacc_effects_consult_s_q2]<br><br>Show the field ONLY if: [vacc_pain_s_q2]>0 OR [vacc_redness_s_q2]>0 OR [vacc_swell_s_q2]>0 OR [vacc_rash_s_q2]>0 OR [vacc_headache_s_q2]>0 OR [vacc_fatigue_s_q2]>0 OR [vacc_fever_s_q2]>0 OR [vacc_chills_s_q2]>0 OR [vacc_joint_s_q2]>0 OR [vacc_muscle_s_q2]>0 OR [vacc_nausea_s_q2]>0	¿Consultó a un médico u otro proveedor de atención médica para los efectos secundarios?	radio<br>1 Sí<br>0 No
1803	[vacc_effects_dose_s_q2]<br><br>Show the field ONLY if: [vacc_pain_s_q2]>0 OR [vacc_redness_s_q2]>0 OR [vacc_swell_s_q2]>0 OR [vacc_rash_s_q2]>0 OR [vacc_headache_s_q2]>0 OR [vacc_fatigue_s_q2]>0 OR [vacc_fever_s_q2]>0 OR [vacc_chills_s_q2]>0 OR [vacc_joint_s_q2]>0 OR [vacc_muscle_s_q2]>0 OR [vacc_nausea_s_q2]>0	¿Como experimento los efectos secundarios después de la secunda dosis de la vacuna en comparación con los efectos secundarios después de la primera dosis de la vacuna?	radio<br>3 más grave<br>2 menos grave<br>1 igualmenta grave<br>0 no corresponde/aún no he recibido la segunda dosis
1804	[receive_booster_s_q2]<br><br>Show the field ONLY if: [language_q2] = '2'	¿Ha recibido una dosis de refuerzo de la vacuna COVID-19?	radio<br>1 Sí<br>0 No
1805	[receive_booster_num_s]<br><br>Show the field ONLY if: [receive_booster_s_q2] = '1'	Por favor, indique la cantidad de dosis de refuerzo que ha recibido:<br>*Por ejemplo: seleccione 2 si recibió 4 dosis de vacunas ARNm, o 2 dosis de vacunas ARNm y 1 de J&J*	dropdown<br>1 1<br>2 2<br>3 3<br>4 4<br>5 5<br>6 6<br>7 7<br>8 8<br>9 9<br>10 10

1806	[boosterlast2week_s]<br><br>Show the field ONLY if:<br>[receive_booster_s_q2] = '1'	¿Recibió un refuerzo de COVID-19 desde que llenó su última encuesta quincenal?	radio<br>0 No<br>1 Sí
1807	[receive_booster2_s_q2]<br><br>Show the field ONLY if:<br>[language_q2] = '2' and [boosterlast2week_s] = '1'	¿Tiene previsto recibir una dosis de refuerzo de COVID-19?	radio<br>1 Sí<br>2 No<br>3 No lo sé
1808	[receive_booster3_s_q2]<br><br>Show the field ONLY if:<br>[language_q2] = '2' and [boosterlast2week_s] = '1'	¿Cuál es el factor principal que ha influido su decisión de recibir la dosis de refuerzo de la vacuna COVID-19 en este momento?<br>*Marque todo lo que corresponda*	radio<br>1 Era necesario para mi empleo<br>2 Mi estado de salud<br>3 Mi edad<br>4 Sugerencia médica<br>5 Preocupación que la vacuna que tomé no proporcionó una protección suficientemente fuerte<br>6 Preocupación por la transmisión del virus a otras personas<br>7 Otra razón
1809	[receive_booster3oth_s_q2]<br><br>Show the field ONLY if:<br>[language_q2] = '2' and [receive_booster3_s_q2] = '7' and [boosterlast2week_s] = '1'	Especifique que otra factor principal que ha influido se decisión	text
1810	[receive_booster4_s_q2]<br><br>Show the field ONLY if:<br>[language_q2] = '2' and [receive_booster_s_q2] = '0'	¿Cuál es el factor principal que influye en su decisión de NO recibir la dosis de refuerzo de la vacuna COVID-19 en este momento?	checkbox<br>1 receive_booster4_s_q2___1 No está disponible para mí<br>2 receive_booster4_s_q2___2 No pensé que fuera necesaria para mí<br>3 receive_booster4_s_q2___3 No me pareció étic[illegible] que otros n[illegible] hayan recibido la primera dosis<br>4 receive_booster4_s_q2___4 Preocupació[illegible] por su inocuidad

			5 receive_booster4_s_q2___5 Simplemente no quiero tomar la dosis de refuerzo<br>6 receive_booster4_s_q2___6 Otra razón
1811	[receive_booster4oth_s_q2]<br><br>Show the field ONLY if: [language_q2] = '2' and [boosterlast2week_s] = '1' and [receive_booster4_s_q2(6)] = '1'	Especifique que otra factor principal que ha influido se decisión de NO recibir la dosis de refuerzo de la vacuna COVID-19 en este momento	text
1812	[receive_booster5_s_q2]<br><br>Show the field ONLY if: [language_q2] = '2' and [boosterlast2week_s] = '1'	¿Qué dosis de refuerzo de COVID-19 recibió?	radio<br>1 Pfizer<br>2 Moderna<br>3 J&J<br>4 Otra
1813	[receive_booster5oth_s_q2]<br><br>Show the field ONLY if: [language_q2] = '2' and [boosterlast2week_s] = '1' and [receive_booster5_s_q2] = '4'	Especifique qué otra vacuna de refuerzo de COVID-19 ha recibido:	text
1814	[receive_boosterdt_s_q2]<br><br>Show the field ONLY if: [language_q2] = '2' and [boosterlast2week_s] = '1'	¿En qué fecha recibió su dosis de refuerzo de COVID-19?	text (date_mdy)
1815	[receive_boosterloc_s_q2]<br><br>Show the field ONLY if: [language_q2] = '2' and [boosterlast2week_s] = '1'	¿En dónde recibió su dosis de refuerzo de COVID-19?	text
1816	[booster_pain_s_q2]<br><br>Show the field ONLY if: [language_q2] = '2' and [boosterlast2week_s] = '1'	Section Header: *Por favor, indique si tuvo alguno de los siguientes síntomas después de recibir la dosis de refuerzo:*<br><br>Dolor en el lugar de la inyección o a su alrededor	radio (Matrix)<br>0 No<br>1 Leve (notas los síntomas, pero no son un problema)<br>2 Moderado (los síntomas limitan sus actividades diarias)<br>3 Grave (los síntomas dificultan o impiden por completo sus actividades diarias)

1817	[booster_redness_s_q2]<br><br>Show the field ONLY if: [language_q2] = '2' and [boosterlast2week_s] = '1'	Enrojecimiento en el lugar de la inyección o a su alrededor	radio (Matrix)<br>0 No<br>1 Leve (notas los síntomas, pero no son un problema)<br>2 Moderado (los síntomas limitan sus actividades diarias)<br>3 Grave (los síntomas dificultan o impiden por completo sus actividades diarias)
1818	[booster_swelling_s_q2]<br><br>Show the field ONLY if: [language_q2] = '2' and [boosterlast2week_s] = '1'	Hinchazón en el lugar de la inyección o a su alrededor	radio (Matrix)<br>0 No<br>1 Leve (notas los síntomas, pero no son un problema)<br>2 Moderado (los síntomas limitan sus actividades diarias)<br>3 Grave (los síntomas dificultan o impiden por completo sus actividades diarias)
1819	[booster_rash_s_q2]<br><br>Show the field ONLY if: [language_q2] = '2' and [boosterlast2week_s] = '1'	Erupción en el lugar de la inyección o a su alrededor	radio (Matrix)<br>0 No<br>1 Leve (notas los síntomas, pero no son un problema)<br>2 Moderado (los síntomas limitan sus actividades diarias)<br>3 Grave (los síntomas dificultan o impiden por completo sus actividades diarias)
1820	[booster_headache_s_q2]<br><br>Show the field ONLY if: [language_q2] = '2' and [boosterlast2week_s] = '1'	Dolor de cabeza	radio (Matrix)<br>0 No<br>1 Leve (notas los síntomas, pero no son un problema)<br>2 Moderado (los síntomas limitan sus actividades diarias)<br>3 Grave (los síntomas dificultan o impiden por completo sus actividades diarias)
1821	[booster_fatigue_s_q2]<br><br>Show the field ONLY if: [language_q2] = '2' and [boosterlast2week_s] = '1'	Fatiga	radio (Matrix)<br>0 No<br>1 Leve (notas los síntomas, pero no son un problema)<br>2 Moderado (los síntomas limitan sus actividades diarias)<br>3 Grave (los síntomas dificultan o impiden por completo sus actividades diarias)

1822	[booster_fever_s_q2]<br><br>Show the field ONLY if: [language_q2] = '2' and [boosterlast2week_s] = '1'	Fiebre (temperaturas sobre >100.4 °F o >38°C)	radio (Matrix)<br>0 No<br>1 Leve (notas los síntomas, pero no son un problema)<br>2 Moderado (los síntomas limitan sus actividades diarias)<br>3 Grave (los síntomas dificultan o impiden por completo sus actividades diarias)
1823	[booster_chills_s_q2]<br><br>Show the field ONLY if: [language_q2] = '2' and [boosterlast2week_s] = '1'	Escalofríos	radio (Matrix)<br>0 No<br>1 Leve (notas los síntomas, pero no son un problema)<br>2 Moderado (los síntomas limitan sus actividades diarias)<br>3 Grave (los síntomas dificultan o impiden por completo sus actividades diarias)
1824	[booster_joint_s_q2]<br><br>Show the field ONLY if: [language_q2] = '2' and [boosterlast2week_s] = '1'	Dolor en las articulaciones	radio (Matrix)<br>0 No<br>1 Leve (notas los síntomas, pero no son un problema)<br>2 Moderado (los síntomas limitan sus actividades diarias)<br>3 Grave (los síntomas dificultan o impiden por completo sus actividades diarias)
1825	[booster_muscle_s_q2]<br><br>Show the field ONLY if: [language_q2] = '2' and [boosterlast2week_s] = '1'	Dolor muscular	radio (Matrix)<br>0 No<br>1 Leve (notas los síntomas, pero no son un problema)<br>2 Moderado (los síntomas limitan sus actividades diarias)<br>3 Grave (los síntomas dificultan o impiden por completo sus actividades diarias)
1826	[booster_nausea_s_q2]<br><br>Show the field ONLY if: [language_q2] = '2' and [boosterlast2week_s] = '1'	Nausea	radio (Matrix)<br>0 No<br>1 Leve (notas los síntomas, pero no son un problema)<br>2 Moderado (los síntomas limitan sus actividades diarias)<br>3 Grave (los síntomas dificultan o impiden por completo sus actividades diarias)

1827	[receive_booster6_s_q2]<br><br>Show the field ONLY if: [booster_pain_s_q2] = '1' or [booster_pain_s_q2] = '2' or [booster_pain_s_q2] = '3' or [booster_redness_s_q2] = '1' or [booster_redness_s_q2] = '2' or [booster_redness_s_q2] = '3' or [booster_swelling_s_q2] = '1' or [booster_swelling_s_q2] = '2' or [booster_swelling_s_q2] = '3' or [booster_rash_s_q2] = '1' or [booster_rash_s_q2] = '2' or [booster_rash_s_q2] = '3' or [booster_headache_s_q2] = '1' or [booster_headache_s_q2] = '2' or [booster_headache_s_q2] = '3' or [booster_fatigue_s_q2] = '1' or [booster_fatigue_s_q2] = '2' or [booster_fatigue_s_q2] = '3' or [booster_fever_s_q2] = '1' or [booster_fever_s_q2] = '2' or [booster_fever_s_q2] = '3' or [booster_chills_s_q2] = '1' or [booster_chills_s_q2] = '2' or [booster_chills_s_q2] = '3' or [booster_joint_s_q2] = '1' or [booster_joint_s_q2] = '2' or [booster_joint_s_q2] = '3' or [booster_muscle_s_q2] = '1' or [booster_muscle_s_q2] = '2' or [booster_muscle_s_q2] = '3' or [booster_nausea_s_q2] = '1' or [booster_nausea_s_q2] = '2' or [booster_nausea_s_q2] = '3'	¿Cuánto tiempo duraron estos efectos secundarios?	radio<br>1 Menos de 12 horas<br>2 Entre 12 y 24 horas<br>3 Mas de 24 horas
1828	[receive_booster7_s_q2]<br><br>Show the field ONLY if: [booster_pain_s_q2] = '1' or [booster_pain_s_q2] = '2' or [booster_pain_s_q2] = '3' or [booster_redness_s_q2] = '1' or [booster_redness_s_q2] = '2' or [booster_red	¿Tomó alguna medicación para estos efectos secundarios?	radio<br>1 Sí<br>0 No

	ness_s_q2] = '3' or [booster_swelling_s_q2] = '1' or [booster_swelling_s_q2] = '2' or [booster_swelling_s_q2] = '3' or [booster_rash_s_q2] = '1' or [booster_rash_s_q2] = '2' or [booster_rash_s_q2] = '3' or [booster_headache_s_q2] = '1' or [booster_headache_s_q2] = '2' or [booster_headache_s_q2] = '3' or [booster_fatigue_s_q2] = '1' or [booster_fatigue_s_q2] = '2' or [booster_fatigue_s_q2] = '3' or [booster_fever_s_q2] = '1' or [booster_fever_s_q2] = '2' or [booster_fever_s_q2] = '3' or [booster_chills_s_q2] = '1' or [booster_chills_s_q2] = '2' or [booster_chills_s_q2] = '3' or [booster_joint_s_q2] = '1' or [booster_joint_s_q2] = '2' or [booster_joint_s_q2] = '3' or [booster_muscle_s_q2] = '1' or [booster_muscle_s_q2] = '2' or [booster_muscle_s_q2] = '3' or [booster_nausea_s_q2] = '1' or [booster_nausea_s_q2] = '2' or [booster_nausea_s_q2] = '3'		
1829	[receive_booster8_s_q2]<br>Show the field ONLY if: [receive_booster7_s_q2] = '1'	¿qué medicamentos tomó para estos efectos secundarios?	text
1830	[receive_booster9_s_q2]<br>Show the field ONLY if: [booster_pain_s_q2] = '1' or [booster_pain_s_q2] = '2' or [booster_pain_s_q2] = '3' or [booster_redness_s_q2] = '1' or [booster_redness_s_q2] = '2' or [booster_redness_s_q2] = '3' or [booster_swelling_s_q2] = '1' or [booster_swelling_s_q2] = '2' or [booster_swelling_s_q2] = '3' or	¿Consultó a un médico u otro profesional de salud por estos efectos secundarios?	radio<br>1 Sí<br>0 No

	[booster_rash_s_q2] = '1' or [booster_rash_s_q2] = '2' or [booster_rash_s_q2] = '3' or [booster_headache_s_q2] = '1' or [booster_headache_s_q2] = '2' or [booster_headache_s_q2] = '3' or [booster_fatigue_s_q2] = '1' or [booster_fatigue_s_q2] = '2' or [booster_fatigue_s_q2] = '3' or [booster_fever_s_q2] = '1' or [booster_fever_s_q2] = '2' or [booster_fever_s_q2] = '3' or [booster_chills_s_q2] = '1' or [booster_chills_s_q2] = '2' or [booster_chills_s_q2] = '3' or [booster_joint_s_q2] = '1' or [booster_joint_s_q2] = '2' or [booster_joint_s_q2] = '3' or [booster_muscle_s_q2] = '1' or [booster_muscle_s_q2] = '2' or [booster_muscle_s_q2] = '3' or [booster_nausea_s_q2] = '1' or [booster_nausea_s_q2] = '2' or [booster_nausea_s_q2] = '3'		
1831	[receive_booster10_s_q2]<br>Show the field ONLY if: [booster_pain_s_q2] = '1' or [booster_pain_s_q2] = '2' or [booster_pain_s_q2] = '3' or [booster_redness_s_q2] = '1' or [booster_redness_s_q2] = '2' or [booster_redness_s_q2] = '3' or [booster_swelling_s_q2] = '1' or [booster_swelling_s_q2] = '2' or [booster_swelling_s_q2] = '3' or [booster_rash_s_q2] = '1' or [booster_rash_s_q2] = '2' or [booster_rash_s_q2] = '3' or [booster_headache_s_q2] = '1' or [booster_headache_s_q2] = '2' or [booster_headache_s_q2] = '3' or [booster_fatigue_s_q2] = '1' or [booster_fatigue_s_q2] = '2' or [booster_f	¿Los efectos secundarios que sintió después de la dosis de refuerzo fueron más o menos graves en comparación con los efectos secundarios de la primera dosis de la vacuna COVID-19?	radio<br>1 Mas grave<br>2 Igual de grave<br>3 Menos grave<br>4 No experimenté ningún efecto secundario con la primera dosis

	atigue_s_q2] = '3' or [booster_fever_s_q2] = '1' or [booster_fever_s_q2] = '2' or [booster_fever_s_q2] = '3' or [booster_chills_s_q2] = '1' or [booster_chills_s_q2] = '2' or [booster_chills_s_q2] = '3' or [booster_joint_s_q2] = '1' or [booster_joint_s_q2] = '2' or [booster_joint_s_q2] = '3' or [booster_muscle_s_q2] = '1' or [booster_muscle_s_q2] = '2' or [booster_muscle_s_q2] = '3' or [booster_nausea_s_q2] = '1' or [booster_nausea_s_q2] = '2' or [booster_nausea_s_q2] = '3'		
1832	[receive_booster11_s_q2]<br><br>Show the field ONLY if: [booster_pain_s_q2] = '1' or [booster_pain_s_q2] = '2' or [booster_pain_s_q2] = '3' or [booster_redness_s_q2] = '1' or [booster_redness_s_q2] = '2' or [booster_redness_s_q2] = '3' or [booster_swelling_s_q2] = '1' or [booster_swelling_s_q2] = '2' or [booster_swelling_s_q2] = '3' or [booster_rash_s_q2] = '1' or [booster_rash_s_q2] = '2' or [booster_rash_s_q2] = '3' or [booster_headache_s_q2] = '1' or [booster_headache_s_q2] = '2' or [booster_headache_s_q2] = '3' or [booster_fatigue_s_q2] = '1' or [booster_fatigue_s_q2] = '2' or [booster_fatigue_s_q2] = '3' or [booster_fever_s_q2] = '1' or [booster_fever_s_q2] = '2' or [booster_fever_s_q2] = '3' or [booster_chills_s_q2] = '1' or [booster_chills_s_q2] = '2' or [booster_chills_s_q2] = '3' or [booster_joint_s_q2] = '1' or [booster_joint_s_q2] = '2' or [boost	¿Los efectos secundarios que sintió después de la dosis de refuerzo fueron más o menos graves en comparación con los efectos secundarios de la segunda dosis de la vacuna COVID-19?	radio<br>1 Mas grave<br>2 Igual de grave<br>3 Menos grave<br>4 No experimenté ningún efecto secundario con la segunda dosis

	er_joint_s_q2] = '3' or [booster_muscle_s_q2] = '1' or [booster_muscle_s_q2] = '2' or [booster_muscle_s_q2] = '3' or [booster_nausea_s_q2] = '1' or [booster_nausea_s_q2] = '2' or [booster_nausea_s_q2] = '3'		
1833	[address_s_q2]<br><br>Show the field ONLY if: [language_q2] = '2'	Section Header: *Proporcione la siguiente informacion sobre su hogar.*<br><br>¿Cuál es su dirección permanente?	text<br>Field Annotation: @DEFAULT="[address_s_q2]"
1834	[address2_s_q2]<br><br>Show the field ONLY if: [language_q2] = '2'	¿Cuanto tiempo ha vivido en esta dirección?	radio<br>1 | 0-3 años<br>2 | 4-6 años<br>3 | 7-10 años<br>4 | Más de 10 años<br><br>Field Annotation: @DEFAULT="[address2_s_q2]"
1835	[hhcount_s_q2]<br><br>Show the field ONLY if: [language_q2] = '2'	¿Cuántas personas (sin contarse usted) viven o pasan una cantidad considerable de tiempo en esta vivienda (más de 40 horas por semana)?	radio<br>0 | 0<br>1 | 1<br>2 | 2<br>3 | 3<br>4 | 4<br>5 | 5<br>6 | 6<br>7 | 7<br>8 | 8<br>9 | 9<br>10 | 10<br>11 | 11<br>12 | 12<br><br>Field Annotation: @DEFAULT="[hhcount_s_q2]"
1836	[hhcount2_s_q2]<br><br>Show the field ONLY if: [language_q2] = '2' and [hhcount_s_q2] > 0	¿Cuántas personas de su grupo familiar son menores de 18 años?	radio<br>0 | 0<br>1 | 1<br>2 | 2<br>3 | 3<br>4 | 4<br>5 | 5

			6 6<br>7 7<br>8 8<br>9 9<br>10 10<br>11 11<br>12 12<br><br>Field Annotation: @DEFAULT="[hhcount2_s_q2]"
1837	[hh1_relationship_s_q2]<br><br>Show the field ONLY if: [language_q2] = '2' and [hhcount_s_q2] > 0 and [hhcount_s_q2] < 13	Section Header: *Por cada persona adicional en su hogar, proporcione la siguiente informacion.*<br><br>Persona 1: ¿Cuál es su relación con esta persona?	radio<br>1 Pareja o cónyuge<br>2 Hijo o hija<br>3 Padre o madre<br>4 Hermano o hermana<br>5 Otro familiar<br>6 Proveedor de cuidado infantil u otros cuidados en casa<br>7 Otra<br><br>Field Annotation: @DEFAULT="[hh1_relationship_s_q2]"
1838	[hh1_relationship2_s_q2]<br><br>Show the field ONLY if: [language_q2] = '2' and [hh1_relationship_s_q2] = '7'	Persona 1: Especifique su relación con esta persona.	text<br>Field Annotation: @DEFAULT="[hh1_relationship2_s_q2]"
1839	[hh1_age_s_q2]<br><br>Show the field ONLY if: [language_q2] = '2' and [hhcount_s_q2] > 0 and [hhcount_s_q2] < 13	Persona 1: ¿Qué edad tiene esta persona?<br>*(Especifique la edad en años)*	text (number, Min: 0, Max: 110)<br>Field Annotation: @DEFAULT="[hh1_age_s_q2]"
1840	[hh1_s_q2ex_s_q2]<br><br>Show the field ONLY if: [language_q2] = '2' and [hhcount_s_q2] > 0 and [hhcount_s_q2] < 13	Persona 1: ¿Cuál es el sexo de esta persona?	radio<br>1 Femenino<br>2 Masculino<br><br>Field Annotation: @DEFAULT="[hh1_s_q2ex_s_q2]"
1841	[hh1_race_s_q2]<br><br>Show the field ONLY if: [language_q2] = '2' and [hhcount_s_q2] > 0 and [hhcount_s_q2] < 13	Persona 1: ¿Cuál es la raza de esta persona?<br>*(Seleccione todo lo que corresponda.)*	checkbox<br>1 hh1_race_s_q2___1 Indio americano o nativo de Alaska<br>2 hh1_race_s_q2___2 Asiático<br>3 hh1_race_s_q2___3 Negro o afroestadounidense

			4 | hh1_race_s_q2___4 | Nativo de Hawái o de las islas del Pacífico<br>5 | hh1_race_s_q2___5 | Blanco<br>6 | hh1_race_s_q2___6 | Otra<br>7 | hh1_race_s_q2___7 | No sabe<br><br>Field Annotation: @DEFAULT=" [hh1_race_s_q2]"
1842	[hh1_ethn_s_q2]<br><br>Show the field ONLY if: [language_q2] = '2' and [hhcount_s_q2] > 0 and [hhcount_s_q2] < 13	Persona 1: ¿Cuál es la identidad étnica de esta persona?	radio<br>1 | Hispano o latino<br>2 | Ni hispano ni latino<br>3 | Otra<br>4 | No sabe<br><br>Field Annotation: @DEFAULT=" [hh1_ethn_s_q2]"
1843	[hh1_edu_s_q2]<br><br>Show the field ONLY if: [language_q2] = '2' and [hhcount_s_q2] > 0 and [hhcount_s_q2] < 13	Persona 1: ¿Cuál es el nivel de educación de esta persona?	radio<br>1 | No tiene educación formal<br>2 | Kínder a 8° grado<br>3 | Estudios de educación secundaria<br>4 | Equivalencia de educación secundaria (GED)<br>5 | Diploma de educación secundaria<br>6 | Estudios de educación superior<br>7 | Título universitario<br>8 | Estudios de posgrado o más<br>9 | No sabe<br><br>Field Annotation: @DEFAULT=" [hh1_edu_s_q2]"
1844	[hh1_work_s_q2]<br><br>Show the field ONLY if: [language_q2] = '2' and [hhcount_s_q2] > 0 and [hhcount_s_q2] < 13	Persona 1: ¿Cuál de las siguientes es la mejor descripción de la situación laboral actual de esta persona?	radio<br>1 | Empleo a tiempo completo<br>2 | Empleo a tiempo parcial<br>3 | En busca de empleo<br>4 | Jubilado<br>5 | Ama de casa<br>6 | Estudiante<br>7 | Permiso de maternidad o paternidad<br>8 | Permiso por enfermedad<br>9 | Desempleado por discapacidad<br>10 | Otra

			11 No sabe<br><br>Field Annotation: @DEFAULT="[hh1_work_s_q2]"
1845	[hh1_work2_s_q2]<br><br>Show the field ONLY if: [language_q2] = '2' and [hhcount_s_q2] > 0 and [hhcount_s_q2] < 13	Persona 1: ¿Se considera esta persona actualmente empleado por cuenta propia (contratista independiente, trabajador esporádico (gig), etc.)?	radio<br>1 Sí<br>0 No<br>2 No sabe<br><br>Field Annotation: @DEFAULT="[hh1_work2_s_q2]"
1846	[hh1_work3_s_q2]<br><br>Show the field ONLY if: [language_q2] = '2' and [hhcount_s_q2] > 0 and [hhcount_s_q2] < 13 and ([hh1_work_s_q2] = '1' or [hh1_work_s_q2] = '2' or [hh1_work2_s_q2] = '1')	Persona 1: ¿Trabaja esta persona actualmente en alguno de los siguientes ambientes de alto riesgo de contagio de COVID-19?	checkbox<br>1 hh1_work3_s_q2___1 Ambiente de asistencia médica (hospital, clínica, centro de urgencias, etc.)<br>2 hh1_work3_s_q2___2 Ambiente residencial denso (hogar de ancianos, otro centro de asistencia de larga duración)<br>3 hh1_work3_s_q2___3 Prisión o cárcel<br>4 hh1_work3_s_q2___4 Establecimiento de envasado de carne<br>5 hh1_work3_s_q2___5 Establecimiento de envío o distribución<br>6 hh1_work3_s_q2___6 Establecimiento minorista de alto volumen (tienda de provisiones, etc.)<br>7 hh1_work3_s_q2___7 No sabe<br><br>Field Annotation: @DEFAULT="[hh1_work3_s_q2]"
1847	[hh1_work4_s_q2]<br><br>Show the field ONLY if: [language_q2] = '2' and [hhcount_s_q2] > 0 and [hhcount_s_q2] < 13 and ([hh1_work_s_q2] = '1' or [hh1_work_s_q2]	Persona 1: ¿Le ofrece el empleador a esta persona alguno de los siguientes beneficios en su empleo principal actual?<br>*(Seleccione todo lo que corresponda.)*	checkbox<br>1 hh1_work4_s_q2___1 Permiso por enfermedad con goce de sueldo<br>2 hh1_work4_s_q2___2 Vacaciones o permiso

<table>
<tr><td></td><td>= '2' or [hh1_work2_s_q2] = '1')</td><td></td><td><table><tr><td></td><td></td><td>personal con goce de sueldo</td></tr><tr><td>3</td><td>hh1_work4_s_q2___3</td><td>Seguro de salud</td></tr><tr><td>4</td><td>hh1_work4_s_q2___4</td><td>Seguro de discapacidad</td></tr><tr><td>5</td><td>hh1_work4_s_q2___5</td><td>Plan de jubilación</td></tr><tr><td>6</td><td>hh1_work4_s_q2___6</td><td>Otro</td></tr><tr><td>7</td><td>hh1_work4_s_q2___7</td><td>No sabe</td></tr></table>Field Annotation: @DEFAULT="[hh1_work4_s_q2]"</td></tr>
<tr><td>1848</td><td>[hh1_work5_s_q2]

Show the field ONLY if: [language_q2] = '2' and [hhcount_s_q2] > 0 and [hhcount_s_q2] < 13 and ([hh1_work_s_q2] = '1' or [hh1_work_s_q2] = '2' or [hh1_work2_s_q2] = '1')</td><td>Persona 1: En una escala de 0 (definitivamente no va a suceder) a 10 (definitivamente va a suceder), ¿qué tan probable es que esta persona pierda su empleo debido a la pandemia de COVID-19?</td><td>text (number, Min: 0, Max: 10)
Field Annotation: @DEFAULT="[hh1_work5_s_q2]"</td></tr>
<tr><td>1849</td><td>[hh1_work6_s_q2]

Show the field ONLY if: [language_q2] = '2' and [hhcount_s_q2] > 0 and [hhcount_s_q2] < 13 and ([hh1_work_s_q2] = '1' or [hh1_work_s_q2] = '2' or [hh1_work2_s_q2] = '1')</td><td>Persona 1: En una escala de 0 (definitivamente no va a suceder) a 10 (definitivamente va a suceder), ¿qué tan probable es que a esta persona se le asignen menos horas de trabajo debido a la pandemia de COVID-19?</td><td>text (number, Min: 0, Max: 10)
Field Annotation: @DEFAULT="[hh1_work6_s_q2]"</td></tr>
<tr><td>1850</td><td>[hh1_work7_s_q2]

Show the field ONLY if: [language_q2] = '2' and [hhcount_s_q2] > 0 and [hhcount_s_q2] < 13 and ([hh1_work_s_q2] = '1' or [hh1_work_s_q2] = '2' or [hh1_work2_s_q2] = '1')</td><td>Persona 1: Actualmente ¿con qué frecuencia se le exige a esta persona que trabaje fuera de su residencia?</td><td>radio (Matrix)<table><tr><td>1</td><td>Todo el tiempo (100%)</td></tr><tr><td>2</td><td>La mayor parte del tiempo (75%)</td></tr><tr><td>3</td><td>La mitad del tiempo (50%)</td></tr><tr><td>4</td><td>Menos de la mitad del tiempo (25%)</td></tr><tr><td>5</td><td>nunca (0%)</td></tr><tr><td>6</td><td>Nunca</td></tr></table></td></tr>
<tr><td>1851</td><td>[hh1_work8_s_q2]

Show the field ONLY if: [language_q2] = '2' and ([hh1_work7_s_q2] = '1' or [hh1_work7_s_q2] = '2' or [hh1_work7_s_q2] = '3' or [hh1_work7_s_q2] = '4')</td><td>Persona 1: Actualmente, ¿con qué frecuencia se encuentra esta persona físicamente cerca de sus compañeros de trabajo mientras trabaja fuera de su residencia?</td><td>radio (Matrix)<table><tr><td>1</td><td>Todo el tiempo (100%)</td></tr><tr><td>2</td><td>La mayor parte del tiempo (75%)</td></tr><tr><td>3</td><td>La mitad del tiempo (50%)</td></tr><tr><td>4</td><td>Menos de la mitad del tiempo (25%)</td></tr><tr><td>5</td><td>nunca (0%)</td></tr><tr><td>6</td><td>Nunca</td></tr></table></td></tr>
</table>

1852	[hh1_work9_s_q2]<br><br>Show the field ONLY if: [language_q2] = '2' and ([hh1_work7_s_q2] = '1' or [hh1_work7_s_q2] = '2' or [hh1_work7_s_q2] = '3' or [hh1_work7_s_q2] = '4')	Persona 1: Actualmente, ¿con qué frecuencia se encuentra esta persona físicamente cerca de sus clientes mientras trabaja fuera de su residencia	radio (Matrix)<br>1 Todo el tiempo (100%)<br>2 La mayor parte del tiempo (75%)<br>3 La mitad del tiempo (50%)<br>4 Menos de la mitad del tiempo (25%)<br>5 nunca (0%)<br>6 Nunca
1853	[hh1_covidvaccine_s_q2]<br><br>Show the field ONLY if: [language_q2] = '2' and [hhcount_s_q2] > 0 and [hhcount_s_q2] < 13	Persona 1: ¿Piensa esta persona vacunarse contra la COVID-19?	radio<br>1 Sí<br>0 No<br>2 No sabe<br>3 Este individuo ya ha sido vacunado<br><br>Field Annotation: @DEFAULT=" [hh1_covidvaccine_s_q2]"
1854	[hh1_covidsymp_s_q2]<br><br>Show the field ONLY if: [language_q2] = '2' and [hhcount_s_q2] > 0 and [hhcount_s_q2] < 13	Persona 1: En las últimas dos semanas, ¿ha tenido esta persona algún síntoma de COVID-19 (tos, fiebre, dificultad para respirar, fatiga, dolores de cuerpo, diarrea, goteo nasal o pérdida del sentido del olfato o del gusto)?	radio<br>1 Sí<br>0 No<br>2 No sabe
1855	[hh1_covidsymp2_s_q2]<br><br>Show the field ONLY if: [language_q2] = '2' and [hh1_covidsymp_s_q2] = '1'	Persona 1: ¿Cuándo le comenzaron los síntomas de COVID-19 a esta persona?	text (date_mdy)
1856	[hh1_covidsymp3_s_q2]<br><br>Show the field ONLY if: [language_q2] = '2' and [hh1_covidsymp_s_q2] = '1'	Persona 1: En vista de sus síntomas, ¿le preocupa a esta persona la posibilidad de tener COVID-19?	radio<br>1 Sí<br>0 No<br>2 No sabe
1857	[hh1_covidsymp4_s_q2]<br><br>Show the field ONLY if: [language_q2] = '2' and [hh1_covidsymp_s_q2] = '1'	Persona 1: ¿Fue esta persona objeto de prejuicio o discriminación debido a sus síntomas?	radio<br>1 Sí<br>0 No<br>2 No sabe
1858	[hh1_covidsymp5_s_q2]<br><br>Show the field ONLY if: [language_q2] = '2' and [hh1_covidsymp_s_q2] = '1'	Persona 1: ¿Qué hizo esta persona en vista de sus síntomas?<br>*(Seleccione todo lo que corresponda.)*	checkbox<br>0 hh1_covidsymp5_s_q2___0 Nada<br>1 hh1_covidsymp5_s_q2___1 Tomó medicament sin receta (ibuprofeno, acetaminofé etc.)

			2 | hh1_covidsymp5_s_q2___2 | Consultó por teléfono a un proveedor de asistencia médica<br>3 | hh1_covidsymp5_s_q2___3 | Fue al consultorio d un proveedo de asistencia médica<br>4 | hh1_covidsymp5_s_q2___4 | Fue a una clínica o una farmacia minorista<br>5 | hh1_covidsymp5_s_q2___5 | Fue a un centro de urgencias (FASTMed, etc.)<br>6 | hh1_covidsymp5_s_q2___6 | Fue a la sala de emergencias<br>7 | hh1_covidsymp5_s_q2___7 | Fue ingresad al hospital<br>8 | hh1_covidsymp5_s_q2___8 | Otra cosa<br>9 | hh1_covidsymp5_s_q2___9 | No sabe
1859	[hh1_covidsymp6_s_q2]<br>Show the field ONLY if: [language_q2] = '2' and [hh1_covidsymp5_s_q2(8)] = '1'	Persona 1: ¿Qué otra cosa hizo esta persona en vista de sus síntomas?	text
1860	[hh1_covidsymp7_s_q2]<br>Show the field ONLY if: [language_q2] = '2' and ([hh1_covidsymp5_s_q2(2)] = '1' or [hh1_covidsymp5_s_q2(3)] = '1' or [hh1_covidsymp5_s_q2(4)] = '1' or [hh1_covidsymp5_s_q2(5)] = '1' or [hh1_covidsymp5_s_q2(6)] = '1' or [hh1_covidsymp5_s_q2(7)] = '1' or [hh1_covidsymp5_s_q2(8)] = '1')	Persona 1: ¿Le dijo un proveedor de asistencia médica a esta persona que era posible que tuviera COVID-19?	radio<br>1 | Sí<br>0 | No<br>2 | No sabe
1861	[hh1_covid_test_s_q2]<br>Show the field ONLY if:	Persona 1: Si a esta persona le hicieron una prueba de COVID-19 en vista de sus síntomas, ¿cuál fue el resultado?	radio<br>1 | Pendiente<br>2 | Positivo

	[language_q2] = '2' and [hh1_covidsymp_s_q2] = '1'		3 Negativo<br>4 No concluyente<br>5 No se hizo la prueba<br>6 No sabe
1862	[hh1_covid_admit_s_q2]<br>Show the field ONLY if: [language_q2] = '2' and [hh1_covidsymp5_s_q2(7)] = '1'	Persona 1: ¿Cuántos días estuvo hospitalizada esta persona?	text (number, Min: 0)
1863	[hh1_covid_admit2_s_q2]<br>Show the field ONLY if: [language_q2] = '2' and [hh1_covidsymp5_s_q2(7)] = '1'	Persona 1: ¿Se le hicieron a esta persona las siguientes intervenciones durante su hospitalización?	checkbox<br>1 hh1_covid_admit2_s_q2___1 Oxígeno adicional por la nariz<br>2 hh1_covid_admit2_s_q2___2 Tratamiento en la Unidad de Cuidados Intensivos (Intensive Care Unit, ICU)<br>3 hh1_covid_admit2_s_q2___3 Ventilación mecánica (intubación o tubo de respiración)<br>4 hh1_covid_admit2_s_q2___4 No sabe
1864	[hh1_covidsymp8_s_q2]<br>Show the field ONLY if: [language_q2] = '2' and [hh1_covidsymp_s_q2] = '1'	Persona 1: ¿Ha vuelto esta persona a su salud normal?	radio<br>1 Sí<br>0 No<br>2 No sabe
1865	[hh1_prevent_s_q2]<br>Show the field ONLY if: [language_q2] = '2' and [hh1_covidsymp_s_q2] = '1'	Persona 1: ¿Cuáles de las siguientes medidas tomó esta persona para proteger a sus amigos y familiares después de que comenzaron sus síntomas?	checkbox<br>1 hh1_prevent_s_q2___1 Ponerse mascarilla con mayor frecuencia<br>2 hh1_prevent_s_q2___2 Lavarse las manos con agua y jabón con mayor frecuencia<br>3 hh1_prevent_s_q2___3 Limpiarse las manos con desinfectante con mayor frecuencia

			4 | hh1_prevent_s_q2___4 | Aislarse en su casa con mayor frecuencia<br>5 | hh1_prevent_s_q2___5 | Quedarse en casa con mayor frecuencia<br>6 | hh1_prevent_s_q2___6 | Ponerse guantes desechables con mayor frecuencia<br>7 | hh1_prevent_s_q2___7 | No sabe
1866	[hh2_relationship_s_q2]<br><br>Show the field ONLY if: [language_q2] = '2' and [hhcount_s_q2] > 1 and [hhcount_s_q2] < 13	Section Header: *Por cada persona adicional en su hogar, proporcione la siguiente informacion.*<br><br>Persona 2: ¿Cuál es su relación con esta persona?	radio<br>1 | Pareja o cónyuge<br>2 | Hijo o hija<br>3 | Padre o madre<br>4 | Hermano o hermana<br>5 | Otro familiar<br>6 | Proveedor de cuidado infantil u otros cuidados en casa<br>7 | Otra<br><br>Field Annotation: @DEFAULT="[hh2_relationship_s_q2]"
1867	[hh2_relationship2_s_q2]<br><br>Show the field ONLY if: [language_q2] = '2' and [hh2_relationship_s_q2] = '7'	Persona 2: Especifique su relación con esta persona.	text<br>Field Annotation: @DEFAULT="[hh2_relationship2_s_q2]"
1868	[hh2_age_s_q2]<br><br>Show the field ONLY if: [language_q2] = '2' and [hhcount_s_q2] > 1 and [hhcount_s_q2] < 13	Persona 2: ¿Qué edad tiene esta persona? *(Especifique la edad en años)*	text (number, Min: 0, Max: 110)<br>Field Annotation: @DEFAULT="[hh2_age_s_q2]"
1869	[hh2_s_q2ex_s_q2]<br><br>Show the field ONLY if: [language_q2] = '2' and [hhcount_s_q2] > 1 and [hhcount_s_q2] < 13	Persona 2: ¿Cuál es el sexo de esta persona?	radio<br>1 | Femenino<br>2 | Masculino<br><br>Field Annotation: @DEFAULT="[hh2_s_q2ex_s_q2]"
1870	[hh2_race_s_q2]<br><br>Show the field ONLY if: [language_q2] = '2' and [hhcount_s_q2] > 1 and	Persona 2: ¿Cuál es la raza de esta persona? *(Seleccione todo lo que corresponda.)*	checkbox<br>1 | hh2_race_s_q2___1 | Indio americano o nativo de Alaska<br>2 | hh2_race_s_q2___2 | Asiático

	[hhcount_s_q2] < 13		3 hh2_race_s_q2___3 Negro o afroestadounidense<br>4 hh2_race_s_q2___4 Nativo de Hawái o de las islas del Pacífico<br>5 hh2_race_s_q2___5 Blanco<br>6 hh2_race_s_q2___6 Otra<br>7 hh2_race_s_q2___7 No sabe<br><br>Field Annotation: @DEFAULT="[hh2_race_s_q2]"
1871	[hh2_ethn_s_q2]<br><br>Show the field ONLY if: [language_q2] = '2' and [hhcount_s_q2] > 1 and [hhcount_s_q2] < 13	Persona 2: ¿Cuál es la identidad étnica de esta persona?	radio<br>1 Hispano o latino<br>2 Ni hispano ni latino<br>3 Otra<br>4 No sabe<br><br>Field Annotation: @DEFAULT="[hh2_ethn_s_q2]"
1872	[hh2_edu_s_q2]<br><br>Show the field ONLY if: [language_q2] = '2' and [hhcount_s_q2] > 1 and [hhcount_s_q2] < 13	Persona 2: ¿Cuál es el nivel de educación de esta persona?	radio<br>1 No tiene educación formal<br>2 Kínder a 8° grado<br>3 Estudios de educación secundaria<br>4 Equivalencia de educación secundaria (GED)<br>5 Diploma de educación secundaria<br>6 Estudios de educación superior<br>7 Título universitario<br>8 Estudios de posgrado o más<br>9 No sabe<br><br>Field Annotation: @DEFAULT="[hh2_edu_s_q2]"
1873	[hh2_work_s_q2]<br><br>Show the field ONLY if: [language_q2] = '2' and [hhcount_s_q2] > 1 and [hhcount_s_q2] < 13	Persona 2: ¿Cuál de las siguientes es la mejor descripción de la situación laboral actual de esta persona?	radio<br>1 Empleo a tiempo completo<br>2 Empleo a tiempo parcial<br>3 En busca de empleo<br>4 Jubilado<br>5 Ama de casa<br>6 Estudiante<br>7 Permiso de maternidad o paternidad<br>8 Permiso por enfermedad

			9 Desempleado por discapacidad<br>10 Otra<br>11 No sabe<br><br>Field Annotation: @DEFAULT="[hh2_work_s_q2]"
1874	[hh2_work2_s_q2]<br><br>Show the field ONLY if: [language_q2] = '2' and [hhcount_s_q2] > 1 and [hhcount_s_q2] < 13	Persona 2: ¿Se considera esta persona actualmente empleado por cuenta propia (contratista independiente, trabajador esporádico (gig), etc.)?	radio<br>1 Sí<br>0 No<br>2 No sabe<br><br>Field Annotation: @DEFAULT="[hh2_work2_s_q2]"
1875	[hh2_work3_s_q2]<br><br>Show the field ONLY if: [language_q2] = '2' and [hhcount_s_q2] > 1 and [hhcount_s_q2] < 13 and ([hh2_work_s_q2] = '1' or [hh2_work_s_q2] = '2' or [hh2_work2_s_q2] = '1')	Persona 2: ¿Trabaja esta persona actualmente en alguno de los siguientes ambientes de alto riesgo de contagio de COVID-19?	checkbox<br>1 hh2_work3_s_q2___1 Ambiente de asistencia médica (hospital, clínica, centro de urgencias, etc.)<br>2 hh2_work3_s_q2___2 Ambiente residencial denso (hogar de ancianos, otro centro de asistencia de larga duración)<br>3 hh2_work3_s_q2___3 Prisión o cárcel<br>4 hh2_work3_s_q2___4 Establecimiento de envasado de carne<br>5 hh2_work3_s_q2___5 Establecimiento de envío o distribución<br>6 hh2_work3_s_q2___6 Establecimiento minorista de alto volumen (tienda de provisiones, etc.)<br>7 hh2_work3_s_q2___7 No sabe<br><br>Field Annotation: @DEFAULT="[hh2_work3_s_q2]"
1876	[hh2_work4_s_q2]<br><br>Show the field ONLY if: [language_q2] = '2' and [hhcount_s_q2] > 1 and	Persona 2: ¿Le ofrece el empleador a esta persona alguno de los siguientes beneficios en su empleo principal actual?<br>*(Seleccione todo lo que corresponda.)*	checkbox

	[hhcount_s_q2] < 13 and ([hh2_work_s_q2] = '1' or [hh2_work_s_q2] = '2' or [hh2_work2_s_q2] = '1')		1 hh2_work4_s_q2___1 Permiso por enfermedad con goce de sueldo<br>2 hh2_work4_s_q2___2 Vacaciones o permiso personal con goce de sueldo<br>3 hh2_work4_s_q2___3 Seguro de salud<br>4 hh2_work4_s_q2___4 Seguro de discapacidad<br>5 hh2_work4_s_q2___5 Plan de jubilación<br>6 hh2_work4_s_q2___6 Otro<br>7 hh2_work4_s_q2___7 No sabe<br><br>Field Annotation: @DEFAULT="[hh2_work4_s_q2]"
1877	[hh2_work5_s_q2]<br><br>Show the field ONLY if: [language_q2] = '2' and [hhcount_s_q2] > 1 and [hhcount_s_q2] < 13 and ([hh2_work_s_q2] = '1' or [hh2_work_s_q2] = '2' or [hh2_work2_s_q2] = '1')	Persona 2: En una escala de 0 (definitivamente no va a suceder) a 10 (definitivamente va a suceder), ¿qué tan probable es que esta persona pierda su empleo debido a la pandemia de COVID-19?	text (number, Min: 0, Max: 10)<br>Field Annotation: @DEFAULT="[hh2_work5_s_q2]"
1878	[hh2_work6_s_q2]<br><br>Show the field ONLY if: [language_q2] = '2' and [hhcount_s_q2] > 1 and [hhcount_s_q2] < 13 and ([hh2_work_s_q2] = '1' or [hh2_work_s_q2] = '2' or [hh2_work2_s_q2] = '1')	Persona 2: En una escala de 0 (definitivamente no va a suceder) a 10 (definitivamente va a suceder), ¿qué tan probable es que a esta persona se le asignen menos horas de trabajo debido a la pandemia de COVID-19?	text (number, Min: 0, Max: 10)<br>Field Annotation: @DEFAULT="[hh2_work6_s_q2]"
1879	[hh2_work7_s_q2]<br><br>Show the field ONLY if: [language_q2] = '2' and [hhcount_s_q2] > 1 and [hhcount_s_q2] < 13 and ([hh2_work_s_q2] = '1' or [hh2_work_s_q2] = '2' or [hh2_work2_s_q2] = '1')	Persona 2: Actualmente ¿con qué frecuencia se le exige a esta persona que trabaje fuera de su residencia?	radio (Matrix)<br>1 Todo el tiempo (100%)<br>2 La mayor parte del tiempo (75%)<br>3 La mitad del tiempo (50%)<br>4 Menos de la mitad del tiempo (25%)<br>5 nunca (0%)<br>6 Nunca
1880	[hh2_work8_s_q2]<br><br>Show the field ONLY if: [language_q2] = '2' and ([hh2_work7_s_q2] = '1'	Persona 2: Actualmente, ¿con qué frecuencia se encuentra esta persona físicamente cerca de sus compañeros de trabajo mientras trabaja fuera de su residencia?	radio (Matrix)<br>1 Todo el tiempo (100%)<br>2 La mayor parte del tiempo (75%)

	or [hh2_work7_s_q2] = '2' or [hh2_work7_s_q2] = '3' or [hh2_work7_s_q2] = '4')		3 La mitad del tiempo (50%)<br>4 Menos de la mitad del tiempo (25%)<br>5 nunca (0%)<br>6 Nunca
1881	[hh2_work9_s_q2]<br><br>Show the field ONLY if: [language_q2] = '2' and ([hh2_work7_s_q2] = '1' or [hh2_work7_s_q2] = '2' or [hh2_work7_s_q2] = '3' or [hh2_work7_s_q2] = '4')	Persona 2: Actualmente, ¿con qué frecuencia se encuentra esta persona físicamente cerca de sus clientes mientras trabaja fuera de su residencia	radio (Matrix)<br>1 Todo el tiempo (100%)<br>2 La mayor parte del tiempo (75%)<br>3 La mitad del tiempo (50%)<br>4 Menos de la mitad del tiempo (25%)<br>5 nunca (0%)<br>6 Nunca
1882	[hh2_covidvaccine_s_q2]<br><br>Show the field ONLY if: [language_q2] = '2' and [hhcount_s_q2] > 1 and [hhcount_s_q2] < 13	Persona 2: ¿Piensa esta persona vacunarse contra la COVID-19?	radio<br>1 Sí<br>0 No<br>2 No sabe<br>3 Este individuo ya ha sido vacunado<br><br>Field Annotation: @DEFAULT=" [hh2_covidvaccine_s_q2]"
1883	[hh2_covidsymp_s_q2]<br><br>Show the field ONLY if: [language_q2] = '2' and [hhcount_s_q2] > 1 and [hhcount_s_q2] < 13	Persona 2: En las últimas dos semanas, ¿ha tenido esta persona algún síntoma de COVID-19 (tos, fiebre, dificultad para respirar, fatiga, dolores de cuerpo, diarrea, goteo nasal o pérdida del sentido del olfato o del gusto)?	radio<br>1 Sí<br>0 No<br>2 No sabe
1884	[hh2_covidsymp2_s_q2]<br><br>Show the field ONLY if: [language_q2] = '2' and [hh2_covidsymp_s_q2] = '1'	Persona 2: ¿Cuándo le comenzaron los síntomas de COVID-19 a esta persona?	text (date_mdy)
1885	[hh2_covidsymp3_s_q2]<br><br>Show the field ONLY if: [language_q2] = '2' and [hh2_covidsymp_s_q2] = '1'	Persona 2: En vista de sus síntomas, ¿le preocupa a esta persona la posibilidad de tener COVID-19?	radio<br>1 Sí<br>0 No<br>2 No sabe
1886	[hh2_covidsymp4_s_q2]<br><br>Show the field ONLY if: [language_q2] = '2' and [hh2_covidsymp_s_q2] = '1'	Persona 2: ¿Fue esta persona objeto de prejuicio o discriminación debido a sus síntomas?	radio<br>1 Sí<br>0 No<br>2 No sabe
1887	[hh2_covidsymp5_s_q2]	Persona 2: ¿Qué hizo esta persona en vista de sus síntomas?	checkbox<br>0 hh2_covidsymp5_s_q2___0 Nada

	Show the field ONLY if:<br>[language_q2] = '2' and [hh2_covidsymp_s_q2] = '1'	*(Seleccione todo lo que corresponda.)*	1 | hh2_covidsymp5_s_q2___1 | Tomó medicament sin receta (ibuprofeno, acetaminofé etc.)<br>2 | hh2_covidsymp5_s_q2___2 | Consultó por teléfono a un proveedor de asistencia médica<br>3 | hh2_covidsymp5_s_q2___3 | Fue al consultorio c un proveedo de asistencia médica<br>4 | hh2_covidsymp5_s_q2___4 | Fue a una clínica o una farmacia minorista<br>5 | hh2_covidsymp5_s_q2___5 | Fue a un centro de urgencias (FASTMed, etc.)<br>6 | hh2_covidsymp5_s_q2___6 | Fue a la sala de emergencias<br>7 | hh2_covidsymp5_s_q2___7 | Fue ingresad al hospital<br>8 | hh2_covidsymp5_s_q2___8 | Otra cosa<br>9 | hh2_covidsymp5_s_q2___9 | No sabe
1888	[hh2_covidsymp6_s_q2]<br>Show the field ONLY if:<br>[language_q2] = '2' and [hh2_covidsymp5_s_q2(8)] = '1'	Persona 2: ¿Qué otra cosa hizo esta persona en vista de sus síntomas?	text
1889	[hh2_covidsymp7_s_q2]<br>Show the field ONLY if:<br>[language_q2] = '2' and ([hh2_covidsymp5_s_q2(2)] = '1' or [hh2_covidsymp5_s_q2(3)] = '1' or [hh2_covidsymp5_s_q2(4)] = '1' or [hh2_covidsymp5_s_q2(5)] = '1' or [hh2_covidsymp5_s_q2(6)] = '1' or [hh2_covidsymp5_s_q2(7)] = '1' or	Persona 2: ¿Le dijo un proveedor de asistencia médica a esta persona que era posible que tuviera COVID-19?	radio<br>1 | Sí<br>0 | No<br>2 | No sabe

	[hh2_covidsymp5_s_q2 (8)] = '1')		
1890	[hh2_covid_test_s_q2]<br><br>Show the field ONLY if: [language_q2] = '2' and [hh2_covidsymp_s_q2] = '1'	Persona 2: Si a esta persona le hicieron una prueba de COVID-19 en vista de sus síntomas, ¿cuál fue el resultado?	radio<br>1 Pendiente<br>2 Positivo<br>3 Negativo<br>4 No concluyente<br>5 No se hizo la prueba<br>6 No sabe
1891	[hh2_covid_admit_s_q2]<br><br>Show the field ONLY if: [language_q2] = '2' and [hh2_covidsymp5_s_q2 (7)] = '1'	Persona 2: ¿Cuántos días estuvo hospitalizada esta persona?	text (number, Min: 0)
1892	[hh2_covid_admit2_s_q2]<br><br>Show the field ONLY if: [language_q2] = '2' and [hh2_covidsymp5_s_q2 (7)] = '1'	Persona 2: ¿Se le hicieron a esta persona las siguientes intervenciones durante su hospitalización?	checkbox<br>1 hh2_covid_admit2_s_q2___1 Oxígeno adicional por la nariz<br>2 hh2_covid_admit2_s_q2___2 Tratamiento en la Unidad de Cuidados Intensivos (Intensive Care Unit, ICU)<br>3 hh2_covid_admit2_s_q2___3 Ventilación mecánica (intubación o tubo de respiración)<br>4 hh2_covid_admit2_s_q2___4 No sabe
1893	[hh2_covidsymp8_s_q2]<br><br>Show the field ONLY if: [language_q2] = '2' and [hh2_covidsymp_s_q2] = '1'	Persona 2: ¿Ha vuelto esta persona a su salud normal?	radio<br>1 Sí<br>0 No<br>2 No sabe
1894	[hh2_prevent_s_q2]<br><br>Show the field ONLY if: [language_q2] = '2' and [hh2_covidsymp_s_q2] = '1'	Persona 2: ¿Cuáles de las siguientes medidas tomó esta persona para proteger a sus amigos y familiares después de que comenzaron sus síntomas?	checkbox<br>1 hh2_prevent_s_q2___1 Ponerse mascarilla con mayor frecuencia

			2 | hh2_prevent_s_q2___2 | Lavarse las manos con agua y jabón con mayor frecuencia<br>3 | hh2_prevent_s_q2___3 | Limpiarse las manos con desinfectante con mayor frecuencia<br>4 | hh2_prevent_s_q2___4 | Aislarse en su casa con mayor frecuencia<br>5 | hh2_prevent_s_q2___5 | Quedarse en casa con mayor frecuencia<br>6 | hh2_prevent_s_q2___6 | Ponerse guantes desechables con mayor frecuencia<br>7 | hh2_prevent_s_q2___7 | No sabe
1895	[hh3_relationship_s_q2]<br>Show the field ONLY if:<br>[language_q2] = '2' and [hhcount_s_q2] > 2 and [hhcount_s_q2] < 13	Section Header: *Por cada persona adicional en su hogar, proporcione la siguiente informacion.*<br>Persona 3: ¿Cuál es su relación con esta persona?	radio<br>1 | Pareja o cónyuge<br>2 | Hijo o hija<br>3 | Padre o madre<br>4 | Hermano o hermana<br>5 | Otro familiar<br>6 | Proveedor de cuidado infantil u otros cuidados en casa<br>7 | Otra<br><br>Field Annotation: @DEFAULT="[hh3_relationship_s_q2]"
1896	[hh3_relationship2_s_q2]<br>Show the field ONLY if:<br>[language_q2] = '2' and [hh3_relationship_s_q2] = '7'	Persona 3: Especifique su relación con esta persona.	text<br>Field Annotation: @DEFAULT="[hh3_relationship2_s_q2]"
1897	[hh3_age_s_q2]<br>Show the field ONLY if:<br>[language_q2] = '2' and [hhcount_s_q2] > 2 and [hhcount_s_q2] < 13	Persona 3: ¿Qué edad tiene esta persona?<br>*(Especifique la edad en años)*	text (number, Min: 0, Max: 110)<br>Field Annotation: @DEFAULT="[hh3_age_s_q2]"
1898	[hh3_s_q2ex_s_q2]	Persona 3: ¿Cuál es el sexo de esta persona?	radio

	Show the field ONLY if: [language_q2] = '2' and [hhcount_s_q2] > 2 and [hhcount_s_q2] < 13		1 Femenino<br>2 Masculino<br><br>Field Annotation: @DEFAULT="[hh3_s_q2ex_s_q2]"
1899	[hh3_race_s_q2]<br><br>Show the field ONLY if: [language_q2] = '2' and [hhcount_s_q2] > 2 and [hhcount_s_q2] < 13	Persona 3: ¿Cuál es la raza de esta persona? *(Seleccione todo lo que corresponda.)*	checkbox<br>1 hh3_race_s_q2___1 Indio americano o nativo de Alaska<br>2 hh3_race_s_q2___2 Asiático<br>3 hh3_race_s_q2___3 Negro o afroestadounidense<br>4 hh3_race_s_q2___4 Nativo de Hawái o de las islas del Pacífico<br>5 hh3_race_s_q2___5 Blanco<br>6 hh3_race_s_q2___6 Otra<br>7 hh3_race_s_q2___7 No sabe<br><br>Field Annotation: @DEFAULT="[hh3_race_s_q2]"
1900	[hh3_ethn_s_q2]<br><br>Show the field ONLY if: [language_q2] = '2' and [hhcount_s_q2] > 2 and [hhcount_s_q2] < 13	Persona 3: ¿Cuál es la identidad étnica de esta persona?	radio<br>1 Hispano o latino<br>2 Ni hispano ni latino<br>3 Otra<br>4 No sabe<br><br>Field Annotation: @DEFAULT="[hh3_ethn_s_q2]"
1901	[hh3_edu_s_q2]<br><br>Show the field ONLY if: [language_q2] = '2' and [hhcount_s_q2] > 2 and [hhcount_s_q2] < 13	Persona 3: ¿Cuál es el nivel de educación de esta persona?	radio<br>1 No tiene educación formal<br>2 Kínder a 8° grado<br>3 Estudios de educación secundaria<br>4 Equivalencia de educación secundaria (GED)<br>5 Diploma de educación secundaria<br>6 Estudios de educación superior<br>7 Título universitario<br>8 Estudios de posgrado o más<br>9 No sabe<br><br>Field Annotation: @DEFAULT="[hh3_edu_s_q2]"
1902	[hh3_work_s_q2]<br><br>Show the field ONLY if:	Persona 3: ¿Cuál de las siguientes es la mejor descripción de la situación laboral actual de esta persona?	radio<br>1 Empleo a tiempo completo

	[language_q2] = '2' and [hhcount_s_q2] > 2 and [hhcount_s_q2] < 13		2 Empleo a tiempo parcial<br>3 En busca de empleo<br>4 Jubilado<br>5 Ama de casa<br>6 Estudiante<br>7 Permiso de maternidad o paternidad<br>8 Permiso por enfermedad<br>9 Desempleado por discapacidad<br>10 Otra<br>11 No sabe<br><br>Field Annotation: @DEFAULT="[hh3_work_s_q2]"
1903	[hh3_work2_s_q2]<br><br>Show the field ONLY if: [language_q2] = '2' and [hhcount_s_q2] > 2 and [hhcount_s_q2] < 13	Persona 3: ¿Se considera esta persona actualmente empleado por cuenta propia (contratista independiente, trabajador esporádico (gig), etc.)?	radio<br>1 Sí<br>0 No<br>2 No sabe<br><br>Field Annotation: @DEFAULT="[hh3_work_s2_q2]"
1904	[hh3_work3_s_q2]<br><br>Show the field ONLY if: [language_q2] = '2' and [hhcount_s_q2] > 2 and [hhcount_s_q2] < 13 and ([hh3_work_s_q2] = '1' or [hh3_work_s_q2] = '2' or [hh3_work2_s_q2] = '1')	Persona 3: ¿Trabaja esta persona actualmente en alguno de los siguientes ambientes de alto riesgo de contagio de COVID-19?	checkbox<br>1 hh3_work3_s_q2___1 Ambiente de asistencia médica (hospital, clínica, centro de urgencias, etc.)<br>2 hh3_work3_s_q2___2 Ambiente residencial denso (hogar de ancianos, otro centro de asistencia de larga duración)<br>3 hh3_work3_s_q2___3 Prisión o cárcel<br>4 hh3_work3_s_q2___4 Establecimiento de envasado de carne<br>5 hh3_work3_s_q2___5 Establecimiento de envío o distribución<br>6 hh3_work3_s_q2___6 Establecimiento minorista de alto volumen (tienda de

			provisiones, etc.)<br>7 | hh3_work3_s_q2___7 | No sabe<br><br>Field Annotation: @DEFAULT="[hh3_work3_s_q2]"
1905	[hh3_work4_s_q2]<br><br>Show the field ONLY if: [language_q2] = '2' and [hhcount_s_q2] > 2 and [hhcount_s_q2] < 13 and ([hh3_work_s_q2] = '1' or [hh3_work_s_q2] = '2' or [hh3_work2_s_q2] = '1')	Persona 3: ¿Le ofrece el empleador a esta persona alguno de los siguientes beneficios en su empleo principal actual?<br>*(Seleccione todo lo que corresponda.)*	checkbox<br>1 | hh3_work4_s_q2___1 | Permiso por enfermedad con goce de sueldo<br>2 | hh3_work4_s_q2___2 | Vacaciones o permiso personal con goce de sueldo<br>3 | hh3_work4_s_q2___3 | Seguro de salud<br>4 | hh3_work4_s_q2___4 | Seguro de discapacidad<br>5 | hh3_work4_s_q2___5 | Plan de jubilación<br>6 | hh3_work4_s_q2___6 | Otro<br>7 | hh3_work4_s_q2___7 | No sabe<br><br>Field Annotation: @DEFAULT="[hh3_work4_s_q2]"
1906	[hh3_work5_s_q2]<br><br>Show the field ONLY if: [language_q2] = '2' and [hhcount_s_q2] > 2 and [hhcount_s_q2] < 13 and ([hh3_work_s_q2] = '1' or [hh3_work_s_q2] = '2' or [hh3_work2_s_q2] = '1')	Persona 3: En una escala de 0 (definitivamente no va a suceder) a 10 (definitivamente va a suceder), ¿qué tan probable es que esta persona pierda su empleo debido a la pandemia de COVID-19?	text (number, Min: 0, Max: 10)<br>Field Annotation: @DEFAULT="[hh3_work5_s_q2]"
1907	[hh3_work6_s_q2]<br><br>Show the field ONLY if: [language_q2] = '2' and [hhcount_s_q2] > 2 and [hhcount_s_q2] < 13 and ([hh3_work_s_q2] = '1' or [hh3_work_s_q2] = '2' or [hh3_work2_s_q2] = '1')	Persona 3: En una escala de 0 (definitivamente no va a suceder) a 10 (definitivamente va a suceder), ¿qué tan probable es que a esta persona se le asignen menos horas de trabajo debido a la pandemia de COVID-19?	text (number, Min: 0, Max: 10)<br>Field Annotation: @DEFAULT="[hh3_work6_s_q2]"
1908	[hh3_work7_s_q2]<br><br>Show the field ONLY if: [language_q2] = '2' and [hhcount_s_q2] > 2 and [hhcount_s_q2] < 13 and ([hh3_work_s_q2] =	Persona 3: Actualmente ¿con qué frecuencia se le exige a esta persona que trabaje fuera de su residencia?	radio (Matrix)<br>1 | Todo el tiempo (100%)<br>2 | La mayor parte del tiempo (75%)<br>3 | La mitad del tiempo (50%)<br>4 | Menos de la mitad del tiempo (25%)

	'1' or [hh3_work_s_q2] = '2' or [hh3_work2_s_q2] = '1')		5 nunca (0%)<br>6 Nunca
1909	[hh3_work8_s_q2]<br><br>Show the field ONLY if: [language_q2] = '2' and ([hh3_work7_s_q2] = '1' or [hh3_work7_s_q2] = '2' or [hh3_work7_s_q2] = '3' or [hh3_work7_s_q2] = '4')	Persona 3: Actualmente, ¿con qué frecuencia se encuentra esta persona físicamente cerca de sus compañeros de trabajo mientras trabaja fuera de su residencia?	radio (Matrix)<br>1 Todo el tiempo (100%)<br>2 La mayor parte del tiempo (75%)<br>3 La mitad del tiempo (50%)<br>4 Menos de la mitad del tiempo (25%)<br>5 nunca (0%)<br>6 Nunca
1910	[hh3_work9_s_q2]<br><br>Show the field ONLY if: [language_q2] = '2' and ([hh3_work7_s_q2] = '1' or [hh3_work7_s_q2] = '2' or [hh3_work7_s_q2] = '3' or [hh3_work7_s_q2] = '4')	Persona 3: Actualmente, ¿con qué frecuencia se encuentra esta persona físicamente cerca de sus clientes mientras trabaja fuera de su residencia	radio (Matrix)<br>1 Todo el tiempo (100%)<br>2 La mayor parte del tiempo (75%)<br>3 La mitad del tiempo (50%)<br>4 Menos de la mitad del tiempo (25%)<br>5 nunca (0%)<br>6 Nunca
1911	[hh3_covidvaccine_s_q2]<br><br>Show the field ONLY if: [language_q2] = '2' and [hhcount_s_q2] > 2 and [hhcount_s_q2] < 13	Persona 3: ¿Piensa esta persona vacunarse contra la COVID-19?	radio<br>1 Sí<br>0 No<br>2 No sabe<br>3 Este individuo ya ha sido vacunado<br><br>Field Annotation: @DEFAULT="[hh3_covidvaccine_s_q2]"
1912	[hh3_covidsymp_s_q2]<br><br>Show the field ONLY if: [language_q2] = '2' and [hhcount_s_q2] > 2 and [hhcount_s_q2] < 13	Persona 3: En las últimas dos semanas, ¿ha tenido esta persona algún síntoma de COVID-19 (tos, fiebre, dificultad para respirar, fatiga, dolores de cuerpo, diarrea, goteo nasal o pérdida del sentido del olfato o del gusto)?	radio<br>1 Sí<br>0 No<br>2 No sabe
1913	[hh3_covidsymp2_s_q2]<br><br>Show the field ONLY if: [language_q2] = '2' and [hh3_covidsymp_s_q2] = '1'	Persona 3: ¿Cuándo le comenzaron los síntomas de COVID-19 a esta persona?	text (date_mdy)
1914	[hh3_covidsymp3_s_q2]<br><br>Show the field ONLY if: [language_q2] = '2' and [hh3_covidsymp_s_q2] = '1'	Persona 3: En vista de sus síntomas, ¿le preocupa a esta persona la posibilidad de tener COVID-19?	radio<br>1 Sí<br>0 No<br>2 No sabe
1915	[hh3_covidsymp4_s_q2]	Persona 3: ¿Fue esta persona objeto de prejuicio o discriminación debido a sus síntomas?	radio

	Show the field ONLY if: [language_q2] = '2' and [hh3_covidsymp_s_q2] = '1'		1 Sí<br>0 No<br>2 No sabe
1916	[hh3_covidsymp5_s_q2]<br><br>Show the field ONLY if: [language_q2] = '2' and [hh3_covidsymp_s_q2] = '1'	Persona 3: ¿Qué hizo esta persona en vista de sus síntomas?<br>*(Seleccione todo lo que corresponda.)*	checkbox<br>0 hh3_covidsymp5_s_q2___0 Nada<br>1 hh3_covidsymp5_s_q2___1 Tomó medicament[illegible] sin receta (ibuprofeno, acetaminofé[illegible] etc.)<br>2 hh3_covidsymp5_s_q2___2 Consultó por teléfono a u[illegible] proveedor d[illegible] asistencia médica<br>3 hh3_covidsymp5_s_q2___3 Fue al consultorio [illegible] un proveedo[illegible] de asistencia médica<br>4 hh3_covidsymp5_s_q2___4 Fue a una clínica o una farmacia minorista<br>5 hh3_covidsymp5_s_q2___5 Fue a un centro de urgencias (FASTMed, etc.)<br>6 hh3_covidsymp5_s_q2___6 Fue a la sala de emergencias<br>7 hh3_covidsymp5_s_q2___7 Fue ingresad[illegible] al hospital<br>8 hh3_covidsymp5_s_q2___8 Otra cosa<br>9 hh3_covidsymp5_s_q2___9 No sabe
1917	[hh3_covidsymp6_s_q2]<br><br>Show the field ONLY if: [language_q2] = '2' and [hh3_covidsymp5_s_q2(8)] = '1'	Persona 3: ¿Qué otra cosa hizo esta persona en vista de sus síntomas?	text
1918	[hh3_covidsymp7_s_q2]<br><br>Show the field ONLY if: [language_q2] = '2' and ([hh3_covidsymp5_s_q2(2)] = '1' or [hh3_covids	Persona 3: ¿Le dijo un proveedor de asistencia médica a esta persona que era posible que tuviera COVID-19?	radio<br>1 Sí<br>0 No<br>2 No sabe

	ymp5_s_q2(3)] = '1' or [hh3_covidsymp5_s_q2(4)] = '1' or [hh3_covidsymp5_s_q2(5)] = '1' or [hh3_covidsymp5_s_q2(6)] = '1' or [hh3_covidsymp5_s_q2(7)] = '1' or [hh3_covidsymp5_s_q2(8)] = '1')		
1919	[hh3_covid_test_s_q2]<br>Show the field ONLY if: [language_q2] = '2' and [hh3_covidsymp_s_q2] = '1'	Persona 3: Si a esta persona le hicieron una prueba de COVID-19 en vista de sus síntomas, ¿cuál fue el resultado?	radio<br>1 Pendiente<br>2 Positivo<br>3 Negativo<br>4 No concluyente<br>5 No se hizo la prueba<br>6 No sabe
1920	[hh3_covid_admit_s_q2]<br>Show the field ONLY if: [language_q2] = '2' and [hh3_covidsymp5_s_q2(7)] = '1'	Persona 3: ¿Cuántos días estuvo hospitalizada esta persona?	text (number, Min: 0)
1921	[hh3_covid_admit2_s_q2]<br>Show the field ONLY if: [language_q2] = '2' and [hh3_covidsymp5_s_q2(7)] = '1'	Persona 3: ¿Se le hicieron a esta persona las siguientes intervenciones durante su hospitalización?	checkbox<br>1 hh3_covid_admit2_s_q2___1 Oxígeno adicional por la nariz<br>2 hh3_covid_admit2_s_q2___2 Tratamiento en la Unidad de Cuidados Intensivos (Intensive Care Unit, ICU)<br>3 hh3_covid_admit2_s_q2___3 Ventilación mecánica (intubación o tubo de respiración)<br>4 hh3_covid_admit2_s_q2___4 No sabe
1922	[hh3_covidsymp8_s_q2]<br>Show the field ONLY if: [language_q2] = '2' and [hh3_covidsymp_s_q2] = '1'	Persona 3: ¿Ha vuelto esta persona a su salud normal?	radio<br>1 Sí<br>0 No<br>2 No sabe
1923	[hh3_prevent_s_q2]<br>Show the field ONLY if:	Persona 3: ¿Cuáles de las siguientes medidas tomó esta persona para proteger a sus amigos y	checkbox<br>1 hh3_prevent_s_q2___1 Ponerse mascarilla

	[language_q2] = '2' and [hh3_covidsymp_s_q2] = '1'	familiares después de que comenzaron sus síntomas?	| | | con mayor frecuencia | | 2 | hh3_prevent_s_q2___2 | Lavarse las manos con agua y jabón con mayor frecuencia | | 3 | hh3_prevent_s_q2___3 | Limpiarse las manos con desinfectante con mayor frecuencia | | 4 | hh3_prevent_s_q2___4 | Aislarse en su casa con mayor frecuencia | | 5 | hh3_prevent_s_q2___5 | Quedarse en casa con mayor frecuencia | | 6 | hh3_prevent_s_q2___6 | Ponerse guantes desechables con mayor frecuencia | | 7 | hh3_prevent_s_q2___7 | No sabe |
1924	[hh4_relationship_s_q2]<br><br>Show the field ONLY if: [language_q2] = '2' and [hhcount_s_q2] > 3 and [hhcount_s_q2] < 13	Section Header: *Por cada persona adicional en su hogar, proporcione la siguiente informacion.*<br><br>Persona 4: ¿Cuál es su relación con esta persona?	radio<br>| 1 | Pareja o cónyuge | | 2 | Hijo o hija | | 3 | Padre o madre | | 4 | Hermano o hermana | | 5 | Otro familiar | | 6 | Proveedor de cuidado infantil u otros cuidados en casa | | 7 | Otra | <br>Field Annotation: @DEFAULT="[hh4_relationship_s_q2]"
1925	[hh4_relationship2_s_q2]<br><br>Show the field ONLY if: [language_q2] = '2' and [hh4_relationship_s_q2] = '7'	Persona 4: Especifique su relación con esta persona.	text<br>Field Annotation: @DEFAULT="[hh4_relationship2_s_q2]"

1926	[hh4_age_s_q2]<br><br>Show the field ONLY if:<br>[language_q2] = '2' and<br>[hhcount_s_q2] > 3 and<br>[hhcount_s_q2] < 13	Persona 4: ¿Qué edad tiene esta persona?<br>*(Especifique la edad en años)*	text (number, Min: 0, Max: 110)<br>Field Annotation: @DEFAULT="<br>[hh4_age_s_q2]"
1927	[hh4_s_q2ex_s_q2]<br><br>Show the field ONLY if:<br>[language_q2] = '2' and<br>[hhcount_s_q2] > 3 and<br>[hhcount_s_q2] < 13	Persona 4: ¿Cuál es el sexo de esta persona?	radio<br>1 Femenino<br>2 Masculino<br><br>Field Annotation: @DEFAULT="<br>[hh4_s_q2ex_s_q2]"
1928	[hh4_race_s_q2]<br><br>Show the field ONLY if:<br>[language_q2] = '2' and<br>[hhcount_s_q2] > 3 and<br>[hhcount_s_q2] < 13	Persona 4: ¿Cuál es la raza de esta persona?<br>*(Seleccione todo lo que corresponda.)*	checkbox<br>1 hh4_race_s_q2___1 Indio americano o nativo de Alaska<br>2 hh4_race_s_q2___2 Asiático<br>3 hh4_race_s_q2___3 Negro o afroestadounidense<br>4 hh4_race_s_q2___4 Nativo de Hawái o de las islas del Pacífico<br>5 hh4_race_s_q2___5 Blanco<br>6 hh4_race_s_q2___6 Otra<br>7 hh4_race_s_q2___7 No sabe<br><br>Field Annotation: @DEFAULT="<br>[hh4_race_s_q2]"
1929	[hh4_ethn_s_q2]<br><br>Show the field ONLY if:<br>[language_q2] = '2' and<br>[hhcount_s_q2] > 3 and<br>[hhcount_s_q2] < 13	Persona 4: ¿Cuál es la identidad étnica de esta persona?	radio<br>1 Hispano o latino<br>2 Ni hispano ni latino<br>3 Otra<br>4 No sabe<br><br>Field Annotation: @DEFAULT="<br>[hh4_ethn_s_q2]"
1930	[hh4_edu_s_q2]<br><br>Show the field ONLY if:<br>[language_q2] = '2' and<br>[hhcount_s_q2] > 3 and<br>[hhcount_s_q2] < 13	Persona 4: ¿Cuál es el nivel de educación de esta persona?	radio<br>1 No tiene educación formal<br>2 Kínder a 8° grado<br>3 Estudios de educación secundaria<br>4 Equivalencia de educación secundaria (GED)<br>5 Diploma de educación secundaria<br>6 Estudios de educación superior<br>7 Título universitario<br>8 Estudios de posgrado o más

			9 No sabe<br><br>Field Annotation: @DEFAULT="[hh4_edu_s_q2]"
1931	[hh4_work_s_q2]<br><br>Show the field ONLY if: [language_q2] = '2' and [hhcount_s_q2] > 3 and [hhcount_s_q2] < 13	Persona 4: ¿Cuál de las siguientes es la mejor descripción de la situación laboral actual de esta persona?	radio<br>1 Empleo a tiempo completo<br>2 Empleo a tiempo parcial<br>3 En busca de empleo<br>4 Jubilado<br>5 Ama de casa<br>6 Estudiante<br>7 Permiso de maternidad o paternidad<br>8 Permiso por enfermedad<br>9 Desempleado por discapacidad<br>10 Otra<br>11 No sabe<br><br>Field Annotation: @DEFAULT="[hh4_work_s_q2]"
1932	[hh4_work2_s_q2]<br><br>Show the field ONLY if: [language_q2] = '2' and [hhcount_s_q2] > 3 and [hhcount_s_q2] < 13	Persona 4: ¿Se considera esta persona actualmente empleado por cuenta propia (contratista independiente, trabajador esporádico (gig), etc.)?	radio<br>1 Sí<br>0 No<br>2 No sabe<br><br>Field Annotation: @DEFAULT="[hh4_work2_s_q2]"
1933	[hh4_work3_s_q2]<br><br>Show the field ONLY if: [language_q2] = '2' and [hhcount_s_q2] > 3 and [hhcount_s_q2] < 13 and ([hh4_work_s_q2] = '1' or [hh4_work_s_q2] = '2' or [hh4_work2_s_q2] = '1')	Persona 4: ¿Trabaja esta persona actualmente en alguno de los siguientes ambientes de alto riesgo de contagio de COVID-19?	checkbox<br>1 hh4_work3_s_q2___1 Ambiente de asistencia médica (hospital, clínica, centro de urgencias, etc.)<br>2 hh4_work3_s_q2___2 Ambiente residencial denso (hogar de ancianos, otro centro de asistencia de larga duración)<br>3 hh4_work3_s_q2___3 Prisión o cárcel<br>4 hh4_work3_s_q2___4 Establecimiento de envasado de carne

			5 hh4_work3_s_q2___5 Establecimiento de envío o distribución<br>6 hh4_work3_s_q2___6 Establecimiento minorista de alto volumen (tienda de provisiones, etc.)<br>7 hh4_work3_s_q2___7 No sabe<br><br>Field Annotation: @DEFAULT=" [hh4_work3_s_q2]"
1934	[hh4_work4_s_q2]<br><br>Show the field ONLY if: [language_q2] = '2' and [hhcount_s_q2] > 3 and [hhcount_s_q2] < 13 and ([hh4_work_s_q2] = '1' or [hh4_work_s_q2] = '2' or [hh4_work2_s_q2] = '1')	Persona 4: ¿Le ofrece el empleador a esta persona alguno de los siguientes beneficios en su empleo principal actual?<br>*(Seleccione todo lo que corresponda.)*	checkbox<br>1 hh4_work4_s_q2___1 Permiso por enfermedad con goce de sueldo<br>2 hh4_work4_s_q2___2 Vacaciones o permiso personal con goce de sueldo<br>3 hh4_work4_s_q2___3 Seguro de salud<br>4 hh4_work4_s_q2___4 Seguro de discapacidad<br>5 hh4_work4_s_q2___5 Plan de jubilación<br>6 hh4_work4_s_q2___6 Otro<br>7 hh4_work4_s_q2___7 No sabe<br><br>Field Annotation: @DEFAULT=" [hh4_work4_s_q2]"
1935	[hh4_work5_s_q2]<br><br>Show the field ONLY if: [language_q2] = '2' and [hhcount_s_q2] > 3 and [hhcount_s_q2] < 13 and ([hh4_work_s_q2] = '1' or [hh4_work_s_q2] = '2' or [hh4_work2_s_q2] = '1')	Persona 4: En una escala de 0 (definitivamente no va a suceder) a 10 (definitivamente va a suceder), ¿qué tan probable es que esta persona pierda su empleo debido a la pandemia de COVID-19?	text (number, Min: 0, Max: 10)<br>Field Annotation: @DEFAULT=" [hh4_work5_s_q2]"
1936	[hh4_work6_s_q2]<br><br>Show the field ONLY if: [language_q2] = '2' and [hhcount_s_q2] > 3 and [hhcount_s_q2] < 13 and ([hh4_work_s_q2] = '1' or [hh4_work_s_q2]	Persona 4: En una escala de 0 (definitivamente no va a suceder) a 10 (definitivamente va a suceder), ¿qué tan probable es que a esta persona se le asignen menos horas de trabajo debido a la pandemia de COVID-19?	text (number, Min: 0, Max: 10)<br>Field Annotation: @DEFAULT=" [hh4_work6_s_q2]"

	= '2' or [hh4_work2_s_q2] = '1')		
1937	[hh4_work7_s_q2]<br><br>Show the field ONLY if: [language_q2] = '2' and [hhcount_s_q2] > 3 and [hhcount_s_q2] < 13 and ([hh4_work_s_q2] = '1' or [hh4_work_s_q2] = '2' or [hh4_work2_s_q2] = '1')	Persona 4: Actualmente ¿con qué frecuencia se le exige a esta persona que trabaje fuera de su residencia?	radio (Matrix)<br>1 Todo el tiempo (100%)<br>2 La mayor parte del tiempo (75%)<br>3 La mitad del tiempo (50%)<br>4 Menos de la mitad del tiempo (25%)<br>5 nunca (0%)<br>6 Nunca
1938	[hh4_work8_s_q2]<br><br>Show the field ONLY if: [language_q2] = '2' and ([hh4_work7_s_q2] = '1' or [hh4_work7_s_q2] = '2' or [hh4_work7_s_q2] = '3' or [hh4_work7_s_q2] = '4')	Persona 4: Actualmente, ¿con qué frecuencia se encuentra esta persona físicamente cerca de sus compañeros de trabajo mientras trabaja fuera de su residencia?	radio (Matrix)<br>1 Todo el tiempo (100%)<br>2 La mayor parte del tiempo (75%)<br>3 La mitad del tiempo (50%)<br>4 Menos de la mitad del tiempo (25%)<br>5 nunca (0%)<br>6 Nunca
1939	[hh4_work9_s_q2]<br><br>Show the field ONLY if: [language_q2] = '2' and ([hh4_work7_s_q2] = '1' or [hh4_work7_s_q2] = '2' or [hh4_work7_s_q2] = '3' or [hh4_work7_s_q2] = '4')	Persona 4: Actualmente, ¿con qué frecuencia se encuentra esta persona físicamente cerca de sus clientes mientras trabaja fuera de su residencia	radio (Matrix)<br>1 Todo el tiempo (100%)<br>2 La mayor parte del tiempo (75%)<br>3 La mitad del tiempo (50%)<br>4 Menos de la mitad del tiempo (25%)<br>5 nunca (0%)<br>6 Nunca
1940	[hh4_covidvaccine_s_q2]<br><br>Show the field ONLY if: [language_q2] = '2' and [hhcount_s_q2] > 3 and [hhcount_s_q2] < 13	Persona 4: ¿Piensa esta persona vacunarse contra la COVID-19?	radio<br>1 Sí<br>0 No<br>2 No sabe<br>3 Este individuo ya ha sido vacunado<br><br>Field Annotation: @DEFAULT="[hh4_covidvaccine_s_q2]"
1941	[hh4_covidsymp_s_q2]<br><br>Show the field ONLY if: [language_q2] = '2' and [hhcount_s_q2] > 3 and [hhcount_s_q2] < 13	Persona 4: En las últimas dos semanas, ¿ha tenido esta persona algún síntoma de COVID-19 (tos, fiebre, dificultad para respirar, fatiga, dolores de cuerpo, diarrea, goteo nasal o pérdida del sentido del olfato o del gusto)?	radio<br>1 Sí<br>0 No<br>2 No sabe
1942	[hh4_covidsymp2_s_q2]<br><br>Show the field ONLY if: [language_q2] = '2' and [hh4_covidsymp_s_q2] = '1'	Persona 4: ¿Cuándo le comenzaron los síntomas de COVID-19 a esta persona?	text (date_mdy)

1943	[hh4_covidsymp3_s_q2]<br>Show the field ONLY if: [language_q2] = '2' and [hh4_covidsymp_s_q2] = '1'	Persona 4: En vista de sus síntomas, ¿le preocupa a esta persona la posibilidad de tener COVID-19?	radio<br>1 Sí<br>0 No<br>2 No sabe
1944	[hh4_covidsymp4_s_q2]<br>Show the field ONLY if: [language_q2] = '2' and [hh4_covidsymp_s_q2] = '1'	Persona 4: ¿Fue esta persona objeto de prejuicio o discriminación debido a sus síntomas?	radio<br>1 Sí<br>0 No<br>2 No sabe
1945	[hh4_covidsymp5_s_q2]<br>Show the field ONLY if: [language_q2] = '2' and [hh4_covidsymp_s_q2] = '1'	Persona 4: ¿Qué hizo esta persona en vista de sus síntomas?<br>*(Seleccione todo lo que corresponda.)*	checkbox<br>0 hh4_covidsymp5_s_q2___0 Nada<br>1 hh4_covidsymp5_s_q2___1 Tomó medicament sin receta (ibuprofeno, acetaminofé etc.)<br>2 hh4_covidsymp5_s_q2___2 Consultó por teléfono a un proveedor de asistencia médica<br>3 hh4_covidsymp5_s_q2___3 Fue al consultorio c un proveedo de asistencia médica<br>4 hh4_covidsymp5_s_q2___4 Fue a una clínica o una farmacia minorista<br>5 hh4_covidsymp5_s_q2___5 Fue a un centro de urgencias (FASTMed, etc.)<br>6 hh4_covidsymp5_s_q2___6 Fue a la sala de emergencias<br>7 hh4_covidsymp5_s_q2___7 Fue ingresad al hospital<br>8 hh4_covidsymp5_s_q2___8 Otra cosa<br>9 hh4_covidsymp5_s_q2___9 No sabe
1946	[hh4_covidsymp6_s_q2]<br>Show the field ONLY if:	Persona 4: ¿Qué otra cosa hizo esta persona en vista de sus síntomas?	text

	[language_q2] = '2' and [hh4_covidsymp5_s_q2(8)] = '1'		
1947	[hh4_covidsymp7_s_q2]<br><br>Show the field ONLY if: [language_q2] = '2' and ([hh4_covidsymp5_s_q2(2)] = '1' or [hh4_covidsymp5_s_q2(3)] = '1' or [hh4_covidsymp5_s_q2(4)] = '1' or [hh4_covidsymp5_s_q2(5)] = '1' or [hh4_covidsymp5_s_q2(6)] = '1' or [hh4_covidsymp5_s_q2(7)] = '1' or [hh4_covidsymp5_s_q2(8)] = '1')	Persona 4: ¿Le dijo un proveedor de asistencia médica a esta persona que era posible que tuviera COVID-19?	radio<br>1 Sí<br>0 No<br>2 No sabe
1948	[hh4_covid_test_s_q2]<br><br>Show the field ONLY if: [language_q2] = '2' and [hh4_covidsymp_s_q2] = '1'	Persona 4: Si a esta persona le hicieron una prueba de COVID-19 en vista de sus síntomas, ¿cuál fue el resultado?	radio<br>1 Pendiente<br>2 Positivo<br>3 Negativo<br>4 No concluyente<br>5 No se hizo la prueba<br>6 No sabe
1949	[hh4_covid_admit_s_q2]<br><br>Show the field ONLY if: [language_q2] = '2' and [hh4_covidsymp5_s_q2(7)] = '1'	Persona 4: ¿Cuántos días estuvo hospitalizada esta persona?	text (number, Min: 0)
1950	[hh4_covid_admit2_s_q2]<br><br>Show the field ONLY if: [language_q2] = '2' and [hh4_covidsymp5_s_q2(7)] = '1'	Persona 4: ¿Se le hicieron a esta persona las siguientes intervenciones durante su hospitalización?	checkbox<br>1 hh4_covid_admit2_s_q2___1 Oxígeno adicional por la nariz<br>2 hh4_covid_admit2_s_q2___2 Tratamiento en la Unidad de Cuidados Intensivos (Intensive Care Unit, ICU)<br>3 hh4_covid_admit2_s_q2___3 Ventilación mecánica (intubación o tubo de respiración)<br>4 hh4_covid_admit2_s_q2___4 No sabe

1951	[hh4_covidsymp8_s_q2]<br><br>Show the field ONLY if: [language_q2] = '2' and [hh4_covidsymp_s_q2] = '1'	Persona 4: ¿Ha vuelto esta persona a su salud normal?	radio<br>1 Sí<br>0 No<br>2 No sabe
1952	[hh4_prevent_s_q2]<br><br>Show the field ONLY if: [language_q2] = '2' and [hh4_covidsymp_s_q2] = '1'	Persona 4: ¿Cuáles de las siguientes medidas tomó esta persona para proteger a sus amigos y familiares después de que comenzaron sus síntomas?	checkbox<br>1 hh4_prevent_s_q2___1 Ponerse mascarilla con mayor frecuencia<br>2 hh4_prevent_s_q2___2 Lavarse las manos con agua y jabón con mayor frecuencia<br>3 hh4_prevent_s_q2___3 Limpiarse las manos con desinfectante con mayor frecuencia<br>4 hh4_prevent_s_q2___4 Aislarse en su casa con mayor frecuencia<br>5 hh4_prevent_s_q2___5 Quedarse en casa con mayor frecuencia<br>6 hh4_prevent_s_q2___6 Ponerse guantes desechables con mayor frecuencia<br>7 hh4_prevent_s_q2___7 No sabe
1953	[hh5_relationship_s_q2]<br><br>Show the field ONLY if: [language_q2] = '2' and [hhcount_s_q2] > 4 and [hhcount_s_q2] < 13	Section Header: *Por cada persona adicional en su hogar, proporcione la siguiente informacion.*<br><br>Persona 5: ¿Cuál es su relación con esta persona?	radio<br>1 Pareja o cónyuge<br>2 Hijo o hija<br>3 Padre o madre<br>4 Hermano o hermana<br>5 Otro familiar<br>6 Proveedor de cuidado infantil u otros cuidados en casa<br>7 Otra<br><br>Field Annotation: @DEFAULT="[hh5_relationship_s_q2]"

1954	[hh5_relationship2_s_q2]<br><br>Show the field ONLY if: [language_q2] = '2' and [hh5_relationship_s_q2] = '7'	Persona 5: Especifique su relación con esta persona.	text<br>Field Annotation: @DEFAULT="[hh5_relationship2_s_q2]"
1955	[hh5_age_s_q2]<br><br>Show the field ONLY if: [language_q2] = '2' and [hhcount_s_q2] > 4 and [hhcount_s_q2] < 13	Persona 5: ¿Qué edad tiene esta persona?<br>*(Especifique la edad en años)*	text (number, Min: 0, Max: 110)<br>Field Annotation: @DEFAULT="[hh5_age_s_q2]"
1956	[hh5_s_q2ex_s_q2]<br><br>Show the field ONLY if: [language_q2] = '2' and [hhcount_s_q2] > 4 and [hhcount_s_q2] < 13	Persona 5: ¿Cuál es el sexo de esta persona?	radio<br>1 Femenino<br>2 Masculino<br><br>Field Annotation: @DEFAULT="[hh5_s_q2ex_s_q2]"
1957	[hh5_race_s_q2]<br><br>Show the field ONLY if: [language_q2] = '2' and [hhcount_s_q2] > 4 and [hhcount_s_q2] < 13	Persona 5: ¿Cuál es la raza de esta persona?<br>*(Seleccione todo lo que corresponda.)*	checkbox<br>1 hh5_race_s_q2___1 Indio americano o nativo de Alaska<br>2 hh5_race_s_q2___2 Asiático<br>3 hh5_race_s_q2___3 Negro o afroestadounidense<br>4 hh5_race_s_q2___4 Nativo de Hawái o de las islas del Pacífico<br>5 hh5_race_s_q2___5 Blanco<br>6 hh5_race_s_q2___6 Otra<br>7 hh5_race_s_q2___7 No sabe<br><br>Field Annotation: @DEFAULT="[hh5_race_s_q2]"
1958	[hh5_ethn_s_q2]<br><br>Show the field ONLY if: [language_q2] = '2' and [hhcount_s_q2] > 4 and [hhcount_s_q2] < 13	Persona 5: ¿Cuál es la identidad étnica de esta persona?	radio<br>1 Hispano o latino<br>2 Ni hispano ni latino<br>3 Otra<br>4 No sabe<br><br>Field Annotation: @DEFAULT="[hh5_ethn_s_q2]"
1959	[hh5_edu_s_q2]<br><br>Show the field ONLY if: [language_q2] = '2' and [hhcount_s_q2] > 4 and [hhcount_s_q2] < 13	Persona 5: ¿Cuál es el nivel de educación de esta persona?	radio<br>1 No tiene educación formal<br>2 Kínder a 8° grado<br>3 Estudios de educación secundaria<br>4 Equivalencia de educación secundaria (GED)

			5 Diploma de educación secundaria<br>6 Estudios de educación superior<br>7 Título universitario<br>8 Estudios de posgrado o más<br>9 No sabe<br><br>Field Annotation: @DEFAULT="[hh5_edu_s_q2]"
1960	[hh5_work_s_q2]<br><br>Show the field ONLY if: [language_q2] = '2' and [hhcount_s_q2] > 4 and [hhcount_s_q2] < 13	Persona 5: ¿Cuál de las siguientes es la mejor descripción de la situación laboral actual de esta persona?	radio<br>1 Empleo a tiempo completo<br>2 Empleo a tiempo parcial<br>3 En busca de empleo<br>4 Jubilado<br>5 Ama de casa<br>6 Estudiante<br>7 Permiso de maternidad o paternidad<br>8 Permiso por enfermedad<br>9 Desempleado por discapacidad<br>10 Otra<br>11 No sabe<br><br>Field Annotation: @DEFAULT="[hh5_work_s_q2]"
1961	[hh5_work2_s_q2]<br><br>Show the field ONLY if: [language_q2] = '2' and [hhcount_s_q2] > 4 and [hhcount_s_q2] < 13	Persona 5: ¿Se considera esta persona actualmente empleado por cuenta propia (contratista independiente, trabajador esporádico (gig), etc.)?	radio<br>1 Sí<br>0 No<br>2 No sabe<br><br>Field Annotation: @DEFAULT="[hh5_work2_s_q2]"
1962	[hh5_work3_s_q2]<br><br>Show the field ONLY if: [language_q2] = '2' and [hhcount_s_q2] > 4 and [hhcount_s_q2] < 13 and ([hh5_work_s_q2] = '1' or [hh5_work_s_q2] = '2' or [hh5_work2_s_q2] = '1')	Persona 5: ¿Trabaja esta persona actualmente en alguno de los siguientes ambientes de alto riesgo de contagio de COVID-19?	checkbox<br>1 hh5_work3_s_q2___1 Ambiente de asistencia médica (hospital, clínica, centro de urgencias, etc.)<br>2 hh5_work3_s_q2___2 Ambiente residencial denso (hogar de ancianos, otro centro de asistencia de larga duración)

			3 hh5_work3_s_q2___3 Prisión o cárcel<br>4 hh5_work3_s_q2___4 Establecimiento de envasado de carne<br>5 hh5_work3_s_q2___5 Establecimiento de envío o distribución<br>6 hh5_work3_s_q2___6 Establecimiento minorista de alto volumen (tienda de provisiones, etc.)<br>7 hh5_work3_s_q2___7 No sabe<br><br>Field Annotation: @DEFAULT="[hh5_work3_s_q2]"
1963	[hh5_work4_s_q2]<br><br>Show the field ONLY if: [language_q2] = '2' and [hhcount_s_q2] > 4 and [hhcount_s_q2] < 13 and ([hh5_work_s_q2] = '1' or [hh5_work_s_q2] = '2' or [hh5_work2_s_q2] = '1')	Persona 5: ¿Le ofrece el empleador a esta persona alguno de los siguientes beneficios en su empleo principal actual?<br>*(Seleccione todo lo que corresponda.)*	checkbox<br>1 hh5_work4_s_q2___1 Permiso por enfermedad con goce de sueldo<br>2 hh5_work4_s_q2___2 Vacaciones o permiso personal con goce de sueldo<br>3 hh5_work4_s_q2___3 Seguro de salud<br>4 hh5_work4_s_q2___4 Seguro de discapacidad<br>5 hh5_work4_s_q2___5 Plan de jubilación<br>6 hh5_work4_s_q2___6 Otro<br>7 hh5_work4_s_q2___7 No sabe<br><br>Field Annotation: @DEFAULT="[hh5_work4_s_q2]"
1964	[hh5_work5_s_q2]<br><br>Show the field ONLY if: [language_q2] = '2' and [hhcount_s_q2] > 4 and [hhcount_s_q2] < 13 and ([hh5_work_s_q2] = '1' or [hh5_work_s_q2] = '2' or [hh5_work2_s_q2] = '1')	Persona 5: En una escala de 0 (definitivamente no va a suceder) a 10 (definitivamente va a suceder), ¿qué tan probable es que esta persona pierda su empleo debido a la pandemia de COVID-19?	text (number, Min: 0, Max: 10)<br>Field Annotation: @DEFAULT="[hh5_work5_s_q2]"
1965	[hh5_work6_s_q2]<br><br>Show the field ONLY if:	Persona 5: En una escala de 0 (definitivamente no va a suceder) a 10 (definitivamente va a suceder), ¿qué tan probable es que a esta persona se le	text (number, Min: 0, Max: 10)<br>Field Annotation: @DEFAULT="[hh5_work6_s_q2]"

	[language_q2] = '2' and [hhcount_s_q2] > 4 and [hhcount_s_q2] < 13 and ([hh5_work_s_q2] = '1' or [hh5_work_s_q2] = '2' or [hh5_work2_s_q2] = '1')	asignen menos horas de trabajo debido a la pandemia de COVID-19?	
1966	[hh5_work7_s_q2]<br>Show the field ONLY if: [language_q2] = '2' and [hhcount_s_q2] > 4 and [hhcount_s_q2] < 13 and ([hh5_work_s_q2] = '1' or [hh5_work_s_q2] = '2' or [hh5_work2_s_q2] = '1')	Persona 5: Actualmente ¿con qué frecuencia se le exige a esta persona que trabaje fuera de su residencia?	radio (Matrix)<br>1 Todo el tiempo (100%)<br>2 La mayor parte del tiempo (75%)<br>3 La mitad del tiempo (50%)<br>4 Menos de la mitad del tiempo (25%)<br>5 nunca (0%)<br>6 Nunca
1967	[hh5_work8_s_q2]<br>Show the field ONLY if: [language_q2] = '2' and ([hh5_work7_s_q2] = '1' or [hh5_work7_s_q2] = '2' or [hh5_work7_s_q2] = '3' or [hh5_work7_s_q2] = '4')	Persona 5: Actualmente, ¿con qué frecuencia se encuentra esta persona físicamente cerca de sus compañeros de trabajo mientras trabaja fuera de su residencia?	radio (Matrix)<br>1 Todo el tiempo (100%)<br>2 La mayor parte del tiempo (75%)<br>3 La mitad del tiempo (50%)<br>4 Menos de la mitad del tiempo (25%)<br>5 nunca (0%)<br>6 Nunca
1968	[hh5_work9_s_q2]<br>Show the field ONLY if: [language_q2] = '2' and ([hh5_work7_s_q2] = '1' or [hh5_work7_s_q2] = '2' or [hh5_work7_s_q2] = '3' or [hh5_work7_s_q2] = '4')	Persona 5: Actualmente, ¿con qué frecuencia se encuentra esta persona físicamente cerca de sus clientes mientras trabaja fuera de su residencia	radio (Matrix)<br>1 Todo el tiempo (100%)<br>2 La mayor parte del tiempo (75%)<br>3 La mitad del tiempo (50%)<br>4 Menos de la mitad del tiempo (25%)<br>5 nunca (0%)<br>6 Nunca
1969	[hh5_covidvaccine_s_q2]<br>Show the field ONLY if: [language_q2] = '2' and [hhcount_s_q2] > 4 and [hhcount_s_q2] < 13	Persona 5: ¿Piensa esta persona vacunarse contra la COVID-19?	radio<br>1 Sí<br>0 No<br>2 No sabe<br>3 Este individuo ya ha sido vacunado<br>Field Annotation: @DEFAULT="[hh5_covidvaccine_s_q2]"
1970	[hh5_covidsymp_s_q2]<br>Show the field ONLY if: [language_q2] = '2' and [hhcount_s_q2] > 4 and [hhcount_s_q2] < 13	Persona 5: En las últimas dos semanas, ¿ha tenido esta persona algún síntoma de COVID-19 (tos, fiebre, dificultad para respirar, fatiga, dolores de cuerpo, diarrea, goteo nasal o pérdida del sentido del olfato o del gusto)?	radio<br>1 Sí<br>0 No<br>2 No sabe

1971	[hh5_covidsymp2_s_q2]<br><br>Show the field ONLY if: [language_q2] = '2' and [hh5_covidsymp_s_q2] = '1'	Persona 5: ¿Cuándo le comenzaron los síntomas de COVID-19 a esta persona?	text (date_mdy)
1972	[hh5_covidsymp3_s_q2]<br><br>Show the field ONLY if: [language_q2] = '2' and [hh5_covidsymp_s_q2] = '1'	Persona 5: En vista de sus síntomas, ¿le preocupa a esta persona la posibilidad de tener COVID-19?	radio<br>1 Sí<br>0 No<br>2 No sabe
1973	[hh5_covidsymp4_s_q2]<br><br>Show the field ONLY if: [language_q2] = '2' and [hh5_covidsymp_s_q2] = '1'	Persona 5: ¿Fue esta persona objeto de prejuicio o discriminación debido a sus síntomas?	radio<br>1 Sí<br>0 No<br>2 No sabe
1974	[hh5_covidsymp5_s_q2]<br><br>Show the field ONLY if: [language_q2] = '2' and [hh5_covidsymp_s_q2] = '1'	Persona 5: ¿Qué hizo esta persona en vista de sus síntomas?<br>*(Seleccione todo lo que corresponda.)*	checkbox<br>0 hh5_covidsymp5_s_q2___0 Nada<br>1 hh5_covidsymp5_s_q2___1 Tomó medicament[illegible] sin receta (ibuprofeno, acetaminofé[illegible] etc.)<br>2 hh5_covidsymp5_s_q2___2 Consultó por teléfono a un proveedor de asistencia médica<br>3 hh5_covidsymp5_s_q2___3 Fue al consultorio [illegible] un proveedo[illegible] de asistencia médica<br>4 hh5_covidsymp5_s_q2___4 Fue a una clínica o una farmacia minorista<br>5 hh5_covidsymp5_s_q2___5 Fue a un centro de urgencias (FASTMed, etc.)<br>6 hh5_covidsymp5_s_q2___6 Fue a la sala de emergencias<br>7 hh5_covidsymp5_s_q2___7 Fue ingresad[illegible] al hospital

			8 hh5_covidsymp5_s_q2___8 Otra cosa<br>9 hh5_covidsymp5_s_q2___9 No sabe
1975	[hh5_covidsymp6_s_q2]<br><br>Show the field ONLY if: [language_q2] = '2' and [hh5_covidsymp5_s_q2(8)] = '1'	Persona 5: ¿Qué otra cosa hizo esta persona en vista de sus síntomas?	text
1976	[hh5_covidsymp7_s_q2]<br><br>Show the field ONLY if: [language_q2] = '2' and ([hh5_covidsymp5_s_q2(2)] = '1' or [hh5_covidsymp5_s_q2(3)] = '1' or [hh5_covidsymp5_s_q2(4)] = '1' or [hh5_covidsymp5_s_q2(5)] = '1' or [hh5_covidsymp5_s_q2(6)] = '1' or [hh5_covidsymp5_s_q2(7)] = '1' or [hh5_covidsymp5_s_q2(8)] = '1')	Persona 5: ¿Le dijo un proveedor de asistencia médica a esta persona que era posible que tuviera COVID-19?	radio<br>1 Sí<br>0 No<br>2 No sabe
1977	[hh5_covid_test_s_q2]<br><br>Show the field ONLY if: [language_q2] = '2' and [hh5_covidsymp_s_q2] = '1'	Persona 5: Si a esta persona le hicieron una prueba de COVID-19 en vista de sus síntomas, ¿cuál fue el resultado?	radio<br>1 Pendiente<br>2 Positivo<br>3 Negativo<br>4 No concluyente<br>5 No se hizo la prueba<br>6 No sabe
1978	[hh5_covid_admit_s_q2]<br><br>Show the field ONLY if: [language_q2] = '2' and [hh5_covidsymp5_s_q2(7)] = '1'	Persona 5: ¿Cuántos días estuvo hospitalizada esta persona?	text (number, Min: 0)
1979	[hh5_covid_admit2_s_q2]<br><br>Show the field ONLY if: [language_q2] = '2' and [hh5_covidsymp5_s_q2(7)] = '1'	Persona 5: ¿Se le hicieron a esta persona las siguientes intervenciones durante su hospitalización?	checkbox<br>1 hh5_covid_admit2_s_q2___1 Oxígeno adicional por la nariz<br>2 hh5_covid_admit2_s_q2___2 Tratamiento en la Unidad de Cuidados Intensivos (Intensive Care Unit, ICU)

			3 hh5_covid_admit2_s_q2___3 Ventilación mecánica (intubación o tubo de respiración)<br>4 hh5_covid_admit2_s_q2___4 No sabe
1980	[hh5_covidsymp8_s_q2]<br><br>Show the field ONLY if: [language_q2] = '2' and [hh5_covidsymp_s_q2] = '1'	Persona 5: ¿Ha vuelto esta persona a su salud normal?	radio<br>1 Sí<br>0 No<br>2 No sabe
1981	[hh5_prevent_s_q2]<br><br>Show the field ONLY if: [language_q2] = '2' and [hh5_covidsymp_s_q2] = '1'	Persona 5: ¿Cuáles de las siguientes medidas tomó esta persona para proteger a sus amigos y familiares después de que comenzaron sus síntomas?	checkbox<br>1 hh5_prevent_s_q2___1 Ponerse mascarilla con mayor frecuencia<br>2 hh5_prevent_s_q2___2 Lavarse las manos con agua y jabón con mayor frecuencia<br>3 hh5_prevent_s_q2___3 Limpiarse las manos con desinfectante con mayor frecuencia<br>4 hh5_prevent_s_q2___4 Aislarse en su casa con mayor frecuencia<br>5 hh5_prevent_s_q2___5 Quedarse en casa con mayor frecuencia<br>6 hh5_prevent_s_q2___6 Ponerse guantes desechables con mayor frecuencia<br>7 hh5_prevent_s_q2___7 No sabe
1982	[hh6_relationship_s_q2]<br><br>Show the field ONLY if: [language_q2] = '2' and [hhcount_s_q2] > 5 and [hhcount_s_q2] < 13	Section Header: *Por cada persona adicional en su hogar, proporcione la siguiente informacion.*<br><br>Persona 6: ¿Cuál es su relación con esta persona?	radio<br>1 Pareja o cónyuge<br>2 Hijo o hija<br>3 Padre o madre<br>4 Hermano o hermana<br>5 Otro familiar

			6 Proveedor de cuidado infantil u otros cuidados en casa<br>7 Otra<br><br>Field Annotation: @DEFAULT="[hh6_relationship_s_q2]"
1983	[hh6_relationship2_s_q2]<br><br>Show the field ONLY if: [language_q2] = '2' and [hh6_relationship_s_q2] = '7'	Persona 6: Especifique su relación con esta persona.	text<br>Field Annotation: @DEFAULT="[hh6_relationship2_s_q2]"
1984	[hh6_age_s_q2]<br><br>Show the field ONLY if: [language_q2] = '2' and [hhcount_s_q2] > 5 and [hhcount_s_q2] < 13	Persona 6: ¿Qué edad tiene esta persona? *(Especifique la edad en años)*	text (number, Min: 0, Max: 110)<br>Field Annotation: @DEFAULT="[hh6_age_s_q2]"
1985	[hh6_s_q2ex_s_q2]<br><br>Show the field ONLY if: [language_q2] = '2' and [hhcount_s_q2] > 5 and [hhcount_s_q2] < 13	Persona 6: ¿Cuál es el sexo de esta persona?	radio<br>1 Femenino<br>2 Masculino<br><br>Field Annotation: @DEFAULT="[hh6_s_q2ex_s_q2]"
1986	[hh6_race_s_q2]<br><br>Show the field ONLY if: [language_q2] = '2' and [hhcount_s_q2] > 5 and [hhcount_s_q2] < 13	Persona 6: ¿Cuál es la raza de esta persona? *(Seleccione todo lo que corresponda.)*	checkbox<br>1 hh6_race_s_q2___1 Indio americano o nativo de Alaska<br>2 hh6_race_s_q2___2 Asiático<br>3 hh6_race_s_q2___3 Negro o afroestadounidense<br>4 hh6_race_s_q2___4 Nativo de Hawái o de las islas del Pacífico<br>5 hh6_race_s_q2___5 Blanco<br>6 hh6_race_s_q2___6 Otra<br>7 hh6_race_s_q2___7 No sabe<br><br>Field Annotation: @DEFAULT="[hh6_race_s_q2]"
1987	[hh6_ethn_s_q2]<br><br>Show the field ONLY if: [language_q2] = '2' and [hhcount_s_q2] > 5 and [hhcount_s_q2] < 13	Persona 6: ¿Cuál es la identidad étnica de esta persona?	radio<br>1 Hispano o latino<br>2 Ni hispano ni latino<br>3 Otra<br>4 No sabe<br><br>Field Annotation: @DEFAULT="[hh6_ethn_s_q2]"

1988	[hh6_edu_s_q2]<br><br>Show the field ONLY if:<br>[language_q2] = '2' and [hhcount_s_q2] > 5 and [hhcount_s_q2] < 13	Persona 6: ¿Cuál es el nivel de educación de esta persona?	radio<br>1 No tiene educación formal<br>2 Kínder a 8° grado<br>3 Estudios de educación secundaria<br>4 Equivalencia de educación secundaria (GED)<br>5 Diploma de educación secundaria<br>6 Estudios de educación superior<br>7 Título universitario<br>8 Estudios de posgrado o más<br>9 No sabe<br><br>Field Annotation: @DEFAULT="[hh6_edu_s_q2]"
1989	[hh6_work_s_q2]<br><br>Show the field ONLY if:<br>[language_q2] = '2' and [hhcount_s_q2] > 5 and [hhcount_s_q2] < 13	Persona 6: ¿Cuál de las siguientes es la mejor descripción de la situación laboral actual de esta persona?	radio<br>1 Empleo a tiempo completo<br>2 Empleo a tiempo parcial<br>3 En busca de empleo<br>4 Jubilado<br>5 Ama de casa<br>6 Estudiante<br>7 Permiso de maternidad o paternidad<br>8 Permiso por enfermedad<br>9 Desempleado por discapacidad<br>10 Otra<br>11 No sabe<br><br>Field Annotation: @DEFAULT="[hh6_work_s_q2]"
1990	[hh6_work2_s_q2]<br><br>Show the field ONLY if:<br>[language_q2] = '2' and [hhcount_s_q2] > 5 and [hhcount_s_q2] < 13	Persona 6: ¿Se considera esta persona actualmente empleado por cuenta propia (contratista independiente, trabajador esporádico (gig), etc.)?	radio<br>1 Sí<br>0 No<br>2 No sabe<br><br>Field Annotation: @DEFAULT="[hh6_work2_s_q2]"
1991	[hh6_work3_s_q2]<br><br>Show the field ONLY if:<br>[language_q2] = '2' and [hhcount_s_q2] > 5 and [hhcount_s_q2] < 13 and ([hh6_work_s_q2] = '1' or [hh6_work_s_q2]	Persona 6: ¿Trabaja esta persona actualmente en alguno de los siguientes ambientes de alto riesgo de contagio de COVID-19?	checkbox<br>1 hh6_work3_s_q2___1 Ambiente de asistencia médica (hospital, clínica, centro

	= '2' or [hh6_work2_s_q2] = '1')		de urgencias, etc.)<br>2 | hh6_work3_s_q2___2 | Ambiente residencial denso (hogar de ancianos, otro centro de asistencia de larga duración)<br>3 | hh6_work3_s_q2___3 | Prisión o cárcel<br>4 | hh6_work3_s_q2___4 | Establecimiento de envasado de carne<br>5 | hh6_work3_s_q2___5 | Establecimiento de envío o distribución<br>6 | hh6_work3_s_q2___6 | Establecimiento minorista de alto volumen (tienda de provisiones, etc.)<br>7 | hh6_work3_s_q2___7 | No sabe<br><br>Field Annotation: @DEFAULT="[hh6_work3_s_q2]"
1992	[hh6_work4_s_q2]<br><br>Show the field ONLY if: [language_q2] = '2' and [hhcount_s_q2] > 5 and [hhcount_s_q2] < 13 and ([hh6_work_s_q2] = '1' or [hh6_work_s_q2] = '2' or [hh6_work2_s_q2] = '1')	Persona 6: ¿Le ofrece el empleador a esta persona alguno de los siguientes beneficios en su empleo principal actual?<br>*(Seleccione todo lo que corresponda.)*	checkbox<br>1 | hh6_work4_s_q2___1 | Permiso por enfermedad con goce de sueldo<br>2 | hh6_work4_s_q2___2 | Vacaciones o permiso personal con goce de sueldo<br>3 | hh6_work4_s_q2___3 | Seguro de salud<br>4 | hh6_work4_s_q2___4 | Seguro de discapacidad<br>5 | hh6_work4_s_q2___5 | Plan de jubilación<br>6 | hh6_work4_s_q2___6 | Otro<br>7 | hh6_work4_s_q2___7 | No sabe<br><br>Field Annotation: @DEFAULT="[hh6_work4_s_q2]"
1993	[hh6_work5_s_q2]<br><br>Show the field ONLY if: [language_q2] = '2' and [hhcount_s_q2] > 5 and	Persona 6: En una escala de 0 (definitivamente no va a suceder) a 10 (definitivamente va a suceder), ¿qué tan probable es que esta persona pierda su empleo debido a la pandemia de COVID-19?	text (number, Min: 0, Max: 10)<br>Field Annotation: @DEFAULT="[hh6_work5_s_q2]"

	[hhcount_s_q2] < 13 and ([hh6_work_s_q2] = '1' or [hh6_work_s_q2] = '2' or [hh6_work2_s_q2] = '1')		
1994	[hh6_work6_s_q2]<br><br>Show the field ONLY if: [language_q2] = '2' and [hhcount_s_q2] > 5 and [hhcount_s_q2] < 13 and ([hh6_work_s_q2] = '1' or [hh6_work_s_q2] = '2' or [hh6_work2_s_q2] = '1')	Persona 6: En una escala de 0 (definitivamente no va a suceder) a 10 (definitivamente va a suceder), ¿qué tan probable es que a esta persona se le asignen menos horas de trabajo debido a la pandemia de COVID-19?	text (number, Min: 0, Max: 10)<br>Field Annotation: @DEFAULT="[hh6_work6_s_q2]"
1995	[hh6_work7_s_q2]<br><br>Show the field ONLY if: [language_q2] = '2' and [hhcount_s_q2] > 5 and [hhcount_s_q2] < 13 and ([hh6_work_s_q2] = '1' or [hh6_work_s_q2] = '2' or [hh6_work2_s_q2] = '1')	Persona 6: Actualmente ¿con qué frecuencia se le exige a esta persona que trabaje fuera de su residencia?	radio (Matrix)<br>1 Todo el tiempo (100%)<br>2 La mayor parte del tiempo (75%)<br>3 La mitad del tiempo (50%)<br>4 Menos de la mitad del tiempo (25%)<br>5 nunca (0%)<br>6 Nunca
1996	[hh6_work8_s_q2]<br><br>Show the field ONLY if: [language_q2] = '2' and ([hh6_work7_s_q2] = '1' or [hh6_work7_s_q2] = '2' or [hh6_work7_s_q2] = '3' or [hh6_work7_s_q2] = '4')	Persona 6: Actualmente, ¿con qué frecuencia se encuentra esta persona físicamente cerca de sus compañeros de trabajo mientras trabaja fuera de su residencia?	radio (Matrix)<br>1 Todo el tiempo (100%)<br>2 La mayor parte del tiempo (75%)<br>3 La mitad del tiempo (50%)<br>4 Menos de la mitad del tiempo (25%)<br>5 nunca (0%)<br>6 Nunca
1997	[hh6_work9_s_q2]<br><br>Show the field ONLY if: [language_q2] = '2' and ([hh6_work7_s_q2] = '1' or [hh6_work7_s_q2] = '2' or [hh6_work7_s_q2] = '3' or [hh6_work7_s_q2] = '4')	Persona 6: Actualmente, ¿con qué frecuencia se encuentra esta persona físicamente cerca de sus clientes mientras trabaja fuera de su residencia	radio (Matrix)<br>1 Todo el tiempo (100%)<br>2 La mayor parte del tiempo (75%)<br>3 La mitad del tiempo (50%)<br>4 Menos de la mitad del tiempo (25%)<br>5 nunca (0%)<br>6 Nunca
1998	[hh6_covidvaccine_s_q2]<br><br>Show the field ONLY if: [language_q2] = '2' and [hhcount_s_q2] > 5 and [hhcount_s_q2] < 13	Persona 6: ¿Piensa esta persona vacunarse contra la COVID-19?	radio<br>1 Sí<br>0 No<br>2 No sabe<br>3 Este individuo ya ha sido vacunado<br><br>Field Annotation: @DEFAULT="[hh6_covidvaccine_s_q2]"

1999	[hh6_covidsymp_s_q2]<br><br>Show the field ONLY if:<br>[language_q2] = '2' and [hhcount_s_q2] > 5 and [hhcount_s_q2] < 13	Persona 6: En las últimas dos semanas, ¿ha tenido esta persona algún síntoma de COVID-19 (tos, fiebre, dificultad para respirar, fatiga, dolores de cuerpo, diarrea, goteo nasal o pérdida del sentido del olfato o del gusto)?	radio<br>1 Sí<br>0 No<br>2 No sabe
2000	[hh6_covidsymp2_s_q2]<br><br>Show the field ONLY if:<br>[language_q2] = '2' and [hh6_covidsymp_s_q2] = '1'	Persona 6: ¿Cuándo le comenzaron los síntomas de COVID-19 a esta persona?	text (date_mdy)
2001	[hh6_covidsymp3_s_q2]<br><br>Show the field ONLY if:<br>[language_q2] = '2' and [hh6_covidsymp_s_q2] = '1'	Persona 6: En vista de sus síntomas, ¿le preocupa a esta persona la posibilidad de tener COVID-19?	radio<br>1 Sí<br>0 No<br>2 No sabe
2002	[hh6_covidsymp4_s_q2]<br><br>Show the field ONLY if:<br>[language_q2] = '2' and [hh6_covidsymp_s_q2] = '1'	Persona 6: ¿Fue esta persona objeto de prejuicio o discriminación debido a sus síntomas?	radio<br>1 Sí<br>0 No<br>2 No sabe
2003	[hh6_covidsymp5_s_q2]<br><br>Show the field ONLY if:<br>[language_q2] = '2' and [hh6_covidsymp_s_q2] = '1'	Persona 6: ¿Qué hizo esta persona en vista de sus síntomas?<br>*(Seleccione todo lo que corresponda.)*	checkbox<br>0 hh6_covidsymp5_s_q2___0 Nada<br>1 hh6_covidsymp5_s_q2___1 Tomó medicament[illegible] sin receta (ibuprofeno, acetaminofé[illegible] etc.)<br>2 hh6_covidsymp5_s_q2___2 Consultó po[illegible] teléfono a u[illegible] proveedor d[illegible] asistencia médica<br>3 hh6_covidsymp5_s_q2___3 Fue al consultorio [illegible] un proveedo[illegible] de asistencia médica<br>4 hh6_covidsymp5_s_q2___4 Fue a una clínica o una farmacia minorista<br>5 hh6_covidsymp5_s_q2___5 Fue a un centro de urgencias (FASTMed, etc.)

			6 | hh6_covidsymp5_s_q2___6 | Fue a la sala de emergencias<br>7 | hh6_covidsymp5_s_q2___7 | Fue ingresad al hospital<br>8 | hh6_covidsymp5_s_q2___8 | Otra cosa<br>9 | hh6_covidsymp5_s_q2___9 | No sabe
2004	[hh6_covidsymp6_s_q2]<br>Show the field ONLY if: [language_q2] = '2' and [hh6_covidsymp5_s_q2(8)] = '1'	Persona 6: ¿Qué otra cosa hizo esta persona en vista de sus síntomas?	text
2005	[hh6_covidsymp7_s_q2]<br>Show the field ONLY if: [language_q2] = '2' and ([hh6_covidsymp5_s_q2(2)] = '1' or [hh6_covidsymp5_s_q2(3)] = '1' or [hh6_covidsymp5_s_q2(4)] = '1' or [hh6_covidsymp5_s_q2(5)] = '1' or [hh6_covidsymp5_s_q2(6)] = '1' or [hh6_covidsymp5_s_q2(7)] = '1' or [hh6_covidsymp5_s_q2(8)] = '1')	Persona 6: ¿Le dijo un proveedor de asistencia médica a esta persona que era posible que tuviera COVID-19?	radio<br>1 | Sí<br>0 | No<br>2 | No sabe
2006	[hh6_covid_test_s_q2]<br>Show the field ONLY if: [language_q2] = '2' and [hh6_covidsymp_s_q2] = '1'	Persona 6: Si a esta persona le hicieron una prueba de COVID-19 en vista de sus síntomas, ¿cuál fue el resultado?	radio<br>1 | Pendiente<br>2 | Positivo<br>3 | Negativo<br>4 | No concluyente<br>5 | No se hizo la prueba<br>6 | No sabe
2007	[hh6_covid_admit_s_q2]<br>Show the field ONLY if: [language_q2] = '2' and [hh6_covidsymp5_s_q2(7)] = '1'	Persona 6: ¿Cuántos días estuvo hospitalizada esta persona?	text (number, Min: 0)
2008	[hh6_covid_admit2_s_q2]<br>Show the field ONLY if: [language_q2] = '2' and [hh6_covidsymp5_s_q2(7)] = '1'	Persona 6: ¿Se le hicieron a esta persona las siguientes intervenciones durante su hospitalización?	checkbox<br>1 | hh6_covid_admit2_s_q2___1 | Oxígeno adicional por la nariz<br>2 | hh6_covid_admit2_s_q2___2 | Tratamiento en la Unidad de

			<table><tr><td></td><td></td><td>Cuidados Intensivos (Intensive Care Unit, ICU)</td></tr><tr><td>3</td><td>hh6_covid_admit2_s_q2___3</td><td>Ventilación mecánica (intubación o tubo de respiración)</td></tr><tr><td>4</td><td>hh6_covid_admit2_s_q2___4</td><td>No sabe</td></tr></table>
2009	[hh6_covidsymp8_s_q2]<br>Show the field ONLY if:<br>[language_q2] = '2' and [hh6_covidsymp_s_q2] = '1'	Persona 6: ¿Ha vuelto esta persona a su salud normal?	radio<br><table><tr><td>1</td><td>Sí</td></tr><tr><td>0</td><td>No</td></tr><tr><td>2</td><td>No sabe</td></tr></table>
2010	[hh6_prevent_s_q2]<br>Show the field ONLY if:<br>[language_q2] = '2' and [hh6_covidsymp_s_q2] = '1'	Persona 6: ¿Cuáles de las siguientes medidas tomó esta persona para proteger a sus amigos y familiares después de que comenzaron sus síntomas?	checkbox<br><table><tr><td>1</td><td>hh6_prevent_s_q2___1</td><td>Ponerse mascarilla con mayor frecuencia</td></tr><tr><td>2</td><td>hh6_prevent_s_q2___2</td><td>Lavarse las manos con agua y jabón con mayor frecuencia</td></tr><tr><td>3</td><td>hh6_prevent_s_q2___3</td><td>Limpiarse las manos con desinfectante con mayor frecuencia</td></tr><tr><td>4</td><td>hh6_prevent_s_q2___4</td><td>Aislarse en su casa con mayor frecuencia</td></tr><tr><td>5</td><td>hh6_prevent_s_q2___5</td><td>Quedarse en casa con mayor frecuencia</td></tr><tr><td>6</td><td>hh6_prevent_s_q2___6</td><td>Ponerse guantes desechables con mayor frecuencia</td></tr><tr><td>7</td><td>hh6_prevent_s_q2___7</td><td>No sabe</td></tr></table>
2011	[hh7_relationship_s_q2]<br>Show the field ONLY if:<br>[language_q2] = '2' and [hhcount_s_q2] > 6 and	Section Header: *Por cada persona adicional en su hogar, proporcione la siguiente informacion.*<br>Persona 7: ¿Cuál es su relación con esta persona?	radio<br><table><tr><td>1</td><td>Pareja o cónyuge</td></tr><tr><td>2</td><td>Hijo o hija</td></tr><tr><td>3</td><td>Padre o madre</td></tr></table>

	[hhcount_s_q2] < 13		4 Hermano o hermana<br>5 Otro familiar<br>6 Proveedor de cuidado infantil u otros cuidados en casa<br>7 Otra<br><br>Field Annotation: @DEFAULT=" [hh7_relationship_s_q2]"
2012	[hh7_relationship2_s_q2]<br><br>Show the field ONLY if: [language_q2] = '2' and [hh7_relationship_s_q2] = '7'	Persona 7: Especifique su relación con esta persona.	text<br>Field Annotation: @DEFAULT=" [hh7_relationship2_s_q2]"
2013	[hh7_age_s_q2]<br><br>Show the field ONLY if: [language_q2] = '2' and [hhcount_s_q2] > 6 and [hhcount_s_q2] < 13	Persona 7: ¿Qué edad tiene esta persona?<br>*(Especifique la edad en años)*	text (number, Min: 0, Max: 110)<br>Field Annotation: @DEFAULT=" [hh7_age_s_q2]"
2014	[hh7_s_q2ex_s_q2]<br><br>Show the field ONLY if: [language_q2] = '2' and [hhcount_s_q2] > 6 and [hhcount_s_q2] < 13	Persona 7: ¿Cuál es el sexo de esta persona?	radio<br>1 Femenino<br>2 Masculino<br><br>Field Annotation: @DEFAULT=" [hh7_s_q2ex_s_q2]"
2015	[hh7_race_s_q2]<br><br>Show the field ONLY if: [language_q2] = '2' and [hhcount_s_q2] > 6 and [hhcount_s_q2] < 13	Persona 7: ¿Cuál es la raza de esta persona?<br>*(Seleccione todo lo que corresponda.)*	checkbox<br>1 hh7_race_s_q2___1 Indio americano o nativo de Alaska<br>2 hh7_race_s_q2___2 Asiático<br>3 hh7_race_s_q2___3 Negro o afroestadounidense<br>4 hh7_race_s_q2___4 Nativo de Hawái o de las islas del Pacífico<br>5 hh7_race_s_q2___5 Blanco<br>6 hh7_race_s_q2___6 Otra<br>7 hh7_race_s_q2___7 No sabe<br><br>Field Annotation: @DEFAULT=" [hh7_race_s_q2]"
2016	[hh7_ethn_s_q2]<br><br>Show the field ONLY if: [language_q2] = '2' and [hhcount_s_q2] > 6 and [hhcount_s_q2] < 13	Persona 7: ¿Cuál es la identidad étnica de esta persona?	radio<br>1 Hispano o latino<br>2 Ni hispano ni latino<br>3 Otra<br>4 No sabe

			Field Annotation: @DEFAULT="[hh7_ethn_s_q2]"
2017	[hh7_edu_s_q2]<br>Show the field ONLY if:<br>[language_q2] = '2' and [hhcount_s_q2] > 6 and [hhcount_s_q2] < 13	Persona 7: ¿Cuál es el nivel de educación de esta persona?	radio<br>1 No tiene educación formal<br>2 Kínder a 8° grado<br>3 Estudios de educación secundaria<br>4 Equivalencia de educación secundaria (GED)<br>5 Diploma de educación secundaria<br>6 Estudios de educación superior<br>7 Título universitario<br>8 Estudios de posgrado o más<br>9 No sabe<br>Field Annotation: @DEFAULT="[hh7_edu_s_q2]"
2018	[hh7_work_s_q2]<br>Show the field ONLY if:<br>[language_q2] = '2' and [hhcount_s_q2] > 6 and [hhcount_s_q2] < 13	Persona 7: ¿Cuál de las siguientes es la mejor descripción de la situación laboral actual de esta persona?	radio<br>1 Empleo a tiempo completo<br>2 Empleo a tiempo parcial<br>3 En busca de empleo<br>4 Jubilado<br>5 Ama de casa<br>6 Estudiante<br>7 Permiso de maternidad o paternidad<br>8 Permiso por enfermedad<br>9 Desempleado por discapacidad<br>10 Otra<br>11 No sabe<br>Field Annotation: @DEFAULT="[hh7_work_s_q2]"
2019	[hh7_work2_s_q2]<br>Show the field ONLY if:<br>[language_q2] = '2' and [hhcount_s_q2] > 6 and [hhcount_s_q2] < 13	Persona 7: ¿Se considera esta persona actualmente empleado por cuenta propia (contratista independiente, trabajador esporádico (gig), etc.)?	radio<br>1 Sí<br>0 No<br>2 No sabe<br>Field Annotation: @DEFAULT="[hh7_work2_s_q2]"
2020	[hh7_work3_s_q2]<br>Show the field ONLY if:<br>[language_q2] = '2' and [hhcount_s_q2] > 6 and	Persona 7: ¿Trabaja esta persona actualmente en alguno de los siguientes ambientes de alto riesgo de contagio de COVID-19?	checkbox<br>1 hh7_work3_s_q2___1 Ambiente de asistencia médica

	[hhcount_s_q2] < 13 and ([hh7_work_s_q2] = '1' or [hh7_work_s_q2] = '2' or [hh7_work2_s_q2] = '1')		(hospital, clínica, centro de urgencias, etc.)<br>2 | hh7_work3_s_q2___2 | Ambiente residencial denso (hogar de ancianos, otro centro de asistencia de larga duración)<br>3 | hh7_work3_s_q2___3 | Prisión o cárcel<br>4 | hh7_work3_s_q2___4 | Establecimiento de envasado de carne<br>5 | hh7_work3_s_q2___5 | Establecimiento de envío o distribución<br>6 | hh7_work3_s_q2___6 | Establecimiento minorista de alto volumen (tienda de provisiones, etc.)<br>7 | hh7_work3_s_q2___7 | No sabe<br><br>Field Annotation: @DEFAULT=" [hh7_work3_s_q2]"
2021	[hh7_work4_s_q2]<br><br>Show the field ONLY if:<br>[language_q2] = '2' and [hhcount_s_q2] > 6 and [hhcount_s_q2] < 13 and ([hh7_work_s_q2] = '1' or [hh7_work_s_q2] = '2' or [hh7_work2_s_q2] = '1')	Persona 7: ¿Le ofrece el empleador a esta persona alguno de los siguientes beneficios en su empleo principal actual?<br>*(Seleccione todo lo que corresponda.)*	checkbox<br>1 | hh7_work4_s_q2___1 | Permiso por enfermedad con goce de sueldo<br>2 | hh7_work4_s_q2___2 | Vacaciones o permiso personal con goce de sueldo<br>3 | hh7_work4_s_q2___3 | Seguro de salud<br>4 | hh7_work4_s_q2___4 | Seguro de discapacidad<br>5 | hh7_work4_s_q2___5 | Plan de jubilación<br>6 | hh7_work4_s_q2___6 | Otro<br>7 | hh7_work4_s_q2___7 | No sabe<br><br>Field Annotation: @DEFAULT=" [hh7_work4_s_q2]"
2022	[hh7_work5_s_q2]<br><br>Show the field ONLY if:	Persona 7: En una escala de 0 (definitivamente no va a suceder) a 10 (definitivamente va a suceder),	text (number, Min: 0, Max: 10)<br>Field Annotation: @DEFAULT="

	[language_q2] = '2' and [hhcount_s_q2] > 6 and [hhcount_s_q2] < 13 an d ([hh7_work_s_q2] = '1' or [hh7_work_s_q2] = '2' or [hh7_work2_s_q 2] = '1')	¿qué tan probable es que esta persona pierda su empleo debido a la pandemia de COVID-19?	[hh7_work5_s_q2]"
2023	[hh7_work6_s_q2]<br><br>Show the field ONLY if: [language_q2] = '2' and [hhcount_s_q2] > 6 and [hhcount_s_q2] < 13 an d ([hh7_work_s_q2] = '1' or [hh7_work_s_q2] = '2' or [hh7_work2_s_q 2] = '1')	Persona 7: En una escala de 0 (definitivamente no va a suceder) a 10 (definitivamente va a suceder), ¿qué tan probable es que a esta persona se le asignen menos horas de trabajo debido a la pandemia de COVID-19?	text (number, Min: 0, Max: 10)<br>Field Annotation: @DEFAULT="[hh7_work6_s_q2]"
2024	[hh7_work7_s_q2]<br><br>Show the field ONLY if: [language_q2] = '2' and [hhcount_s_q2] > 6 and [hhcount_s_q2] < 13 an d ([hh7_work_s_q2] = '1' or [hh7_work_s_q2] = '2' or [hh7_work2_s_q 2] = '1')	Persona 7: Actualmente ¿con qué frecuencia se le exige a esta persona que trabaje fuera de su residencia?	radio (Matrix)<br>1 Todo el tiempo (100%)<br>2 La mayor parte del tiempo (75%)<br>3 La mitad del tiempo (50%)<br>4 Menos de la mitad del tiempo (25%)<br>5 nunca (0%)<br>6 Nunca
2025	[hh7_work8_s_q2]<br><br>Show the field ONLY if: [language_q2] = '2' and ([hh7_work7_s_q2] = '1' or [hh7_work7_s_q2] = '2' or [hh7_work7_s_q2] = '3' or [hh7_work7_s_q 2] = '4')	Persona 7: Actualmente, ¿con qué frecuencia se encuentra esta persona físicamente cerca de sus compañeros de trabajo mientras trabaja fuera de su residencia?	radio (Matrix)<br>1 Todo el tiempo (100%)<br>2 La mayor parte del tiempo (75%)<br>3 La mitad del tiempo (50%)<br>4 Menos de la mitad del tiempo (25%)<br>5 nunca (0%)<br>6 Nunca
2026	[hh7_work9_s_q2]<br><br>Show the field ONLY if: [language_q2] = '2' and ([hh7_work7_s_q2] = '1' or [hh7_work7_s_q2] = '2' or [hh7_work7_s_q2] = '3' or [hh7_work7_s_q 2] = '4')	Persona 7: Actualmente, ¿con qué frecuencia se encuentra esta persona físicamente cerca de sus clientes mientras trabaja fuera de su residencia	radio (Matrix)<br>1 Todo el tiempo (100%)<br>2 La mayor parte del tiempo (75%)<br>3 La mitad del tiempo (50%)<br>4 Menos de la mitad del tiempo (25%)<br>5 nunca (0%)<br>6 Nunca
2027	[hh7_covidvaccine_s _q2]<br><br>Show the field ONLY if: [language_q2] = '2' and [hhcount_s_q2] > 6 and [hhcount_s_q2] < 13	Persona 7: ¿Piensa esta persona vacunarse contra la COVID-19?	radio<br>1 Sí<br>0 No<br>2 No sabe<br>3 Este individuo ya ha sido vacunado<br><br>Field Annotation: @DEFAULT="

			[hh7_covidvaccine_s_q2]"
2028	[hh7_covidsymp_s_q2]<br><br>Show the field ONLY if:<br>[language_q2] = '2' and<br>[hhcount_s_q2] > 6 and<br>[hhcount_s_q2] < 13	Persona 7: En las últimas dos semanas, ¿ha tenido esta persona algún síntoma de COVID-19 (tos, fiebre, dificultad para respirar, fatiga, dolores de cuerpo, diarrea, goteo nasal o pérdida del sentido del olfato o del gusto)?	radio<br>1 Sí<br>0 No<br>2 No sabe
2029	[hh7_covidsymp2_s_q2]<br><br>Show the field ONLY if:<br>[language_q2] = '2' and<br>[hh7_covidsymp_s_q2] = '1'	Persona 7: ¿Cuándo le comenzaron los síntomas de COVID-19 a esta persona?	text (date_mdy)
2030	[hh7_covidsymp3_s_q2]<br><br>Show the field ONLY if:<br>[language_q2] = '2' and<br>[hh7_covidsymp_s_q2] = '1'	Persona 7: En vista de sus síntomas, ¿le preocupa a esta persona la posibilidad de tener COVID-19?	radio<br>1 Sí<br>0 No<br>2 No sabe
2031	[hh7_covidsymp4_s_q2]<br><br>Show the field ONLY if:<br>[language_q2] = '2' and<br>[hh7_covidsymp_s_q2] = '1'	Persona 7: ¿Fue esta persona objeto de prejuicio o discriminación debido a sus síntomas?	radio<br>1 Sí<br>0 No<br>2 No sabe
2032	[hh7_covidsymp5_s_q2]<br><br>Show the field ONLY if:<br>[language_q2] = '2' and<br>[hh7_covidsymp_s_q2] = '1'	Persona 7: ¿Qué hizo esta persona en vista de sus síntomas?<br>*(Seleccione todo lo que corresponda.)*	checkbox<br>0 hh7_covidsymp5_s_q2___0 Nada<br>1 hh7_covidsymp5_s_q2___1 Tomó medicament[illegible] sin receta (ibuprofeno, acetaminofé[illegible] etc.)<br>2 hh7_covidsymp5_s_q2___2 Consultó po[illegible] teléfono a u[illegible] proveedor d[illegible] asistencia médica<br>3 hh7_covidsymp5_s_q2___3 Fue al consultorio [illegible] un proveedo[illegible] de asistencia médica<br>4 hh7_covidsymp5_s_q2___4 Fue a una clínica o una farmacia minorista<br>5 hh7_covidsymp5_s_q2___5 Fue a un centro de urgencias

			(FASTMed, etc.)<br>6 | hh7_covidsymp5_s_q2___6 | Fue a la sala de emergencias<br>7 | hh7_covidsymp5_s_q2___7 | Fue ingresad al hospital<br>8 | hh7_covidsymp5_s_q2___8 | Otra cosa<br>9 | hh7_covidsymp5_s_q2___9 | No sabe
2033	[hh7_covidsymp6_s_q2]<br><br>Show the field ONLY if: [language_q2] = '2' and [hh7_covidsymp5_s_q2(8)] = '1'	Persona 7: ¿Qué otra cosa hizo esta persona en vista de sus síntomas?	text
2034	[hh7_covidsymp7_s_q2]<br><br>Show the field ONLY if: [language_q2] = '2' and ([hh7_covidsymp5_s_q2(2)] = '1' or [hh7_covidsymp5_s_q2(3)] = '1' or [hh7_covidsymp5_s_q2(4)] = '1' or [hh7_covidsymp5_s_q2(5)] = '1' or [hh7_covidsymp5_s_q2(6)] = '1' or [hh7_covidsymp5_s_q2(7)] = '1' or [hh7_covidsymp5_s_q2(8)] = '1')	Persona 7: ¿Le dijo un proveedor de asistencia médica a esta persona que era posible que tuviera COVID-19?	radio<br>1 | Sí<br>0 | No<br>2 | No sabe
2035	[hh7_covid_test_s_q2]<br><br>Show the field ONLY if: [language_q2] = '2' and [hh7_covidsymp_s_q2] = '1'	Persona 7: Si a esta persona le hicieron una prueba de COVID-19 en vista de sus síntomas, ¿cuál fue el resultado?	radio<br>1 | Pendiente<br>2 | Positivo<br>3 | Negativo<br>4 | No concluyente<br>5 | No se hizo la prueba<br>6 | No sabe
2036	[hh7_covid_admit_s_q2]<br><br>Show the field ONLY if: [language_q2] = '2' and [hh7_covidsymp5_s_q2(7)] = '1'	Persona 7: ¿Cuántos días estuvo hospitalizada esta persona?	text (number, Min: 0)
2037	[hh7_covid_admit2_s_q2]<br><br>Show the field ONLY if: [language_q2] = '2' and [hh7_covidsymp5_s_q2	Persona 7: ¿Se le hicieron a esta persona las siguientes intervenciones durante su hospitalización?	checkbox<br>1 | hh7_covid_admit2_s_q2___1 | Oxígeno adicional por la nariz

	(7)] = '1'		2 hh7_covid_admit2_s_q2___2 Tratamiento en la Unidad de Cuidados Intensivos (Intensive Care Unit, ICU)<br>3 hh7_covid_admit2_s_q2___3 Ventilación mecánica (intubación o tubo de respiración)<br>4 hh7_covid_admit2_s_q2___4 No sabe
2038	[hh7_covidsymp8_s_q2]<br><br>Show the field ONLY if: [language_q2] = '2' and [hh7_covidsymp_s_q2] = '1'	Persona 7: ¿Ha vuelto esta persona a su salud normal?	radio<br>1 Sí<br>0 No<br>2 No sabe
2039	[hh7_prevent_s_q2]<br><br>Show the field ONLY if: [language_q2] = '2' and [hh7_covidsymp_s_q2] = '1'	Persona 7: ¿Cuáles de las siguientes medidas tomó esta persona para proteger a sus amigos y familiares después de que comenzaron sus síntomas?	checkbox<br>1 hh7_prevent_s_q2___1 Ponerse mascarilla con mayor frecuencia<br>2 hh7_prevent_s_q2___2 Lavarse las manos con agua y jabón con mayor frecuencia<br>3 hh7_prevent_s_q2___3 Limpiarse las manos con desinfectante con mayor frecuencia<br>4 hh7_prevent_s_q2___4 Aislarse en su casa con mayor frecuencia<br>5 hh7_prevent_s_q2___5 Quedarse en casa con mayor frecuencia<br>6 hh7_prevent_s_q2___6 Ponerse guantes desechables con mayor frecuencia<br>7 hh7_prevent_s_q2___7 No sabe
2040	[hh8_relationship_s_q2]	Section Header: *Por cada persona adicional en su hogar, proporcione la siguiente informacion.*	radio

	Show the field ONLY if: [language_q2] = '2' and [hhcount_s_q2] > 7 and [hhcount_s_q2] < 13	Persona 8: ¿Cuál es su relación con esta persona?	1 Pareja o cónyuge<br>2 Hijo o hija<br>3 Padre o madre<br>4 Hermano o hermana<br>5 Otro familiar<br>6 Proveedor de cuidado infantil u otros cuidados en casa<br>7 Otra<br><br>Field Annotation: @DEFAULT="[hh8_relationship_s_q2]"
2041	[hh8_relationship2_s_q2]<br><br>Show the field ONLY if: [language_q2] = '2' and [hh8_relationship_s_q2] = '7'	Persona 8: Especifique su relación con esta persona.	text<br>Field Annotation: @DEFAULT="[hh8_relationship2_s_q2]"
2042	[hh8_age_s_q2]<br><br>Show the field ONLY if: [language_q2] = '2' and [hhcount_s_q2] > 7 and [hhcount_s_q2] < 13	Persona 8: ¿Qué edad tiene esta persona?<br>*(Especifique la edad en años)*	text (number, Min: 0, Max: 110)<br>Field Annotation: @DEFAULT="[hh8_age_s_q2]"
2043	[hh8_s_q2ex_s_q2]<br><br>Show the field ONLY if: [language_q2] = '2' and [hhcount_s_q2] > 7 and [hhcount_s_q2] < 13	Persona 8: ¿Cuál es el sexo de esta persona?	radio<br>1 Femenino<br>2 Masculino<br><br>Field Annotation: @DEFAULT="[hh8_s_q2ex_s_q2]"
2044	[hh8_race_s_q2]<br><br>Show the field ONLY if: [language_q2] = '2' and [hhcount_s_q2] > 7 and [hhcount_s_q2] < 13	Persona 8: ¿Cuál es la raza de esta persona?<br>*(Seleccione todo lo que corresponda.)*	checkbox<br>1 hh8_race_s_q2___1 Indio americano o nativo de Alaska<br>2 hh8_race_s_q2___2 Asiático<br>3 hh8_race_s_q2___3 Negro o afroestadounidense<br>4 hh8_race_s_q2___4 Nativo de Hawái o de las islas del Pacífico<br>5 hh8_race_s_q2___5 Blanco<br>6 hh8_race_s_q2___6 Otra<br>7 hh8_race_s_q2___7 No sabe<br><br>Field Annotation: @DEFAULT="[hh8_race_s_q2]"
2045	[hh8_ethn_s_q2]<br><br>Show the field ONLY if:	Persona 8: ¿Cuál es la identidad étnica de esta persona?	radio<br>1 Hispano o latino

	[language_q2] = '2' and [hhcount_s_q2] > 7 and [hhcount_s_q2] < 13		2 Ni hispano ni latino<br>3 Otra<br>4 No sabe<br><br>Field Annotation: @DEFAULT=" [hh8_ethn_s_q2]"
2046	[hh8_edu_s_q2]<br><br>Show the field ONLY if: [language_q2] = '2' and [hhcount_s_q2] > 7 and [hhcount_s_q2] < 13	Persona 8: ¿Cuál es el nivel de educación de esta persona?	radio<br>1 No tiene educación formal<br>2 Kínder a 8° grado<br>3 Estudios de educación secundaria<br>4 Equivalencia de educación secundaria (GED)<br>5 Diploma de educación secundaria<br>6 Estudios de educación superior<br>7 Título universitario<br>8 Estudios de posgrado o más<br>9 No sabe<br><br>Field Annotation: @DEFAULT=" [hh8_edu_s_q2]"
2047	[hh8_work_s_q2]<br><br>Show the field ONLY if: [language_q2] = '2' and [hhcount_s_q2] > 7 and [hhcount_s_q2] < 13	Persona 8: ¿Cuál de las siguientes es la mejor descripción de la situación laboral actual de esta persona?	radio<br>1 Empleo a tiempo completo<br>2 Empleo a tiempo parcial<br>3 En busca de empleo<br>4 Jubilado<br>5 Ama de casa<br>6 Estudiante<br>7 Permiso de maternidad o paternidad<br>8 Permiso por enfermedad<br>9 Desempleado por discapacidad<br>10 Otra<br>11 No sabe<br><br>Field Annotation: @DEFAULT=" [hh8_work_s_q2]"
2048	[hh8_work2_s_q2]<br><br>Show the field ONLY if: [language_q2] = '2' and [hhcount_s_q2] > 7 and [hhcount_s_q2] < 13	Persona 8: ¿Se considera esta persona actualmente empleado por cuenta propia (contratista independiente, trabajador esporádico (gig), etc.)?	radio<br>1 Sí<br>0 No<br>2 No sabe<br><br>Field Annotation: @DEFAULT=" [hh8_work2_s_q2]"

2049	[hh8_work3_s_q2]<br><br>Show the field ONLY if:<br>[language_q2] = '2' and [hhcount_s_q2] > 7 and [hhcount_s_q2] < 13 and ([hh8_work_s_q2] = '1' or [hh8_work_s_q2] = '2' or [hh8_work2_s_q2] = '1')	Persona 8: ¿Trabaja esta persona actualmente en alguno de los siguientes ambientes de alto riesgo de contagio de COVID-19?	checkbox<br>1 hh8_work3_s_q2___1 Ambiente de asistencia médica (hospital, clínica, centro de urgencias, etc.)<br>2 hh8_work3_s_q2___2 Ambiente residencial denso (hogar de ancianos, otro centro de asistencia de larga duración)<br>3 hh8_work3_s_q2___3 Prisión o cárcel<br>4 hh8_work3_s_q2___4 Establecimiento de envasado de carne<br>5 hh8_work3_s_q2___5 Establecimiento de envío o distribución<br>6 hh8_work3_s_q2___6 Establecimiento minorista de alto volumen (tienda de provisiones, etc.)<br>7 hh8_work3_s_q2___7 No sabe<br><br>Field Annotation: @DEFAULT="[hh8_work3_s_q2]"
2050	[hh8_work4_s_q2]<br><br>Show the field ONLY if:<br>[language_q2] = '2' and [hhcount_s_q2] > 7 and [hhcount_s_q2] < 13 and ([hh8_work_s_q2] = '1' or [hh8_work_s_q2] = '2' or [hh8_work2_s_q2] = '1')	Persona 8: ¿Le ofrece el empleador a esta persona alguno de los siguientes beneficios en su empleo principal actual?<br>*(Seleccione todo lo que corresponda.)*	checkbox<br>1 hh8_work4_s_q2___1 Permiso por enfermedad con goce de sueldo<br>2 hh8_work4_s_q2___2 Vacaciones o permiso personal con goce de sueldo<br>3 hh8_work4_s_q2___3 Seguro de salud<br>4 hh8_work4_s_q2___4 Seguro de discapacidad<br>5 hh8_work4_s_q2___5 Plan de jubilación<br>6 hh8_work4_s_q2___6 Otro<br>7 hh8_work4_s_q2___7 No sabe

			Field Annotation: @DEFAULT="[hh8_work4_s_q2]"
2051	[hh8_work5_s_q2]<br><br>Show the field ONLY if:<br>[language_q2] = '2' and [hhcount_s_q2] > 7 and [hhcount_s_q2] < 13 and ([hh8_work_s_q2] = '1' or [hh8_work_s_q2] = '2' or [hh8_work2_s_q2] = '1')	Persona 8: En una escala de 0 (definitivamente no va a suceder) a 10 (definitivamente va a suceder), ¿qué tan probable es que esta persona pierda su empleo debido a la pandemia de COVID-19?	text (number, Min: 0, Max: 10)<br>Field Annotation: @DEFAULT="[hh8_work5_s_q2]"
2052	[hh8_work6_s_q2]<br><br>Show the field ONLY if:<br>[language_q2] = '2' and [hhcount_s_q2] > 7 and [hhcount_s_q2] < 13 and ([hh8_work_s_q2] = '1' or [hh8_work_s_q2] = '2' or [hh8_work2_s_q2] = '1')	Persona 8: En una escala de 0 (definitivamente no va a suceder) a 10 (definitivamente va a suceder), ¿qué tan probable es que a esta persona se le asignen menos horas de trabajo debido a la pandemia de COVID-19?	text (number, Min: 0, Max: 10)<br>Field Annotation: @DEFAULT="[hh8_work6_s_q2]"
2053	[hh8_work7_s_q2]<br><br>Show the field ONLY if:<br>[language_q2] = '2' and [hhcount_s_q2] > 7 and [hhcount_s_q2] < 13 and ([hh8_work_s_q2] = '1' or [hh8_work_s_q2] = '2' or [hh8_work2_s_q2] = '1')	Persona 8: Actualmente ¿con qué frecuencia se le exige a esta persona que trabaje fuera de su residencia?	radio (Matrix)<br>1 Todo el tiempo (100%)<br>2 La mayor parte del tiempo (75%)<br>3 La mitad del tiempo (50%)<br>4 Menos de la mitad del tiempo (25%)<br>5 nunca (0%)<br>6 Nunca
2054	[hh8_work8_s_q2]<br><br>Show the field ONLY if:<br>[language_q2] = '2' and ([hh8_work7_s_q2] = '1' or [hh8_work7_s_q2] = '2' or [hh8_work7_s_q2] = '3' or [hh8_work7_s_q2] = '4')	Persona 8: Actualmente, ¿con qué frecuencia se encuentra esta persona físicamente cerca de sus compañeros de trabajo mientras trabaja fuera de su residencia?	radio (Matrix)<br>1 Todo el tiempo (100%)<br>2 La mayor parte del tiempo (75%)<br>3 La mitad del tiempo (50%)<br>4 Menos de la mitad del tiempo (25%)<br>5 nunca (0%)<br>6 Nunca
2055	[hh8_work9_s_q2]<br><br>Show the field ONLY if:<br>[language_q2] = '2' and ([hh8_work7_s_q2] = '1' or [hh8_work7_s_q2] = '2' or [hh8_work7_s_q2] = '3' or [hh8_work7_s_q2] = '4')	Persona 8: Actualmente, ¿con qué frecuencia se encuentra esta persona físicamente cerca de sus clientes mientras trabaja fuera de su residencia	radio (Matrix)<br>1 Todo el tiempo (100%)<br>2 La mayor parte del tiempo (75%)<br>3 La mitad del tiempo (50%)<br>4 Menos de la mitad del tiempo (25%)<br>5 nunca (0%)<br>6 Nunca
2056	[hh8_covidvaccine_s_q2]	Persona 8: ¿Piensa esta persona vacunarse contra la COVID-19?	radio<br>1 Sí

	Show the field ONLY if:<br>[language_q2] = '2' and<br>[hhcount_s_q2] > 7 and<br>[hhcount_s_q2] < 13		0 | No<br>2 | No sabe<br>3 | Este individuo ya ha sido vacunado<br><br>Field Annotation: @DEFAULT="[hh8_covidvaccine_s_q2]"
2057	[hh8_covidsymp_s_q2]<br><br>Show the field ONLY if:<br>[language_q2] = '2' and<br>[hhcount_s_q2] > 7 and<br>[hhcount_s_q2] < 13	Persona 8: En las últimas dos semanas, ¿ha tenido esta persona algún síntoma de COVID-19 (tos, fiebre, dificultad para respirar, fatiga, dolores de cuerpo, diarrea, goteo nasal o pérdida del sentido del olfato o del gusto)?	radio<br>1 | Sí<br>0 | No<br>2 | No sabe
2058	[hh8_covidsymp2_s_q2]<br><br>Show the field ONLY if:<br>[language_q2] = '2' and<br>[hh8_covidsymp_s_q2] = '1'	Persona 8: ¿Cuándo le comenzaron los síntomas de COVID-19 a esta persona?	text (date_mdy)
2059	[hh8_covidsymp3_s_q2]<br><br>Show the field ONLY if:<br>[language_q2] = '2' and<br>[hh8_covidsymp_s_q2] = '1'	Persona 8: En vista de sus síntomas, ¿le preocupa a esta persona la posibilidad de tener COVID-19?	radio<br>1 | Sí<br>0 | No<br>2 | No sabe
2060	[hh8_covidsymp4_s_q2]<br><br>Show the field ONLY if:<br>[language_q2] = '2' and<br>[hh8_covidsymp_s_q2] = '1'	Persona 8: ¿Fue esta persona objeto de prejuicio o discriminación debido a sus síntomas?	radio<br>1 | Sí<br>0 | No<br>2 | No sabe
2061	[hh8_covidsymp5_s_q2]<br><br>Show the field ONLY if:<br>[language_q2] = '2' and<br>[hh8_covidsymp_s_q2] = '1'	Persona 8: ¿Qué hizo esta persona en vista de sus síntomas?<br>*(Seleccione todo lo que corresponda.)*	checkbox<br>0 | hh8_covidsymp5_s_q2___0 | Nada<br>1 | hh8_covidsymp5_s_q2___1 | Tomó medicament sin receta (ibuprofeno, acetaminofé etc.)<br>2 | hh8_covidsymp5_s_q2___2 | Consultó po teléfono a ur proveedor de asistencia médica<br>3 | hh8_covidsymp5_s_q2___3 | Fue al consultorio c un proveedo de asistencia médica<br>4 | hh8_covidsymp5_s_q2___4 | Fue a una clínica o una

			farmacia minorista<br>5 | hh8_covidsymp5_s_q2___5 | Fue a un centro de urgencias (FASTMed, etc.)<br>6 | hh8_covidsymp5_s_q2___6 | Fue a la sala de emergencias<br>7 | hh8_covidsymp5_s_q2___7 | Fue ingresado al hospital<br>8 | hh8_covidsymp5_s_q2___8 | Otra cosa<br>9 | hh8_covidsymp5_s_q2___9 | No sabe
2062	[hh8_covidsymp6_s_q2]<br>Show the field ONLY if: [language_q2] = '2' and [hh8_covidsymp5_s_q2(8)] = '1'	Persona 8: ¿Qué otra cosa hizo esta persona en vista de sus síntomas?	text
2063	[hh8_covidsymp7_s_q2]<br>Show the field ONLY if: [language_q2] = '2' and ([hh8_covidsymp5_s_q2(2)] = '1' or [hh8_covidsymp5_s_q2(3)] = '1' or [hh8_covidsymp5_s_q2(4)] = '1' or [hh8_covidsymp5_s_q2(5)] = '1' or [hh8_covidsymp5_s_q2(6)] = '1' or [hh8_covidsymp5_s_q2(7)] = '1' or [hh8_covidsymp5_s_q2(8)] = '1')	Persona 8: ¿Le dijo un proveedor de asistencia médica a esta persona que era posible que tuviera COVID-19?	radio<br>1 | Sí<br>0 | No<br>2 | No sabe
2064	[hh8_covid_test_s_q2]<br>Show the field ONLY if: [language_q2] = '2' and [hh8_covidsymp_s_q2] = '1'	Persona 8: Si a esta persona le hicieron una prueba de COVID-19 en vista de sus síntomas, ¿cuál fue el resultado?	radio<br>1 | Pendiente<br>2 | Positivo<br>3 | Negativo<br>4 | No concluyente<br>5 | No se hizo la prueba<br>6 | No sabe
2065	[hh8_covid_admit_s_q2]<br>Show the field ONLY if: [language_q2] = '2' and [hh8_covidsymp5_s_q2(7)] = '1'	Persona 8: ¿Cuántos días estuvo hospitalizada esta persona?	text (number, Min: 0)

2066	[hh8_covid_admit2_s_q2]<br>Show the field ONLY if: [language_q2] = '2' and [hh8_covidsymp5_s_q2(7)] = '1'	Persona 8: ¿Se le hicieron a esta persona las siguientes intervenciones durante su hospitalización?	checkbox<br>1 hh8_covid_admit2_s_q2___1 Oxígeno adicional por la nariz<br>2 hh8_covid_admit2_s_q2___2 Tratamiento en la Unidad de Cuidados Intensivos (Intensive Care Unit, ICU)<br>3 hh8_covid_admit2_s_q2___3 Ventilación mecánica (intubación o tubo de respiración)<br>4 hh8_covid_admit2_s_q2___4 No sabe
2067	[hh8_covidsymp8_s_q2]<br>Show the field ONLY if: [language_q2] = '2' and [hh8_covidsymp_s_q2] = '1'	Persona 8: ¿Ha vuelto esta persona a su salud normal?	radio<br>1 Sí<br>0 No<br>2 No sabe
2068	[hh8_prevent_s_q2]<br>Show the field ONLY if: [language_q2] = '2' and [hh8_covidsymp_s_q2] = '1'	Persona 8: ¿Cuáles de las siguientes medidas tomó esta persona para proteger a sus amigos y familiares después de que comenzaron sus síntomas?	checkbox<br>1 hh8_prevent_s_q2___1 Ponerse mascarilla con mayor frecuencia<br>2 hh8_prevent_s_q2___2 Lavarse las manos con agua y jabón con mayor frecuencia<br>3 hh8_prevent_s_q2___3 Limpiarse las manos con desinfectante con mayor frecuencia<br>4 hh8_prevent_s_q2___4 Aislarse en su casa con mayor frecuencia<br>5 hh8_prevent_s_q2___5 Quedarse en casa con mayor frecuencia<br>6 hh8_prevent_s_q2___6 Ponerse guantes desechables

			con mayor frecuencia<br>7 | hh8_prevent_s_q2___7 | No sabe
2069	[hh9_relationship_s_q2]<br><br>Show the field ONLY if: [language_q2] = '2' and [hhcount_s_q2] > 8 and [hhcount_s_q2] < 13	Section Header: *Por cada persona adicional en su hogar, proporcione la siguiente informacion.*<br><br>Persona 9: ¿Cuál es su relación con esta persona?	radio<br>1 | Pareja o cónyuge<br>2 | Hijo o hija<br>3 | Padre o madre<br>4 | Hermano o hermana<br>5 | Otro familiar<br>6 | Proveedor de cuidado infantil u otros cuidados en casa<br>7 | Otra<br><br>Field Annotation: @DEFAULT=" [hh9_relationship_s_q2]"
2070	[hh9_relationship2_s_q2]<br><br>Show the field ONLY if: [language_q2] = '2' and [hh9_relationship_s_q2] = '7'	Persona 9: Especifique su relación con esta persona.	text<br>Field Annotation: @DEFAULT=" [hh9_relationship2_s_q2]"
2071	[hh9_age_s_q2]<br><br>Show the field ONLY if: [language_q2] = '2' and [hhcount_s_q2] > 8 and [hhcount_s_q2] < 13	Persona 9: ¿Qué edad tiene esta persona?<br>*(Especifique la edad en años)*	text (number, Min: 0, Max: 110)<br>Field Annotation: @DEFAULT=" [hh9_age_s_q2]"
2072	[hh9_s_q2ex_s_q2]<br><br>Show the field ONLY if: [language_q2] = '2' and [hhcount_s_q2] > 8 and [hhcount_s_q2] < 13	Persona 9: ¿Cuál es el sexo de esta persona?	radio<br>1 | Femenino<br>2 | Masculino<br><br>Field Annotation: @DEFAULT=" [hh9_s_q2ex_s_q2]"
2073	[hh9_race_s_q2]<br><br>Show the field ONLY if: [language_q2] = '2' and [hhcount_s_q2] > 8 and [hhcount_s_q2] < 13	Persona 9: ¿Cuál es la raza de esta persona?<br>*(Seleccione todo lo que corresponda.)*	checkbox<br>1 | hh9_race_s_q2___1 | Indio americano o nativo de Alaska<br>2 | hh9_race_s_q2___2 | Asiático<br>3 | hh9_race_s_q2___3 | Negro o afroestadounidense<br>4 | hh9_race_s_q2___4 | Nativo de Hawái o de las islas del Pacífico<br>5 | hh9_race_s_q2___5 | Blanco<br>6 | hh9_race_s_q2___6 | Otra<br>7 | hh9_race_s_q2___7 | No sabe<br><br>Field Annotation: @DEFAULT="

			[hh9_race_s_q2]"
2074	[hh9_ethn_s_q2]<br><br>Show the field ONLY if:<br>[language_q2] = '2' and<br>[hhcount_s_q2] > 8 and<br>[hhcount_s_q2] < 13	Persona 9: ¿Cuál es la identidad étnica de esta persona?	radio<br>1 Hispano o latino<br>2 Ni hispano ni latino<br>3 Otra<br>4 No sabe<br><br>Field Annotation: @DEFAULT="[hh9_ethn_s_q2]"
2075	[hh9_edu_s_q2]<br><br>Show the field ONLY if:<br>[language_q2] = '2' and<br>[hhcount_s_q2] > 8 and<br>[hhcount_s_q2] < 13	Persona 9: ¿Cuál es el nivel de educación de esta persona?	radio<br>1 No tiene educación formal<br>2 Kínder a 8° grado<br>3 Estudios de educación secundaria<br>4 Equivalencia de educación secundaria (GED)<br>5 Diploma de educación secundaria<br>6 Estudios de educación superior<br>7 Título universitario<br>8 Estudios de posgrado o más<br>9 No sabe<br><br>Field Annotation: @DEFAULT="[hh9_edu_s_q2]"
2076	[hh9_work_s_q2]<br><br>Show the field ONLY if:<br>[language_q2] = '2' and<br>[hhcount_s_q2] > 8 and<br>[hhcount_s_q2] < 13	Persona 9: ¿Cuál de las siguientes es la mejor descripción de la situación laboral actual de esta persona?	radio<br>1 Empleo a tiempo completo<br>2 Empleo a tiempo parcial<br>3 En busca de empleo<br>4 Jubilado<br>5 Ama de casa<br>6 Estudiante<br>7 Permiso de maternidad o paternidad<br>8 Permiso por enfermedad<br>9 Desempleado por discapacidad<br>10 Otra<br>11 No sabe<br><br>Field Annotation: @DEFAULT="[hh9_work_s_q2]"
2077	[hh9_work2_s_q2]<br><br>Show the field ONLY if:<br>[language_q2] = '2' and<br>[hhcount_s_q2] > 8 and	Persona 9: ¿Se considera esta persona actualmente empleado por cuenta propia (contratista independiente, trabajador esporádico (gig), etc.)?	radio<br>1 Sí<br>0 No

	[hhcount_s_q2] < 13		2 No sabe<br><br>Field Annotation: @DEFAULT="[hh9_work2_s_q2]"
2078	[hh9_work3_s_q2]<br><br>Show the field ONLY if: [language_q2] = '2' and [hhcount_s_q2] > 8 and [hhcount_s_q2] < 13 and ([hh9_work_s_q2] = '1' or [hh9_work_s_q2] = '2' or [hh9_work2_s_q2] = '1')	Persona 9: ¿Trabaja esta persona actualmente en alguno de los siguientes ambientes de alto riesgo de contagio de COVID-19?	checkbox<br>1 hh9_work3_s_q2___1 Ambiente de asistencia médica (hospital, clínica, centro de urgencias, etc.)<br>2 hh9_work3_s_q2___2 Ambiente residencial denso (hogar de ancianos, otro centro de asistencia de larga duración)<br>3 hh9_work3_s_q2___3 Prisión o cárcel<br>4 hh9_work3_s_q2___4 Establecimiento de envasado de carne<br>5 hh9_work3_s_q2___5 Establecimiento de envío o distribución<br>6 hh9_work3_s_q2___6 Establecimiento minorista de alto volumen (tienda de provisiones, etc.)<br>7 hh9_work3_s_q2___7 No sabe<br><br>Field Annotation: @DEFAULT="[hh9_work3_s_q2]"
2079	[hh9_work4_s_q2]<br><br>Show the field ONLY if: [language_q2] = '2' and [hhcount_s_q2] > 8 and [hhcount_s_q2] < 13 and ([hh9_work_s_q2] = '1' or [hh9_work_s_q2] = '2' or [hh9_work2_s_q2] = '1')	Persona 9: ¿Le ofrece el empleador a esta persona alguno de los siguientes beneficios en su empleo principal actual?<br>*(Seleccione todo lo que corresponda.)*	checkbox<br>1 hh9_work4_s_q2___1 Permiso por enfermedad con goce de sueldo<br>2 hh9_work4_s_q2___2 Vacaciones o permiso personal con goce de sueldo<br>3 hh9_work4_s_q2___3 Seguro de salud<br>4 hh9_work4_s_q2___4 Seguro de discapacidad<br>5 hh9_work4_s_q2___5 Plan de jubilación

			6 hh9_work4_s_q2___6 Otro<br>7 hh9_work4_s_q2___7 No sabe<br><br>Field Annotation: @DEFAULT="[hh9_work4_s_q2]"
2080	[hh9_work5_s_q2]<br><br>Show the field ONLY if: [language_q2] = '2' and [hhcount_s_q2] > 8 and [hhcount_s_q2] < 13 and ([hh9_work_s_q2] = '1' or [hh9_work_s_q2] = '2' or [hh9_work2_s_q2] = '1')	Persona 9: En una escala de 0 (definitivamente no va a suceder) a 10 (definitivamente va a suceder), ¿qué tan probable es que esta persona pierda su empleo debido a la pandemia de COVID-19?	text (number, Min: 0, Max: 10)<br>Field Annotation: @DEFAULT="[hh9_work5_s_q2]"
2081	[hh9_work6_s_q2]<br><br>Show the field ONLY if: [language_q2] = '2' and [hhcount_s_q2] > 8 and [hhcount_s_q2] < 13 and ([hh9_work_s_q2] = '1' or [hh9_work_s_q2] = '2' or [hh9_work2_s_q2] = '1')	Persona 9: En una escala de 0 (definitivamente no va a suceder) a 10 (definitivamente va a suceder), ¿qué tan probable es que a esta persona se le asignen menos horas de trabajo debido a la pandemia de COVID-19?	text (number, Min: 0, Max: 10)<br>Field Annotation: @DEFAULT="[hh9_work6_s_q2]"
2082	[hh9_work7_s_q2]<br><br>Show the field ONLY if: [language_q2] = '2' and [hhcount_s_q2] > 8 and [hhcount_s_q2] < 13 and ([hh9_work_s_q2] = '1' or [hh9_work_s_q2] = '2' or [hh9_work2_s_q2] = '1')	Persona 9: Actualmente ¿con qué frecuencia se le exige a esta persona que trabaje fuera de su residencia?	radio (Matrix)<br>1 Todo el tiempo (100%)<br>2 La mayor parte del tiempo (75%)<br>3 La mitad del tiempo (50%)<br>4 Menos de la mitad del tiempo (25%)<br>5 nunca (0%)<br>6 Nunca
2083	[hh9_work8_s_q2]<br><br>Show the field ONLY if: [language_q2] = '2' and ([hh9_work7_s_q2] = '1' or [hh9_work7_s_q2] = '2' or [hh9_work7_s_q2] = '3' or [hh9_work7_s_q2] = '4')	Persona 9: Actualmente, ¿con qué frecuencia se encuentra esta persona físicamente cerca de sus compañeros de trabajo mientras trabaja fuera de su residencia?	radio (Matrix)<br>1 Todo el tiempo (100%)<br>2 La mayor parte del tiempo (75%)<br>3 La mitad del tiempo (50%)<br>4 Menos de la mitad del tiempo (25%)<br>5 nunca (0%)<br>6 Nunca
2084	[hh9_work9_s_q2]<br><br>Show the field ONLY if: [language_q2] = '2' and ([hh9_work7_s_q2] = '1' or [hh9_work7_s_q2] = '2' or [hh9_work7_s_q2] = '3' or [hh9_work7_s_q2] = '4')	Persona 9: Actualmente, ¿con qué frecuencia se encuentra esta persona físicamente cerca de sus clientes mientras trabaja fuera de su residencia	radio (Matrix)<br>1 Todo el tiempo (100%)<br>2 La mayor parte del tiempo (75%)<br>3 La mitad del tiempo (50%)<br>4 Menos de la mitad del tiempo (25%)<br>5 nunca (0%)<br>6 Nunca

2085	[hh9_covidvaccine_s_q2]<br><br>Show the field ONLY if: [language_q2] = '2' and [hhcount_s_q2] > 8 and [hhcount_s_q2] < 13	Persona 9: ¿Piensa esta persona vacunarse contra la COVID-19?	radio<br>1 Sí<br>0 No<br>2 No sabe<br>3 Este individuo ya ha sido vacunado<br><br>Field Annotation: @DEFAULT="[hh9_covidvaccine_s_q2]"
2086	[hh9_covidsymp_s_q2]<br><br>Show the field ONLY if: [language_q2] = '2' and [hhcount_s_q2] > 8 and [hhcount_s_q2] < 13	Persona 9: En las últimas dos semanas, ¿ha tenido esta persona algún síntoma de COVID-19 (tos, fiebre, dificultad para respirar, fatiga, dolores de cuerpo, diarrea, goteo nasal o pérdida del sentido del olfato o del gusto)?	radio<br>1 Sí<br>0 No<br>2 No sabe
2087	[hh9_covidsymp2_s_q2]<br><br>Show the field ONLY if: [language_q2] = '2' and [hh9_covidsymp_s_q2] = '1'	Persona 9: ¿Cuándo le comenzaron los síntomas de COVID-19 a esta persona?	text (date_mdy)
2088	[hh9_covidsymp3_s_q2]<br><br>Show the field ONLY if: [language_q2] = '2' and [hh9_covidsymp_s_q2] = '1'	Persona 9: En vista de sus síntomas, ¿le preocupa a esta persona la posibilidad de tener COVID-19?	radio<br>1 Sí<br>0 No<br>2 No sabe
2089	[hh9_covidsymp4_s_q2]<br><br>Show the field ONLY if: [language_q2] = '2' and [hh9_covidsymp_s_q2] = '1'	Persona 9: ¿Fue esta persona objeto de prejuicio o discriminación debido a sus síntomas?	radio<br>1 Sí<br>0 No<br>2 No sabe
2090	[hh9_covidsymp5_s_q2]<br><br>Show the field ONLY if: [language_q2] = '2' and [hh9_covidsymp_s_q2] = '1'	Persona 9: ¿Qué hizo esta persona en vista de sus síntomas?<br>*(Seleccione todo lo que corresponda.)*	checkbox<br>0 hh9_covidsymp5_s_q2___0 Nada<br>1 hh9_covidsymp5_s_q2___1 Tomó medicament sin receta (ibuprofeno, acetaminofé etc.)<br>2 hh9_covidsymp5_s_q2___2 Consultó por teléfono a un proveedor de asistencia médica<br>3 hh9_covidsymp5_s_q2___3 Fue al consultorio d un proveedo

			de asistencia médica<br>4 | hh9_covidsymp5_s_q2___4 | Fue a una clínica o una farmacia minorista<br>5 | hh9_covidsymp5_s_q2___5 | Fue a un centro de urgencias (FASTMed, etc.)<br>6 | hh9_covidsymp5_s_q2___6 | Fue a la sala de emergencias<br>7 | hh9_covidsymp5_s_q2___7 | Fue ingresad al hospital<br>8 | hh9_covidsymp5_s_q2___8 | Otra cosa<br>9 | hh9_covidsymp5_s_q2___9 | No sabe
2091	[hh9_covidsymp6_s_q2]<br>Show the field ONLY if: [language_q2] = '2' and [hh9_covidsymp5_s_q2(8)] = '1'	Persona 9: ¿Qué otra cosa hizo esta persona en vista de sus síntomas?	text
2092	[hh9_covidsymp7_s_q2]<br>Show the field ONLY if: [language_q2] = '2' and ([hh9_covidsymp5_s_q2(2)] = '1' or [hh9_covidsymp5_s_q2(3)] = '1' or [hh9_covidsymp5_s_q2(4)] = '1' or [hh9_covidsymp5_s_q2(5)] = '1' or [hh9_covidsymp5_s_q2(6)] = '1' or [hh9_covidsymp5_s_q2(7)] = '1' or [hh9_covidsymp5_s_q2(8)] = '1')	Persona 9: ¿Le dijo un proveedor de asistencia médica a esta persona que era posible que tuviera COVID-19?	radio<br>1 | Sí<br>0 | No<br>2 | No sabe
2093	[hh9_covid_test_s_q2]<br>Show the field ONLY if: [language_q2] = '2' and [hh9_covidsymp_s_q2] = '1'	Persona 9: Si a esta persona le hicieron una prueba de COVID-19 en vista de sus síntomas, ¿cuál fue el resultado?	radio<br>1 | Pendiente<br>2 | Positivo<br>3 | Negativo<br>4 | No concluyente<br>5 | No se hizo la prueba<br>6 | No sabe
2094	[hh9_covid_admit_s_q2]	Persona 9: ¿Cuántos días estuvo hospitalizada esta persona?	text (number, Min: 0)

	Show the field ONLY if: [language_q2] = '2' and [hh9_covidsymp5_s_q2(7)] = '1'		
2095	[hh9_covid_admit2_s_q2] Show the field ONLY if: [language_q2] = '2' and [hh9_covidsymp5_s_q2(7)] = '1'	Persona 9: ¿Se le hicieron a esta persona las siguientes intervenciones durante su hospitalización?	checkbox<br>1 hh9_covid_admit2_s_q2___1 Oxígeno adicional por la nariz<br>2 hh9_covid_admit2_s_q2___2 Tratamiento en la Unidad de Cuidados Intensivos (Intensive Care Unit, ICU)<br>3 hh9_covid_admit2_s_q2___3 Ventilación mecánica (intubación o tubo de respiración)<br>4 hh9_covid_admit2_s_q2___4 No sabe
2096	[hh9_covidsymp8_s_q2] Show the field ONLY if: [language_q2] = '2' and [hh9_covidsymp_s_q2] = '1'	Persona 9: ¿Ha vuelto esta persona a su salud normal?	radio<br>1 Sí<br>0 No<br>2 No sabe
2097	[hh9_prevent_s_q2] Show the field ONLY if: [language_q2] = '2' and [hh9_covidsymp_s_q2] = '1'	Persona 9: ¿Cuáles de las siguientes medidas tomó esta persona para proteger a sus amigos y familiares después de que comenzaron sus síntomas?	checkbox<br>1 hh9_prevent_s_q2___1 Ponerse mascarilla con mayor frecuencia<br>2 hh9_prevent_s_q2___2 Lavarse las manos con agua y jabón con mayor frecuencia<br>3 hh9_prevent_s_q2___3 Limpiarse las manos con desinfectante con mayor frecuencia<br>4 hh9_prevent_s_q2___4 Aislarse en su casa con mayor frecuencia<br>5 hh9_prevent_s_q2___5 Quedarse en casa con mayor frecuencia

			6 | hh9_prevent_s_q2___6 | Ponerse guantes desechables con mayor frecuencia<br>7 | hh9_prevent_s_q2___7 | No sabe
2098	[hh10_relationship_s_q2]<br><br>Show the field ONLY if: [language_q2] = '2' and [hhcount_s_q2] > 9 and [hhcount_s_q2] < 13	Section Header: *Por cada persona adicional en su hogar, proporcione la siguiente informacion.*<br><br>Persona 10: ¿Cuál es su relación con esta persona?	radio<br>1 | Pareja o cónyuge<br>2 | Hijo o hija<br>3 | Padre o madre<br>4 | Hermano o hermana<br>5 | Otro familiar<br>6 | Proveedor de cuidado infantil u otros cuidados en casa<br>7 | Otra<br><br>Field Annotation: @DEFAULT="[hh10_relationship_s_q2]"
2099	[hh10_relationship2_s_q2]<br><br>Show the field ONLY if: [language_q2] = '2' and [hh10_relationship_s_q2] = '7'	Persona 10: Especifique su relación con esta persona.	text<br>Field Annotation: @DEFAULT="[hh10_relationship2_s_q2]"
2100	[hh10_age_s_q2]<br><br>Show the field ONLY if: [language_q2] = '2' and [hhcount_s_q2] > 9 and [hhcount_s_q2] < 13	Persona 10: ¿Qué edad tiene esta persona?<br>*(Especifique la edad en años)*	text (number, Min: 0, Max: 110)<br>Field Annotation: @DEFAULT="[hh10_age_s_q2]"
2101	[hh10_s_q2ex_s_q2]<br><br>Show the field ONLY if: [language_q2] = '2' and [hhcount_s_q2] > 9 and [hhcount_s_q2] < 13	Persona 10: ¿Cuál es el sexo de esta persona?	radio<br>1 | Femenino<br>2 | Masculino<br><br>Field Annotation: @DEFAULT="[hh10_s_q2ex_s_q2]"
2102	[hh10_race_s_q2]<br><br>Show the field ONLY if: [language_q2] = '2' and [hhcount_s_q2] > 9 and [hhcount_s_q2] < 13	Persona 10: ¿Cuál es la raza de esta persona?<br>*(Seleccione todo lo que corresponda.)*	checkbox<br>1 | hh10_race_s_q2___1 | Indio americano o nativo de Alaska<br>2 | hh10_race_s_q2___2 | Asiático<br>3 | hh10_race_s_q2___3 | Negro o afroestadounidens<br>4 | hh10_race_s_q2___4 | Nativo de Hawái o de las islas del Pacífico<br>5 | hh10_race_s_q2___5 | Blanco<br>6 | hh10_race_s_q2___6 | Otra

			7 hh10_race_s_q2___7 No sabe<br><br>Field Annotation: @DEFAULT="[hh10_race_s_q2]"
2103	[hh10_ethn_s_q2]<br><br>Show the field ONLY if:<br>[language_q2] = '2' and<br>[hhcount_s_q2] > 9 and<br>[hhcount_s_q2] < 13	Persona 10: ¿Cuál es la identidad étnica de esta persona?	radio<br>1 Hispano o latino<br>2 Ni hispano ni latino<br>3 Otra<br>4 No sabe<br><br>Field Annotation: @DEFAULT="[hh10_ethn_s_q2]"
2104	[hh10_edu_s_q2]<br><br>Show the field ONLY if:<br>[language_q2] = '2' and<br>[hhcount_s_q2] > 9 and<br>[hhcount_s_q2] < 13	Persona 10: ¿Cuál es el nivel de educación de esta persona?	radio<br>1 No tiene educación formal<br>2 Kínder a 8° grado<br>3 Estudios de educación secundaria<br>4 Equivalencia de educación secundaria (GED)<br>5 Diploma de educación secundaria<br>6 Estudios de educación superior<br>7 Título universitario<br>8 Estudios de posgrado o más<br>9 No sabe<br><br>Field Annotation: @DEFAULT="[hh10_edu_s_q2]"
2105	[hh10_work_s_q2]<br><br>Show the field ONLY if:<br>[language_q2] = '2' and<br>[hhcount_s_q2] > 9 and<br>[hhcount_s_q2] < 13	Persona 10: ¿Cuál de las siguientes es la mejor descripción de la situación laboral actual de esta persona?	radio<br>1 Empleo a tiempo completo<br>2 Empleo a tiempo parcial<br>3 En busca de empleo<br>4 Jubilado<br>5 Ama de casa<br>6 Estudiante<br>7 Permiso de maternidad o paternidad<br>8 Permiso por enfermedad<br>9 Desempleado por discapacidad<br>10 Otra<br>11 No sabe<br><br>Field Annotation: @DEFAULT="[hh10_work_s_q2]"

2106	[hh10_work2_s_q2]<br><br>Show the field ONLY if:<br>[language_q2] = '2' and [hhcount_s_q2] > 9 and [hhcount_s_q2] < 13	Persona 10: ¿Se considera esta persona actualmente empleado por cuenta propia (contratista independiente, trabajador esporádico (gig), etc.)?	radio<br>1 Sí<br>0 No<br>2 No sabe<br><br>Field Annotation: @DEFAULT="[hh10_work2_s_q2]"
2107	[hh10_work3_s_q2]<br><br>Show the field ONLY if:<br>[language_q2] = '2' and [hhcount_s_q2] > 9 and [hhcount_s_q2] < 13 and ([hh10_work_s_q2] = '1' or [hh10_work_s_q2] = '2' or [hh10_work2_s_q2] = '1')	Persona 10: ¿Trabaja esta persona actualmente en alguno de los siguientes ambientes de alto riesgo de contagio de COVID-19?	checkbox<br>1 hh10_work3_s_q2___1 Ambiente de asistencia médica (hospital, clínica, centro de urgencias, etc.)<br>2 hh10_work3_s_q2___2 Ambiente residencial denso (hogar de ancianos, otro centro de asistencia de larga duración)<br>3 hh10_work3_s_q2___3 Prisión o cárcel<br>4 hh10_work3_s_q2___4 Establecimiento de envasado de carne<br>5 hh10_work3_s_q2___5 Establecimiento de envío o distribución<br>6 hh10_work3_s_q2___6 Establecimiento minorista de alto volumen (tienda de provisiones, etc.)<br>7 hh10_work3_s_q2___7 No sabe<br><br>Field Annotation: @DEFAULT=[q2_2_arm_1][hh10_work3_s_q2:value] @DEFAULT="[hh10_work3_s_q2]"
2108	[hh10_work4_s_q2]<br><br>Show the field ONLY if:<br>[language_q2] = '2' and [hhcount_s_q2] > 9 and [hhcount_s_q2] < 13 and ([hh10_work_s_q2] = '1' or [hh10_work_s_q2] = '2' or [hh10_work2_s_q2] = '1')	Persona 10: ¿Le ofrece el empleador a esta persona alguno de los siguientes beneficios en su empleo principal actual?<br>*(Seleccione todo lo que corresponda.)*	checkbox<br>1 hh10_work4_s_q2___1 Permiso por enfermedad con goce de sueldo<br>2 hh10_work4_s_q2___2 Vacaciones o permiso personal con goce de sueldo

			3 hh10_work4_s_q2___3 Seguro de salud<br>4 hh10_work4_s_q2___4 Seguro de discapacidad<br>5 hh10_work4_s_q2___5 Plan de jubilación<br>6 hh10_work4_s_q2___6 Otro<br>7 hh10_work4_s_q2___7 No sabe<br><br>Field Annotation: @DEFAULT="[hh10_work4_s_q2]"
2109	[hh10_work5_s_q2]<br><br>Show the field ONLY if: [language_q2] = '2' and [hhcount_s_q2] > 9 and [hhcount_s_q2] < 13 and ([hh10_work_s_q2] = '1' or [hh10_work_s_q2] = '2' or [hh10_work2_s_q2] = '1')	Persona 10: En una escala de 0 (definitivamente no va a suceder) a 10 (definitivamente va a suceder), ¿qué tan probable es que esta persona pierda su empleo debido a la pandemia de COVID-19?	text (number, Min: 0, Max: 10)<br>Field Annotation: @DEFAULT="[hh10_work5_s_q2]"
2110	[hh10_work6_s_q2]<br><br>Show the field ONLY if: [language_q2] = '2' and [hhcount_s_q2] > 9 and [hhcount_s_q2] < 13 and ([hh10_work_s_q2] = '1' or [hh10_work_s_q2] = '2' or [hh10_work2_s_q2] = '1')	Persona 10: En una escala de 0 (definitivamente no va a suceder) a 10 (definitivamente va a suceder), ¿qué tan probable es que a esta persona se le asignen menos horas de trabajo debido a la pandemia de COVID-19?	text (number, Min: 0, Max: 10)<br>Field Annotation: @DEFAULT="[hh10_work6_s_q2]"
2111	[hh10_work7_s_q2]<br><br>Show the field ONLY if: [language_q2] = '2' and [hhcount_s_q2] > 9 and [hhcount_s_q2] < 13 and ([hh10_work_s_q2] = '1' or [hh10_work_s_q2] = '2' or [hh10_work2_s_q2] = '1')	Persona 10: Actualmente ¿con qué frecuencia se le exige a esta persona que trabaje fuera de su residencia?	radio (Matrix)<br>1 Todo el tiempo (100%)<br>2 La mayor parte del tiempo (75%)<br>3 La mitad del tiempo (50%)<br>4 Menos de la mitad del tiempo (25%)<br>5 nunca (0%)<br>6 Nunca
2112	[hh10_work8_s_q2]<br><br>Show the field ONLY if: [language_q2] = '2' and ([hh10_work7_s_q2] = '1' or [hh10_work7_s_q2] = '2' or [hh10_work7_s_q2] = '3' or [hh10_work7_s_q2] = '4')	Persona 10: Actualmente, ¿con qué frecuencia se encuentra esta persona físicamente cerca de sus compañeros de trabajo mientras trabaja fuera de su residencia?	radio (Matrix)<br>1 Todo el tiempo (100%)<br>2 La mayor parte del tiempo (75%)<br>3 La mitad del tiempo (50%)<br>4 Menos de la mitad del tiempo (25%)<br>5 nunca (0%)<br>6 Nunca
2113	[hh10_work9_s_q2]<br><br>Show the field ONLY if:	Persona 10: Actualmente, ¿con qué frecuencia se encuentra esta persona físicamente cerca de sus clientes mientras trabaja fuera de su residencia	radio (Matrix)<br>1 Todo el tiempo (100%)

	[language_q2] = '2' and ([hh10_work7_s_q2] = '1' or [hh10_work7_s_q2] = '2' or [hh10_work7_s_q2] = '3' or [hh10_work7_s_q2] = '4')		2 La mayor parte del tiempo (75%)<br>3 La mitad del tiempo (50%)<br>4 Menos de la mitad del tiempo (25%)<br>5 nunca (0%)<br>6 Nunca
2114	[hh10_covidvaccine_s_q2]<br><br>Show the field ONLY if: [language_q2] = '2' and [hhcount_s_q2] > 9 and [hhcount_s_q2] < 13	Persona 10: ¿Piensa esta persona vacunarse contra la COVID-19?	radio<br>1 Sí<br>0 No<br>2 No sabe<br>3 Este individuo ya ha sido vacunado<br><br>Field Annotation: @DEFAULT="[hh10_covidvaccine_s_q2]"
2115	[hh10_covidsymp_s_q2]<br><br>Show the field ONLY if: [language_q2] = '2' and [hhcount_s_q2] > 9 and [hhcount_s_q2] < 13	Persona 10: En las últimas dos semanas, ¿ha tenido esta persona algún síntoma de COVID-19 (tos, fiebre, dificultad para respirar, fatiga, dolores de cuerpo, diarrea, goteo nasal o pérdida del sentido del olfato o del gusto)?	radio<br>1 Sí<br>0 No<br>2 No sabe
2116	[hh10_covidsymp2_s_q2]<br><br>Show the field ONLY if: [language_q2] = '2' and [hh10_covidsymp_s_q2] = '1'	Persona 10: ¿Cuándo le comenzaron los síntomas de COVID-19 a esta persona?	text (date_mdy)
2117	[hh10_covidsymp3_s_q2]<br><br>Show the field ONLY if: [language_q2] = '2' and [hh10_covidsymp_s_q2] = '1'	Persona 10: En vista de sus síntomas, ¿le preocupa a esta persona la posibilidad de tener COVID-19?	radio<br>1 Sí<br>0 No<br>2 No sabe
2118	[hh10_covidsymp4_s_q2]<br><br>Show the field ONLY if: [language_q2] = '2' and [hh10_covidsymp_s_q2] = '1'	Persona 10: ¿Fue esta persona objeto de prejuicio o discriminación debido a sus síntomas?	radio<br>1 Sí<br>0 No<br>2 No sabe
2119	[hh10_covidsymp5_s_q2]<br><br>Show the field ONLY if: [language_q2] = '2' and [hh10_covidsymp_s_q2] = '1'	Persona 10: ¿Qué hizo esta persona en vista de sus síntomas?<br>*(Seleccione todo lo que corresponda.)*	checkbox<br>0 hh10_covidsymp5_s_q2___0 Nada<br>1 hh10_covidsymp5_s_q2___1 Tomó medicamen sin receta (ibuprofenc acetaminofe etc.)<br>2 hh10_covidsymp5_s_q2___2 Consultó pc teléfono a u

			<table><tr><td></td><td></td><td>proveedor [illegible] asistencia médica</td></tr><tr><td>3</td><td>hh10_covidsymp5_s_q2___3</td><td>Fue al consultorio [illegible] un proveed[illegible] de asistenc[illegible] médica</td></tr><tr><td>4</td><td>hh10_covidsymp5_s_q2___4</td><td>Fue a una clínica o un[illegible] farmacia minorista</td></tr><tr><td>5</td><td>hh10_covidsymp5_s_q2___5</td><td>Fue a un centro de urgencias (FASTMed, etc.)</td></tr><tr><td>6</td><td>hh10_covidsymp5_s_q2___6</td><td>Fue a la sal[illegible] de emergencia[illegible]</td></tr><tr><td>7</td><td>hh10_covidsymp5_s_q2___7</td><td>Fue ingresa[illegible] al hospital</td></tr><tr><td>8</td><td>hh10_covidsymp5_s_q2___8</td><td>Otra cosa</td></tr><tr><td>9</td><td>hh10_covidsymp5_s_q2___9</td><td>No sabe</td></tr></table>
2120	[hh10_covidsymp6_s_q2]<br><br>Show the field ONLY if: [language_q2] = '2' and [hh10_covidsymp5_s_q2(8)] = '1'	Persona 10: ¿Qué otra cosa hizo esta persona en vista de sus síntomas?	text
2121	[hh10_covidsymp7_s_q2]<br><br>Show the field ONLY if: [language_q2] = '2' and ([hh10_covidsymp5_s_q2(2)] = '1' or [hh10_covidsymp5_s_q2(3)] = '1' or [hh10_covidsymp5_s_q2(4)] = '1' or [hh10_covidsymp5_s_q2(5)] = '1' or [hh10_covidsymp5_s_q2(6)] = '1' or [hh10_covidsymp5_s_q2(7)] = '1' or [hh10_covidsymp5_s_q2(8)] = '1')	Persona 10: ¿Le dijo un proveedor de asistencia médica a esta persona que era posible que tuviera COVID-19?	radio<br><table><tr><td>1</td><td>Sí</td></tr><tr><td>0</td><td>No</td></tr><tr><td>2</td><td>No sabe</td></tr></table>
2122	[hh10_covid_test_s_q2]<br><br>Show the field ONLY if: [language_q2] = '2' and [hh10_covidsymp_s_q2] = '1'	Persona 10: Si a esta persona le hicieron una prueba de COVID-19 en vista de sus síntomas, ¿cuál fue el resultado?	radio<br><table><tr><td>1</td><td>Pendiente</td></tr><tr><td>2</td><td>Positivo</td></tr><tr><td>3</td><td>Negativo</td></tr><tr><td>4</td><td>No concluyente</td></tr></table>

			5 No se hizo la prueba<br>6 No sabe
2123	[hh10_covid_admit_s_q2]<br><br>Show the field ONLY if: [language_q2] = '2' and [hh10_covidsymp5_s_q2(7)] = '1'	Persona 10: ¿Cuántos días estuvo hospitalizada esta persona?	text (number, Min: 0)
2124	[hh10_covid_admit2_s_q2]<br><br>Show the field ONLY if: [language_q2] = '2' and [hh10_covidsymp5_s_q2(7)] = '1'	Persona 10: ¿Se le hicieron a esta persona las siguientes intervenciones durante su hospitalización?	checkbox<br>1 hh10_covid_admit2_s_q2___1 Oxígeno adiciona[illegible] por la nari[illegible]<br>2 hh10_covid_admit2_s_q2___2 Tratamien[illegible] en la Unidad de Cuidados Intensivos (Intensive Care Unit, ICU)<br>3 hh10_covid_admit2_s_q2___3 Ventilació[illegible] mecánica (intubació[illegible] o tubo de respiració[illegible]<br>4 hh10_covid_admit2_s_q2___4 No sabe
2125	[hh10_covidsymp8_s_q2]<br><br>Show the field ONLY if: [language_q2] = '2' and [hh10_covidsymp_s_q2] = '1'	Persona 10: ¿Ha vuelto esta persona a su salud normal?	radio<br>1 Sí<br>0 No<br>2 No sabe
2126	[hh10_prevent_s_q2]<br><br>Show the field ONLY if: [language_q2] = '2' and [hh10_covidsymp_s_q2] = '1'	Persona 10: ¿Cuáles de las siguientes medidas tomó esta persona para proteger a sus amigos y familiares después de que comenzaron sus síntomas?	checkbox<br>1 hh10_prevent_s_q2___1 Ponerse mascarilla con mayor frecuencia<br>2 hh10_prevent_s_q2___2 Lavarse las manos con agua y jabón con mayor frecuencia<br>3 hh10_prevent_s_q2___3 Limpiarse las manos con desinfectante con mayor frecuencia<br>4 hh10_prevent_s_q2___4 Aislarse en su casa con

			(continued) | | | mayor frecuencia | | 5 | hh10_prevent_s_q2___5 | Quedarse en casa con mayor frecuencia | | 6 | hh10_prevent_s_q2___6 | Ponerse guantes desechables con mayor frecuencia | | 7 | hh10_prevent_s_q2___7 | No sabe |
2127	[hh11_relationship_s_q2]<br><br>Show the field ONLY if: [language_q2] = '2' and [hhcount_s_q2] > 10 and [hhcount_s_q2] < 13	Section Header: *Por cada persona adicional en su hogar, proporcione la siguiente informacion.*<br><br>Persona 11: ¿Cuál es su relación con esta persona?	radio<br>1 Pareja o cónyuge<br>2 Hijo o hija<br>3 Padre o madre<br>4 Hermano o hermana<br>5 Otro familiar<br>6 Proveedor de cuidado infantil u otros cuidados en casa<br>7 Otra<br><br>Field Annotation: @DEFAULT="[hh11_relationship_s_q2]"
2128	[hh11_relationship2_s_q2]<br><br>Show the field ONLY if: [language_q2] = '2' and [hh11_relationship_s_q2] = '7'	Persona 11: Especifique su relación con esta persona.	text<br>Field Annotation: @DEFAULT="[hh11_relationship2_s_q2]"
2129	[hh11_age_s_q2]<br><br>Show the field ONLY if: [language_q2] = '2' and [hhcount_s_q2] > 10 and [hhcount_s_q2] < 13	Persona 11: ¿Qué edad tiene esta persona? *(Especifique la edad en años)*	text (number, Min: 0, Max: 110)<br>Field Annotation: @DEFAULT="[hh11_age_s_q2]"
2130	[hh11_s_q2ex_s_q2]<br><br>Show the field ONLY if: [language_q2] = '2' and [hhcount_s_q2] > 10 and [hhcount_s_q2] < 13	Persona 11: ¿Cuál es el sexo de esta persona?	radio<br>1 Femenino<br>2 Masculino<br><br>Field Annotation: @DEFAULT="[hh11_s_q2ex_s_q2]"
2131	[hh11_race_s_q2]<br><br>Show the field ONLY if: [language_q2] = '2' and [hhcount_s_q2] > 10 and [hhcount_s_q2] < 13	Persona 11: ¿Cuál es la raza de esta persona? *(Seleccione todo lo que corresponda.)*	checkbox<br>1 hh11_race_s_q2___1 Indio americano o nativo de Alaska<br>2 hh11_race_s_q2___2 Asiático<br>3 hh11_race_s_q2___3 Negro o afroestadounidens

			4 | hh11_race_s_q2___4 | Nativo de Hawái o de las islas del Pacífico<br>5 | hh11_race_s_q2___5 | Blanco<br>6 | hh11_race_s_q2___6 | Otra<br>7 | hh11_race_s_q2___7 | No sabe<br><br>Field Annotation: @DEFAULT="[hh11_race_s_q2]"
2132	[hh11_ethn_s_q2]<br><br>Show the field ONLY if: [language_q2] = '2' and [hhcount_s_q2] > 10 and [hhcount_s_q2] < 13	Persona 11: ¿Cuál es la identidad étnica de esta persona?	radio<br>1 | Hispano o latino<br>2 | Ni hispano ni latino<br>3 | Otra<br>4 | No sabe<br><br>Field Annotation: @DEFAULT="[hh11_ethn_s_q2]"
2133	[hh11_edu_s_q2]<br><br>Show the field ONLY if: [language_q2] = '2' and [hhcount_s_q2] > 10 and [hhcount_s_q2] < 13	Persona 11: ¿Cuál es el nivel de educación de esta persona?	radio<br>1 | No tiene educación formal<br>2 | Kínder a 8° grado<br>3 | Estudios de educación secundaria<br>4 | Equivalencia de educación secundaria (GED)<br>5 | Diploma de educación secundaria<br>6 | Estudios de educación superior<br>7 | Título universitario<br>8 | Estudios de posgrado o más<br>9 | No sabe<br><br>Field Annotation: @DEFAULT="[hh11_edu_s_q2]"
2134	[hh11_work_s_q2]<br><br>Show the field ONLY if: [language_q2] = '2' and [hhcount_s_q2] > 10 and [hhcount_s_q2] < 13	Persona 11: ¿Cuál de las siguientes es la mejor descripción de la situación laboral actual de esta persona?	radio<br>1 | Empleo a tiempo completo<br>2 | Empleo a tiempo parcial<br>3 | En busca de empleo<br>4 | Jubilado<br>5 | Ama de casa<br>6 | Estudiante<br>7 | Permiso de maternidad o paternidad<br>8 | Permiso por enfermedad<br>9 | Desempleado por discapacidad<br>10 | Otra

			11 No sabe<br><br>Field Annotation: @DEFAULT="[hh11_work_s_q2]"
2135	[hh11_work2_s_q2]<br><br>Show the field ONLY if: [language_q2] = '2' and [hhcount_s_q2] > 10 and [hhcount_s_q2] < 13	Persona 11: ¿Se considera esta persona actualmente empleado por cuenta propia (contratista independiente, trabajador esporádico (gig), etc.)?	radio<br>1 Sí<br>0 No<br>2 No sabe<br><br>Field Annotation: @DEFAULT="[hh11_work2_s_q2]"
2136	[hh11_work3_s_q2]<br><br>Show the field ONLY if: [language_q2] = '2' and [hhcount_s_q2] > 10 and [hhcount_s_q2] < 13 and ([hh11_work_s_q2] = '1' or [hh11_work_s_q2] = '2' or [hh11_work2_s_q2] = '1')	Persona 11: ¿Trabaja esta persona actualmente en alguno de los siguientes ambientes de alto riesgo de contagio de COVID-19?	checkbox<br>1 hh11_work3_s_q2___1 Ambiente de asistencia médica (hospital, clínica, centro de urgencias, etc.)<br>2 hh11_work3_s_q2___2 Ambiente residencial denso (hogar de ancianos, otro centro de asistencia de larga duración)<br>3 hh11_work3_s_q2___3 Prisión o cárcel<br>4 hh11_work3_s_q2___4 Establecimiento de envasado de carne<br>5 hh11_work3_s_q2___5 Establecimiento de envío o distribución<br>6 hh11_work3_s_q2___6 Establecimiento minorista de alto volumen (tienda de provisiones, etc.)<br>7 hh11_work3_s_q2___7 No sabe<br><br>Field Annotation: @DEFAULT="[hh11_work3_s_q2]"
2137	[hh11_work4_s_q2]<br><br>Show the field ONLY if: [language_q2] = '2' and [hhcount_s_q2] > 10 and [hhcount_s_q2] < 13 and ([hh11_work_s_q2] = '1' or [hh11_work_s_q	Persona 11: ¿Le ofrece el empleador a esta persona alguno de los siguientes beneficios en su empleo principal actual?<br>*(Seleccione todo lo que corresponda.)*	checkbox<br>1 hh11_work4_s_q2___1 Permiso por enfermedad con goce de sueldo<br>2 hh11_work4_s_q2___2 Vacaciones o permiso

	2] = '2' or [hh11_work2_s_q2] = '1')		personal con goce de sueldo<br>3 hh11_work4_s_q2___3 Seguro de salud<br>4 hh11_work4_s_q2___4 Seguro de discapacidad<br>5 hh11_work4_s_q2___5 Plan de jubilación<br>6 hh11_work4_s_q2___6 Otro<br>7 hh11_work4_s_q2___7 No sabe<br><br>Field Annotation: @DEFAULT="[hh11_work4_s_q2]"
2138	[hh11_work5_s_q2]<br><br>Show the field ONLY if: [language_q2] = '2' and [hhcount_s_q2] > 10 and [hhcount_s_q2] < 13 and ([hh11_work_s_q2] = '1' or [hh11_work_s_q2] = '2' or [hh11_work2_s_q2] = '1')	Persona 11: En una escala de 0 (definitivamente no va a suceder) a 10 (definitivamente va a suceder), ¿qué tan probable es que esta persona pierda su empleo debido a la pandemia de COVID-19?	text (number, Min: 0, Max: 10)<br>Field Annotation: @DEFAULT="[hh11_work5_s_q2]"
2139	[hh11_work6_s_q2]<br><br>Show the field ONLY if: [language_q2] = '2' and [hhcount_s_q2] > 10 and [hhcount_s_q2] < 13 and ([hh11_work_s_q2] = '1' or [hh11_work_s_q2] = '2' or [hh11_work2_s_q2] = '1')	Persona 11: En una escala de 0 (definitivamente no va a suceder) a 10 (definitivamente va a suceder), ¿qué tan probable es que a esta persona se le asignen menos horas de trabajo debido a la pandemia de COVID-19?	text (number, Min: 0, Max: 10)<br>Field Annotation: @DEFAULT="[hh11_work6_s_q2]"
2140	[hh11_work7_s_q2]<br><br>Show the field ONLY if: [language_q2] = '2' and [hhcount_s_q2] > 10 and [hhcount_s_q2] < 13 and ([hh11_work_s_q2] = '1' or [hh11_work_s_q2] = '2' or [hh11_work2_s_q2] = '1')	Persona 11: Actualmente ¿con qué frecuencia se le exige a esta persona que trabaje fuera de su residencia?	radio (Matrix)<br>1 Todo el tiempo (100%)<br>2 La mayor parte del tiempo (75%)<br>3 La mitad del tiempo (50%)<br>4 Menos de la mitad del tiempo (25%)<br>5 nunca (0%)<br>6 Nunca
2141	[hh11_work8_s_q2]<br><br>Show the field ONLY if: [language_q2] = '2' and ([hh11_work7_s_q2] = '1' or [hh11_work7_s_q2] = '2' or [hh11_work7_s_q2] = '3' or [hh11_work7_s_q2] = '4')	Persona 11: Actualmente, ¿con qué frecuencia se encuentra esta persona físicamente cerca de sus compañeros de trabajo mientras trabaja fuera de su residencia?	radio (Matrix)<br>1 Todo el tiempo (100%)<br>2 La mayor parte del tiempo (75%)<br>3 La mitad del tiempo (50%)<br>4 Menos de la mitad del tiempo (25%)<br>5 nunca (0%)<br>6 Nunca

2142	[hh11_work9_s_q2]<br><br>Show the field ONLY if: [language_q2] = '2' and ([hh11_work7_s_q2] = '1' or [hh11_work7_s_q2] = '2' or [hh11_work7_s_q2] = '3' or [hh11_work7_s_q2] = '4')	Persona 11: Actualmente, ¿con qué frecuencia se encuentra esta persona físicamente cerca de sus clientes mientras trabaja fuera de su residencia	radio (Matrix)<br>1 Todo el tiempo (100%)<br>2 La mayor parte del tiempo (75%)<br>3 La mitad del tiempo (50%)<br>4 Menos de la mitad del tiempo (25%)<br>5 nunca (0%)<br>6 Nunca
2143	[hh11_covidvaccine_s_q2]<br><br>Show the field ONLY if: [language_q2] = '2' and [hhcount_s_q2] > 10 and [hhcount_s_q2] < 13	Persona 11: ¿Piensa esta persona vacunarse contra la COVID-19?	radio<br>1 Sí<br>0 No<br>2 No sabe<br>3 Este individuo ya ha sido vacunado<br><br>Field Annotation: @DEFAULT="[hh11_covidvaccine_s_q2]"
2144	[hh11_covidsymp_s_q2]<br><br>Show the field ONLY if: [language_q2] = '2' and [hhcount_s_q2] > 10 and [hhcount_s_q2] < 13	Persona 11: En las últimas dos semanas, ¿ha tenido esta persona algún síntoma de COVID-19 (tos, fiebre, dificultad para respirar, fatiga, dolores de cuerpo, diarrea, goteo nasal o pérdida del sentido del olfato o del gusto)?	radio<br>1 Sí<br>0 No<br>2 No sabe
2145	[hh11_covidsymp2_s_q2]<br><br>Show the field ONLY if: [language_q2] = '2' and [hh11_covidsymp_s_q2] = '1'	Persona 11: ¿Cuándo le comenzaron los síntomas de COVID-19 a esta persona?	text (date_mdy)
2146	[hh11_covidsymp3_s_q2]<br><br>Show the field ONLY if: [language_q2] = '2' and [hh11_covidsymp_s_q2] = '1'	Persona 11: En vista de sus síntomas, ¿le preocupa a esta persona la posibilidad de tener COVID-19?	radio<br>1 Sí<br>0 No<br>2 No sabe
2147	[hh11_covidsymp4_s_q2]<br><br>Show the field ONLY if: [language_q2] = '2' and [hh11_covidsymp_s_q2] = '1'	Persona 11: ¿Fue esta persona objeto de prejuicio o discriminación debido a sus síntomas?	radio<br>1 Sí<br>0 No<br>2 No sabe
2148	[hh11_covidsymp5_s_q2]<br><br>Show the field ONLY if: [language_q2] = '2' and [hh11_covidsymp_s_q2] = '1'	Persona 11: ¿Qué hizo esta persona en vista de sus síntomas?<br>*(Seleccione todo lo que corresponda.)*	checkbox<br>0 hh11_covidsymp5_s_q2___0 Nada<br>1 hh11_covidsymp5_s_q2___1 Tomó medicamen[illegible] sin receta (ibuprofen[illegible] acetaminof[illegible] etc.)

			2 | hh11_covidsymp5_s_q2___2 | Consultó p[illegible] teléfono a [illegible] proveedor [illegible] asistencia médica<br>3 | hh11_covidsymp5_s_q2___3 | Fue al consultorio [illegible] un proveed[illegible] de asistenci[illegible] médica<br>4 | hh11_covidsymp5_s_q2___4 | Fue a una clínica o un[illegible] farmacia minorista<br>5 | hh11_covidsymp5_s_q2___5 | Fue a un centro de urgencias (FASTMed, etc.)<br>6 | hh11_covidsymp5_s_q2___6 | Fue a la sal[illegible] de emergencia[illegible]<br>7 | hh11_covidsymp5_s_q2___7 | Fue ingres[illegible] al hospital<br>8 | hh11_covidsymp5_s_q2___8 | Otra cosa<br>9 | hh11_covidsymp5_s_q2___9 | No sabe
2149	[hh11_covidsymp6_s_q2]<br><br>Show the field ONLY if: [language_q2] = '2' and [hh11_covidsymp5_s_q2(8)] = '1'	Persona 11: ¿Qué otra cosa hizo esta persona en vista de sus síntomas?	text
2150	[hh11_covidsymp7_s_q2]<br><br>Show the field ONLY if: [language_q2] = '2' and ([hh11_covidsymp5_s_q2(2)] = '1' or [hh11_covidsymp5_s_q2(3)] = '1' or [hh11_covidsymp5_s_q2(4)] = '1' or [hh11_covidsymp5_s_q2(5)] = '1' or [hh11_covidsymp5_s_q2(6)] = '1' or [hh11_covidsymp5_s_q2(7)] = '1' or [hh11_covidsymp5_s_q2(8)] = '1')	Persona 11: ¿Le dijo un proveedor de asistencia médica a esta persona que era posible que tuviera COVID-19?	radio<br>1 | Sí<br>0 | No<br>2 | No sabe
2151	[hh11_covid_test_s_q2]<br><br>Show the field ONLY if:	Persona 11: Si a esta persona le hicieron una prueba de COVID-19 en vista de sus síntomas, ¿cuál fue el resultado?	radio<br>1 | Pendiente<br>2 | Positivo

	[language_q2] = '2' and [hh11_covidsymp_s_q2] = '1'		3 Negativo<br>4 No concluyente<br>5 No se hizo la prueba<br>6 No sabe
2152	[hh11_covid_admit_s_q2]<br><br>Show the field ONLY if: [language_q2] = '2' and [hh11_covidsymp5_s_q2(7)] = '1'	Persona 11: ¿Cuántos días estuvo hospitalizada esta persona?	text (number, Min: 0)
2153	[hh11_covid_admit2_s_q2]<br><br>Show the field ONLY if: [language_q2] = '2' and [hh11_covidsymp5_s_q2(7)] = '1'	Persona 11: ¿Se le hicieron a esta persona las siguientes intervenciones durante su hospitalización?	checkbox<br>1 hh11_covid_admit2_s_q2___1 Oxígeno adiciona[illegible] por la nari[illegible]<br>2 hh11_covid_admit2_s_q2___2 Tratamien[illegible] en la Unidad de Cuidados Intensivos (Intensive Care Unit, ICU)<br>3 hh11_covid_admit2_s_q2___3 Ventilació[illegible] mecánica (intubació[illegible] o tubo de respiració[illegible]<br>4 hh11_covid_admit2_s_q2___4 No sabe
2154	[hh11_covidsymp8_s_q2]<br><br>Show the field ONLY if: [language_q2] = '2' and [hh11_covidsymp_s_q2] = '1'	Persona 11: ¿Ha vuelto esta persona a su salud normal?	radio<br>1 Sí<br>0 No<br>2 No sabe
2155	[hh11_prevent_s_q2]<br><br>Show the field ONLY if: [language_q2] = '2' and [hh11_covidsymp_s_q2] = '1'	Persona 11: ¿Cuáles de las siguientes medidas tomó esta persona para proteger a sus amigos y familiares después de que comenzaron sus síntomas?	checkbox<br>1 hh11_prevent_s_q2___1 Ponerse mascarilla con mayor frecuencia<br>2 hh11_prevent_s_q2___2 Lavarse las manos con agua y jabón con mayor frecuencia<br>3 hh11_prevent_s_q2___3 Limpiarse las manos con desinfectante con mayor frecuencia

			4 hh11_prevent_s_q2___4 Aislarse en su casa con mayor frecuencia<br>5 hh11_prevent_s_q2___5 Quedarse en casa con mayor frecuencia<br>6 hh11_prevent_s_q2___6 Ponerse guantes desechables con mayor frecuencia<br>7 hh11_prevent_s_q2___7 No sabe
2156	[hh12_relationship_s_q2]<br><br>Show the field ONLY if: [language_q2] = '2' and [hhcount_s_q2] > 11 and [hhcount_s_q2] < 13	Section Header: *Por cada persona adicional en su hogar, proporcione la siguiente informacion.*<br><br>Persona 12: ¿Cuál es su relación con esta persona?	radio<br>1 Pareja o cónyuge<br>2 Hijo o hija<br>3 Padre o madre<br>4 Hermano o hermana<br>5 Otro familiar<br>6 Proveedor de cuidado infantil u otros cuidados en casa<br>7 Otra<br><br>Field Annotation: @DEFAULT="[hh12_relationship_s_q2]"
2157	[hh12_relationship2_s_q2]<br><br>Show the field ONLY if: [language_q2] = '2' and [hh12_relationship_s_q2] = '7'	Persona 12: Especifique su relación con esta persona.	text<br>Field Annotation: @DEFAULT="[hh12_relationship2_s_q2]"
2158	[hh12_age_s_q2]<br><br>Show the field ONLY if: [language_q2] = '2' and [hhcount_s_q2] > 11 and [hhcount_s_q2] < 13	Persona 12: ¿Qué edad tiene esta persona? *(Especifique la edad en años)*	text (number, Min: 0, Max: 110)<br>Field Annotation: @DEFAULT="[hh12_age_s_q2]"
2159	[hh12_s_q2ex_s_q2]<br><br>Show the field ONLY if: [language_q2] = '2' and [hhcount_s_q2] > 11 and [hhcount_s_q2] < 13	Persona 12: ¿Cuál es el sexo de esta persona?	radio<br>1 Femenino<br>2 Masculino<br><br>Field Annotation: @DEFAULT="[hh12_s_q2ex_s_q2]"
2160	[hh12_race_s_q2]<br><br>Show the field ONLY if: [language_q2] = '2' and [hhcount_s_q2] > 11 an	Persona 12: ¿Cuál es la raza de esta persona? *(Seleccione todo lo que corresponda.)*	checkbox<br>1 hh12_race_s_q2___1 Indio americano o nativo de Alaska<br>2 hh12_race_s_q2___2 Asiático

	d [hhcount_s_q2] < 13		3 hh12_race_s_q2___3 Negro o afroestadounidens<br>4 hh12_race_s_q2___4 Nativo de Hawái o de las islas del Pacífico<br>5 hh12_race_s_q2___5 Blanco<br>6 hh12_race_s_q2___6 Otra<br>7 hh12_race_s_q2___7 No sabe<br><br>Field Annotation: @DEFAULT="[hh12_race_s_q2]"
2161	[hh12_ethn_s_q2]<br><br>Show the field ONLY if: [language_q2] = '2' and [hhcount_s_q2] > 11 and [hhcount_s_q2] < 13	Persona 12: ¿Cuál es la identidad étnica de esta persona?	radio<br>1 Hispano o latino<br>2 Ni hispano ni latino<br>3 Otra<br>4 No sabe<br><br>Field Annotation: @DEFAULT="[hh12_ethn_s_q2]"
2162	[hh12_edu_s_q2]<br><br>Show the field ONLY if: [language_q2] = '2' and [hhcount_s_q2] > 11 and [hhcount_s_q2] < 13	Persona 12: ¿Cuál es el nivel de educación de esta persona?	radio<br>1 No tiene educación formal<br>2 Kínder a 8° grado<br>3 Estudios de educación secundaria<br>4 Equivalencia de educación secundaria (GED)<br>5 Diploma de educación secundaria<br>6 Estudios de educación superior<br>7 Título universitario<br>8 Estudios de posgrado o más<br>9 No sabe<br><br>Field Annotation: @DEFAULT="[hh12_edu_s_q2]"
2163	[hh12_work_s_q2]<br><br>Show the field ONLY if: [language_q2] = '2' and [hhcount_s_q2] > 11 and [hhcount_s_q2] < 13	Persona 12: ¿Cuál de las siguientes es la mejor descripción de la situación laboral actual de esta persona?	radio<br>1 Empleo a tiempo completo<br>2 Empleo a tiempo parcial<br>3 En busca de empleo<br>4 Jubilado<br>5 Ama de casa<br>6 Estudiante<br>7 Permiso de maternidad o paternidad<br>8 Permiso por enfermedad

			9 Desempleado por discapacidad<br>10 Otra<br>11 No sabe<br><br>Field Annotation: @DEFAULT="[hh12_work_s_q2]"
2164	[hh12_work2_s_q2]<br><br>Show the field ONLY if: [language_q2] = '2' and [hhcount_s_q2] > 11 and [hhcount_s_q2] < 13	Persona 12: ¿Se considera esta persona actualmente empleado por cuenta propia (contratista independiente, trabajador esporádico (gig), etc.)?	radio<br>1 Sí<br>0 No<br>2 No sabe<br><br>Field Annotation: @DEFAULT="[hh12_work2_s_q2]"
2165	[hh12_work3_s_q2]<br><br>Show the field ONLY if: [language_q2] = '2' and [hhcount_s_q2] > 11 and [hhcount_s_q2] < 13 and ([hh12_work_s_q2] = '1' or [hh12_work_s_q2] = '2' or [hh12_work2_s_q2] = '1')	Persona 12: ¿Trabaja esta persona actualmente en alguno de los siguientes ambientes de alto riesgo de contagio de COVID-19?	checkbox<br>1 hh12_work3_s_q2___1 Ambiente de asistencia médica (hospital, clínica, centro de urgencias, etc.)<br>2 hh12_work3_s_q2___2 Ambiente residencial denso (hogar de ancianos, otro centro de asistencia de larga duración)<br>3 hh12_work3_s_q2___3 Prisión o cárcel<br>4 hh12_work3_s_q2___4 Establecimiento de envasado de carne<br>5 hh12_work3_s_q2___5 Establecimiento de envío o distribución<br>6 hh12_work3_s_q2___6 Establecimiento minorista de alto volumen (tienda de provisiones, etc.)<br>7 hh12_work3_s_q2___7 No sabe<br><br>Field Annotation: @DEFAULT="[hh12_work3_s_q2]"
2166	[hh12_work4_s_q2]<br><br>Show the field ONLY if: [language_q2] = '2' and [hhcount_s_q2] > 11 an	Persona 12: ¿Le ofrece el empleador a esta persona alguno de los siguientes beneficios en su empleo principal actual?<br>*(Seleccione todo lo que corresponda.)*	checkbox

	d [hhcount_s_q2] < 13 and ([hh12_work_s_q2] = '1' or [hh12_work_s_q2] = '2' or [hh12_work2_s_q2] = '1')		1 hh12_work4_s_q2___1 Permiso por enfermedad con goce de sueldo<br>2 hh12_work4_s_q2___2 Vacaciones o permiso personal con goce de sueldo<br>3 hh12_work4_s_q2___3 Seguro de salud<br>4 hh12_work4_s_q2___4 Seguro de discapacidad<br>5 hh12_work4_s_q2___5 Plan de jubilación<br>6 hh12_work4_s_q2___6 Otro<br>7 hh12_work4_s_q2___7 No sabe<br><br>Field Annotation: @DEFAULT="[hh12_work4_s_q2]"
2167	[hh12_work5_s_q2]<br><br>Show the field ONLY if: [language_q2] = '2' and [hhcount_s_q2] > 11 and [hhcount_s_q2] < 13 and ([hh12_work_s_q2] = '1' or [hh12_work_s_q2] = '2' or [hh12_work2_s_q2] = '1')	Persona 12: En una escala de 0 (definitivamente no va a suceder) a 10 (definitivamente va a suceder), ¿qué tan probable es que esta persona pierda su empleo debido a la pandemia de COVID-19?	text (number, Min: 0, Max: 10)<br>Field Annotation: @DEFAULT="[hh12_work5_s_q2]"
2168	[hh12_work6_s_q2]<br><br>Show the field ONLY if: [language_q2] = '2' and [hhcount_s_q2] > 11 and [hhcount_s_q2] < 13 and ([hh12_work_s_q2] = '1' or [hh12_work_s_q2] = '2' or [hh12_work2_s_q2] = '1')	Persona 12: En una escala de 0 (definitivamente no va a suceder) a 10 (definitivamente va a suceder), ¿qué tan probable es que a esta persona se le asignen menos horas de trabajo debido a la pandemia de COVID-19?	text (number, Min: 0, Max: 10)<br>Field Annotation: @DEFAULT="[hh12_work6_s_q2]"
2169	[hh12_work7_s_q2]<br><br>Show the field ONLY if: [language_q2] = '2' and [hhcount_s_q2] > 11 and [hhcount_s_q2] < 13 and ([hh12_work_s_q2] = '1' or [hh12_work_s_q2] = '2' or [hh12_work2_s_q2] = '1')	Persona 12: Actualmente ¿con qué frecuencia se le exige a esta persona que trabaje fuera de su residencia?	radio (Matrix)<br>1 Todo el tiempo (100%)<br>2 La mayor parte del tiempo (75%)<br>3 La mitad del tiempo (50%)<br>4 Menos de la mitad del tiempo (25%)<br>5 nunca (0%)<br>6 Nunca
2170	[hh12_work8_s_q2]<br><br>Show the field ONLY if:	Persona 12: Actualmente, ¿con qué frecuencia se encuentra esta persona físicamente cerca de sus	radio (Matrix)<br>1 Todo el tiempo (100%)

	[language_q2] = '2' and ([hh12_work7_s_q2] = '1' or [hh12_work7_s_q2] = '2' or [hh12_work7_s_q2] = '3' or [hh12_work7_s_q2] = '4')	compañeros de trabajo mientras trabaja fuera de su residencia?	2 La mayor parte del tiempo (75%)<br>3 La mitad del tiempo (50%)<br>4 Menos de la mitad del tiempo (25%)<br>5 nunca (0%)<br>6 Nunca
2171	[hh12_work9_s_q2]<br><br>Show the field ONLY if: [language_q2] = '2' and ([hh12_work7_s_q2] = '1' or [hh12_work7_s_q2] = '2' or [hh12_work7_s_q2] = '3' or [hh12_work7_s_q2] = '4')	Persona 12: Actualmente, ¿con qué frecuencia se encuentra esta persona físicamente cerca de sus clientes mientras trabaja fuera de su residencia	radio (Matrix)<br>1 Todo el tiempo (100%)<br>2 La mayor parte del tiempo (75%)<br>3 La mitad del tiempo (50%)<br>4 Menos de la mitad del tiempo (25%)<br>5 nunca (0%)<br>6 Nunca
2172	[hh12_covidvaccine_s_q2]<br><br>Show the field ONLY if: [language_q2] = '2' and [hhcount_s_q2] > 11 and [hhcount_s_q2] < 13	Persona 12: ¿Piensa esta persona vacunarse contra la COVID-19?	radio<br>1 Sí<br>0 No<br>2 No sabe<br>3 Este individuo ya ha sido vacunado<br><br>Field Annotation: @DEFAULT="[hh12_covidvaccine_s_q2]"
2173	[hh12_covidsymp_s_q2]<br><br>Show the field ONLY if: [language_q2] = '2' and [hhcount_s_q2] > 11 and [hhcount_s_q2] < 13	Persona 12: En las últimas dos semanas, ¿ha tenido esta persona algún síntoma de COVID-19 (tos, fiebre, dificultad para respirar, fatiga, dolores de cuerpo, diarrea, goteo nasal o pérdida del sentido del olfato o del gusto)?	radio<br>1 Sí<br>0 No<br>2 No sabe
2174	[hh12_covidsymp2_s_q2]<br><br>Show the field ONLY if: [language_q2] = '2' and [hh12_covidsymp_s_q2] = '1'	Persona 12: ¿Cuándo le comenzaron los síntomas de COVID-19 a esta persona?	text (date_mdy)
2175	[hh12_covidsymp3_s_q2]<br><br>Show the field ONLY if: [language_q2] = '2' and [hh12_covidsymp_s_q2] = '1'	Persona 12: En vista de sus síntomas, ¿le preocupa a esta persona la posibilidad de tener COVID-19?	radio<br>1 Sí<br>0 No<br>2 No sabe
2176	[hh12_covidsymp4_s_q2]<br><br>Show the field ONLY if: [language_q2] = '2' and [hh12_covidsymp_s_q2] = '1'	Persona 12: ¿Fue esta persona objeto de prejuicio o discriminación debido a sus síntomas?	radio<br>1 Sí<br>0 No<br>2 No sabe

2177	[hh12_covidsymp5_s_q2]<br>Show the field ONLY if:<br>[language_q2] = '2' and [hh12_covidsymp_s_q2] = '1'	Persona 12: ¿Qué hizo esta persona en vista de sus síntomas?<br>*(Seleccione todo lo que corresponda.)*	checkbox<br>0 hh12_covidsymp5_s_q2___0 Nada<br>1 hh12_covidsymp5_s_q2___1 Tomó medicamen[illegible] sin receta (ibuprofen[illegible] acetaminof[illegible] etc.)<br>2 hh12_covidsymp5_s_q2___2 Consultó p[illegible] teléfono a [illegible] proveedor [illegible] asistencia médica<br>3 hh12_covidsymp5_s_q2___3 Fue al consultorio [illegible] un proveed[illegible] de asistenci[illegible] médica<br>4 hh12_covidsymp5_s_q2___4 Fue a una clínica o un[illegible] farmacia minorista<br>5 hh12_covidsymp5_s_q2___5 Fue a un centro de urgencias (FASTMed[illegible] etc.)<br>6 hh12_covidsymp5_s_q2___6 Fue a la sal[illegible] de emergencia[illegible]<br>7 hh12_covidsymp5_s_q2___7 Fue ingres[illegible] al hospital<br>8 hh12_covidsymp5_s_q2___8 Otra cosa<br>9 hh12_covidsymp5_s_q2___9 No sabe
2178	[hh12_covidsymp6_s_q2]<br>Show the field ONLY if:<br>[language_q2] = '2' and [hh12_covidsymp5_s_q2(8)] = '1'	Persona 12: ¿Qué otra cosa hizo esta persona en vista de sus síntomas?	text
2179	[hh12_covidsymp7_s_q2]<br>Show the field ONLY if:<br>[language_q2] = '2' and ([hh12_covidsymp5_s_q2(2)] = '1' or [hh12_covidsymp5_s_q2(3)] = '1' or [hh12_covidsymp5_s_q2(4)] = '1' or [hh12_covidsymp5_s_q2(5)] = '1' or [hh12_covidsymp5_s	Persona 12: ¿Le dijo un proveedor de asistencia médica a esta persona que era posible que tuviera COVID-19?	radio<br>1 Sí<br>0 No<br>2 No sabe

	_q2(6)] = '1' or [hh12_covidsymp5_s_q2(7)] = '1' or [hh12_covidsymp5_s_q2(8)] = '1')		
2180	[hh12_covid_test_s_q2]<br><br>Show the field ONLY if: [language_q2] = '2' and [hh12_covidsymp_s_q2] = '1'	Persona 12: Si a esta persona le hicieron una prueba de COVID-19 en vista de sus síntomas, ¿cuál fue el resultado?	radio<br>1 Pendiente<br>2 Positivo<br>3 Negativo<br>4 No concluyente<br>5 No se hizo la prueba<br>6 No sabe
2181	[hh12_covid_admit_s_q2]<br><br>Show the field ONLY if: [language_q2] = '2' and [hh12_covidsymp5_s_q2(7)] = '1'	Persona 12: ¿Cuántos días estuvo hospitalizada esta persona?	text (number, Min: 0)
2182	[hh12_covid_admit2_s_q2]<br><br>Show the field ONLY if: [language_q2] = '2' and [hh12_covidsymp5_s_q2(7)] = '1'	Persona 12: ¿Se le hicieron a esta persona las siguientes intervenciones durante su hospitalización?	checkbox<br>1 hh12_covid_admit2_s_q2___1 Oxígeno adiciona[illegible] por la nari[illegible]<br>2 hh12_covid_admit2_s_q2___2 Tratamien[illegible] en la Unidad de Cuidados Intensivos (Intensive Care Unit, ICU)<br>3 hh12_covid_admit2_s_q2___3 Ventilació[illegible] mecánica (intubació[illegible] o tubo de respiració[illegible]<br>4 hh12_covid_admit2_s_q2___4 No sabe
2183	[hh12_covidsymp8_s_q2]<br><br>Show the field ONLY if: [language_q2] = '2' and [hh12_covidsymp_s_q2] = '1'	Persona 12: ¿Ha vuelto esta persona a su salud normal?	radio<br>1 Sí<br>0 No<br>2 No sabe
2184	[hh12_prevent_s_q2]<br><br>Show the field ONLY if: [language_q2] = '2' and [hh12_covidsymp_s_q2] = '1'	Persona 12: ¿Cuáles de las siguientes medidas tomó esta persona para proteger a sus amigos y familiares después de que comenzaron sus síntomas?	checkbox<br>1 hh12_prevent_s_q2___1 Ponerse mascarilla con mayor frecuencia<br>2 hh12_prevent_s_q2___2 Lavarse las manos con agua y jabón

			con mayor frecuencia<br>3 hh12_prevent_s_q2___3 Limpiarse las manos con desinfectante con mayor frecuencia<br>4 hh12_prevent_s_q2___4 Aislarse en su casa con mayor frecuencia<br>5 hh12_prevent_s_q2___5 Quedarse en casa con mayor frecuencia<br>6 hh12_prevent_s_q2___6 Ponerse guantes desechables con mayor frecuencia<br>7 hh12_prevent_s_q2___7 No sabe
2185	[serious1_s_q2]<br>Show the field ONLY if: [language_q2] = '2'	Section Header: *Complete la siguiente informacion sobre su salud mental y bienestar*<br>A su juicio, ¿qué tan grave es para usted personalmente la pandemia de COVID-19 en este momento?	radio<br>1 Muy grave<br>2 Más o menos grave<br>3 No demasiado grave<br>4 Nada grave
2186	[serious2_s_q2]<br>Show the field ONLY if: [language_q2] = '2'	A su juicio, ¿qué tan grave es actualmente la pandemia de COVID-19 para los integrantes de su comunidad?	radio<br>1 Muy grave<br>2 Más o menos grave<br>3 No demasiado grave<br>4 Nada grave
2187	[serious3_s_q2]<br>Show the field ONLY if: [language_q2] = '2'	A su juicio, ¿qué tan grave es actualmente la pandemia de COVID-19 para los habitantes de Estados Unidos?	radio<br>1 Muy grave<br>2 Más o menos grave<br>3 No demasiado grave<br>4 Nada grave
2188	[serious4_s_q2]<br>Show the field ONLY if: [language_q2] = '2'	A su juicio, ¿qué tan grave es actualmente la pandemia de COVID-19 para los habitantes de todo el mundo?	radio<br>1 Muy grave<br>2 Más o menos grave<br>3 No demasiado grave<br>4 Nada grave
2189	[racism1_s_q2]<br>Show the field ONLY if: [language_q2] = '2'	En las últimas dos semanas, ¿con qué frecuencia ha encontrado usted medios de comunicación, hechos o tendencias virales, conversaciones en	radio<br>1 Nunca<br>2 Aproximadamente una vez al mes

		línea, fotos o videos en línea en los cuales una persona como usted es amenazada o lastimada?	3 Aproximadamente una vez a la semana<br>4 Aproximadamente una vez al día<br>5 Varias veces al día
2190	[racism2_s_q2]<br>Show the field ONLY if:<br>[language_q2] = '2'	En las últimas dos semanas, ¿con qué frecuencia ha encontrado usted medios de comunicación, hechos o tendencias virales, conversaciones en línea, fotos o videos en línea en los cuales se presentan estereotipos negativos de personas como usted como si fueran verdaderos?	radio<br>1 Nunca<br>2 Aproximadamente una vez al mes<br>3 Aproximadamente una vez a la semana<br>4 Aproximadamente una vez al día<br>5 Varias veces al día
2191	[concern1_s_q2]<br>Show the field ONLY if:<br>[language_q2] = '2'	Section Header: *La pandemia de COVID-19 puede cuasar desafios por algunas personas sin importar a que esten infectados. Que tan preocupado/a esta usted sobre cada una de la siguiente?*<br>Recibir la asistencia médica que necesita (incluyendo la asistencia de salud mental)	radio (Matrix)<br>1 Nada<br>2 No mucho<br>3 Un poco<br>4 Mucho
2192	[concern2_s_q2]<br>Show the field ONLY if:<br>[language_q2] = '2'	Tener un lugar para vivir	radio (Matrix)<br>1 Nada<br>2 No mucho<br>3 Un poco<br>4 Mucho
2193	[concern3_s_q2]<br>Show the field ONLY if:<br>[language_q2] = '2'	Poder interactuar con otras personas	radio (Matrix)<br>1 Nada<br>2 No mucho<br>3 Un poco<br>4 Mucho
2194	[concern4_s_q2]<br>Show the field ONLY if:<br>[language_q2] = '2'	Conseguir comida, agua y otros suministros domésticos	radio (Matrix)<br>1 Nada<br>2 No mucho<br>3 Un poco<br>4 Mucho
2195	[concern5_s_q2]<br>Show the field ONLY if:<br>[language_q2] = '2'	Conseguir medicamentos	radio (Matrix)<br>1 Nada<br>2 No mucho<br>3 Un poco<br>4 Mucho
2196	[concern6_s_q2]<br>Show the field ONLY if:<br>[language_q2] = '2'	Tener transporte para llegar adonde tiene que ir	radio (Matrix)<br>1 Nada<br>2 No mucho

			3 Un poco<br>4 Mucho
2197	[concern7_s_q2]<br>Show the field ONLY if:<br>[language_q2] = '2'	Cuidar de su familia y a sus amigos	radio (Matrix)<br>1 Nada<br>2 No mucho<br>3 Un poco<br>4 Mucho
2198	[gad7_1_s_q2]<br>Show the field ONLY if:<br>[language_q2] = '2'	Section Header: *Durante las ultimas 2 semanas, que tan seguido ha tenido molestias debido a los siguientes problemas?*<br>Nerviosismo o ansiedad	radio (Matrix)<br>0 Nunca<br>1 Varios días<br>2 Más de la mitad de los días<br>3 Casi todos los días
2199	[gad7_2_s_q2]<br>Show the field ONLY if:<br>[language_q2] = '2'	Incapacidad de dejar de preocuparse o controlar las preocupaciones	radio (Matrix)<br>0 Nunca<br>1 Varios días<br>2 Más de la mitad de los días<br>3 Casi todos los días
2200	[gad7_3_s_q2]<br>Show the field ONLY if:<br>[language_q2] = '2'	Exceso de preocupación por diferentes cosas	radio (Matrix)<br>0 Nunca<br>1 Varios días<br>2 Más de la mitad de los días<br>3 Casi todos los días
2201	[gad7_4_s_q2]<br>Show the field ONLY if:<br>[language_q2] = '2'	Dificultad para tranquilizarse	radio (Matrix)<br>0 Nunca<br>1 Varios días<br>2 Más de la mitad de los días<br>3 Casi todos los días
2202	[gad7_5_s_q2]<br>Show the field ONLY if:<br>[language_q2] = '2'	Tanta inquietud que le es difícil estarse quieto	radio (Matrix)<br>0 Nunca<br>1 Varios días<br>2 Más de la mitad de los días<br>3 Casi todos los días
2203	[gad7_6_s_q2]<br>Show the field ONLY if:<br>[language_q2] = '2'	Facilidad para molestarse o irritarse	radio (Matrix)<br>0 Nunca<br>1 Varios días<br>2 Más de la mitad de los días<br>3 Casi todos los días
2204	[gad7_7_s_q2]	Temor de que algo horrible podría pasar	radio (Matrix)

	Show the field ONLY if: [language_q2] = '2'		0 Nunca<br>1 Varios días<br>2 Más de la mitad de los días<br>3 Casi todos los días
2205	[gad7_8_s_q2]<br><br>Show the field ONLY if: [language_q2] = '2' and ([gad7_1_s_q2] > 0 or [gad7_2_s_q2] > 0 or [gad7_3_s_q2] > 0 or [gad7_4_s_q2] > 0 or [gad7_5_s_q2] > 0 or [gad7_6_s_q2] > 0 or [gad7_7_s_q2] > 0)	¿Cuánta dificultad le han causado estos problemas para hacer su trabajo, ocuparse de las cosas de su casa o llevarse bien con los demás?	radio<br>0 Nada de dificultad<br>1 Un poco de dificultad<br>2 Bastante dificultad<br>3 Mucha dificultad
2206	[cesd_1_s_q2]<br><br>Show the field ONLY if: [language_q2] = '2'	Section Header: *Durante las ultimas 2 semanas, que tan seguido ha tenido los siguentes sentimientos y comportamientos?*<br><br>Me ha molestado lo que normalmente no me molesta.	radio (Matrix)<br>0 Rara vez o nunca (menos de 1 día)<br>1 Pocas veces (1-2 días)<br>2 De vez en cuando (3-4 días)<br>3 La mayor parte del tiempo o todo el tiempo (5-7 días)
2207	[cesd_2_s_q2]<br><br>Show the field ONLY if: [language_q2] = '2'	No he tenido ganas de comer; he tenido poco apetito.	radio (Matrix)<br>0 Rara vez o nunca (menos de 1 día)<br>1 Pocas veces (1-2 días)<br>2 De vez en cuando (3-4 días)<br>3 La mayor parte del tiempo o todo el tiempo (5-7 días)
2208	[cesd_3_s_q2]<br><br>Show the field ONLY if: [language_q2] = '2'	He sentido que no podía librarme de la tristeza, ni siquiera con la ayuda de mis familiares o amigos.	radio (Matrix)<br>0 Rara vez o nunca (menos de 1 día)<br>1 Pocas veces (1-2 días)<br>2 De vez en cuando (3-4 días)<br>3 La mayor parte del tiempo o todo el tiempo (5-7 días)
2209	[cesd_4_s_q2]<br><br>Show the field ONLY if: [language_q2] = '2'	He sentido que yo no soy tan bueno como otras personas.	radio (Matrix)<br>0 Rara vez o nunca (menos de 1 día)<br>1 Pocas veces (1-2 días)<br>2 De vez en cuando (3-4 días)<br>3 La mayor parte del tiempo o todo el tiempo (5-7 días)
2210	[cesd_5_s_q2]<br><br>Show the field ONLY if: [language_q2] = '2'	Me ha costado concentrarme en lo que hacía.	radio (Matrix)<br>0 Rara vez o nunca (menos de 1 día)<br>1 Pocas veces (1-2 días)<br>2 De vez en cuando (3-4 días)

			3 La mayor parte del tiempo o todo el tiempo (5-7 días)
2211	[cesd_6_s_q2]<br>Show the field ONLY if:<br>[language_q2] = '2'	Me he sentido deprimido.	radio (Matrix)<br>0 Rara vez o nunca (menos de 1 día)<br>1 Pocas veces (1-2 días)<br>2 De vez en cuando (3-4 días)<br>3 La mayor parte del tiempo o todo el tiempo (5-7 días)
2212	[cesd_7_s_q2]<br>Show the field ONLY if:<br>[language_q2] = '2'	He sentido que todo lo que hacía implicaba un gran esfuerzo.	radio (Matrix)<br>0 Rara vez o nunca (menos de 1 día)<br>1 Pocas veces (1-2 días)<br>2 De vez en cuando (3-4 días)<br>3 La mayor parte del tiempo o todo el tiempo (5-7 días)
2213	[cesd_8_s_q2]<br>Show the field ONLY if:<br>[language_q2] = '2'	He sentido desesperanza respecto al futuro.	radio (Matrix)<br>0 Rara vez o nunca (menos de 1 día)<br>1 Pocas veces (1-2 días)<br>2 De vez en cuando (3-4 días)<br>3 La mayor parte del tiempo o todo el tiempo (5-7 días)
2214	[cesd_9_s_q2]<br>Show the field ONLY if:<br>[language_q2] = '2'	He pensado que mi vida es un fracaso.	radio (Matrix)<br>0 Rara vez o nunca (menos de 1 día)<br>1 Pocas veces (1-2 días)<br>2 De vez en cuando (3-4 días)<br>3 La mayor parte del tiempo o todo el tiempo (5-7 días)
2215	[cesd_10_s_q2]<br>Show the field ONLY if:<br>[language_q2] = '2'	He tenido miedo.	radio (Matrix)<br>0 Rara vez o nunca (menos de 1 día)<br>1 Pocas veces (1-2 días)<br>2 De vez en cuando (3-4 días)<br>3 La mayor parte del tiempo o todo el tiempo (5-7 días)
2216	[cesd_11_s_q2]<br>Show the field ONLY if:<br>[language_q2] = '2'	No he dormido bien.	radio (Matrix)<br>0 Rara vez o nunca (menos de 1 día)<br>1 Pocas veces (1-2 días)<br>2 De vez en cuando (3-4 días)<br>3 La mayor parte del tiempo o todo el tiempo (5-7 días)
2217	[cesd_12_s_q2]<br>Show the field ONLY if:<br>[language_q2] = '2'	No he sido feliz.	radio (Matrix)<br>0 Rara vez o nunca (menos de 1 día)<br>1 Pocas veces (1-2 días)

			2 De vez en cuando (3-4 días)<br>3 La mayor parte del tiempo o todo el tiempo (5-7 días)
2218	[cesd_13_s_q2]<br>Show the field ONLY if: [language_q2] = '2'	He hablado menos de lo habitual.	radio (Matrix)<br>0 Rara vez o nunca (menos de 1 día)<br>1 Pocas veces (1-2 días)<br>2 De vez en cuando (3-4 días)<br>3 La mayor parte del tiempo o todo el tiempo (5-7 días)
2219	[cesd_14_s_q2]<br>Show the field ONLY if: [language_q2] = '2'	Me he sentido solo.	radio (Matrix)<br>0 Rara vez o nunca (menos de 1 día)<br>1 Pocas veces (1-2 días)<br>2 De vez en cuando (3-4 días)<br>3 La mayor parte del tiempo o todo el tiempo (5-7 días)
2220	[cesd_15_s_q2]<br>Show the field ONLY if: [language_q2] = '2'	Las personas han sido poco amistosas.	radio (Matrix)<br>0 Rara vez o nunca (menos de 1 día)<br>1 Pocas veces (1-2 días)<br>2 De vez en cuando (3-4 días)<br>3 La mayor parte del tiempo o todo el tiempo (5-7 días)
2221	[cesd_16_s_q2]<br>Show the field ONLY if: [language_q2] = '2'	No he disfrutado de la vida.	radio (Matrix)<br>0 Rara vez o nunca (menos de 1 día)<br>1 Pocas veces (1-2 días)<br>2 De vez en cuando (3-4 días)<br>3 La mayor parte del tiempo o todo el tiempo (5-7 días)
2222	[cesd_17_s_q2]<br>Show the field ONLY if: [language_q2] = '2'	He tenido episodios de llanto.	radio (Matrix)<br>0 Rara vez o nunca (menos de 1 día)<br>1 Pocas veces (1-2 días)<br>2 De vez en cuando (3-4 días)<br>3 La mayor parte del tiempo o todo el tiempo (5-7 días)
2223	[cesd_18_s_q2]<br>Show the field ONLY if: [language_q2] = '2'	Me he sentido triste.	radio (Matrix)<br>0 Rara vez o nunca (menos de 1 día)<br>1 Pocas veces (1-2 días)<br>2 De vez en cuando (3-4 días)<br>3 La mayor parte del tiempo o todo el tiempo (5-7 días)
2224	[cesd_19_s_q2]<br>Show the field ONLY if:	He sentido que no les agrado a los demás.	radio (Matrix)<br>0 Rara vez o nunca (menos de 1 día)

[language_q2] = '2'		1 Pocas veces (1-2 días)<br>2 De vez en cuando (3-4 días)<br>3 La mayor parte del tiempo o todo el tiempo (5-7 días)
2225 [cesd_20_s_q2]<br>Show the field ONLY if:<br>[language_q2] = '2'	No he podido "ponerme en marcha".	radio (Matrix)<br>0 Rara vez o nunca (menos de 1 día)<br>1 Pocas veces (1-2 días)<br>2 De vez en cuando (3-4 días)<br>3 La mayor parte del tiempo o todo el tiempo (5-7 días)
2226 [survey_s_q2]<br>Show the field ONLY if:<br>[language_q2] = '2'	¿Cómo llenó esta encuesta?	radio<br>1 En una computadora (portátil o de escritorio)<br>2 En un dispositivo móvil (tableta o teléfono celular)<br>3 Por teléfono con un entrevistador<br>4 Otra manera
2227 [c4_q2weeks_complete]	Section Header: *Form Status*<br>Complete?	dropdown<br>0 Incomplete<br>1 Unverified<br>2 Complete

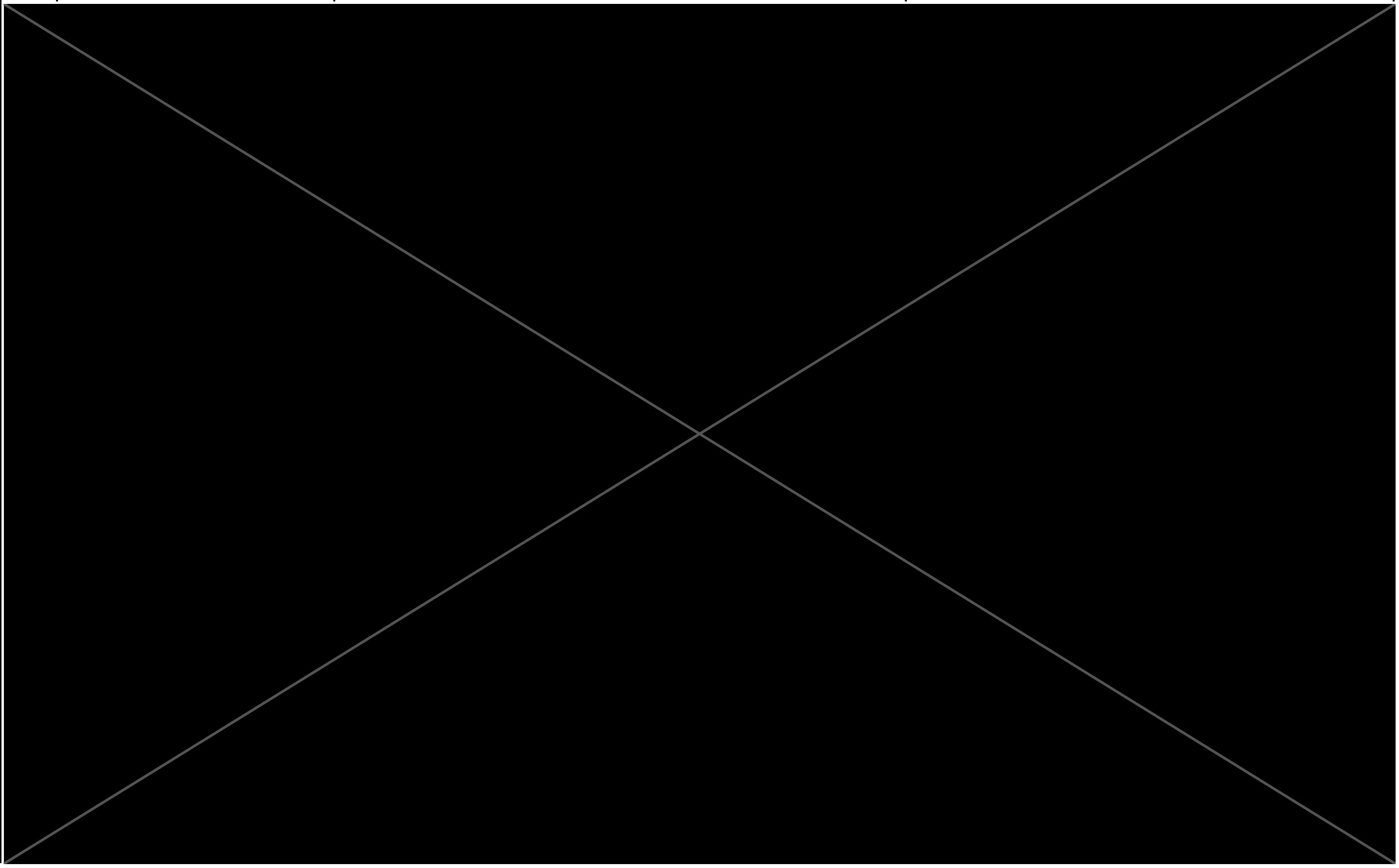

Supplement: S2 File — (PDF) [file pmen.0000410.s004.pdf]
