# Supplementary material for: The association between depressive symptoms and antibody response following SARS-CoV-2 vaccination among central North Carolina residents
Source: PLOS Ment Health. 2025 Sep 17;2(9):e0000410. doi: 10.1371/journal.pmen.0000410 (PMC12448651; doi:10.1371/journal.pmen.0000410)
Supplement: Attachment — Submitted filename: ResponseToReviewers_07MAY25.docx [file pmen.0000410.s005.docx]

May 6, 2025

Amin Tajerian, M.D.
Academic Editor
PLOS Mental Health

Dear Dr. Tajerian,

Thank you for the constructive review of our manuscript entitled, “**The association between depressive symptoms and antibody response following SARS-CoV-2 vaccination among central North Carolina residents**” (PMEN-D-25-00059). Please find our responses (in blue) to journal requirements as well as editor and reviewers’ comments below.

**Journal Requirements:**

1. We noticed you have some minor occurrence of overlapping text with the following previous publication(s), which needs to be addressed:

- <https://doi.org/10.3389/fimmu.2022.965971> 
- DOI: 10.1097/EDE.0000000000001625
- doi: 10.1136/bmj-2021-068302

In your revision ensure you cite all your sources (including your own works), and quote or rephrase any duplicated text outside the methods section. Further consideration is dependent on these concerns being addressed.

*In response to these concerns, we used iThenticate to search for matches in our text and these articles. For the first article, the match that was found is the in the first line of our abstract: "against SARS-CoV-2 infection and COVID-19". We replaced this sentence in our abstract to better align with the aim of our study.*

*For the second article, a few matches were identified since this article and the submitted manuscript were written by the same first author. We have revised all overlapping text outside of the methods section.*

*For the third article, the only matches flagged by iThenticate were keywords and references, so we are not sure what overlapping text was detected.*

*We diligently used iThenticate to check our revised manuscript against these three sources, as well as other sources to avoid overlapping text. We would be happy to respond to specific concerns regarding overlapping text if provided by the editorial office.*

2. Please amend your detailed Financial Disclosure statement. This is published with the article. It must therefore be completed in full sentences and contain the exact wording you wish to be published.

i. Please clarify all sources of funding (financial or material support) for your study. List the grants (with grant number) or organizations (with url) that supported your study, including funding received from your institution. 
ii. State the initials, alongside each funding source, of each author to receive each grant.
iii. State what role the funders took in the study. If the funders had no role in your study, please state: “The funders had no role in study design, data collection and analysis, decision to publish, or preparation of the manuscript.”
iv. If any authors received a salary from any of your funders, please state which authors and which funders.

*Updated.*

3. Please make sure the funding information on the submission form matches your financial disclosure statement. Please indicate by return the full and correct funding information for your study and confirm the order in which funding contributions should appear. Please be sure to indicate whether the funders played any role in the study design, data collection and analysis, decision to publish, or preparation of the manuscript.

*Updated.*

4. Please insert an Ethics Statement at the beginning of your Methods section, under a subheading 'Ethics Statement'.

*Updated.*

5. Please provide separate figure files in .tif or .eps format.

For more information about figure files please see our guidelines:

<https://journals.plos.org/mentalhealth/s/figures> 
<https://journals.plos.org/mentalhealth/s/figures#loc-file-requirements>

*Updated.*

6. We have noticed that you have uploaded Supporting Information files, but you have not included a list of legends. Please add a full list of legends for your Supporting Information files after the references list.

*Included as attachment in submission ("Supplement.docx).*

7. In the online submission form, you indicated that “Deidentified individual data that supports the results will be shared beginning 9 to 36 months following publication upon request to the corresponding author, provided the investigator who proposes to use the data has approval from an Institutional Review Board (IRB), Independent Ethics Committee (IEC), or Research Ethics Board (REB), as applicable, and executes a data use/sharing agreement with UNC. Principal Investigator should submit a request to the UNC Industry Contracting team (OSPContracting@unc.edu) to initiate the data use/sharing agreement.”

All PLOS journals now require all data underlying the findings described in their manuscript to be freely available to other researchers, either 
1. In a public repository, 
2. Within the manuscript itself, or 
3. Uploaded as supplementary information.

This policy applies to all data except where public deposition would breach compliance with the protocol approved by your research ethics board. If your data cannot be made publicly available for ethical or legal reasons (e.g., public availability would compromise patient privacy), please explain your reasons by return email and your exemption request will be escalated to the editor for approval. Your exemption request will be handled independently and will not hold up the peer review process, but will need to be resolved should your manuscript be accepted for publication. One of the Editorial team will then be in touch if there are any issues.

*Email sent.*

**Editor Comments:**
**#1.** The CESD-20 is designed to assess current depressive symptomatology experienced over the past two weeks in the general population. However, it does not capture the chronicity or functional impairment necessary for a clinical diagnosis of Major Depressive Disorder (MDD). While the CESD-20 demonstrates high sensitivity, making it effective in identifying individuals with depressive symptoms, it has only moderate specificity. This means it may occasionally classify individuals without clinically significant depression as positive, leading to potential false positives. CESD-20 is useful for capturing short-term fluctuations in mood, making it well-suited for large-scale, cross-sectional studies. However, its limitations become more pronounced in longitudinal research or studies with small sample sizes, where the lack of information on symptom chronicity and functional impairment may reduce its utility and increase the risk of misclassification. Please discuss how the CESD-20’s focus on current depressive symptoms is relevant to your study and address validity, reliability, sensitivity, and specificity of using CESD-20 >16 cutoff in COVID19 era.

*Thank you for raising this important point. We now include the following paragraph in the discussion:*

*"The use of the CESD-20 has its own set of limitations. First, this analysis only accounts for CESD-20 scores during a single period, in the four weeks prior to vaccination. Depression scores at other points in time may be associated differently with the outcome. Further, the performance of the CESD-20 screening tool may not have the same reliability in all populations. In a meta-analysis of 28 studies, the CESD-20 with a cutoff ≥16 demonstrated higher pooled sensitivity than specificity (0.87 vs 0.70), highlighting that the tool will produce a greater number of false-positive than false-negative results. Higher cutoffs have been suggested (e.g. ≥20), which have increased specificity at the expensive of decreased sensitivity. Our study may be subject to both false-negative and false-positive classifications of depressive symptoms. Due to our small sample size, we were unable to evaluate other cutoffs, however, we reached similar conclusions with a dichotomized CESD-20 score and a continuous score."*

**#2.** The use of GEE with interaction terms may be too complex for the small subgroup.
*Thank you for this comment, this is something we had considered during the modeling phase of our analysis. However, since we leverage multiple imputation, we believe that we maintain a large enough sample size to include interaction terms in our longitudinal model. Without multiple imputation, we agree that this would likely be an issue.*

**#3.** Greater transparency in reporting statistical differences in IgG responses is recommended.

*We have now revised the results section to mention that the 95% confidence interval for the difference does not cross the null (Page 11). We prefer rely on tests of statistical significance, following guidelines from the American Statistical Association.*^1^

**#4.** Consider modeling CESD-20 as a continuous variable and report the effects of covariates used to adjust the IgG response.

*Thank you for this suggestion. We now report the results of modeling CESD-20 as a continuous variable in the results (Page 11) and include the effects of these covariates in a supplemental table.*

*We chose to emphasize only the main exposure effect estimates in the main text. Specifically, we did not report the effect estimates for other covariates to avoid the "Table 2 Fallacy".*^2^ *Because the covariates in the model were chosen using a directed acyclic graph (DAG), these covariates are confounders between the exposure and outcome. Their effect estimates should not be interpreted as causal relationships between themselves and the outcome.*

**#6.** Consider using a post hoc power analysis to objectively assess the impact of the small sample size on the study’s findings.

*We include a statement about the small sample size, and resulting imprecision, to the Discussion on Page 12. Based on recent guidance, we do not feel comfortable conducting a post hoc power analysis.*^3,4^

**Reviewer #1:**

**#1.** This study addresses an important and underexplored area of research, providing insights into how mental health factors may influence immune responses to vaccination. However, there are several areas where the study could be strengthened.
Statistical Power and Sample Size: Given the small number of participants with depressive symptoms (n=14), I suggest including a power calculation to assess whether the study is adequately powered to detect meaningful differences in antibody levels.

*Please see response to Editor comment #6 above.*

**#2.** Measurement of Depressive Symptoms: The use of the CESD-20 is appropriate, but I suggest considering an analysis where depressive symptoms are treated as a continuous variable or stratified by severity, rather than a strict binary cutoff (≥16), to provide a more nuanced understanding of the association.

*We now report the results of modeling CESD-20 as a continuous variable in the results (Page 11).*

*"In the secondary analysis, pre-vaccination continuous CESD-20 score was negatively associated with mean IgG (parameter estimate=-0.243, 95% CI=(-0.485, -0.001))."*

**#3.** Interpretation of Findings: The overlapping confidence intervals in IgG levels between groups indicate that the observed differences might be due to variability and limited sample size. I suggest discussing whether these differences have clinical relevance or are primarily a statistical finding.

*We have updated the first paragraph of the discussion to clarify that the clinical significance of this finding is unclear (Page 12).*

*"There is modest evidence that individuals with depressive symptoms have a smaller initial antibody response following vaccination compared to individuals without depressive symptoms, though the clinical relevance is unclear."*

**#4.** Missing Data and Imputation Strategy: Since multiple imputation was used for missing IgG values and covariates, I recommend providing more details about the assumptions made, how missingness was assessed, and whether sensitivity analyses were performed to ensure the robustness of the results.
*Thank you for this suggestion. We updated the methods to include more details about the missing data on Page 8.*

*"Missing outcomes (IgG values) and covariates were non-monotone and assumed to be missing at random (MAR). These were imputed via multiple imputation with fully conditional specification using 30 burn-in iterations and 30 imputations. The final imputation model included all confounding variables."*

**#5.** Data Visualization: Figure 2 could be improved by adding clearer annotations and possibly overlaying individual data points on the model-predicted means to better illustrate trends.
*Individual data points are (already) overlaid on the model-predicted means. We welcome further clarification upon the next review.*

**#6.** Discussion on Public Health Implications: Expanding the discussion to consider potential interventions (e.g., psychological or behavioral strategies to improve vaccine efficacy in individuals with depressive symptoms) would strengthen the paper’s impact.

*We have now added text to the Discussion about the public health implications of the paper, specifically emphasizing that differences in IgG response to the vaccine are small and may not impact vaccine efficacy (Page 15).*

**Reviewer #2:**

**#1.** Thank you for submitting your interesting manuscript to this journal.
Methods: Your study has a solid methodology, utilizing GEE and a reliable sampling method. You also explain the handling of missing data, which enhances the robustness of your work. My main concern is the disparity between your aim and methods compared to your results and discussion. I will address this in the following paragraphs.

*Thank you for your review of our study.*

**#2.** Results: Your demographic data is well described, although you did not include important baseline characteristics and comorbid conditions that could impact the immune response, such as diabetes mellitus, autoimmune diseases, immunosuppressant drug use, chemotherapy, and cancer. This bias is particularly critical in your study because your primary outcome is IgG, and your participants were predominantly older individuals.
*Thank you for this suggestion. We updated Table 1 to include counts and percentages for three comorbid conditions, including diabetes mellitus, cancer, and immunosuppression. Other variables suggested are unavailable in our data.*

**#3.** You did not report any follow-up on depressive symptoms in your results, which contradicts the methods section, where you mention a biweekly survey to evaluate depressive symptoms. Additionally, you highlight this repetitive depressive symptom measurement in both your strengths and limitations sections, which is inconsistent.

*Thank you for mentioning this, we realize that our manuscript was unclear. We do use follow-up of depressive symptoms in our analysis since the exposure in our model is an average of* *CESD-20 scores from all surveys completed within 4 weeks prior to vaccination.* *We have now revised a sentence in the discussion to clarify this on Page 13:*

*"Second, the study measured both IgG in serum and depressive symptoms via survey at regular intervals during follow-up, and we leverage longitudinal IgG and depressive symptom data in our models."*

*We also include the biweekly depressive symptoms score in the limitations because our model only accounts for depressive symptoms in the 4 weeks prior to vaccination, meaning that we did not assess depressive symptoms after vaccination, which is a limitation. This is distinct from the strengths mentioned above. We prefer to keep both mentions of the depressive symptoms survey as a strength and limitation in the discussion.*

**#4.** The analysis report is unclear. You did not explain why you treated the CESD-20 score as a binary exposure. Moreover, you did not report the p-value or effect size for your model.
*We now include a sentence in section 2.4 explaining why the CESD-20 cutoff of 16 was chosen (Page 6) and include results where CESD-20 is continuous (Page 11).*

*"This cutoff is frequently chosen to maintain relatively high sensitivity and specificity and is appropriate in most populations."*

*The effect size for the CESD-20-outcome association is now reported in the supplement. Hypothesis testing and p-values were intentionally omitted, following guidance from The American Statistical Association recommends against using p-values in many situations.*^1^

**#5.** Discussion: Attitudes and beliefs about vaccines are mentioned as a strength: “the frequency of surveys allows for monitoring of participant attitudes and beliefs towards vaccination”, yet there is no corresponding information about these surveys in the results or discussion sections.

*Thank you for mentioning this. The discussion of attitudes towards vaccination was removed and the sentence was re-written as:*

*"... the frequency of surveys captures changes in mental health indicators, including depression." (Page 13)*

**#6.** The section in the discussion about COVID-19 outcomes seems irrelevant—why was it included?

*We have now revised this paragraph to to clarify that the discussion of COVID-19 outcomes was included to emphasize the other impacts of depression or depressive symptoms on COVID-19 burden.*

*"The COVID-19 pandemic has highlighted several health-related disparities in certain populations. People with mental health disorders, including depression, are one such group that experiences worse COVID- 19 outcomes than others. These individuals face an increased risk of COVID-19 infection and mortality In this study, we found that depressive symptoms play a role in altering the initial response to COVID-19 vaccines. This aligns with the conclusions of previous studies that psychological stress and mental health disorders can alter initial vaccine response. Individuals with depressive symptoms face multifactorial burdens related to COVID-19."*

**#7.** Additionally, there is no conclusion section in your manuscript.

*The conclusions are in integrated into the discussion section.*

Kind regards,

Caitlin Cassidy

Doctoral Candidate

Department of Epidemiology

The University of North Carolina at Chapel Hill

*References:*

1.	Wasserstein RL, Schirm ,Allen L., and Lazar NA. Moving to a World Beyond “p < 0.05.” *The American Statistician*. 2019;73(sup1):1-19. doi:10.1080/00031305.2019.1583913

2.	Westreich D, Greenland S. The Table 2 Fallacy: Presenting and Interpreting Confounder and Modifier Coefficients. *American Journal of Epidemiology*. 2013;177(4):292-298. doi:10.1093/aje/kws412

3.	Griffith KN, Feyman Y. Amplifying the Noise: The Dangers of Post Hoc Power Analyses. *J Surg Res*. 2021;259:A9-A11. doi:10.1016/j.jss.2019.09.075

4.	Dziak JJ, Dierker LC, Abar B. The Interpretation of Statistical Power after the Data have been Gathered. *Curr Psychol*. 2020;39(3):870-877. doi:10.1007/s12144-018-0018-1
