# Supplementary material for: The association between depressive symptoms and antibody response following SARS-CoV-2 vaccination among central North Carolina residents
Source: PLOS Ment Health. 2025 Sep 17;2(9):e0000410. doi: 10.1371/journal.pmen.0000410 (PMC12448651; doi:10.1371/journal.pmen.0000410)
Supplement: Attachment — Submitted filename: ResponseToReviewers_29JUL25.docx [file pmen.0000410.s006.docx]

July 29, 2025

Amin Tajerian, M.D.
Academic Editor
PLOS Mental Health

Dear Dr. Tajerian,

Thank you for reviewing the revision of our manuscript entitled, “**The association between depressive symptoms and antibody response following SARS-CoV-2 vaccination among central North Carolina residents**” (PMEN-D-25-00059). Please find our responses (in blue).

**Editor Comments:**
**#1.** The statistical analysis, as presented, raises concerns regarding both integrity and transparency. Specifically, the claim of statistical significance at month 0 is not supported by the reported data. You state: "Note, the 95% confidence interval for the difference does not include the null (Table 2, Fig 2)."
However, the confidence interval for the group difference at month 0 is -3.55 (95% CI: -12.01, 4.90), which does include the null hypothesis value of zero. This corresponds to an approximate p-value of 0.41, indicating no statistically significant difference between groups at that time point.
Moreover, all other confidence intervals for group differences (at months 3 and 6) also include zero, suggesting that these comparisons are likewise not statistically significant or meaningful.
It should not be the reader’s responsibility to reconstruct the statistical logic or determine significance from confidence intervals. It is the authors' responsibility to present results accurately, clearly, and without misrepresentation. As currently written, the reporting of statistical findings lacks the clarity and rigor. I recommend revising the manuscript to reflect the correct interpretation of the data and ensure transparent communication of statistical findings.

*Thank you for reviewing our revised manuscript. We greatly appreciate the editor for pointing out this mistake, which reflects a typo rather than what we intended to say. We have removed the mentioned sentence and included a new sentence that reflects that the 95% CI for all differences includes the null: "Note, the 95% confidence intervals for the differences at months 0, 3, and 6 include the null (Table 2, Fig 2)." The sentence in the discussion is correct: "As such, the 95% confidence intervals for individuals with and without depressive symptoms overlap." and reflects that the results are not statistically significant.*

*However, we disagree with the idea that "comparisons are likewise not statistically significant or meaningful". Clinical or public health significance or meaning is not determined by statistical significance. The lack of statistical significance does not imply that the results are meaningless. This uncertainty about the clinical significance is reflected in our discussion: "There is modest evidence that individuals with depressive symptoms have a smaller initial antibody response following vaccination compared to individuals without depressive symptoms, though the clinical relevance is unclear."*

**Reviewer Comments:**

All comments have been addressed.

*We are grateful for the reviewers for taking the time to thoroughly examine our revised manuscript. We are glad that our revisions have clarified all prior comments/concerns.*

Kind regards,

Caitlin Cassidy

Doctoral Candidate

Department of Epidemiology

The University of North Carolina at Chapel Hill
